# Genomic aberrations in young and elderly  breast cancer patients

**DOI:** 10.1186/s12916-015-0504-3

**Published:** 2015-10-15

**Authors:** Hatem A. Azim, Bastien Nguyen, Sylvain Brohée, Gabriele Zoppoli, Christos Sotiriou

**Affiliations:** Breast Cancer Translational Research Laboratory, Institut Jules Bordet, Université Libre de Bruxelles, Boulevard de Waterloo, 121, Brussels, Belgium; Department of Internal Medicine, University of Genova and IRCCS AOU San Martino - IST, Genoa, Italy

**Keywords:** Age, Breast cancer biology, Breast cancer in the elderly, Breast cancer in young patients, GATA3, Gene expression, Mutations

## Abstract

**Background:**

Age at breast cancer diagnosis is a known prognostic factor. Previously, several groups including ours have shown that young age at diagnosis is associated with higher prevalence of basal-like tumors and aggressive tumor phenotypes. Yet the impact of age at diagnosis on the genomic landscape of breast cancer remains unclear. In this study, we examined the pattern of somatic mutations, chromosomal copy number variations (CNVs) and transcriptomic profiles in young and elderly breast cancer patients.

**Methods:**

Analyses were performed on The Cancer Genome Atlas (TCGA) dataset. Patients with metastatic disease at diagnosis, classified as normal-like by PAM50 or had missing clinical information were excluded. Young patients were defined as ≤45 years of age, while elderly patients were those ≥70 years of age at breast cancer diagnosis. The remaining patients were classified as “intermediate”. We evaluated the association between age at diagnosis and somatic mutations, CNV and gene expression in a logistic regression model adjusting for tumor size, nodal status, histology and breast cancer subtype. All analyses were corrected for multiple testing using the Benjamini–Hochberg approach.

**Results:**

In this study, 125, 486 and 169 patients were ≤45, 46–69 and ≥70 years of age, respectively. Older patients had more somatic mutations (n = 44 versus 35 versus 31; *P* = 0.0009) and more CNVs, especially in ductal tumors (*P* = 0.02). Eleven mutations were independently associated with age at diagnosis, of which only GATA3 was associated with young age (15.2 % versus 8.2 % versus 9 %; *P* = 0.003). Only two CNV events were independently associated with age, with more chr18p losses in older patients and more chr6q27 deletions in younger ones. Younger age at diagnosis was associated with higher expression of gene signatures related to proliferation, stem cell features and endocrine resistance.

**Conclusions:**

Age adds a layer of biological complexity beyond breast cancer molecular subtypes, classic pathological and clinical variables, worthy of further consideration in future drug development as we seek to refine therapeutic strategies in the era of personalized medicine.

**Electronic supplementary material:**

The online version of this article (doi:10.1186/s12916-015-0504-3) contains supplementary material, which is available to authorized users.

## Background

Young age at breast cancer diagnosis is a known poor prognostic factor [1, 2]. Previous studies have indicated higher prevalence of poorly differentiated, estrogen receptor (ER)-negative and human epidermal growth factor receptor 2 (HER2)-positive tumors in women diagnosed at a young age [3, 4]. Further genomic characterization has revealed enrichment with basal-like tumors [5, 6]. While these observations could well explain the poorer outcome of young breast cancer patients compared to their older counterparts, younger age remains an independent poor determinant of long-term outcome [5]. This underscores the need to further refine our understanding of the impact of age on cancer biology, which could have relevant implications on patient management.

On the other hand, few data are available with respect to the biological features of tumors arising in the elderly. Currently, around 30–35 % of breast cancer patients are over 70 years of age at the time of diagnosis and this is expected to increase in the coming years [7]. While these patients appear to develop relatively more “indolent” tumors characterized by high endocrine receptor expression [8], the late onset of these tumors may also suggest accumulation of several genomic aberrations over time, due to the stochastic nature of DNA damage in eukaryotic cells during the replication process. Acknowledging that morbidities other than cancer itself often contribute to mortality of older patients [9], it is very important to refine our understanding of the biology of these tumors in an attempt to optimize their management.

Previously, our group and others have published on the differences at the transcriptomic level according to age at diagnosis, investigating selected genes or pathways [5, 6, 10]. However, we lack studies that evaluate the differences at the DNA level. In the current study,we investigated for the first time the differences in somatic mutations and copy number variations (CNVs) between young and older breast cancer patients. In addition, we evaluated the expression of thousands of relevant genomic signatures at the RNA level.

## Methods

### Eligible patients

All analyses were performed on The Cancer Genome Atlas (TCGA) publicly available dataset. Eligible patients were those with non-metastatic disease who had complete information on age at breast cancer diagnosis, tumor histology, tumor size and lymph node status. For each patient, we determined the breast cancer molecular subtype using PAM50 [11]. PAM50 classes were determined from the TCGA RNA-Seq gene expression data using the genefu package of the R/Bioconductor statistical package. Samples of patients classified as normal-like were excluded, as they often represent an artifact due to limited tumor cellularity and a large background of normal breast cells in the sample [12].

Young patients were defined as ≤45 years of age, while elderly patients were defined as those ≥70 years of age at breast cancer diagnosis. The remaining patients were classified as “intermediate”. Since the TCGA dataset is publicly available, ethics committee approval was not needed. In addition, neither patient informed consent nor permission to use this data was required to perform this analysis.

### Genomic analysis

We evaluated three parameters: 1) somatic mutations using exome sequencing; 2) somatic CNV; and 3) transcriptomic profiles. We downloaded the data from the TCGA online repository in February 2015.

In the current analysis, all somatic mutations were considered apart from those referred to as “silent” mutations. Somatic CNV was evaluated using array comparative genomic hybridization (CGH) data, available as pre-processed, publicly available information and not validated by any other methodology. Segmented data were used as input for Genomic Identification of Significant Targets in Cancer, version 2.0 (GISTIC 2.0) and version 6.2 on the Broad Institute GenePattern cloud server to obtain somatic focal and broad CNV events [13]. These were then parsed in R. For focal events, only “high-level” focal amplification events, defined as log2 ratio >0.9 were retained, whereas focal losses were retained with log2 ratio >0.3 and with a *Q* value <0.25. Broad events, defined as arm-level events encompassing 98 % or more of a chromosome arm, were computed using GISTIC as well.

For transcriptomic profiling, we used the RNA sequencing data to evaluate differences in transcriptomic profiles according to age. Data were downloaded from the TCGA online repository and RNA-Seq absolute expression values were log2 transformed before performing the analyses.

### Statistical analyses

The association between age groups, that is, young (≤45 years), intermediate (46–49 years) and elderly patients (≥70 years), with clinicopathological characteristics was evaluated using Pearson’s chi-squared test. The Kruskal–Wallis test was used to compare the number of mutations and CNVs according to age group. For mutations that were represented in at least 5 % in any age group, we evaluated their independent association with age at diagnosis (as a continuous variable) in a logistic regression model adjusting for tumor size (≤2 cm versus >2 cm), nodal status (negative versus positive), tumor histology (ductal versus lobular) and breast cancer subtype (luminal-A versus luminal-B versus HER2 versus basal). A similar model was used to evaluate the independent association between age, CNV and gene expression using the Molecular Signatures Database (MSigDB; PMID: 16199517). All analyses were corrected for multiple testing using the Benjamini–Hochberg approach [14].

## Results

A total of 780 patients from the TCGA dataset where included, of whom 125, 486 and 169 were ≤45, 46–69 and ≥70 years of age, respectively. Transcriptomic data was available for all patients, while 722 (92.5 %) and 713 (91.4 %) had available somatic mutation and CNV data, respectively.

Table 1 summarizes the main characteristics of patients. As expected, young patients had less lobular cancer (7 % versus 24 % versus 29 %; *P* <0.001), fewer node-negative tumors (38 % versus 49 % versus 49 %; *P* = 0.05) and a trend of more basal-like tumors (20 % versus 18 % versus 14 %; *P* = 0.16).

**Table 1:** Main characteristics of patients

Characteristic	The Cancer Genome Atlas (N = 780)			
	≤45 years of age	46–69 years of age	≥70 years of age	*P* value
Number	125	486	169	
Tumor size				
≤2 cm	30 (24 %)	135 (28 %)	43 (26 %)	0.64
>2 cm	95 (76 %)	351 (72 %)	126 (74 %)	
Nodal status				
Negative	47 (38 %)	241 (49 %)	83 (49 %)	0.05
Positive	78 (62 %)	245 (51 %)	86 (51 %)	
Histology				
Ductal	116 (93 %)	371 (76 %)	121 (71 %)	<0.001
Lobular	9 (7 %)	95 (24 %)	48 (29 %)	
PAM50 subtype				
Luminal-A	44 (35 %)	200 (41 %)	70 (41 %)	0.16
Luminal-B	41 (33 %)	140 (29 %)	64 (38 %)	
HER2	15 (12 %)	57 (12 %)	12 (7 %)	
Basal	25 (20 %)	89 (18 %)	23 (14 %)	

### Somatic mutations according to age

**Fig. 1:**
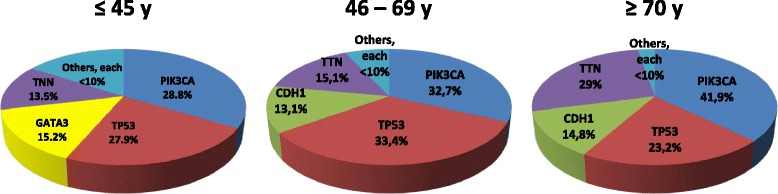
Prevalence of somatic mutations according to age

We found a significant association between age at diagnosis and the prevalence of somatic mutations. Median number of somatic mutations in the young group was 31, compared to 35 and 44 in the intermediate and older patient groups, respectively (*P* value = 0.0009). Figure 1 shows the four most prevalent somatic mutations in the different age groups. PIK3CA and TP53 were the most common somatic mutations, constituting around 50–60 % of all mutations across the different age groups. The striking difference between the three age groups was for GATA3, which was the third most common somatic mutation in young patients, constituting 15.2 %, while TTN mutation was the third most frequent mutation in the intermediate (15.1 %) and older patient groups (29 %).

To evaluate the independent effect of age on the prevalence of somatic mutations, we performed a logistic regression analysis adjusted for tumor size, nodal status, histology and breast cancer molecular subtype. We found 11 mutations to be independently associated with age at diagnosis (Table 2). All were associated with older age at diagnosis, except GATA3, which was independently associated with breast cancer arising in young women (15.2 % versus 8.2 % versus 9 %; *P* = 0.003, false discovery rate (FDR) = 0.033).

**Table 2:** The independent association between age at diagnosis and somatic mutations

	Young age	Intermediate age	Older age	Logistic model	FDR
	(≤45 years, n = 118)	(46–69 years, n = 449)	(≥70 years, n = 155)	(*P* value)^a^	
Mutations independently associated with young age at diagnosis					
GATA3	18 (15.2 %)	37 (8.2 %)	14 (9 %)	0.003	0.033
Mutations independently associated with older age at diagnosis					
TTN	16 (13.5 %)	68 (15.1 %)	45 (29 %)	0.0003	0.01
KMT2D	1 (0.8 %)	9 (2 %)	9 (5.8 %)	0.0003	0.01
CSPP1	0	3 (0.6 %)	8 (5.1 %)	0.0002	0.01
FOXA1	1 (0.8 %)	6 (1.3 %)	9 (5.8 %)	0.0009	0.013
XIST	0	6 (1.3 %)	9 (5.8 %)	0.0008	0.013
KMT2C	4 (3.3 %)	26 (5.7 %)	18 (11.6 %)	0.002	0.027
SYNE2	3 (2.5 %)	16 (3.5 %)	13 (8.3 %)	0.005	0.033
SPEN	2 (1.6 %)	13 (2.8 %)	12 (7.7 %)	0.005	0.033
USP34	1 (0.8 %)	12 (2.6 %)	9 (5.8 %)	0.004	0.033
ANK2	0	11 (2.4 %)	9 (5.8 %)	0.007	0.043

^a^Analysis adjusted for age, tumor size, nodal status, histology and breast cancer subtype. Only mutations with a minimum prevalence of 5 % in at least one age group is included. FDR, false discovery rate

### Somatic CNV events according to age

**Fig. 2:**
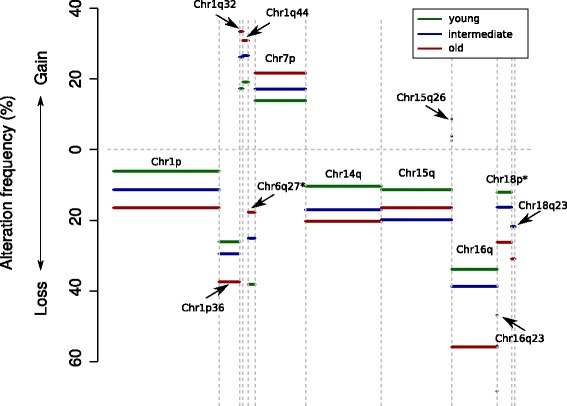
Copy number variation (CNV) events that are significantly different according to age (*P* <0.05 in the adjusted logistic regression model). Green represents younger patients (≤45 years), blue represents intermediate (46–69 years) and red represents elderly patients (≥70 years). The Y access shows the percentage and indicates the direction of CNV gain (above 0) or loss (below 0). *Aberrations that show a false discovery rate (FDR) <0.05

We evaluated the prevalence of CNV events according to age. We found a tendency of higher focal and broad CNV in older patients (mean = 15), compared to 13.9 and 13.5 in the intermediate and younger age groups, respectively (*P* = 0.2). The differences were more apparent when restricting the analysis to patients with ductal carcinoma (mean CNV in older patients = 16.4 versus 14.9 in intermediate versus 13.8 in young patients; *P* = 0.05). In a logistic regression model, we found 13 CNV events to be independently associated with age (Fig. 2, Additional file 1). However, upon adjusting for multiple testing, only two CNV events maintained a *P* value <0.05: chr18p loss and chr6q27 deletion; the former was associated with tumors diagnosed in older patients, while the latter was more common in younger patients.

### Gene expression differences according to age

We evaluated the association between age at diagnosis and the expression of 10,296 gene expression signatures. In a logistic regression model adjusted for tumor size, nodal status, histology and breast cancer molecular subtype, we found around 1,200 gene signatures to be independently associated with age at diagnosis (FDR <0.05), mainly in younger patients (Additional file 2). The main themes that emerged from this analysis are summarized in Table 3 and indicated higher expression of signatures related to proliferation, stem cell and endocrine resistance in tumors arising at young age.

**Table 3:** Selected gene expression signatures that are highly expressed in young breast cancer patients

Signature	Logistic regression^b^	FDR
Endocrine resistance		
Creighton_Endocrine_Therapy_Resistance^a^	1.03E-18	1.07E-14
Massarweh_Tamaxifen_Resistance	1.79E-14	1.84-10
Masri_resistance_to Tamoxifen_And_Aromatase_Inhibitor	7.58E-09	7.66E-05
Proliferation		
Regulation of cell cycle	1.43E-11	1.47E-07
Regulation of Mitotic_Cell_Cycle	8.43E-10	8.59E-06
Stem cell		
Lim_Mammary_Stem_Cell	3.62E-08	0.0003
PID_Notch_Pathway	1.52E-11	1.56E-07
Benporath_SOX2_Targets	1.58E-10	1.62E-06
Galie_Tumor_Stemness_Genes	1.62E-07	0.001
Notch_Signaling_Pathway	1.00E_06	0.009
Nguyen_Notch_Targets_Up	1.12E-06	0.01

^a^More than one signature; ^b^model adjusted for tumor size, nodal status, tumor histology and breast cancer subtype. FDR, false discovery rate

## Discussion

This is the first analysis to explore the prevalence of somatic mutations and CNV according to age. Our findings indicate that age is associated with unique biological features at the DNA level, independent of tumor stage, histology and breast cancer molecular subtype. In addition, age at diagnosis appears to impact the tumor transcriptome confirming previous observations, but also highlighting novel findings. While previous studies provide ample information on the differences at the pathological level according to age [2, 15], this study provides further insights on differences at the genomic level as well. This is also in line with previous studies that showed changes in the normal breast at both the genomic and epigenetic level between young and older women, including changes in genes that are known to be relevant in breast carcinogenesis [16, 17]. Such evidence may suggest the need to explore treatment strategies in patients diagnosed at extremes of age based on their unique molecular makeup.

Different themes emerged from our analysis. First, older patients have more mutations and CNV events. This is likely a reflection of more genomic errors accumulated in the DNA as women age. We found that several somatic mutations were independently associated with older age at diagnosis. Of particular relevance, the high prevalence of KMT2D mutations. Since this gene was recently shown to be involved in tumor proliferation and cell migration [18], we speculate that KMT2D mutations may alter breast cancer behavior. Another finding is the high prevalence of FOXA1 mutations. The latter is required for ER-alpha as a cofactor for chromatin binding and constitutes a major proliferative and survival pathway for luminal-A tumors [19], which are common in older patients [20]. Nevertheless, it is yet to be determined whether these mutations and/or others represent key driver mutations of tumors arising in older patients and the optimal way of targeting them.

On the other hand, GATA3 mutation was the main somatic event that characterized tumors arising at a younger age, which could have relevant clinical implications. GATA3 is an essential component of the ER complex and its mutations are likely to affect ER-regulated transcriptional activity [21, 22]. GATA3 directly upregulates ER-alpha and other proto-oncogenes suggesting that it may promote tumorigenesis in luminal cancer [23]. Preclinical data indicate that mutations in GATA3 also affect ER binding to DNA [22, 24], modulate response of breast cancer cells to estrogen signaling [25], could promote tumor growth [21, 26] and could be associated with endocrine resistance [25]. This is of extreme relevance, since the poor prognosis associated with younger age at diagnosis has been mainly observed in patients with ER-positive breast cancers [3, 5]. We could speculate that the higher prevalence of GATA3 mutations in these patients may render these patients more resistant to endocrine therapy. Our transcriptomic analyses also highlights the high expression of endocrine resistance signatures in younger patients, thus suggesting that endocrine resistance is an important hallmark of tumors arising in young women, worthy of further exploration. Of note, previous preclinical studies have shown that GATA expression (not mutation) results in reversal of the epithelial-mesenchymal transition (EMT) and induction of differentiation in basal-like tumors [27, 28]. Therefore, it is the loss of GATA3 expression that was suggested to contribute to the aggressiveness of basal-like tumors. Using our dataset, we found that GATA3 expression is higher in patients with GATA3 mutation (data not shown). These mutations were mostly exclusive in patients with ER-positive breast cancer. Thus, based on our findings, we cannot assume that the higher rate of GATA3 mutations observed in younger patients is linked to the known increased incidence of basal-like tumors in these patients.

CNVs are genomic events that are regarded as highly biologically relevant in breast cancer [29] and we found two events, more chr18p losses and chr6q27 deletions, to be independently associated with age at diagnosis. chr18p loss was more common in older patients and previous data indicated that it is associated with higher risk of recurrence [30]. Of note, chr18 also harbors SMAD4, which is a known tumor suppressor gene and has been shown to be associated with poor prognosis in several tumor types when lost [31–33]. On the other hand, very little is known on its significance in breast cancer. A previous study showed that chromosome 6 is frequently rearranged in breast cancer, particularly at three regions, including 6q27 [34]. In addition, chr6q27 deletion appears to be more prevalent in tumors with aggressive features [34]. This may suggest that this region could harbor relevant tumor suppressor genes that may contribute to the aggressive nature of tumors arising in younger patients.

Another key point emerging from our study is the existence of relevant gene expression differences according to age. Previously, we showed that tumors arising in young women are enriched with stem cell-related genes [5]. In addition, Pirone et al. have shown that pathways implicated in maintaining stem cell dynamics, Wnt/β-catenin and ephrin receptor signaling [35, 36] were differentially expressed in the normal breast between young and older women [16]. The current analysis corroborates this association and suggests that targeting the stem cell component is a strategy that deserves exploration in young breast cancer patients. Currently, there are several drugs in development, such as Notch inhibitors that are known to target the stem cell compartment [37]. Of note, in the current analysis, we found high expression of signatures related to Notch signaling pathways (Table 3) in young breast cancer patients, which may suggest the potential relevance of exploring such strategies in younger patients.

We recently initiated a preoperative window trial evaluating the role of targeting RANKL, a known stem cell regulator [38] and in which we have previously shown to be  highly expressed in tumors arising at a young age [5, 39]. In this trial (D-BEYOND; NCT01864798), all patients are premenopausal and receive the anti-RANKL monoclonal antibody denosumab before surgery. The aim is to evaluate the impact of RANKL inhibition on several biological processes, including proliferation, stem cell markers, immune-related markers, and many others. The trial has recruited >50 % of its target accrual and represents a proof of concept that could open the door for designing future trials in women diagnosed at extremes of age, based on a better understanding of the biology of their tumors.

## Conclusion

In conclusion, the present work shows that tumors arising at different ages are biologically distinct, not only at the protein level, as previously shown, but also at the RNA and DNA levels. This includes aberrations in relevant cancer-related genes. While current treatment decision-making is mainly based on tumor stage and breast cancer subtype, our analysis suggests that age adds a layer of biological complexity, worthy of investigating tailored therapeutic strategies in specific age groups. This could further result in refining therapeutic strategies as we embark on an era of personalized medicine.

## Additional files

Additional file 1:
**The independent association between age at diagnosis and chromosomal copy number variation (CNV) events.** (DOCX 15 kb) Additional file 2:
**The independent association between age at diagnosis and gene expression signatures (>10,000) in a logistic regression model adjusted for tumor size, nodal status, tumor histology and breast cancer subtype.** (PDF 1780 kb)

## Abbreviations

CGH: Comparative genomic hybridization
CNV: Copy number variation
EMT: Epithelial-mesenchymal transition
ER: Estrogen receptor
FDR: False discovery rate
GISTIC 2.0: Genomic Identification of Significant Targets in Cancer, version 2.0
HER2: Human epidermal growth factor receptor 2
MSigDB: Molecular Signatures Database
TCGA: The Cancer Genome Atlas
