# Supplementary material for: Genomic aberrations in young and elderly breast cancer patients
Source: BMC Med. 2015 Oct 15;13:266. doi: 10.1186/s12916-015-0504-3 (PMC4606505; doi:10.1186/s12916-015-0504-3)
Supplement: Additional file 1: — The independent association between age at diagnosis and chromosomal copy number variation (CNV) events. (DOCX 15 kb) [file 12916_2015_504_MOESM1_ESM.docx]

**Additional file 1:** The independent association between age at diagnosis and chromosomal copy number variation events.

|  | Young (≤45y, n=115) | Intermediate (46 – 69y, n=446) | Old (≥70y, n=152) | Logistic model* (p-value) | FDR |
| --- | --- | --- | --- | --- | --- |
| Chr18p_loss | 14 (12.1%) | 73 (16.3%) | 40 (26.3) | <0.0001 | 0.003 |
| Chr6q27_deletion | 44 (38.2%) | 112 (25.1%) | 27 (17.7%) | 0.0001 | 0.006 |
| Chr1q32_1_amplification | 20 (17.3%) | 117 (26.2%) | 51 (33.5%) | 0.003 | 0.09 |
| Chr14q_loss | 12 (10.4%) | 76 (17.0%) | 31 (20.3%) | 0.005 | 0.1 |
| Chr1p36_21_deletion | 30 (26.1%) | 132 (29.5%) | 57 (37.5%) | 0.007 | 0.1 |
| Chr1p_loss | 7 (6.1%) | 51 (11.4%) | 25 (16.4%) | 0.008 | 0.1 |
| Cr18q23_deletion | 25 (21.7%) | 98 (21.9%) | 47 (30.9%) | 0.008 | 0.1 |
| Chr16q23_3_deletion | 54 (46.9%) | 248 (55.6%) | 104 (68.4%) | 0.017 | 0.17 |
| Chr16q_loss | 39 (33.9%) | 173 (38.7%) | 85 (55.9%) | 0.025 | 0.22 |
| Chr15q26_3_amplification | 10 (8.6%) | 17 (3.8%) | 4 (2.6%) | 0.03 | 0.22 |
| Chr7p_gain | 16 (13.9%) | 77 (17.2%) | 33 (21.7%) | 0.03 | 0.22 |
| Chr1q44_amplification | 22 (19.1%) | 119 (26.6%) | 47 (30.9%) | 0.03 | 0.22 |
| Chr15q_loss | 13 (11.3%) | 89 (19.9%) | 25 (16.4%) | 0.03 | 0.22 |

* Analysis adjusted for age, tumor size, nodal status, histology and breast cancer subtype. FDR: false discovery rate.
