# Supplementary material for: Genomic aberrations in young and elderly breast cancer patients
Source: BMC Med. 2015 Oct 15;13:266. doi: 10.1186/s12916-015-0504-3 (PMC4606505; doi:10.1186/s12916-015-0504-3)
Supplement: Additional file 2: — The independent association between age at diagnosis and gene expression signatures (>10,000) in a logistic regression model adjusted for tumor size, nodal status, tumor histology and breast cancer subtype. (PDF 1780 kb) [file 12916_2015_504_MOESM2_ESM.pdf]

Signature	corr	pval	pval.adj	fdr.adj
CREIGHTON_ENDOCRINE_THERAPY_RESISTAN(	-0,136	2E-04	1E-18	1E-14
POSITIVE_REGULATION_OF_DNA_METABOLIC_	-0,29	7E-16	2,3E-16	2E-12
CREIGHTON_ENDOCRINE_THERAPY_RESISTAN(	-0,15	4E-05	1,2E-15	1E-11
EGFR_UP.V1_DN	-0,187	3E-07	2,2E-15	2E-11
MYLLYKANGAS_AMPLIFICATION_HOT_SPOT_3	-0,27	7E-14	4,5E-15	5E-11
MASSARWEH_TAMOXIFEN_RESISTANCE_DN	-0,085	0,02	1,8E-14	2E-10
chr4q33	-0,265	2E-13	1,8E-14	2E-10
NEBEN_AML_WITH_FLT3_OR_NRAS_DN	-0,27	7E-14	4,8E-14	5E-10
MATZUK_CUMULUS_EXPANSION	-0,188	2E-07	2,4E-13	2E-09
JISON_SICKLE_CELL_DISEASE_DN	-0,221	1E-09	2,9E-13	3E-09
V$TAXCREB_02	-0,277	1E-14	3,8E-13	4E-09
V$E2F_Q6_01	-0,258	8E-13	4,8E-13	5E-09
KTGGYRSGAA_UNKNOWN	-0,269	7E-14	5,3E-13	5E-09
MODULE_202	-0,199	4E-08	1E-12	1E-08
GSE27786_LIN_NEG_VS_NKCELL_UP	-0,266	1E-13	1,3E-12	1E-08
WILLIAMS_ESR1_TARGETS_UP	-0,184	5E-07	2E-12	2E-08
REGULATION_OF_CELL_DIFFERENTIATION	-0,183	5E-07	2E-12	2E-08
REGULATION_OF_DNA_METABOLIC_PROCESS	-0,264	3E-13	2,3E-12	2E-08
PANGAS_TUMOR_SUPPRESSION_BY_SMAD1_/	-0,197	6E-08	2,4E-12	2E-08
V$E2F_03	-0,245	1E-11	5,1E-12	5E-08
NIELSEN_GIST_AND_SYNOVIAL_SARCOMA_UI	-0,208	1E-08	5,2E-12	5E-08
GSE11924_TH2_VS_TH17_CD4_TCELL_DN	-0,213	4E-09	5,3E-12	5E-08
KEGG_TIGHT_JUNCTION	-0,228	3E-10	6,3E-12	6E-08
FRASOR_RESPONSE_TO_ESTRADIOL_UP	-0,062	0,091	6,9E-12	7E-08
MASSARWEH_RESPONSE_TO_ESTRADIOL	-0,062	0,091	8,4E-12	9E-08
STAEGE_EWING_FAMILY_TUMOR	-0,176	1E-06	9E-12	9E-08
SIG_PIP3_SIGNALING_IN_CARDIAC_MYOCTES	-0,212	5E-09	1E-11	1E-07
V$E2F_Q4_01	-0,245	1E-11	1,2E-11	1E-07
TRANSCRIPTION_COFACTOR_ACTIVITY	-0,206	1E-08	1,3E-11	1E-07
REGULATION_OF_CELL_CYCLE	-0,247	9E-12	1,4E-11	1E-07
PID_NOTCH_PATHWAY	-0,216	3E-09	1,5E-11	2E-07
KEGG_ERBB_SIGNALING_PATHWAY	-0,235	8E-11	1,6E-11	2E-07
KEGG_P53_SIGNALING_PATHWAY	-0,255	2E-12	1,8E-11	2E-07
SMID_BREAST_CANCER_ERBB2_DN	-0,198	5E-08	2,1E-11	2E-07
BOYLAN_MULTIPLE_MYELOMA_C_CLUSTER_U	-0,245	1E-11	2,4E-11	2E-07
GSE3982_BASOPHIL_VS_CENT_MEMORY_CD4	-0,182	6E-07	2,4E-11	2E-07
V$TAXCREB_01	-0,212	5E-09	2,5E-11	3E-07
VERHAAK_GLIOBLASTOMA_PRONEURAL	-0,239	4E-11	2,5E-11	3E-07
DOUBLE_STRANDED_DNA_BINDING	-0,25	4E-12	2,9E-11	3E-07
LUI_THYROID_CANCER_CLUSTER_1	-0,146	6E-05	3,7E-11	4E-07
NUCLEUS	-0,244	1E-11	3,9E-11	4E-07

GSE11924_TH1_VS_TH2_CD4_TCELL_UP	-0,211	6E-09	4,4E-11	4E-07
V$SPZ1_01	-0,197	6E-08	4,7E-11	5E-07
V$AREB6_03	-0,167	4E-06	4,7E-11	5E-07
V$E2F_Q3_01	-0,239	4E-11	5,5E-11	6E-07
V$PAX3_B	-0,207	1E-08	5,6E-11	6E-07
chr4q34	-0,183	5E-07	8,1E-11	8E-07
DEURIG_T_CELL_PROLYMPHOCYTIC_LEUKEMI/	-0,228	3E-10	8,5E-11	9E-07
STRUCTURE_SPECIFIC_DNA_BINDING	-0,248	7E-12	8,5E-11	9E-07
PID_ERBB_NETWORK_PATHWAY	-0,223	8E-10	8,9E-11	9E-07
GSE3982_NEUTROPHIL_VS_TH2_DN	-0,248	6E-12	1,1E-10	1E-06
TRANSCRIPTION_COACTIVATOR_ACTIVITY	-0,188	3E-07	1,3E-10	1E-06
MARTINEZ_RB1_AND_TP53_TARGETS_DN	-0,226	4E-10	1,3E-10	1E-06
TRANSCRIPTION_ACTIVATOR_ACTIVITY	-0,191	2E-07	1,4E-10	1E-06
REACTOME_CELL_JUNCTION_ORGANIZATION	-0,23	2E-10	1,4E-10	1E-06
GSE33513_TCF7_KO_VS_HET_EARLY_THYMI	-0,189	2E-07	1,5E-10	2E-06
MIKKELSEN_MEF_ICP_WITH_H3K4ME3_AND_I	-0,184	4E-07	1,5E-10	2E-06
LOPEZ_TRANSLATION_VIA_FN1_SIGNALING	-0,207	1E-08	1,5E-10	2E-06
V$E2F1_Q4_01	-0,235	8E-11	1,6E-10	2E-06
BENPORATH_SOX2_TARGETS	-0,247	8E-12	1,6E-10	2E-06
REACTOME_PI3K_AKT_ACTIVATION	-0,139	1E-04	1,6E-10	2E-06
HONRADO_BREAST_CANCER_BRCA1_VS_BRC	-0,245	1E-11	1,7E-10	2E-06
GSE27786_LIN_NEG_VS_ERYTHROBLAST_DN	-0,178	1E-06	1,9E-10	2E-06
TRANSCRIPTION_FACTOR_BINDING	-0,198	5E-08	1,9E-10	2E-06
PEDERSEN_METASTASIS_BY_ERBB2_ISOFORM	-0,217	2E-09	1,9E-10	2E-06
MCCABE_HOXC6_TARGETS_CANCER_UP	-0,186	3E-07	2,3E-10	2E-06
GSE37416_CTRL_VS_0H_F_TULARENSIS_LV!	-0,205	2E-08	2,4E-10	3E-06
GLUTAMATE_SIGNALING_PATHWAY	-0,174	2E-06	2,6E-10	3E-06
GSE27786_ERYTHROBLAST_VS_MONO_MAC_	-0,214	3E-09	2,6E-10	3E-06
V$HMGIY_Q6	-0,215	3E-09	2,8E-10	3E-06
REACTOME_CELL_CELL_JUNCTION_ORGANIZA	-0,24	3E-11	2,8E-10	3E-06
CUI_TCF21_TARGETS_2_UP	-0,217	2E-09	2,9E-10	3E-06
MUSCLE_CELL_DIFFERENTIATION	-0,163	7E-06	3E-10	3E-06
NUCLEOBASENUCLEOSIDENUCLEOTIDE_AND_I	-0,223	8E-10	3E-10	3E-06
V$EGR1_01	-0,198	5E-08	3,4E-10	3E-06
NIELSEN_LEIOMYOSARCOMA_CNN1_UP	-0,204	2E-08	3,5E-10	4E-06
CONCANNON_APOPTOSIS_BY_EPOXOMICIN_D	-0,233	1E-10	3,8E-10	4E-06
V$E2F_Q2	-0,19	2E-07	3,9E-10	4E-06
GNF2_CDH3	-0,235	9E-11	3,9E-10	4E-06
REGULATION_OF_DNA_REPLICATION	-0,233	1E-10	4E-10	4E-06
V$AP2_Q6_01	-0,188	2E-07	4,3E-10	4E-06
DUTERTRE_ESTRADIOL_RESPONSE_6HR_UP	-0,167	5E-06	4,4E-10	4E-06
INTRACELLULAR_NON_MEMBRANE_BOUND_O	-0,241	3E-11	4,5E-10	5E-06

NON_MEMBRANE_BOUND_ORGANELLE	-0,241	3E-11	4,5E-10	5E-06
ASTON_MAJOR_DEPRESSIVE_DISORDER_UP	-0,182	6E-07	4,8E-10	5E-06
PHOSPHATE_TRANSMEMBRANE_TRANSPORTE	-0,216	2E-09	4,9E-10	5E-06
ARGGGTTAA_UNKNOWN	-0,163	8E-06	5,5E-10	6E-06
YANG_BREAST_CANCER_ESR1_DN	-0,204	2E-08	5,6E-10	6E-06
V$CREB_Q3	-0,163	8E-06	5,9E-10	6E-06
REACTOME_PI3K_EVENTS_IN_ERBB4_SIGNALI	-0,17	3E-06	5,9E-10	6E-06
GSE12845_IGD_NEG_BLOOD_VS_DARKZONE_	-0,243	2E-11	6,1E-10	6E-06
GSE3982_BASOPHIL_VS_TH2_DN	-0,234	1E-10	6,6E-10	7E-06
V$AP2ALPHA_01	-0,196	7E-08	6,6E-10	7E-06
RNA_METABOLIC_PROCESS	-0,214	3E-09	6,8E-10	7E-06
YAO_TEMPORAL_RESPONSE_TO_PROGESTER(	-0,141	1E-04	6,9E-10	7E-06
RRCCGTTA_UNKNOWN	-0,234	1E-10	7E-10	7E-06
LANDEMAINE_LUNG_METASTASIS	-0,188	3E-07	7,6E-10	8E-06
KCCGNSWTTT_UNKNOWN	-0,213	4E-09	7,6E-10	8E-06
CELL_CYCLE_ARREST_GO_0007050	-0,224	6E-10	7,6E-10	8E-06
WANG_NEOPLASTIC_TRANSFORMATION_BY_(	-0,174	2E-06	7,7E-10	8E-06
DNA_METABOLIC_PROCESS	-0,209	8E-09	7,9E-10	8E-06
LE_SKI_TARGETS_DN	-0,181	7E-07	8E-10	8E-06
REGULATION_OF_MITOTIC_CELL_CYCLE	-0,213	4E-09	8,4E-10	9E-06
ZHENG_GLIOBLASTOMA_PLASTICITY_UP	-0,24	3E-11	8,5E-10	9E-06
GSE20366_CD103_KLRG1_DP_VS_DN_TREG_	-0,161	1E-05	8,7E-10	9E-06
GSE14000_TRANSLATED_RNA_VS_MRNA_16	-0,165	6E-06	8,9E-10	9E-06
CADWELL_ATG16L1_TARGETS_DN	-0,208	1E-08	9E-10	9E-06
REACTOME_SHC1_EVENTS_IN_ERBB4_SIGNAL	-0,224	7E-10	9,3E-10	1E-05
V$E2F1_Q4	-0,22	1E-09	9,5E-10	1E-05
DARWICHE_PAPILLOMA_RISK_HIGH_VS_LOW_	-0,151	3E-05	1E-09	1E-05
V$ER_Q6_01	-0,17	3E-06	1,1E-09	1E-05
GSE8515_IL1_VS_IL6_4H_STIM_)MAC_DN	-0,113	0,002	1,1E-09	1E-05
NEGATIVE_REGULATION_OF_NUCLEOBASENU(	-0,186	3E-07	1,2E-09	1E-05
MODULE_533	-0,2	3E-08	1,2E-09	1E-05
GSE27786_CD8_TCELL_VS_NKCELL_UP	-0,197	6E-08	1,3E-09	1E-05
FIGUEROA_AML_METHYLATION_CLUSTER_2_I	-0,212	5E-09	1,3E-09	1E-05
PRC2_EZH2_UP.V1_UP	-0,227	4E-10	1,4E-09	1E-05
CELL_CYCLE_GO_0007049	-0,213	5E-09	1,5E-09	2E-05
GSE10325_MYELOID_VS_LUPUS_MYELOID_UI	-0,149	4E-05	1,5E-09	2E-05
GSE3982_NEUTROPHIL_VS_CENT_MEMORY_(	-0,212	5E-09	1,6E-09	2E-05
GSE3982_EFF_MEMORY_VS_CENT_MEMORY_	-0,181	7E-07	1,7E-09	2E-05
V$ER_Q6	-0,167	4E-06	1,7E-09	2E-05
V$E2F_01	-0,239	4E-11	1,8E-09	2E-05
V$AHR_01	-0,201	3E-08	1,9E-09	2E-05
GSE1448_CTRL_VS_ANTI_VALPHA2_DP_THY	-0,215	3E-09	2E-09	2E-05

GSE3982_CENT_MEMORY_CD4_TCELL_VS_N	-0,171	3E-06	2E-09	2E-05
NEGATIVE_REGULATION_OF_TRANSCRIPTION	-0,168	4E-06	2E-09	2E-05
GNF2_SERPINB5	-0,226	4E-10	2E-09	2E-05
REACTOME_ACTIVATED_NOTCH1_TRANSMITS	-0,19	2E-07	2,1E-09	2E-05
MCCABE_BOUND_BY_HOXC6	-0,151	3E-05	2,3E-09	2E-05
CAHOY_OLIGODENDROCUTIC	-0,195	8E-08	2,3E-09	2E-05
KIM_MYCN_AMPLIFICATION_TARGETS_UP	-0,196	7E-08	2,4E-09	2E-05
GNF2_RTN1	-0,188	2E-07	2,5E-09	2E-05
REGULATION_OF_TRANSCRIPTION_FROM_RNA	-0,178	1E-06	2,5E-09	3E-05
CTGRYYYNATT_UNKNOWN	-0,194	9E-08	2,6E-09	3E-05
GNF2_AF1Q	-0,19	2E-07	2,6E-09	3E-05
DORN_ADENOVIRUS_INFECTION_24HR_UP	-0,188	2E-07	2,6E-09	3E-05
TURASHVILI_BREAST_DUCTAL_CARCINOMA_V	-0,168	4E-06	2,7E-09	3E-05
REACTOME_SIGNALING_BY_ERBB2	-0,163	8E-06	2,7E-09	3E-05
V$CREB_02	-0,191	2E-07	2,7E-09	3E-05
POSITIVE_REGULATION_OF_CELL_DIFFERENTI	-0,148	5E-05	2,8E-09	3E-05
V$LBP1_Q6	-0,154	3E-05	2,8E-09	3E-05
MODULE_438	-0,229	3E-10	2,8E-09	3E-05
PID_DELTANP63PATHWAY	-0,218	2E-09	2,9E-09	3E-05
V$MYB_Q6	-0,161	1E-05	3E-09	3E-05
MARKEY_RB1_CHRONIC_LOF_UP	-0,23	2E-10	3E-09	3E-05
SANSOM_APC_TARGETS	-0,186	3E-07	3E-09	3E-05
BIOCARTA_ERK_PATHWAY	-0,212	5E-09	3,1E-09	3E-05
TESAR_ALK_TARGETS_HUMAN_ES_4D_DN	-0,182	6E-07	3,1E-09	3E-05
MARTINEZ_TP53_TARGETS_DN	-0,205	2E-08	3,5E-09	4E-05
V$AP2GAMMA_01	-0,188	2E-07	3,6E-09	4E-05
BOYLAN_MULTIPLE_MYELOMA_C_D_UP	-0,224	7E-10	3,6E-09	4E-05
BAKER_HEMATOPOESIS_STAT5_TARGETS	-0,191	2E-07	3,6E-09	4E-05
GTCNYYATGR_UNKNOWN	-0,153	3E-05	3,6E-09	4E-05
GSE13485_DAY3_VS_DAY7_YF17D_VACCINI	-0,088	0,016	3,7E-09	4E-05
YAMAZAKI_TCEB3_TARGETS_DN	-0,237	6E-11	3,8E-09	4E-05
PID_TRKRPATHWAY	-0,157	2E-05	3,8E-09	4E-05
V$ER_Q6_02	-0,185	4E-07	3,9E-09	4E-05
BYSTRYKH_HEMATOPOIESIS_STEM_CELL_FLI1	-0,161	1E-05	4,3E-09	4E-05
NUNODA_RESPONSE_TO_DASATINIB_IMATINIB	-0,211	6E-09	4,3E-09	4E-05
POMEROY_MEDULLOBLASTOMA_PROGNOSIS_I	-0,166	5E-06	4,4E-09	4E-05
HATADA_METHYLATED_IN_LUNG_CANCER_UI	-0,147	6E-05	4,6E-09	5E-05
SIG_REGULATION_OF_THE_ACTIN_CYTOSKELI	-0,235	9E-11	4,7E-09	5E-05
GSE27786_CD4_TCELL_VS_ERYTHTROBLAST	-0,132	3E-04	5E-09	5E-05
GACAGGG,MIR-339	-0,107	0,003	5,2E-09	5E-05
NEGATIVE_REGULATION_OF_CELL_DIFFERENT	-0,146	7E-05	5,3E-09	5E-05
GGGCGGR_V$SP1_Q6	-0,169	3E-06	5,4E-09	5E-05

MORF_WNT1	-0,152	3E-05	5,5E-09	6E-05
V$MYCMAX_01	-0,209	8E-09	5,5E-09	6E-05
NIELSEN_SCHWANNOMA_DN	-0,163	8E-06	5,5E-09	6E-05
V$MAZ_Q6	-0,154	3E-05	5,7E-09	6E-05
RYTAAWNNNTGAY_UNKNOWN	-0,152	3E-05	5,8E-09	6E-05
IVANOVA_HEMATOPOIESIS_STEM_CELL_LONG	-0,135	2E-04	6E-09	6E-05
CTGCAGY_UNKNOWN	-0,152	3E-05	6E-09	6E-05
GSE15324_NAIVE_VS_ACTIVATED_CD8_TCE	-0,178	1E-06	6,1E-09	6E-05
ATGTCAC,MIR-489	-0,182	6E-07	6,1E-09	6E-05
TRANSCRIPTION_DNA_DEPENDENT	-0,184	5E-07	6,2E-09	6E-05
REACTOME_SIGNALING_BY_NODAL	-0,149	4E-05	6,2E-09	6E-05
V$VDR_Q3	-0,16	1E-05	6,4E-09	7E-05
DORN_ADENOVIRUS_INFECTION_12HR_UP	-0,183	5E-07	6,4E-09	7E-05
HOELZEL_NF1_TARGETS_DN	-0,157	2E-05	6,6E-09	7E-05
GSE17721_CPG_VS_GARDIQUIMOD_0.5H_BM	-0,194	1E-07	6,7E-09	7E-05
RNA_BIOSYNTHETIC_PROCESS	-0,184	4E-07	6,8E-09	7E-05
REGULATION_OF_CHROMOSOME_ORGANIZATI	-0,241	3E-11	7,1E-09	7E-05
V$MYCMAX_02	-0,211	6E-09	7,1E-09	7E-05
OLSSON_E2F3_TARGETS_DN	-0,231	2E-10	7,2E-09	7E-05
FIGUEROA_AML_METHYLATION_CLUSTER_4_U	-0,202	3E-08	7,2E-09	7E-05
POTASSIUM_ION_TRANSPORT	-0,16	1E-05	7,4E-09	8E-05
HEDENFALK_BREAST_CANCER_BRCA1_VS_BR	-0,23	2E-10	7,5E-09	8E-05
MASRI_RESISTANCE_TO_TAMOXIFEN_AND_AR	-0,045	0,216	7,6E-09	8E-05
GSE17721_CTRL_VS_LPS_1H_BMDM_UP	-0,148	5E-05	7,6E-09	8E-05
KONDO_PROSTATE_CANCER_HCP_WITH_H3K	-0,13	4E-04	7,9E-09	8E-05
RIZKI_TUMOR_INVASIVENESS_2D_DN	-0,207	1E-08	7,9E-09	8E-05
V$MIF1_01	-0,108	0,003	8,2E-09	8E-05
GSE27786_LSK_VS_BCELL_UP	-0,138	2E-04	8,2E-09	8E-05
JEON_SMAD6_TARGETS_DN	-0,225	5E-10	8,3E-09	8E-05
INAMURA_LUNG_CANCER_SCC_UP	-0,23	2E-10	8,4E-09	9E-05
TISSUE_MORPHOGENESIS	-0,132	3E-04	8,5E-09	9E-05
GSE29614_CTRL_VS_DAY3_TIV_FLU_VACCIN	-0,187	3E-07	8,6E-09	9E-05
V$GATA2_01	-0,132	3E-04	9E-09	9E-05
SCHAEFFER_PROSTATE_DEVELOPMENT_12HR	-0,208	1E-08	9E-09	9E-05
PAL_PRMT5_TARGETS_UP	-0,219	1E-09	9,1E-09	9E-05
V$DR4_Q2	-0,161	1E-05	9,1E-09	9E-05
BENPORATH_NANOG_TARGETS	-0,223	8E-10	9,2E-09	9E-05
SA_TRKA_RECEPTOR	-0,185	4E-07	9,5E-09	1E-04
FIGUEROA_AML_METHYLATION_CLUSTER_7_U	-0,192	1E-07	9,8E-09	1E-04
ZHAN_MULTIPLE_MYELOMA_SPIKED	-0,177	1E-06	1E-08	0,0001
GSE27786_LIN_NEG_VS_BCELL_UP	-0,223	8E-10	1E-08	0,0001
V$STAT6_01	-0,181	7E-07	1E-08	0,0001

HOLLEMAN_ASPARAGINASE_RESISTANCE_B_/	-0,157	2E-05	1E-08	0,0001
INTERPHASE	-0,206	1E-08	1E-08	0,0001
SCHAEFFER_SOX9_TARGETS_IN_PROSTATE_[	-0,22	1E-09	1,1E-08	0,0001
NEGATIVE_REGULATION_OF_CELL_CYCLE	-0,194	9E-08	1,1E-08	0,0001
CCTGTGA,MIR-513	-0,154	2E-05	1,1E-08	0,0001
V$ZF5_01	-0,193	1E-07	1,1E-08	0,0001
V$LEF1_Q6	-0,168	4E-06	1,1E-08	0,0001
KEGG_ACUTE_MYELOID_LEUKEMIA	-0,174	2E-06	1,1E-08	0,0001
RAF_UP.V1_DN	-0,049	0,184	1,2E-08	0,0001
ENZYME_LINKED_RECEPTOR_PROTEIN_SIGNAL	-0,125	6E-04	1,2E-08	0,0001
BHAT_ESR1_TARGETS_NOT_VIA_AKT1_UP	-0,091	0,013	1,2E-08	0,0001
MACROMOLECULAR_COMPLEX	-0,217	2E-09	1,2E-08	0,0001
REACTOME_SIGNALING_BY_ROBO_RECEPTOR	-0,176	1E-06	1,2E-08	0,0001
V$ZIC2_01	-0,175	2E-06	1,2E-08	0,0001
FARMER_BREAST_CANCER_BASAL_VS_LULMI	-0,092	0,012	1,3E-08	0,0001
KEGG_SMALL_CELL_LUNG_CANCER	-0,205	2E-08	1,3E-08	0,0001
TRANSMEMBRANE_RECEPTOR_PROTEIN_TYRO	-0,129	4E-04	1,3E-08	0,0001
V$E12_Q6	-0,15	4E-05	1,3E-08	0,0001
STOSSI_RESPONSE_TO_ESTRADIOL	-0,104	0,005	1,3E-08	0,0001
INTERPHASE_OF_MITOTIC_CELL_CYCLE	-0,199	4E-08	1,3E-08	0,0001
REGULATION_OF_NUCLEOBASENUCLEOSIDENU	-0,174	2E-06	1,3E-08	0,0001
TRANSCRIPTION_FROM_RNA_POLYMERASE_II_	-0,19	2E-07	1,4E-08	0,0001
POTASSIUM_CHANNEL_ACTIVITY	-0,181	7E-07	1,4E-08	0,0001
NEGATIVE_REGULATION_OF_RNA_METABOLIC	-0,143	9E-05	1,4E-08	0,0001
NEGATIVE_REGULATION_OF_TRANSCRIPTION_	-0,143	9E-05	1,4E-08	0,0001
ACEVEDO_FGFR1_TARGETS_IN_PROSTATE_C.	-0,148	5E-05	1,5E-08	0,0001
PID_HEDGEHOG_2PATHWAY	-0,147	5E-05	1,5E-08	0,0001
GSE3982_EOSINOPHIL_VS_MAC_UP	-0,149	5E-05	1,5E-08	0,0001
BIOPOLYMER_METABOLIC_PROCESS	-0,204	2E-08	1,5E-08	0,0001
RYTGCNWTGGNR_UNKNOWN	-0,122	9E-04	1,5E-08	0,0002
TRANSCRIPTION	-0,172	2E-06	1,5E-08	0,0002
TURASHVILI_BREAST_LOBULAR_CARCINOMA_	-0,154	2E-05	1,5E-08	0,0002
V$STAT5A_02	-0,134	2E-04	1,5E-08	0,0002
chr18q12	-0,218	2E-09	1,6E-08	0,0002
V$RORA1_01	-0,159	1E-05	1,6E-08	0,0002
BIOCARTA_NDKDYNAMIN_PATHWAY	-0,203	2E-08	1,8E-08	0,0002
GSE22886_NAIVE_VS_MEMORY_TCELL_UP	-0,138	2E-04	1,8E-08	0,0002
V$P53_02	-0,159	1E-05	1,8E-08	0,0002
V$HEB_Q6	-0,156	2E-05	1,8E-08	0,0002
CHEN_LVAD_SUPPORT_OF_FAILING_HEART_U	-0,146	7E-05	1,8E-08	0,0002
MYAATNNNNNNNGGC_UNKNOWN	-0,207	1E-08	1,9E-08	0,0002
V$PAX8_B	-0,132	3E-04	1,9E-08	0,0002

IVANOVA_HEMATOPOIESIS_EARLY_PROGENIT(	-0,212	5E-09	1,9E-08	0,0002
V$NGFIC_01	-0,179	9E-07	2E-08	0,0002
REGULATION_OF_RNA_METABOLIC_PROCESS	-0,162	9E-06	2E-08	0,0002
GSE18791_CTRL_VS_NEWCASTLE_VIRUS_DC	-0,143	9E-05	2,1E-08	0,0002
V$CREB_Q2_01	-0,187	3E-07	2,1E-08	0,0002
CELLULAR_PROTEIN_COMPLEX_DISASSEMBLY	-0,232	1E-10	2,2E-08	0,0002
CCTGCTG,MIR-214	-0,14	1E-04	2,3E-08	0,0002
REACTOME_APOPTOTIC_EXECUTION_PHASE	-0,234	1E-10	2,3E-08	0,0002
GCAAGGA,MIR-502	-0,176	1E-06	2,3E-08	0,0002
REACTOME_IONOTROPIC_ACTIVITY_OF_KAINA	-0,116	0,002	2,3E-08	0,0002
GSE14000_4H_VS_16H_LPS_DC_TRANSLAT	-0,152	3E-05	2,3E-08	0,0002
MARTINEZ_RB1_TARGETS_DN	-0,183	5E-07	2,4E-08	0,0002
CATTGTYY_V$SOX9_B1	-0,19	2E-07	2,4E-08	0,0002
DAZARD_RESPONSE_TO_UV_SCC_DN	-0,197	6E-08	2,5E-08	0,0003
REGULATION_OF_TRANSCRIPTIONDNA_DEPEN	-0,157	2E-05	2,6E-08	0,0003
V$E2F1DP1_01	-0,206	1E-08	2,7E-08	0,0003
V$E2F1DP2_01	-0,206	1E-08	2,7E-08	0,0003
V$E2F4DP2_01	-0,206	1E-08	2,7E-08	0,0003
GSE27786_NKTCELL_VS_ERYTHROBLAST_DN	-0,132	3E-04	2,7E-08	0,0003
ERB2_UP.V1_DN	-0,176	1E-06	2,7E-08	0,0003
YRTCANNRCGC_UNKNOWN	-0,212	5E-09	2,7E-08	0,0003
GSE27786_ERYTHROBLAST_VS_NEUTROPHIL_	-0,179	8E-07	2,7E-08	0,0003
V$E2F_Q3	-0,207	1E-08	2,7E-08	0,0003
V$YY1_Q6	-0,172	2E-06	2,8E-08	0,0003
chr6p	-0,213	5E-09	2,8E-08	0,0003
PDGF_UP.V1_UP	-0,202	3E-08	2,8E-08	0,0003
BROWNE_HCMV_INFECTION_24HR_UP	-0,218	2E-09	2,9E-08	0,0003
V$E47_02	-0,134	2E-04	2,9E-08	0,0003
AUNG_GASTRIC_CANCER	-0,213	4E-09	2,9E-08	0,0003
GSE360_L_DONOVANI_VS_T_GONDII_DC_DN	-0,218	2E-09	2,9E-08	0,0003
MODULE_567	-0,223	8E-10	2,9E-08	0,0003
MORF_EPHA7	-0,14	1E-04	3E-08	0,0003
KEGG_GLIOMA	-0,214	3E-09	3E-08	0,0003
SHEPARD_CRUSH_AND_BURN_MUTANT_DN	-0,214	4E-09	3E-08	0,0003
SGCGSSAAA_V$E2F1DP2_01	-0,201	3E-08	3E-08	0,0003
NGUYEN_NOTCH1_TARGETS_DN	-0,188	2E-07	3E-08	0,0003
GSE26495_NAIVE_VS_PD1LOW_CD8_TCELL_	-0,133	3E-04	3E-08	0,0003
V$FOXO1_01	-0,152	3E-05	3,2E-08	0,0003
OSADA_ASCL1_TARGETS_UP	-0,131	3E-04	3,2E-08	0,0003
XU_GH1_AUTOCRINE_TARGETS_UP	-0,139	1E-04	3,2E-08	0,0003
CAGGTA_V$AREB6_01	-0,151	3E-05	3,2E-08	0,0003
MCBRYAN_PUBERTAL_BREAST_4_5WK_UP	-0,188	2E-07	3,2E-08	0,0003

DEVELOPMENTAL_MATURATION	-0,119	0,001	3,3E-08	0,0003
chr1p34	-0,201	3E-08	3,3E-08	0,0003
CELL_MATURATION	-0,11	0,003	3,3E-08	0,0003
HOLLEMAN_VINCRISTINE_RESISTANCE_ALL_L	-0,218	2E-09	3,4E-08	0,0003
NEGATIVE_REGULATION_OF_METABOLIC_PRC	-0,16	1E-05	3,4E-08	0,0003
V$ATF3_Q6	-0,159	1E-05	3,5E-08	0,0003
REGULATION_OF_TRANSCRIPTION	-0,152	3E-05	3,5E-08	0,0004
GSE1460_INTRATHYMIC_T_PROGENITOR_VS_	-0,215	3E-09	3,5E-08	0,0004
RNGTGGGC_UNKNOWN	-0,162	9E-06	3,5E-08	0,0004
BROWNE_HCMV_INFECTION_2HR_DN	-0,226	4E-10	3,6E-08	0,0004
GSE29618_PRE_VS_DAY7_POST_TIV_FLU_V	-0,147	6E-05	3,6E-08	0,0004
LIM_MAMMARY_STEM_CELL_UP	-0,165	6E-06	3,6E-08	0,0004
HOLLEMAN_ASPARAGINASE_RESISTANCE_ALI	-0,173	2E-06	3,7E-08	0,0004
MODULE_201	-0,136	2E-04	3,8E-08	0,0004
TESAR_ALK_TARGETS_HUMAN_ES_5D_DN	-0,177	1E-06	3,8E-08	0,0004
GSE339_CD8POS_VS_CD4CD8DN_DC_IN_CUI	-0,212	5E-09	3,8E-08	0,0004
ZEMBUTSU_SENSITIVITY_TO_NIMUSTINE	-0,196	7E-08	3,8E-08	0,0004
BRUINS_UVC_RESPONSE_VIA_TP53_GROUP_(	-0,151	3E-05	3,9E-08	0,0004
YTAAYNGCT_UNKNOWN	-0,169	3E-06	3,9E-08	0,0004
NEGATIVE_REGULATION_OF_CELLULAR_MET/	-0,164	7E-06	3,9E-08	0,0004
V$CREBP1CJUN_01	-0,181	6E-07	3,9E-08	0,0004
KEGG_NEUROTROPHIN_SIGNALING_PATHWAY	-0,175	2E-06	4E-08	0,0004
YGCGYRCGC_UNKNOWN	-0,163	8E-06	4E-08	0,0004
GRADE_COLON_VS_RECTAL_CANCER_DN	-0,18	8E-07	4E-08	0,0004
V$SMAD4_Q6	-0,173	2E-06	4E-08	0,0004
GSE1460_INTRATHYMIC_T_PROGENITOR_VS_	-0,184	4E-07	4,1E-08	0,0004
REACTOME_SIGNALING_BY_NOTCH1	-0,176	1E-06	4,2E-08	0,0004
GSE18148_CBFB_KO_VS_WT_TREG_DN	-0,135	2E-04	4,2E-08	0,0004
VOLTAGE_GATED_POTASSIUM_CHANNEL_CO	-0,172	2E-06	4,2E-08	0,0004
MIKKELSEN_MCV6_ICP_WITH_H3K4ME3_AND	-0,158	1E-05	4,3E-08	0,0004
REACTOME_DOWNREGULATION_OF_ERBB2_EI	-0,186	3E-07	4,3E-08	0,0004
CCTNTMAGA_UNKNOWN	-0,143	9E-05	4,3E-08	0,0004
GSE17721_PAM3CSK4_VS_CPG_0.5H_BMDM	-0,14	1E-04	4,3E-08	0,0004
GSE14308_INDUCED_VS_NATURAL_TREG_UF	-0,2	4E-08	4,4E-08	0,0004
DELPUECH_FOXO3_TARGETS_DN	-0,19	2E-07	4,4E-08	0,0004
chr7q	-0,188	3E-07	4,4E-08	0,0004
YTAATTAA_V$LHX3_01	-0,16	1E-05	4,5E-08	0,0004
V$E2F1_Q3	-0,207	1E-08	4,5E-08	0,0005
REACTOME_CELL_CELL_COMMUNICATION	-0,207	1E-08	4,6E-08	0,0005
MARKEY_RB1_ACUTE_LOF_DN	-0,195	8E-08	4,6E-08	0,0005
BEGUM_TARGETS_OF_PAX3_FOXO1_FUSION_	-0,189	2E-07	4,6E-08	0,0005
V$CREB_Q4_01	-0,176	1E-06	4,6E-08	0,0005

AAGCAAT,MIR-137	-0,146	6E-05	4,7E-08	0,0005
V$SP1_Q6	-0,159	1E-05	4,8E-08	0,0005
V$SF1_Q6	-0,184	4E-07	4,8E-08	0,0005
BIOCARTA_G1_PATHWAY	-0,215	3E-09	4,8E-08	0,0005
DNA_REPLICATION	-0,197	6E-08	4,9E-08	0,0005
GAUSSMANN_MLL_AF4_FUSION_TARGETS_G_	-0,137	2E-04	4,9E-08	0,0005
PID_TAP63PATHWAY	-0,194	1E-07	5E-08	0,0005
V$E2F1_Q6	-0,207	1E-08	5,1E-08	0,0005
GSE29618_PRE_VS_DAY7_POST_TIV_FLU_V	-0,167	5E-06	5,1E-08	0,0005
DNA_BINDING	-0,178	1E-06	5,1E-08	0,0005
MEISSNER_BRAIN_HCP_WITH_H3K4ME2	-0,187	3E-07	5,2E-08	0,0005
GALLUZZI_PERMEABILIZE_MITOCHONDRIA	-0,193	1E-07	5,3E-08	0,0005
GSE29618_PRE_VS_DAY7_POST_LAIV_FLU_	-0,151	4E-05	5,4E-08	0,0005
CROONQUIST_NRAS_VS_STROMAL_STIMULAT	-0,202	3E-08	5,5E-08	0,0005
LI_WILMS_TUMOR_VS_FETAL_KIDNEY_2_UP	-0,18	8E-07	5,6E-08	0,0006
ZHAN_MULTIPLE_MYELOMA_MS_UP	-0,122	9E-04	5,6E-08	0,0006
AMBROSINI_FLAVOPIRIDOL_TREATMENT_TP53	-0,15	4E-05	5,7E-08	0,0006
MODULE_69	-0,15	4E-05	5,7E-08	0,0006
V$E2F_02	-0,202	3E-08	5,7E-08	0,0006
V$VDR_Q6	-0,137	2E-04	5,8E-08	0,0006
MODULE_154	-0,203	2E-08	5,8E-08	0,0006
DING_LUNG_CANCER_BY_MUTATION_RATE	-0,12	0,001	5,9E-08	0,0006
TGACGTCA_V$ATF3_Q6	-0,185	4E-07	5,9E-08	0,0006
LIM_MAMMARY_LUMINAL_MATURE_DN	-0,184	4E-07	6E-08	0,0006
V$POU3F2_01	-0,171	3E-06	6E-08	0,0006
BENPORATH_ES_CORE_NINE_CORRELATED	-0,175	2E-06	6,1E-08	0,0006
V$ALPHACP1_01	-0,18	8E-07	6,3E-08	0,0006
CSR_LATE_UP.V1_UP	-0,209	8E-09	6,4E-08	0,0006
GCANCTGNY_V$MYOD_Q6	-0,138	2E-04	6,4E-08	0,0006
GSE360_L_MAJOR_VS_B_MALAYI_LOW_DOS	-0,193	1E-07	6,4E-08	0,0006
V$OCT_C	-0,167	5E-06	6,4E-08	0,0006
PASTURAL_RIZ1_TARGETS_DN	-0,163	8E-06	6,5E-08	0,0006
ORGANELLE_PART	-0,218	2E-09	6,5E-08	0,0006
PDGF_ERK_DN.V1_DN	-0,151	4E-05	6,6E-08	0,0007
REGULATION_OF_GENE_EXPRESSION_EPIGENE	-0,192	1E-07	6,8E-08	0,0007
GSE14308_TH1_VS_NAIVE_CD4_TCELL_DN	-0,158	1E-05	6,8E-08	0,0007
GNF2_HDAC1	-0,208	1E-08	7,1E-08	0,0007
VETTER_TARGETS_OF_PRKCA_AND_ETS1_UI	-0,219	1E-09	7,2E-08	0,0007
GSE17721_LPS_VS_PAM3CSK4_2H_BMDM_I	-0,176	1E-06	7,3E-08	0,0007
TGCGCANK_UNKNOWN	-0,174	2E-06	7,5E-08	0,0007
MODULE_316	-0,154	2E-05	7,5E-08	0,0007
MODULE_72	-0,221	1E-09	7,7E-08	0,0008

MODULE_105	-0,217	2E-09	7,8E-08	0,0008
YAGI_AML_WITH_INV_16_TRANSLOCATION	-0,167	5E-06	7,9E-08	0,0008
V$LHX3_01	-0,151	4E-05	8E-08	0,0008
V$E2A_Q2	-0,163	8E-06	8,3E-08	0,0008
V$RFX1_02	-0,104	0,004	8,3E-08	0,0008
DAVICIONI_PAX_FOXO1_SIGNATURE_IN_ARMS	-0,198	5E-08	8,4E-08	0,0008
V$GRE_C	-0,161	1E-05	8,4E-08	0,0008
KAECH_NAIVE_VS_DAY8_EFF_CD8_TCELL_U	-0,14	1E-04	8,5E-08	0,0008
V$IK3_01	-0,129	4E-04	8,6E-08	0,0009
GAZDA_DIAMOND_BLACKFAN_ANEMIA_ERYTI	-0,14	1E-04	8,7E-08	0,0009
V$E2F1_Q3_01	-0,191	2E-07	8,9E-08	0,0009
INTRACELLULAR_ORGANELLE_PART	-0,216	3E-09	9E-08	0,0009
REACTOME_SMAD2_SMAD3_SMAD4_HETERO	-0,2	4E-08	9E-08	0,0009
WANG_CISPLATIN_RESPONSE_AND_XPC_UP	-0,196	7E-08	9,2E-08	0,0009
V$TCF1P_Q6	-0,182	5E-07	9,3E-08	0,0009
V$ATF_B	-0,176	1E-06	9,3E-08	0,0009
GSE29618_LAIV_VS_TIV_FLU_VACCINE_DAY	-0,175	2E-06	9,3E-08	0,0009
MODULE_329	-0,132	3E-04	9,3E-08	0,0009
KAAB_HEART_ATRIUM_VS_VENTRICLE_UP	-0,136	2E-04	9,6E-08	0,001
MONOVALENT_INORGANIC_CATION_TRANSPC	-0,122	8E-04	9,7E-08	0,001
MODULE_387	-0,136	2E-04	9,8E-08	0,001
ASGHARZADEH_NEUROBLASTOMA_POOR_SUI	-0,154	2E-05	9,8E-08	0,001
CERVERA_SDHB_TARGETS_2	-0,158	2E-05	9,8E-08	0,001
INORGANIC_ANION_TRANSMEMBRANE_TRANS	-0,187	3E-07	9,8E-08	0,001
GSE29618_PRE_VS_DAY7_POST_LAIV_FLU_	-0,144	8E-05	9,9E-08	0,001
GSE26495_NAIVE_VS_PD1HIGH_CD8_TCELL_	-0,112	0,002	9,9E-08	0,001
V$USF_C	-0,189	2E-07	9,9E-08	0,001
MODULE_197	-0,196	7E-08	1E-07	0,001
GSE10239_NAIVE_VS_DAY4.5_EFF_CD8_TCI	-0,129	4E-04	1E-07	0,001
GSE17721_LPS_VS_CPG_0.5H_BMDM_UP	-0,146	6E-05	1E-07	0,001
SETLUR_PROSTATE_CANCER_TMPRSS2_ERG_	-0,165	6E-06	1,1E-07	0,0011
GSE24634_NAIVE_CD4_TCELL_VS_DAY10_II	-0,126	6E-04	1,1E-07	0,0011
V$MEIS1BHOXA9_01	-0,18	8E-07	1,1E-07	0,0011
GLI1_UP.V1_UP	-0,135	2E-04	1,1E-07	0,0011
ZIRN_TRETINOIN_RESPONSE_WT1_UP	-0,175	2E-06	1,1E-07	0,0011
V$AP2_Q6	-0,159	1E-05	1,1E-07	0,0011
PTEN_DN.V1_UP	-0,156	2E-05	1,1E-07	0,0011
GSE13485_CTRL_VS_DAY7_YF17D_VACCINE	-0,175	1E-06	1,1E-07	0,0011
GSE19825_NAIVE_VS_IL2RAHIGH_DAY3_EFF	-0,134	2E-04	1,1E-07	0,0011
V$AP3_Q6	-0,163	8E-06	1,1E-07	0,0011
MIKKELSEN_NPC_ICP_WITH_H3K4ME3	-0,131	4E-04	1,1E-07	0,0011
chr18q11	-0,176	1E-06	1,1E-07	0,0011

GSE9650_NAIVE_VS_EFF_CD8_TCELL_UP	-0,151	4E-05	1,1E-07	0,0011
AKT_UP_MTOR_DN.V1_DN	-0,156	2E-05	1,1E-07	0,0011
V$FREAC3_01	-0,153	3E-05	1,1E-07	0,0011
FOURNIER_ACINAR_DEVELOPMENT_LATE_UP	-0,186	3E-07	1,2E-07	0,0011
GSE36476_YOUNG_VS_OLD_DONOR_MEMORY	-0,163	7E-06	1,2E-07	0,0012
CAGGTG_V$E12_Q6	-0,143	9E-05	1,2E-07	0,0012
MEIOTIC_CELL_CYCLE	-0,204	2E-08	1,2E-07	0,0012
V$CREBP1_01	-0,158	1E-05	1,2E-07	0,0012
JAATINEN_HEMATOPOIETIC_STEM_CELL_UP	-0,17	3E-06	1,2E-07	0,0012
GSE9006_TYPE_1_DIABETES_AT_DX_VS_1M	-0,133	3E-04	1,2E-07	0,0012
COLDREN_GEFITINIB_RESISTANCE_UP	-0,208	1E-08	1,2E-07	0,0012
TGTYNNNNNRGCARM_UNKNOWN	-0,09	0,014	1,2E-07	0,0012
PID_P75NTRPATHWAY	-0,159	1E-05	1,2E-07	0,0012
TURASHVILI_BREAST_DUCTAL_CARCINOMA_V	-0,159	1E-05	1,2E-07	0,0012
RICKMAN_TUMOR_DIFFERENTIATED_WELL_VS	-0,171	3E-06	1,2E-07	0,0012
GSE17721_CTRL_VS_PAM3CSK4_0.5H_BMDM	-0,17	3E-06	1,3E-07	0,0012
V$CREB_01	-0,179	8E-07	1,3E-07	0,0013
ORGANELLE_ORGANIZATION_AND_BIOGENESIS	-0,191	2E-07	1,3E-07	0,0013
CACCAGC,MIR-138	-0,163	8E-06	1,3E-07	0,0013
MULLIGHAN_MLL_SIGNATURE_2_DN	-0,16	1E-05	1,3E-07	0,0013
V$FOXM1_01	-0,169	4E-06	1,3E-07	0,0013
MODULE_136	-0,153	3E-05	1,3E-07	0,0013
YCATTAA_UNKNOWN	-0,143	9E-05	1,3E-07	0,0013
GSE17721_CTRL_VS_CPG_6H_BMDM_DN	-0,192	1E-07	1,3E-07	0,0013
GSE1460_NAIVE_CD4_TCELL_CORD_BLOOD_	-0,154	2E-05	1,3E-07	0,0013
V$FREAC7_01	-0,163	7E-06	1,4E-07	0,0013
GSE11924_TFH_VS_TH2_CD4_TCELL_DN	-0,198	5E-08	1,4E-07	0,0014
GSE29618_LAIV_VS_TIV_FLU_VACCINE_DAY	-0,166	5E-06	1,4E-07	0,0014
GSE15324_ELF4_KO_VS_WT_NAIVE_CD8_TC	-0,149	4E-05	1,4E-07	0,0014
GGGAGGRR_V$MAZ_Q6	-0,143	9E-05	1,4E-07	0,0014
V$MYOD_Q6_01	-0,156	2E-05	1,4E-07	0,0014
E2F1_UP.V1_UP	-0,194	1E-07	1,4E-07	0,0014
CTTTGT_V$LEF1_Q2	-0,165	6E-06	1,4E-07	0,0014
NOTCH_DN.V1_UP	-0,163	8E-06	1,4E-07	0,0014
GSE3982_MAC_VS_TH2_DN	-0,189	2E-07	1,4E-07	0,0014
TGAYRTCA_V$ATF3_Q6	-0,16	1E-05	1,4E-07	0,0014
V$YY1_02	-0,123	8E-04	1,4E-07	0,0014
NF_KAPPAB_BINDING	-0,201	3E-08	1,5E-07	0,0014
GSE29618_LAIV_VS_TIV_FLU_VACCINE_DAY	-0,181	7E-07	1,5E-07	0,0015
V$ERR1_Q2	-0,182	6E-07	1,5E-07	0,0015
TAYLOR_METHYLATED_IN_ACUTE_LYMPHOBL	-0,19	2E-07	1,5E-07	0,0015
GTTRYCATRR_UNKNOWN	-0,093	0,011	1,5E-07	0,0015

WILCOX_PRESPONSE_TO_ROGESTERONE_UP	-0,214	4E-09	1,5E-07	0,0015
V$SP1_Q2_01	-0,138	2E-04	1,5E-07	0,0015
CAAGGAT,MIR-362	-0,149	4E-05	1,6E-07	0,0015
V$AP4_Q6	-0,148	5E-05	1,6E-07	0,0015
LEE_AGING_CEREBELLUM_DN	-0,17	3E-06	1,6E-07	0,0016
DAVICIONI_MOLECULAR_ARMS_VS_ERMS_UP	-0,147	5E-05	1,6E-07	0,0016
GSE14769_UNSTIM_VS_40MIN_LPS_BMDM_U	-0,145	7E-05	1,6E-07	0,0016
ALFANO_MYC_TARGETS	-0,196	7E-08	1,6E-07	0,0016
GSE14308_TH1_VS_TH17_UP	-0,201	3E-08	1,6E-07	0,0016
GALIE_TUMOR_STEMNESS_GENES	-0,176	1E-06	1,6E-07	0,0016
MODY_HIPPOCAMPUS_NEONATAL	-0,23	2E-10	1,6E-07	0,0016
BROWNE_HCMV_INFECTION_4HR_DN	-0,166	6E-06	1,7E-07	0,0016
GSE11924_TFH_VS_TH1_CD4_TCELL_DN	-0,15	4E-05	1,7E-07	0,0017
MODULE_357	-0,198	5E-08	1,7E-07	0,0017
V$MYCMAX_B	-0,172	2E-06	1,7E-07	0,0017
CTCTAGA,MIR-526C,MIR-518F,MIR-526A	-0,155	2E-05	1,7E-07	0,0017
TSENG_ADIPOGENIC_POTENTIAL_UP	-0,177	1E-06	1,7E-07	0,0017
V$NKX62_Q2	-0,15	4E-05	1,7E-07	0,0017
YAO_TEMPORAL_RESPONSE_TO_PROGESTERO	-0,17	3E-06	1,7E-07	0,0017
MULLIGHAN_MLL_SIGNATURE_1_DN	-0,142	1E-04	1,7E-07	0,0017
WHITFIELD_CELL_CYCLE_G2	-0,187	3E-07	1,7E-07	0,0017
V$NKX61_01	-0,156	2E-05	1,8E-07	0,0017
DORN_ADENOVIRUS_INFECTION_32HR_DN	-0,165	6E-06	1,8E-07	0,0017
WANG_IMMORTALIZED_BY_HOXA9_AND_MEIS	-0,13	4E-04	1,8E-07	0,0017
KATSANOU_ELAVL1_TARGETS_DN	-0,193	1E-07	1,8E-07	0,0017
GSE17721_CPG_VS_GARDIQUIMOD_2H_BMDM	-0,181	7E-07	1,8E-07	0,0018
HUI_MAPK14_TARGETS_UP	-0,158	1E-05	1,8E-07	0,0018
SYATTGTG_UNKNOWN	-0,152	3E-05	1,8E-07	0,0018
DNA_DEPENDENT_DNA_REPLICATION	-0,192	1E-07	1,8E-07	0,0018
POOLA_INVASIVE_BREAST_CANCER_DN	-0,058	0,116	1,9E-07	0,0018
V$CREBP1_Q2	-0,155	2E-05	1,9E-07	0,0018
CCAGGGG,MIR-331	-0,149	4E-05	1,9E-07	0,0018
MODULE_297	-0,197	6E-08	1,9E-07	0,0019
CCAWYNNGAAR_UNKNOWN	-0,202	3E-08	1,9E-07	0,0019
REGULATION_OF_GENE_EXPRESSION	-0,15	4E-05	1,9E-07	0,0019
GATTGGY_V$NFY_Q6_01	-0,187	3E-07	1,9E-07	0,0019
ESC_J1_UP_LATE.V1_DN	-0,159	1E-05	1,9E-07	0,0019
GSE12366_GC_VS_MEMORY_BCELL_UP	-0,2	4E-08	1,9E-07	0,0019
REGULATION_OF_CELLULAR_COMPONENT_OF	-0,148	5E-05	2E-07	0,0019
REACTOME_SIGNALING_BY_NOTCH	-0,162	9E-06	2E-07	0,0019
V$CHX10_01	-0,145	7E-05	2E-07	0,0019
KORKOLA_YOLK_SAC_TUMOR	-0,13	4E-04	2E-07	0,002

GAGCCAG,MIR-149	-0,154	2E-05	2E-07	0,002
GSE15930_STIM_VS_STIM_AND_TRICHOSTA1	-0,14	1E-04	2E-07	0,002
V$CP2_01	-0,148	5E-05	2E-07	0,002
HERNANDEZ_MITOTIC_ARREST_BY_DOCETAXE	-0,21	8E-09	2E-07	0,002
PID_INTEGRIN4_PATHWAY	-0,175	2E-06	2,1E-07	0,0021
MITOTIC_CELL_CYCLE	-0,184	4E-07	2,1E-07	0,0021
AGTTCTC,MIR-146A,MIR-146B	-0,156	2E-05	2,1E-07	0,0021
DELAYED_RECTIFIER_POTASSIUM_CHANNEL_/	-0,153	3E-05	2,1E-07	0,0021
GSE26928_CENTR_MEMORY_VS_CXCR5_POS_	-0,144	8E-05	2,2E-07	0,0021
MORF_LCAT	-0,144	8E-05	2,2E-07	0,0021
V$ZID_01	-0,166	5E-06	2,2E-07	0,0022
GSE9988_LPS_VS_LOW_LPS_MONOCYTE_UP	-0,14	1E-04	2,2E-07	0,0022
chr17q24	-0,093	0,011	2,2E-07	0,0022
GSE360_DC_VS_MAC_L_MAJOR_DN	-0,153	3E-05	2,3E-07	0,0022
V$PAX6_01	-0,168	4E-06	2,3E-07	0,0022
V$CMYB_01	-0,126	6E-04	2,3E-07	0,0022
MCCLUNG_COCAINE_REWARD_5D	-0,128	5E-04	2,3E-07	0,0022
VANASSE_BCL2_TARGETS_UP	-0,165	6E-06	2,3E-07	0,0023
MCCLUNG_DELTA_FOSB_TARGETS_2WK	-0,182	6E-07	2,3E-07	0,0023
GSE19825_NAIVE_VS_IL2RALOW_DAY3_EFF	-0,15	4E-05	2,3E-07	0,0023
CAIRO_LIVER_DEVELOPMENT_UP	-0,166	5E-06	2,3E-07	0,0023
GSE360_L_MAJOR_VS_T_GONDII_MAC_DN	-0,206	1E-08	2,3E-07	0,0023
REGULATION_OF_AXONOGENESIS	-0,119	0,001	2,3E-07	0,0023
GSE27786_LIN_NEG_VS_MONO_MAC_UP	-0,205	2E-08	2,3E-07	0,0023
GSE27786_NKCELL_VS_NEUTROPHIL_DN	-0,104	0,004	2,3E-07	0,0023
GGGYGTGNY_UNKNOWN	-0,15	4E-05	2,3E-07	0,0023
REACTOME_MYOGENESIS	-0,193	1E-07	2,4E-07	0,0023
V$E2F1_Q6_01	-0,193	1E-07	2,4E-07	0,0023
GSE14350_TREG_VS_TEFF_IN_IL2RB_KO_DN	-0,198	5E-08	2,4E-07	0,0023
MODULE_458	-0,151	3E-05	2,4E-07	0,0023
BIOCARTA_AGR_PATHWAY	-0,193	1E-07	2,4E-07	0,0024
CAGNWMCNNNGAC_UNKNOWN	-0,181	6E-07	2,4E-07	0,0024
PRC2_EDD_UP.V1_UP	-0,19	2E-07	2,5E-07	0,0024
GSE20366_EX_VIVO_VS_HOMEOSTATIC_CON	-0,191	2E-07	2,5E-07	0,0024
V$EGR3_01	-0,196	7E-08	2,5E-07	0,0024
RICKMAN_HEAD_AND_NECK_CANCER_A	-0,182	5E-07	2,5E-07	0,0024
chr15q26	-0,182	6E-07	2,5E-07	0,0025
GSE3982_EOSINOPHIL_VS_BCELL_DN	-0,207	1E-08	2,6E-07	0,0025
BREUHAHN_GROWTH_FACTOR_SIGNALING_IN_	-0,113	0,002	2,6E-07	0,0025
GARGALOVIC_RESPONSE_TO_OXIDIZED_PHOS	-0,207	1E-08	2,6E-07	0,0025
PROTEIN_COMPLEX	-0,189	2E-07	2,6E-07	0,0025
LIU_PROSTATE_CANCER_DN	-0,144	8E-05	2,6E-07	0,0025

GSE29618_PRE_VS_DAY7_FLU_VACCINE_MD	-0,156	2E-05	2,6E-07	0,0026
SEQUENCE_SPECIFIC_DNA_BINDING	-0,173	2E-06	2,6E-07	0,0026
BHAT_ESR1_TARGETS_VIA_AKT1_UP	-0,089	0,015	2,7E-07	0,0026
KORKOLA_EMBRYONIC_CARCINOMA_VS_SEMI	-0,186	3E-07	2,7E-07	0,0026
KEGG_GAP_JUNCTION	-0,157	2E-05	2,8E-07	0,0027
CELL_CYCLE_PHASE	-0,183	5E-07	2,8E-07	0,0027
SANSOM_APC_TARGETS_UP	-0,148	5E-05	2,8E-07	0,0027
GSE6269_FLU_VS_E_COLI_INF_PBMC_DN	-0,152	3E-05	2,8E-07	0,0028
CCATCCA,MIR-432	-0,148	5E-05	2,8E-07	0,0028
KEGG_CHRONIC_MYELOID_LEUKEMIA	-0,179	9E-07	2,8E-07	0,0028
FIGUEROA_AML_METHYLATION_CLUSTER_1_U	-0,156	2E-05	2,8E-07	0,0028
V$GATA1_01	-0,177	1E-06	2,9E-07	0,0028
GSE7460_CTRL_VS_TGFB_TREATED_ACT_CD	-0,176	1E-06	2,9E-07	0,0028
V$FOXO4_01	-0,145	7E-05	2,9E-07	0,0028
REACTOME_TGF_BETA_RECEPTOR_SIGNALING	-0,21	7E-09	2,9E-07	0,0028
GGCNRNWCTTYS_UNKNOWN	-0,126	6E-04	2,9E-07	0,0028
REACTOME_SIGNALING_BY_EGFR_IN_CANCER	-0,146	6E-05	2,9E-07	0,0028
CYTOSKELETAL_PART	-0,204	2E-08	2,9E-07	0,0029
LINDGREN_BLADDER_CANCER_CLUSTER_3_D	-0,091	0,013	2,9E-07	0,0029
SIMBULAN_UV_RESPONSE_NORMAL_DN	-0,195	8E-08	3E-07	0,0029
LTE2_UP.V1_DN	-0,169	4E-06	3E-07	0,0029
MORF_FRK	-0,135	2E-04	3E-07	0,0029
GSE13411_NAIVE_VS_SWITCHED_MEMORY_E	-0,145	8E-05	3E-07	0,0029
MACROMOLECULAR_COMPLEX_DISASSEMBLY	-0,21	7E-09	3E-07	0,003
V$OLF1_01	-0,142	1E-04	3,1E-07	0,003
REGULATION_OF_METABOLIC_PROCESS	-0,157	2E-05	3,1E-07	0,003
GSE7460_FOXP3_MUT_VS_HET_ACT_TCONV	-0,17	3E-06	3,1E-07	0,003
REACTOME_PI3K_EVENTS_IN_ERBB2_SIGNALI	-0,137	2E-04	3,1E-07	0,003
V$T3R_Q6	-0,138	2E-04	3,1E-07	0,003
GSE27786_LSK_VS_CD8_TCELL_DN	-0,168	4E-06	3,1E-07	0,003
V$MYOD_01	-0,162	9E-06	3,1E-07	0,0031
PROTEIN_COMPLEX_DISASSEMBLY	-0,211	6E-09	3,2E-07	0,0031
ZWANG_EGF_INTERVAL_UP	-0,18	7E-07	3,2E-07	0,0031
GSE15930_NAIVE_VS_72H_IN_VITRO_STIM_	-0,17	3E-06	3,2E-07	0,0031
MCBRYAN_PUBERTAL_BREAST_6_7WK_DN	-0,194	9E-08	3,2E-07	0,0031
GSE20715_0H_VS_48H_OZONE_LUNG_DN	-0,208	1E-08	3,2E-07	0,0031
DUAN_PRDM5_TARGETS	-0,158	1E-05	3,2E-07	0,0031
V$TST1_01	-0,166	5E-06	3,2E-07	0,0031
SCHAEFFER_PROSTATE_DEVELOPMENT_6HR_	-0,19	2E-07	3,3E-07	0,0032
BACOLOD_RESISTANCE_TO_ALKYLATING_AG	-0,157	2E-05	3,3E-07	0,0032
GOLGI_STACK	0,19	2E-07	3,3E-07	0,0032
V$MYOD_Q6	-0,154	2E-05	3,3E-07	0,0032

WGGAATGY_V$TEF1_Q6	-0,147	5E-05	3,3E-07	0,0032
REACTOME_POTASSIUM_CHANNELS	-0,166	5E-06	3,3E-07	0,0032
GSE17721_CTRL_VS_POLYIC_2H_BMDM_DN	-0,16	1E-05	3,4E-07	0,0033
MORF_SNRP70	-0,176	1E-06	3,5E-07	0,0034
V$MTF1_Q4	-0,149	4E-05	3,5E-07	0,0034
HIRSCH_CELLULAR_TRANSFORMATION_SIGNA	-0,195	7E-08	3,5E-07	0,0034
ACACTCC,MIR-122A	-0,138	2E-04	3,5E-07	0,0034
GSE17721_CTRL_VS_CPG_0.5H_BMDM_DN	-0,17	3E-06	3,5E-07	0,0034
WATTEL_AUTONOMOUS_THYROID_ADENOMA	-0,126	6E-04	3,5E-07	0,0034
GTGGTGA,MIR-197	-0,167	5E-06	3,6E-07	0,0035
CORRADETTI_MTOR_PATHWAY_REGULATORS_	-0,193	1E-07	3,6E-07	0,0035
V$HFH1_01	-0,153	3E-05	3,7E-07	0,0035
BLALOCK_ALZHEIMERS_DISEASE_UP	-0,15	4E-05	3,7E-07	0,0036
REACTOME_APOPTOTIC_CLEAVAGE_OF_CELL	-0,194	9E-08	3,7E-07	0,0036
MODULE_67	-0,149	4E-05	3,7E-07	0,0036
CROMER_METASTASIS_DN	-0,195	8E-08	3,8E-07	0,0036
BIOCARTA_ACTINY_PATHWAY	-0,221	1E-09	3,8E-07	0,0036
VERHAAK_GLIOBLASTOMA_CLASSICAL	-0,129	4E-04	3,8E-07	0,0037
chr1q43	-0,163	8E-06	3,8E-07	0,0037
MIKKELSEN_ES_ICP_WITH_H3K4ME3_AND_H3	-0,137	2E-04	3,8E-07	0,0037
REACTOME_SOS_MEDIATED_SIGNALLING	-0,16	1E-05	3,8E-07	0,0037
OSMAN_BLADDER_CANCER_DN	-0,159	1E-05	3,9E-07	0,0038
REGULATION_OF_CELLULAR_METABOLIC_PRC	-0,157	2E-05	3,9E-07	0,0038
TGCCAAR_V$NF1_Q6	-0,139	1E-04	3,9E-07	0,0038
BIOCARTA_CDK5_PATHWAY	-0,158	1E-05	3,9E-07	0,0038
GSE27786_CD8_TCELL_VS_MONO_MAC_DN	-0,188	2E-07	4E-07	0,0039
GAMETE_GENERATION	-0,134	2E-04	4E-07	0,0039
LOPES_METHYLATED_IN_COLON_CANCER_DN	-0,137	2E-04	4E-07	0,0039
SHEPARD_BMYB_MORPHOLINO_DN	-0,189	2E-07	4E-07	0,0039
PID_ERA_GENOMIC_PATHWAY	-0,045	0,216	4E-07	0,0039
PEDRIOLI_MIR31_TARGETS_UP	-0,169	4E-06	4E-07	0,0039
REACTOME_SIGNALING_BY_BMP	-0,138	2E-04	4,1E-07	0,004
V$AP4_Q6_01	-0,15	4E-05	4,2E-07	0,004
DORN_ADENOVIRUS_INFECTION_48HR_DN	-0,163	8E-06	4,2E-07	0,004
GSE26669_CD4_VS_CD8_TCELL_IN_MLR_UP	-0,172	2E-06	4,2E-07	0,004
GSE13306_RA_VS_UNTREATED_TREG_UP	-0,155	2E-05	4,2E-07	0,004
REACTOME_TIGHT_JUNCTION_INTERACTIONS	-0,175	1E-06	4,2E-07	0,0041
BENPORATH_OCT4_TARGETS	-0,18	8E-07	4,3E-07	0,0041
GSE17721_CPG_VS_GARDIQUIMOD_0.5H_BM	-0,146	6E-05	4,3E-07	0,0041
chr1p31	-0,166	5E-06	4,3E-07	0,0041
IDENTICAL_PROTEIN_BINDING	-0,158	1E-05	4,3E-07	0,0041
CCAWNWWNNNGGC_UNKNOWN	-0,186	3E-07	4,3E-07	0,0042

GSE29618_PRE_VS_DAY7_POST_LAIV_FLU_	-0,17	3E-06	4,3E-07	0,0042
MODULE_37	-0,127	5E-04	4,3E-07	0,0042
NAGY_TFTC_COMPONENTS_HUMAN	-0,182	6E-07	4,4E-07	0,0042
GSE29614_CTRL_VS_TIV_FLU_VACCINE_PBM	-0,184	4E-07	4,4E-07	0,0043
CACGTG_V$MYC_Q2	-0,174	2E-06	4,5E-07	0,0043
VECCHI_GASTRIC_CANCER_EARLY_DN	-0,118	0,001	4,5E-07	0,0043
GSE6269_E_COLI_VS_STREP_AUREUS_INF_P	-0,151	3E-05	4,5E-07	0,0043
GNF2_DNM1	-0,135	2E-04	4,5E-07	0,0043
GSE34205_RSV_VS_FLU_INF_INFANT_PBMC_	-0,109	0,003	4,5E-07	0,0044
HOELZEL_NF1_TARGETS_UP	-0,173	2E-06	4,6E-07	0,0044
V$DR3_Q4	-0,162	9E-06	4,6E-07	0,0044
GSE25087_TREG_VS_TCONV_ADULT_DN	-0,119	0,001	4,6E-07	0,0045
ABRAHAM_ALPC_VS_MULTIPLE_MYELOMA_D	-0,184	4E-07	4,6E-07	0,0045
LEE_NEURAL_CREST_STEM_CELL_DN	-0,172	2E-06	4,6E-07	0,0045
ACAGGGT,MIR-10A,MIR-10B	-0,194	1E-07	4,7E-07	0,0045
KEGG_FOCAL_ADHESION	-0,163	8E-06	4,7E-07	0,0045
CAGCTG_V$AP4_Q5	-0,134	3E-04	4,7E-07	0,0045
SCHAEFFER_PROSTATE_DEVELOPMENT_AND_	-0,158	1E-05	4,7E-07	0,0046
BRUINS_UVC_RESPONSE_MIDDLE	-0,19	2E-07	4,8E-07	0,0046
TURASHVILI_BREAST_LOBULAR_CARCINOMA_	-0,154	2E-05	4,8E-07	0,0046
AGGGCCA,MIR-328	-0,176	1E-06	4,8E-07	0,0046
GSE7852_LN_VS_THYMUS_TREG_DN	-0,141	1E-04	4,8E-07	0,0046
NAKAMURA_LUNG_CANCER	-0,223	7E-10	4,8E-07	0,0047
CHIARADONNA_NEOPLASTIC_TRANSFORMATI	-0,215	3E-09	4,9E-07	0,0047
CALCIUM_INDEPENDENT_CELL_CELL_ADHESI	-0,178	1E-06	4,9E-07	0,0047
V$PIT1_Q6	-0,15	4E-05	4,9E-07	0,0048
CHICAS_RB1_TARGETS_GROWING	-0,202	2E-08	4,9E-07	0,0048
VERHAAK_GLIOBLASTOMA_MESENCHYMAL	-0,121	9E-04	4,9E-07	0,0048
STK33_SKM_DN	-0,167	4E-06	5,1E-07	0,0049
V$CDP_02	-0,139	1E-04	5,1E-07	0,0049
NUCLEAR_PART	-0,196	6E-08	5,2E-07	0,005
GSE15767_MED_VS_SCS_MAC_LN_DN	-0,154	2E-05	5,2E-07	0,005
V$GFI1_01	-0,145	7E-05	5,2E-07	0,005
GSE28237_FOLLICULAR_VS_EARLY_GC_BCEL	-0,209	8E-09	5,2E-07	0,005
NUCLEAR_LUMEN	-0,201	3E-08	5,2E-07	0,005
ACCTGTTG_UNKNOWN	-0,143	8E-05	5,2E-07	0,005
PID_AURORA_B_PATHWAY	-0,183	5E-07	5,3E-07	0,0051
BROWNE_HCMV_INFECTION_14HR_DN	-0,171	3E-06	5,3E-07	0,0051
V$HFH8_01	-0,125	6E-04	5,4E-07	0,0052
GSE27786_LIN_NEG_VS_CD8_TCELL_UP	-0,2	3E-08	5,4E-07	0,0052
GSE3982_DC_VS_TH1_DN	-0,195	9E-08	5,4E-07	0,0052
PID_LIS1PATHWAY	-0,223	8E-10	5,5E-07	0,0053

NADELLA_PRKAR1A_TARGETS_DN	-0,185	4E-07	5,6E-07	0,0054
DOANE_BREAST_CANCER_CLASSES_DN	-0,183	5E-07	5,6E-07	0,0054
SHEPARD_BMYB_TARGETS	-0,189	2E-07	5,7E-07	0,0055
IWANAGA_CARCINOGENESIS_BY_KRAS_PTEN_	-0,161	1E-05	5,7E-07	0,0055
YAP1_UP	-0,148	5E-05	5,8E-07	0,0056
GSE36476_CTRL_VS_TSST_ACT_72H_MEMO	-0,121	9E-04	5,8E-07	0,0056
PID_P53REGULATIONPATHWAY	-0,191	1E-07	5,8E-07	0,0056
V$SMAD_Q6	-0,156	2E-05	5,9E-07	0,0056
POTASSIUM_CHANNEL_REGULATOR_ACTIVITY	-0,14	1E-04	5,9E-07	0,0057
GSE29618_MONOCYTE_VS_PDC_DN	-0,176	1E-06	6E-07	0,0058
SIMBULAN_PARP1_TARGETS_DN	-0,196	7E-08	6E-07	0,0058
MYOBLAST_DIFFERENTIATION	-0,11	0,003	6E-07	0,0058
V$STAT_Q6	-0,164	7E-06	6E-07	0,0058
APICAL_JUNCTION_COMPLEX	-0,145	7E-05	6,2E-07	0,006
APICOLATERAL_PLASMA_MEMBRANE	-0,145	7E-05	6,2E-07	0,006
V$GATA3_01	-0,13	4E-04	6,2E-07	0,006
V$SP1_01	-0,112	0,002	6,3E-07	0,006
KANG_IMMORTALIZED_BY_TERT_DN	-0,138	2E-04	6,3E-07	0,006
STRUCTURAL_MOLECULE_ACTIVITY	-0,165	6E-06	6,3E-07	0,006
CACTGCC,MIR-34A,MIR-34C,MIR-449	-0,147	6E-05	6,3E-07	0,006
KYNG_ENVIRONMENTAL_STRESS_RESPONSE_	-0,144	8E-05	6,3E-07	0,006
REACTOME_ACTIVATION_OF_RAC	-0,183	5E-07	6,3E-07	0,006
PLASARI_TGFB1_SIGNALING_VIA_NFIC_10HR_	-0,162	9E-06	6,3E-07	0,0061
CELL_CYCLE_PROCESS	-0,178	1E-06	6,3E-07	0,0061
V$FOXO3_01	-0,124	7E-04	6,4E-07	0,0061
chr15q24	-0,175	1E-06	6,4E-07	0,0061
GSE1460_INTRATHYMIC_T_PROGENITOR_VS_	-0,171	3E-06	6,4E-07	0,0061
GSE27786_LIN_NEG_VS_NEUTROPHIL_UP	-0,165	6E-06	6,4E-07	0,0062
NRL_DN.V1_DN	-0,144	8E-05	6,5E-07	0,0063
METAL_ION_TRANSPORT	-0,117	0,001	6,5E-07	0,0063
YANG_BREAST_CANCER_ESR1_LASER_DN	-0,188	2E-07	6,5E-07	0,0063
GSE17721_PAM3CSK4_VS_GADIQUIMOD_2H_	-0,127	5E-04	6,7E-07	0,0064
WANG_TUMOR_INVASIVENESS_UP	-0,202	3E-08	6,7E-07	0,0064
V$SOX9_B1	-0,152	3E-05	6,7E-07	0,0064
ACAWYAAAG_UNKNOWN	-0,157	2E-05	6,7E-07	0,0065
GOBERT_CORE_OLIGODENDROCYTE_DIFFEREN	-0,16	1E-05	6,8E-07	0,0065
GSE3982_MAST_CELL_VS_DC_UP	-0,153	3E-05	6,8E-07	0,0066
PID_P73PATHWAY	-0,193	1E-07	6,9E-07	0,0066
LEE_LIVER_CANCER_SURVIVAL_DN	-0,191	1E-07	6,9E-07	0,0067
GSE27786_CD8_TCELL_VS_ERYTHROBLAST_	-0,111	0,002	7E-07	0,0067
GSE13493_CD4INTCD8POS_VS_CD8POS_THY	-0,142	1E-04	7E-07	0,0067
PID_IGF1_PATHWAY	-0,129	4E-04	7,2E-07	0,0069

ACCGAGC,MIR-423	-0,174	2E-06	7,2E-07	0,0069
CHENG_IMPRINTED_BY_ESTRADIOL	-0,176	1E-06	7,3E-07	0,007
SRC_UP.V1_DN	-0,204	2E-08	7,3E-07	0,007
GSE26928_NAIVE_VS_EFF_MEMORY_CD4_TC	-0,097	0,008	7,3E-07	0,007
OUELLET_CULTURED_OVARIAN_CANCER_INV/	-0,203	2E-08	7,3E-07	0,007
V$ATF_01	-0,158	1E-05	7,4E-07	0,0071
MODULE_179	-0,156	2E-05	7,4E-07	0,0071
LI_WILMS_TUMOR_VS_FETAL_KIDNEY_1_DN	-0,183	5E-07	7,4E-07	0,0071
CELL_CELL_ADHESION	-0,16	1E-05	7,4E-07	0,0071
REACTOME_SIGNALLING_BY_NGF	-0,122	9E-04	7,5E-07	0,0071
CTCAAGA,MIR-526B	-0,183	5E-07	7,5E-07	0,0072
ZHONG_RESPONSE_TO_AZACITIDINE_AND_TS	-0,169	4E-06	7,5E-07	0,0072
VOLTAGE_GATED_POTASSIUM_CHANNEL_AC	-0,14	1E-04	7,7E-07	0,0073
REACTOME_SIGNALING_BY_PDGF	-0,148	5E-05	7,7E-07	0,0074
BASEMENT_MEMBRANE	-0,143	9E-05	7,7E-07	0,0074
V$PAX8_01	-0,127	5E-04	7,8E-07	0,0075
V$PPARA_02	-0,156	2E-05	7,8E-07	0,0075
GSE7400_CTRL_VS_CSF3_IN_VIVO_TREATEI	-0,188	2E-07	7,8E-07	0,0075
SKELETAL_MUSCLE_DEVELOPMENT	-0,133	3E-04	7,9E-07	0,0075
KAMMINGA_SENESCENCE	-0,153	3E-05	7,9E-07	0,0076
GSE3982_EOSINOPHIL_VS_CENT_MEMORY_CI	-0,174	2E-06	7,9E-07	0,0076
POSITIVE_REGULATION_OF_CELL_CYCLE	-0,198	5E-08	8E-07	0,0076
WANG_LSD1_TARGETS_DN	-0,155	2E-05	8E-07	0,0076
GSE13411_IGM_VS_SWITCHED_MEMORY_BCE	-0,15	4E-05	8E-07	0,0076
REACTOME_SIGNALING_BY_FGFR	-0,11	0,003	8E-07	0,0077
MEISSNER_NPC_HCP_WITH_H3_UNMETHYLAT	-0,145	7E-05	8,1E-07	0,0078
V$NMYC_01	-0,168	4E-06	8,2E-07	0,0078
GSE360_T_GONDII_VS_M_TUBERCULOSIS_M/	-0,169	4E-06	8,2E-07	0,0079
GSE3982_MAC_VS_CENT_MEMORY_CD4_TCE	-0,138	2E-04	8,3E-07	0,0079
GSE10463_CD40L_AND_VA347_VS_CD40L_	-0,104	0,004	8,5E-07	0,0081
YAATNRNNNYNATT_UNKNOWN	-0,136	2E-04	8,6E-07	0,0082
chr4q12	-0,209	8E-09	8,6E-07	0,0082
MODULE_49	-0,128	5E-04	8,6E-07	0,0082
CHEBOTAEV_GR_TARGETS_UP	-0,128	5E-04	8,6E-07	0,0082
GSE10239_MEMORY_VS_KLRG1HIGH_EFF_CD	-0,112	0,002	8,7E-07	0,0083
FEMALE_GAMETE_GENERATION	-0,159	1E-05	8,7E-07	0,0083
WAKABAYASHI_ADIPOGENESIS_PPARG_BOUNI	-0,124	7E-04	8,7E-07	0,0083
GSE22886_UNSTIM_VS_IL15_STIM_NKCELL_	-0,098	0,008	8,7E-07	0,0083
HUANG_FOXA2_TARGETS_UP	-0,176	1E-06	8,7E-07	0,0083
TRANSCRIPTION_REPRESSOR_ACTIVITY	-0,118	0,001	8,9E-07	0,0085
GSE13411_NAIVE_BCELL_VS_PLASMA_CELL	-0,184	4E-07	8,9E-07	0,0085
RIZ_ERYTHROID_DIFFERENTIATION_HEMGN	-0,141	1E-04	8,9E-07	0,0085

MODULE_66	-0,125	6E-04	9E-07	0,0085
MYOFIBRIL	-0,153	3E-05	9E-07	0,0086
CCCNNGGGAR_V$OLF1_01	-0,13	4E-04	9E-07	0,0086
CYTOSKELETON	-0,198	5E-08	9E-07	0,0086
MOHANKUMAR_TLX1_TARGETS_DN	-0,119	0,001	9,1E-07	0,0086
IVANOVA_HEMATOPOIESIS_STEM_CELL_AND_	-0,121	1E-03	9,1E-07	0,0087
JI_CARCINOGENESIS_BY_KRAS_AND_STK11_I	-0,195	8E-08	9,1E-07	0,0087
MODULE_100	-0,126	5E-04	9,2E-07	0,0087
GSE2706_R848_VS_R848_AND_LPS_2H_STI	-0,118	0,001	9,2E-07	0,0087
LEIN_LOCALIZED_TO_DISTAL_AND_PROXIMAI	-0,097	0,008	9,2E-07	0,0088
GOLUB_ALL_VS_AML_UP	-0,186	3E-07	9,3E-07	0,0088
NAKAYAMA_SOFT_TISSUE_TUMORS_PCA1_DI	-0,137	2E-04	9,3E-07	0,0088
PID_MYC_PATHWAY	-0,179	8E-07	9,3E-07	0,0089
GTGACGY_V$E4F1_Q6	-0,151	4E-05	9,3E-07	0,0089
REGULATION_OF_PROTEIN_STABILITY	-0,165	6E-06	9,5E-07	0,009
GSE9037_WT_VS_IRAK4_KO_LPS_1H_STIM_	-0,172	2E-06	9,5E-07	0,009
V$CP2_02	-0,14	1E-04	9,5E-07	0,0091
BROWNE_HCMV_INFECTION_48HR_UP	-0,172	2E-06	9,6E-07	0,0091
GSE17974_IL4_AND_ANTI_IL12_VS_UNTREA	-0,105	0,004	9,7E-07	0,0092
WU_APOPTOSIS_BY_CDKN1A_NOT_VIA_TP53	-0,174	2E-06	9,7E-07	0,0092
HOLLEMAN_PREDNISOLONE_RESISTANCE_B_A	-0,196	7E-08	9,8E-07	0,0093
TIGHT_JUNCTION	-0,148	5E-05	9,8E-07	0,0093
VOLTAGE_GATED_CHANNEL_ACTIVITY	-0,088	0,016	9,8E-07	0,0093
IWANAGA_E2F1_TARGETS_NOT_INDUCED_BY	-0,186	3E-07	9,8E-07	0,0093
GSE27786_LSK_VS_NKTCELL_UP	-0,198	5E-08	9,9E-07	0,0094
RTAAACA_V$FREAC2_01	-0,125	6E-04	9,9E-07	0,0094
GCM_RING1	-0,135	2E-04	9,9E-07	0,0094
V$GATA1_02	-0,139	1E-04	9,9E-07	0,0095
NOTCH_SIGNALING_PATHWAY	-0,159	1E-05	1E-06	0,0095
V$CREB_Q4	-0,145	7E-05	1E-06	0,0096
SPIELMAN_LYMPHOBLAST_EUROPEAN_VS_AS	-0,178	9E-07	1E-06	0,0097
TCTATGA,MIR-376A,MIR-376B	-0,16	1E-05	1E-06	0,0097
AFFAR_YY1_TARGETS_UP	-0,144	8E-05	1E-06	0,0097
SHIN_B_CELL_LYMPHOMA_CLUSTER_7	-0,09	0,014	1E-06	0,0097
CHIN_BREAST_CANCER_COPY_NUMBER_DN	-0,235	9E-11	1E-06	0,0098
V$TEF1_Q6	-0,157	2E-05	1E-06	0,0098
POMEROY_MEDULLOBLASTOMA_DESMOPLASI	-0,151	4E-05	1E-06	0,0099
GAANYNYGACNY_UNKNOWN	-0,141	1E-04	1E-06	0,01
GTGGGTGK_UNKNOWN	-0,138	2E-04	1E-06	0,01
V$S8_01	-0,128	5E-04	1,1E-06	0,01
CUI_TCF21_TARGETS_UP	-0,173	2E-06	1,1E-06	0,01
BASAKI_YBX1_TARGETS_UP	-0,186	3E-07	1,1E-06	0,01

V$P300_01	-0,128	5E-04	1,1E-06	0,0101
PID_BMPPATHWAY	-0,139	1E-04	1,1E-06	0,0101
G_PROTEIN_SIGNALING_COUPLED_TO_CAMP_	-0,127	5E-04	1,1E-06	0,0101
VOLTAGE_GATED_CATION_CHANNEL_ACTIVI	-0,092	0,012	1,1E-06	0,0101
REACTOME_GRB2_EVENTS_IN_ERBB2_SIGNAL	-0,185	4E-07	1,1E-06	0,0102
WANG_CLIM2_TARGETS_UP	-0,083	0,023	1,1E-06	0,0102
REACTOME_NGF_SIGNALLING_VIA_TRKA_FRC	-0,118	0,001	1,1E-06	0,0103
TCCATTKW_UNKNOWN	-0,15	4E-05	1,1E-06	0,0104
AMUNDSON_GENOTOXIC_SIGNATURE	-0,172	2E-06	1,1E-06	0,0104
KEGG_COLORECTAL_CANCER	-0,14	1E-04	1,1E-06	0,0105
DETECTION_OF_STIMULUS	-0,09	0,014	1,1E-06	0,0105
V$POU3F2_02	-0,139	1E-04	1,1E-06	0,0106
KEGG_ECM_RECEPTOR_INTERACTION	-0,151	4E-05	1,1E-06	0,0106
GSE29618_LAIV_VS_TIV_FLU_VACCINE_DAY	-0,149	4E-05	1,1E-06	0,0106
TRANSFORMING_GROWTH_FACTOR_BETA_RE	-0,1	0,006	1,1E-06	0,0106
NGUYEN_NOTCH1_TARGETS_UP	-0,1	0,006	1,1E-06	0,0107
V$NRF2_01	-0,199	5E-08	1,1E-06	0,0107
DETECTION_OF_ABIOTIC_STIMULUS	-0,081	0,026	1,1E-06	0,0107
GSE29618_PRE_VS_DAY7_POST_TIV_FLU_V	-0,195	8E-08	1,1E-06	0,0107
HOEGERKORP_CD44_TARGETS_TEMPORAL_U	-0,147	6E-05	1,1E-06	0,0107
REACTOME_RETROGRADE_NEUROTROPHIN_SI	-0,167	5E-06	1,1E-06	0,0107
WTTGKCTG_UNKNOWN	-0,127	5E-04	1,1E-06	0,0109
V$AR_01	-0,127	5E-04	1,1E-06	0,0109
CAMP_MEDIATED_SIGNALING	-0,125	6E-04	1,2E-06	0,0109
REGULATION_OF_DEFENSE_RESPONSE	-0,174	2E-06	1,2E-06	0,0109
YAO_TEMPORAL_RESPONSE_TO_PROGESTERC	-0,16	1E-05	1,2E-06	0,0109
MODULE_11	-0,122	8E-04	1,2E-06	0,0109
REN_ALVEOLAR_RHABDOMYOSARCOMA_UP	-0,142	1E-04	1,2E-06	0,011
V$HEN1_01	-0,134	2E-04	1,2E-06	0,011
GSE2706_LPS_VS_R848_AND_LPS_2H_STIM	-0,127	5E-04	1,2E-06	0,011
V$AP4_Q5	-0,1	0,006	1,2E-06	0,0111
MORF_ITGA2	-0,169	4E-06	1,2E-06	0,0112
GSE29615_CTRL_VS_LAIV_FLU_VACCINE_PE	-0,118	0,001	1,2E-06	0,0113
V$MYOGENIN_Q6	-0,122	9E-04	1,2E-06	0,0114
GSE3982_EOSINOPHIL_VS_NEUTROPHIL_UP	-0,161	1E-05	1,2E-06	0,0114
REACTOME_ADHERENS_JUNCTIONS_INTERAC	-0,209	9E-09	1,2E-06	0,0115
NEUROGENESIS	-0,125	7E-04	1,2E-06	0,0116
MODULE_137	-0,124	7E-04	1,2E-06	0,0116
RPS14_DN.V1_DN	-0,189	2E-07	1,2E-06	0,0118
JUBAN_TARGETS_OF_SPI1_AND_FLI1_DN	-0,194	9E-08	1,3E-06	0,0118
RB_P107_DN.V1_UP	-0,182	6E-07	1,3E-06	0,0118
STRIATED_MUSCLE_DEVELOPMENT	-0,128	5E-04	1,3E-06	0,0119

GSE17721_CTRL_VS_CPG_2H_BMDM_UP	-0,158	1E-05	1,3E-06	0,0119
NEGATIVE_REGULATION_OF_CELLULAR_PROC	-0,156	2E-05	1,3E-06	0,012
V$PR_Q2	-0,13	4E-04	1,3E-06	0,0121
GSE17721_PAM3CSK4_VS_CPG_2H_BMDM_U	-0,126	5E-04	1,3E-06	0,0121
V$SRF_01	-0,131	3E-04	1,3E-06	0,0122
CAIRO_HEPATOBLASTOMA_UP	-0,163	7E-06	1,3E-06	0,0122
GSE15659_CD45RA_NEG_CD4_TCELL_VS_A	-0,152	3E-05	1,3E-06	0,0123
GHANDHI_BYSTANDER_IRRADIATION_DN	-0,156	2E-05	1,3E-06	0,0124
INTERCELLULAR_JUNCTION	-0,145	7E-05	1,3E-06	0,0124
GSE27786_NKTCELL_VS_NEUTROPHIL_DN	-0,078	0,034	1,3E-06	0,0124
V$PTF1BETA_Q6	-0,152	3E-05	1,3E-06	0,0125
GSE2197_IMMUNOSUPPRESSIVE_DNA__VS_U	-0,168	4E-06	1,3E-06	0,0125
SATO_SILENCED_BY_DEACETYLATION_IN_PA	-0,149	4E-05	1,3E-06	0,0126
MICROTUBULE_ASSOCIATED_COMPLEX	-0,178	1E-06	1,3E-06	0,0126
V$EFC_Q6	-0,116	0,001	1,3E-06	0,0126
BCAT_GDS748_DN	-0,163	8E-06	1,3E-06	0,0126
DAVICIONI_RHABDOMYOSARCOMA_PAX_FOXC	-0,173	2E-06	1,3E-06	0,0127
GSE29617_DAY3_VS_DAY7_TIV_FLU_VACCI	-0,168	4E-06	1,4E-06	0,0129
CONTRACTILE_FIBER	-0,145	7E-05	1,4E-06	0,0129
GENERATION_OF_NEURONS	-0,125	6E-04	1,4E-06	0,013
SAFFORD_T_LYMPHOCYTE_ANERGY	-0,161	1E-05	1,4E-06	0,0131
GSE14308_TH2_VS_INDUCED_TREG_DN	-0,134	2E-04	1,4E-06	0,0131
PDZ_DOMAIN_BINDING	-0,069	0,061	1,4E-06	0,0131
GSE22886_NAIVE_BCELL_VS_DC_UP	-0,154	2E-05	1,4E-06	0,0131
BCAT_BILD_ET_AL_UP	-0,125	6E-04	1,4E-06	0,0132
COLINA_TARGETS_OF_4EBP1_AND_4EBP2	-0,182	5E-07	1,4E-06	0,0132
REACTOME_CONVERSION_FROM_APC_C_CDC	-0,171	3E-06	1,4E-06	0,0132
V$NF1_Q6	-0,12	0,001	1,4E-06	0,0133
MODULE_317	-0,153	3E-05	1,4E-06	0,0134
PID_A6B1_A6B4_INTEGRIN_PATHWAY	-0,179	9E-07	1,4E-06	0,0134
PID_REELINPATHWAY	-0,174	2E-06	1,4E-06	0,0134
SINGH_KRAS_DEPENDENCY_SIGNATURE_	-0,186	3E-07	1,4E-06	0,0134
SABATES_COLORECTAL_ADENOMA_DN	-0,114	0,002	1,4E-06	0,0135
V$AP2REP_01	-0,129	4E-04	1,4E-06	0,0136
GOBERT_OLIGODENDROCYTE_DIFFERENTIATIO	-0,138	1E-04	1,5E-06	0,0138
GSE13493_CD4INTCD8POS_VS_CD8POS_THY	-0,082	0,025	1,5E-06	0,0138
GTTGNYNNRGNAAC_UNKNOWN	-0,118	0,001	1,5E-06	0,0139
MRNA_PROCESSING_GO_0006397	-0,202	3E-08	1,5E-06	0,0139
MCMURRAY_TP53_HRAS_COOPERATION_RESP	-0,142	1E-04	1,5E-06	0,014
REACTOME_DOWNREGULATION_OF_TGF_BET	-0,198	5E-08	1,5E-06	0,014
GSE360_L_MAJOR_VS_M_TUBERCULOSIS_MA	-0,176	1E-06	1,5E-06	0,014
V$TATA_01	-0,149	5E-05	1,5E-06	0,0141

TAATTA_V$CHX10_01	-0,136	2E-04	1,5E-06	0,0141
GOBERT_OLIGODENDROCYTE_DIFFERENTIATIO	-0,168	4E-06	1,5E-06	0,0141
YNTTTNNNANGCARM_UNKNOWN	-0,145	7E-05	1,5E-06	0,0141
PID_MYC_ACTIVPATHWAY	-0,187	3E-07	1,5E-06	0,0142
MODULE_98	-0,186	3E-07	1,5E-06	0,0142
MODULE_94	-0,117	0,001	1,5E-06	0,0142
SMIRNOV_RESPONSE_TO_IR_2HR_DN	-0,188	2E-07	1,5E-06	0,0143
V$MYB_Q5_01	-0,147	6E-05	1,5E-06	0,0143
GGATCCG,MIR-127	-0,082	0,025	1,5E-06	0,0143
POTTI_5FU_SENSITIVITY	-0,146	6E-05	1,5E-06	0,0144
BERENJENO_TRANSFORMED_BY_RHOA_FORE\	-0,165	6E-06	1,5E-06	0,0144
MODULE_378	-0,152	3E-05	1,5E-06	0,0144
MODULE_214	-0,175	2E-06	1,5E-06	0,0145
GSE27786_NKCELL_VS_NKTCELL_DN	-0,129	4E-04	1,5E-06	0,0145
MODULE_459	-0,152	3E-05	1,6E-06	0,0147
GSE15930_NAIVE_VS_48H_IN_VITRO_STIM_	-0,122	8E-04	1,6E-06	0,0147
GGGATGC,MIR-324-5P	-0,17	3E-06	1,6E-06	0,0147
GSE13229_IMM_VS_INTMATURE_NKCELL_UP	-0,131	3E-04	1,6E-06	0,0149
FORTSCHEGGER_PHF8_TARGETS_UP	-0,157	2E-05	1,6E-06	0,0149
REGULATION_OF_MITOSIS	-0,181	6E-07	1,6E-06	0,0151
REACTOME_SIGNALING_BY_FGFR_IN_DISEASE	-0,111	0,002	1,6E-06	0,0152
GATED_CHANNEL_ACTIVITY	-0,11	0,003	1,6E-06	0,0152
V$RSRFC4_01	-0,127	5E-04	1,6E-06	0,0152
HOXA9_DN.V1_DN	-0,202	3E-08	1,6E-06	0,0153
SCHLESINGER_H3K27ME3_IN_NORMAL_AND_	-0,126	6E-04	1,6E-06	0,0154
YWATTWNNRGCT_UNKNOWN	-0,133	3E-04	1,7E-06	0,0155
GSE360_L_DONOVANI_VS_T_GONDII_MAC_D	-0,185	3E-07	1,7E-06	0,0156
MEISSNER_NPC_HCP_WITH_H3K4ME2	-0,139	1E-04	1,7E-06	0,0156
NAKAMURA_TUMOR_ZONE_PERIPHERAL_VS_(	-0,213	4E-09	1,7E-06	0,0156
GSE20366_EX_VIVO_VS_DEC205_CONVERSI	-0,127	5E-04	1,7E-06	0,0156
GSE10239_NAIVE_VS_KLRG1HIGH_EFF_CD8_	-0,134	3E-04	1,7E-06	0,0156
chrxq23	-0,186	3E-07	1,7E-06	0,0157
GSE17721_POLYIC_VS_CPG_0.5H_BMDM_UP	-0,116	0,002	1,7E-06	0,0158
V$CREB_Q2	-0,141	1E-04	1,7E-06	0,0158
CELL_PROLIFERATION_GO_0008283	-0,172	2E-06	1,7E-06	0,0159
ENDOCYTIC_VESICLE	-0,164	7E-06	1,7E-06	0,0159
KIM_MYC_AMPLIFICATION_TARGETS_UP	-0,191	1E-07	1,7E-06	0,016
MICROTUBULE_CYTOSKELETON	-0,188	2E-07	1,7E-06	0,0161
MODULE_497	-0,125	6E-04	1,7E-06	0,0161
AMIT_EGF_RESPONSE_120_HELA	-0,162	9E-06	1,7E-06	0,0161
CTCTGGA,MIR-520A,MIR-525	-0,192	1E-07	1,7E-06	0,0162
YAATNANRNNNCAG_UNKNOWN	-0,126	5E-04	1,7E-06	0,0162

ESTABLISHMENT_AND_OR_MAINTENANCE_OF	-0,19	2E-07	1,7E-06	0,0163
CTTTAAR_UNKNOWN	-0,129	4E-04	1,7E-06	0,0163
TCTGGAC,MIR-198	-0,168	4E-06	1,7E-06	0,0163
CATION_CHANNEL_ACTIVITY	-0,124	7E-04	1,7E-06	0,0164
DOANE_BREAST_CANCER_ESR1_DN	-0,172	2E-06	1,7E-06	0,0164
REACTOME_ACTIVATION_OF_KAINATE_RECEF	-0,12	0,001	1,8E-06	0,0165
BURTON_ADIPOGENESIS_1	-0,134	2E-04	1,8E-06	0,0167
MARTORIATI_MDM4_TARGETS_NEUROEPITHEL	-0,156	2E-05	1,8E-06	0,0167
SMID_BREAST_CANCER_BASAL_UP	-0,177	1E-06	1,8E-06	0,0168
GSE22886_TH1_VS_TH2_12H_ACT_UP	-0,147	5E-05	1,8E-06	0,017
HERNANDEZ_MITOTIC_ARREST_BY_DOCETAXE	-0,152	3E-05	1,8E-06	0,0171
GUO_TARGETS_OF_IRS1_AND_IRS2	-0,104	0,005	1,8E-06	0,0171
YAUCH_HEDGEHOG_SIGNALING_PARACRINE_U	-0,089	0,015	1,8E-06	0,0173
GSE3982_BCELL_VS_TH2_DN	-0,198	5E-08	1,9E-06	0,0174
GSE13306_TREG_RA_VS_TCONV_RA_UP	-0,145	7E-05	1,9E-06	0,0174
V$TFIIA_Q6	-0,122	8E-04	1,9E-06	0,0174
WEIGEL_OXIDATIVE_STRESS_BY_HNE_AND_H	-0,193	1E-07	1,9E-06	0,0174
V$LXR_DR4_Q3	-0,161	1E-05	1,9E-06	0,0174
PHESSE_TARGETS_OF_APC_AND_MBD2_UP	-0,086	0,019	1,9E-06	0,0174
GSE27786_BCELL_VS_ERYTHROBLAST_DN	-0,091	0,013	1,9E-06	0,0176
MIKKELSEN_NPC_WITH_LCP_H3K27ME3	-0,101	0,006	1,9E-06	0,0176
HERNANDEZ_MITOTIC_ARREST_BY_DOCETAXE	-0,168	4E-06	1,9E-06	0,0177
GSE27786_NKTCELL_VS_NEUTROPHIL_UP	-0,183	5E-07	1,9E-06	0,0177
V$FOXO4_02	-0,125	6E-04	1,9E-06	0,0177
WEIGEL_OXIDATIVE_STRESS_RESPONSE	-0,191	1E-07	1,9E-06	0,0177
GSE24634_TEFF_VS_TCONV_DAY10_IN_CUL	-0,191	2E-07	1,9E-06	0,0178
TAKADA_GASTRIC_CANCER_COPY_NUMBER_U	-0,19	2E-07	1,9E-06	0,0178
V$AREB6_01	-0,152	3E-05	1,9E-06	0,0178
STEGER_ADIPOGENESIS_DN	-0,137	2E-04	1,9E-06	0,0179
GSE3982_MAC_VS_TH1_DN	-0,184	4E-07	1,9E-06	0,0179
V$STAT5A_01	-0,14	1E-04	1,9E-06	0,018
V$STAT1_01	-0,157	2E-05	1,9E-06	0,018
GSE10325_CD4_TCELL_VS_LUPUS_CD4_TCE	-0,089	0,016	1,9E-06	0,0181
MOTOR_ACTIVITY	-0,149	4E-05	2E-06	0,0182
ACEVEDO_LIVER_CANCER_WITH_H3K27ME3_	-0,166	6E-06	2E-06	0,0183
JECHLINGER_EPITHELIAL_TO_MESENCHYMAL_	-0,138	2E-04	2E-06	0,0184
ION_CHANNEL_ACTIVITY	-0,12	0,001	2E-06	0,0186
BILD_E2F3_ONCOGENIC_SIGNATURE	-0,134	2E-04	2E-06	0,0186
MEISSNER_BRAIN_HCP_WITH_H3K4ME2_AND_	-0,158	1E-05	2E-06	0,0187
NIKOLSKY_BREAST_CANCER_1Q32_AMPLICO	-0,128	5E-04	2E-06	0,019
CHANDRAN_METASTASIS_DN	-0,094	0,01	2E-06	0,019
CTGYNNCTYTAA_UNKNOWN	-0,162	9E-06	2E-06	0,019

GSE1460_DP_VS_CD4_THYMOCYTE_UP	-0,177	1E-06	2E-06	0,0191
BERENJENO_TRANSFORMED_BY_RHOA_REVEF	-0,158	2E-05	2,1E-06	0,0191
ION_TRANSPORT	-0,12	0,001	2,1E-06	0,0193
MODULE_255	-0,147	5E-05	2,1E-06	0,0193
MORF_MAP3K14	-0,114	0,002	2,1E-06	0,0196
BENPORATH_SUZ12_TARGETS	-0,124	7E-04	2,1E-06	0,0196
V$E2F4DP1_01	-0,182	6E-07	2,1E-06	0,0196
V$LMO2COM_01	-0,101	0,006	2,1E-06	0,0197
PUJANA_BRCA1_PCC_NETWORK	-0,191	1E-07	2,1E-06	0,0198
MODULE_397	-0,174	2E-06	2,1E-06	0,0198
V$GATA_C	-0,122	8E-04	2,1E-06	0,0199
GSE15659_NAIVE_CD4_TCELL_VS_ACTIVATI	-0,186	3E-07	2,2E-06	0,02
V$NFY_C	-0,173	2E-06	2,2E-06	0,0201
SIG_IL4RECEPTOR_IN_B_LYPHOCYTES	-0,138	2E-04	2,2E-06	0,0203
GSE1460_DP_THYMOCYTE_VS_NAIVE_CD4_	-0,199	4E-08	2,2E-06	0,0204
RICKMAN_HEAD_AND_NECK_CANCER_D	-0,125	6E-04	2,2E-06	0,0206
CYTOSKELETAL_PROTEIN_BINDING	-0,156	2E-05	2,2E-06	0,0207
GSE11057_NAIVE_VS_EFF_MEMORY_CD4_TC	-0,118	0,001	2,2E-06	0,0208
PRC2_SUZ12_UP.V1_UP	-0,145	7E-05	2,2E-06	0,0208
MODULE_147	-0,185	3E-07	2,2E-06	0,0209
AATGGAG,MIR-136	-0,138	2E-04	2,3E-06	0,0211
V$NFAT_Q6	-0,148	5E-05	2,3E-06	0,0211
BYSTRYKH_HEMATOPOIESIS_STEM_CELL_AND	-0,184	4E-07	2,3E-06	0,0211
G_PROTEIN_SIGNALING_ADENYLATE_CYCLAS	-0,122	8E-04	2,3E-06	0,0214
V$LEF1_Q2	-0,154	2E-05	2,3E-06	0,0214
FIGUEROA_AML_METHYLATION_CLUSTER_6_U	-0,136	2E-04	2,3E-06	0,0214
RAMASWAMY_METASTASIS_UP	-0,177	1E-06	2,3E-06	0,0217
GSE1448_ANTI_VALPHA2_VS_VBETA5_DP_	-0,142	1E-04	2,3E-06	0,0217
GSE32423_CTRL_VS_IL4_MEMORY_CD8_TCE	-0,133	3E-04	2,3E-06	0,0217
PID_RB_1PATHWAY	-0,183	5E-07	2,3E-06	0,0218
LU_TUMOR_ENDOTHELIAL_MARKERS_DN	-0,089	0,015	2,3E-06	0,0218
TGATTTRY_V$GFI1_01	-0,122	8E-04	2,4E-06	0,0219
REACTOME_DOWNSTREAM_SIGNAL_TRANSDU	-0,125	7E-04	2,4E-06	0,0221
V$STAT5B_01	-0,136	2E-04	2,4E-06	0,0222
V$PAX4_03	-0,128	5E-04	2,4E-06	0,0222
REACTOME_VOLTAGE_GATED_POTASSIUM_CI	-0,151	4E-05	2,4E-06	0,0223
KEGG_MTOR_SIGNALING_PATHWAY	-0,149	5E-05	2,4E-06	0,0223
GSE15930_NAIVE_VS_48H_IN_VITRO_STIM_	-0,112	0,002	2,4E-06	0,0223
GSE360_CTRL_VS_T_GONDII_MAC_DN	-0,17	3E-06	2,4E-06	0,0224
chr11p11	-0,166	6E-06	2,4E-06	0,0225
GSE27786_LSK_VS_NKCELL_UP	-0,151	4E-05	2,4E-06	0,0226
GGGTGGRR_V$PAX4_03	-0,132	3E-04	2,4E-06	0,0226

POSITIVE_REGULATION_OF_NUCLEOBASENUC	-0,128	4E-04	2,4E-06	0,0228
NEGATIVE_REGULATION_OF_TRANSCRIPTION_	-0,125	6E-04	2,5E-06	0,0228
GSE7460_FOXP3_MUT_VS_WT_ACT_TCONV_	-0,112	0,002	2,5E-06	0,023
DAWSON_METHYLATED_IN_LYMPHOMA_TCL1	-0,145	7E-05	2,5E-06	0,023
GSE29615_DAY3_VS_DAY7_LAIV_FLU_VAC(	-0,09	0,014	2,5E-06	0,0231
NEGATIVE_REGULATION_OF_BIOLOGICAL_PR(	-0,147	6E-05	2,5E-06	0,0232
GSE15659_NONSUPPRESSIVE_TCELL_VS_ACT	-0,179	9E-07	2,5E-06	0,0232
FOURNIER_ACINAR_DEVELOPMENT_EARLY_DN	-0,194	9E-08	2,5E-06	0,0233
MCBRYAN_PUBERTAL_TGFB1_TARGETS_DN	-0,164	7E-06	2,5E-06	0,0234
RESPONSE_TO_ENDOGENOUS_STIMULUS	-0,15	4E-05	2,5E-06	0,0235
KORKOLA_TERATOMA	-0,16	1E-05	2,6E-06	0,0237
GSE11924_TFH_VS_TH17_CD4_TCELL_DN	-0,191	1E-07	2,6E-06	0,0238
GOLDRATH_EFF_VS_MEMORY_CD8_TCELL_DI	-0,114	0,002	2,6E-06	0,024
HOSHIDA_LIVER_CANCER_SURVIVAL_UP	-0,15	4E-05	2,6E-06	0,0241
LEE_CALORIE_RESTRICTION_NEOCORTEX_DN	-0,155	2E-05	2,6E-06	0,0242
PID_NFAT_3PATHWAY	-0,172	2E-06	2,6E-06	0,0243
V$ELK1_01	-0,122	9E-04	2,6E-06	0,0243
CHANG_POU5F1_TARGETS_DN	-0,141	1E-04	2,6E-06	0,0244
GTCGATC,MIR-369-5P	-0,156	2E-05	2,6E-06	0,0244
SUZUKI_RESPONSE_TO_TSA_AND_DECITABIN	-0,15	4E-05	2,6E-06	0,0244
TRANSMEMBRANE_RECEPTOR_PROTEIN_SERIN	-0,101	0,006	2,6E-06	0,0245
V$E2F_Q6	-0,181	6E-07	2,6E-06	0,0245
V$AMEF2_Q6	-0,131	3E-04	2,7E-06	0,0246
GRAESSMANN_RESPONSE_TO_MC_AND_DOX(	-0,191	1E-07	2,7E-06	0,0248
BENPORATH_CYCLING_GENES	-0,175	1E-06	2,7E-06	0,025
GTCAACC,MIR-380-5P	-0,189	2E-07	2,7E-06	0,0251
REACTOME_NFKB_IS_ACTIVATED_AND_SIGNA	-0,136	2E-04	2,7E-06	0,0253
V$AREB6_02	-0,14	1E-04	2,8E-06	0,0256
FIGUEROA_AML_METHYLATION_CLUSTER_3_l	-0,163	8E-06	2,8E-06	0,0257
PID_PI3KPLCTRKPATHWAY	-0,152	3E-05	2,8E-06	0,0258
CTAGGAA,MIR-384	-0,134	2E-04	2,8E-06	0,0258
REGULATION_OF_ORGANELLE_ORGANIZATION	-0,122	8E-04	2,8E-06	0,0258
GSE14000_4H_VS_16H_LPS_DC_DN	-0,176	1E-06	2,8E-06	0,0258
MODULE_532	-0,149	5E-05	2,8E-06	0,026
MODULE_284	-0,146	7E-05	2,8E-06	0,026
V$TITF1_Q3	-0,13	4E-04	2,8E-06	0,0261
PIGF_UP.V1_DN	-0,143	9E-05	2,8E-06	0,0261
KEGG_PATHWAYS_IN_CANCER	-0,155	2E-05	2,8E-06	0,0262
GSE2706_R848_VS_LPS_2H_STIM_DC_UP	-0,135	2E-04	2,9E-06	0,0264
GSE3982_MAST_CELL_VS_CENT_MEMORY_C	-0,141	1E-04	2,9E-06	0,0265
BENPORATH_EED_TARGETS	-0,13	4E-04	2,9E-06	0,0266
TGACAGNY_V$MEIS1_01	-0,133	3E-04	2,9E-06	0,0266

REACTOME_EGFR_DOWNREGULATION	-0,185	4E-07	2,9E-06	0,0267
GSE15930_STIM_VS_STIM_AND_IFNAB_72H_	-0,133	3E-04	2,9E-06	0,0268
MODULE_356	-0,182	6E-07	2,9E-06	0,0268
CTTTGA_V$LEF1_Q2	-0,137	2E-04	2,9E-06	0,0268
KEGG_VASCULAR_SMOOTH_MUSCLE_CONTRA	-0,107	0,003	2,9E-06	0,0268
DOUGLAS_BMI1_TARGETS_UP	-0,171	3E-06	2,9E-06	0,0271
HOFMANN_MYELODYSPLASTIC_SYNDROM_HIG	-0,135	2E-04	3E-06	0,0273
BHATI_G2M_ARREST_BY_2METHOXYESTRADI(	-0,194	1E-07	3E-06	0,0273
GSE3982_BCELL_VS_CENT_MEMORY_CD4_T(	-0,135	2E-04	3E-06	0,0274
NEURON_DEVELOPMENT	-0,105	0,004	3E-06	0,0278
BONOME_OVARIAN_CANCER_SURVIVAL_SUB(	-0,095	0,01	3E-06	0,0278
S_PHASE	-0,172	2E-06	3E-06	0,0278
GSE1460_CD4_THYMOCYTE_VS_NAIVE_CD4_	-0,158	2E-05	3E-06	0,028
TGFB_UP.V1_UP	-0,142	1E-04	3E-06	0,0281
MORF_ORC1L	-0,148	5E-05	3,1E-06	0,0282
STEIN_ESR1_TARGETS	-0,1	0,006	3,1E-06	0,0283
NOUZOVA_METHYLATED_IN_APL	-0,127	5E-04	3,1E-06	0,0284
V$SP1_Q4_01	-0,129	4E-04	3,1E-06	0,0285
CYTOSKELETON_ORGANIZATION_AND_BIOGEN	-0,155	2E-05	3,1E-06	0,0285
GSE30962_PRIMARY_VS_SECONDARY_CHRON	-0,199	5E-08	3,1E-06	0,0285
PEPPER_CHRONIC_LYMPHOCYTIC_LEUKEMIA_I	-0,088	0,017	3,2E-06	0,0291
GSE17721_POLYIC_VS_GARDIQUIMOD_0.5H_	-0,138	2E-04	3,2E-06	0,0292
HOLLEMAN_VINCRISTINE_RESISTANCE_B_ALL	-0,186	3E-07	3,2E-06	0,0292
TERAO_AOX4_TARGETS_SKIN_DN	-0,133	3E-04	3,2E-06	0,0295
KRAS.600_UP.V1_UP	-0,146	7E-05	3,2E-06	0,0295
CHROMOSOME_ORGANIZATION_AND_BIOGENE	-0,178	1E-06	3,2E-06	0,0296
TTGTTT_V$FOXO4_01	-0,129	4E-04	3,2E-06	0,0297
SCHAEFFER_PROSTATE_DEVELOPMENT_12HR	-0,136	2E-04	3,2E-06	0,0297
RCGCANGCGY_V$NRF1_Q6	-0,158	2E-05	3,3E-06	0,03
GSE17721_CTRL_VS_CPG_24H_BMDM_DN	-0,185	4E-07	3,3E-06	0,0302
POSITIVE_REGULATION_OF_EPITHELIAL_CELL	-0,206	1E-08	3,3E-06	0,0303
TTCNRGNNNNTTC_V$HSF_Q6	-0,151	4E-05	3,3E-06	0,0303
V$HEN1_02	-0,124	7E-04	3,3E-06	0,0304
V$RORA2_01	-0,112	0,002	3,3E-06	0,0305
V$GR_Q6	-0,123	8E-04	3,3E-06	0,0306
CELLULAR_COMPONENT_ASSEMBLY	-0,146	7E-05	3,3E-06	0,0307
BERENJENO_TRANSFORMED_BY_RHOA_UP	-0,184	4E-07	3,3E-06	0,0308
V$GATA6_01	-0,128	5E-04	3,4E-06	0,0311
GSE13229_MATURE_VS_INTMATURE_NKCELL	-0,145	7E-05	3,4E-06	0,0312
V$STAT5A_03	-0,143	9E-05	3,4E-06	0,0312
DING_LUNG_CANCER_MUTATED_SIGNIFICANT	-0,13	4E-04	3,5E-06	0,0319
BENPORATH_ES_2	-0,183	5E-07	3,5E-06	0,032

GSE3982_NEUTROPHIL_VS_EFF_MEMORY_CD	-0,182	6E-07	3,5E-06	0,0321
MEISSNER_BRAIN_HCP_WITH_H3K4ME3_AND_	-0,141	1E-04	3,5E-06	0,0321
V$WHN_B	-0,121	9E-04	3,5E-06	0,0322
V$POU1F1_Q6	-0,154	3E-05	3,5E-06	0,0325
GSE15324_ELF4_KO_VS_WT_ACTIVATED_CI	-0,157	2E-05	3,5E-06	0,0326
MODULE_198	-0,178	1E-06	3,5E-06	0,0326
SCHAEFFER_PROSTATE_DEVELOPMENT_48HR	-0,131	3E-04	3,6E-06	0,0333
BIOCARTA_MPR_PATHWAY	-0,152	3E-05	3,6E-06	0,0335
PARK_HSC_MARKERS	-0,158	2E-05	3,7E-06	0,0337
GSE17974_IL4_AND_ANTI_IL12_VS_UNTREA	-0,16	1E-05	3,7E-06	0,0337
RNA_BINDING	-0,176	1E-06	3,7E-06	0,034
MORF_DCC	-0,145	7E-05	3,7E-06	0,0342
PROTEIN_COMPLEX_BINDING	-0,143	9E-05	3,7E-06	0,0344
GSE15930_NAIVE_VS_72H_IN_VITRO_STIM_	-0,133	3E-04	3,8E-06	0,0346
GSE360_L_MAJOR_VS_T_GONDII_DC_DN	-0,122	9E-04	3,8E-06	0,0347
REACTOME_DEVELOPMENTAL_BIOLOGY	-0,137	2E-04	3,8E-06	0,0349
YAMASHITA_LIVER_CANCER_WITH_EPCAM_UI	-0,173	2E-06	3,8E-06	0,035
ZHENG_GLIOBLASTOMA_PLASTICITY_DN	-0,118	0,001	3,8E-06	0,0351
GSE29618_PRE_VS_DAY7_FLU_VACCINE_MC	-0,147	5E-05	3,8E-06	0,0353
LEIN_MIDBRAIN_MARKERS	-0,126	5E-04	3,9E-06	0,0354
XU_RESPONSE_TO_TRETINOIN_DN	-0,202	3E-08	3,9E-06	0,0355
GSE27786_NKCELL_VS_ERYTHROBLAST_DN	-0,107	0,003	3,9E-06	0,0356
V$CEBPGAMMA_Q6	-0,135	2E-04	3,9E-06	0,0358
RESPONSE_TO_RADIATION	-0,146	7E-05	3,9E-06	0,0359
LIU_CDX2_TARGETS_UP	-0,137	2E-04	3,9E-06	0,036
V$ATF4_Q2	-0,144	8E-05	3,9E-06	0,0363
V$MZF1_01	-0,133	3E-04	4E-06	0,0363
SIG_INSULIN_RECEPTOR_PATHWAY_IN_CARDI	-0,173	2E-06	4E-06	0,0364
TATAAA_V$TATA_01	-0,135	2E-04	4E-06	0,0365
WHITFIELD_CELL_CYCLE_G2_M	-0,177	1E-06	4E-06	0,0368
MODULE_381	-0,097	0,008	4E-06	0,0369
GSE3982_MAST_CELL_VS_TH2_DN	-0,176	1E-06	4E-06	0,0369
KOYAMA_SEMA3B_TARGETS_DN	-0,162	9E-06	4E-06	0,037
SCHRAMM_INHBA_TARGETS_DN	-0,15	4E-05	4E-06	0,037
TTCYRGAA_UNKNOWN	-0,149	4E-05	4E-06	0,0371
MODULE_220	-0,154	2E-05	4,1E-06	0,0372
PERIPHERAL_NERVOUS_SYSTEM_DEVELOPMEI	-0,164	7E-06	4,1E-06	0,0372
V$FREAC2_01	-0,146	6E-05	4,1E-06	0,0375
MURAKAMI_UV_RESPONSE_6HR_UP	-0,161	1E-05	4,1E-06	0,0375
GSE13485_DAY1_VS_DAY21_YF17D_VACCII	-0,111	0,002	4,1E-06	0,0376
V$NRF2_Q4	-0,151	4E-05	4,1E-06	0,0376
GSE15659_NAIVE_CD4_TCELL_VS_ACTIVATI	-0,158	2E-05	4,1E-06	0,0376

MOROSETTI_FACIOSCAPULOHUMERAL_MUSCU	-0,152	3E-05	4,1E-06	0,0377
MORF_IL9	-0,108	0,003	4,2E-06	0,038
GSE27786_NKTCELL_VS_MONO_MAC_DN	-0,17	3E-06	4,2E-06	0,0381
FUJIWARA_PARK2_IN_LIVER_CANCER_DN	-0,18	8E-07	4,2E-06	0,0381
REACTOME_SIGNALING_BY_TGF_BETA_RECEP	-0,195	8E-08	4,2E-06	0,0382
CHICAS_RB1_TARGETS_SENESCENT	-0,19	2E-07	4,2E-06	0,0384
ZWANG_DOWN_BY_2ND_EGF_PULSE	-0,121	1E-03	4,2E-06	0,0385
GAUSSMANN_MLL_AF4_FUSION_TARGETS_D_	-0,147	5E-05	4,2E-06	0,0386
GSE3982_DC_VS_TH2_DN	-0,178	1E-06	4,3E-06	0,0389
V$MYB_Q3	-0,121	9E-04	4,3E-06	0,0389
REACTOME_P75NTR_SIGNALS_VIA_NFKB	-0,161	9E-06	4,3E-06	0,039
GSE3982_MAC_VS_BASOPHIL_DN	-0,135	2E-04	4,3E-06	0,0392
GTGTGAG,MIR-342	-0,191	1E-07	4,3E-06	0,0393
KEGG_TGF_BETA_SIGNALING_PATHWAY	-0,138	2E-04	4,3E-06	0,0393
CRX_NRL_DN.V1_UP	-0,098	0,007	4,3E-06	0,0393
GSE9006_1MONTH_VS_4MONTH_POST_TYPE	-0,13	4E-04	4,3E-06	0,0394
V$POU6F1_01	-0,115	0,002	4,3E-06	0,0394
NEURON_DIFFERENTIATION	-0,114	0,002	4,3E-06	0,0396
GSE3982_DC_VS_EFF_MEMORY_CD4_TCELL_	-0,124	7E-04	4,4E-06	0,0398
TENEDINI_MEGAKARYOCYTE_MARKERS	-0,145	7E-05	4,4E-06	0,0403
TGGAAA_V$NFAT_Q4_01	-0,128	4E-04	4,4E-06	0,0403
V$SRF_Q6	-0,129	4E-04	4,4E-06	0,0403
MODULE_18	-0,197	6E-08	4,4E-06	0,0403
GSE14308_TH17_VS_INDUCED_TREG_DN	-0,165	6E-06	4,4E-06	0,0403
GSE22886_NAIVE_TCELL_VS_NKCELL_UP	-0,151	4E-05	4,4E-06	0,0405
MAHADEVAN_GIST_MORPHOLOGICAL_SWITCH	-0,157	2E-05	4,5E-06	0,0407
MODULE_206	-0,153	3E-05	4,5E-06	0,0409
GSE14769_UNSTIM_VS_360MIN_LPS_BMDM_	-0,186	3E-07	4,5E-06	0,0409
HORMONE_ACTIVITY	-0,111	0,002	4,5E-06	0,0411
GSE13411_IGM_MEMORY_BCELL_VS_PLASMA	-0,183	5E-07	4,5E-06	0,0411
GSE9988_LPS_VS_LOW_LPS_MONOCYTE_DN	-0,136	2E-04	4,5E-06	0,0412
V$HOXA4_Q2	-0,145	7E-05	4,5E-06	0,0413
M_PHASE	-0,167	5E-06	4,5E-06	0,0413
GSE26928_EFF_MEM_VS_CENTR_MEM_CD4_	-0,156	2E-05	4,5E-06	0,0413
V$NFY_Q6_01	-0,19	2E-07	4,5E-06	0,0414
PID_SYNDECAN_4_PATHWAY	-0,173	2E-06	4,5E-06	0,0415
GSE14769_UNSTIM_VS_240MIN_LPS_BMDM_	-0,178	1E-06	4,6E-06	0,0418
MCCLUNG_CREB1_TARGETS_DN	-0,151	4E-05	4,6E-06	0,0418
GSE24026_PD1_LIGATION_VS_CTRL_IN_ACT	-0,191	1E-07	4,6E-06	0,0421
GSE13485_DAY7_VS_DAY21_YF17D_VACCI	-0,138	2E-04	4,6E-06	0,0421
SEXUAL_REPRODUCTION	-0,105	0,004	4,6E-06	0,0423
GSE18791_UNSTIM_VS_NEWCATSLE_VIRUS_	-0,164	7E-06	4,6E-06	0,0423

TGCACGA,MIR-517A,MIR-517C	-0,142	1E-04	4,6E-06	0,0423
MEL18_DN.V1_DN	-0,121	1E-03	4,7E-06	0,0427
MORF_RFC5	-0,189	2E-07	4,7E-06	0,0429
V$NKX25_02	-0,138	2E-04	4,7E-06	0,043
BIOCARTA_SHH_PATHWAY	-0,127	5E-04	4,7E-06	0,0431
MUELLER_PLURINET	-0,172	2E-06	4,7E-06	0,0431
V$SP3_Q3	-0,111	0,002	4,7E-06	0,0431
chr7p14	-0,141	1E-04	4,7E-06	0,0431
MODULE_163	-0,128	5E-04	4,7E-06	0,0431
MORI_EMU_MYC_LYMPHOMA_BY_ONSET_TIMI	-0,169	4E-06	4,8E-06	0,0433
KONDO_COLON_CANCER_HCP_WITH_H3K27M	-0,179	9E-07	4,8E-06	0,0434
CELL_JUNCTION	-0,128	5E-04	4,8E-06	0,0434
GSE20366_CD103_POS_VS_NEG_TREG_KLR(	-0,145	7E-05	4,8E-06	0,0436
V$HSF_Q6	-0,142	1E-04	4,8E-06	0,0439
GAUSSMANN_MLL_AF4_FUSION_TARGETS_C_	-0,159	1E-05	4,8E-06	0,0441
GSE28237_FOLLICULAR_VS_EARLY_GC_BCEL	-0,126	6E-04	4,9E-06	0,0442
STAMBOLSKY_TARGETS_OF_MUTATED_TP53	-0,167	4E-06	4,9E-06	0,0443
REACTOME_DOWNSTREAM_SIGNALING_OF_A(	-0,091	0,013	4,9E-06	0,0443
WCTCNATGGY_UNKNOWN	-0,076	0,038	4,9E-06	0,0444
NIELSEN_LEIOMYOSARCOMA_CNN1_DN	-0,165	6E-06	4,9E-06	0,0446
GCM_MAPK10	-0,151	3E-05	4,9E-06	0,0448
NRL_DN.V1_UP	-0,119	0,001	4,9E-06	0,0448
KOHOUTEK_CCNT2_TARGETS	-0,136	2E-04	4,9E-06	0,0449
GSE36476_YOUNG_VS_OLD_DONOR_MEMOR'	-0,112	0,002	5E-06	0,0453
HEIDENBLAD_AMPLICON_12P11_12_DN	-0,14	1E-04	5E-06	0,0453
V$PAX2_02	-0,13	4E-04	5E-06	0,0454
V$OCT1_05	-0,138	2E-04	5E-06	0,0454
S_PHASE_OF_MITOTIC_CELL_CYCLE	-0,157	2E-05	5,1E-06	0,0461
NUYTTEN_EZH2_TARGETS_DN	-0,17	3E-06	5,1E-06	0,0463
RADAEVA_RESPONSE_TO_IFNA1_DN	-0,147	6E-05	5,1E-06	0,0463
BILANGES_SERUM_SENSITIVE_VIA_TSC1	-0,107	0,004	5,1E-06	0,0466
PID_ILK_PATHWAY	-0,19	2E-07	5,2E-06	0,0471
HAN_SATB1_TARGETS_UP	-0,144	8E-05	5,2E-06	0,0472
TOMIDA_METASTASIS_UP	-0,197	6E-08	5,2E-06	0,0472
V$OCT1_Q6	-0,133	3E-04	5,2E-06	0,0473
CCAWWNAAGG_V$SRF_Q4	-0,125	6E-04	5,2E-06	0,0477
WONG_ENDMETRIUM_CANCER_DN	-0,095	0,009	5,3E-06	0,0477
CAIRO_PML_TARGETS_BOUND_BY_MYC_UP	-0,179	8E-07	5,3E-06	0,0478
INGRAM_SHH_TARGETS_DN	-0,143	9E-05	5,3E-06	0,0478
VANHARANTA_UTERINE_FIBROID_UP	-0,162	8E-06	5,3E-06	0,048
WU_ALZHEIMER_DISEASE_UP	-0,164	7E-06	5,3E-06	0,0483
V$NKX3A_01	-0,132	3E-04	5,3E-06	0,0485

TRANSCRIPTION_COREPRESSOR_ACTIVITY	-0,132	3E-04	5,4E-06	0,0486
NEURITE_DEVELOPMENT	-0,114	0,002	5,4E-06	0,0487
PID_HEDGEHOG_GLIPATHWAY	-0,113	0,002	5,4E-06	0,0491
V$LXR_Q3	-0,152	3E-05	5,4E-06	0,0492
V$CART1_01	-0,148	5E-05	5,4E-06	0,0492
RNCTGNYNRNCTGNY_UNKNOWN	-0,126	6E-04	5,4E-06	0,0494
FIGUEROA_AML_METHYLATION_CLUSTER_7_I	-0,103	0,005	5,5E-06	0,0497
NAKAYAMA_FGF2_TARGETS	-0,161	9E-06	5,5E-06	0,0497
EBAUER_TARGETS_OF_PAX3_FOXO1_FUSION_	-0,154	2E-05	5,5E-06	0,0497
V$FOXJ2_01	-0,133	3E-04	5,5E-06	0,05
SIMBULAN_UV_RESPONSE_IMMORTALIZED_DN	-0,174	2E-06	5,5E-06	0,0502
MYLLYKANGAS_AMPLIFICATION_HOT_SPOT_1	-0,16	1E-05	5,5E-06	0,0502
GSE3982_EOSINOPHIL_VS_TH2_DN	-0,188	2E-07	5,6E-06	0,0504
REGULATION_OF_NEUROGENESIS	-0,103	0,005	5,6E-06	0,0505
LEE_CALORIE_RESTRICTION_NEOCORTEX_UP	-0,161	9E-06	5,6E-06	0,0506
RYTGCNNRGNAAC_V$MIF1_01	-0,078	0,033	5,6E-06	0,0507
chr9q12	-0,144	8E-05	5,6E-06	0,0508
MULTICELLULAR_ORGANISMAL_DEVELOPMENT	-0,146	7E-05	5,6E-06	0,051
GSE29618_PRE_VS_DAY7_FLU_VACCINE_PD	-0,135	2E-04	5,6E-06	0,0511
BIOCARTA_CTCF_PATHWAY	-0,137	2E-04	5,6E-06	0,0511
KYNG_ENVIRONMENTAL_STRESS_RESPONSE_	-0,15	4E-05	5,7E-06	0,0512
SHETH_LIVER_CANCER_VS_TXNIP_LOSS_PAM	-0,164	7E-06	5,7E-06	0,0515
BRUINS_UVC_RESPONSE_LATE	-0,158	1E-05	5,7E-06	0,0518
BROWNE_HCMV_INFECTION_1HR_DN	-0,15	4E-05	5,8E-06	0,0522
TSENG_IRS1_TARGETS_UP	-0,183	5E-07	5,8E-06	0,0524
V$E47_01	-0,15	4E-05	5,8E-06	0,0525
GSE25087_TREG_VS_TCONV_FETUS_DN	-0,13	4E-04	5,8E-06	0,0525
KRAS.300_UP.V1_UP	-0,149	4E-05	5,8E-06	0,0527
V$E4BP4_01	-0,129	4E-04	5,8E-06	0,0529
AXONOGENESIS	-0,104	0,005	5,9E-06	0,053
V$HNF3ALPHA_Q6	-0,116	0,002	5,9E-06	0,0534
GSE3982_NEUTROPHIL_VS_NKCELL_DN	-0,164	7E-06	5,9E-06	0,0535
QI_PLASMACYTOMA_DN	-0,162	9E-06	5,9E-06	0,0536
V$CDC5_01	-0,12	0,001	5,9E-06	0,0538
GSE15750_WT_VS_TRAF6KO_DAY6_EFF_CD	-0,151	4E-05	6E-06	0,0538
V$AREB6_04	-0,143	9E-05	6E-06	0,0539
GSE15930_NAIVE_VS_48H_IN_VITRO_STIM_	-0,094	0,011	6E-06	0,0539
TONKS_TARGETS_OF_RUNX1_RUNX1T1_FUSI	-0,118	0,001	6E-06	0,0544
MEMBRANE_ENCLOSED_LUMEN	-0,186	3E-07	6,1E-06	0,0548
ORGANELLE_LUMEN	-0,186	3E-07	6,1E-06	0,0548
GSE17721_CPG_VS_GARDIQUIMOD_4H_BMDM	-0,124	7E-04	6,1E-06	0,0554
chr4p15	-0,16	1E-05	6,2E-06	0,056

AAAYWAACM_V$HFH4_01	-0,113	0,002	6,2E-06	0,056
TGCTGAY_UNKNOWN	-0,134	2E-04	6,2E-06	0,0561
CONRAD_STEM_CELL	-0,176	1E-06	6,2E-06	0,0564
GSE22886_NAIVE_CD8_TCELL_VS_NKCELL_	-0,143	9E-05	6,3E-06	0,0566
DUTERTRE_ESTRADIOL_RESPONSE_24HR_UP	-0,149	5E-05	6,3E-06	0,0567
BOYAULT_LIVER_CANCER_SUBCLASS_G56_U	-0,083	0,024	6,3E-06	0,0568
REGIONALIZATION	-0,107	0,003	6,3E-06	0,0571
LE_EGR2_TARGETS_UP	-0,166	5E-06	6,3E-06	0,0571
V$PITX2_Q2	-0,147	5E-05	6,3E-06	0,0571
REACTOME_NCAM1_INTERACTIONS	-0,144	8E-05	6,4E-06	0,0575
GHO_ATF5_TARGETS_DN	-0,187	3E-07	6,4E-06	0,0575
GSE2706_LPS_VS_R848_AND_LPS_2H_STIM	-0,122	8E-04	6,4E-06	0,0577
GSE27786_BCELL_VS_NKCELL_UP	-0,146	6E-05	6,4E-06	0,0577
GSE15930_STIM_VS_STIM_AND_IFNAB_24H_	-0,131	4E-04	6,5E-06	0,0584
GSE3982_MAC_VS_BCELL_DN	-0,127	5E-04	6,5E-06	0,0585
AAAGGAT,MIR-501	-0,159	1E-05	6,5E-06	0,0586
GROWTH	-0,096	0,009	6,6E-06	0,0591
GSE29618_PRE_VS_DAY7_FLU_VACCINE_BC	-0,166	5E-06	6,6E-06	0,0594
MOREAUX_MULTIPLE_MYELOMA_BY_TACI_UP	-0,122	9E-04	6,6E-06	0,0594
GSE17721_POLYIC_VS_CPG_1H_BMDM_UP	-0,143	9E-05	6,6E-06	0,0595
GCM_SUPT4H1	-0,131	3E-04	6,6E-06	0,0595
REACTOME_MUSCLE_CONTRACTION	-0,142	1E-04	6,6E-06	0,0597
V$MAZR_01	-0,101	0,006	6,6E-06	0,0598
CERVERA_SDHB_TARGETS_1_UP	-0,114	0,002	6,6E-06	0,0599
GSE15659_CD45RA_NEG_CD4_TCELL_VS_N	-0,119	0,001	6,6E-06	0,0599
KEGG_LONG_TERM_POTENTIATION	-0,149	5E-05	6,7E-06	0,0603
MODULE_112	-0,126	6E-04	6,7E-06	0,0603
TRAYNOR_RETT_SYNDROM_DN	-0,107	0,003	6,8E-06	0,0609
KYNG_ENVIRONMENTAL_STRESS_RESPONSE_	-0,138	2E-04	6,8E-06	0,0613
V$AP1_Q2_01	-0,159	1E-05	6,8E-06	0,0614
RAO_BOUND_BY_SALL4_ISOFORM_A	-0,124	7E-04	6,8E-06	0,0616
JIANG_VHL_TARGETS	-0,18	7E-07	6,8E-06	0,0616
BMI1_DN_MEL18_DN.V1_DN	-0,116	0,002	6,9E-06	0,0617
chr9q13	-0,144	8E-05	6,9E-06	0,0617
DANG_BOUND_BY_MYC	-0,187	3E-07	6,9E-06	0,0619
KAYO_AGING_MUSCLE_UP	-0,148	5E-05	6,9E-06	0,062
PID_ERBB1_INTERNALIZATION_PATHWAY	-0,168	4E-06	6,9E-06	0,0625
MISHRA_CARCINOMA_ASSOCIATED_FIBROBLA	-0,128	4E-04	7E-06	0,0627
GCCNNNWTAAR_UNKNOWN	-0,137	2E-04	7E-06	0,0629
PID_RHOA_PATHWAY	-0,161	1E-05	7E-06	0,0629
BENPORATH_ES_WITH_H3K27ME3	-0,112	0,002	7E-06	0,0633
V$MMEF2_Q6	-0,126	6E-04	7,1E-06	0,0634

AGGAAGC,MIR-516-3P	-0,152	3E-05	7,1E-06	0,0636
RIGGI_EWING_SARCOMA_PROGENITOR_UP	-0,122	9E-04	7,1E-06	0,0636
BENPORATH_ES_1	-0,19	2E-07	7,1E-06	0,0637
MODULE_124	-0,165	6E-06	7,1E-06	0,0637
PURBEY_TARGETS_OF_CTBP1_AND_SATB1_[	-0,165	6E-06	7,1E-06	0,064
WONG_ADULT_TISSUE_STEM_MODULE	-0,117	0,001	7,1E-06	0,0642
SYSTEM_DEVELOPMENT	-0,147	5E-05	7,1E-06	0,0642
KEGG_CELL_CYCLE	-0,166	5E-06	7,2E-06	0,0643
STRUCTURAL_CONSTITUENT_OF_CYTOSKELET	-0,15	4E-05	7,2E-06	0,0645
TESAR_ALK_AND_JAK_TARGETS_MOUSE_ES_	-0,152	3E-05	7,2E-06	0,0646
GSE10325_BCELL_VS_LUPUS_BCELL_UP	-0,072	0,048	7,2E-06	0,0648
CELLULAR_PROTEIN_METABOLIC_PROCESS	-0,159	1E-05	7,2E-06	0,0649
VALK_AML_CLUSTER_12	-0,152	3E-05	7,3E-06	0,0655
CHESLER_BRAIN_HIGHEST_EXPRESSION	-0,121	1E-03	7,3E-06	0,0655
NUCLEOPLASM	-0,18	8E-07	7,3E-06	0,0657
GSE22886_NAIVE_CD4_TCELL_VS_48H_ACT	-0,066	0,072	7,4E-06	0,066
BOYLAN_MULTIPLE_MYELOMA_C_UP	-0,164	7E-06	7,4E-06	0,0661
GSE9650_EFFECTOR_VS_MEMORY_CD8_TCEL	-0,113	0,002	7,4E-06	0,0661
GSE14308_TH2_VS_NAIVE_CD4_TCELL_UP	-0,181	6E-07	7,4E-06	0,0661
GSE339_CD4POS_VS_CD4CD8DN_DC_IN_CUI	-0,142	1E-04	7,4E-06	0,0663
REACTOME_SMOOTH_MUSCLE_CONTRACTION	-0,152	3E-05	7,4E-06	0,0663
V$MEF2_03	-0,119	0,001	7,5E-06	0,0671
EBAUER_MYOGENIC_TARGETS_OF_PAX3_FOX	-0,115	0,002	7,5E-06	0,0672
V$HAND1E47_01	-0,139	1E-04	7,5E-06	0,0673
DACOSTA_ERCC3_ALLELE_XPCS_VS_TTD_UI	-0,176	1E-06	7,6E-06	0,0678
MODULE_215	-0,172	2E-06	7,6E-06	0,068
INACTIVATION_OF_MAPK_ACTIVITY	-0,169	4E-06	7,6E-06	0,0685
TGACCTY_V$ERR1_Q2	-0,129	4E-04	7,7E-06	0,0688
GSE3982_MAC_VS_EFF_MEMORY_CD4_TCEL	-0,153	3E-05	7,7E-06	0,0692
MODULE_433	-0,153	3E-05	7,7E-06	0,0692
GSE15659_NAIVE_CD4_TCELL_VS_RESTING_	-0,157	2E-05	7,7E-06	0,0693
YAUCH_HEDGEHOG_SIGNALING_PARACRINE_[	-0,126	6E-04	7,7E-06	0,0693
V$AR_03	-0,082	0,026	7,7E-06	0,0694
REACTOME_NEGATIVE_REGULATION_OF_THE_	-0,143	9E-05	7,8E-06	0,0696
PEDERSEN_METASTASIS_BY_ERBB2_ISOFORM	-0,106	0,004	7,8E-06	0,0698
GSE13493_DP_VS_CD8POS_THYMOCYTE_UP	-0,128	4E-04	7,8E-06	0,0698
GSE3982_NEUTROPHIL_VS_TH1_DN	-0,185	4E-07	7,8E-06	0,0699
CORDENONSI_YAP_CONSERVED_SIGNATURE	-0,176	1E-06	7,8E-06	0,0699
GSE15750_WT_VS_TRAF6KO_DAY10_EFF_C	-0,117	0,001	7,8E-06	0,0702
GSE10239_MEMORY_VS_KLRG1INT_EFF_CD8	-0,108	0,003	7,8E-06	0,0702
REGULATION_OF_ANATOMICAL_STRUCTURE_	-0,113	0,002	7,8E-06	0,0702
INAMURA_LUNG_CANCER_SCC_DN	-0,104	0,005	7,9E-06	0,0705

CCANNAGRKGGC_UNKNOWN	-0,09	0,014	7,9E-06	0,0707
V$PBX1_01	-0,122	8E-04	7,9E-06	0,0711
SENESE_HDAC3_TARGETS_DN	-0,17	3E-06	8E-06	0,0712
MODULE_195	-0,172	2E-06	8E-06	0,0712
MIKKELSEN_MEF_HCP_WITH_H3_UNMETHYLA	-0,143	9E-05	8E-06	0,0712
PROTEIN_TYROSINE_KINASE_ACTIVITY	-0,137	2E-04	8E-06	0,0712
MODULE_95	-0,125	6E-04	8E-06	0,0716
LABBE_TGFB1_TARGETS_UP	-0,165	6E-06	8E-06	0,0719
BENPORATH_NOS_TARGETS	-0,166	5E-06	8,1E-06	0,0722
GSE7460_CTRL_VS_TGFB_TREATED_ACT_TF	-0,117	0,001	8,1E-06	0,0724
HANSON_HRAS_SIGNALING_VIA_NFKB	-0,16	1E-05	8,1E-06	0,0724
V$P53_DECAMER_Q2	-0,123	7E-04	8,2E-06	0,0731
DNA_MODIFICATION	-0,176	1E-06	8,2E-06	0,0733
POSITIVE_REGULATION_OF_CELLULAR_COMP(	-0,095	0,01	8,2E-06	0,0737
GSE15930_STIM_VS_STIM_AND_IL-12_72H_	-0,153	3E-05	8,3E-06	0,0742
SMOOTH_MUSCLE_CONTRACTION_GO_00069	-0,162	9E-06	8,4E-06	0,075
GSE22886_IGA_VS_IGM_MEMORY_BCELL_UP	-0,152	3E-05	8,4E-06	0,075
ESC_V6.5_UP_LATE.V1_DN	-0,156	2E-05	8,4E-06	0,0752
V$GR_Q6_01	-0,124	7E-04	8,4E-06	0,0754
TGGNNNNNNKCCAR_UNKNOWN	-0,107	0,003	8,6E-06	0,0767
GNF2_MAPT	-0,107	0,004	8,6E-06	0,0771
NUCLEAR_BODY	-0,137	2E-04	8,6E-06	0,0772
MEISSNER_ES_ICP_WITH_H3K4ME3_AND_H3I	-0,108	0,003	8,7E-06	0,0773
SCHLINGEMANN_SKIN_CARCINOGENESIS_TPA_	-0,105	0,004	8,7E-06	0,0777
MEK_UP.V1_DN	-0,091	0,012	8,7E-06	0,0779
MIKKELSEN_ES_ICP_WITH_H3K4ME3	-0,089	0,015	8,7E-06	0,0779
GENTILE_RESPONSE_CLUSTER_D3	-0,191	1E-07	8,7E-06	0,0779
KEGG_NEUROACTIVE_LIGAND_RECEPTOR_INT	-0,117	0,001	8,7E-06	0,0779
GSE24634_IL4_VS_CTRL_TREATED_NAIVE_C	-0,151	4E-05	8,8E-06	0,0786
KEGG_PURINE_METABOLISM	-0,122	8E-04	8,8E-06	0,0786
RIZKI_TUMOR_INVASIVENESS_3D_UP	-0,109	0,003	8,8E-06	0,0787
CELL_DEVELOPMENT	-0,141	1E-04	8,8E-06	0,0789
BIOCARTA_TEL_PATHWAY	-0,152	3E-05	8,9E-06	0,0791
GCM_PTPRU	-0,166	5E-06	8,9E-06	0,0792
V$E2F_Q4	-0,174	2E-06	8,9E-06	0,0793
GSE3982_NKCELL_VS_TH2_UP	-0,134	2E-04	8,9E-06	0,0794
V$SRF_Q5_01	-0,116	0,002	9E-06	0,0798
TTTNNANAGCYR_UNKNOWN	-0,202	3E-08	9E-06	0,0805
SESTO_RESPONSE_TO_UV_C8	-0,167	4E-06	9E-06	0,0806
JEPSEN_SMRT_TARGETS	-0,144	8E-05	9,1E-06	0,0808
TGGTGCT,MIR-29A,MIR-29B,MIR-29C	-0,145	7E-05	9,1E-06	0,0811
V$BRN2_01	-0,119	0,001	9,2E-06	0,0817

ISSAEVA_MLL2_TARGETS	-0,163	8E-06	9,2E-06	0,0818
RAAGNYNNCTTY_UNKNOWN	-0,138	2E-04	9,2E-06	0,0819
V$TAL1BETAITF2_01	-0,118	0,001	9,2E-06	0,0823
GSE1460_NAIVE_CD4_TCELL_ADULT_BLOOD	-0,189	2E-07	9,2E-06	0,0824
CASORELLI_APL_SECONDARY_VS_DE_NOVO_	-0,16	1E-05	9,3E-06	0,0826
REACTOME_AXON_GUIDANCE	-0,145	7E-05	9,4E-06	0,0837
ENGELMANN_CANCER_PROGENITORS_DN	-0,082	0,025	9,5E-06	0,0843
PLASARI_TGFB1_TARGETS_10HR_UP	-0,149	4E-05	9,5E-06	0,0844
TTCYNRGAA_V$STAT5B_01	-0,133	3E-04	9,5E-06	0,0845
SUZUKI_RESPONSE_TO_TSA_AND_DECITABIN	-0,125	6E-04	9,5E-06	0,0846
GSE13485_DAY3_VS_DAY21_YF17D_VACCII	-0,106	0,004	9,6E-06	0,0851
REGULATION_OF_ACTION_POTENTIAL	-0,158	1E-05	9,6E-06	0,0858
GSE7460_CTRL_VS_FOXP3_OVEREXPR_TCON	-0,13	4E-04	9,7E-06	0,0862
GSE6269_STREP_AUREUS_VS_STREP_PNEUM	-0,129	4E-04	9,7E-06	0,0864
STAMBOLSKY_BOUND_BY_MUTATED_TP53	-0,131	4E-04	9,8E-06	0,0868
GSE3982_CTRL_VS_LPS_1H_NEUTROPHIL_DI	-0,117	0,001	9,8E-06	0,0868
G1_PHASE	-0,161	1E-05	9,8E-06	0,0868
GSE360_L_MAJOR_VS_T_GONDII_DC_UP	-0,118	0,001	9,8E-06	0,087
G_PROTEIN_SIGNALING_ADENYLATE_CYCLAS	-0,119	0,001	9,9E-06	0,0876
V$CACCCBINDINGFACTOR_Q6	-0,13	4E-04	9,9E-06	0,0876
V$CDPCR1_01	-0,111	0,002	9,9E-06	0,0879
GSE17974_0H_VS_0.5H_IN_VITRO_ACT_CD4	-0,148	5E-05	9,9E-06	0,0882
GSE24142_ADULT_VS_FETAL_EARLY_THYMI	-0,112	0,002	1E-05	0,0885
CELLULAR_MACROMOLECULE_METABOLIC_PR	-0,154	2E-05	1E-05	0,0887
RNA_PROCESSING	-0,183	5E-07	1E-05	0,0894
HOMOPHILIC_CELL_ADHESION	-0,134	2E-04	1E-05	0,0895
SMID_BREAST_CANCER_LUMINAL_B_DN	-0,168	4E-06	1E-05	0,0897
V$E2F1DP1RB_01	-0,169	3E-06	1E-05	0,0897
IL21_UP.V1_UP	-0,129	4E-04	1E-05	0,0897
GSE22886_TH1_VS_TH2_48H_ACT_UP	-0,152	3E-05	1E-05	0,09
GSE2706_UNSTIM_VS_2H_LPS_DC_UP	-0,086	0,018	1E-05	0,09
GSE17721_PAM3CSK4_VS_GADIQUIMOD_0.5	-0,141	1E-04	1E-05	0,0905
GSE1460_DP_THYMOCYTE_VS_NAIVE_CD4_	-0,114	0,002	1E-05	0,0907
CELL_SURFACE	-0,102	0,005	1E-05	0,0909
TGACATY_UNKNOWN	-0,113	0,002	1E-05	0,0911
G1_PHASE_OF_MITOTIC_CELL_CYCLE	-0,16	1E-05	1E-05	0,0918
VALK_AML_CLUSTER_6	-0,131	3E-04	1E-05	0,0922
NADLER_HYPERGLYCEMIA_AT_OBESITY	-0,167	5E-06	1E-05	0,0924
V$PAX4_01	-0,097	0,008	1E-05	0,0928
SUBSTRATE_SPECIFIC_CHANNEL_ACTIVITY	-0,114	0,002	1,1E-05	0,0932
BENPORATH_PRC2_TARGETS	-0,108	0,003	1,1E-05	0,0932
RASHI_RESPONSE_TO_IONIZING_RADIATION_5	-0,11	0,003	1,1E-05	0,0945

GSE15324_ELF4_KO_VS_WT_NAIVE_CD8_T(	-0,125	6E-04	1,1E-05	0,0946
V$AHRARNT_01	-0,144	8E-05	1,1E-05	0,0949
MODULE_12	-0,129	4E-04	1,1E-05	0,095
V$MZF1_02	-0,138	2E-04	1,1E-05	0,0951
SMTTTTGT_UNKNOWN	-0,145	7E-05	1,1E-05	0,0952
VART_KSHV_INFECTION_ANGIOGENIC_MARKE	-0,132	3E-04	1,1E-05	0,096
RECEPTOR_BINDING	-0,138	2E-04	1,1E-05	0,097
DCA_UP.V1_UP	-0,124	7E-04	1,1E-05	0,0972
GSE30083_SP1_VS_SP4_THYMOCYTE_UP	-0,153	3E-05	1,1E-05	0,0974
BORLAK_LIVER_CANCER_EGF_UP	-0,132	3E-04	1,1E-05	0,0975
BIOCARTA_NGF_PATHWAY	-0,137	2E-04	1,1E-05	0,0983
FUKUSHIMA_TNFSF11_TARGETS	-0,143	9E-05	1,1E-05	0,0991
PHOSPHORYLATION	-0,146	7E-05	1,1E-05	0,0992
MACROMOLECULAR_COMPLEX_ASSEMBLY	-0,139	1E-04	1,1E-05	0,0998
CELL_DIVISION	-0,179	9E-07	1,1E-05	0,0999
MODULE_176	-0,123	8E-04	1,1E-05	0,1002
RGAANNTTC_V$HSF1_01	-0,127	5E-04	1,1E-05	0,1004
ADENYLATE_CYCLASE_ACTIVATION	-0,117	0,001	1,1E-05	0,1007
BILD_MYC_ONCOGENIC_SIGNATURE	-0,167	5E-06	1,1E-05	0,1008
HERNANDEZ_ABERRANT_MITOSIS_BY_DOCET/	-0,124	7E-04	1,1E-05	0,1011
GNF2_RAB3A	-0,138	1E-04	1,1E-05	0,1014
REACTOME_SIGNALING_BY_ERBB4	-0,066	0,073	1,1E-05	0,1014
GSE17721_CPG_VS_GARDIQUIMOD_24H_BMI	-0,147	6E-05	1,2E-05	0,1023
GSE13411_NAIVE_VS_MEMORY_BCELL_UP	-0,126	6E-04	1,2E-05	0,103
CHIARETTI_T_ALL_RELAPSE_PROGNOSIS	-0,163	8E-06	1,2E-05	0,1031
V$SMAD3_Q6	-0,111	0,002	1,2E-05	0,1032
SHETH_LIVER_CANCER_VS_TXNIP_LOSS_PAN	-0,151	3E-05	1,2E-05	0,1033
MCBRYAN_PUBERTAL_BREAST_3_4WK_UP	-0,154	2E-05	1,2E-05	0,1034
GCM_CDH5	-0,144	8E-05	1,2E-05	0,1036
WGTTNNNNNAAA_UNKNOWN	-0,112	0,002	1,2E-05	0,104
CATION_TRANSPORT	-0,109	0,003	1,2E-05	0,1043
BLUM_RESPONSE_TO_SALIRASIB_DN	-0,166	5E-06	1,2E-05	0,1047
GSE6269_E_COLI_VS_STREP_PNEUMO_INF_P	-0,117	0,001	1,2E-05	0,1051
PENG_GLUCOSE_DEPRIVATION_DN	-0,191	2E-07	1,2E-05	0,1051
BANDRES_RESPONSE_TO_CARMUSTIN_WITHC	-0,152	3E-05	1,2E-05	0,1054
WANG_PROSTATE_CANCER_ANDROGEN_INDE	-0,129	4E-04	1,2E-05	0,1062
GSE17721_PAM3CSK4_VS_CPG_0.5H_BMDM	-0,127	5E-04	1,2E-05	0,107
V$COMP1_01	-0,12	1E-03	1,2E-05	0,1071
GSE15659_RESTING_TREG_VS_NONSUPPRESS	-0,135	2E-04	1,2E-05	0,1073
MIKKELSEN_NPC_HCP_WITH_H3K4ME3_AND_	-0,131	3E-04	1,2E-05	0,1075
GSE3982_NEUTROPHIL_VS_BCELL_DN	-0,168	4E-06	1,2E-05	0,1075
BIOCARTA_TRKA_PATHWAY	-0,153	3E-05	1,2E-05	0,1088

GSE12366_GC_VS_NAIVE_BCELL_UP	-0,177	1E-06	1,2E-05	0,109
GSE27786_LSK_VS_CD8_TCELL_UP	-0,159	1E-05	1,2E-05	0,1096
GSE24142_ADULT_VS_FETAL_EARLY_THYMI	-0,193	1E-07	1,2E-05	0,11
BUYTAERT_PHOTODYNAMIC_THERAPY_STRES	-0,177	1E-06	1,2E-05	0,1101
GSE22886_NAIVE_BCELL_VS_MONOCYTE_UF	-0,153	3E-05	1,3E-05	0,1105
ST_INTEGRIN_SIGNALING_PATHWAY	-0,164	7E-06	1,3E-05	0,1106
YAMASHITA_LIVER_CANCER_STEM_CELL_UP	-0,137	2E-04	1,3E-05	0,1106
KOKKINAKIS_METHIONINE_DEPRIVATION_48H	-0,152	3E-05	1,3E-05	0,1112
GSE6566_STRONG_VS_WEAK_DC_STIMULAT	-0,155	2E-05	1,3E-05	0,1115
PEART_HDAC_PROLIFERATION_CLUSTER_UP	-0,157	2E-05	1,3E-05	0,1116
SHI_SPARC_TARGETS_DN	-0,075	0,042	1,3E-05	0,1117
CHIARETTI_ACUTE_LYMPHOBLASTIC_LEUKEMI	-0,126	6E-04	1,3E-05	0,1132
V$AP1_C	-0,149	4E-05	1,3E-05	0,1134
HOLLEMAN_PREDNISOLONE_RESISTANCE_ALL	-0,18	7E-07	1,3E-05	0,1139
AKT_UP.V1_DN	-0,103	0,005	1,3E-05	0,1143
RICKMAN_HEAD_AND_NECK_CANCER_F	-0,106	0,004	1,3E-05	0,1144
GSE32423_MEMORY_VS_NAIVE_CD8_TCELL_	-0,102	0,005	1,3E-05	0,1145
BIOCARTA_IL4_PATHWAY	-0,121	9E-04	1,3E-05	0,1152
POSITIVE_REGULATION_OF_CELL_PROLIFERAT	-0,155	2E-05	1,3E-05	0,1156
BECKER_TAMOXIFEN_RESISTANCE_DN	-0,11	0,003	1,3E-05	0,1161
KRAS.600_UP.V1_DN	-0,143	9E-05	1,3E-05	0,1162
AACTTT_UNKNOWN	-0,125	6E-04	1,3E-05	0,1164
ZHOU_CELL_CYCLE_GENES_IN_IR_RESPONSE_	-0,175	2E-06	1,3E-05	0,1165
MADAN_DPPA4_TARGETS	-0,151	4E-05	1,3E-05	0,1168
GSE29615_DAY3_VS_DAY7_LAIV_FLU_VACC	-0,157	2E-05	1,3E-05	0,1174
IL2_UP.V1_DN	-0,112	0,002	1,3E-05	0,1179
MRNA_BINDING	-0,177	1E-06	1,3E-05	0,1185
YAO_TEMPORAL_RESPONSE_TO_PROGESTERC	-0,192	1E-07	1,4E-05	0,1188
GSE36392_EOSINOPHIL_VS_MAC_IL25_TREA	-0,106	0,004	1,4E-05	0,1188
SASAKI_ADULT_T_CELL_LEUKEMIA	-0,175	1E-06	1,4E-05	0,1191
HAHTOLA_MYCOSIS_FUNGOIDES_DN	-0,112	0,002	1,4E-05	0,1191
NOTCH_DN.V1_DN	-0,127	5E-04	1,4E-05	0,1192
MATSUDA_NATURAL_KILLER_DIFFERENTIATIC	-0,163	8E-06	1,4E-05	0,1199
V$NFKB_C	-0,138	2E-04	1,4E-05	0,1206
STK33_DN	-0,15	4E-05	1,4E-05	0,121
LI_CYTIDINE_ANALOGS_CYCTOTOXICITY	-0,179	9E-07	1,4E-05	0,121
CACTGTG,MIR-128A,MIR-128B	-0,14	1E-04	1,4E-05	0,1215
GSE360_HIGH_VS_LOW_DOSE_B_MALAYI_M/	-0,103	0,005	1,4E-05	0,1217
GCTNWTTGK_UNKNOWN	-0,15	4E-05	1,4E-05	0,122
KAECH_DAY15_EFF_VS_MEMORY_CD8_TCEL	-0,138	2E-04	1,4E-05	0,122
RNTCANNRNNYNATTW_UNKNOWN	-0,119	0,001	1,4E-05	0,122
V$NFMUE1_Q6	-0,143	9E-05	1,4E-05	0,1225

V$SREBP_Q3	-0,12	0,001	1,4E-05	0,1228
CASORELLI_ACUTE_PROMYELOCYTIC_LEUKEM	-0,181	6E-07	1,4E-05	0,1229
BRUINS_UVC_RESPONSE_VIA_TP53_GROUP_[	-0,138	2E-04	1,4E-05	0,1231
PID_BETACATENIN_NUC_PATHWAY	-0,152	3E-05	1,4E-05	0,1233
LEE_AGING_MUSCLE_UP	-0,088	0,016	1,4E-05	0,1236
MORF_RAB3A	-0,097	0,008	1,4E-05	0,1237
MODULE_48	-0,114	0,002	1,4E-05	0,1238
XU_GH1_AUTOCRINE_TARGETS_DN	-0,039	0,292	1,4E-05	0,1238
CHROMATIN	-0,18	8E-07	1,4E-05	0,1247
V$CDPCR3HD_01	-0,102	0,005	1,4E-05	0,1247
PANGAS_TUMOR_SUPPRESSION_BY_SMAD1_/	-0,125	6E-04	1,4E-05	0,125
V$FOXO1_02	-0,131	4E-04	1,4E-05	0,1253
HEPARIN_BINDING	-0,107	0,003	1,4E-05	0,126
V$NCX_01	-0,13	4E-04	1,4E-05	0,1262
POS_RESPONSE_TO_HISTAMINE_UP	-0,181	7E-07	1,4E-05	0,1265
GSE32423_CTRL_VS_IL7_IL4_MEMORY_CD8_	-0,148	5E-05	1,4E-05	0,1266
RRAGTTGT_UNKNOWN	-0,123	8E-04	1,4E-05	0,1266
SENESE_HDAC2_TARGETS_DN	-0,142	1E-04	1,4E-05	0,1266
REACTOME_G1_PHASE	-0,17	3E-06	1,4E-05	0,1267
GSE27786_LSK_VS_NKTCELL_DN	-0,155	2E-05	1,4E-05	0,1268
SAMOLS_TARGETS_OF_KHSV_MIRNAS_UP	-0,202	3E-08	1,4E-05	0,1269
GSE360_T_GONDII_VS_B_MALAYI_HIGH_DOS	-0,168	4E-06	1,4E-05	0,127
LY_AGING_PREMATURE_DN	-0,174	2E-06	1,5E-05	0,1273
PROTEIN_METABOLIC_PROCESS	-0,15	4E-05	1,5E-05	0,1275
GSE22045_TREG_VS_TCONV_DN	-0,094	0,01	1,5E-05	0,1278
MEISSNER_NPC_HCP_WITH_H3K4ME3_AND_H	-0,134	2E-04	1,5E-05	0,128
RESPONSE_TO_DNA_DAMAGE_STIMULUS	-0,145	8E-05	1,5E-05	0,1281
LU_AGING_BRAIN_UP	-0,126	6E-04	1,5E-05	0,1281
NUCLEOPLASM_PART	-0,174	2E-06	1,5E-05	0,1282
CELLULAR_MORPHOGENESIS_DURING_DIFFERE	-0,092	0,012	1,5E-05	0,1294
GSE360_CTRL_VS_B_MALAYI_LOW_DOSE_D(	-0,128	5E-04	1,5E-05	0,1296
chr4q21	-0,164	6E-06	1,5E-05	0,1297
YOSHIMURA_MAPK8_TARGETS_UP	-0,109	0,003	1,5E-05	0,1308
GCM_HMGA2	-0,134	2E-04	1,5E-05	0,1308
GSE14769_UNSTIM_VS_20MIN_LPS_BMDM_U	-0,116	0,002	1,5E-05	0,1318
PATTERN_SPECIFICATION_PROCESS	-0,12	0,001	1,5E-05	0,1319
DOANE_RESPONSE_TO_ANDROGEN_DN	-0,083	0,024	1,5E-05	0,1319
ZHANG_GATA6_TARGETS_DN	-0,168	4E-06	1,5E-05	0,1323
GGCCAGT,MIR-193A,MIR-193B	-0,116	0,001	1,5E-05	0,1326
BURTON_ADIPOGENESIS_4	-0,162	9E-06	1,5E-05	0,1327
GSE12845_IGD_POS_BLOOD_VS_PRE_GC_TC	-0,09	0,014	1,5E-05	0,1332
IVANOVA_HEMATOPOIESIS_STEM_CELL	-0,103	0,005	1,5E-05	0,1333

MRNA_METABOLIC_PROCESS	-0,188	2E-07	1,5E-05	0,1334
PID_AURORA_A_PATHWAY	-0,165	6E-06	1,5E-05	0,1341
chr11q22	-0,161	1E-05	1,5E-05	0,1344
GSE12845_IGD_POS_VS_NEG_BLOOD_BCELL	-0,121	1E-03	1,5E-05	0,1349
SMID_BREAST_CANCER_RELAPSE_IN_BONE_[	-0,167	5E-06	1,5E-05	0,135
GSE19825_NAIVE_VS_DAY3_EFF_CD8_TCEL	-0,118	0,001	1,6E-05	0,1355
MORF_RBM8A	-0,083	0,024	1,6E-05	0,1358
GSE11864_CSF1_VS_CSF1_PAM3CYS_IN_MA	-0,177	1E-06	1,6E-05	0,1363
GCM_PRKAG1	-0,115	0,002	1,6E-05	0,1369
TTAYRTAA_V$E4BP4_01	-0,122	9E-04	1,6E-05	0,137
KOBAYASHI_EGFR_SIGNALING_24HR_DN	-0,163	8E-06	1,6E-05	0,1375
PRC2_SUZ12_UP.V1_DN	-0,134	2E-04	1,6E-05	0,1376
GSE20715_0H_VS_6H_OZONE_LUNG_DN	-0,142	1E-04	1,6E-05	0,1383
V$E4F1_Q6	-0,128	5E-04	1,6E-05	0,1384
GRADE_COLON_AND_RECTAL_CANCER_DN	-0,087	0,018	1,6E-05	0,1384
LIANG_HEMATOPOIESIS_STEM_CELL_NUMBER	-0,172	2E-06	1,6E-05	0,1386
NIELSEN_MALIGNAT_FIBROUS_HISTIOCYTOMA	-0,083	0,024	1,6E-05	0,1389
POSITIVE_REGULATION_OF_DEVELOPMENTAL	-0,139	1E-04	1,6E-05	0,139
PID_ERBB4_PATHWAY	-0,12	0,001	1,6E-05	0,1393
LIAO_METASTASIS	-0,185	4E-07	1,6E-05	0,1396
MODULE_382	-0,11	0,003	1,6E-05	0,1398
GROSS_HYPOXIA_VIA_ELK3_UP	-0,158	1E-05	1,6E-05	0,14
REACTOME_P75NTR_RECRUITS_SIGNALLING_(	-0,155	2E-05	1,6E-05	0,1402
LABBE_WNT3A_TARGETS_UP	-0,177	1E-06	1,6E-05	0,1402
MAGRANGEAS_MULTIPLE_MYELOMA_IGLL_VS	-0,155	2E-05	1,6E-05	0,1403
GSE15930_STIM_VS_STIM_AND_TRICHOSTAT	-0,138	2E-04	1,6E-05	0,1404
V$SRY_01	-0,115	0,002	1,6E-05	0,1404
chr12q	-0,137	2E-04	1,6E-05	0,1404
COLIN_PILOCYTIC_ASTROCYTOMA_VS_GLIOBI	-0,149	5E-05	1,6E-05	0,1407
TBK1.DN.48HRS_UP	-0,168	4E-06	1,6E-05	0,1418
ALCALAY_AML_BY_NPM1_LOCALIZATION_DN	-0,158	2E-05	1,6E-05	0,1421
LOPEZ_MBD_TARGETS	-0,16	1E-05	1,6E-05	0,1423
INSULIN_LIKE_GROWTH_FACTOR_RECEPTOR_	-0,071	0,054	1,6E-05	0,1424
V$NFAT_Q4_01	-0,127	5E-04	1,6E-05	0,1431
GOZGIT_ESR1_TARGETS_UP	-0,142	1E-04	1,6E-05	0,1434
REGULATION_OF_CELL_GROWTH	-0,117	0,001	1,7E-05	0,1438
HAHTOLA_MYCOSIS_FUNGOIDES_SKIN_UP	-0,115	0,002	1,7E-05	0,1438
SCHAVOLT_TARGETS_OF_TP53_AND_TP63	-0,177	1E-06	1,7E-05	0,1445
GUO_HEX_TARGETS_UP	-0,133	3E-04	1,7E-05	0,1447
ASTON_MAJOR_DEPRESSIVE_DISORDER_DN	-0,145	7E-05	1,7E-05	0,1449
GSE3982_EOSINOPHIL_VS_NKCELL_DN	-0,134	2E-04	1,7E-05	0,1454
GSE7852_LN_VS_THYMUS_TCONV_DN	-0,17	3E-06	1,7E-05	0,1457

DELACROIX_RAR_BOUND_ES	-0,117	0,001	1,7E-05	0,1459
DELYS_THYROID_CANCER_DN	-0,093	0,011	1,7E-05	0,146
CCCACAT,MIR-299-3P	-0,13	4E-04	1,7E-05	0,1464
REGULATION_OF_RESPONSE_TO_EXTERNAL_S	-0,145	7E-05	1,7E-05	0,1473
KEGG_LYSINE_DEGRADATION	-0,193	1E-07	1,7E-05	0,1474
GSE13411_SWITCHED_MEMORY_BCELL_VS_F	-0,161	1E-05	1,7E-05	0,1475
GSE15659_CD45RA_NEG_CD4_TCELL_VS_RI	-0,117	0,001	1,7E-05	0,1476
VALK_AML_WITH_T_8_21_TRANSLOCATION	-0,108	0,003	1,7E-05	0,1477
GSE11057_NAIVE_CD4_VS_PBMC_CD4_TCEL	-0,072	0,049	1,7E-05	0,1482
GINESTIER_BREAST_CANCER_20Q13_AMPLIFI	-0,152	3E-05	1,7E-05	0,1491
MODULE_192	-0,072	0,051	1,7E-05	0,1501
MATZUK_MEIOTIC_AND_DNA_REPAIR	-0,16	1E-05	1,7E-05	0,1502
GSE3982_EOSINOPHIL_VS_DC_UP	-0,133	3E-04	1,7E-05	0,1513
SCHWAB_TARGETS_OF_BMYB_POLYMORPHIC_	-0,108	0,003	1,7E-05	0,1517
BRCA1_DN.V1_DN	-0,101	0,006	1,7E-05	0,152
KRASNOSELSKAYA_ILF3_TARGETS_DN	-0,161	1E-05	1,8E-05	0,1524
V$MEIS1_01	-0,134	2E-04	1,8E-05	0,1526
LOPEZ_MESOTHELIOMA_SURVIVAL_WORST_V	-0,164	7E-06	1,8E-05	0,1527
MURAKAMI_UV_RESPONSE_24HR	-0,134	2E-04	1,8E-05	0,1534
NIKOLSKY_BREAST_CANCER_15Q26_AMPLICO	-0,144	8E-05	1,8E-05	0,1543
GSE29618_PRE_VS_DAY7_POST_LAIV_FLU_	-0,125	7E-04	1,8E-05	0,1545
MODULE_340	-0,136	2E-04	1,8E-05	0,1547
WWTAAGGC_UNKNOWN	-0,132	3E-04	1,8E-05	0,1554
BAE_BRCA1_TARGETS_DN	-0,128	5E-04	1,8E-05	0,1556
PROTEIN_DNA_COMPLEX_ASSEMBLY	-0,176	1E-06	1,8E-05	0,1558
V$EVI1_04	-0,106	0,004	1,8E-05	0,1559
GSE14308_TH1_VS_INDUCED_TREG_DN	-0,164	7E-06	1,8E-05	0,1561
ANATOMICAL_STRUCTURE_DEVELOPMENT	-0,139	1E-04	1,8E-05	0,1561
GSE9006_TYPE_1_DIABETES_AT_DX_VS_4M	-0,118	0,001	1,8E-05	0,1562
V$SRF_Q4	-0,126	6E-04	1,8E-05	0,1565
V$HP1SITEFACTOR_Q6	-0,134	3E-04	1,8E-05	0,1568
GSE17721_POLYIC_VS_GARDIQUIMOD_1H_BM	-0,139	1E-04	1,8E-05	0,157
REGULATION_OF_MYELOID_CELL_DIFFERENTIA	-0,125	6E-04	1,8E-05	0,1574
GSE18791_CTRL_VS_NEWCASTLE_VIRUS_DC	-0,185	3E-07	1,8E-05	0,1582
V$PPARA_01	-0,078	0,033	1,8E-05	0,1587
GSE13485_PRE_VS_POST_YF17D_VACCINAT	-0,156	2E-05	1,8E-05	0,1587
SAMOLS_TARGETS_OF_KHSV_MIRNAS_DN	-0,123	7E-04	1,9E-05	0,1608
MODULE_308	-0,157	2E-05	1,9E-05	0,161
GSE27786_LIN_NEG_VS_NKTCELL_UP	-0,164	7E-06	1,9E-05	0,1611
GSE27786_CD4_TCELL_VS_NKCELL_UP	-0,093	0,011	1,9E-05	0,1622
DCA_UP.V1_DN	-0,151	3E-05	1,9E-05	0,1629
GSE360_CTRL_VS_L_MAJOR_MAC_DN	-0,145	7E-05	1,9E-05	0,1629

DARWICHE_SQUAMOUS_CELL_CARCINOMA_D	-0,142	1E-04	1,9E-05	0,1637
ACAWNRNSRCGG_UNKNOWN	-0,112	0,002	1,9E-05	0,1654
V$ETF_Q6	-0,156	2E-05	1,9E-05	0,1655
ESC_J1_UP_LATE.V1_UP	-0,119	0,001	1,9E-05	0,1656
V$ATF6_01	-0,084	0,022	1,9E-05	0,1656
PID_EPHBFWDPATHWAY	-0,18	8E-07	1,9E-05	0,1674
REACTOME_GPCR_DOWNSTREAM_SIGNALING	-0,127	5E-04	1,9E-05	0,1674
WYAAANNRNNNGCG_UNKNOWN	-0,12	0,001	1,9E-05	0,1676
V$HNF3_Q6	-0,105	0,004	1,9E-05	0,1686
BIOCARTA_PGC1A_PATHWAY	-0,143	9E-05	2E-05	0,1688
LINDGREN_BLADDER_CANCER_HIGH_RECURRE	-0,148	5E-05	2E-05	0,169
MODULE_131	-0,115	0,002	2E-05	0,1703
PROTEINACEOUS_EXTRACELLULAR_MATRIX	-0,122	9E-04	2E-05	0,1707
MODULE_26	-0,156	2E-05	2E-05	0,1709
YYCATTCAWW_UNKNOWN	-0,149	4E-05	2E-05	0,1709
TRANSMISSION_OF_NERVE_IMPULSE	-0,117	0,001	2E-05	0,1713
MITOTIC_CELL_CYCLE_CHECKPOINT	-0,164	7E-06	2E-05	0,1715
TRANSCRIPTION_FACTOR_ACTIVITY	-0,125	6E-04	2E-05	0,1718
REACTOME_PIP3_ACTIVATES_AKT_SIGNALIN(	-0,082	0,025	2E-05	0,1718
GSE17721_CPG_VS_GARDIQUIMOD_12H_BMI	-0,156	2E-05	2E-05	0,1719
GAUSSMANN_MLL_AF4_FUSION_TARGETS_G_	-0,167	4E-06	2E-05	0,1723
LINDGREN_BLADDER_CANCER_CLUSTER_1_D	-0,168	4E-06	2E-05	0,1728
V$USF2_Q6	-0,144	8E-05	2E-05	0,1734
POSITIVE_REGULATION_OF_TRANSCRIPTIONDI	-0,105	0,004	2E-05	0,1735
WEBER_METHYLATED_IN_COLON_CANCER	-0,095	0,01	2E-05	0,1736
GUILLAUMOND_KLF10_TARGETS_UP	-0,133	3E-04	2E-05	0,1739
PROTEIN_POLYMERIZATION	-0,085	0,021	2E-05	0,1746
MORF_CDC2L5	-0,083	0,023	2E-05	0,1747
KUNINGER_IGF1_VS_PDGFB_TARGETS_UP	-0,117	0,001	2E-05	0,1749
IWANAGA_CARCINOGENESIS_BY_KRAS_PTEN_	-0,157	2E-05	2E-05	0,175
THEODOROU_MAMMARY_TUMORIGENESIS	-0,094	0,011	2E-05	0,1755
MASRI_RESISTANCE_TO_TAMOXIFEN_AND_AI	-0,162	8E-06	2E-05	0,1755
GATAAGR_V$GATA_C	-0,117	0,001	2E-05	0,1759
V$PXR_Q2	-0,096	0,008	2E-05	0,1764
BLALOCK_ALZHEIMERS_DISEASE_DN	-0,167	4E-06	2E-05	0,1766
GEISS_RESPONSE_TO_DSRNA_DN	-0,199	4E-08	2,1E-05	0,1769
ATGCTGG,MIR-338	-0,086	0,019	2,1E-05	0,177
AAANWWTGC_UNKNOWN	-0,126	6E-04	2,1E-05	0,1772
GSE10325_LUPUS_BCELL_VS_LUPUS_MYELO	-0,163	8E-06	2,1E-05	0,1774
YANG_BCL3_TARGETS_UP	-0,143	9E-05	2,1E-05	0,1776
GSE1460_INTRATHYMIC_T_PROGENITOR_VS_	-0,172	2E-06	2,1E-05	0,1777
KAECH_DAY8_EFF_VS_MEMORY_CD8_TCELL_	-0,092	0,012	2,1E-05	0,1781

GSE14308_TH2_VS_NATURAL_TREG_UP	-0,156	2E-05	2,1E-05	0,1782
BIOCARTA_MAL_PATHWAY	-0,163	7E-06	2,1E-05	0,1783
MUSCLE_DEVELOPMENT	-0,102	0,006	2,1E-05	0,1799
RAO_BOUND_BY_SALL4_ISOFORM_B	-0,152	3E-05	2,1E-05	0,1803
GSE15930_STIM_VS_STIM_AND_IL-12_72H_	-0,117	0,001	2,1E-05	0,1803
REACTOME_OPIOID_SIGNALLING	-0,134	3E-04	2,1E-05	0,1807
LE_NEURONAL_DIFFERENTIATION_DN	-0,154	2E-05	2,1E-05	0,1809
CTCCAAG,MIR-432	-0,111	0,002	2,1E-05	0,1813
GSE29615_CTRL_VS_DAY7_LAIV_FLU_VACC	-0,133	3E-04	2,1E-05	0,1816
EXTRACELLULAR_MATRIX	-0,121	9E-04	2,1E-05	0,1816
KEGG_MELANOGENESIS	-0,141	1E-04	2,1E-05	0,1817
NAGY_STAGA_COMPONENTS_HUMAN	-0,119	0,001	2,1E-05	0,1819
PTEN_DN.V1_DN	-0,128	5E-04	2,1E-05	0,1821
BOHN_PRIMARY_IMMUNODEFICIENCY_SYNDRO	-0,171	3E-06	2,1E-05	0,1822
POSITIVE_REGULATION_OF_DEFENSE_RESPON	-0,164	6E-06	2,2E-05	0,1852
KEGG_THYROID_CANCER	-0,12	0,001	2,2E-05	0,1854
PURBEY_TARGETS_OF_CTBP1_NOT_SATB1_U	-0,146	6E-05	2,2E-05	0,1855
MODULE_512	-0,109	0,003	2,2E-05	0,1893
IONOTROPIC_GLUTAMATE_RECEPTOR_ACTIVIT	-0,07	0,056	2,2E-05	0,1894
NUCLEAR_CHROMOSOME	-0,17	3E-06	2,2E-05	0,1896
CAHOY_NEURONAL	-0,122	8E-04	2,2E-05	0,1897
GSE27786_CD8_TCELL_VS_NEUTROPHIL_UP	-0,177	1E-06	2,2E-05	0,1898
GSE17721_LPS_VS_POLYIC_1H_BMDM_DN	-0,132	3E-04	2,2E-05	0,1898
GSE27786_BCELL_VS_MONO_MAC_DN	-0,158	2E-05	2,2E-05	0,1903
GSE17721_POLYIC_VS_PAM3CSK4_1H_BMDM	-0,103	0,005	2,2E-05	0,1903
GAGCCTG,MIR-484	-0,127	5E-04	2,2E-05	0,1908
G_PROTEIN_SIGNALING_COUPLED_TO_CYCLIC	-0,117	0,001	2,2E-05	0,1908
HADDAD_T_LYMPHOCYTE_AND_NK_PROGENI	-0,102	0,006	2,2E-05	0,1908
GSE31082_CD4_VS_CD8_SP_THYMOCYTE_D	-0,153	3E-05	2,2E-05	0,191
ZEMBUTSU_SENSITIVITY_TO_CYCLOPHOSPHAI	-0,081	0,027	2,2E-05	0,1913
V$GCM_Q2	-0,138	2E-04	2,2E-05	0,1918
GSE22045_TREG_VS_TCONV_UP	-0,166	5E-06	2,2E-05	0,1922
V$TATA_C	-0,105	0,004	2,2E-05	0,1923
GSE29618_MONOCYTE_VS_MDC_DN	-0,149	5E-05	2,2E-05	0,1926
GSE22886_IL2_VS_IL15_STIM_NKCELL_DN	-0,175	1E-06	2,2E-05	0,193
CHIN_BREAST_CANCER_COPY_NUMBER_UP	-0,188	2E-07	2,2E-05	0,193
GCM_ERCC4	-0,113	0,002	2,3E-05	0,1941
CELL_SURFACE_RECEPTOR_LINKED_SIGNAL_T	-0,12	0,001	2,3E-05	0,1953
FEVR_CTNNB1_TARGETS_DN	-0,176	1E-06	2,3E-05	0,1965
ACTIN_FILAMENT_POLYMERIZATION	-0,128	5E-04	2,3E-05	0,1969
ACAACCT,MIR-453	-0,126	6E-04	2,3E-05	0,1971
CTAWWWATA_V$RSRFC4_Q2	-0,1	0,007	2,3E-05	0,1974

COLLER_MYC_TARGETS_DN	-0,14	1E-04	2,3E-05	0,2006
V$RSRFC4_Q2	-0,094	0,01	2,3E-05	0,2013
GSE22886_IL2_VS_IL15_STIM_NKCELL_UP	-0,12	0,001	2,4E-05	0,2017
REGULATION_OF_DEVELOPMENTAL_PROCESS	-0,141	1E-04	2,4E-05	0,2018
CYCLIC_NUCLEOTIDE_MEDIATED_SIGNALING	-0,114	0,002	2,4E-05	0,2023
IWANAGA_CARCINOGENESIS_BY_KRAS_DN	-0,076	0,039	2,4E-05	0,2023
GSE22886_NAIVE_CD4_TCELL_VS_MEMORY_	-0,136	2E-04	2,4E-05	0,2025
METAL_ION_TRANSMEMBRANE_TRANSPORTEI	-0,103	0,005	2,4E-05	0,2028
TRANSMEMBRANE_RECEPTOR_PROTEIN_TYRO	-0,128	5E-04	2,4E-05	0,2029
V$HSF1_01	-0,113	0,002	2,4E-05	0,2038
GGCNKCCATNK_UNKNOWN	-0,141	1E-04	2,4E-05	0,2039
CAFFAREL_RESPONSE_TO_THC_DN	-0,169	4E-06	2,4E-05	0,2041
GCNP_SHH_UP_LATE.V1_UP	-0,167	4E-06	2,4E-05	0,2043
WEST_ADRENOCORTICAL_TUMOR_DN	-0,073	0,047	2,4E-05	0,2047
GSE3982_EOSINOPHIL_VS_BASOPHIL_DN	-0,132	3E-04	2,4E-05	0,2054
MODULE_320	-0,158	1E-05	2,4E-05	0,2057
TTANWNANTGGM_UNKNOWN	-0,129	4E-04	2,4E-05	0,2077
KEGG_OTHER_GLYCAN_DEGRADATION	0,171	3E-06	2,4E-05	0,2087
GSE12845_IGD_NEG_BLOOD_VS_NAIVE_TON	-0,111	0,002	2,4E-05	0,2091
GSE30083_SP2_VS_SP3_THYMOCYTE_DN	-0,094	0,011	2,4E-05	0,2095
GSE17721_LPS_VS_POLYIC_0.5H_BMDM_UP	-0,111	0,002	2,5E-05	0,2098
STRUCTURAL_CONSTITUENT_OF_MUSCLE	-0,126	6E-04	2,5E-05	0,2103
HETEROPHILIC_CELL_ADHESION	-0,139	1E-04	2,5E-05	0,2119
BERTUCCI_INVASIVE_CARCINOMA_DUCTAL_V	-0,087	0,018	2,5E-05	0,2127
FERREIRA_EWINGS_SARCOMA_UNSTABLE_VS_	-0,158	2E-05	2,5E-05	0,214
GSE3982_DC_VS_BASOPHIL_DN	-0,107	0,003	2,5E-05	0,2176
GRABARCZYK_BCL11B_TARGETS_DN	-0,139	1E-04	2,6E-05	0,2181
MODULE_447	-0,145	7E-05	2,6E-05	0,2186
V$STAT_01	-0,127	5E-04	2,6E-05	0,2188
STEROID_HORMONE_RECEPTOR_BINDING	-0,144	8E-05	2,6E-05	0,2188
REACTOME_SIGNALLING_TO_P38_VIA_RIT_AI	-0,165	6E-06	2,6E-05	0,2192
GSE15659_NAIVE_CD4_TCELL_VS_RESTING_	-0,132	3E-04	2,6E-05	0,2199
GSE11057_NAIVE_VS_MEMORY_CD4_TCELL_	-0,108	0,003	2,6E-05	0,2199
GRESHOCK_CANCER_COPY_NUMBER_UP	-0,159	1E-05	2,6E-05	0,2202
RAY_TUMORIGENESIS_BY_ERBB2_CDC25A_UI	-0,14	1E-04	2,6E-05	0,2211
FLOTHO_PEDIATRIC_ALL_THERAPY_RESPONS	-0,136	2E-04	2,6E-05	0,2211
NOJIMA_SFRP2_TARGETS_UP	-0,102	0,005	2,6E-05	0,2216
WANG_LMO4_TARGETS_UP	-0,064	0,081	2,6E-05	0,2218
V$MYC_Q2	-0,155	2E-05	2,6E-05	0,222
LEE_TARGETS_OF_PTCH1_AND_SUFU_DN	-0,097	0,008	2,6E-05	0,2224
V$SOX5_01	-0,108	0,003	2,6E-05	0,2225
TTANTCA_UNKNOWN	-0,137	2E-04	2,6E-05	0,2225

REACTOME_G_ALPHA_S_SIGNALLING_EVENT!	-0,086	0,019	2,6E-05	0,2232
MOLENAAR_TARGETS_OF_CCND1_AND_CDK4	-0,085	0,02	2,6E-05	0,2254
ZWANG_CLASS_2_TRANSIENTLY_INDUCED_B	-0,138	2E-04	2,6E-05	0,2255
BIOCARTA_PLATELETAPP_PATHWAY	-0,154	2E-05	2,7E-05	0,227
MARIADASON_RESPONSE_TO_BUTYRATE_SUL	-0,141	1E-04	2,7E-05	0,227
V$PR_01	-0,102	0,005	2,7E-05	0,228
GSE15930_STIM_VS_STIM_AND_TRICHOSTAT	-0,139	1E-04	2,7E-05	0,2283
V$ATF1_Q6	-0,111	0,003	2,7E-05	0,2289
REGULATION_OF_CYCLIN_DEPENDENT_PROTE	-0,159	1E-05	2,7E-05	0,229
ZWANG_TRANSIENTLY_UP_BY_2ND_EGF_PUL	-0,105	0,004	2,7E-05	0,2291
V$CREL_01	-0,145	8E-05	2,7E-05	0,231
OHM_EMBRYONIC_CARCINOMA_UP	-0,199	5E-08	2,7E-05	0,2314
GSE27786_NEUTROPHIL_VS_MONO_MAC_DN	-0,168	4E-06	2,7E-05	0,2314
GSE360_HIGH_VS_LOW_DOSE_B_MALAYI_DC	-0,109	0,003	2,7E-05	0,2314
YKACATTT_UNKNOWN	-0,106	0,004	2,7E-05	0,2316
AATGTGA,MIR-23A,MIR-23B	-0,141	1E-04	2,7E-05	0,2327
PHOTORECEPTOR_CELL_MAINTENANCE	-0,131	3E-04	2,7E-05	0,2337
GSE3982_BASOPHIL_VS_EFF_MEMORY_CD4_	-0,13	4E-04	2,7E-05	0,2339
V$PAX_Q6	-0,101	0,006	2,8E-05	0,2345
MODULE_175	-0,126	6E-04	2,8E-05	0,2347
GSE13306_TREG_RA_VS_TCONV_RA_DN	-0,114	0,002	2,8E-05	0,2363
MODULE_244	-0,165	6E-06	2,8E-05	0,2374
CHIARADONNA_NEOPLASTIC_TRANSFORMATI(	-0,15	4E-05	2,8E-05	0,2376
MODULE_118	-0,175	2E-06	2,8E-05	0,2381
GGCAGAC,MIR-346	-0,124	7E-04	2,8E-05	0,2381
STEIN_ESRRA_TARGETS_RESPONSIVE_TO_ES'	-0,084	0,022	2,8E-05	0,2382
KRAS.DF.V1_DN	-0,146	7E-05	2,8E-05	0,2383
LEE_TARGETS_OF_PTCH1_AND_SUFU_UP	-0,137	2E-04	2,8E-05	0,2385
V$LMO2COM_02	-0,114	0,002	2,8E-05	0,2385
SYNAPTIC_TRANSMISSION	-0,113	0,002	2,8E-05	0,2386
MORI_SMALL_PRE_BII_LYMPHOCYTE_UP	-0,133	3E-04	2,8E-05	0,2387
V$OCT1_B	-0,113	0,002	2,8E-05	0,2395
GSE17721_LPS_VS_PAM3CSK4_4H_BMDM_L	-0,137	2E-04	2,8E-05	0,2418
AAAYRNCTG_UNKNOWN	-0,105	0,004	2,9E-05	0,2423
FOURNIER_ACINAR_DEVELOPMENT_LATE_2	-0,172	2E-06	2,9E-05	0,2431
BILANGES_SERUM_RESPONSE_TRANSLATION	-0,132	3E-04	2,9E-05	0,2439
PUJANA_BRCA2_PCC_NETWORK	-0,16	1E-05	2,9E-05	0,2439
GSE9037_CTRL_VS_LPS_1H_STIM_IRAK4_K(	-0,078	0,032	2,9E-05	0,2441
CAGTCAC,MIR-134	-0,121	9E-04	2,9E-05	0,2444
V$OCT1_Q5_01	-0,115	0,002	2,9E-05	0,2444
POSITIVE_REGULATION_OF_RNA_METABOLIC_	-0,105	0,004	2,9E-05	0,245
MICROTUBULE_BINDING	-0,154	2E-05	2,9E-05	0,2459

AMINO_ACID_DERIVATIVE_BIOSYNTHETIC_PR(	-0,099	0,007	2,9E-05	0,2465
BIOCARTA_P38MAPK_PATHWAY	-0,132	3E-04	2,9E-05	0,2465
NIKOLSKY_BREAST_CANCER_7Q21_Q22_AMI	-0,138	2E-04	2,9E-05	0,2469
FRIDMAN_SENESCENCE_DN	-0,134	2E-04	2,9E-05	0,2474
RESPONSE_TO_LIGHT_STIMULUS	-0,119	0,001	2,9E-05	0,2477
KANG_AR_TARGETS_UP	-0,154	2E-05	2,9E-05	0,249
GSE30962_PRIMARY_VS_SECONDARY_ACUTE	-0,155	2E-05	2,9E-05	0,2492
MITOSIS	-0,159	1E-05	2,9E-05	0,2502
SODIUM_CHANNEL_ACTIVITY	-0,088	0,016	3E-05	0,2534
LEE_NEURAL_CREST_STEM_CELL_UP	-0,129	4E-04	3E-05	0,2546
ORGAN_DEVELOPMENT	-0,139	1E-04	3E-05	0,2548
CTACCTC,LET-7A,LET-7B,LET-7C,LET-7D,LET-	-0,134	2E-04	3E-05	0,2559
SCHRAETS_MLL_TARGETS_UP	-0,13	4E-04	3E-05	0,2561
REACTOME_SIGNALING_BY_RHO_GTPASES	-0,126	6E-04	3E-05	0,2564
GSE34205_HEALTHY_VS_FLU_INF_INFANT_F	-0,098	0,007	3E-05	0,2566
V$TCF11_01	-0,11	0,003	3E-05	0,2575
GSE36392_TYPE_2_MYELOID_VS_MAC_IL25_	-0,112	0,002	3E-05	0,258
REGULATION_OF_CELL_PROLIFERATION	-0,148	5E-05	3,1E-05	0,2585
V$GR_01	-0,1	0,006	3,1E-05	0,2587
YAGUE_PRETUMOR_DRUG_RESISTANCE_UP	-0,135	2E-04	3,1E-05	0,2595
GSE20715_0H_VS_24H_OZONE_TLR4_KO_L	-0,113	0,002	3,1E-05	0,2641
VERRECCHIA_RESPONSE_TO_TGFB1_C1	-0,114	0,002	3,1E-05	0,2668
GSE1460_DP_THYMOCYTE_VS_THYMIC_STR(	-0,158	2E-05	3,2E-05	0,2669
ATGGYGGA_UNKNOWN	-0,113	0,002	3,2E-05	0,2671
KRAS.KIDNEY_UP.V1_UP	-0,104	0,005	3,2E-05	0,2681
DORSEY_GAB2_TARGETS	-0,139	1E-04	3,2E-05	0,2684
MARTIN_INTERACT_WITH_HDAC	-0,126	6E-04	3,2E-05	0,2691
CELL_PROJECTION	-0,093	0,011	3,2E-05	0,2692
POMEROY_MEDULLOBLASTOMA_PROGNOSIS_I	-0,168	4E-06	3,2E-05	0,2698
RIZ_ERYTHROID_DIFFERENTIATION_APOBEC2	-0,12	0,001	3,2E-05	0,2698
GSE29615_CTRL_VS_DAY7_LAIV_FLU_VACC	-0,123	8E-04	3,2E-05	0,2723
V$PAX4_04	-0,117	0,001	3,2E-05	0,2724
GARGALOVIC_RESPONSE_TO_OXIDIZED_PHOS	-0,139	1E-04	3,2E-05	0,2725
GSE13484_UNSTIM_VS_12H_YF17D_VACCIN	-0,093	0,011	3,2E-05	0,2726
GSE22886_NAIVE_CD4_TCELL_VS_12H_ACT	-0,066	0,071	3,3E-05	0,2756
GSE360_L_DONOVANI_VS_L_MAJOR_MAC_D	-0,134	3E-04	3,3E-05	0,2758
MIKKELSEN_IPS_ICP_WITH_H3K4ME3_AND_H	-0,104	0,004	3,3E-05	0,276
ENGELMANN_CANCER_PROGENITORS_UP	-0,078	0,033	3,3E-05	0,2762
WEI_MIR34A_TARGETS	-0,136	2E-04	3,3E-05	0,2768
MODULE_52	-0,185	4E-07	3,3E-05	0,277
GSE3982_MAST_CELL_VS_MAC_UP	-0,136	2E-04	3,3E-05	0,2777
MODULE_485	-0,164	7E-06	3,3E-05	0,2783

CTGAGCC,MIR-24	-0,103	0,005	3,3E-05	0,2807
VARELA_ZMPSTE24_TARGETS_UP	-0,066	0,07	3,3E-05	0,2817
NERVOUS_SYSTEM_DEVELOPMENT	-0,139	1E-04	3,3E-05	0,2819
DING_LUNG_CANCER_MUTATED_RECURRENTL	-0,16	1E-05	3,4E-05	0,2847
KIM_MYCL1_AMPLIFICATION_TARGETS_UP	-0,12	0,001	3,4E-05	0,2849
MORF_RBBP8	-0,092	0,012	3,4E-05	0,2855
GSE11924_TH1_VS_TH17_CD4_TCELL_UP	-0,127	5E-04	3,4E-05	0,2871
OHGUCHI_LIVER_HNF4A_TARGETS_UP	-0,138	2E-04	3,4E-05	0,2874
GSE32423_MEMORY_VS_NAIVE_CD8_TCELL_	-0,128	5E-04	3,4E-05	0,2874
V$PBX1_02	-0,146	6E-05	3,4E-05	0,2876
BIOCARTA_EPHA4_PATHWAY	-0,17	3E-06	3,4E-05	0,2877
KRIEG_HYPOXIA_NOT_VIA_KDM3A	-0,174	2E-06	3,4E-05	0,288
PROTEIN_AMINO_ACID_PHOSPHORYLATION	-0,134	2E-04	3,4E-05	0,2892
V$PPAR_DR1_Q2	-0,097	0,008	3,4E-05	0,2894
POTTI_CYTOXAN_SENSITIVITY	-0,154	2E-05	3,4E-05	0,2902
chr1q32	-0,134	3E-04	3,4E-05	0,2903
JU_AGING_TERC_TARGETS_UP	-0,135	2E-04	3,5E-05	0,2912
REGULATION_OF_SYNAPSE_STRUCTURE_AND	-0,16	1E-05	3,5E-05	0,2927
GSE17721_POLYIC_VS_CPG_12H_BMDM_DN	-0,172	2E-06	3,5E-05	0,2936
WIEDERSCHAIN_TARGETS_OF_BMI1_AND_PC(	-0,169	3E-06	3,5E-05	0,2938
CCTGAGT,MIR-510	-0,084	0,021	3,5E-05	0,2968
TRANSFERASE_ACTIVITY_TRANSFERRING_PH(	-0,15	4E-05	3,5E-05	0,299
POSITIVE_REGULATION_OF_TRANSCRIPTION	-0,099	0,007	3,5E-05	0,299
GSE27786_LSK_VS_LIN_NEG_CELL_UP	-0,091	0,013	3,6E-05	0,3
CHEBOTAEV_GR_TARGETS_DN	-0,105	0,004	3,6E-05	0,3004
SHETH_LIVER_CANCER_VS_TXNIP_LOSS_PAM	-0,167	5E-06	3,6E-05	0,3011
GENTILE_UV_RESPONSE_CLUSTER_D7	-0,164	6E-06	3,6E-05	0,3022
GSE15733_BM_VS_SPLEEN_MEMORY_CD4_T	-0,141	1E-04	3,6E-05	0,3023
PID_RAS_PATHWAY	-0,149	5E-05	3,6E-05	0,3035
SENESE_HDAC1_AND_HDAC2_TARGETS_DN	-0,141	1E-04	3,6E-05	0,3035
GSE1460_INTRATHYMIC_T_PROGENITOR_VS_	-0,129	4E-04	3,6E-05	0,3035
V$ZIC1_01	-0,115	0,002	3,6E-05	0,3044
EBAUER_TARGETS_OF_PAX3_FOXO1_FUSION_	-0,106	0,004	3,6E-05	0,3053
PEDERSEN_METASTASIS_BY_ERBB2_ISOFORM	-0,109	0,003	3,6E-05	0,3062
CASTELLANO_NRAS_TARGETS_DN	-0,156	2E-05	3,6E-05	0,3067
MICROTUBULE_ORGANIZING_CENTER_PART	-0,137	2E-04	3,7E-05	0,3071
GSE30083_SP3_VS_SP4_THYMOCYTE_UP	-0,103	0,005	3,7E-05	0,3084
BRUINS_UVC_RESPONSE_VIA_TP53_GROUP_/	-0,106	0,004	3,7E-05	0,3086
GROSS_HYPOXIA_VIA_ELK3_ONLY_DN	-0,132	3E-04	3,7E-05	0,309
MARSON_BOUND_BY_E2F4_UNSTIMULATED	-0,157	2E-05	3,7E-05	0,31
CELL_MIGRATION	-0,136	2E-04	3,7E-05	0,3103
GSE17721_POLYIC_VS_PAM3CSK4_24H_BMI	-0,173	2E-06	3,7E-05	0,3106

REACTOME_ARMS_MEDIATED_ACTIVATION	-0,169	3E-06	3,7E-05	0,3112
GGCKCATGS_UNKNOWN	-0,079	0,031	3,7E-05	0,3118
DELASERNA_MYOD_TARGETS_UP	-0,086	0,018	3,7E-05	0,3118
GSE3982_MAC_VS_NKCELL_DN	-0,103	0,005	3,7E-05	0,3118
ENZYME_ACTIVATOR_ACTIVITY	-0,118	0,001	3,7E-05	0,312
GSE27786_BCELL_VS_NEUTROPHIL_DN	-0,112	0,002	3,7E-05	0,3131
AGTCTTA,MIR-499	-0,147	6E-05	3,7E-05	0,314
MORF_PRKCA	-0,136	2E-04	3,7E-05	0,3147
GSE18791_CTRL_VS_NEWCASTLE_VIRUS_DC	-0,172	2E-06	3,7E-05	0,3148
HYDROLASE_ACTIVITY_ACTING_ON_ACID_AN	-0,117	0,001	3,8E-05	0,3164
SENESE_HDAC1_TARGETS_DN	-0,1	0,006	3,8E-05	0,3166
MARTORIATI_MDM4_TARGETS_NEUROEPITHEL	-0,158	1E-05	3,8E-05	0,3184
V$AP4_01	-0,111	0,002	3,8E-05	0,3195
RORIE_TARGETS_OF_EWSR1_FLI1_FUSION_DI	-0,139	1E-04	3,8E-05	0,3195
INTEGRIN_BINDING	-0,133	3E-04	3,8E-05	0,3201
REACTOME_NCAM_SIGNALING_FOR_NEURITE_	-0,132	3E-04	3,8E-05	0,3208
YTATTTTNR_V$MEF2_02	-0,113	0,002	3,8E-05	0,3211
AMIT_SERUM_RESPONSE_120_MCF10A	-0,135	2E-04	3,8E-05	0,3212
ESC_V6.5_UP_LATE.V1_UP	-0,107	0,003	3,8E-05	0,3217
M_PHASE_OF_MITOTIC_CELL_CYCLE	-0,158	2E-05	3,9E-05	0,3235
MODULE_123	-0,139	1E-04	3,9E-05	0,3248
WANG_METHYLATED_IN_BREAST_CANCER	-0,175	2E-06	3,9E-05	0,3249
TGTTTGY_V$HNF3_Q6	-0,086	0,019	3,9E-05	0,3257
GSE24634_IL4_VS_CTRL_TREATED_NAIVE_C	-0,158	2E-05	3,9E-05	0,3266
PUJANA_ATM_PCC_NETWORK	-0,163	8E-06	3,9E-05	0,3272
BIOCARTA_SALMONELLA_PATHWAY	-0,19	2E-07	3,9E-05	0,3278
LANDIS_ERBB2_BREAST_TUMORS_65_DN	-0,114	0,002	3,9E-05	0,3281
BIOCARTA_CARDIACEGF_PATHWAY	-0,106	0,004	3,9E-05	0,3283
GSE3982_DC_VS_CENT_MEMORY_CD4_TCEL	-0,11	0,003	3,9E-05	0,3283
MODULE_164	-0,14	1E-04	3,9E-05	0,3286
GSE3982_BASOPHIL_VS_NKCELL_UP	-0,116	0,002	3,9E-05	0,33
GSE11864_CSF1_PAM3CYS_VS_CSF1_IFNG_	-0,16	1E-05	3,9E-05	0,3304
GSE17721_CTRL_VS_CPG_2H_BMDM_DN	-0,114	0,002	3,9E-05	0,3304
BIOCARTA_RAC1_PATHWAY	-0,164	7E-06	4E-05	0,3312
HARRIS_BRAIN_CANCER_PROGENITORS	-0,128	5E-04	4E-05	0,3312
HEIDENBLAD_AMPLIFIED_IN_SOFT_TISSUE_CA	-0,085	0,02	4E-05	0,3314
MORF_DMPK	-0,153	3E-05	4E-05	0,3325
MODULE_9	-0,095	0,01	4E-05	0,335
PID_NEPHRIN_NEPH1_PATHWAY	-0,137	2E-04	4E-05	0,3355
ODONNELL_METASTASIS_UP	-0,095	0,009	4E-05	0,3365
CELL_MATRIX_ADHESION	-0,116	0,001	4E-05	0,3373
DASU_IL6_SIGNALING_SCAR_UP	-0,147	6E-05	4E-05	0,3374

DITTMER_PTHLH_TARGETS_UP	-0,169	3E-06	4E-05	0,3384
FRIDMAN_IMMORTALIZATION_DN	-0,123	7E-04	4E-05	0,3386
ATF2_S_UP.V1_UP	-0,157	2E-05	4,1E-05	0,3388
V$NFKAPPAB65_01	-0,142	1E-04	4,1E-05	0,3388
KYNG_DNA_DAMAGE_BY_4NQO_OR_UV	-0,135	2E-04	4,1E-05	0,3391
SCHUETZ_BREAST_CANCER_DUCTAL_INVASIV	-0,032	0,379	4,1E-05	0,3396
BROWNE_HCMV_INFECTION_12HR_DN	-0,15	4E-05	4,1E-05	0,3406
V$AP2_Q3	-0,131	3E-04	4,1E-05	0,3406
TOMLINS_PROSTATE_CANCER_DN	-0,118	0,001	4,1E-05	0,3406
GSE15659_TREG_VS_TCONV_UP	-0,124	7E-04	4,1E-05	0,3414
MORF_BRCA1	-0,16	1E-05	4,1E-05	0,3416
PROVENZANI_METASTASIS_UP	-0,163	8E-06	4,1E-05	0,3419
AGCGCAG,MIR-191	-0,042	0,256	4,1E-05	0,3423
KEGG_WNT_SIGNALING_PATHWAY	-0,146	6E-05	4,1E-05	0,3429
V$CHOP_01	-0,122	9E-04	4,1E-05	0,3439
V$PAX4_02	-0,109	0,003	4,1E-05	0,3439
V$DBP_Q6	-0,101	0,006	4,1E-05	0,3445
VOLTAGE_GATED_SODIUM_CHANNEL_ACTIVIT	-0,07	0,057	4,1E-05	0,3445
TGANTCA_V$AP1_C	-0,141	1E-04	4,1E-05	0,346
REACTOME_NEURONAL_SYSTEM	-0,113	0,002	4,2E-05	0,3469
ATGCAGT,MIR-217	-0,134	2E-04	4,2E-05	0,3473
TISSUE_DEVELOPMENT	-0,162	9E-06	4,2E-05	0,3493
MATTHEWS_AP1_TARGETS	-0,148	5E-05	4,2E-05	0,3501
KIM_WT1_TARGETS_UP	-0,15	4E-05	4,2E-05	0,3509
MTOR_UP.V1_DN	-0,096	0,009	4,2E-05	0,3511
chr9p13	-0,136	2E-04	4,2E-05	0,3514
GSE6269_HEALTHY_VS_FLU_INF_PBMC_UP	-0,103	0,005	4,2E-05	0,3515
HEDENFALK_BREAST_CANCER_HEREDITARY_V	-0,185	4E-07	4,2E-05	0,3525
GSE27786_NKCELL_VS_NEUTROPHIL_UP	-0,162	9E-06	4,2E-05	0,3527
GSE28237_FOLLICULAR_VS_LATE_GC_BCELL	-0,149	4E-05	4,2E-05	0,3532
REACTOME_PROLONGED_ERK_ACTIVATION_E	-0,157	2E-05	4,2E-05	0,3538
GGATTA_V$PITX2_Q2	-0,112	0,002	4,2E-05	0,3538
GSE17721_CTRL_VS_GARDIQUIMOD_1H_BMD	-0,157	2E-05	4,3E-05	0,3546
GSE8515_CTRL_VS_IL6_4H_STIM_MAC_DN	-0,097	0,008	4,3E-05	0,3552
AMIT_EGF_RESPONSE_240_HELA	-0,169	3E-06	4,3E-05	0,3562
PUJANA_BREAST_CANCER_WITH_BRCA1_MUT	-0,155	2E-05	4,3E-05	0,3562
GSE27786_LIN_NEG_VS_CD4_TCELL_UP	-0,182	6E-07	4,3E-05	0,3563
V$AFP1_Q6	-0,107	0,003	4,3E-05	0,3563
REACTOME_GASTRIN_CREB_SIGNALLING_PAT	-0,125	6E-04	4,3E-05	0,3578
GSE3982_EFF_MEMORY_CD4_TCELL_VS_NKC	-0,125	6E-04	4,3E-05	0,358
CAGCCTC,MIR-485-5P	-0,097	0,008	4,3E-05	0,3582
KEGG_PROGESTERONE_MEDIATED_OOCYTE_M	-0,151	3E-05	4,3E-05	0,359

LIM_MAMMARY_LUMINAL_PROGENITOR_DN	-0,114	0,002	4,3E-05	0,3592
BYSTRYKH_HEMATOPOIESIS_STEM_CELL_FGF	-0,172	2E-06	4,3E-05	0,3609
YATGNWAAT_V$OCT_C	-0,1	0,006	4,3E-05	0,3611
V$HFH4_01	-0,108	0,003	4,3E-05	0,3611
WATTEL_AUTONOMOUS_THYROID_ADENOMA	-0,143	9E-05	4,3E-05	0,3612
REACTOME_NOTCH1_INTRACELLULAR_DOMAI	-0,138	2E-04	4,3E-05	0,3616
GSE22886_NAIVE_VS_IGM_MEMORY_BCELL_	-0,096	0,009	4,4E-05	0,3621
PLASARI_TGFB1_SIGNALING_VIA_NFIC_1HR_I	-0,092	0,012	4,4E-05	0,365
GSE8515_CTRL_VS_IL1_4H_STIM_MAC_UP	-0,084	0,022	4,4E-05	0,366
GSE15659_TREG_VS_TCONV_DN	-0,101	0,006	4,4E-05	0,3684
BILANGES_SERUM_SENSITIVE_GENES	-0,115	0,002	4,4E-05	0,3689
KIM_WT1_TARGETS_DN	-0,17	3E-06	4,4E-05	0,369
DAIRKEE_CANCER_PRONE_RESPONSE_BPA_E2	-0,159	1E-05	4,4E-05	0,3692
GSE2706_LPS_VS_R848_AND_LPS_8H_STIM	-0,136	2E-04	4,4E-05	0,3692
KEGG_RENAL_CELL_CARCINOMA	-0,145	7E-05	4,5E-05	0,3701
HELLEBREKERS_SILENCED_DURING_TUMOR_A	-0,146	6E-05	4,5E-05	0,3705
GAVIN_FOXP3_TARGETS_CLUSTER_P7	-0,121	1E-03	4,5E-05	0,3708
GSE13411_NAIVE_VS_IGM_MEMORY_BCELL_	-0,137	2E-04	4,5E-05	0,3716
CAATGCA,MIR-33	-0,151	4E-05	4,5E-05	0,3717
CHICAS_RB1_TARGETS_CONFLUENT	-0,132	3E-04	4,5E-05	0,3724
GSE36476_YOUNG_VS_OLD_DONOR_MEMORY	-0,153	3E-05	4,5E-05	0,3724
AUXILIARY_TRANSPORT_PROTEIN_ACTIVITY	-0,08	0,03	4,5E-05	0,3765
GSE17721_POLYIC_VS_CPG_16H_BMDM_DN	-0,17	3E-06	4,5E-05	0,3769
BHATTACHARYA_EMBRYONIC_STEM_CELL	-0,17	3E-06	4,5E-05	0,3769
REACTOME_SPRY_REGULATION_OF_FGF_SIGN	-0,164	7E-06	4,5E-05	0,3772
GSE7460_CTRL_VS_TGFB_TREATED_ACT_TF	-0,171	3E-06	4,6E-05	0,3785
GSE27786_LSK_VS_ERYTHROBLAST_DN	-0,104	0,004	4,6E-05	0,3794
NIKOLSKY_MUTATED_AND_AMPLIFIED_IN_BRI	-0,096	0,009	4,6E-05	0,3827
HEIDENBLAD_AMPLIFIED_IN_PANCREATIC_CAI	-0,171	3E-06	4,6E-05	0,3828
GSE13306_RA_VS_UNTREATED_TREG_DN	-0,1	0,006	4,6E-05	0,3836
GNF2_RBBP6	-0,164	7E-06	4,7E-05	0,386
MODULE_8	-0,181	7E-07	4,7E-05	0,3862
POSITIVE_REGULATION_OF_TRANSCRIPTION_I	-0,107	0,003	4,7E-05	0,3863
DELACROIX_RAR_TARGETS_DN	-0,135	2E-04	4,7E-05	0,3866
AFFAR_YY1_TARGETS_DN	-0,173	2E-06	4,7E-05	0,388
MORF_MAGEA8	-0,129	4E-04	4,7E-05	0,3888
SWEET_LUNG_CANCER_KRAS_DN	-0,102	0,005	4,7E-05	0,3901
GSE20715_WT_VS_TLR4_KO_48H_OZONE_L	-0,092	0,012	4,7E-05	0,3906
KEGG_OOCYTE_MEIOSIS	-0,155	2E-05	4,7E-05	0,3908
DNA_RECOMBINATION	-0,15	4E-05	4,7E-05	0,391
GSE17721_LPS_VS_PAM3CSK4_0.5H_BMDM_	-0,134	2E-04	4,7E-05	0,3924
GSE27786_LSK_VS_ERYTHROBLAST_UP	-0,107	0,003	4,8E-05	0,3941

GSE8384_CTRL_VS_B_ABORTUS_4H_MAC_C	-0,133	3E-04	4,8E-05	0,3954
CHEN_HOXA5_TARGETS_6HR_UP	-0,132	3E-04	4,8E-05	0,3998
POST_TRANSLATIONAL_PROTEIN_MODIFICATI	-0,14	1E-04	4,8E-05	0,3999
REGULATION_OF_GROWTH	-0,105	0,004	4,8E-05	0,3999
GSE20366_EX_VIVO_VS_HOMEOSTATIC_CON	-0,131	4E-04	4,8E-05	0,4004
BIOCARTA_INTRINSIC_PATHWAY	-0,13	4E-04	4,8E-05	0,4011
JNK_DN.V1_DN	-0,118	0,001	4,9E-05	0,4034
KIM_WT1_TARGETS_12HR_UP	-0,123	8E-04	4,9E-05	0,404
RAPA_EARLY_UP.V1_DN	-0,128	4E-04	4,9E-05	0,4044
V$USF_02	-0,092	0,012	4,9E-05	0,406
BMI1_DN.V1_DN	-0,083	0,023	4,9E-05	0,4061
PTEN_DN.V2_DN	-0,107	0,004	4,9E-05	0,4063
LASTOWSKA_NEUROBLASTOMA_COPY_NUMB	-0,129	4E-04	4,9E-05	0,4076
GSE14026_TH1_VS_TH17_UP	-0,137	2E-04	5E-05	0,41
PETROVA_PROX1_TARGETS_UP	-0,16	1E-05	5E-05	0,413
MODULE_252	-0,162	9E-06	5E-05	0,4134
chr4q22	-0,101	0,006	5E-05	0,414
RESPONSE_TO_ABIOTIC_STIMULUS	-0,141	1E-04	5E-05	0,416
MORF_PAX7	-0,113	0,002	5,1E-05	0,418
V$CEBP_C	-0,129	4E-04	5,1E-05	0,4187
V$SREBP1_Q6	-0,092	0,012	5,1E-05	0,4209
GSE17721_LPS_VS_POLYIC_6H_BMDM_UP	-0,151	3E-05	5,1E-05	0,4222
GAUSSMANN_MLL_AF4_FUSION_TARGETS_C_	-0,115	0,002	5,1E-05	0,4232
COLLIS_PRKDC_SUBSTRATES	-0,165	6E-06	5,2E-05	0,4257
VEGF_A_UP.V1_DN	-0,18	7E-07	5,2E-05	0,428
COLLAGEN_BINDING	-0,124	7E-04	5,2E-05	0,4283
V$STAT1_02	-0,137	2E-04	5,2E-05	0,4309
MORF_TNFRSF25	-0,104	0,005	5,2E-05	0,4309
GNF2_APEX1	-0,167	4E-06	5,2E-05	0,4313
HU_GENOTOXIN_ACTION_DIRECT_VS_INDIREC	-0,115	0,002	5,2E-05	0,4314
CHROMOSOME	-0,157	2E-05	5,2E-05	0,4325
ST_WNT_BETA_CATENIN_PATHWAY	-0,118	0,001	5,2E-05	0,4328
SNACANNNYSYAGA_UNKNOWN	-0,097	0,008	5,3E-05	0,4336
DAIRKEE_CANCER_PRONE_RESPONSE_E2	-0,116	0,001	5,3E-05	0,4367
GSE22886_NAIVE_TCELL_VS_NEUTROPHIL_U	-0,172	2E-06	5,3E-05	0,4392
MISSIAGLIA_REGULATED_BY_METHYLATION_D	-0,148	5E-05	5,3E-05	0,4392
MORF_TNFRSF6	-0,122	8E-04	5,3E-05	0,4392
WEINMANN_ADAPTATION_TO_HYPOXIA_UP	-0,151	3E-05	5,3E-05	0,4396
JEON_SMAD6_TARGETS_UP	-0,16	1E-05	5,3E-05	0,4405
NEGATIVE_REGULATION_OF_CELL_PROLIFERA	-0,143	9E-05	5,3E-05	0,4407
GSE36476_YOUNG_VS_OLD_DONOR_MEMORY	-0,119	0,001	5,4E-05	0,4418
SNF5_DN.V1_DN	-0,121	9E-04	5,4E-05	0,4423

GSE15659_RESTING_VS_ACTIVATED_TREG_U	-0,112	0,002	5,4E-05	0,443
VALK_AML_CLUSTER_10	-0,044	0,234	5,4E-05	0,4439
MORF_PSMF1	-0,122	8E-04	5,4E-05	0,4455
PHOSPHOPROTEIN_PHOSPHATASE_ACTIVITY	-0,117	0,001	5,4E-05	0,4455
REACTOME_COLLAGEN_FORMATION	-0,145	7E-05	5,4E-05	0,4457
TONKS_TARGETS_OF_RUNX1_RUNX1T1_FUSI	-0,126	6E-04	5,4E-05	0,4472
GSE1448_CTRL_VS_ANTI_VBETA5_DP_THYM	-0,135	2E-04	5,4E-05	0,4476
LEE_DOUBLE_POLAR_THYMOCYTE	-0,097	0,008	5,4E-05	0,4476
NUTT_GBM_VS_AO_GLIOMA_DN	-0,103	0,005	5,4E-05	0,4476
V$CIZ_01	-0,128	5E-04	5,4E-05	0,4484
GSE15659_RESTING_TREG_VS_NONSUPPRESS	-0,156	2E-05	5,5E-05	0,4497
ROVERSI_GLIOMA_LOH_REGIONS	-0,143	9E-05	5,5E-05	0,4497
V$FXR_Q3	-0,1	0,006	5,5E-05	0,4512
DNA_PACKAGING	-0,159	1E-05	5,5E-05	0,4528
EXTRACELLULAR_MATRIX_PART	-0,126	5E-04	5,5E-05	0,4529
ACCAAAG,MIR-9	-0,125	6E-04	5,5E-05	0,4543
GSE360_T_GONDII_VS_B_MALAYI_HIGH_DOS	-0,133	3E-04	5,5E-05	0,4554
HEIDENBLAD_AMPLICON_12P11_12_UP	-0,097	0,008	5,6E-05	0,4564
BAKKER_FOXO3_TARGETS_DN	-0,13	4E-04	5,6E-05	0,4577
MILICIC_FAMILIAL_ADENOMATOUS_POLYPOSIS	-0,116	0,001	5,6E-05	0,4589
GSE27786_LSK_VS_NEUTROPHIL_UP	-0,116	0,002	5,6E-05	0,46
LI_CISPLATIN_RESISTANCE_DN	-0,136	2E-04	5,6E-05	0,4621
TTGCWCAAY_V$CEBPB_02	-0,159	1E-05	5,6E-05	0,4628
PEDERSEN_METASTASIS_BY_ERBB2_ISOFORM	-0,14	1E-04	5,7E-05	0,4657
CHANNEL_REGULATOR_ACTIVITY	-0,091	0,013	5,7E-05	0,4667
KIM_WT1_TARGETS_8HR_UP	-0,146	6E-05	5,7E-05	0,4706
GSE12845_IGD_NEG_BLOOD_VS_PRE_GC_TO	-0,171	3E-06	5,7E-05	0,4708
GSE14350_TREG_VS_TEFF_DN	-0,128	5E-04	5,7E-05	0,4713
GSE2706_R848_VS_LPS_8H_STIM_DC_UP	-0,074	0,044	5,8E-05	0,4731
MODY_HIPPOCAMPUS_PRENATAL	-0,151	3E-05	5,8E-05	0,4743
GSE360_L_MAJOR_VS_B_MALAYI_LOW_DOS	-0,125	6E-04	5,8E-05	0,4752
G_PROTEIN_SIGNALING_COUPLED_TO_IP3_SE	-0,12	0,001	5,8E-05	0,4755
V$SP1_Q6_01	-0,128	4E-04	5,8E-05	0,4771
BYSTRYKH_HEMATOPOIESIS_STEM_CELL_SCP	-0,047	0,195	5,8E-05	0,4775
JIANG_TIP30_TARGETS_UP	-0,16	1E-05	5,8E-05	0,478
BIOCARTA_CELLCYCLE_PATHWAY	-0,162	9E-06	5,9E-05	0,4806
PID_LYSOPHOSPHOLIPID_PATHWAY	-0,107	0,004	5,9E-05	0,4811
GSE360_T_GONDII_VS_M_TUBERCULOSIS_DC	-0,129	4E-04	5,9E-05	0,485
PID_INTEGRIN1_PATHWAY	-0,139	1E-04	5,9E-05	0,4851
GSE11057_NAIVE_VS_CENT_MEMORY_CD4_	-0,102	0,005	5,9E-05	0,4867
MODULE_2	-0,102	0,005	5,9E-05	0,487
BAKER_HEMATOPOIESIS_STAT3_TARGETS	-0,163	8E-06	6E-05	0,4896

REACTOME_EXTRACELLULAR_MATRIX_ORGAN	-0,143	9E-05	6E-05	0,492
MESODERM_DEVELOPMENT	-0,112	0,002	6E-05	0,4923
GCM_FANCC	-0,13	4E-04	6E-05	0,4943
MCCLUNG_CREB1_TARGETS_UP	-0,139	1E-04	6E-05	0,4952
BURTON_ADIPOGENESIS_PEAK_AT_2HR	-0,111	0,002	6,1E-05	0,4992
POSITIVE_REGULATION_OF_CELLULAR_METAI	-0,108	0,003	6,1E-05	0,4992
BILD_SRC_ONCOGENIC_SIGNATURE	-0,142	1E-04	6,1E-05	0,4994
PID_THROMBIN_PAR1_PATHWAY	-0,12	0,001	6,1E-05	0,4995
GSE14350_IL2RB_KO_VS_WT_TEFF_DN	-0,141	1E-04	6,1E-05	0,5032
KEGG_GNRH_SIGNALING_PATHWAY	-0,093	0,011	6,2E-05	0,5037
PYROPHOSPHATASE_ACTIVITY	-0,112	0,002	6,2E-05	0,5045
GSE7852_TREG_VS_TCONV_LN_DN	-0,122	8E-04	6,2E-05	0,5059
V$OCT1_07	-0,112	0,002	6,2E-05	0,5059
KRAS.BREAST_UP.V1_UP	-0,147	6E-05	6,2E-05	0,5085
GCAAAAA,MIR-129	-0,128	5E-04	6,2E-05	0,5102
GSE19825_NAIVE_VS_IL2RALOW_DAY3_EFF	-0,156	2E-05	6,2E-05	0,5105
NIKOLSKY_BREAST_CANCER_17Q21_Q25_AI	-0,11	0,003	6,3E-05	0,5129
GSE17721_POLYIC_VS_GARDIQUIMOD_24H_I	-0,119	0,001	6,3E-05	0,5136
GSE17721_PAM3CSK4_VS_GADIQUIMOD_24I	-0,14	1E-04	6,3E-05	0,5138
CTCAGGG,MIR-125B,MIR-125A	-0,099	0,007	6,3E-05	0,5138
WHITEFORD_PEDIATRIC_CANCER_MARKERS	-0,151	3E-05	6,3E-05	0,5147
GROSS_ELK3_TARGETS_UP	-0,111	0,002	6,3E-05	0,5147
GSE30083_SP2_VS_SP4_THYMOCYTE_UP	-0,116	0,002	6,3E-05	0,515
POSITIVE_REGULATION_OF_CELLULAR_PROCE	-0,131	3E-04	6,3E-05	0,5169
INTERMEDIATE_FILAMENT	-0,155	2E-05	6,3E-05	0,5173
INTERMEDIATE_FILAMENT_CYTOSKELETON	-0,155	2E-05	6,3E-05	0,5173
MORF_CTSB	-0,15	4E-05	6,3E-05	0,5176
AMUNDSON_POOR_SURVIVAL_AFTER_GAMMA	-0,143	9E-05	6,3E-05	0,5177
STANELLE_E2F1_TARGETS	-0,131	3E-04	6,3E-05	0,5184
GSE15324_NAIVE_VS_ACTIVATED_ELF4_KO	-0,087	0,017	6,4E-05	0,5186
LIN_NPAS4_TARGETS_DN	-0,09	0,014	6,4E-05	0,5189
GSE17721_POLYIC_VS_GARDIQUIMOD_1H_BI	-0,15	4E-05	6,4E-05	0,52
MORF_THRA	-0,144	8E-05	6,4E-05	0,5205
SILIGAN_BOUND_BY_EWS_FLT1_FUSION	-0,072	0,05	6,4E-05	0,5223
TTTGTAG,MIR-520D	-0,117	0,001	6,4E-05	0,5227
MORF_RAD51L3	-0,116	0,002	6,4E-05	0,5241
GENTILE_UV_HIGH_DOSE_DN	-0,166	5E-06	6,4E-05	0,5255
GSE17974_CTRL_VS_ACT_IL4_AND_ANTI_IL	-0,099	0,007	6,5E-05	0,5269
V$HNF6_Q6	-0,113	0,002	6,5E-05	0,5274
LEF1_UP.V1_UP	-0,142	1E-04	6,5E-05	0,5275
YGCANTGCR_UNKNOWN	-0,104	0,005	6,5E-05	0,5275
MODULE_183	-0,167	5E-06	6,5E-05	0,529

GSE3982_BCELL_VS_BASOPHIL_UP	-0,126	5E-04	6,5E-05	0,529
GAL_LEUKEMIC_STEM_CELL_UP	-0,114	0,002	6,5E-05	0,5295
REACTOME_PLC_BETA_MEDIATED_EVENTS	-0,11	0,003	6,5E-05	0,5304
WANG_CISPLATIN_RESPONSE_AND_XPC_DN	-0,107	0,003	6,5E-05	0,5324
MITOTIC_SPINDLE_ORGANIZATION_AND_BIOG	-0,159	1E-05	6,5E-05	0,5334
V$COUP_DR1_Q6	-0,083	0,023	6,6E-05	0,5339
TIEN_INTESTINE_PROBIOTICS_6HR_UP	-0,155	2E-05	6,6E-05	0,5343
V$FOXJ2_02	-0,104	0,005	6,6E-05	0,5344
KRAS.600.LUNG.BREAST_UP.V1_DN	-0,128	5E-04	6,6E-05	0,5353
YNGTTNNNATT_UNKNOWN	-0,115	0,002	6,6E-05	0,5384
POSITIVE_REGULATION_OF_METABOLIC_PROC	-0,109	0,003	6,6E-05	0,5385
GSE24142_ADULT_VS_FETAL_DN3_THYMOC	-0,116	0,002	6,6E-05	0,5385
V$EGR_Q6	-0,118	0,001	6,6E-05	0,5388
V$ARP1_01	-0,087	0,017	6,6E-05	0,5391
GLUCOSE_METABOLIC_PROCESS	-0,143	9E-05	6,6E-05	0,5407
BYSTRYKH_HEMATOPOIESIS_STEM_CELL_QTL	-0,109	0,003	6,6E-05	0,5411
BROWNE_HCMV_INFECTION_18HR_DN	-0,1	0,006	6,7E-05	0,5413
GSE3982_CTRL_VS_LPS_48H_DC_UP	-0,097	0,008	6,7E-05	0,5413
V$ZIC3_01	-0,12	0,001	6,7E-05	0,5422
LABBE_TARGETS_OF_TGFB1_AND_WNT3A_U	-0,163	8E-06	6,7E-05	0,5425
BOYAULT_LIVER_CANCER_SUBCLASS_G6_UP	-0,126	6E-04	6,7E-05	0,5425
GSE7852_THYMUS_VS_FAT_TCONV_UP	-0,145	7E-05	6,7E-05	0,5488
GSE20366_EX_VIVO_VS_DEC205_CONVERSI	-0,11	0,003	6,8E-05	0,5504
CARBOHYDRATE_BIOSYNTHETIC_PROCESS	-0,132	3E-04	6,8E-05	0,553
SMID_BREAST_CANCER_RELAPSE_IN_BRAIN_U	-0,159	1E-05	6,8E-05	0,5547
GSE17721_PAM3CSK4_VS_CPG_6H_BMDM_D	-0,134	2E-04	6,8E-05	0,5558
YAO_TEMPORAL_RESPONSE_TO_PROGESTERO	-0,076	0,037	6,9E-05	0,5568
GSE3982_DC_VS_BCELL_DN	-0,128	4E-04	6,9E-05	0,5569
BERTUCCI_MEDULLARY_VS_DUCTAL_BREAST_	-0,113	0,002	6,9E-05	0,5586
KEGG_BIOSYNTHESIS_OF_UNSATURATED_FAT	-0,149	5E-05	6,9E-05	0,5601
GSE15930_STIM_VS_STIM_AND_IL-12_24H_	-0,115	0,002	6,9E-05	0,5611
GSE2826_XID_VS_BTK_KO_BCELL_UP	-0,136	2E-04	6,9E-05	0,5645
ENK_UV_RESPONSE_EPIDERMIS_DN	-0,139	1E-04	7E-05	0,5649
GGGNRMNNYCAT_UNKNOWN	-0,06	0,102	7E-05	0,5651
GSE3982_NEUTROPHIL_VS_BASOPHIL_DN	-0,14	1E-04	7E-05	0,5654
KRAS.600.LUNG.BREAST_UP.V1_UP	-0,125	6E-04	7E-05	0,5676
EPIDERMIS_DEVELOPMENT	-0,165	6E-06	7E-05	0,568
REACTOME_ACTIVATION_OF_NMDA_RECEPTO	-0,111	0,002	7E-05	0,5702
MATZUK_CENTRAL_FOR_FEMALE_FERTILITY	-0,093	0,011	7E-05	0,5702
AAACCAC,MIR-140	-0,147	6E-05	7E-05	0,5707
WNT_UP.V1_UP	-0,101	0,006	7E-05	0,5707
SASAI_RESISTANCE_TO_NEOPLASTIC_TRANSI	-0,115	0,002	7E-05	0,571

PRC2_EZH2_UP.V1_DN	-0,144	8E-05	7E-05	0,5712
GSE15659_RESTING_VS_ACTIVATED_TREG_[	-0,165	6E-06	7E-05	0,5716
SMID_BREAST_CANCER_LUMINAL_A_DN	-0,158	1E-05	7,1E-05	0,5722
BOQUEST_STEM_CELL_UP	-0,091	0,013	7,1E-05	0,5723
CYCLIN_D1_KE_.V1_DN	-0,113	0,002	7,1E-05	0,5732
PID_AJDISS_2PATHWAY	-0,123	7E-04	7,1E-05	0,5738
V$TBP_01	-0,099	0,007	7,1E-05	0,5752
GSE15750_DAY6_VS_DAY10_TRAF6KO_EFF_	-0,071	0,054	7,1E-05	0,5782
MURAKAMI_UV_RESPONSE_6HR_DN	-0,142	1E-04	7,1E-05	0,5794
GSE29618_PRE_VS_DAY7_POST_LAIV_FLU_	-0,102	0,005	7,3E-05	0,5889
REPRODUCTION	-0,118	0,001	7,3E-05	0,5896
PID_SYNDECAN_2_PATHWAY	-0,155	2E-05	7,3E-05	0,5902
KIM_WT1_TARGETS_8HR_DN	-0,123	8E-04	7,3E-05	0,5906
ESC_V6.5_UP_EARLY.V1_DN	-0,125	6E-04	7,3E-05	0,5913
GSE22886_NAIVE_TCELL_VS_DC_UP	-0,102	0,006	7,3E-05	0,5943
GRAESSMANN_APOPTOSIS_BY_DOXORUBICIN_	-0,155	2E-05	7,3E-05	0,5949
GARCIA_TARGETS_OF_FLI1_AND_DAX1_DN	-0,159	1E-05	7,4E-05	0,5976
TRANSMEMBRANE_RECEPTOR_PROTEIN_KINAS	-0,115	0,002	7,4E-05	0,5976
ANATOMICAL_STRUCTURE_MORPHOGENESIS	-0,116	0,001	7,4E-05	0,5988
REACTOME_G_ALPHA_Q_SIGNALLING_EVENT	-0,114	0,002	7,4E-05	0,5988
V$YY1_01	-0,113	0,002	7,4E-05	0,5998
GSE14026_TH1_VS_TH17_DN	-0,131	3E-04	7,4E-05	0,6014
MCBRYAN_TERMINAL_END_BUD_UP	-0,152	3E-05	7,4E-05	0,6023
V$MEF2_Q6_01	-0,116	0,001	7,4E-05	0,6025
KEGG_MELANOMA	-0,143	9E-05	7,5E-05	0,6044
NEUROLOGICAL_SYSTEM_PROCESS	-0,118	0,001	7,5E-05	0,6044
ROSS_AML_WITH_PML_RARA_FUSION	-0,115	0,002	7,5E-05	0,6044
BOYLAN_MULTIPLE_MYELOMA_D_UP	-0,114	0,002	7,5E-05	0,6053
V$EGR2_01	-0,112	0,002	7,5E-05	0,6072
GSE32423_IL7_VS_IL4_MEMORY_CD8_TCELL	-0,15	4E-05	7,5E-05	0,6075
GSE17974_IL4_AND_ANTI_IL12_VS_UNTREA	-0,131	3E-04	7,5E-05	0,6079
CELL_SUBSTRATE_ADHESION	-0,109	0,003	7,5E-05	0,6099
CELL_CELL_SIGNALING	-0,12	0,001	7,6E-05	0,6122
MORF_MT4	-0,096	0,008	7,6E-05	0,6133
PROTEIN_KINASE_ACTIVITY	-0,142	1E-04	7,6E-05	0,6146
GSE10094_LCMV_VS_LISTERIA_IND_EFF_CD4	-0,112	0,002	7,6E-05	0,6149
GSE14308_TH2_VS_NATURAL_TREG_DN	-0,065	0,077	7,6E-05	0,6151
GSE29618_MONOCYTE_VS_PDC_DAY7_FLU_	-0,131	3E-04	7,6E-05	0,6171
GSE17721_CTRL_VS_LPS_12H_BMDM_DN	-0,146	6E-05	7,6E-05	0,6175
WTGAAAT_UNKNOWN	-0,114	0,002	7,7E-05	0,6176
PHONG_TNF_RESPONSE_VIA_P38_PARTIAL	-0,148	5E-05	7,7E-05	0,6182
REACTOME_G_ALPHA_Z_SIGNALLING_EVENTS	-0,105	0,004	7,7E-05	0,6212

RAPA_EARLY_UP.V1_UP	-0,098	0,007	7,7E-05	0,6221
GSE17721_LPS_VS_CPG_1H_BMDM_UP	-0,168	4E-06	7,7E-05	0,6226
REACTOME_NRIF_SIGNALS_CELL_DEATH_FRC	-0,09	0,014	7,8E-05	0,6259
V$FOXD3_01	-0,103	0,005	7,8E-05	0,6302
GTACAGG,MIR-486	-0,126	6E-04	7,8E-05	0,6307
GSE36476_YOUNG_VS_OLD_DONOR_MEMOR	-0,102	0,005	7,8E-05	0,6308
V$MYCMAX_03	-0,138	2E-04	7,8E-05	0,6326
BRIDEAU_IMPRINTED_GENES	-0,118	0,001	7,8E-05	0,6329
GGCAGCT,MIR-22	-0,137	2E-04	7,9E-05	0,633
GSE17721_CTRL_VS_LPS_24H_BMDM_DN	-0,153	3E-05	7,9E-05	0,6347
MODULE_372	-0,165	6E-06	7,9E-05	0,6348
chr1p35	-0,124	7E-04	7,9E-05	0,6375
V$STAT4_01	-0,086	0,019	7,9E-05	0,6385
ZHENG_RESPONSE_TO_ARSENITE_DN	-0,166	5E-06	8E-05	0,641
NOUSHMEHR_GBM_SILENCED_BY_METHYLATI	-0,141	1E-04	8E-05	0,6411
CALVET_IRINOTECAN_SENSITIVE_VS_RESIST	-0,126	5E-04	8E-05	0,6425
MAINA_VHL_TARGETS_UP	-0,13	4E-04	8E-05	0,6426
GSE17721_LPS_VS_GARDIQUIMOD_4H_BMDM	-0,122	9E-04	8E-05	0,6438
HOFMANN_MYELODYSPLASTIC_SYNDROM_LO	-0,137	2E-04	8E-05	0,6445
GSE36392_MAC_VS_NEUTROPHIL_IL25_TRE	-0,053	0,152	8E-05	0,6445
DEPHOSPHORYLATION	-0,098	0,007	8E-05	0,6447
GSE360_L_MAJOR_VS_B_MALAYI_HIGH_DOS	-0,11	0,003	8E-05	0,6462
GSE17721_CTRL_VS_GARDIQUIMOD_0.5H_BI	-0,135	2E-04	8E-05	0,6471
KORKOLA_YOLK_SAC_TUMOR_UP	-0,16	1E-05	8,1E-05	0,6481
V$HMX1_01	-0,086	0,018	8,1E-05	0,6493
GSE29614_CTRL_VS_DAY7_TIV_FLU_VACCI	-0,166	5E-06	8,1E-05	0,65
MODULE_51	-0,146	7E-05	8,1E-05	0,6506
TURJANSKI_MAPK1_AND_MAPK2_TARGETS	-0,067	0,066	8,1E-05	0,6517
SANSOM_APC_TARGETS_REQUIRE_MYC	-0,145	7E-05	8,1E-05	0,6519
NAKAYAMA_SOFT_TISSUE_TUMORS_PCA2_UI	-0,169	3E-06	8,1E-05	0,6523
P53_DN.V1_UP	-0,155	2E-05	8,1E-05	0,6529
GSE9037_WT_VS_IRAK4_KO_LPS_1H_STIM_	-0,121	9E-04	8,2E-05	0,656
KYNG_WERNER_SYNDROM_UP	-0,144	8E-05	8,2E-05	0,6566
GSE37416_CTRL_VS_0H_F_TULARENSIS_LV	-0,098	0,008	8,2E-05	0,6593
GCCATNTTG_V$YY1_Q6	-0,119	0,001	8,2E-05	0,6617
MODULE_279	-0,075	0,041	8,2E-05	0,6626
WARTERS_RESPONSE_TO_IR_SKIN	-0,091	0,013	8,3E-05	0,664
GSE7460_CTRL_VS_TGFB_TREATED_ACT_FC	-0,127	5E-04	8,3E-05	0,6652
SIG_CHEMOTAXIS	-0,155	2E-05	8,3E-05	0,6654
V$CRX_Q4	-0,104	0,004	8,3E-05	0,6676
MITSIADES_RESPONSE_TO_APLIDIN_UP	-0,106	0,004	8,3E-05	0,6677
GSE17721_POLYIC_VS_PAM3CSK4_0.5H_BM	-0,139	1E-04	8,3E-05	0,6684

NUCLEAR_SPECK	-0,055	0,13	8,3E-05	0,6687
GSE7764_IL15_NK_CELL_24H_VS_SPLENOC	-0,162	9E-06	8,3E-05	0,6701
GSE26928_NAIVE_VS_CXCR5_POS_CD4_TCE	-0,08	0,03	8,4E-05	0,6711
ZHENG_IL22_SIGNALING_DN	-0,135	2E-04	8,4E-05	0,6731
VECCHI_GASTRIC_CANCER_EARLY_UP	-0,166	5E-06	8,4E-05	0,6757
MORF_CD8A	-0,111	0,002	8,4E-05	0,6758
ZEMBUTSU_SENSITIVITY_TO_MITOMYCIN	-0,127	5E-04	8,4E-05	0,6764
GSE13411_PLASMA_CELL_VS_MEMORY_BCEI	-0,18	8E-07	8,4E-05	0,6771
SNIJDERS_AMPLIFIED_IN_HEAD_AND_NECK_T	-0,159	1E-05	8,5E-05	0,6784
REACTOME_FACTORS_INVOLVED_IN_MEGAKA	-0,152	3E-05	8,5E-05	0,6785
V$OCT1_06	-0,104	0,005	8,5E-05	0,6791
GSE3982_BCELL_VS_TH1_DN	-0,171	3E-06	8,5E-05	0,6793
CELL_CYCLE_CHECKPOINT_GO_0000075	-0,152	3E-05	8,5E-05	0,6816
HEDENFALK_BREAST_CANCER_BRACX_UP	-0,139	1E-04	8,5E-05	0,6818
PASINI_SUZ12_TARGETS_DN	-0,136	2E-04	8,5E-05	0,684
PID_SYNDECAN_3_PATHWAY	-0,167	4E-06	8,6E-05	0,687
V$LYF1_01	-0,092	0,012	8,6E-05	0,6887
BOYLAN_MULTIPLE_MYELOMA_PCA3_DN	-0,114	0,002	8,6E-05	0,6921
GSE36392_MAC_VS_NEUTROPHIL_IL25_TRE/	-0,14	1E-04	8,6E-05	0,6923
PID_CMYB_PATHWAY	-0,111	0,002	8,7E-05	0,6953
MARTINEZ_RB1_TARGETS_UP	-0,131	3E-04	8,7E-05	0,6991
REACTOME_NUCLEAR_SIGNALING_BY_ERBB4	-0,037	0,312	8,7E-05	0,7003
GSE17974_IL4_AND_ANTI_IL12_VS_UNTREA	-0,104	0,005	8,8E-05	0,7011
MYC_UP.V1_UP	-0,146	6E-05	8,8E-05	0,7031
CAGGGTC,MIR-504	-0,094	0,01	8,8E-05	0,7045
HELLER_HDAC_TARGETS_SILENCED_BY_METH	-0,138	1E-04	8,8E-05	0,7046
GNF2_DENR	-0,169	3E-06	8,8E-05	0,7074
GSE7460_CTRL_VS_TGFB_TREATED_ACT_TC	-0,105	0,004	8,8E-05	0,7074
SCIAN_INVERSED_TARGETS_OF_TP53_AND_T	-0,141	1E-04	8,9E-05	0,7082
GSE27786_CD4_TCELL_VS_NKTCELL_UP	-0,121	9E-04	8,9E-05	0,7088
GSE14308_TH17_VS_NAIVE_CD4_TCELL_DN	-0,112	0,002	8,9E-05	0,7115
BLALOCK_ALZHEIMERS_DISEASE_INCIPIENT_U	-0,121	9E-04	8,9E-05	0,7138
NUCLEAR_CHROMATIN	-0,173	2E-06	9E-05	0,7184
VART_KSHV_INFECTION_ANGIOGENIC_MARKE	-0,129	4E-04	9E-05	0,721
V$ZF5_B	-0,117	0,001	9E-05	0,7218
LIANG_HEMATOPOIESIS_STEM_CELL_NUMBER	-0,15	4E-05	9E-05	0,7225
SMID_BREAST_CANCER_LUMINAL_A_UP	-0,007	0,858	9E-05	0,7226
G1_S_TRANSITION_OF_MITOTIC_CELL_CYCLE	-0,167	5E-06	9,1E-05	0,7245
KEGG_DILATED_CARDIOMYOPATHY	-0,074	0,043	9,1E-05	0,7245
HETEROGENEOUS_NUCLEAR_RIBONUCLEOPRO	-0,119	0,001	9,1E-05	0,7263
STK33_NOMO_DN	-0,139	1E-04	9,1E-05	0,727
GSE17721_LPS_VS_PAM3CSK4_0.5H_BMDM_	-0,124	7E-04	9,1E-05	0,7275

SARRIO_EPITHELIAL_MESENCHYMAL_TRANSIT	-0,134	2E-04	9,1E-05	0,7301
LIU_IL13_PRIMING_MODEL	-0,12	1E-03	9,1E-05	0,7304
HELLER_HDAC_TARGETS_UP	-0,127	5E-04	9,2E-05	0,7325
CATRRAGC_UNKNOWN	-0,113	0,002	9,2E-05	0,7334
OHASHI_AURKA_TARGETS	-0,154	2E-05	9,2E-05	0,7338
GNF2_TM4SF2	-0,127	5E-04	9,2E-05	0,7342
GSE17721_LPS_VS_GARDIQUIMOD_0.5H_BMI	-0,151	3E-05	9,2E-05	0,7343
CENTRAL_NERVOUS_SYSTEM_DEVELOPMENT	-0,127	5E-04	9,2E-05	0,7348
MODULE_332	-0,152	3E-05	9,2E-05	0,7356
STEIN_ESRRA_TARGETS_RESPONSIVE_TO_ES	-0,152	3E-05	9,2E-05	0,7363
MODULE_120	-0,138	2E-04	9,2E-05	0,7364
V$BACH1_01	-0,134	2E-04	9,3E-05	0,7384
TOMLINS_METASTASIS_UP	-0,169	4E-06	9,3E-05	0,7422
MICROTUBULE_MOTOR_ACTIVITY	-0,126	6E-04	9,3E-05	0,7428
MODULE_36	-0,158	2E-05	9,3E-05	0,7429
GENTILE_UV_RESPONSE_CLUSTER_D1	-0,164	7E-06	9,3E-05	0,7432
REGULATION_OF_CATALYTIC_ACTIVITY	-0,132	3E-04	9,3E-05	0,7432
V$STAT1_03	-0,101	0,006	9,3E-05	0,7435
KANG_IMMORTALIZED_BY_TERT_UP	-0,144	8E-05	9,3E-05	0,7439
KIM_WT1_TARGETS_12HR_DN	-0,164	7E-06	9,4E-05	0,7496
LEE_BMP2_TARGETS_UP	-0,073	0,046	9,4E-05	0,7511
GTCTTCC,MIR-7	-0,114	0,002	9,4E-05	0,7518
CERIBELLI_PROMOTERS_INACTIVE_AND_BOUN	-0,165	6E-06	9,4E-05	0,752
REACTOME_TGF_BETA_RECEPTOR_SIGNALING	-0,146	6E-05	9,5E-05	0,7526
GSE15215_CD2_POS_VS_NEG_PDC_UP	-0,102	0,005	9,5E-05	0,7562
CTTTGTA,MIR-524	-0,121	1E-03	9,5E-05	0,7573
MURAKAMI_UV_RESPONSE_1HR_UP	-0,148	5E-05	9,6E-05	0,7613
GSE1460_CD4_THYMOCYTE_VS_NAIVE_CD4_	-0,157	2E-05	9,6E-05	0,7617
GSE360_T_GONDII_VS_B_MALAYI_LOW_DOS	-0,14	1E-04	9,6E-05	0,7623
REACTOME_CGMP_EFFECTS	-0,1	0,006	9,6E-05	0,7651
chr9p21	-0,137	2E-04	9,6E-05	0,7676
GSE22886_NAIVE_BCELL_VS_NEUTROPHIL_L	-0,134	2E-04	9,7E-05	0,7677
NAGASHIMA_NRG1_SIGNALING_UP	-0,114	0,002	9,7E-05	0,7681
TGANNYRGCA_V$TCF11MAFG_01	-0,125	6E-04	9,7E-05	0,7687
REACTOME_TRAFFICKING_OF_AMPA_RECEPT(	-0,075	0,04	9,7E-05	0,7688
GSE9037_WT_VS_IRAK4_KO_BMDM_DN	-0,128	5E-04	9,7E-05	0,7691
LEE_BMP2_TARGETS_DN	-0,179	9E-07	9,7E-05	0,77
MODULE_576	-0,153	3E-05	9,7E-05	0,77
MODULE_99	-0,11	0,003	9,7E-05	0,7711
GSE14350_IL2RB_KO_VS_WT_TEFF_UP	-0,069	0,058	9,7E-05	0,7711
V$MAX_01	-0,142	1E-04	9,7E-05	0,7725
GSE17721_0.5H_VS_24H_PAM3CSK4_BMDM	-0,143	9E-05	9,7E-05	0,7726

QI_HYPOXIA_TARGETS_OF_HIF1A_AND_FOXA	-0,153	3E-05	9,7E-05	0,7732
ACTGTGA,MIR-27A,MIR-27B	-0,107	0,004	9,7E-05	0,7732
MACLACHLAN_BRCA1_TARGETS_DN	-0,143	9E-05	9,8E-05	0,7761
GSE7852_THYMUS_VS_FAT_TREG_UP	-0,095	0,009	9,8E-05	0,7772
REACTOME_G_ALPHA_I_SIGNALLING_EVENTS	-0,136	2E-04	9,8E-05	0,7773
REGULATION_OF_PROTEIN_KINASE_ACTIVITY	-0,132	3E-04	9,8E-05	0,7773
MODULE_3	-0,182	6E-07	9,8E-05	0,7799
BIOPOLYMER_MODIFICATION	-0,14	1E-04	9,9E-05	0,7833
CAGCAGG,MIR-370	-0,134	3E-04	9,9E-05	0,7848
PID_FOXM1PATHWAY	-0,151	4E-05	9,9E-05	0,7868
V$SRY_02	-0,11	0,003	9,9E-05	0,787
FONTAINE_PAPILLARY_THYROID_CARCINOMA_	-0,093	0,011	1E-04	0,7902
GSE29614_CTRL_VS_DAY7_TIV_FLU_VACCII	-0,095	0,009	1E-04	0,7915
OUILLETTE_CLL_13Q14_DELETION_DN	-0,121	1E-03	1E-04	0,7918
ZHENG_FOXP3_TARGETS_IN_T_LYMPHOCYTE_	-0,107	0,003	1E-04	0,7918
MODULE_200	-0,125	6E-04	0,0001	0,7944
RUIZ_TNC_TARGETS_DN	-0,167	5E-06	0,0001	0,8001
CCCAGAG,MIR-326	-0,12	0,001	0,0001	0,8008
V$HNF3B_01	-0,092	0,012	0,0001	0,8028
RESPONSE_TO_UV	-0,163	8E-06	0,0001	0,8042
TOYOTA_TARGETS_OF_MIR34B_AND_MIR34C	-0,152	3E-05	0,0001	0,8042
ACEVEDO_LIVER_CANCER_WITH_H3K27ME3_	-0,133	3E-04	0,0001	0,8042
GSE360_L_MAJOR_VS_B_MALAYI_HIGH_DOS	-0,13	4E-04	0,0001	0,8042
CHARAFE_BREAST_CANCER_LUMINAL_VS_BA	-0,165	6E-06	0,0001	0,8044
chr11p	-0,162	9E-06	0,0001	0,8078
KRAS.LUNG.BREAST_UP.V1_UP	-0,138	1E-04	0,0001	0,8078
KEGG_CALCIUM_SIGNALING_PATHWAY	-0,104	0,005	0,0001	0,8083
HANN_RESISTANCE_TO_BCL2_INHIBITOR_UP	-0,151	4E-05	0,0001	0,8089
REACTOME_SIGNALING_BY_GPCR	-0,12	0,001	0,0001	0,8102
SPECIFIC_RNA_POLYMERASE_II_TRANSCRIPTIC	-0,085	0,02	0,0001	0,8117
HOOI_ST7_TARGETS_UP	-0,126	6E-04	0,0001	0,813
MORF_IL13	-0,105	0,004	0,0001	0,813
MODULE_371	-0,175	2E-06	0,0001	0,8141
GSE26669_CTRL_VS_COSTIM_BLOCK_MLR_C	-0,1	0,006	0,0001	0,8142
SYSTEM_PROCESS	-0,106	0,004	0,0001	0,8173
V$NERF_Q2	-0,103	0,005	0,0001	0,818
chr2p23	-0,169	3E-06	0,0001	0,8186
KAECH_NAIVE_VS_DAY15_EFF_CD8_TCELL_	-0,117	0,001	0,0001	0,8191
FURUKAWA_DUSP6_TARGETS_PCI35_DN	-0,155	2E-05	0,0001	0,8201
STAMBOLSKY_RESPONSE_TO_VITAMIN_D3_U	-0,153	3E-05	0,0001	0,8201
CYTOPLASM	-0,131	3E-04	0,0001	0,8207
GSE29618_PRE_VS_DAY7_POST_LAIV_FLU_	-0,107	0,003	0,0001	0,8228

MORF_PPP2R5B	-0,111	0,002	0,0001	0,8255
BROWNE_HCMV_INFECTION_14HR_UP	-0,155	2E-05	0,0001	0,8257
CYCLIN_D1_UP.V1_DN	-0,109	0,003	0,0001	0,8262
REACTOME_PHOSPHOLIPASE_C_MEDIATED_C/	-0,07	0,055	0,0001	0,8274
GSE17580_UNINFECTED_VS_S_MANSONI_INF	-0,104	0,004	0,0001	0,828
GSE17721_LPS_VS_GARDIQUIMOD_16H_BMD	-0,163	7E-06	0,00011	0,8334
DURCHDEWALD_SKIN_CARCINOGENESIS_UP	-0,14	1E-04	0,00011	0,8334
V$TCF4_Q5	-0,114	0,002	0,00011	0,8336
GSE17721_LPS_VS_CPG_1H_BMDM_DN	-0,143	9E-05	0,00011	0,8342
ABE_VEGFA_TARGETS	-0,142	1E-04	0,00011	0,8368
V$GATA_Q6	-0,101	0,006	0,00011	0,8397
GTGCCAT,MIR-183	-0,136	2E-04	0,00011	0,8403
GSE27786_LSK_VS_MONO_MAC_UP	-0,135	2E-04	0,00011	0,8411
GROSS_HYPOXIA_VIA_HIF1A_UP	-0,159	1E-05	0,00011	0,8426
PID_IL2_PI3KPATHWAY	-0,13	4E-04	0,00011	0,8426
NEGATIVE_REGULATION_OF_GROWTH	-0,089	0,015	0,00011	0,8437
ECTODERM_DEVELOPMENT	-0,165	6E-06	0,00011	0,8452
GSE9650_EXHAUSTED_VS_MEMORY_CD8_TC	-0,125	7E-04	0,00011	0,8468
PID_SHP2_PATHWAY	-0,123	7E-04	0,00011	0,8495
BOYLAN_MULTIPLE_MYELOMA_D_CLUSTER_L	-0,098	0,008	0,00011	0,8511
KINASE_INHIBITOR_ACTIVITY	-0,112	0,002	0,00011	0,8514
V$NFY_Q6	-0,158	2E-05	0,00011	0,8552
KIM_TIAL1_TARGETS	-0,179	9E-07	0,00011	0,8578
GSE27786_CD4_TCELL_VS_NKTCELL_DN	-0,118	0,001	0,00011	0,8588
SIGNAL_TRANSDUCTION	-0,116	0,001	0,00011	0,863
CTIP_DN.V1_DN	-0,097	0,008	0,00011	0,8638
SCHURINGA_STAT5A_TARGETS_UP	-0,116	0,002	0,00011	0,8643
KYNG_ENVIRONMENTAL_STRESS_RESPONSE_	-0,101	0,006	0,00011	0,8679
MODULE_80	-0,146	6E-05	0,00011	0,8685
MODULE_129	-0,112	0,002	0,00011	0,8762
BIOCARTA_ACE2_PATHWAY	-0,124	7E-04	0,00011	0,8764
HUTTMANN_B_CLL_POOR_SURVIVAL_DN	-0,128	5E-04	0,00011	0,8768
BIOCARTA_AMI_PATHWAY	-0,092	0,012	0,00011	0,8769
V$SREBP1_02	-0,143	9E-05	0,00011	0,8789
CTIP_DN.V1_UP	-0,131	3E-04	0,00011	0,8805
GSE18791_UNSTIM_VS_NEWCATSLE_VIRUS_	-0,072	0,049	0,00011	0,8807
MORI_MATURE_B_LYMPHOCYTE_DN	-0,147	6E-05	0,00011	0,8839
REACTOME_FATTY_ACYL_COA_BIOSYNTHESIS	-0,144	8E-05	0,00011	0,8848
BILD_HRAS_ONCOGENIC_SIGNATURE	-0,16	1E-05	0,00011	0,8867
GRAESSMANN_RESPONSE_TO_MC_AND_SERU	-0,156	2E-05	0,00011	0,8869
PARENT_MTOR_SIGNALING_DN	-0,125	6E-04	0,00011	0,8871
REACTOME_DESTABILIZATION_OF_MRNA_BY_	-0,142	1E-04	0,00011	0,8903

XU_GH1_EXOGENOUS_TARGETS_UP	-0,096	0,009	0,00011	0,8903
GSE24634_NAIVE_CD4_TCELL_VS_DAY5_IL4	-0,069	0,061	0,00011	0,8929
CHROMATIN_ASSEMBLY_OR_DISASSEMBLY	-0,162	8E-06	0,00011	0,903
KYNG_RESPONSE_TO_H2O2_VIA_ERCC6_DN	-0,133	3E-04	0,00012	0,9043
RICKMAN_HEAD_AND_NECK_CANCER_B	-0,148	5E-05	0,00012	0,9043
GROWTH_FACTOR_BINDING	-0,093	0,011	0,00012	0,9071
ODONNELL_TFRC_TARGETS_UP	-0,094	0,01	0,00012	0,9128
V$MEF2_02	-0,09	0,014	0,00012	0,9143
SABATES_COLORECTAL_ADENOMA_UP	-0,165	6E-06	0,00012	0,9147
REACTOME_DAG_AND_IP3_SIGNALING	-0,071	0,052	0,00012	0,9152
MODULE_13	-0,109	0,003	0,00012	0,9161
RB_P130_DN.V1_UP	-0,131	3E-04	0,00012	0,9173
ZHU_SKIL_TARGETS_UP	-0,124	7E-04	0,00012	0,9209
YAO_HOXA10_TARGETS_VIA_PROGESTERONE	-0,065	0,075	0,00012	0,9225
MODULE_196	-0,177	1E-06	0,00012	0,9262
SASSON_RESPONSE_TO_FORSKOLIN_DN	-0,134	3E-04	0,00012	0,9269
GSE36392_TYPE_2_MYELOID_VS_MAC_IL25_	-0,106	0,004	0,00012	0,9269
GSE9006_HEALTHY_VS_TYPE_1_DIABETES_I	-0,113	0,002	0,00012	0,9288
GSE360_HIGH_VS_LOW_DOSE_B_MALAYI_DC	-0,126	6E-04	0,00012	0,9296
GSE29618_PRE_VS_DAY7_POST_LAIV_FLU_	-0,146	6E-05	0,00012	0,9316
REGULATION_OF_KINASE_ACTIVITY	-0,132	3E-04	0,00012	0,9329
REACTOME_TRANSCRIPTIONAL_ACTIVITY_OF_	-0,159	1E-05	0,00012	0,9342
V$FREAC4_01	-0,092	0,012	0,00012	0,9351
SEMBA_FHIT_TARGETS_DN	-0,138	2E-04	0,00012	0,9363
MORF_SUPT3H	-0,11	0,003	0,00012	0,9381
ATM_DN.V1_UP	-0,106	0,004	0,00012	0,9404
MAGNESIUM_ION_BINDING	-0,17	3E-06	0,00012	0,9449
TMTCGCGANR_UNKNOWN	-0,161	9E-06	0,00012	0,9473
REGULATION_OF_TRANSFERASE_ACTIVITY	-0,134	2E-04	0,00012	0,9509
ODONNELL_TFRC_TARGETS_DN	-0,154	2E-05	0,00012	0,9516
PID_ENDOTHELINPATHWAY	-0,107	0,003	0,00012	0,9542
REACTOME_SIGNALING_BY_SCF_KIT	-0,135	2E-04	0,00012	0,9545
CHIBA_RESPONSE_TO_TSA_UP	-0,093	0,011	0,00012	0,9546
REACTOME_CELL_CYCLE	-0,154	2E-05	0,00012	0,9554
BIOCARTA_VITCB_PATHWAY	-0,139	1E-04	0,00012	0,9567
MATZUK_EARLY_ANTRAL_FOLLICLE	-0,129	4E-04	0,00012	0,9592
GGCNNMSMYNTTG_UNKNOWN	-0,112	0,002	0,00012	0,9594
NUCLEOSIDE_TRIPHOSPHATASE_ACTIVITY	-0,112	0,002	0,00012	0,9603
BROWNE_HCMV_INFECTION_24HR_DN	-0,112	0,002	0,00012	0,9611
BREDEMEYER_RAG_SIGNALING_NOT_VIA_ATM	-0,101	0,006	0,00012	0,9613
GSE20715_0H_VS_6H_OZONE_TLR4_KO_LU	-0,109	0,003	0,00012	0,9617
GSE24142_EARLY_THYMIC_PROGENITOR_VS_	-0,133	3E-04	0,00012	0,9631

JAEGER_METASTASIS_DN	-0,151	4E-05	0,00012	0,9669
CROONQUIST_STROMAL_STIMULATION_UP	-0,127	5E-04	0,00012	0,9716
V$AHR_Q5	-0,123	7E-04	0,00012	0,973
LEE_RECENT_THYMIC_EMIGRANT	-0,138	2E-04	0,00012	0,9761
GSE36476_CTRL_VS_TSST_ACT_40H_MEMO	-0,09	0,014	0,00013	0,9772
KIM_MYCL1_AMPLIFICATION_TARGETS_DN	-0,177	1E-06	0,00013	0,9779
REACTOME_NEUROTRANSMITTER_RECEPTOR_	-0,116	0,002	0,00013	0,987
GENTLES_LEUKEMIC_STEM_CELL_DN	-0,164	6E-06	0,00013	0,9873
GSE24634_NAIVE_CD4_TCELL_VS_DAY7_IL4	-0,06	0,1	0,00013	0,9888
GSE24142_DN2_VS_DN3_THYMOCYTE_ADUL	-0,133	3E-04	0,00013	0,993
KYNG_RESPONSE_TO_H2O2_VIA_ERCC6	-0,121	9E-04	0,00013	0,9951
GUENTHER_GROWTH_SPHERICAL_VS_ADHERE	-0,151	3E-05	0,00013	0,9972
CSR_LATE_UP.V1_DN	-0,093	0,011	0,00013	0,9992
GSE7764_IL15_TREATED_VS_CTRL_NK_CELI	-0,079	0,032	0,00013	0,9992
GGAMTNNNNNTCCY_UNKNOWN	-0,107	0,003	0,00013	0,9994
MULLIGAN_NTF3_SIGNALING_VIA_INSR_AND_	-0,18	8E-07	0,00023	1
NADELLA_PRKAR1A_TARGETS_UP	-0,179	9E-07	0,00164	1
LANDIS_ERBB2_BREAST_PRENEOPLASTIC_UP	-0,179	9E-07	0,00038	1
OXIDOREDUCTASE_ACTIVITY_GO_0016706	-0,177	1E-06	0,00037	1
KARAKAS_TGFB1_SIGNALING	-0,176	1E-06	0,0003	1
BACOLOD_RESISTANCE_TO_ALKYLATING_AG	-0,176	1E-06	0,00032	1
KARLSSON_TGFB1_TARGETS_UP	-0,175	2E-06	0,00133	1
FLECHNER_PBL_KIDNEY_TRANSPLANT_REJEC	-0,172	2E-06	0,00017	1
LANDIS_ERBB2_BREAST_TUMORS_324_UP	-0,172	2E-06	0,00023	1
LI_LUNG_CANCER	-0,171	3E-06	0,00025	1
REACTOME_TRANSCRIPTION	-0,171	3E-06	0,00071	1
PID_CDC42_PATHWAY	-0,171	3E-06	0,00035	1
WIERENGA_PML_INTERACTOME	-0,17	3E-06	0,00053	1
MORF_BMI1	-0,169	3E-06	0,00108	1
YIH_RESPONSE_TO_ARSENITE_C3	-0,169	3E-06	0,00019	1
MODULE_253	-0,169	4E-06	0,00031	1
RICKMAN_HEAD_AND_NECK_CANCER_C	-0,169	4E-06	0,00014	1
MODULE_421	-0,168	4E-06	0,00137	1
RICKMAN_TUMOR_DIFFERENTIATED_WELL_VS	-0,168	4E-06	0,00026	1
BENPORATH_MYC_MAX_TARGETS	-0,168	4E-06	0,00027	1
GSE17721_POLYIC_VS_GARDIQUIMOD_12H_E	-0,167	4E-06	0,00015	1
JAZAG_TGFB1_SIGNALING_UP	-0,166	5E-06	0,00028	1
NUCLEOLUS	-0,166	5E-06	0,00017	1
GSE20715_0H_VS_48H_OZONE_TLR4_KO_L	-0,166	5E-06	0,00104	1
KAYO_AGING_MUSCLE_DN	-0,166	5E-06	0,00019	1
PHESSE_TARGETS_OF_APC_AND_MBD2_DN	-0,166	5E-06	0,00043	1
MODULE_264	-0,166	5E-06	0,00041	1

WANG_RESPONSE_TO_PACLITAXEL_VIA_MAF	-0,166	5E-06	0,00013	1
RNA_SPLICING	-0,166	6E-06	0,00013	1
KEGG_PATHOGENIC_ESCHERICHIA_COLI_INFE(	-0,166	6E-06	0,00019	1
GSE17974_CTRL_VS_ACT_IL4_AND_ANTI_IL	-0,165	6E-06	0,00115	1
BLALOCK_ALZHEIMERS_DISEASE_INCIPIENT_D	-0,165	6E-06	0,00017	1
REACTOME_MRNA_3_END_PROCESSING	-0,165	6E-06	0,00091	1
ONDER_CDH1_TARGETS_2_DN	-0,165	6E-06	0,00017	1
GSE1460_INTRATHYMIC_T_PROGENITOR_VS_	-0,165	6E-06	0,00022	1
GSE13493_DP_VS_CD4INTCD8POS_THYMOC\	-0,165	6E-06	0,00032	1
HUMMEL_BURKITTS_LYMPHOMA_UP	-0,165	6E-06	0,00017	1
CHROMATIN_REMODELING	-0,164	7E-06	0,00064	1
KEGG_ADHERENS_JUNCTION	-0,164	7E-06	0,00016	1
VANTVEER_BREAST_CANCER_BRCA1_UP	-0,164	7E-06	0,00025	1
GNF2_RPA1	-0,164	7E-06	0,00035	1
SMID_BREAST_CANCER_RELAPSE_IN_LUNG_L	-0,163	7E-06	0,00037	1
GSE12366_GC_BCELL_VS_PLASMA_CELL_UF	-0,163	8E-06	0,00013	1
GNF2_NPM1	-0,163	8E-06	0,00017	1
WANG_SMARCE1_TARGETS_DN	-0,163	8E-06	0,00074	1
RHEIN_ALL_GLUCOCORTICOID_THERAPY_DN	-0,162	8E-06	0,00014	1
PID_DNAPK_PATHWAY	-0,162	8E-06	0,00016	1
REACTOME_AMINO_ACID_AND_OLIGOPEPTIDE	-0,162	9E-06	0,00018	1
PASINI_SUZ12_TARGETS_UP	-0,162	9E-06	0,00015	1
DORMOY_ELAVL1_TARGETS	-0,162	9E-06	0,00018	1
SANA_RESPONSE_TO_IFNG_DN	-0,162	9E-06	0,00159	1
TUBULIN_BINDING	-0,162	9E-06	0,0002	1
KAPOSI_LIVER_CANCER_MET_UP	-0,161	1E-05	0,00109	1
GSE17721_POLYIC_VS_GARDIQUIMOD_24H_E	-0,161	1E-05	0,00016	1
BRUECKNER_TARGETS_OF_MIRLET7A3_DN	-0,161	1E-05	0,00015	1
GSE25087_FETAL_VS_ADULT_TCONV_UP	-0,161	1E-05	0,00202	1
DODD_NASOPHARYNGEAL_CARCINOMA_DN	-0,161	1E-05	0,00027	1
GSE31082_DN_VS_CD8_SP_THYMOCYTE_UP	-0,161	1E-05	0,00037	1
SESTO_RESPONSE_TO_UV_C7	-0,16	1E-05	0,00146	1
GSE14308_TH17_VS_NAIVE_CD4_TCELL_UP	-0,16	1E-05	0,00033	1
LIAO_HAVE_SOX4_BINDING_SITES	-0,16	1E-05	0,00093	1
REACTOME_APOPTOSIS	-0,16	1E-05	0,00242	1
GCNP_SHH_UP_EARLY.V1_UP	-0,16	1E-05	0,00016	1
MTOR_UP.V1_UP	-0,16	1E-05	0,00069	1
JOHANSSON_GLIOMAGENESIS_BY_PDGFB_UP	-0,159	1E-05	0,00028	1
CHANG_CORE_SERUM_RESPONSE_UP	-0,159	1E-05	0,00034	1
RNA_POLYMERASE_II_TRANSCRIPTION_MEDIA	-0,159	1E-05	0,00067	1
REACTOME_APOPTOTIC_CLEAVAGE_OF_CELL	-0,159	1E-05	0,00169	1
STONER_ESOPHAGEAL_CARCINOGENESIS_DN	-0,159	1E-05	0,00018	1

BILANGES_SERUM_SENSITIVE_VIA_TSC2	-0,159	1E-05	0,00013	1
SERVITJA_LIVER_HNF1A_TARGETS_UP	-0,159	1E-05	0,00019	1
PUIFFE_INVASION_INHIBITED_BY_ASCITES_DN	-0,158	1E-05	0,00034	1
KOKKINAKIS_METHIONINE_DEPRIVATION_96H	-0,158	1E-05	0,00022	1
MORF_CDC16	-0,158	1E-05	0,00098	1
PID_ECADHERIN_KERATINOCYTE_PATHWAY	-0,158	1E-05	0,00389	1
PUJANA_CHEK2_PCC_NETWORK	-0,158	1E-05	0,00013	1
STONER_ESOPHAGEAL_CARCINOGENESIS_UP	-0,158	1E-05	0,00157	1
GSE3982_EFF_MEMORY_CD4_TCELL_VS_TH2	-0,158	1E-05	0,00042	1
NUCLEAR_CHROMOSOME_PART	-0,158	1E-05	0,0002	1
KOINUMA_TARGETS_OF_SMAD2_OR_SMAD3	-0,158	1E-05	0,00015	1
GSE24634_TEFF_VS_TCONV_DAY3_IN_CULT	-0,158	1E-05	0,00013	1
ST_PHOSPHOINOSITIDE_3_KINASE_PATHWAY	-0,158	1E-05	0,00054	1
GCM_CBFB	-0,158	2E-05	0,00016	1
YAMASHITA_METHYLATED_IN_PROSTATE_CA	-0,158	2E-05	0,0002	1
REACTOME_MEIOSIS	-0,158	2E-05	0,0013	1
ZHAN_VARIABLE_EARLY_DIFFERENTIATION_G	-0,158	2E-05	0,00054	1
MICROTUBULE_CYTOSKELETON_ORGANIZATIO	-0,158	2E-05	0,00015	1
LEI_MYB_TARGETS	-0,157	2E-05	0,0005	1
MORF_PPP1CC	-0,157	2E-05	0,00028	1
FERRANDO_HOX11_NEIGHBORS	-0,157	2E-05	0,00047	1
HU_ANGIOGENESIS_DN	-0,157	2E-05	0,00194	1
GSE22886_UNSTIM_VS_IL2_STIM_NKCELL_D	-0,157	2E-05	0,0002	1
chr13q11	-0,157	2E-05	0,00015	1
MODULE_39	-0,157	2E-05	0,00068	1
PORE_COMPLEX	-0,157	2E-05	0,00095	1
MICROTUBULE_BASED_PROCESS	-0,157	2E-05	0,00024	1
GSE10239_NAIVE_VS_KLRG1HIGH_EFF_CD8_	-0,157	2E-05	0,00031	1
RNA_SPLICINGVIA_TRANSESTERIFICATION_RE	-0,156	2E-05	0,00055	1
HINATA_NFKB_TARGETS_KERATINOCYTE_DN	-0,156	2E-05	0,00063	1
MUELLER_METHYLATED_IN_GLIOBLASTOMA	-0,156	2E-05	0,00028	1
KRIGE_RESPONSE_TO_TOSEDOSTAT_24HR_D	-0,156	2E-05	0,00022	1
BIOCARTA_ARF_PATHWAY	-0,156	2E-05	0,00017	1
HORIUCHI_WTAP_TARGETS_DN	-0,156	2E-05	0,00014	1
ROYLANCE_BREAST_CANCER_16Q_COPY_NU	-0,156	2E-05	0,00661	1
HASLINGER_B_CLL_WITH_CHROMOSOME_12_	-0,156	2E-05	0,00027	1
BURTON_ADIPOGENESIS_2	-0,156	2E-05	0,00061	1
CHROMATIN_BINDING	-0,156	2E-05	0,00028	1
MODULE_395	-0,156	2E-05	0,00472	1
HESS_TARGETS_OF_HOXA9_AND_MEIS1_UP	-0,155	2E-05	0,00018	1
KINSEY_TARGETS_OF_EWSR1_FLII_FUSION_U	-0,155	2E-05	0,00051	1
MORF_DNMT1	-0,155	2E-05	0,00019	1

BIOCARTA_CDC42RAC_PATHWAY	-0,155	2E-05	0,0023	1
GNF2_ELAC2	-0,155	2E-05	0,00043	1
GSE30962_ACUTE_VS_CHRONIC_LCMV_PRIM	-0,155	2E-05	0,00054	1
ZUCCHI_METASTASIS_UP	-0,155	2E-05	0,00021	1
GSE17974_CTRL_VS_ACT_IL4_AND_ANTI_IL	-0,154	2E-05	0,00076	1
WAESCH_ANAPHASE_PROMOTING_COMPLEX	-0,154	2E-05	0,01468	1
GSE17721_CPG_VS_GARDIQUIMOD_1H_BMDI	-0,154	2E-05	0,00028	1
GNF2_KPNB1	-0,154	2E-05	0,00163	1
V$ALX4_01	-0,154	2E-05	0,00022	1
WANG_RESPONSE_TO_GSK3_INHIBITOR_SB21	-0,154	2E-05	0,00036	1
REACTOME_CHROMOSOME_MAINTENANCE	-0,154	2E-05	0,00113	1
CAIRO_HEPATOBLASTOMA_CLASSES_UP	-0,154	3E-05	0,00018	1
LY_AGING_OLD_DN	-0,154	3E-05	0,00019	1
BIOCARTA_RHO_PATHWAY	-0,154	3E-05	0,00016	1
GSE3982_EOSINOPHIL_VS_MAST_CELL_DN	-0,154	3E-05	0,00077	1
GSE3982_MEMORY_CD4_TCELL_VS_TH2_DN	-0,154	3E-05	0,00054	1
GSE3982_NKCELL_VS_TH2_DN	-0,154	3E-05	0,00034	1
GCM_PPP1CC	-0,153	3E-05	0,00095	1
GSE10239_NAIVE_VS_DAY4.5_EFF_CD8_TCI	-0,153	3E-05	0,00016	1
ROESSLER_LIVER_CANCER_METASTASIS_DN	-0,153	3E-05	0,00026	1
JACKSON_DNMT1_TARGETS_UP	-0,153	3E-05	0,00026	1
MYLLYKANGAS_AMPLIFICATION_HOT_SPOT_1	-0,153	3E-05	0,00123	1
NAKAMURA_METASTASIS_MODEL_UP	-0,153	3E-05	0,00088	1
MODULE_53	-0,153	3E-05	0,00025	1
JACKSON_DNMT1_TARGETS_DN	-0,153	3E-05	0,00026	1
V$CETS1P54_01	-0,153	3E-05	0,00016	1
chr2p16	-0,152	3E-05	0,00174	1
TESAR_ALK_AND_JAK_TARGETS_MOUSE_ES_	-0,152	3E-05	0,00104	1
GSE13306_TREG_VS_TCONV_DN	-0,152	3E-05	0,00026	1
GSE3982_BASOPHIL_VS_TH1_DN	-0,152	3E-05	0,00159	1
YAO_TEMPORAL_RESPONSE_TO_PROGESTERC	-0,152	3E-05	0,00244	1
VANTVEER_BREAST_CANCER_ESR1_DN	-0,152	3E-05	0,00063	1
GSE25087_FETAL_VS_ADULT_TREG_UP	-0,152	3E-05	0,00152	1
MICROBODY_MEMBRANE	0,152	3E-05	0,00035	1
PEROXISOMAL_MEMBRANE	0,152	3E-05	0,00035	1
DANG_MYC_TARGETS_UP	-0,152	3E-05	0,00064	1
SPINDLE_ORGANIZATION_AND_BIOGENESIS	-0,152	3E-05	0,00019	1
GSE17721_CTRL_VS_GARDIQUIMOD_6H_BMD	-0,152	3E-05	0,00058	1
CHROMOSOMAL_PART	-0,152	3E-05	0,00015	1
REACTOME_AMINO_ACID_TRANSPORT_ACROS	-0,151	3E-05	0,00049	1
GSE12845_IGD_POS_BLOOD_VS_PRE_GC_TC	-0,151	3E-05	0,00025	1
MORF_TERF1	-0,151	4E-05	0,00069	1

LIANG_SILENCED_BY_METHYLATION_UP	-0,151	4E-05	0,00016	1
MCMURRAY_TP53_HRAS_COOPERATION_RESP	-0,151	4E-05	0,00072	1
TRANSCRIPTION_FACTOR_COMPLEX	-0,151	4E-05	0,00047	1
REACTOME_INTEGRATION_OF_PROVIRUS	-0,151	4E-05	0,00111	1
MODULE_392	-0,151	4E-05	0,00051	1
GSE16522_ANTI_CD3CD28_STIM_VS_UNSTIM	-0,151	4E-05	0,00055	1
HE_PTEN_TARGETS_UP	-0,151	4E-05	0,0002	1
SUNG_METASTASIS_STROMA_DN	-0,15	4E-05	0,00128	1
TESAR_ALK_TARGETS_EPISC_4D_UP	-0,15	4E-05	0,00191	1
CHARAFE_BREAST_CANCER_LUMINAL_VS_ME	-0,15	4E-05	0,00026	1
FIGUEROA_AML_METHYLATION_CLUSTER_5_U	-0,15	4E-05	0,00087	1
MONNIER_POSTRADIATION_TUMOR_ESCAPE_U	-0,15	4E-05	0,0011	1
MODULE_153	-0,15	4E-05	0,00145	1
ZAMORA_NOS2_TARGETS_UP	-0,15	4E-05	0,00073	1
REACTOME_ZINC_TRANSPORTERS	0,15	4E-05	0,00016	1
INTEGRAL_TO_ORGANELLE_MEMBRANE	0,15	4E-05	0,00035	1
BIOCARTA_SKP2E2F_PATHWAY	-0,15	4E-05	0,00108	1
REACTOME_DEADENYLATION_DEPENDENT_MR	-0,15	4E-05	0,00118	1
GCM_AQP4	-0,15	4E-05	0,00014	1
MORF_ACP1	-0,15	4E-05	0,00102	1
chr15q25	-0,149	4E-05	0,00057	1
chr6p22	-0,149	4E-05	0,00591	1
GSE17974_0H_VS_6H_IN_VITRO_ACT_CD4_	-0,149	4E-05	0,00458	1
GSE17721_LPS_VS_CPG_4H_BMDM_DN	-0,149	4E-05	0,0069	1
HUPER_BREAST_BASAL_VS_LUMINAL_UP	-0,149	4E-05	0,00368	1
SENGUPTA_NASOPHARYNGEAL_CARCINOMA_	-0,149	4E-05	0,00041	1
CALMODULIN_BINDING	-0,149	4E-05	0,00019	1
TRANSCRIPTION_INITIATION_FROM_RNA_POLY	-0,149	4E-05	0,00178	1
MODULE_453	-0,149	5E-05	0,0005	1
WU_APOPTOSIS_BY_CDKN1A_VIA_TP53	-0,149	5E-05	0,00019	1
REACTOME_METABOLISM_OF_PROTEINS	-0,149	5E-05	0,00044	1
PID_MTOR_4PATHWAY	-0,149	5E-05	0,00204	1
VERNELL_RETINOBLASTOMA_PATHWAY_UP	-0,149	5E-05	0,00021	1
GENERAL_RNA_POLYMERASE_II_TRANSCRIPTI	-0,149	5E-05	0,00362	1
SMITH_TERT_TARGETS_UP	-0,149	5E-05	0,00633	1
chr1p32	-0,149	5E-05	0,00013	1
GLI1_UP.V1_DN	-0,149	5E-05	0,00346	1
SCIBETTA_KDM5B_TARGETS_DN	-0,149	5E-05	0,00093	1
OSADA_ASCL1_TARGETS_DN	-0,148	5E-05	0,00035	1
GSE15324_NAIVE_VS_ACTIVATED_ELF4_KO_	-0,148	5E-05	0,00069	1
BURTON_ADIPOGENESIS_PEAK_AT_24HR	-0,148	5E-05	0,00088	1
GNF2_TDG	-0,148	5E-05	0,00118	1

MODULE_503	-0,148	5E-05	0,00017	1
PID_SYNDECAN_1_PATHWAY	-0,148	5E-05	0,00013	1
GSE1460_CD4_THYMOCYTE_VS_THYMIC_STI	-0,148	5E-05	0,00021	1
G2_M_TRANSITION_OF_MITOTIC_CELL_CYCLI	-0,148	5E-05	0,00039	1
BIERIE_INFLAMMATORY_RESPONSE_TGFB1	-0,148	5E-05	0,00099	1
MODULE_126	-0,148	5E-05	0,00094	1
GGCACTT,MIR-519E	-0,148	5E-05	0,00055	1
GSE15930_STIM_VS_STIM_AND_IL-12_24H_	-0,148	5E-05	0,00034	1
REACTOME_CLEAVAGE_OF_GROWING_TRANS	-0,148	5E-05	0,00192	1
LINSLEY_MIR16_TARGETS	-0,148	5E-05	0,00017	1
STEROID_BIOSYNTHETIC_PROCESS	-0,148	5E-05	0,00226	1
KANG_FLUOROURACIL_RESISTANCE_DN	-0,148	5E-05	0,00177	1
KEGG_CYSTEINE_AND_METHIONINE_METABOL	-0,147	5E-05	0,00089	1
PENG_GLUTAMINE_DEPRIVATION_DN	-0,147	5E-05	0,0072	1
XU_HGF_SIGNALING_NOT_VIA_AKT1_48HR_I	-0,147	5E-05	0,00072	1
BIOCARTA_RANMS_PATHWAY	-0,147	5E-05	0,00023	1
GSE1460_NAIVE_CD4_TCELL_CORD_BLOOD_	-0,147	6E-05	0,00451	1
AMIT_EGF_RESPONSE_480_HELA	-0,147	6E-05	0,00089	1
LEI_HOXC8_TARGETS_DN	-0,147	6E-05	0,00025	1
GSE29615_CTRL_VS_DAY3_LAIV_IFLU_VAC(	-0,147	6E-05	0,00125	1
ZHANG_BREAST_CANCER_PROGENITORS_UP	-0,147	6E-05	0,00145	1
INTRINSIC_TO_ORGANELLE_MEMBRANE	0,147	6E-05	0,00059	1
MATZUK_SPERMATOCYTE	-0,147	6E-05	0,00028	1
MORI_PRE_BI_LYMPHOCYTE_UP	-0,147	6E-05	0,00014	1
GSE29617_CTRL_VS_DAY7_TIV_FLU_VACCII	-0,147	6E-05	0,00311	1
BRACHAT_RESPONSE_TO_CAMPTOTHECIN_DI	-0,147	6E-05	0,00205	1
ASTIER_INTEGRIN_SIGNALING	-0,147	6E-05	0,00069	1
GSE17721_0.5H_VS_24H_POLYIC_BMDM_UP	-0,147	6E-05	0,00084	1
GNF2_DEK	-0,147	6E-05	0,00107	1
REGULATION_OF_JAK_STAT_CASCADE	-0,147	6E-05	0,0002	1
NEGATIVE_REGULATION_OF_TRANSPORT	-0,147	6E-05	0,00032	1
GSE12845_NAIVE_VS_DARKZONE_GC_TONSI	-0,147	6E-05	0,0046	1
MODULE_315	-0,147	6E-05	0,00075	1
GSE17974_0H_VS_24H_IN_VITRO_ACT_CD4	-0,147	6E-05	0,00062	1
WONG_EMBRYONIC_STEM_CELL_CORE	-0,147	6E-05	0,00055	1
FUJII_YBX1_TARGETS_DN	-0,146	6E-05	0,00034	1
ONDER_CDH1_TARGETS_1_DN	-0,146	6E-05	0,01263	1
GSE17721_LPS_VS_CPG_6H_BMDM_DN	-0,146	6E-05	0,00068	1
GSE17721_CTRL_VS_LPS_0.5H_BMDM_UP	-0,146	6E-05	0,0003	1
BURTON_ADIPOGENESIS_PEAK_AT_8HR	-0,146	6E-05	0,00078	1
DANG_REGULATED_BY_MYC_UP	-0,146	6E-05	0,00091	1
WEINMANN_ADAPTATION_TO_HYPOXIA_DN	-0,146	6E-05	0,00025	1

BIOCARTA_SRCRPTP_PATHWAY	-0,146	6E-05	0,00032	1
CYTOKINESIS	-0,146	6E-05	0,00037	1
RAO_BOUND_BY_SALL4	-0,146	6E-05	0,00016	1
SARRIO_EPITHELIAL_MESENCHYMAL_TRANSIT	-0,146	6E-05	0,00038	1
REACTOME_RNA_POL_III_TRANSCRIPTION_TEI	-0,146	6E-05	0,00158	1
REGULATION_OF_TYROSINE_PHOSPHORYLATI(	-0,146	6E-05	0,00018	1
GSE14769_20MIN_VS_360MIN_LPS_BMDM_U	-0,146	6E-05	0,00059	1
GSE11864_CSF1_IFNG_VS_CSF1_PAM3CYS_	-0,146	7E-05	0,00084	1
AZARE_STAT3_TARGETS	-0,146	7E-05	0,00073	1
GSE29614_DAY3_VS_DAY7_TIV_FLU_VACCI	-0,145	7E-05	0,00298	1
WILLIAMS_ESR2_TARGETS_DN	-0,145	7E-05	0,00063	1
GSE24634_TEFF_VS_TCONV_DAY5_IN_CULT	-0,145	7E-05	0,00057	1
NUTT_GBM_VS_AO_GLIOMA_UP	-0,145	7E-05	0,00052	1
PUJANA_XPRSS_INT_NETWORK	-0,145	7E-05	0,00019	1
GSE24634_TEFF_VS_TCONV_DAY7_IN_CULT	-0,145	7E-05	0,00023	1
BURTON_ADIPOGENESIS_3	-0,145	7E-05	0,0002	1
REACTOME_PROCESSING_OF_CAPPED_INTRON	-0,145	7E-05	0,00118	1
KEGG_PANCREATIC_CANCER	-0,145	7E-05	0,00036	1
LI_WILMS_TUMOR_ANAPLASTIC_UP	-0,145	7E-05	0,00023	1
REACTOME_MEIOTIC_RECOMBINATION	-0,145	7E-05	0,00433	1
MICROTUBULE_ORGANIZING_CENTER	-0,145	7E-05	0,00025	1
XU_HGF_TARGETS_REPRESSED_BY_AKT1_UP	-0,145	7E-05	0,0006	1
TYROSINE_PHOSPHORYLATION_OF_STAT_PRC	-0,145	7E-05	0,00019	1
RAF_UP.V1_UP	-0,145	7E-05	0,0011	1
LY_AGING_MIDDLE_DN	-0,145	7E-05	0,00016	1
MODULE_127	-0,145	7E-05	0,00675	1
XU_RESPONSE_TO_TRETINOIN_AND_NSC682!	-0,145	7E-05	0,00667	1
MODULE_303	-0,145	7E-05	0,00036	1
NUCLEOTIDYLTRANSFERASE_ACTIVITY	-0,145	7E-05	0,0008	1
KEGG_REGULATION_OF_ACTIN_CYTOSKELETC	-0,145	7E-05	0,00014	1
PROTEIN_AMINO_ACID_LIPIDATION	0,145	7E-05	0,00123	1
CHROMOSOME_CONDENSATION	-0,145	7E-05	0,00031	1
V$TCF11MAFG_01	-0,145	7E-05	0,00015	1
PATIL_LIVER_CANCER	-0,145	7E-05	0,00068	1
ZHOU_TNF_SIGNALING_4HR	-0,145	8E-05	0,00187	1
GSE7460_CD8_TCELL_VS_TREG_ACT_DN	-0,144	8E-05	0,00194	1
RASHI_RESPONSE_TO_IONIZING_RADIATION_4	-0,144	8E-05	0,00015	1
MODULE_32	-0,144	8E-05	0,00048	1
REACTOME_RNA_POL_I_RNA_POL_III_AND_M	-0,144	8E-05	0,00438	1
SHEDDEN_LUNG_CANCER_GOOD_SURVIVAL_/	-0,144	8E-05	0,00016	1
GRAHAM_CML_DIVIDING_VS_NORMAL_QUIES(	-0,144	8E-05	0,00017	1
CHIANG_LIVER_CANCER_SUBCLASS_PROLIFEF	-0,144	8E-05	0,00075	1

GSE15659_NAIVE_CD4_TCELL_VS_NONSUPF	-0,144	8E-05	0,00039	1
MORF_XRCC5	-0,144	8E-05	0,00284	1
REGULATION_OF_CELL_CELL_ADHESION	-0,144	8E-05	0,00171	1
TESAR_ALK_TARGETS_EPISC_3D_UP	-0,144	8E-05	0,00364	1
MORF_PRKDC	-0,144	8E-05	0,00276	1
DASU_IL6_SIGNALING_UP	-0,144	8E-05	0,00039	1
SHEDDEN_LUNG_CANCER_POOR_SURVIVAL_/	-0,144	8E-05	0,00053	1
GSE24634_TREG_VS_TCONV_POST_DAY10_	-0,144	8E-05	0,00035	1
V$NFY_01	-0,144	8E-05	0,00143	1
WILLIAMS_ESR2_TARGETS_UP	0,144	8E-05	0,00089	1
CROMER_TUMORIGENESIS_UP	-0,144	8E-05	0,00304	1
GAJATE_RESPONSE_TO_TRABECTEDIN_DN	-0,143	9E-05	0,00216	1
GSE24634_TREG_VS_TCONV_POST_DAY7_IL	-0,143	9E-05	0,00022	1
LEE_LIVER_CANCER_MYC_UP	-0,143	9E-05	0,00047	1
MODULE_321	-0,143	9E-05	0,005	1
GENTILE_UV_RESPONSE_CLUSTER_D8	-0,143	9E-05	0,00036	1
PEDERSEN_TARGETS_OF_611CTF_ISOFORM_(	-0,143	9E-05	0,00202	1
GYORFFY_DOXORUBICIN_RESISTANCE	-0,143	9E-05	0,00021	1
GSE9650_NAIVE_VS_EXHAUSTED_CD8_TCEL	-0,143	9E-05	0,00017	1
REACTOME_MRNA_PROCESSING	-0,143	9E-05	0,00106	1
GSE17974_0H_VS_4H_IN_VITRO_ACT_CD4_	-0,143	9E-05	0,00255	1
LEE_INTRATHYMIC_T_PROGENITOR	-0,143	9E-05	0,00014	1
GSE22886_NEUTROPHIL_VS_MONOCYTE_UP	-0,143	9E-05	0,00018	1
GSE3982_MAST_CELL_VS_TH1_DN	-0,143	9E-05	0,00025	1
REACTOME_FORMATION_OF_TUBULIN_FOLDIN	-0,143	9E-05	0,00358	1
PEROXISOME_ORGANIZATION_AND_BIOGENES	0,143	9E-05	0,001	1
FLECHNER_PBL_KIDNEY_TRANSPLANT_OK_V:	-0,143	9E-05	0,00026	1
MODULE_281	-0,143	9E-05	0,00214	1
WANG_RECURRENT_LIVER_CANCER_UP	-0,142	1E-04	0,00087	1
POS_HISTAMINE_RESPONSE_NETWORK	-0,142	1E-04	0,00088	1
WHITFIELD_CELL_CYCLE_S	-0,142	1E-04	0,00056	1
CHAUHAN_RESPONSE_TO_METHOXYESTRADI(	-0,142	1E-04	0,00205	1
TONKS_TARGETS_OF_RUNX1_RUNX1T1_FUSI	-0,142	1E-04	0,0043	1
ENK_UV_RESPONSE_EPIDERMIS_UP	-0,142	1E-04	0,00865	1
BROWN_MYELOID_CELL_DEVELOPMENT_DN	-0,142	1E-04	0,00084	1
GCM_APEX1	-0,142	1E-04	0,00079	1
GSE17974_0.5H_VS_72H_IL4_AND_ANTI_IL	-0,142	1E-04	0,00144	1
SA_REG_CASCADE_OF_CYCLIN_EXPR	-0,142	1E-04	0,00048	1
REACTOME_TRANSPORT_OF_INORGANIC_CAT	-0,142	1E-04	0,00016	1
BIOCARTA_PTDINS_PATHWAY	-0,142	1E-04	0,0015	1
SENGUPTA_NASOPHARYNGEAL_CARCINOMA_	-0,142	1E-04	0,00172	1
TRANSCRIPTION_INITIATION	-0,142	1E-04	0,00374	1

REGULATION_OF_PHOSPHORYLATION	-0,142	1E-04	0,00013	1
WU_CELL_MIGRATION	-0,142	1E-04	0,00112	1
BMI1_DN_MEL18_DN.V1_UP	-0,142	1E-04	0,00103	1
P53_DN.V2_UP	-0,142	1E-04	0,00017	1
GSE9006_TYPE_1_DIABETES_AT_DX_VS_1M	-0,142	1E-04	0,00013	1
GSE12845_IGD_POS_BLOOD_VS_DARKZONE_	-0,142	1E-04	0,00843	1
NAKAMURA_METASTASIS_MODEL_DN	-0,142	1E-04	0,00033	1
TANG_SENESCENCE_TP53_TARGETS_DN	-0,142	1E-04	0,00032	1
CHEMNITZ_RESPONSE_TO_PROSTAGLANDIN_I	-0,142	1E-04	0,00025	1
HOFFMANN_LARGE_TO_SMALL_PRE_BII_LYMI	-0,142	1E-04	0,0003	1
LEIN_OLIGODENDROCYTE_MARKERS	-0,142	1E-04	0,00018	1
GSE15750_DAY6_VS_DAY10_TRAF6KO_EFF_	-0,141	1E-04	0,00026	1
CUI_GLUCOSE_DEPRIVATION	-0,141	1E-04	0,00892	1
HOSHIDA_LIVER_CANCER_LATE_RECURRENCE	-0,141	1E-04	0,00061	1
MODULE_324	-0,141	1E-04	0,00016	1
V$ELK1_02	-0,141	1E-04	0,00203	1
MORI_LARGE_PRE_BII_LYMPHOCYTE_UP	-0,141	1E-04	0,00023	1
GNF2_MSH2	-0,141	1E-04	0,00043	1
GNF2_DAP3	-0,141	1E-04	0,00026	1
SCIAN_CELL_CYCLE_TARGETS_OF_TP53_ANI	-0,141	1E-04	0,00014	1
TAKAO_RESPONSE_TO_UVB_RADIATION_DN	-0,141	1E-04	0,00024	1
MODULE_57	-0,141	1E-04	0,00039	1
GSE360_DC_VS_MAC_B_MALAYI_LOW_DOSE	-0,141	1E-04	0,00019	1
GSE1460_DP_THYMOCYTE_VS_NAIVE_CD4_T	-0,141	1E-04	0,00083	1
PID_REG_GR_PATHWAY	-0,141	1E-04	0,00016	1
CHUANG_OXIDATIVE_STRESS_RESPONSE_DN	-0,141	1E-04	0,00092	1
RIZ_ERYTHROID_DIFFERENTIATION_6HR	-0,141	1E-04	0,00044	1
KRCTCNNNNMANAGC_UNKNOWN	-0,141	1E-04	0,0076	1
GSE10239_NAIVE_VS_KLRG1INT_EFF_CD8_T	-0,141	1E-04	0,00093	1
TCGATGG,MIR-213	-0,141	1E-04	0,00083	1
CEBALLOS_TARGETS_OF_TP53_AND_MYC_D	-0,141	1E-04	0,00036	1
GSE24142_EARLY_THYMIC_PROGENITOR_VS_	-0,141	1E-04	0,00015	1
PETROVA_ENDOTHELIUM_LYMPHATIC_VS_BL	-0,141	1E-04	0,00013	1
REACTOME_APC_CDC20_MEDIATED_DEGRAD	-0,141	1E-04	0,00014	1
GSE8515_CTRL_VS_IL1_4H_STIM_MAC_DN	-0,14	1E-04	0,00049	1
GSE12845_PRE_GC_VS_DARKZONE_GC_TON	-0,14	1E-04	0,00051	1
GSE17721_POLYIC_VS_CPG_24H_BMDM_DN	-0,14	1E-04	0,0211	1
REACTOME_SYNTHESIS_OF_VERY_LONG_CHA	-0,14	1E-04	0,00015	1
OHASHI_AURKB_TARGETS	-0,14	1E-04	0,00052	1
REACTOME_G0_AND_EARLY_G1	-0,14	1E-04	0,00067	1
HOLLMANN_APOPTOSIS_VIA_CD40_UP	-0,14	1E-04	0,00123	1
GCM_HDAC1	-0,14	1E-04	0,00049	1

GEORGES_CELL_CYCLE_MIR192_TARGETS	-0,14	1E-04	0,00251	1
YIH_RESPONSE_TO_ARSENITE_C2	-0,14	1E-04	0,00371	1
ZEILSTRA_CD44_TARGETS_DN	-0,14	1E-04	0,00296	1
PID_E2F_PATHWAY	-0,14	1E-04	0,00048	1
ZHOU_CELL_CYCLE_GENES_IN_IR_RESPONSE_	-0,14	1E-04	0,0002	1
REACTOME_APC_C_CDC20_MEDIATED_DEGR/	-0,14	1E-04	0,00023	1
REACTOME_AMYLOIDS	-0,14	1E-04	0,00768	1
BOGNI_TREATMENT_RELATED_MYELOID_LEUK	-0,14	1E-04	0,00092	1
APPIERTO_RESPONSE_TO_FENRETINIDE_DN	-0,14	1E-04	0,01061	1
GSE17721_LPS_VS_POLYIC_4H_BMDM_UP	-0,14	1E-04	0,00057	1
MORF_CUL1	-0,14	1E-04	0,02192	1
POSITIVE_REGULATION_OF_CELL_MIGRATION	-0,14	1E-04	0,0007	1
MORF_RRM1	-0,14	1E-04	0,00116	1
MA_MYELOID_DIFFERENTIATION_UP	-0,14	1E-04	0,00014	1
LU_AGING_BRAIN_DN	-0,14	1E-04	0,00013	1
POTTI_ADRIAMYCIN_SENSITIVITY	-0,14	1E-04	0,00034	1
GNF2_FBL	-0,14	1E-04	0,00026	1
BOYERINAS_ONCOFETAL_TARGETS_OF_LET7/	-0,14	1E-04	0,00697	1
BOYAULT_LIVER_CANCER_SUBCLASS_G23_U	-0,14	1E-04	0,00233	1
CHICAS_RB1_TARGETS_LOW_SERUM	-0,14	1E-04	0,00117	1
MODULE_205	-0,14	1E-04	0,00014	1
FRASOR_RESPONSE_TO_SERM_OR_FULVESTR	-0,14	1E-04	0,00027	1
GSE339_EX_VIVO_VS_IN_CULTURE_CD4CD8I	-0,139	1E-04	0,00049	1
WEI_MYCN_TARGETS_WITH_E_BOX	-0,139	1E-04	0,01651	1
GSE17721_PAM3CSK4_VS_CPG_6H_BMDM_U	-0,139	1E-04	0,00438	1
MODULE_54	-0,139	1E-04	0,00059	1
TARTE_PLASMA_CELL_VS_PLASMABLAST_DI	-0,139	1E-04	0,00389	1
GSE17721_POLYIC_VS_CPG_6H_BMDM_UP	-0,139	1E-04	0,00299	1
GSE20366_TREG_VS_NAIVE_CD4_TCELL_HC	-0,139	1E-04	0,00055	1
ROY_WOUND_BLOOD_VESSEL_DN	-0,139	1E-04	0,00081	1
BROWNE_HCMV_INFECTION_12HR_UP	-0,139	1E-04	0,00125	1
ZHANG_PROLIFERATING_VS_QUIESCENT	-0,139	1E-04	0,00042	1
GSE17721_POLYIC_VS_GARDIQUIMOD_8H_BM	-0,139	1E-04	0,0008	1
CAMP_UP.V1_DN	-0,139	1E-04	0,00015	1
REACTOME_O_LINKED_GLYCOSYLATION_OF_I	-0,139	1E-04	0,00376	1
HESSON_TUMOR_SUPPRESSOR_CLUSTER_3P2	0,139	1E-04	0,03687	1
JIANG_AGING_CEREBRAL_CORTEX_UP	-0,139	1E-04	0,0002	1
TUOMISTO_TUMOR_SUPPRESSION_BY_COL13/	-0,139	1E-04	0,00069	1
MODULE_403	-0,139	1E-04	0,00035	1
chr10q11	-0,139	1E-04	0,00027	1
GSE18791_CTRL_VS_NEWCASTLE_VIRUS_DC	-0,139	1E-04	0,00393	1
GABA_RECEPTOR_ACTIVITY	-0,139	1E-04	0,00395	1

MARTINEZ_RESPONSE_TO_TRABECTEDIN_DN	-0,139	1E-04	0,00774	1
REACTOME_DEPOSITION_OF_NEW_CENPA_CC	-0,139	1E-04	0,00941	1
DARWICHE_PAPILLOMA_RISK_LOW_UP	-0,139	1E-04	0,00161	1
ACOSTA_PROLIFERATION_INDEPENDENT_MYC	-0,139	1E-04	0,00252	1
GAVIN_FOXP3_TARGETS_CLUSTER_P6	-0,138	2E-04	0,00055	1
REACTOME_PEROXISOMAL_LIPID_METABOLISM	0,138	2E-04	0,00137	1
GSE14308_TH2_VS_TH17_UP	-0,138	2E-04	0,00348	1
BIOSYNTHETIC_PROCESS	-0,138	2E-04	0,00018	1
GSE7460_CTRL_VS_FOXP3_OVEREXPR_TCON	-0,138	2E-04	0,00025	1
REACTOME_L1CAM_INTERACTIONS	-0,138	2E-04	0,00025	1
GSE3982_CENT_MEMORY_CD4_TCELL_VS_TI	-0,138	2E-04	0,0042	1
CAGGTCC,MIR-492	-0,138	2E-04	0,00013	1
ISHIDA_E2F_TARGETS	-0,138	2E-04	0,00059	1
MODULE_68	-0,138	2E-04	0,004	1
CELLULAR_BIOSYNTHETIC_PROCESS	-0,138	2E-04	0,00035	1
YOSHIOKA_LIVER_CANCER_EARLY_RECURREN	-0,138	2E-04	0,00722	1
STREICHER_LSM1_TARGETS_DN	-0,138	2E-04	0,00036	1
GSE5463_CTRL_VS_DEXAMETHASONE_TREAT	-0,138	2E-04	0,00064	1
PYEON_HPV_POSITIVE_TUMORS_UP	-0,138	2E-04	0,0002	1
MICROBODY_PART	0,138	2E-04	0,0035	1
PEROXISOMAL_PART	0,138	2E-04	0,0035	1
PROVENZANI_METASTASIS_DN	-0,138	2E-04	0,0013	1
V$TGIF_01	-0,138	2E-04	0,00017	1
BANDRES_RESPONSE_TO_CARMUSTIN_MGMT_	-0,138	2E-04	0,00027	1
GCM_ANP32B	-0,138	2E-04	0,00072	1
GSE17721_LPS_VS_PAM3CSK4_16H_BMDM_	-0,138	2E-04	0,00089	1
PURBEY_TARGETS_OF_CTBP1_AND_SATB1_U	-0,138	2E-04	0,00019	1
DNA_DIRECTED_RNA_POLYMERASE_COMPLEX	-0,138	2E-04	0,00083	1
NUCLEAR_DNA_DIRECTED_RNA_POLYMERASE	-0,138	2E-04	0,00083	1
RNA_POLYMERASE_COMPLEX	-0,138	2E-04	0,00083	1
INDUCTION_OF_APOPTOSIS_BY_EXTRACELLUI	-0,138	2E-04	0,0005	1
V$AP1_Q4	-0,138	2E-04	0,00073	1
V$HLF_01	-0,138	2E-04	0,00017	1
IYENGAR_RESPONSE_TO_ADIPOCYTE_FACTOF	-0,138	2E-04	0,00243	1
HU_GENOTOXIC_DAMAGE_4HR	-0,138	2E-04	0,00064	1
GAUSSMANN_MLL_AF4_FUSION_TARGETS_E_	-0,138	2E-04	0,00113	1
SENESE_HDAC1_AND_HDAC2_TARGETS_UP	-0,138	2E-04	0,00248	1
MORI_IMMATURE_B_LYMPHOCYTE_DN	-0,138	2E-04	0,00048	1
LIPID_BIOSYNTHETIC_PROCESS	-0,137	2E-04	0,00113	1
GSE3982_EOSINOPHIL_VS_TH1_DN	-0,137	2E-04	0,0053	1
MITSIADES_RESPONSE_TO_APLIDIN_DN	-0,137	2E-04	0,00259	1
MODULE_552	-0,137	2E-04	0,0117	1

DAZARD_UV_RESPONSE_CLUSTER_G4	-0,137	2E-04	0,00077	1
V$NRSF_01	-0,137	2E-04	0,00041	1
GHANDHI_DIRECT_IRRADIATION_UP	-0,137	2E-04	0,00169	1
KORKOLA_CORRELATED_WITH_POU5F1	-0,137	2E-04	0,00236	1
KRAS.LUNG_UP.V1_DN	-0,137	2E-04	0,0004	1
KEGG_BASAL_TRANSCRIPTION_FACTORS	-0,137	2E-04	0,01015	1
GNF2_MCM5	-0,137	2E-04	0,00067	1
BIOCARTA_DNAFRAGMENT_PATHWAY	-0,137	2E-04	0,00151	1
HUANG_GATA2_TARGETS_DN	-0,137	2E-04	0,00204	1
MODULE_509	-0,137	2E-04	0,01924	1
GSE1460_CORD_VS_ADULT_BLOOD_NAIVE_(	-0,137	2E-04	0,00016	1
CROONQUIST_IL6_DEPRIVATION_DN	-0,137	2E-04	0,00036	1
YE_METASTATIC_LIVER_CANCER	-0,137	2E-04	0,00013	1
GSE24142_ADULT_VS_FETAL_DN2_THYMOC	-0,137	2E-04	0,00029	1
DNA_DIRECTED_RNA_POLYMERASEII_HOLOEN	-0,137	2E-04	0,00293	1
V$HIF1_Q5	-0,137	2E-04	0,00225	1
FOURNIER_ACINAR_DEVELOPMENT_LATE_DN	-0,137	2E-04	0,00391	1
MYC_UP.V1_DN	-0,137	2E-04	0,00015	1
GSE29615_CTRL_VS_LAIV_FLU_VACCINE_PE	-0,137	2E-04	0,00056	1
GARGALOVIC_RESPONSE_TO_OXIDIZED_PHOS	-0,137	2E-04	0,00035	1
NUCLEAR_IMPORT	-0,137	2E-04	0,00879	1
NFE2L2.V2	-0,137	2E-04	0,00056	1
chr16q24	-0,137	2E-04	0,00548	1
GSE24634_TREG_VS_TCONV_POST_DAY3_IL	-0,137	2E-04	0,00135	1
MAYBURD_RESPONSE_TO_L663536_DN	-0,137	2E-04	0,00377	1
HANN_RESISTANCE_TO_BCL2_INHIBITOR_DN	-0,137	2E-04	0,00038	1
COATED_VESICLE_MEMBRANE	-0,137	2E-04	0,00414	1
LU_TUMOR_ANGIOGENESIS_UP	-0,136	2E-04	0,00342	1
GSE360_CTRL_VS_L_DONOVANI_DC_DN	-0,136	2E-04	0,00025	1
NAKAMURA_CANCER_MICROENVIRONMENT_D	-0,136	2E-04	0,00402	1
KEGG_DORSO_VENTRAL_AXIS_FORMATION	-0,136	2E-04	0,00099	1
NAKAYAMA_FRA2_TARGETS	-0,136	2E-04	0,00024	1
LIANG_HEMATOPOIESIS_STEM_CELL_NUMBER	-0,136	2E-04	0,00297	1
GU_PDEF_TARGETS_UP	-0,136	2E-04	0,00013	1
SCHLOSSER_MYC_TARGETS_AND_SERUM_RES	-0,136	2E-04	0,01291	1
LEE_EARLY_T_LYMPHOCYTE_UP	-0,136	2E-04	0,00084	1
PYEON_HPV_POSITIVE_TUMORS_DN	-0,136	2E-04	0,00029	1
KEGG_BLADDER_CANCER	-0,136	2E-04	0,01093	1
REACTOME_APOBEC3G_MEDIATED_RESISTAN(	-0,136	2E-04	0,00148	1
REACTOME_METABOLISM_OF_RNA	-0,136	2E-04	0,00104	1
REACTOME_MITOTIC_G2_G2_M_PHASES	-0,136	2E-04	0,00077	1
KRAS.DF.V1_UP	-0,136	2E-04	0,00017	1

GSE10239_MEMORY_VS_KLRG1INT_EFF_CD8	-0,136	2E-04	0,00372	1
IIZUKA_LIVER_CANCER_PROGRESSION_L0_L1	-0,136	2E-04	0,00077	1
TURASHVILI_BREAST_NORMAL_DUCTAL_VS_	-0,136	2E-04	0,0003	1
SATO_SILENCED_BY_METHYLATION_IN_PANC	-0,136	2E-04	0,00157	1
REACTOME_RNA_POL_III_TRANSCRIPTION	-0,136	2E-04	0,00415	1
GSE11864_UNTREATED_VS_CSF1_PAM3CYS_	-0,135	2E-04	0,00123	1
V$ARNT_02	-0,135	2E-04	0,00348	1
ZHANG_TLX_TARGETS_60HR_DN	-0,135	2E-04	0,00069	1
REACTOME_TERMINATION_OF_O_GLYCAN_BI(	-0,135	2E-04	0,00207	1
KAECH_NAIVE_VS_DAY8_EFF_CD8_TCELL_D	-0,135	2E-04	0,00221	1
BOYAULT_LIVER_CANCER_SUBCLASS_G123_	-0,135	2E-04	0,00401	1
ZHAN_MULTIPLE_MYELOMA_CD2_DN	-0,135	2E-04	0,01228	1
FLECHNER_PBL_KIDNEY_TRANSPLANT_REJEC	-0,135	2E-04	0,00266	1
SCHLOSSER_MYC_TARGETS_AND_SERUM_RES	-0,135	2E-04	0,01995	1
GNF2_SMC4L1	-0,135	2E-04	0,0006	1
chr2p	-0,135	2E-04	0,00523	1
GNF2_MLH1	-0,135	2E-04	0,00592	1
FIRESTEIN_PROLIFERATION	-0,135	2E-04	0,00069	1
chrxq25	-0,135	2E-04	0,00022	1
CHANG_CYCLING_GENES	-0,135	2E-04	0,00066	1
MODULE_70	-0,135	2E-04	0,00021	1
TRANSFERASE_ACTIVITY_TRANSFERRING_GLY	-0,135	2E-04	0,00654	1
KYNG_DNA_DAMAGE_DN	-0,135	2E-04	0,00019	1
ZERBINI_RESPONSE_TO_SULINDAC_DN	-0,135	2E-04	0,0003	1
GSE17721_LPS_VS_PAM3CSK4_1H_BMDM_L	-0,135	2E-04	0,00055	1
GSE17721_LPS_VS_GARDIQUIMOD_6H_BMDN	-0,135	2E-04	0,00546	1
KEGG_BUTANOATE_METABOLISM	0,135	2E-04	0,00185	1
MODULE_451	-0,135	2E-04	0,0006	1
REACTOME_INHIBITION_OF_VOLTAGE_GATED	-0,135	2E-04	0,00162	1
MODULE_222	-0,135	2E-04	0,03576	1
WHITFIELD_CELL_CYCLE_G1_S	-0,135	2E-04	0,00042	1
PROTEIN_HETERODIMERIZATION_ACTIVITY	-0,135	2E-04	0,00094	1
chr2p24	-0,135	2E-04	0,00292	1
GOLDRATH_NAIVE_VS_MEMORY_CD8_TCELL_	-0,135	2E-04	0,00035	1
LIANG_HEMATOPOIESIS_STEM_CELL_NUMBER	-0,135	2E-04	0,00058	1
LASTOWSKA_COAMPLIFIED_WITH_MYCN	-0,135	2E-04	0,00669	1
ZHOU_TNF_SIGNALING_30MIN	-0,135	2E-04	0,00049	1
THILLAINADESAN_ZNF217_TARGETS_UP	-0,135	2E-04	0,00074	1
MANALO_HYPOXIA_DN	-0,135	2E-04	0,00298	1
GSE17721_POLYIC_VS_CPG_6H_BMDM_DN	-0,134	2E-04	0,00029	1
MONTERO_THYROID_CANCER_POOR_SURVIVA	-0,134	2E-04	0,00139	1
MORF_CDK2	-0,134	2E-04	0,01313	1

REACTOME_TRANSPORT_OF_MATURE_TRANS	-0,134	2E-04	0,00611	1
GSE17721_LPS_VS_CPG_4H_BMDM_UP	-0,134	2E-04	0,00016	1
KIM_PTEN_TARGETS_DN	-0,134	2E-04	0,00112	1
chr15q	-0,134	2E-04	0,00755	1
AGTCTAG,MIR-151	-0,134	2E-04	0,00018	1
GCM_PSME1	-0,134	2E-04	0,00225	1
GSE17974_2.5H_VS_72H_IL4_AND_ANTI_IL	-0,134	2E-04	0,00068	1
chr7q22	-0,134	2E-04	0,00022	1
CHOI_ATL_STAGE_PREDICTOR	-0,134	2E-04	0,00876	1
REACTOME_CRMPS_IN_SEMA3A_SIGNALING	-0,134	2E-04	0,00607	1
REACTOME_HIV_LIFE_CYCLE	-0,134	2E-04	0,00474	1
BENPORATH_MYC_TARGETS_WITH_EBOX	-0,134	2E-04	0,00141	1
MATTIOLI_MGUS_VS_PCL	-0,134	2E-04	0,0052	1
CONDENSED_CHROMOSOME	-0,134	2E-04	0,00076	1
GOLDRATH_EFF_VS_MEMORY_CD8_TCELL_UI	-0,134	2E-04	0,00222	1
REACTOME_MRNA_SPLICING	-0,134	2E-04	0,00446	1
RODRIGUES_THYROID_CARCINOMA_ANAPLAS	-0,134	2E-04	0,03135	1
GSE2706_R848_VS_R848_AND_LPS_8H_STI	-0,134	2E-04	0,00067	1
GOTZMANN_EPITHELIAL_TO_MESENCHYMAL_	-0,134	2E-04	0,0019	1
PROTEIN_MODIFICATION_PROCESS	-0,134	2E-04	0,00016	1
MARTORIATI_MDM4_TARGETS_FETAL_LIVER_	-0,134	2E-04	0,00018	1
PROTEIN_SERINE_THREONINE_KINASE_ACTIVI	-0,134	2E-04	0,0008	1
KUROKAWA_LIVER_CANCER_EARLY_RECURRE	-0,134	3E-04	0,01081	1
REACTOME_TELOMERE_MAINTENANCE	-0,134	3E-04	0,01124	1
GSE9006_HEALTHY_VS_TYPE_1_DIABETES_I	-0,134	3E-04	0,00165	1
REACTOME_ANTIGEN_PROCESSING_UBIQUITIN	-0,134	3E-04	0,01205	1
GACTGTT,MIR-212,MIR-132	-0,134	3E-04	0,00031	1
SCHLESINGER_METHYLATED_IN_COLON_CAN	-0,134	3E-04	0,00041	1
GSE13411_NAIVE_VS_IGM_MEMORY_BCELL_	-0,134	3E-04	0,00027	1
GSE24634_TREG_VS_TCONV_POST_DAY5_IL	-0,134	3E-04	0,00666	1
GSE2826_XID_VS_BTK_KO_BCELL_DN	-0,134	3E-04	0,00098	1
NEMETH_INFLAMMATORY_RESPONSE_LPS_DN	-0,134	3E-04	0,0033	1
WATANABE_RECTAL_CANCER_RADIOTHERAP	-0,134	3E-04	0,00268	1
NITROGEN_COMPOUND_BIOSYNTHETIC_PROCE	-0,134	3E-04	0,0003	1
HELLER_HDAC_TARGETS_DN	-0,134	3E-04	0,00131	1
REACTOME_METABOLISM_OF_POLYAMINES	-0,133	3E-04	0,00749	1
MARCHINI_TRABECTEDIN_RESISTANCE_DN	-0,133	3E-04	0,0052	1
CHEN_NEUROBLASTOMA_COPY_NUMBER_GAI	-0,133	3E-04	0,00013	1
WHITE_NEUROBLASTOMA_WITH_1P36.3_DEL	-0,133	3E-04	0,00033	1
MEIOTIC_RECOMBINATION	-0,133	3E-04	0,00022	1
GSE5463_CTRL_VS_DEXAMETHASONE_TREA	-0,133	3E-04	0,00024	1
KONG_E2F3_TARGETS	-0,133	3E-04	0,00084	1

GCM_ACTG1	-0,133	3E-04	0,00199	1
MORF_EIF4A2	-0,133	3E-04	0,00054	1
GSE17721_LPS_VS_GARDIQUIMOD_4H_BMDM	-0,133	3E-04	0,00323	1
GSE339_CD4POS_VS_CD8POS_DC_IN_CULTU	-0,133	3E-04	0,0008	1
MODULE_181	-0,133	3E-04	0,00019	1
CAHOY_ASTROCYTIC	-0,133	3E-04	0,00081	1
GNF2_H2AFX	-0,133	3E-04	0,00048	1
GSE17721_LPS_VS_CPG_6H_BMDM_UP	-0,133	3E-04	0,00029	1
MODULE_298	-0,133	3E-04	0,00538	1
KRIGE_RESPONSE_TO_TOSEDOSTAT_6HR_DN	-0,133	3E-04	0,00279	1
LIANG_SILENCED_BY_METHYLATION_DN	-0,133	3E-04	0,00068	1
GRUETZMANN_PANCREATIC_CANCER_UP	-0,133	3E-04	0,00147	1
REACTOME_MEIOTIC_SYNAPSIS	-0,133	3E-04	0,01115	1
WEST_ADRENOCORTICAL_TUMOR_UP	-0,133	3E-04	0,00751	1
GSE29618_PRE_VS_DAY7_POST_TIV_FLU_V	-0,133	3E-04	0,00028	1
PHONG_TNF_RESPONSE_NOT_VIA_P38	-0,133	3E-04	0,0006	1
KEGG_ENDOMETRIAL_CANCER	-0,133	3E-04	0,00038	1
GSE17721_PAM3CSK4_VS_CPG_24H_BMDM_	-0,133	3E-04	0,00023	1
chr2p21	-0,133	3E-04	0,01152	1
PID_EPHRINBREVPATHWAY	-0,133	3E-04	0,0011	1
MGGAAGTG_V$GABP_B	-0,133	3E-04	0,00019	1
GCAAGAC,MIR-431	-0,133	3E-04	0,00222	1
E2F3_UP.V1_DN	-0,133	3E-04	0,00048	1
REACTOME_CELL_CYCLE_MITOTIC	-0,133	3E-04	0,00131	1
REACTOME_REGULATION_OF_THE_FANCONI_	-0,133	3E-04	0,00321	1
WANG_METASTASIS_OF_BREAST_CANCER_ES	-0,133	3E-04	0,00065	1
CROONQUIST_NRAS_SIGNALING_DN	-0,133	3E-04	0,0007	1
GSE26669_CTRL_VS_COSTIM_BLOCK_MLR_C	-0,133	3E-04	0,00163	1
GSE17721_12H_VS_24H_PAM3CSK4_BMDM_	-0,132	3E-04	0,00092	1
PEPTIDYL_TYROSINE_PHOSPHORYLATION	-0,132	3E-04	0,00061	1
GSE17721_CTRL_VS_CPG_24H_BMDM_UP	-0,132	3E-04	0,00015	1
NEGATIVE_REGULATION_OF_MAP_KINASE_AC	-0,132	3E-04	0,00024	1
V$GNCF_01	-0,132	3E-04	0,00056	1
GSE3982_CTRL_VS_IGE_STIM_MAST_CELL_D	-0,132	3E-04	0,00026	1
GSE17721_CTRL_VS_LPS_2H_BMDM_DN	-0,132	3E-04	0,0003	1
REACTOME_PREFOLDIN_MEDIATED_TRANSFEF	-0,132	3E-04	0,01119	1
V$IPF1_Q4	-0,132	3E-04	0,00017	1
APRELIKOVA_BRCA1_TARGETS	-0,132	3E-04	0,01896	1
V$CEBPB_01	-0,132	3E-04	0,0002	1
GNF2_MMP1	-0,132	3E-04	0,01247	1
TELOMERIC_DNA_BINDING	-0,132	3E-04	0,00099	1
ESTABLISHMENT_OF_ORGANELLE_LOCALIZAT	-0,132	3E-04	0,00207	1

GSE29618_PRE_VS_DAY7_POST_TIV_FLU_V	-0,132	3E-04	0,00022	1
LEE_LIVER_CANCER_MYC_TGFA_UP	-0,132	3E-04	0,00175	1
JNK_DN.V1_UP	-0,132	3E-04	0,00025	1
RHODES_UNDIFFERENTIATED_CANCER	-0,132	3E-04	0,00187	1
KAUFFMANN_DNA_REPAIR_GENES	-0,132	3E-04	0,00026	1
BURTON_ADIPOGENESIS_PEAK_AT_16HR	-0,132	3E-04	0,00272	1
ST_FAS_SIGNALING_PATHWAY	-0,132	3E-04	0,00358	1
GSE27786_BCELL_VS_MONO_MAC_UP	-0,132	3E-04	0,00035	1
V$EVI1_06	-0,132	3E-04	0,00027	1
KEGG_GLYCOSYLPHOSPHATIDYLINOSITOL_GPI	0,132	3E-04	0,00434	1
CHROMATIN_MODIFICATION	-0,132	3E-04	0,00226	1
MORF_RAD54L	-0,132	3E-04	0,01399	1
REACTOME_DESTABILIZATION_OF_MRNA_BY_	-0,132	3E-04	0,00139	1
SATO_SILENCED_EPIGENETICALLY_IN_PANCRI	-0,132	3E-04	0,00277	1
AMIT_SERUM_RESPONSE_480_MCF10A	-0,132	3E-04	0,05391	1
MORF_SS18	-0,132	3E-04	0,00044	1
GRAHAM_NORMAL_QUIESCENT_VS_NORMAL_	-0,132	3E-04	0,00107	1
GALACTOSYLTRANSFERASE_ACTIVITY	-0,132	3E-04	0,00166	1
V$AP1_01	-0,132	3E-04	0,00112	1
GCM_NPM1	-0,132	3E-04	0,0004	1
GSE29618_BCELL_VS_MONOCYTE_UP	-0,132	3E-04	0,0003	1
FIGUEROA_AML_METHYLATION_CLUSTER_3_I	-0,132	3E-04	0,00036	1
YANG_BREAST_CANCER_ESR1_BULK_DN	-0,132	3E-04	0,02009	1
GNF2_CDC2	-0,132	3E-04	0,00072	1
MODULE_168	-0,132	3E-04	0,04122	1
REGULATION_OF_RESPONSE_TO_STIMULUS	-0,131	3E-04	0,00035	1
GNF2_ANP32B	-0,131	3E-04	0,00303	1
MORF_BUB1B	-0,131	3E-04	0,00231	1
SOUCEK_MYC_TARGETS	-0,131	3E-04	0,00545	1
MODULE_328	-0,131	3E-04	0,00201	1
VITAMIN_METABOLIC_PROCESS	-0,131	3E-04	0,00307	1
REACTOME_TANDEM_PORE_DOMAIN_POTASS	-0,131	3E-04	0,00044	1
GCM_CSNK2B	-0,131	3E-04	0,00263	1
RIGGI_EWING_SARCOMA_PROGENITOR_DN	-0,131	3E-04	0,00019	1
GNF2_PTX3	-0,131	3E-04	0,00101	1
REACTOME_SIGNAL_TRANSDUCTION_BY_L1	-0,131	3E-04	0,01142	1
OKAWA_NEUROBLASTOMA_1P36_31_DELETI	-0,131	3E-04	0,00035	1
chr2p25	-0,131	3E-04	0,00959	1
REACTOME_G1_S_SPECIFIC_TRANSCRIPTION	-0,131	3E-04	0,00186	1
GSE17721_LPS_VS_POLYIC_8H_BMDM_UP	-0,131	3E-04	0,01908	1
ST_INTERLEUKIN_4_PATHWAY	-0,131	3E-04	0,00038	1
GSE17721_4_VS_24H_GARDIQUIMOD_BMDM_	-0,131	3E-04	0,00036	1

MORF_RFC1	-0,131	3E-04	0,01635	1
GNF2_BUB1	-0,131	3E-04	0,00127	1
AMINO_ACID_CATABOLIC_PROCESS	0,131	3E-04	0,00583	1
GSE17721_PAM3CSK4_VS_GADIQUIMOD_1H_	-0,131	3E-04	0,02183	1
GNF2_CENPE	-0,131	3E-04	0,00077	1
PID_FGF_PATHWAY	-0,131	3E-04	0,00044	1
GSE36476_CTRL_VS_TSST_ACT_72H_MEMO	-0,131	3E-04	0,0029	1
REGULATION_OF_PEPTIDYL_TYROSINE_PHOSF	-0,131	3E-04	0,00097	1
ZERBINI_RESPONSE_TO_SULINDAC_UP	-0,131	3E-04	0,00117	1
STEIN_ESRRA_TARGETS_DN	-0,131	3E-04	0,00031	1
chr13q31	-0,131	3E-04	0,00409	1
GSE3982_MAC_VS_NEUTROPHIL_LPS_STIM_U	-0,131	3E-04	0,01102	1
BIOCARTA_G2_PATHWAY	-0,131	3E-04	0,00259	1
GSE3982_NKCELL_VS_TH1_DN	-0,131	3E-04	0,01271	1
REACTOME_RNA_POL_I_TRANSCRIPTION	-0,131	3E-04	0,01092	1
GSE18791_CTRL_VS_NEWCASTLE_VIRUS_DC	-0,131	3E-04	0,00339	1
GSE18791_CTRL_VS_NEWCASTLE_VIRUS_DC	-0,131	3E-04	0,0175	1
GSE360_CTRL_VS_B_MALAYI_LOW_DOSE_D	-0,131	3E-04	0,00013	1
JIANG_HYPOXIA_VIA_VHL	-0,131	3E-04	0,00495	1
GSE36392_TYPE_2_MYELOID_VS_NEUTROPH	-0,131	4E-04	0,00059	1
FONTAINE_THYROID_TUMOR_UNCERTAIN_MAI	-0,131	4E-04	0,00029	1
SASSON_RESPONSE_TO_GONADOTROPHINS_U	-0,131	4E-04	0,0039	1
RASHI_RESPONSE_TO_IONIZING_RADIATION_2	-0,131	4E-04	0,00082	1
DNA_POLYMERASE_ACTIVITY	-0,131	4E-04	0,00022	1
GRAHAM_CML_DIVIDING_VS_NORMAL_DIVIDII	-0,131	4E-04	0,01987	1
AMUNDSON_RESPONSE_TO_ARSENITE	-0,131	4E-04	0,00383	1
REACTOME_CYCLIN_A_B1_ASSOCIATED_EVEI	-0,131	4E-04	0,00118	1
RNA_POLYMERASE_II_TRANSCRIPTION_FACTC	-0,13	4E-04	0,00029	1
DNA_INTEGRITY_CHECKPOINT	-0,13	4E-04	0,00206	1
KOMMAGANI_TP63_GAMMA_TARGETS	-0,13	4E-04	0,001	1
TACTTGA,MIR-26A,MIR-26B	-0,13	4E-04	0,00031	1
GSE17721_0.5H_VS_8H_LPS_BMDM_UP	-0,13	4E-04	0,00055	1
KANNAN_TP53_TARGETS_DN	-0,13	4E-04	0,05589	1
WANG_METASTASIS_OF_BREAST_CANCER	-0,13	4E-04	0,01634	1
YU_MYC_TARGETS_UP	-0,13	4E-04	0,00156	1
SARTIPY_BLUNTED_BY_INSULIN_RESISTANCE_	-0,13	4E-04	0,00165	1
KAN_RESPONSE_TO_ARSENIC_TRIOXIDE	-0,13	4E-04	0,00983	1
BMI1_DN.V1_UP	-0,13	4E-04	0,00216	1
chr1p	-0,13	4E-04	0,00463	1
CHEN_LUNG_CANCER_SURVIVAL	-0,13	4E-04	0,00641	1
HORMONE_RECEPTOR_BINDING	-0,13	4E-04	0,00452	1
GNF2_CENPF	-0,13	4E-04	0,00079	1

TGACCTTG_V$SF1_Q6	-0,13	4E-04	0,001	1
GCM_DDX5	-0,13	4E-04	0,01013	1
GSE17721_LPS_VS_POLYIC_1H_BMDM_UP	-0,13	4E-04	0,00057	1
GSE17974_CTRL_VS_ACT_IL4_AND_ANTI_IL	-0,13	4E-04	0,03211	1
GCGNNANTTCC_UNKNOWN	-0,13	4E-04	0,00028	1
GSE339_CD4POS_VS_CD4CD8DN_DC_DN	-0,13	4E-04	0,00133	1
RIZ_ERYTHROID_DIFFERENTIATION	-0,13	4E-04	0,00103	1
DARWICHE_SKIN_TUMOR_PROMOTER_UP	-0,13	4E-04	0,00232	1
REACTOME_CHONDROITIN_SULFATE_BIOSYNT	-0,13	4E-04	0,00069	1
DAZARD_UV_RESPONSE_CLUSTER_G6	-0,13	4E-04	0,00223	1
PACHER_TARGETS_OF_IGF1_AND_IGF2_UP	-0,13	4E-04	0,00107	1
SONG_TARGETS_OF_IE86_CMV_PROTEIN	-0,13	4E-04	0,00124	1
MORF_RPA2	-0,13	4E-04	0,01481	1
KINASE_ACTIVITY	-0,13	4E-04	0,00018	1
KEGG_NON_SMALL_CELL_LUNG_CANCER	-0,13	4E-04	0,00388	1
V$AP1_Q2	-0,13	4E-04	0,00032	1
MICROTUBULE	-0,13	4E-04	0,0005	1
GNF2_PCNA	-0,13	4E-04	0,00086	1
JECHLINGER_EPITHELIAL_TO_MESENCHYMAL_	-0,13	4E-04	0,00609	1
chr4q27	-0,13	4E-04	0,01181	1
MANN_RESPONSE_TO_AMIFOSTINE_DN	-0,13	4E-04	0,00266	1
LIEN_BREAST_CARCINOMA_METAPLASTIC_VS	-0,13	4E-04	0,00697	1
MYLLYKANGAS_AMPLIFICATION_HOT_SPOT_2	-0,13	4E-04	0,00025	1
ABRAMSON_INTERACT_WITH_AIRE	-0,13	4E-04	0,01021	1
BIOGENIC_AMINE_METABOLIC_PROCESS	-0,13	4E-04	0,00137	1
ONGUSAHA_TP53_TARGETS	-0,13	4E-04	0,00489	1
GNF2_RFC3	-0,129	4E-04	0,0013	1
REACTOME_RNA_POL_I_PROMOTER_OPENING	-0,129	4E-04	0,01264	1
SESTO_RESPONSE_TO_UV_C6	-0,129	4E-04	0,00052	1
NEGATIVE_REGULATION_OF_INTRACELLULAR_	-0,129	4E-04	0,00139	1
MORF_SMC1L1	-0,129	4E-04	0,01602	1
GSE22886_NAIVE_CD4_TCELL_VS_NEUTROP	-0,129	4E-04	0,00038	1
TGASTMAGC_V$NFE2_01	-0,129	4E-04	0,00044	1
NGO_MALIGNANT_GLIOMA_1P_LOH	-0,129	4E-04	0,0589	1
REACTOME_G2_M_DNA_DAMAGE_CHECKPOIN	-0,129	4E-04	0,00105	1
BROWNE_HCMV_INFECTION_8HR_DN	-0,129	4E-04	0,00221	1
GSE36476_YOUNG_VS_OLD_DONOR_MEMOR	-0,129	4E-04	0,0017	1
GNF2_RRM1	-0,129	4E-04	0,0014	1
FONTAINE_THYROID_TUMOR_UNCERTAIN_MA	-0,129	4E-04	0,00096	1
JOHANSSON_GLIOMAGENESIS_BY_PDGFB_DN	-0,129	4E-04	0,00061	1
LOPEZ_MESOTHELIOMA_SURVIVAL_DN	-0,129	4E-04	0,01892	1
REACTOME_DOWNREGULATION_OF_SMAD2_3	-0,129	4E-04	0,00144	1

ZHAN_MULTIPLE_MYELOMA_MS_DN	-0,129	4E-04	0,00336	1
LEE_METASTASIS_AND_RNA_PROCESSING_UI	-0,129	4E-04	0,01019	1
GNF2_TTK	-0,129	4E-04	0,00112	1
GNF2_CCNA2	-0,129	4E-04	0,0011	1
GNF2_CKS1B	-0,129	4E-04	0,00188	1
KUMAR_PATHOGEN_LOAD_BY_MACROPHAGE:	-0,129	4E-04	0,00033	1
BOYAULT_LIVER_CANCER_SUBCLASS_G3_UP	-0,129	4E-04	0,01759	1
RIZKI_TUMOR_INVASIVENESS_3D_DN	-0,129	4E-04	0,00115	1
DNA_DAMAGE_RESPONSESIGNAL_TRANSDUC	-0,129	4E-04	0,00039	1
IKEDA_MIR30_TARGETS_DN	-0,129	4E-04	0,001	1
HOEBEKE_LYMPHOID_STEM_CELL_DN	-0,129	4E-04	0,00037	1
REGULATION_OF_PROTEIN_MODIFICATION_PR	-0,129	4E-04	0,00029	1
GSE17974_CTRL_VS_ACT_IL4_AND_ANTI_IL	-0,129	4E-04	0,01305	1
GSE3982_MAC_VS_NEUTROPHIL_UP	-0,129	4E-04	0,03139	1
MOLENAAR_TARGETS_OF_CCND1_AND_CDK4	-0,129	4E-04	0,00244	1
CCAGGTT,MIR-490	-0,129	4E-04	0,00611	1
DAVIES_MULTIPLE_MYELOMA_VS_MGUS_UP	-0,129	4E-04	0,00026	1
GRAHAM_CML_QUIESCENT_VS_NORMAL_QUI	-0,129	4E-04	0,00239	1
MITOTIC_SISTER_CHROMATID_SEGREGATION	-0,129	4E-04	0,0023	1
GRASEMANN_RETINOBLASTOMA_WITH_6P_A	-0,129	4E-04	0,0146	1
SHAFFER_IRF4_TARGETS_IN_ACTIVATED_DE	-0,129	4E-04	0,01143	1
BIOCARTA_PARKIN_PATHWAY	-0,129	4E-04	0,00029	1
MORF_BUB3	-0,129	4E-04	0,01468	1
WINNEPENNINCKX_MELANOMA_METASTASIS_	-0,129	4E-04	0,00323	1
WANG_BARRETTS_ESOPHAGUS_AND_ESOPH/	-0,129	4E-04	0,00573	1
GSE7852_TREG_VS_TCONV_FAT_DN	-0,129	4E-04	0,00035	1
REACTOME_MITOTIC_G1_G1_S_PHASES	-0,129	4E-04	0,00543	1
GSE31082_DP_VS_CD8_SP_THYMOCYTE_UP	-0,129	4E-04	0,00028	1
GSE17721_12H_VS_24H_POLYIC_BMDM_UP	-0,129	4E-04	0,00198	1
GSE3982_EFF_MEMORY_CD4_TCELL_VS_TH1	-0,129	4E-04	0,01825	1
PID_RAC1_PATHWAY	-0,129	4E-04	0,0141	1
GNF2_HMMR	-0,129	4E-04	0,00143	1
REACTOME_LOSS_OF_NLP_FROM_MITOTIC_C	-0,129	4E-04	0,00425	1
MORF_GNB1	-0,128	4E-04	0,01955	1
ATACTGT,MIR-144	-0,128	4E-04	0,00039	1
GRAHAM_CML_QUIESCENT_VS_NORMAL_DIVI	-0,128	4E-04	0,00136	1
EGUCHI_CELL_CYCLE_RB1_TARGETS	-0,128	4E-04	0,00151	1
KAECH_DAY8_EFF_VS_DAY15_EFF_CD8_TCE	-0,128	4E-04	0,00426	1
GSE17721_LPS_VS_POLYIC_12H_BMDM_UP	-0,128	4E-04	0,01537	1
MORF_CCNI	-0,128	4E-04	0,00167	1
PID_EPHA_FWDPATHWAY	-0,128	5E-04	0,00091	1
MORI_SMALL_PRE_BII_LYMPHOCYTE_DN	-0,128	5E-04	0,01843	1

PID_PRLSIGNALINGEVENTSPATHWAY	-0,128	5E-04	0,00491	1
GSE17721_LPS_VS_CPG_12H_BMDM_DN	-0,128	5E-04	0,00077	1
IWANAGA_E2F1_TARGETS_INDUCED_BY_SER	-0,128	5E-04	0,00384	1
MARIADASON_REGULATED_BY_HISTONE_ACE	-0,128	5E-04	0,00042	1
GSE20715_WT_VS_TLR4_KO_6H_OZONE_LU	-0,128	5E-04	0,00015	1
GSE10325_BCELL_VS_MYELOID_UP	-0,128	5E-04	0,00027	1
MEINHOLD_OVARIAN_CANCER_LOW_GRADE_	-0,128	5E-04	0,00288	1
REACTOME_INHIBITION_OF_THE_PROTEOLYTI	-0,128	5E-04	0,0011	1
FINETTI_BREAST_CANCER_KINOME_RED	-0,128	5E-04	0,00262	1
HILLION_HMGA1_TARGETS	-0,128	5E-04	0,0004	1
ZHOU_CELL_CYCLE_GENES_IN_IR_RESPONSE_	-0,128	5E-04	0,00087	1
MCCLUNG_COCAIN_REWARD_4WK	-0,128	5E-04	0,00074	1
GNF2_FEN1	-0,128	5E-04	0,00115	1
GSE17974_0H_VS_12H_IN_VITRO_ACT_CD4	-0,128	5E-04	0,01454	1
GSE13493_DP_VS_CD8POS_THYMOCYTE_DN	-0,128	5E-04	0,00099	1
GSE7460_WT_VS_FOXP3_HET_ACT_WITH_T	-0,128	5E-04	0,00066	1
V$ROAZ_01	-0,128	5E-04	0,00661	1
PID_PDGFRAPATHWAY	-0,128	5E-04	0,00032	1
HELLER_SILENCED_BY_METHYLATION_DN	-0,128	5E-04	0,00186	1
MODULE_7	-0,128	5E-04	0,00058	1
BIOCARTA_RANKL_PATHWAY	-0,128	5E-04	0,0078	1
SCHMIDT_POR_TARGETS_IN_LIMB_BUD_UP	-0,128	5E-04	0,02359	1
GOLDRATH_NAIVE_VS_EFF_CD8_TCELL_DN	-0,128	5E-04	0,00456	1
MODULE_16	-0,128	5E-04	0,0059	1
DARWICHE_PAPILLOMA_RISK_HIGH_UP	-0,128	5E-04	0,00531	1
GNF2_SMC2L1	-0,128	5E-04	0,00127	1
GSE32423_MEMORY_VS_NAIVE_CD8_TCELL_	-0,128	5E-04	0,00127	1
LI_CISPLATIN_RESISTANCE_UP	-0,127	5E-04	0,00042	1
MODULE_385	-0,127	5E-04	0,00149	1
GSE7852_LN_VS_FAT_TREG_UP	-0,127	5E-04	0,00151	1
RESPONSE_TO_IONIZING_RADIATION	-0,127	5E-04	0,00121	1
DEBIASI_APOPTOSIS_BY_REOVIRUS_INFECTIO	-0,127	5E-04	0,00165	1
GNF2_CCNB2	-0,127	5E-04	0,00137	1
MEIOSIS_I	-0,127	5E-04	0,00062	1
SMIRNOV_RESPONSE_TO_IR_6HR_DN	-0,127	5E-04	0,00146	1
MORF_ESPL1	-0,127	5E-04	0,00274	1
KEGG_VALINE_LEUCINE_AND_ISOLEUCINE_DE	0,127	5E-04	0,01542	1
MCCABE_HOXC6_TARGETS_DN	-0,127	5E-04	0,00045	1
V$AP1FJ_Q2	-0,127	5E-04	0,00039	1
PHOSPHOINOSITIDE_MEDIATED_SIGNALING	-0,127	5E-04	0,00018	1
GSE22886_NAIVE_BCELL_VS_BLOOD_PLASM	-0,127	5E-04	0,07271	1
SPLICEOSOME_ASSEMBLY	-0,127	5E-04	0,00471	1

GSE17721_POLYIC_VS_CPG_8H_BMDM_DN	-0,127	5E-04	0,00542	1
PID_PLK1_PATHWAY	-0,127	5E-04	0,00213	1
SANDERSON_PPARA_TARGETS	0,127	5E-04	0,04707	1
CHIARADONNA_NEOPLASTIC_TRANSFORMATI(	-0,127	5E-04	0,0108	1
GNF2_MKI67	-0,127	5E-04	0,00145	1
REACTOME_RNA_POL_II_TRANSCRIPTION	-0,127	5E-04	0,01395	1
NEGATIVE_REGULATION_OF_DEVELOPMENTAI	-0,127	5E-04	0,00024	1
CHEN_ETV5_TARGETS_TESTIS	-0,127	5E-04	0,00601	1
GNF2_CDC20	-0,127	5E-04	0,00158	1
AMINE_CATABOLIC_PROCESS	0,127	5E-04	0,01459	1
GSE36476_CTRL_VS_TSST_ACT_72H_MEMO	-0,127	5E-04	0,00318	1
PHOSPHOTRANSFERASE_ACTIVITY_ALCOHOL_	-0,127	5E-04	0,00031	1
chr3q25	-0,127	5E-04	0,02969	1
PUJANA_BRCA_CENTERED_NETWORK	-0,127	5E-04	0,00267	1
BROWNE_HCMV_INFECTION_18HR_UP	-0,127	5E-04	0,00064	1
GRADE_COLON_AND_RECTAL_CANCER_UP	-0,127	5E-04	0,04014	1
GNF2_RRM2	-0,127	5E-04	0,00165	1
V$MYOGNF1_01	-0,127	5E-04	0,00034	1
ENK_UV_RESPONSE_KERATINOCYTE_DN	-0,127	5E-04	0,0073	1
POSITIVE_REGULATION_OF_CELL_ADHESION	-0,127	5E-04	0,0002	1
GSE3982_MEMORY_CD4_TCELL_VS_TH1_DN	-0,127	5E-04	0,0224	1
GSE34205_HEALTHY_VS_RSV_INF_INFANT_I	-0,127	5E-04	0,02096	1
POSITIVE_REGULATION_OF_HYDROLASE_ACT	-0,127	5E-04	0,00103	1
KYNG_WERNER_SYNDROM_AND_NORMAL_AG	-0,127	5E-04	0,00131	1
GSE17721_CTRL_VS_CPG_8H_BMDM_DN	-0,127	5E-04	0,00035	1
GSE29618_LAIV_VS_TIV_FLU_VACCINE_DAY	-0,127	5E-04	0,00029	1
GSE2826_WT_VS_BTK_KO_BCELL_UP	-0,127	5E-04	0,00046	1
GSE3982_CTRL_VS_PMA_STIM_EOSINOPHIL_	-0,127	5E-04	0,00021	1
XU_HGF_TARGETS_INDUCED_BY_AKT1_48HR	-0,127	5E-04	0,00014	1
NUCLEASE_ACTIVITY	-0,127	5E-04	0,00271	1
GSE32423_CTRL_VS_IL7_IL4_MEMORY_CD8_	-0,127	5E-04	0,00022	1
GSE17721_4H_VS_24H_POLYIC_BMDM_UP	-0,127	5E-04	0,00552	1
RODRIGUES_THYROID_CARCINOMA_POORLY_I	-0,127	5E-04	0,04186	1
GSE17721_CTRL_VS_POLYIC_12H_BMDM_DI	-0,127	5E-04	0,00068	1
VICENT_METASTASIS_UP	-0,127	5E-04	0,00118	1
GU_PDEF_TARGETS_DN	-0,127	5E-04	0,06513	1
GSE8868_SPLEEN_VS_INTESTINE_CD11B_PO	-0,127	5E-04	0,0003	1
BONCI_TARGETS_OF_MIR15A_AND_MIR16_1	-0,127	5E-04	0,00153	1
GSE17721_0.5H_VS_12H_POLYIC_BMDM_UP	-0,126	5E-04	0,00115	1
WORSCHECH_TUMOR_REJECTION_DN	-0,126	5E-04	0,00611	1
GNF2_CKS2	-0,126	5E-04	0,00149	1
REACTOME_PURINE_METABOLISM	-0,126	5E-04	0,04284	1

GNF2_MCM4	-0,126	6E-04	0,00143	1
NUYTTEN_NIPP1_TARGETS_UP	-0,126	6E-04	0,0015	1
PHOSPHOINOSITIDE_BIOSYNTHETIC_PROCESS	0,126	6E-04	0,00647	1
GNF2_ESPL1	-0,126	6E-04	0,00224	1
RIBONUCLEASE_ACTIVITY	-0,126	6E-04	0,02404	1
NUCLEAR_HORMONE_RECEPTOR_BINDING	-0,126	6E-04	0,00628	1
REACTOME_ASSOCIATION_OF_LICENSING_FA(	-0,126	6E-04	0,00247	1
GSE17721_CTRL_VS_PAM3CSK4_12H_BMDN	-0,126	6E-04	0,00449	1
REACTOME_POST_CHAPERONIN_TUBULIN_FOI	-0,126	6E-04	0,00196	1
BERTUCCI_INVASIVE_CARCINOMA_DUCTAL_V	-0,126	6E-04	0,05457	1
REICHERT_MITOSIS_LIN9_TARGETS	-0,126	6E-04	0,00384	1
MODULE_563	0,126	6E-04	0,00938	1
GSE32423_CTRL_VS_IL4_MEMORY_CD8_TCE	-0,126	6E-04	0,00145	1
GSE8868_SPLEEN_VS_INTESTINE_CD11B_PO	-0,126	6E-04	0,00025	1
YAMAZAKI_TCEB3_TARGETS_UP	-0,126	6E-04	0,00047	1
GSE17721_LPS_VS_CPG_24H_BMDM_DN	-0,126	6E-04	0,00174	1
ATAAGCT,MIR-21	-0,126	6E-04	0,00018	1
GNF2_RFC4	-0,126	6E-04	0,00179	1
GRAHAM_CML_DIVIDING_VS_NORMAL_DIVIDII	-0,126	6E-04	0,00097	1
REICHERT_G1S_REGULATORS_AS_PI3K_TARG	-0,126	6E-04	0,0032	1
TERAMOTO_OPN_TARGETS_CLUSTER_7	-0,126	6E-04	0,04988	1
GSE22886_UNSTIM_VS_IL15_STIM_NKCELL_	-0,126	6E-04	0,02094	1
GSE15930_STIM_VS_STIM_AND_TRICHOSTA	-0,126	6E-04	0,0003	1
ZHANG_TLX_TARGETS_UP	-0,126	6E-04	0,00274	1
MACROMOLECULE_BIOSYNTHETIC_PROCESS	-0,126	6E-04	0,00031	1
SENGUPTA_NASOPHARYNGEAL_CARCINOMA_	-0,126	6E-04	0,00166	1
REN_BOUND_BY_E2F	-0,126	6E-04	0,00564	1
GSE32423_IL7_VS_IL4_MEMORY_CD8_TCELI	-0,126	6E-04	0,00055	1
GGCAGTG,MIR-324-3P	-0,125	6E-04	0,0003	1
PID_CASPASE_PATHWAY	-0,125	6E-04	0,00059	1
GSE15733_BM_VS_SPLEEN_MEMORY_CD4_T	-0,125	6E-04	0,00071	1
GSE29618_PRE_VS_DAY7_POST_TIV_FLU_V	-0,125	6E-04	0,0015	1
WEST_ADRENOCORTICAL_TUMOR_MARKERS_	-0,125	6E-04	0,00424	1
INTRINSIC_TO_GOLGI_MEMBRANE	0,125	6E-04	0,00925	1
SUZUKI_AMPLIFIED_IN_ORAL_CANCER	-0,125	6E-04	0,00104	1
CENTROSOME	-0,125	6E-04	0,00214	1
MODULE_204	-0,125	6E-04	0,00226	1
KAMMINGA_EZH2_TARGETS	-0,125	6E-04	0,0027	1
DEVELOPMENTAL_GROWTH	-0,125	6E-04	0,0002	1
LEIN_LOCALIZED_TO_PROXIMAL_DENDRITES	-0,125	6E-04	0,00021	1
DORN_ADENOVIRUS_INFECTION_48HR_UP	-0,125	6E-04	0,00034	1
LOPEZ_MESOTELIOMA_SURVIVAL_TIME_UP	-0,125	6E-04	0,00495	1

MODULE_544	-0,125	6E-04	0,00013	1
GSE17721_0.5H_VS_4H_GARDIQUIMOD_BMD	-0,125	6E-04	0,0015	1
NAKAMURA_METASTASIS	-0,125	6E-04	0,00545	1
POSITIVE_REGULATION_OF_RESPONSE_TO_ST	-0,125	6E-04	0,00105	1
ION_BINDING	-0,125	6E-04	0,0005	1
MCAATNNNNNGCG_UNKNOWN	-0,125	6E-04	0,02703	1
FUJIWARA_PARK2_HEPATOCYTE_PROLIFERAT	-0,125	6E-04	0,00249	1
AACGGTT,MIR-451	-0,125	6E-04	0,00289	1
WONG_IFNA2_RESISTANCE_DN	-0,125	6E-04	0,00015	1
GARGALOVIC_RESPONSE_TO_OXIDIZED_PHOS	-0,125	6E-04	0,00466	1
GSE26669_CTRL_VS_COSTIM_BLOCK_MLR_C	-0,125	6E-04	0,01788	1
KEGG_GLYCOSPHINGOLIPID_BIOSYNTHESIS_LA	-0,125	6E-04	0,03222	1
chr11p14	-0,125	6E-04	0,00076	1
PHOSPHOLIPASE_C_ACTIVATION	-0,125	6E-04	0,00275	1
VANHARANTA_UTERINE_FIBROID_WITH_7Q_D	-0,125	6E-04	0,00254	1
KANG_DOXORUBICIN_RESISTANCE_UP	-0,125	6E-04	0,00132	1
WINTER_HYPOXIA_UP	-0,125	6E-04	0,08723	1
NAKAMURA_ADIPOGENESIS_EARLY_UP	-0,125	6E-04	0,00023	1
ALONSO_METASTASIS_NEURAL_UP	-0,125	6E-04	0,00601	1
GSE16522_MEMORY_VS_NAIVE_CD8_TCELL_	-0,125	6E-04	0,00114	1
GSE17721_PAM3CSK4_VS_CPG_8H_BMDM_U	-0,125	7E-04	0,01144	1
MORF_CDC10	-0,125	7E-04	0,03543	1
MORF_AATF	-0,125	7E-04	0,04431	1
V$AP1_Q4_01	-0,125	7E-04	0,00025	1
BURTON_ADIPOGENESIS_7	-0,124	7E-04	0,00079	1
SINGLE_STRANDED_DNA_BINDING	-0,124	7E-04	0,00308	1
GSE39820_CTRL_VS_TGFBETA3_IL6_CD4_T	-0,124	7E-04	0,00392	1
PID_P53DOWNSTREAMPATHWAY	-0,124	7E-04	0,00039	1
GSE15750_DAY6_VS_DAY10_EFF_CD8_TCEL	-0,124	7E-04	0,00262	1
REACTOME_METABOLISM_OF_MRNA	-0,124	7E-04	0,00208	1
PID_MET_PATHWAY	-0,124	7E-04	0,00471	1
KAECH_DAY8_EFF_VS_MEMORY_CD8_TCELL_	-0,124	7E-04	0,01075	1
TAKAYAMA_BOUND_BY_AR	-0,124	7E-04	0,00017	1
EXTRACELLULAR_MATRIX_STRUCTURAL_CONS	-0,124	7E-04	0,00021	1
MODULE_501	-0,124	7E-04	0,00057	1
AZARE_NEOPLASTIC_TRANSFORMATION_BY_S	-0,124	7E-04	0,00714	1
MODULE_189	-0,124	7E-04	0,03547	1
GSE29618_MONOCYTE_VS_MDC_DAY7_FLU_	-0,124	7E-04	0,00055	1
KAUFFMANN_DNA_REPLICATION_GENES	-0,124	7E-04	0,00233	1
GSE22886_NAIVE_CD4_TCELL_VS_12H_ACT	-0,124	7E-04	0,05685	1
DELYS_THYROID_CANCER_UP	-0,124	7E-04	0,00361	1
LIM_MAMMARY_LUMINAL_PROGENITOR_UP	-0,124	7E-04	0,01506	1

GSE17974_CTRL_VS_ACT_IL4_AND_ANTI_IL	-0,124	7E-04	0,00042	1
DEVELOPMENT_OF_PRIMARY_SEXUAL_CHARA	-0,124	7E-04	0,00071	1
GSE12366_PLASMA_CELL_VS_NAIVE_BCELL_	-0,124	7E-04	0,00013	1
WINZEN_DEGRADED_VIA_KHSRP	-0,124	7E-04	0,00044	1
YAGI_AML_SURVIVAL	-0,124	7E-04	0,00062	1
WANG_ESOPHAGUS_CANCER_PROGRESSION_	-0,124	7E-04	0,01915	1
PROTEIN_TRANSPORTER_ACTIVITY	0,124	7E-04	0,06243	1
STEARMAN_LUNG_CANCER_EARLY_VS_LATE_	-0,124	7E-04	0,00114	1
GSE14308_NAIVE_CD4_TCELL_VS_INDUCED_	-0,124	7E-04	0,00125	1
BENPORATH_ES_CORE_NINE	-0,124	7E-04	0,02333	1
GSE17721_CTRL_VS_LPS_1H_BMDM_DN	-0,124	7E-04	0,00034	1
GGGNNTTTCC_V$NFKB_Q6_01	-0,124	7E-04	0,00036	1
GSE7852_LN_VS_THYMUS_TCONV_UP	-0,124	7E-04	0,00026	1
CYTOPLASMIC_VESICLE_MEMBRANE	-0,124	7E-04	0,01126	1
CYTOPLASMIC_VESICLE_PART	-0,124	7E-04	0,01126	1
YAO_TEMPORAL_RESPONSE_TO_PROGESTERO	-0,124	7E-04	0,00156	1
chr3q28	-0,124	7E-04	0,02212	1
PECE_MAMMARY_STEM_CELL_DN	-0,124	7E-04	0,0048	1
GSE22886_CD4_TCELL_VS_BCELL_NAIVE_D	-0,124	7E-04	0,00023	1
GEORGES_TARGETS_OF_MIR192_AND_MIR21	-0,124	7E-04	0,00493	1
IRITANI_MAD1_TARGETS_DN	-0,124	7E-04	0,00927	1
VANTVEER_BREAST_CANCER_METASTASIS_D	-0,124	7E-04	0,04512	1
GSE360_DC_VS_MAC_DN	-0,124	7E-04	0,0007	1
CRX_DN.V1_DN	-0,124	7E-04	0,00015	1
ALONSO_METASTASIS_EMT_UP	-0,124	7E-04	0,00049	1
GNF2_BUB1B	-0,124	7E-04	0,00274	1
GSE17721_LPS_VS_CPG_2H_BMDM_UP	-0,124	7E-04	0,00068	1
MODULE_104	-0,124	7E-04	0,00043	1
MODULE_389	-0,123	7E-04	0,00047	1
GCM_RAF1	-0,123	7E-04	0,00108	1
GSE17721_PAM3CSK4_VS_CPG_1H_BMDM_I	-0,123	7E-04	0,00018	1
MODULE_229	-0,123	7E-04	0,00211	1
LINDVALL_IMMORTALIZED_BY_TERT_DN	-0,123	7E-04	0,00024	1
PID_P38ALPHABETAPATHWAY	-0,123	7E-04	0,00053	1
V$BACH2_01	-0,123	7E-04	0,0009	1
MODULE_543	-0,123	7E-04	0,00256	1
MODULE_17	-0,123	7E-04	0,02218	1
CHEN_HOXA5_TARGETS_9HR_DN	-0,123	7E-04	0,00499	1
GSE31082_DP_VS_CD4_SP_THYMOCYTE_UP	-0,123	7E-04	0,00334	1
RB_DN.V1_UP	-0,123	7E-04	0,00118	1
KRAS.300_UP.V1_DN	-0,123	7E-04	0,00115	1
POSITIVE_REGULATION_OF_BIOLOGICAL_PRO	-0,123	7E-04	0,00015	1

BIOCARTA_BCELLSURVIVAL_PATHWAY	-0,123	7E-04	0,00111	1
DNA_DAMAGE_CHECKPOINT	-0,123	7E-04	0,00463	1
HELLER_HDAC_TARGETS_SILENCED_BY_METI	-0,123	7E-04	0,00213	1
SCHAEFFER_PROSTATE_DEVELOPMENT_AND_	-0,123	7E-04	0,00055	1
NUCLEOSOME_ASSEMBLY	-0,123	7E-04	0,00034	1
V$MEIS1AHOXA9_01	-0,123	8E-04	0,00015	1
SPINDLE	-0,123	8E-04	0,00911	1
ORGAN_MORPHOGENESIS	-0,123	8E-04	0,00044	1
PID_ATM_PATHWAY	-0,123	8E-04	0,00212	1
NUCLEOCYTOPLASMIC_TRANSPORT	-0,123	8E-04	0,02138	1
MORF_DEAF1	-0,123	8E-04	0,03051	1
GSE7764_IL15_TREATED_VS_CTRL_NK_CELI	-0,123	8E-04	0,00506	1
MODULE_545	-0,123	8E-04	0,00762	1
chr16q13	-0,123	8E-04	0,00944	1
CHEOK_RESPONSE_TO_MERCAPTOPURINE_DN	-0,123	8E-04	0,00179	1
MCBRYAN_TERMINAL_END_BUD_DN	-0,123	8E-04	0,00176	1
BAFNA_MUC4_TARGETS_DN	-0,123	8E-04	0,02641	1
KYNG_RESPONSE_TO_H2O2_VIA_ERCC6_UP	-0,123	8E-04	0,00142	1
BIOCARTA_INTEGRIN_PATHWAY	-0,123	8E-04	0,00654	1
BROWNE_HCMV_INFECTION_20HR_UP	-0,123	8E-04	0,00027	1
SATO_SILENCED_BY_METHYLATION_IN_PANC	-0,123	8E-04	0,0003	1
MODULE_267	-0,123	8E-04	0,00016	1
SARTIPY_NORMAL_AT_INSULIN_RESISTANCE_	-0,123	8E-04	0,0057	1
GSE18791_CTRL_VS_NEWCASTLE_VIRUS_DC	-0,123	8E-04	0,00562	1
GSE360_L_DONOVANI_VS_L_MAJOR_MAC_U	-0,123	8E-04	0,00077	1
ABE_VEGFA_TARGETS_2HR	-0,123	8E-04	0,00462	1
FRIDMAN_SENESCENCE_UP	-0,123	8E-04	0,0007	1
PARK_OSTEOBLAST_DIFFERENTIATION_BY_PI	-0,123	8E-04	0,00563	1
chr9q21	-0,123	8E-04	0,00414	1
MORF_IFNA1	-0,123	8E-04	0,00026	1
KEGG_EPITHELIAL_CELL_SIGNALING_IN_HELIC	-0,123	8E-04	0,01682	1
MYELOID_CELL_DIFFERENTIATION	-0,123	8E-04	0,00057	1
SULFUR_METABOLIC_PROCESS	-0,123	8E-04	0,00251	1
REACTOME_INTERACTIONS_OF_VPR_WITH_H(	-0,123	8E-04	0,01623	1
GOTZMANN_EPITHELIAL_TO_MESENCHYMAL_	-0,123	8E-04	0,01957	1
chr6p12	-0,123	8E-04	0,00369	1
TRANSCRIPTION_ELONGATION_REGULATOR_A	-0,123	8E-04	0,00466	1
GRAESSMANN_APOPTOSIS_BY_SERUM_DEPRIV	-0,123	8E-04	0,00014	1
LA_MEN1_TARGETS	-0,123	8E-04	0,00019	1
OXFORD_RALA_OR_RALB_TARGETS_UP	-0,123	8E-04	0,0053	1
ROSTY_CERVICAL_CANCER_PROLIFERATION_(	-0,123	8E-04	0,00283	1
ZWANG_CLASS_1_TRANSIENTLY_INDUCED_B	-0,123	8E-04	0,00023	1

PHOSPHATASE_REGULATOR_ACTIVITY	-0,123	8E-04	0,0008	1
chr18q21	-0,123	8E-04	0,00196	1
SOLUBLE_FRACTION	-0,123	8E-04	0,00049	1
SPLICEOSOME	-0,123	8E-04	0,00774	1
PHONG_TNF_RESPONSE_VIA_P38_COMPLETE	-0,123	8E-04	0,0005	1
MODULE_318	-0,123	8E-04	0,00378	1
AMUNDSON_GAMMA_RADIATION_RESPONSE	-0,122	8E-04	0,00263	1
CAFFAREL_RESPONSE_TO_THC_24HR_3_DN	-0,122	8E-04	0,00983	1
GCM_SMARCD1	-0,122	8E-04	0,00013	1
MODULE_481	-0,122	8E-04	0,00014	1
MODULE_33	-0,122	8E-04	0,00027	1
ODONNELL_TARGETS_OF_MYC_AND_TFRC_D	-0,122	8E-04	0,00559	1
REGULATION_OF_MOLECULAR_FUNCTION	-0,122	8E-04	0,00018	1
GSE17721_CPG_VS_GARDIQUIMOD_8H_BMDI	-0,122	8E-04	0,00057	1
PID_GLYPICAN_1PATHWAY	-0,122	8E-04	0,00024	1
AGCATTA,MIR-155	-0,122	8E-04	0,00084	1
REACTOME_PROTEIN_FOLDING	-0,122	8E-04	0,02195	1
TRACEY_RESISTANCE_TO_IFNA2_UP	-0,122	8E-04	0,00515	1
YANAGIHARA_ESX1_TARGETS	-0,122	8E-04	0,00038	1
HINATA_NFKB_TARGETS_FIBROBLAST_UP	-0,122	8E-04	0,01264	1
GSE36476_CTRL_VS_TSST_ACT_40H_MEMO	-0,122	8E-04	0,00726	1
GCM_RAD21	-0,122	8E-04	0,03599	1
MANTOVANI_VIRAL_GPCR_SIGNALING_DN	-0,122	8E-04	0,00101	1
REACTOME_TIE2_SIGNALING	-0,122	8E-04	0,0044	1
NUCLEAR_TRANSPORT	-0,122	8E-04	0,02223	1
WU_HBX_TARGETS_2_UP	-0,122	8E-04	0,00841	1
MARTORIATI_MDM4_TARGETS_FETAL_LIVER_	-0,122	8E-04	0,00221	1
SHETH_LIVER_CANCER_VS_TXNIP_LOSS_PAM	-0,122	8E-04	0,00062	1
MULLIGHAN_NPM1_SIGNATURE_3_DN	-0,122	9E-04	0,00014	1
GSE12845_NAIVE_VS_PRE_GC_TONSIL_BCEL	-0,122	9E-04	0,03888	1
ZHAN_V1_LATE_DIFFERENTIATION_GENES_DI	-0,122	9E-04	0,00288	1
KRAS.50_UP.V1_UP	-0,122	9E-04	0,00061	1
MORF_EIF4E	-0,122	9E-04	0,07785	1
GSE13306_LAMINA_PROPRIA_VS_SPLEEN_TI	-0,122	9E-04	0,00097	1
ONDER_CDH1_TARGETS_3_DN	-0,122	9E-04	0,02736	1
BIOCARTA_CCR3_PATHWAY	-0,122	9E-04	0,018	1
HOFMANN_CELL_LYMPHOMA_DN	-0,122	9E-04	0,0005	1
NUCLEAR_MEMBRANE_PART	-0,122	9E-04	0,05224	1
ZHAN_EARLY_DIFFERENTIATION_GENES_DN	-0,122	9E-04	0,00114	1
ZHAN_MULTIPLE_MYELOMA_CD1_VS_CD2_DI	-0,122	9E-04	0,0028	1
HWANG_PROSTATE_CANCER_MARKERS	0,122	9E-04	0,01731	1
BENPORATH_PROLIFERATION	-0,122	9E-04	0,01585	1

SESTO_RESPONSE_TO_UV_C4	-0,122	9E-04	0,11666	1
GSE3982_DC_VS_NKCELL_DN	-0,122	9E-04	0,00018	1
GNF2_XRCC5	-0,122	9E-04	0,10359	1
GSE27786_LSK_VS_LIN_NEG_CELL_DN	-0,122	9E-04	0,00032	1
REACTOME_GLYCOSAMINOGLYCAN_METABOL	-0,121	9E-04	0,00088	1
GSE30962_ACUTE_VS_CHRONIC_LCMV_SECC	-0,121	9E-04	0,01658	1
YANG_BREAST_CANCER_ESR1_LASER_UP	0,121	9E-04	0,10783	1
EXTRACELLULAR_REGION_PART	-0,121	9E-04	0,00017	1
MODULE_439	-0,121	9E-04	0,00672	1
REACTOME_LATE_PHASE_OF_HIV_LIFE_CYCL	-0,121	9E-04	0,01888	1
WALLACE_PROSTATE_CANCER_DN	-0,121	9E-04	0,00052	1
MORF_RFC4	-0,121	9E-04	0,03	1
MARSON_FOXP3_TARGETS_DN	-0,121	9E-04	0,00037	1
MYLLYKANGAS_AMPLIFICATION_HOT_SPOT_8	-0,121	9E-04	0,00124	1
HOMEOSTASIS_OF_NUMBER_OF_CELLS	-0,121	9E-04	0,00236	1
GSE29618_BCELL_VS_MONOCYTE_DAY7_FLU	-0,121	9E-04	0,00048	1
CYCLIN_D1_KE_.V1_UP	-0,121	9E-04	0,00025	1
HOFFMANN_SMALL_PRE_BII_TO_IMMATURE_B	-0,121	9E-04	0,0002	1
CHOW_RASSF1_TARGETS_UP	-0,121	9E-04	0,0038	1
HAN_JNK_SINGALING_DN	-0,121	9E-04	0,0006	1
ULE_SPLICING_VIA_NOVA2	-0,121	9E-04	0,001	1
GENTILE_UV_RESPONSE_CLUSTER_D4	-0,121	9E-04	0,01194	1
ACATATC,MIR-190	-0,121	9E-04	0,00389	1
MORF_MLLT10	-0,121	9E-04	0,00014	1
DURCHDEWALD_SKIN_CARCINOGENESIS_DN	-0,121	9E-04	0,00302	1
REACTOME_KINESINS	-0,121	9E-04	0,00394	1
MODULE_159	-0,121	9E-04	0,01674	1
REACTOME_POST_TRANSLATIONAL_MODIFICA	0,121	9E-04	0,00941	1
SCHUHMACHER_MYC_TARGETS_UP	-0,121	9E-04	0,05261	1
GARY_CD5_TARGETS_DN	-0,121	9E-04	0,03033	1
LE_NEURONAL_DIFFERENTIATION_UP	-0,121	9E-04	0,00053	1
PEPTIDYL_TYROSINE_MODIFICATION	-0,121	9E-04	0,00162	1
AMINE_BIOSYNTHETIC_PROCESS	-0,121	1E-03	0,00015	1
KEGG_AXON_GUIDANCE	-0,121	1E-03	0,00014	1
ZHAN_MULTIPLE_MYELOMA_CD1_VS_CD2_UI	-0,121	1E-03	0,00099	1
TERAO_AOX4_TARGETS_HG_DN	-0,121	1E-03	0,0031	1
PID_P38_MK2PATHWAY	-0,121	1E-03	0,01663	1
HUANG_DASATINIB_RESISTANCE_UP	-0,121	1E-03	0,00614	1
GSE22886_TCELL_VS_BCELL_NAIVE_DN	-0,121	1E-03	0,0006	1
GSE17721_CTRL_VS_PAM3CSK4_6H_BMDM_	-0,121	1E-03	0,01651	1
chr13q22	-0,121	1E-03	0,00028	1
POSITIVE_REGULATION_OF_CYTOKINE_PRODU	-0,121	1E-03	0,0018	1

KONG_E2F1_TARGETS	-0,12	1E-03	0,06166	1
CHIANG_LIVER_CANCER_SUBCLASS_POLYSOM	-0,12	1E-03	0,01881	1
CROSBY_E2F4_TARGETS	-0,12	1E-03	0,00586	1
REGULATION_OF_PROGRAMMED_CELL_DEATH	-0,12	1E-03	0,00147	1
GSE36392_EOSINOPHIL_VS_NEUTROPHIL_IL2	-0,12	1E-03	0,00359	1
GSE3982_NEUTROPHIL_VS_TH1_UP	-0,12	1E-03	0,0013	1
ONE_CARBON_COMPOUND_METABOLIC_PROC	-0,12	1E-03	0,00644	1
KRAS.LUNG.BREAST_UP.V1_DN	-0,12	1E-03	0,0002	1
MORF_CCNF	-0,12	1E-03	0,00987	1
SASAI_TARGETS_OF_CXCR6_AND_PTCH1_UP	-0,12	1E-03	0,01846	1
GSE22886_NEUTROPHIL_VS_DC_UP	-0,12	1E-03	0,00027	1
AACTGGA,MIR-145	-0,12	0,001	0,00041	1
MORF_RAD23A	-0,12	0,001	0,04495	1
GSE27786_CD4_VS_CD8_TCELL_DN	-0,12	0,001	0,00076	1
REACTOME_METABOLISM_OF_CARBOHYDRATI	-0,12	0,001	0,00761	1
BILD_CTNNB1_ONCOGENIC_SIGNATURE	-0,12	0,001	0,00054	1
PASQUALUCCI_LYMPHOMA_BY_GC_STAGE_UI	-0,12	0,001	0,00231	1
GSE17974_2.5H_VS_72H_IL4_AND_ANTI_IL	-0,12	0,001	0,00934	1
WANG_ESOPHAGUS_CANCER_VS_NORMAL_D	-0,12	0,001	0,00534	1
PID_S1P_S1P4_PATHWAY	-0,12	0,001	0,00119	1
V$CDPCR3_01	-0,12	0,001	0,00114	1
ZHU_SKIL_TARGETS_DN	-0,12	0,001	0,00498	1
SASAKI_TARGETS_OF_TP73_AND_TP63	-0,12	0,001	0,00208	1
GSE17721_LPS_VS_GARDIQUIMOD_2H_BMDM	-0,12	0,001	0,00013	1
NIELSEN_LIPOSARCOMA_DN	-0,12	0,001	0,07273	1
CONDENSED_NUCLEAR_CHROMOSOME	-0,12	0,001	0,0052	1
LI_WILMS_TUMOR	-0,12	0,001	0,0009	1
GSE17721_CPG_VS_GARDIQUIMOD_16H_BMI	-0,12	0,001	0,0006	1
MODULE_138	-0,12	0,001	0,00025	1
V$NFKAPPAB_01	-0,12	0,001	0,00106	1
CAFFAREL_RESPONSE_TO_THC_8HR_5_DN	-0,12	0,001	0,01238	1
HE_PTEN_TARGETS_DN	-0,12	0,001	0,00109	1
CTATGCA,MIR-153	-0,12	0,001	0,00067	1
V$CEBPB_02	-0,12	0,001	0,00048	1
KEGG_RNA_POLYMERASE	-0,12	0,001	0,02398	1
DIAZ_CHRONIC_MEYLOGENOUS_LEUKEMIA_UP	-0,12	0,001	0,05314	1
REACTOME_RECRUITMENT_OF_MITOTIC_CENT	-0,12	0,001	0,00433	1
SHIPP_DLBCL_CURED_VS_FATAL_UP	-0,12	0,001	0,00156	1
BIOCARTA_ECM_PATHWAY	-0,12	0,001	0,0084	1
PENG_LEUCINE_DEPRIVATION_DN	-0,12	0,001	0,08415	1
CLASPER_LYMPHATIC_VESSELS_DURING_MET	-0,12	0,001	0,00045	1
PROTEIN_IMPORT_INTO_NUCLEUS	-0,12	0,001	0,02575	1

GSE17974_IL4_AND_ANTI_IL12_VS_UNTREA	-0,12	0,001	0,00028	1
WESTON_VEGFA_TARGETS_12HR	-0,12	0,001	0,00015	1
MODULE_377	0,12	0,001	0,01113	1
AGGAGTG,MIR-483	-0,12	0,001	0,00027	1
DAVICIONI_TARGETS_OF_PAX_FOXO1_FUSIOI	-0,12	0,001	0,00017	1
GSE15659_NAIVE_VS_PTPRC_NEG_CD4_TCE	-0,119	0,001	0,00299	1
SHEN_SMARCA2_TARGETS_DN	-0,119	0,001	0,00512	1
GSE12845_IGD_POS_VS_NEG_BLOOD_BCELL	-0,119	0,001	0,09324	1
GNF2_PA2G4	-0,119	0,001	0,04786	1
ARYLSULFATASE_ACTIVITY	-0,119	0,001	0,00133	1
TANG_SENESCENCE_TP53_TARGETS_UP	-0,119	0,001	0,0004	1
GGCACAT,MIR-455	-0,119	0,001	0,00085	1
GSE15930_NAIVE_VS_72H_IN_VITRO_STIM_	-0,119	0,001	0,01115	1
ROME_INSULIN_TARGETS_IN_MUSCLE_DN	-0,119	0,001	0,00013	1
REACTOME_PEPTIDE_LIGAND_BINDING_RECEP	-0,119	0,001	0,00047	1
SIALYLTRANSFERASE_ACTIVITY	-0,119	0,001	0,00131	1
BOUDOUKHA_BOUND_BY_IGF2BP2	-0,119	0,001	0,01	1
REACTOME_HEPARAN_SULFATE_HEPARIN_HS	-0,119	0,001	0,00105	1
SEIDEN_ONCOGENESIS_BY_MET	-0,119	0,001	0,02947	1
BIOCARTA_MYOSIN_PATHWAY	-0,119	0,001	0,00023	1
GSE27786_LSK_VS_NEUTROPHIL_DN	-0,119	0,001	0,00016	1
MIKKELSEN_ES_HCP_WITH_H3_UNMETHYLAT	-0,119	0,001	0,00013	1
SOTIRIOU_BREAST_CANCER_GRADE_1_VS_3_	-0,119	0,001	0,00628	1
ACEVEDO_FGFR1_TARGETS_IN_PROSTATE_C.	-0,119	0,001	0,00677	1
REACTOME_DEGRADATION_OF_THE_EXTRACE	-0,119	0,001	0,00068	1
REACTOME_SIGNALING_BY_CONSTITUTIVELY_	-0,119	0,001	0,01593	1
chr10p12	-0,119	0,001	0,00295	1
KUMAMOTO_RESPONSE_TO_NUTLIN_3A_DN	-0,119	0,001	0,0051	1
SISTER_CHROMATID_SEGREGATION	-0,119	0,001	0,00919	1
EMBRYONIC_DEVELOPMENT	-0,119	0,001	0,00027	1
TIEN_INTESTINE_PROBIOTICS_24HR_UP	-0,119	0,001	0,04434	1
P53_DN.V1_DN	-0,119	0,001	0,00017	1
ROSS_AML_OF_FAB_M7_TYPE	-0,119	0,001	0,0007	1
MRNA_SPLICE_SITE_SELECTION	-0,119	0,001	0,00171	1
MELLMAN_TUT1_TARGETS_DN	-0,119	0,001	0,01092	1
REGULATION_OF_APOPTOSIS	-0,119	0,001	0,00184	1
REACTOME_TRANSPORT_OF_VITAMINS_NUCL	-0,119	0,001	0,00854	1
GSE15930_STIM_VS_STIM_AND_IL-12_48H_	-0,119	0,001	0,00142	1
IM_SREBF1A_TARGETS	0,119	0,001	0,13268	1
TIEN_INTESTINE_PROBIOTICS_2HR_UP	-0,119	0,001	0,00118	1
GSE3982_EOSINOPHIL_VS_BASOPHIL_UP	-0,119	0,001	0,00083	1
NUYTTEN_NIPP1_TARGETS_DN	-0,118	0,001	0,00628	1

SU_TESTIS	-0,118	0,001	0,01788	1
PETROVA_ENDOTHELIUM_LYMPHATIC_VS_BL(	-0,118	0,001	0,0029	1
PID_MAPKTRKPATHWAY	-0,118	0,001	0,00052	1
chr12p11	-0,118	0,001	0,0026	1
GNF2_EIF3S6	-0,118	0,001	0,00105	1
MORF_CDH4	-0,118	0,001	0,00023	1
SA_G2_AND_M_PHASES	-0,118	0,001	0,01229	1
VERRECCHIA_RESPONSE_TO_TGFB1_C4	-0,118	0,001	0,00956	1
POS_RESPONSE_TO_HISTAMINE_DN	-0,118	0,001	0,02021	1
GOTTWEIN_TARGETS_OF_KSHV_MIR_K12_11	-0,118	0,001	0,00064	1
ANION_TRANSMEMBRANE_TRANSPORTER_AC	-0,118	0,001	0,00016	1
CHEN_HOXA5_TARGETS_9HR_UP	-0,118	0,001	0,00053	1
GNF2_BUB3	-0,118	0,001	0,04321	1
GSE13306_TREG_VS_TCONV_LAMINA_PROPI	-0,118	0,001	0,00015	1
GSE18791_UNSTIM_VS_NEWCATSLE_VIRUS_	-0,118	0,001	0,00411	1
INTEGRIN_COMPLEX	-0,118	0,001	0,00159	1
RHODES_CANCER_META_SIGNATURE	-0,118	0,001	0,03043	1
KEGG_PROSTATE_CANCER	-0,118	0,001	0,00013	1
TRAYNOR_RETT_SYNDROM_UP	-0,118	0,001	0,00079	1
GSE17721_POLYIC_VS_PAM3CSK4_16H_BMI	-0,118	0,001	0,00178	1
BASSO_B_LYMPHOCYTE_NETWORK	-0,118	0,001	0,0237	1
GOLDRATH_ANTIGEN_RESPONSE	-0,118	0,001	0,00949	1
MARCINIAK_ER_STRESS_RESPONSE_VIA_CHO	-0,118	0,001	0,02615	1
KEGG_PEROXISOME	0,118	0,001	0,01459	1
GERHOLD_ADIPOGENESIS_DN	-0,118	0,001	0,00183	1
MORF_IL16	-0,118	0,001	0,00051	1
GSE12845_IGD_POS_BLOOD_VS_NAIVE_TON	-0,118	0,001	0,005	1
AZARE_NEOPLASTIC_TRANSFORMATION_BY_!	-0,118	0,001	0,00223	1
RB_P107_DN.V1_DN	-0,118	0,001	0,1558	1
REACTOME_CHONDROITIN_SULFATE_DERMAT	-0,118	0,001	0,0008	1
YIH_RESPONSE_TO_ARSENITE_C4	-0,118	0,001	0,02722	1
GSE19825_CD24LOW_VS_IL2RA_HIGH_DAY3	-0,118	0,001	0,04833	1
KEGG_FC_GAMMA_R_MEDIATED_PHAGOCYTO	-0,118	0,001	0,00364	1
MORF_BMPR2	-0,118	0,001	0,00209	1
GSE14769_20MIN_VS_360MIN_LPS_BMDM_I	-0,118	0,001	0,0009	1
chr15q13	-0,118	0,001	0,00127	1
SLEBOS_HEAD_AND_NECK_CANCER_WITH_H	-0,118	0,001	0,02975	1
PID_ERBB2ERBB3PATHWAY	-0,118	0,001	0,00034	1
XU_GH1_EXOGENOUS_TARGETS_DN	-0,118	0,001	0,0002	1
PTEN_DN.V2_UP	-0,118	0,001	0,00039	1
WOO_LIVER_CANCER_RECURRENCE_UP	-0,118	0,001	0,00055	1
WANG_TARGETS_OF_MLL_CBP_FUSION_DN	-0,118	0,001	0,15605	1

REACTOME_DNA_REPLICATION	-0,118	0,001	0,01386	1
REACTOME_REGULATION_OF_MITOTIC_CELL_	-0,118	0,001	0,03462	1
MARSHALL_VIRAL_INFECTION_RESPONSE_DN	-0,118	0,001	0,00055	1
GSE17974_0.5H_VS_72H_UNTREATED_IN_V	-0,118	0,001	0,01012	1
KEGG_RNA_DEGRADATION	-0,118	0,001	0,02884	1
MCBRYAN_PUBERTAL_TGFB1_TARGETS_UP	-0,118	0,001	0,00013	1
GSE1460_CD4_THYMOCYTE_VS_NAIVE_CD4_	-0,118	0,001	0,02455	1
TOMIDA_LUNG_CANCER_POOR_SURVIVAL	-0,118	0,001	0,03316	1
GSE26928_NAIVE_VS_CENT_MEMORY_CD4_	-0,117	0,001	0,0021	1
LEE_LIVER_CANCER_DENA_UP	-0,117	0,001	0,0185	1
PID_PDGFRBPATHWAY	-0,117	0,001	0,00194	1
MOHANKUMAR_TLX1_TARGETS_UP	-0,117	0,001	0,00019	1
MORF_CAMK4	-0,117	0,001	0,00018	1
PROGRAMMED_CELL_DEATH	-0,117	0,001	0,001	1
VANTVEER_BREAST_CANCER_POOR_PROGNO	-0,117	0,001	0,00105	1
MODULE_219	-0,117	0,001	0,0257	1
REGULATION_OF_INTRACELLULAR_TRANSPOR	-0,117	0,001	0,01362	1
GSE17721_POLYIC_VS_PAM3CSK4_1H_BMDI	-0,117	0,001	0,00129	1
GSE17580_TREG_VS_TEFF_S_MANSONI_INF_	-0,117	0,001	0,00013	1
GGGCATT,MIR-365	-0,117	0,001	0,00098	1
WOOD_EBV_EBNA1_TARGETS_UP	-0,117	0,001	0,0069	1
PROTEIN_KINASE_INHIBITOR_ACTIVITY	-0,117	0,001	0,00014	1
REACTOME_E2F_MEDIATED_REGULATION_OF_	-0,117	0,001	0,01144	1
GSE24102_GRANULOCYSTIC_MDSC_VS_NEUT	-0,117	0,001	0,00887	1
GSE15930_STIM_VS_STIM_AND_IFNAB_48H_	-0,117	0,001	0,00214	1
WANG_BARRETTS_ESOPHAGUS_DN	-0,117	0,001	0,05689	1
BIOCARTA_RARRXR_PATHWAY	0,117	0,001	0,00994	1
ST_PAC1_RECEPTOR_PATHWAY	0,117	0,001	0,05073	1
chr4q31	-0,117	0,001	0,00061	1
TGCTGCT,MIR-15A,MIR-16,MIR-15B,MIR-195,M	-0,117	0,001	0,00016	1
MODULE_312	-0,117	0,001	0,02167	1
GSE36476_CTRL_VS_TSST_ACT_40H_MEMO	-0,117	0,001	0,02452	1
ZHAN_MULTIPLE_MYELOMA_MF_UP	-0,117	0,001	0,00079	1
REACTOME_PURINE_CATABOLISM	-0,117	0,001	0,04813	1
PEART_HDAC_PROLIFERATION_CLUSTER_DN	-0,117	0,001	0,01071	1
PID_PI3KCIAKTPATHWAY	-0,117	0,001	0,01553	1
SHAFFER_IRF4_TARGETS_IN_MYELOMA_VS_N	-0,117	0,001	0,041	1
HADDAD_B_LYMPHOCYTE_PROGENITOR	-0,117	0,001	0,00047	1
GRADE_COLON_CANCER_UP	-0,117	0,001	0,00815	1
SESTO_RESPONSE_TO_UV_C0	-0,117	0,001	0,06547	1
GSE13229_MATURE_VS_INTMATURE_NKCELL	-0,117	0,001	0,00015	1
ATF2_UP.V1_UP	-0,117	0,001	0,00019	1

INORGANIC_ANION_TRANSPORT	-0,117	0,001	0,0011	1
REACTOME_INFLUENZA_LIFE_CYCLE	-0,117	0,001	0,00169	1
BRUINS_UVC_RESPONSE_EARLY_LATE	-0,117	0,001	0,0004	1
KEGG_LEUKOCYTE_TRANSENDOTHELIAL_MIGI	-0,117	0,001	0,00145	1
GSE17974_CTRL_VS_ACT_IL4_AND_ANTI_IL	-0,117	0,001	0,0462	1
GSE27786_NKCELL_VS_MONO_MAC_DN	-0,117	0,001	0,00052	1
RNA_SPLICING_FACTOR_ACTIVITYTRANSESTE	-0,117	0,001	0,05386	1
PURBEY_TARGETS_OF_CTBP1_NOT_SATB1_[	-0,117	0,001	0,00057	1
CASTELLANO_HRAS_TARGETS_DN	-0,117	0,001	0,00356	1
PID_INTEGRIN3_PATHWAY	-0,117	0,001	0,00652	1
LEARNING_AND_OR_MEMORY	-0,117	0,001	0,00409	1
GSE339_CD8POS_VS_CD4CD8DN_DC_DN	-0,117	0,001	0,00077	1
REACTOME_MITOTIC_PROMETAPHASE	-0,117	0,001	0,01142	1
YU_BAP1_TARGETS	-0,117	0,001	0,01954	1
SASSON_RESPONSE_TO_GONADOTROPHINS_[	-0,117	0,001	0,00046	1
MORF_BUB1	-0,117	0,001	0,01806	1
GSE26495_PD1HIGH_VS_PD1LOW_CD8_TCEI	-0,117	0,001	0,00014	1
HASINA_NOL7_TARGETS_DN	-0,116	0,001	0,0479	1
MODULE_166	-0,116	0,001	0,00031	1
WHITFIELD_CELL_CYCLE_LITERATURE	-0,116	0,001	0,00686	1
TUBE_DEVELOPMENT	-0,116	0,001	0,00026	1
LAU_APOPTOSIS_CDKN2A_UP	-0,116	0,001	0,00359	1
JAZAERI_BREAST_CANCER_BRCA1_VS_BRCA	-0,116	0,001	0,16558	1
KERATINOCYTE_DIFFERENTIATION	-0,116	0,001	0,02648	1
TAKEDA_TARGETS_OF_NUP98_HOXA9_FUSIC	-0,116	0,001	0,00021	1
REACTOME_A_TETRASACCHARIDE_LINKER_SI	-0,116	0,001	0,0018	1
DARWICHE_SKIN_TUMOR_PROMOTER_DN	-0,116	0,001	0,00058	1
GSE17721_LPS_VS_GARDIQUIMOD_2H_BMDN	-0,116	0,001	0,0005	1
MORF_EIF3S2	-0,116	0,001	0,05411	1
GSE3982_CENT_MEMORY_CD4_TCELL_VS_TI	-0,116	0,001	0,02652	1
THUM_MIR21_TARGETS_HEART_DISEASE_DN	-0,116	0,001	0,00289	1
GSE5960_TH1_VS_ANERGIC_TH1_UP	-0,116	0,001	0,00017	1
GSE13411_NAIVE_VS_MEMORY_BCELL_DN	-0,116	0,001	0,00336	1
GSE3982_MAST_CELL_VS_BASOPHIL_UP	-0,116	0,001	0,00523	1
HALMOS_CEBPA_TARGETS_UP	-0,116	0,001	0,00842	1
GSE39820_IL1B_IL6_VS_IL1B_IL6_IL23A_TI	-0,116	0,001	0,0004	1
GSE17721_POLYIC_VS_CPG_16H_BMDM_UP	-0,116	0,001	0,00068	1
MODULE_125	-0,116	0,001	0,00532	1
E2F3_UP.V1_UP	-0,116	0,001	0,00025	1
chr12q23	-0,116	0,001	0,00037	1
AMUNDSON_GAMMA_RADIATION_RESISTANCI	-0,116	0,002	0,00104	1
REACTOME_SIGNALLING_TO_ERKS	-0,116	0,002	0,00442	1

NADERI_BREAST_CANCER_PROGNOSIS_UP	-0,116	0,002	0,04028	1
REGULATION_OF_HYDROLASE_ACTIVITY	-0,116	0,002	0,00551	1
APOPTOSIS_GO	-0,116	0,002	0,00121	1
OUILLETTE_CLL_13Q14_DELETION_UP	-0,116	0,002	0,00743	1
TSAI_DNAJB4_TARGETS_DN	-0,116	0,002	0,02661	1
NAM_FXYD5_TARGETS_DN	-0,116	0,002	0,1336	1
TATTATA,MIR-374	-0,116	0,002	0,00016	1
GSE17721_POLYIC_VS_CPG_4H_BMDM_UP	-0,116	0,002	0,00044	1
REACTOME_PROCESSING_OF_CAPPED_INTRON	-0,116	0,002	0,03835	1
GSE360_CTRL_VS_B_MALAYI_HIGH_DOSE_D	-0,116	0,002	0,00032	1
VACUOLE_ORGANIZATION_AND_BIOGENESIS	0,116	0,002	0,01173	1
MODULE_158	-0,116	0,002	0,0045	1
MAHADEVAN_IMATINIB_RESISTANCE_UP	-0,116	0,002	0,00033	1
PARK_HSC_VS_MULTIPOTENT_PROGENITORS_	-0,116	0,002	0,05716	1
OXFORD_RALA_TARGETS_DN	-0,116	0,002	0,00173	1
MORF_THPO	-0,116	0,002	0,0004	1
KEGG_GLYCOSAMINOGLYCAN_BIOSYNTHESIS_	-0,116	0,002	0,00426	1
PEPTIDYL_AMINO_ACID_MODIFICATION	-0,116	0,002	0,01713	1
REACTOME_DEADENYLATION_OF_MRNA	-0,115	0,002	0,01072	1
TURJANSKI_MAPK7_TARGETS	-0,115	0,002	0,00123	1
GNF2_MBD4	-0,115	0,002	0,00023	1
REACTOME_GABA_B_RECEPTOR_ACTIVATION	-0,115	0,002	0,00014	1
MODULE_121	-0,115	0,002	0,00052	1
KINESIN_COMPLEX	-0,115	0,002	0,00923	1
ESC_J1_UP_EARLY.V1_DN	-0,115	0,002	0,00024	1
CELL_FATE_COMMITMENT	-0,115	0,002	0,00097	1
GSE360_L_DONOVANI_VS_B_MALAYI_LOW_	-0,115	0,002	0,00473	1
INAMURA_LUNG_CANCER_SCC_SUBTYPES_UP	-0,115	0,002	0,01247	1
MODULE_242	-0,115	0,002	0,00042	1
KUWANO_RNA_STABILIZED_BY_NO	-0,115	0,002	0,0021	1
FORTSCHEGGER_PHF8_TARGETS_DN	-0,115	0,002	0,00053	1
GSE27786_LSK_VS_CD4_TCELL_UP	-0,115	0,002	0,00082	1
TGTTTAC,MIR-30A-5P,MIR-30C,MIR-30D,MIR-3	-0,115	0,002	0,00048	1
GSE17580_TREG_VS_TEFF_UP	-0,115	0,002	0,00019	1
GRAHAM_CML_QUIESCENT_VS_CML_DIVIDING	-0,115	0,002	0,0218	1
GSE17721_LPS_VS_PAM3CSK4_4H_BMDM_D	-0,115	0,002	0,00186	1
SHIN_B_CELL_LYMPHOMA_CLUSTER_9	-0,115	0,002	0,02306	1
PKCA_DN.V1_DN	-0,115	0,002	0,00022	1
REACTOME_REGULATION_OF_GLUCOKINASE_	-0,115	0,002	0,02134	1
DNA_REPAIR	-0,115	0,002	0,00026	1
GCM_SMARCC1	-0,115	0,002	0,0008	1
GSE17721_LPS_VS_GARDIQUIMOD_24H_BMD	-0,115	0,002	0,02256	1

MODULE_207	-0,115	0,002	0,0015	1
CASPASE_ACTIVATION	-0,115	0,002	0,0068	1
REACTOME_G2_M_CHECKPOINTS	-0,115	0,002	0,00995	1
MODULE_534	-0,115	0,002	0,04786	1
HAHTOLA_MYCOSIS_FUNGOIDES_SKIN_DN	-0,115	0,002	0,08586	1
BARRIER_CANCER_RELAPSE_TUMOR_SAMPLE_	-0,115	0,002	0,00134	1
VALK_AML_CLUSTER_2	-0,115	0,002	0,00278	1
CYCLIN_D1_UP.V1_UP	-0,115	0,002	0,00055	1
GCM_TPT1	-0,115	0,002	0,00088	1
CHROMATIN_ASSEMBLY	-0,115	0,002	0,00241	1
KYNG_NORMAL_AGING_UP	-0,115	0,002	0,00637	1
MORF_ZNF10	-0,115	0,002	0,00109	1
GSE9650_EXHAUSTED_VS_MEMORY_CD8_TC	-0,115	0,002	0,00276	1
FOSTER_KDM1A_TARGETS_UP	-0,115	0,002	0,00124	1
ZHANG_TLX_TARGETS_36HR_DN	-0,115	0,002	0,0162	1
GRAHAM_CML_QUIESCENT_VS_NORMAL_QUI	-0,115	0,002	0,00095	1
MODULE_259	-0,115	0,002	0,00038	1
GSE39820_CTRL_VS_TGFBETA3_IL6_IL23A_	-0,115	0,002	0,00558	1
MODULE_354	-0,115	0,002	0,00028	1
TRANSLATION	-0,115	0,002	0,00246	1
AMIT_SERUM_RESPONSE_240_MCF10A	-0,115	0,002	0,03606	1
REACTOME_APOPTOSIS_INDUCED_DNA_FRAG	-0,115	0,002	0,03106	1
PID_FAK_PATHWAY	-0,115	0,002	0,00256	1
MODULE_90	-0,115	0,002	0,05155	1
CHIBA_RESPONSE_TO_TSA_DN	-0,115	0,002	0,04315	1
CHARAFE_BREAST_CANCER_BASAL_VS_MESI	-0,115	0,002	0,03467	1
TTGCCAA,MIR-182	-0,115	0,002	0,00036	1
RICKMAN_METASTASIS_UP	-0,115	0,002	0,00023	1
ELLWOOD_MYC_TARGETS_UP	-0,115	0,002	0,05016	1
MORF_NPM1	-0,115	0,002	0,00392	1
GSE22886_UNSTIM_VS_STIM_MEMORY_TCEL	-0,115	0,002	0,10704	1
GSE22886_NAIVE_CD4_TCELL_VS_48H_ACT	-0,115	0,002	0,05847	1
REACTOME_PACKAGING_OF_TELOMERE_ENDS	-0,115	0,002	0,03811	1
SECOND_MESSENGER_MEDIATED_SIGNALING	-0,115	0,002	0,00014	1
GREENBAUM_E2A_TARGETS_UP	-0,114	0,002	0,01747	1
REACTOME_ACTIVATION_OF_GENES_BY_ATF	-0,114	0,002	0,03378	1
NATSUME_RESPONSE_TO_INTERFERON_BETA	-0,114	0,002	0,00202	1
REACTOME_MITOTIC_M_M_G1_PHASES	-0,114	0,002	0,0238	1
WNT_SIGNALING	-0,114	0,002	0,00027	1
REACTOME_CELL_EXTRACELLULAR_MATRIX_I	-0,114	0,002	0,0032	1
REACTOME_SEMA3A_PAK_DEPENDENT_AXON	-0,114	0,002	0,03585	1
MODULE_571	-0,114	0,002	0,0009	1

BIOCARTA_CBL_PATHWAY	-0,114	0,002	0,00747	1
GCM_PRKCG	-0,114	0,002	0,00099	1
GSE1448_ANTI_VALPHA2_VS_VBETA5_DP_T	-0,114	0,002	0,00077	1
ATACCTC,MIR-202	-0,114	0,002	0,00033	1
MORF_PTEN	-0,114	0,002	0,00411	1
REACTOME_REGULATION_OF_KIT_SIGNALING	-0,114	0,002	0,00586	1
BROWNE_HCMV_INFECTION_4HR_UP	-0,114	0,002	0,00036	1
LIU_CMYB_TARGETS_DN	-0,114	0,002	0,01948	1
KOKKINAKIS_METHIONINE_DEPRIVATION_48H	-0,114	0,002	0,00319	1
BIOCARTA_STATHMIN_PATHWAY	-0,114	0,002	0,00122	1
SMID_BREAST_CANCER_NORMAL_LIKE_UP	-0,114	0,002	0,00014	1
TIEN_INTESTINE_PROBIOTICS_2HR_DN	-0,114	0,002	0,0167	1
GSE17721_CTRL_VS_CPG_12H_BMDM_DN	-0,114	0,002	0,01824	1
ZUCCHI_METASTASIS_DN	-0,114	0,002	0,00232	1
chr11q23	-0,114	0,002	0,00121	1
TGCACTG,MIR-148A,MIR-152,MIR-148B	-0,114	0,002	0,00019	1
LINDGREN_BLADDER_CANCER_CLUSTER_3_UI	-0,114	0,002	0,03457	1
chr7q31	-0,114	0,002	0,00054	1
V$NFKB_Q6_01	-0,114	0,002	0,00043	1
DNA_DIRECTED_DNA_POLYMERASE_ACTIVITY	-0,114	0,002	0,00086	1
NIKOLSKY_BREAST_CANCER_16Q24_AMPLIC(	-0,114	0,002	0,02601	1
GSE9650_NAIVE_VS_EXHAUSTED_CD8_TCEL	-0,114	0,002	0,00124	1
KEGG_VIBRIO_CHOLERAE_INFECTION	-0,114	0,002	0,14488	1
BORCZUK_MALIGNANT_MESOTHELIOMA_UP	-0,114	0,002	0,08752	1
GSE27786_CD8_TCELL_VS_MONO_MAC_UP	-0,114	0,002	0,00279	1
MIYAGAWA_TARGETS_OF_EWSR1_ETS_FUSIC	-0,114	0,002	0,00152	1
HYDROLASE_ACTIVITY_ACTING_ON_ESTER_B	-0,114	0,002	0,0005	1
GSE26495_PD1HIGH_VS_PD1LOW_CD8_TCEI	-0,114	0,002	0,00016	1
SCHEIDEREIT_IKK_INTERACTING_PROTEINS	-0,114	0,002	0,00156	1
REACTOME_METABOLISM_OF_NON_CODING_I	-0,114	0,002	0,02519	1
REGULATION_OF_CYTOKINE_PRODUCTION	-0,114	0,002	0,00192	1
LEE_LIVER_CANCER_HEPATOBLAST	-0,114	0,002	0,00359	1
V$STAT3_01	-0,114	0,002	0,00042	1
SHETH_LIVER_CANCER_VS_TXNIP_LOSS_PAM	-0,114	0,002	0,00029	1
PID_S1P_META_PATHWAY	-0,114	0,002	0,00046	1
NUCLEAR_PORE	-0,114	0,002	0,10571	1
CLAUS_PGR_POSITIVE_MENINGIOMA_DN	-0,114	0,002	0,00031	1
MORF_UBE2N	-0,114	0,002	0,08276	1
REACTOME_PROCESSING_OF_INTRONLESS_PR	-0,114	0,002	0,08842	1
chr3q12	-0,114	0,002	0,02481	1
REACTOME_REGULATION_OF_INSULIN_SECRET	-0,114	0,002	0,0208	1
GSE1460_CORD_VS_ADULT_BLOOD_NAIVE_(	-0,114	0,002	0,00299	1

GSE3982_DC_VS_NEUTROPHIL_DN	-0,114	0,002	0,0006	1
GSE17580_TREG_VS_TEFF_S_MANSONI_INF_	-0,114	0,002	0,00087	1
WELCSH_BRCA1_TARGETS_UP	-0,114	0,002	0,0095	1
COULOUARN_TEMPORAL_TGFB1_SIGNATURE_	-0,113	0,002	0,00609	1
BIOCARTA_RB_PATHWAY	-0,113	0,002	0,00605	1
MICROBODY	0,113	0,002	0,02218	1
PEROXISOME	0,113	0,002	0,02218	1
JAZAERI_BREAST_CANCER_BRCA1_VS_BRCA	-0,113	0,002	0,00065	1
SCHMAHL_PDGF_SIGNALING	-0,113	0,002	0,00034	1
CAIRO_HEPATOBLASTOMA_POOR_SURVIVAL	-0,113	0,002	0,00774	1
OSWALD_HEMATOPOIETIC_STEM_CELL_IN_C(	-0,113	0,002	0,0005	1
VESICLE_MEMBRANE	-0,113	0,002	0,0143	1
MODULE_122	-0,113	0,002	0,00052	1
MODULE_379	-0,113	0,002	0,00053	1
NUCLEAR_MEMBRANE	-0,113	0,002	0,05873	1
FARMER_BREAST_CANCER_CLUSTER_2	-0,113	0,002	0,01675	1
PUIFFE_INVASION_INHIBITED_BY_ASCITES_UP	-0,113	0,002	0,07626	1
GTCAGGA,MIR-378	-0,113	0,002	0,00131	1
SWEET_LUNG_CANCER_KRAS_UP	-0,113	0,002	0,02057	1
MORF_HDAC2	-0,113	0,002	0,13544	1
MORF_BCL2L11	-0,113	0,002	0,00099	1
TRANSLATION_FACTOR_ACTIVITY_NUCLEIC_/	-0,113	0,002	0,08128	1
TIEN_INTESTINE_PROBIOTICS_6HR_DN	-0,113	0,002	0,00738	1
chr13q33	-0,113	0,002	0,00271	1
SHAFFER_IRF4_MULTIPLE_MYELOMA_PROGRA	-0,113	0,002	0,02411	1
JOHNSTONE_PARVB_TARGETS_3_DN	-0,113	0,002	0,04472	1
REACTOME_KERATAN_SULFATE_BIOSYNTHES	-0,113	0,002	0,00323	1
PETROVA_PROX1_TARGETS_DN	-0,113	0,002	0,0041	1
FEEDING_BEHAVIOR	-0,113	0,002	0,00406	1
GHANDHI_BYSTANDER_IRRADIATION_UP	-0,113	0,002	0,02012	1
GSE15930_STIM_VS_STIM_AND_TRICHOSTAT	-0,113	0,002	0,00323	1
SABATES_COLORECTAL_ADENOMA_SIZE_DN	0,113	0,002	0,05846	1
WANG_ESOPHAGUS_CANCER_VS_NORMAL_U	-0,113	0,002	0,0025	1
GSE17721_CTRL_VS_POLYIC_24H_BMDM_DI	-0,113	0,002	0,00515	1
GSE17721_CTRL_VS_CPG_12H_BMDM_UP	-0,113	0,002	0,00031	1
CALVET_IRINOTECAN_SENSITIVE_VS_RESIST/	-0,113	0,002	0,00396	1
V$CEBP_Q3	-0,113	0,002	0,00017	1
LEE_LIVER_CANCER_ACOX1_UP	-0,113	0,002	0,00583	1
NEGATIVE_REGULATION_OF_CELLULAR_COMI	-0,113	0,002	0,01774	1
TONKS_TARGETS_OF_RUNX1_RUNX1T1_FUSI	-0,113	0,002	0,00014	1
CHIANG_LIVER_CANCER_SUBCLASS_CTNNB1_	-0,113	0,002	0,00035	1
ENDOTHELIAL_CELL_MIGRATION	-0,113	0,002	0,07949	1

ABBUD_LIF_SIGNALING_2_DN	-0,113	0,002	0,00013	1
GSE17974_1.5H_VS_72H_IL4_AND_ANTI_IL	-0,113	0,002	0,01411	1
DIGESTION	-0,113	0,002	0,00563	1
GSE17721_CTRL_VS_POLYIC_6H_BMDM_DN	-0,113	0,002	0,00055	1
chrxq11	-0,113	0,002	0,00353	1
ZHENG_FOXP3_TARGETS_DN	-0,113	0,002	0,00373	1
REACTOME_CELL_CYCLE_CHECKPOINTS	-0,113	0,002	0,03517	1
DAZARD_RESPONSE_TO_UV_NHEK_DN	-0,113	0,002	0,00515	1
CHROMOSOMEPERICENTRIC_REGION	-0,113	0,002	0,01414	1
SPINDLE_POLE	-0,113	0,002	0,02714	1
ADP_BINDING	-0,113	0,002	0,01009	1
V$CEBPA_01	-0,113	0,002	0,00478	1
V$NFKB_Q6	-0,113	0,002	0,00017	1
GNF2_HAT1	-0,113	0,002	0,04295	1
GSE13738_TCR_VS_BYSTANDER_ACTIVATED	-0,113	0,002	0,00039	1
REACTOME_SEMAPHORIN_INTERACTIONS	-0,113	0,002	0,00087	1
ACTIN_CYTOSKELETON	-0,113	0,002	0,00046	1
INTRACELLULAR_SIGNALING_CASCADE	-0,113	0,002	0,00048	1
PROTEIN_DIMERIZATION_ACTIVITY	-0,113	0,002	0,00014	1
MATZUK_MATERNAL_EFFECT	-0,113	0,002	0,00527	1
ATP_DEPENDENT_DNA_HELICASE_ACTIVITY	-0,112	0,002	0,00697	1
MALE_GONAD_DEVELOPMENT	-0,112	0,002	0,01968	1
DAMAGED_DNA_BINDING	-0,112	0,002	0,00171	1
GSE17721_PAM3CSK4_VS_GADIQUIMOD_6H_	-0,112	0,002	0,00807	1
EXONUCLEASE_ACTIVITY	-0,112	0,002	0,01227	1
WEIGEL_OXIDATIVE_STRESS_BY_TBH_AND_H	-0,112	0,002	0,00638	1
REACTOME_INTEGRIN_CELL_SURFACE_INTERA	-0,112	0,002	0,0021	1
OHM_EMBRYONIC_CARCINOMA_DN	-0,112	0,002	0,00027	1
GSE9650_EFFECTOR_VS_MEMORY_CD8_TCEL	-0,112	0,002	0,02973	1
GSE27786_LSK_VS_MONO_MAC_DN	-0,112	0,002	0,00146	1
PHONG_TNF_TARGETS_UP	-0,112	0,002	0,00105	1
INTRA_GOLGI_VESICLE_MEDIATED_TRANSPOR	0,112	0,002	0,02471	1
GSE14350_IL2RB_KO_VS_WT_TREG_UP	-0,112	0,002	0,00073	1
GSE27786_CD4_TCELL_VS_NEUTROPHIL_UP	-0,112	0,002	0,00323	1
MENSSEN_MYC_TARGETS	-0,112	0,002	0,17343	1
V$AML_Q6	-0,112	0,002	0,00023	1
GSE20715_0H_VS_6H_OZONE_TLR4_KO_LU	-0,112	0,002	0,00023	1
PID_ERBB1_DOWNSTREAM_PATHWAY	-0,112	0,002	0,00275	1
NUCLEAR_ENVELOPE	-0,112	0,002	0,03094	1
GSE360_L_MAJOR_VS_M_TUBERCULOSIS_MA	-0,112	0,002	0,00061	1
KINASE_REGULATOR_ACTIVITY	-0,112	0,002	0,00088	1
V$ETS1_B	-0,112	0,002	0,00018	1

BANDRES_RESPONSE_TO_CARMUSTIN_WITHC	-0,112	0,002	0,00047	1
WELCSH_BRCA1_TARGETS_DN	-0,112	0,002	0,04918	1
GUTIERREZ_CHRONIC_LYMPHOCYTIC_LEUKEMI	-0,112	0,002	0,004	1
chr1p21	-0,112	0,002	0,00128	1
GLUTAMINE_FAMILY_AMINO_ACID_METABOLI	0,112	0,002	0,00597	1
ANION_TRANSPORT	-0,112	0,002	0,00106	1
WANG_RESPONSE_TO_BEXAROTENE_UP	-0,112	0,002	0,03511	1
PID_AVB3_INTEGRIN_PATHWAY	-0,112	0,002	0,00053	1
N_ACYLTRANSFERASE_ACTIVITY	0,112	0,002	0,02983	1
chr13q12	-0,112	0,002	0,00083	1
MODULE_210	-0,112	0,002	0,02554	1
GSE15930_NAIVE_VS_72H_IN_VITRO_STIM_	-0,112	0,002	0,04202	1
GSE11057_EFF_MEM_VS_CENT_MEM_CD4_T	-0,112	0,002	0,00178	1
GSE17721_POLYIC_VS_CPG_2H_BMDM_UP	-0,112	0,002	0,00143	1
REACTOME_SIGNALING_BY_THE_B_CELL_REC	-0,112	0,002	0,02554	1
GSE360_L_DONOVANI_VS_B_MALAYI_LOW_	-0,112	0,002	0,00284	1
REACTOME_FANCONI_ANEMIA_PATHWAY	-0,112	0,002	0,01112	1
GCNP_SHH_UP_LATE.V1_DN	-0,112	0,002	0,00071	1
AMYLOID_PRECURSOR_PROTEIN_METABOLIC_	0,112	0,002	0,02852	1
PUJANA_BREAST_CANCER_LIT_INT_NETWORI	-0,112	0,002	0,01101	1
CASTELLANO_HRAS_AND_NRAS_TARGETS_U	-0,112	0,002	0,00612	1
SPIELMAN_LYMPHOBLAST_EUROPEAN_VS_AS	-0,112	0,002	0,01029	1
RASHI_RESPONSE_TO_IONIZING_RADIATION_1	-0,112	0,002	0,00019	1
NAGY_PCAF_COMPONENTS_HUMAN	-0,112	0,002	0,00161	1
MORF_IL4	-0,112	0,002	0,00052	1
POSITIVE_REGULATION_OF_CASPASE_ACTIVIT	-0,112	0,002	0,00555	1
HEART_DEVELOPMENT	-0,112	0,002	0,00017	1
GSE17974_0H_VS_48H_IN_VITRO_ACT_CD4	-0,112	0,002	0,03704	1
GSE17721_0.5H_VS_4H_POLYIC_BMDM_UP	-0,112	0,002	0,00062	1
DAZARD_UV_RESPONSE_CLUSTER_G24	-0,112	0,002	0,0188	1
GSE18791_CTRL_VS_NEWCASTLE_VIRUS_DC	-0,112	0,002	0,00909	1
REACTOME_POST_NMDA_RECEPTOR_ACTIVAT	-0,112	0,002	0,0006	1
HOFFMANN_PRE_BI_TO_LARGE_PRE_BII_LYMI	-0,112	0,002	0,00165	1
GSE6269_HEALTHY_VS_STREP_AUREUS_INF_	-0,112	0,002	0,0002	1
BIOCARTA_CXCR4_PATHWAY	-0,112	0,002	0,014	1
chr15q11	-0,112	0,002	0,01543	1
TRANSLATION_INITIATION_FACTOR_ACTIVITY	-0,112	0,002	0,11595	1
MORF_UBE2I	-0,112	0,002	0,01667	1
LI_AMPLIFIED_IN_LUNG_CANCER	-0,112	0,002	0,04536	1
MARTINEZ_TP53_TARGETS_UP	-0,112	0,002	0,00031	1
GTATTAT,MIR-369-3P	-0,112	0,002	0,00107	1
COLLER_MYC_TARGETS_UP	-0,112	0,002	0,12388	1

GSE27786_CD8_TCELL_VS_NEUTROPHIL_DN	-0,112	0,002	0,00204	1
COLLAGEN	-0,112	0,002	0,00122	1
RODRIGUES_THYROID_CARCINOMA_DN	-0,112	0,002	0,00144	1
REACTOME_AMINE_LIGAND_BINDING_RECEPT(	-0,112	0,002	0,00074	1
GTAAGAT,MIR-200A	-0,112	0,002	0,00168	1
MORF_ESR1	-0,112	0,002	0,00038	1
GSE27786_NKCELL_VS_MONO_MAC_UP	-0,111	0,002	0,00086	1
CELLULAR_COMPONENT_DISASSEMBLY	-0,111	0,002	0,00612	1
chr18q22	-0,111	0,002	0,0003	1
MODULE_337	-0,111	0,002	0,05946	1
CTACTGT,MIR-199A	-0,111	0,002	0,00048	1
BIOCARTA_MAPK_PATHWAY	-0,111	0,002	0,0004	1
ZHAN_MULTIPLE_MYELOMA_PR_UP	-0,111	0,002	0,01601	1
GSE18791_CTRL_VS_NEWCASTLE_VIRUS_DC	-0,111	0,002	0,00039	1
BASE_EXCISION_REPAIR	-0,111	0,002	0,00027	1
MORF_DDB1	-0,111	0,002	0,06621	1
GSE11864_CSF1_VS_CSF1_IFNG_PAM3CYS_	-0,111	0,002	0,04026	1
MORF_PCNA	-0,111	0,002	0,0604	1
MAINTENANCE_OF_PROTEIN_LOCALIZATION	-0,111	0,002	0,03616	1
KYNG_DNA_DAMAGE_BY_GAMMA_RADIATION	-0,111	0,002	0,00157	1
MORF_DEK	-0,111	0,002	0,09119	1
CAGTATT,MIR-200B,MIR-200C,MIR-429	-0,111	0,002	0,00039	1
GSE8515_IL1_VS_IL6_4H_STIM_)MAC_UP	-0,111	0,002	0,01415	1
REACTOME_HIV_INFECTION	-0,111	0,002	0,07117	1
ACTWSNACTNY_UNKNOWN	-0,111	0,002	0,00039	1
SASAI_TARGETS_OF_CXCR6_AND_PTCH1_DN	-0,111	0,002	0,00261	1
MODULE_184	0,111	0,002	0,09649	1
CARD_MIR302A_TARGETS	-0,111	0,002	0,00256	1
MORF_MAP2K7	-0,111	0,002	0,00073	1
MARIADASON_RESPONSE_TO_BUTYRATE_CUF	-0,111	0,002	0,00975	1
ATCATGA,MIR-433	-0,111	0,002	0,00043	1
MODULE_199	-0,111	0,002	0,00015	1
KYNG_DNA_DAMAGE_BY_4NQO_OR_GAMMA_	-0,111	0,002	0,00107	1
N_ACETYLTRANSFERASE_ACTIVITY	0,111	0,002	0,03177	1
HASLINGER_B_CLL_WITH_13Q14_DELETION	-0,111	0,002	0,00151	1
GSE17721_LPS_VS_GARDIQUIMOD_0.5H_BMI	-0,111	0,002	0,00048	1
BIOCARTA_P27_PATHWAY	-0,111	0,002	0,01211	1
TANAKA_METHYLATED_IN_ESOPHAGEAL_CAI	-0,111	0,002	0,00177	1
GSE11864_UNTREATED_VS_CSF1_IN_MAC_U	-0,111	0,002	0,00026	1
GSE13485_CTRL_VS_DAY21_YF17D_VACCIN	-0,111	0,002	0,0097	1
GRAHAM_NORMAL_QUIESCENT_VS_NORMAL_	-0,111	0,002	0,00306	1
CHROMOSOME_SEGREGATION	-0,111	0,002	0,01161	1

DEBIASI_APOPTOSIS_BY_REOVIRUS_INFECTIOI	-0,111	0,002	0,00412	1
GSE29618_BCELL_VS_MDC_DAY7_FLU_VAC	-0,111	0,002	0,00164	1
SCHEIDEREIT_IKK_TARGETS	0,111	0,002	0,45531	1
GSE27786_ERYTHROBLAST_VS_MONO_MAC_	-0,111	0,002	0,00042	1
EXTRACELLULAR_REGION	-0,111	0,002	0,00048	1
GSE17721_CTRL_VS_GARDIQUIMOD_2H_BMD	-0,111	0,002	0,0003	1
FERRANDO_T_ALL_WITH_MLL_ENL_FUSION_I	-0,111	0,002	0,00208	1
MULLIGHAN_NPM1_MUTATED_SIGNATURE_1_	-0,111	0,002	0,00067	1
GSE17721_POLYIC_VS_PAM3CSK4_0.5H_BM	-0,111	0,002	0,00481	1
CHROMATIN_REMODELING_COMPLEX	-0,111	0,002	0,00245	1
MODULE_341	-0,111	0,002	0,02941	1
LEE_AGING_NEOCORTEX_DN	-0,111	0,002	0,00044	1
MIKKELSEN_MEF_HCP_WITH_H3K27ME3	-0,111	0,003	0,00032	1
GSE15659_NAIVE_VS_PTPRC_NEG_CD4_TCE	-0,111	0,003	0,00033	1
ALK_DN.V1_UP	-0,111	0,003	0,00602	1
GSE17721_0.5H_VS_12H_GARDIQUIMOD_BM	-0,111	0,003	0,01633	1
GNF2_RAN	-0,111	0,003	0,11871	1
RIBONUCLEOPROTEIN_COMPLEX_BIOGENESIS_	-0,111	0,003	0,02923	1
REACTOME_E2F_ENABLED_INHIBITION_OF_PR	-0,111	0,003	0,01393	1
GROSS_HYPOXIA_VIA_ELK3_DN	-0,11	0,003	0,00556	1
FERNANDEZ_BOUND_BY_MYC	-0,11	0,003	0,01537	1
ENZYME_REGULATOR_ACTIVITY	-0,11	0,003	0,00014	1
GAUTSCHI_SRC_SIGNALING	-0,11	0,003	0,00031	1
chr18p11	-0,11	0,003	0,00178	1
WALLACE_JAK2_TARGETS_UP	-0,11	0,003	0,02136	1
ST_JAK_STAT_PATHWAY	-0,11	0,003	0,00215	1
XU_HGF_TARGETS_REPRESSED_BY_AKT1_DN	-0,11	0,003	0,00054	1
GSE28237_FOLLICULAR_VS_LATE_GC_BCELI	-0,11	0,003	0,00028	1
SAKAI_TUMOR_INFILTRATING_MONOCYTES_U	-0,11	0,003	0,00556	1
KRAS.LUNG_UP.V1_UP	-0,11	0,003	0,00223	1
GSE3982_DC_VS_NEUTROPHIL_LPS_STIM_UF	-0,11	0,003	0,00786	1
GSE360_CTRL_VS_M_TUBERCULOSIS_MAC_D	-0,11	0,003	0,00341	1
RODWELL_AGING_KIDNEY_NO_BLOOD_UP	-0,11	0,003	0,00034	1
chr19q12	-0,11	0,003	0,00746	1
MORF_FBL	-0,11	0,003	0,04716	1
DYSTROPHIN_ASSOCIATED_GLYCOPROTEIN_C	-0,11	0,003	0,00013	1
SIRNA_EIF4GI_DN	-0,11	0,003	0,08223	1
SASSON_RESPONSE_TO_FORSKOLIN_UP	-0,11	0,003	0,01738	1
ZWANG_CLASS_3_TRANSIENTLY_INDUCED_B	-0,11	0,003	0,00132	1
PODAR_RESPONSE_TO_ADAPHOSTIN_DN	-0,11	0,003	0,26102	1
V$GATA4_Q3	-0,11	0,003	0,00042	1
V$NFE2_01	-0,11	0,003	0,00076	1

REACTOME_FGFR4_LIGAND_BINDING_AND_A(	-0,11	0,003	0,00025	1
TBK1.DF_UP	-0,11	0,003	0,00847	1
GSE39820_TGFBETA3_IL6_VS_TGFBETA3_IL	-0,11	0,003	0,00158	1
ABDELMOHSEN_ELAVL4_TARGETS	-0,11	0,003	0,00434	1
REACTOME_PHOSPHORYLATION_OF_THE_APC	-0,11	0,003	0,00778	1
GCM_PFN1	-0,11	0,003	0,01617	1
GSE17721_POLYIC_VS_GARDIQUIMOD_12H_I	-0,11	0,003	0,00118	1
GSE17721_CTRL_VS_LPS_8H_BMDM_DN	-0,11	0,003	0,00021	1
KEGG_OLFACTORY_TRANSDUCTION	-0,11	0,003	0,00588	1
BIOCARTA_BIOPEPTIDES_PATHWAY	-0,11	0,003	0,00029	1
GSE29618_PDC_VS_MDC_DAY7_FLU_VACCII	-0,11	0,003	0,00084	1
CHIARETTI_T_ALL_REFRACTORY_TO_THERAP	-0,11	0,003	0,00571	1
DAZARD_RESPONSE_TO_UV_NHEK_UP	-0,11	0,003	0,0069	1
GSE360_T_GONDII_VS_B_MALAYI_LOW_DOS	-0,11	0,003	0,00496	1
GSE17721_LPS_VS_CPG_16H_BMDM_DN	-0,11	0,003	0,00081	1
LU_EZH2_TARGETS_DN	-0,11	0,003	0,00691	1
MODULE_14	-0,11	0,003	0,00106	1
CAHOY_ASTROGLIAL	-0,11	0,003	0,0004	1
GSE29618_PDC_VS_MDC_UP	-0,11	0,003	0,00064	1
GSE39820_CTRL_VS_TGFBETA1_IL6_CD4_T	-0,11	0,003	0,00865	1
LIU_VMYB_TARGETS_UP	-0,11	0,003	0,00643	1
GSE17721_LPS_VS_PAM3CSK4_24H_BMDM_	-0,11	0,003	0,00855	1
TAGGTCA,MIR-192,MIR-215	-0,11	0,003	0,00023	1
ZHAN_LATE_DIFFERENTIATION_GENES_DN	-0,11	0,003	0,00089	1
REACTOME_NONSENSE_MEDIATED_DECAY_EI	-0,11	0,003	0,00137	1
GSE9650_GP33_VS_GP276_LCMV_SPECIFIC_	-0,11	0,003	0,0596	1
GSE2706_2H_VS_8H_R848_AND_LPS_STIM_	-0,11	0,003	0,0289	1
MORF_PPP5C	-0,11	0,003	0,00374	1
GNF2_SMC1L1	-0,11	0,003	0,04569	1
MORF_ATF2	-0,11	0,003	0,00025	1
TORCHIA_TARGETS_OF_EWSR1_FLI1_FUSION	-0,11	0,003	0,00071	1
AAAGGGA,MIR-204,MIR-211	-0,11	0,003	0,00096	1
LEE_AGING_NEOCORTEX_UP	-0,109	0,003	0,0028	1
GSE24142_EARLY_THYMIC_PROGENITOR_VS_	-0,109	0,003	0,00155	1
JAZAG_TGFB1_SIGNALING_VIA_SMAD4_DN	-0,109	0,003	0,00168	1
HINATA_NFKB_TARGETS_KERATINOCYTE_UP	-0,109	0,003	0,04262	1
ROVERSI_GLIOMA_COPY_NUMBER_DN	-0,109	0,003	0,00046	1
GSE17721_0.5H_VS_24H_LPS_BMDM_DN	-0,109	0,003	0,00204	1
LIPOPROTEIN_BIOSYNTHETIC_PROCESS	0,109	0,003	0,04533	1
DELASERNA_MYOD_TARGETS_DN	-0,109	0,003	0,00308	1
PID_BARD1PATHWAY	-0,109	0,003	0,03188	1
DORN_ADENOVIRUS_INFECTION_24HR_DN	-0,109	0,003	0,00043	1

REACTOME_EARLY_PHASE_OF_HIV_LIFE_CYC	-0,109	0,003	0,01793	1
MODULE_411	-0,109	0,003	0,02944	1
GAZDA_DIAMOND_BLACKFAN_ANEMIA_PROG	-0,109	0,003	0,02293	1
REACTOME_LIGAND_GATED_ION_CHANNEL_T	-0,109	0,003	0,01728	1
RICKMAN_TUMOR_DIFFERENTIATED_MODERAT	-0,109	0,003	0,00369	1
TGCAAAC,MIR-452	-0,109	0,003	0,00058	1
ACATTCC,MIR-1,MIR-206	-0,109	0,003	0,00099	1
LIN_NPAS4_TARGETS_UP	-0,109	0,003	0,00743	1
GSE13229_IMM_VS_MATURE_NKCELL_UP	-0,109	0,003	0,00014	1
BOYAULT_LIVER_CANCER_SUBCLASS_G2	-0,109	0,003	0,0144	1
BAELDE_DIABETIC_NEPHROPATHY_DN	-0,109	0,003	0,00146	1
GSE17721_CTRL_VS_PAM3CSK4_8H_BMDM_	-0,109	0,003	0,00302	1
GSE17721_CTRL_VS_POLYIC_2H_BMDM_UP	-0,109	0,003	0,00176	1
MORF_MSH2	-0,109	0,003	0,14323	1
RNA_3END_PROCESSING	-0,109	0,003	0,10751	1
REACTOME_NITRIC_OXIDE_STIMULATES_GUAN	-0,109	0,003	0,00014	1
ZHANG_RESPONSE_TO_CANTHARIDIN_DN	-0,109	0,003	0,0078	1
NEBEN_AML_WITH_FLT3_OR_NRAS_UP	0,109	0,003	0,42563	1
MARSHALL_VIRAL_INFECTION_RESPONSE_UP	-0,109	0,003	0,09302	1
REACTOME_GPCR_LIGAND_BINDING	-0,109	0,003	0,00059	1
BIOCARTA_MCM_PATHWAY	-0,109	0,003	0,01478	1
GSE17974_IL4_AND_ANTI_IL12_VS_UNTREA	-0,109	0,003	0,00128	1
XU_CREBBP_TARGETS_DN	-0,109	0,003	0,00099	1
SULFURIC_ESTER_HYDROLASE_ACTIVITY	-0,109	0,003	0,0041	1
GSE17974_CTRL_VS_ACT_IL4_AND_ANTI_IL	-0,109	0,003	0,09364	1
GSE24142_DN2_VS_DN3_THYMOCYTE_FETA	-0,109	0,003	0,00357	1
OXFORD_RALA_AND_RALB_TARGETS_UP	-0,109	0,003	0,28007	1
GSE29618_PRE_VS_DAY7_POST_TIV_FLU_V	-0,109	0,003	0,00058	1
OXIDOREDUCTASE_ACTIVITY_ACTING_ON_SU	0,109	0,003	0,00978	1
GSE10325_LUPUS_CD4_TCELL_VS_LUPUS_M	-0,109	0,003	0,00016	1
MODULE_289	-0,109	0,003	0,00112	1
REACTOME_S_PHASE	-0,109	0,003	0,04802	1
TTTTGAG,MIR-373	-0,109	0,003	0,00314	1
FERRANDO_T_ALL_WITH_MLL_ENL_FUSION_	-0,109	0,003	0,01161	1
GSE7460_CTRL_VS_TGFB_TREATED_ACT_TC	-0,109	0,003	0,00158	1
GSE17721_12H_VS_24H_POLYIC_BMDM_DN	-0,109	0,003	0,00108	1
MORF_TPT1	-0,109	0,003	0,00173	1
RICKMAN_TUMOR_DIFFERENTIATED_WELL_VS	-0,109	0,003	0,06373	1
AACTGAC,MIR-223	-0,109	0,003	0,00082	1
chr12p12	-0,109	0,003	0,00443	1
GNF2_MSH6	-0,109	0,003	0,06243	1
AMINE_RECEPTOR_ACTIVITY	-0,109	0,003	0,00022	1

GSE14308_TH17_VS_NATURAL_TREG_UP	-0,109	0,003	0,00929	1
CYCLIC_NUCLEOTIDE_METABOLIC_PROCESS	-0,109	0,003	0,00277	1
GSE7852_THYMUS_VS_FAT_TCONV_DN	-0,109	0,003	0,00219	1
LI_INDUCED_T_TO_NATURAL_KILLER_DN	-0,108	0,003	0,00039	1
DER_IFN_GAMMA_RESPONSE_DN	-0,108	0,003	0,05277	1
chr4p12	-0,108	0,003	0,02924	1
NEGATIVE_REGULATION_OF_DNA_METABOLIC	-0,108	0,003	0,04136	1
GSE17721_LPS_VS_PAM3CSK4_8H_BMDM_D	-0,108	0,003	0,0122	1
REACTOME_NRAGE_SIGNALS_DEATH_THROUG	-0,108	0,003	0,00035	1
BROWNE_HCMV_INFECTION_30MIN_DN	-0,108	0,003	0,00155	1
MAINTENANCE_OF_CELLULAR_PROTEIN_LOCA	-0,108	0,003	0,05884	1
KALMA_E2F1_TARGETS	-0,108	0,003	0,01894	1
GSE17721_POLYIC_VS_GARDIQUIMOD_16H_E	-0,108	0,003	0,03726	1
GSE13306_RA_VS_UNTREATED_MEM_CD4_T	-0,108	0,003	0,02152	1
HOLLEMAN_DAUNORUBICIN_ALL_UP	-0,108	0,003	0,05091	1
GNF2_CDH11	-0,108	0,003	0,00281	1
ROZANOV_MMP14_TARGETS_SUBSET	-0,108	0,003	0,0084	1
KEGG_NOTCH_SIGNALING_PATHWAY	-0,108	0,003	0,00021	1
ATP_BINDING	-0,108	0,003	0,00074	1
BREDEMEYER_RAG_SIGNALING_NOT_VIA_ATM	-0,108	0,003	0,00242	1
MODULE_248	-0,108	0,003	0,00072	1
COFACTOR_TRANSPORTER_ACTIVITY	0,108	0,003	0,15723	1
CELL_PROJECTION_BIOGENESIS	-0,108	0,003	0,00089	1
MORF_FEN1	-0,108	0,003	0,04538	1
MEL18_DN.V1_UP	-0,108	0,003	0,01091	1
GSE24634_NAIVE_CD4_TCELL_VS_DAY7_IL4	-0,108	0,003	0,03447	1
GSE15930_NAIVE_VS_24H_IN_VITRO_STIM_	-0,108	0,003	0,06711	1
REACTOME_SYNTHESIS_OF_GLYCOSYLPHOSPH	0,108	0,003	0,056	1
BIOCARTA_NKCELLS_PATHWAY	-0,108	0,003	0,00647	1
TGTATGA,MIR-485-3P	-0,108	0,003	0,0005	1
GSE17721_PAM3CSK4_VS_CPG_4H_BMDM_U	-0,108	0,003	0,00339	1
chr5q32	-0,108	0,003	0,00384	1
BRCA1_DN.V1_UP	-0,108	0,003	0,00061	1
PHOSPHORIC_DIESTER_HYDROLASE_ACTIVITY	-0,108	0,003	0,00482	1
GSE24634_NAIVE_CD4_TCELL_VS_DAY3_IL4	-0,108	0,003	0,07004	1
GSE3982_MAST_CELL_VS_NKCELL_UP	-0,108	0,003	0,00731	1
REACTOME_PERK_REGULATED_GENE_EXPRESS	-0,108	0,003	0,08568	1
DARWICHE_PAPILLOMA_RISK_HIGH_DN	-0,108	0,003	0,00032	1
GSE9988_LOW_LPS_VS_VEHICLE_TREATED_	-0,108	0,003	0,01403	1
GSE32423_CTRL_VS_IL7_MEMORY_CD8_TCE	-0,108	0,003	0,00192	1
BIDUS_METASTASIS_UP	-0,108	0,003	0,06473	1
OKUMURA_INFLAMMATORY_RESPONSE_LPS	-0,108	0,003	0,00202	1

MCGOWAN_RSP6_TARGETS_UP	-0,108	0,003	0,00099	1
GAVIN_FOXP3_TARGETS_CLUSTER_P2	-0,108	0,003	0,00226	1
GSE17721_CTRL_VS_LPS_6H_BMDM_DN	-0,108	0,003	0,00324	1
DAZARD_UV_RESPONSE_CLUSTER_G3	-0,108	0,003	0,04523	1
GSE16522_ANTI_CD3CD28_STIM_VS_UNSTIM	-0,108	0,003	0,00015	1
BRUECKNER_TARGETS_OF_MIRLET7A3_UP	-0,108	0,003	0,04899	1
GSE3982_BCELL_VS_EFF_MEMORY_CD4_TCE	-0,108	0,003	0,00045	1
OZANNE_AP1_TARGETS_UP	-0,108	0,003	0,04655	1
GSE17721_PAM3CSK4_VS_CPG_24H_BMDM_	-0,108	0,003	0,00292	1
chr3q11	-0,108	0,003	0,01862	1
TRANSCRIPTION_FACTOR_TFIID_COMPLEX	-0,108	0,003	0,04994	1
KUMAR_AUTOPHAGY_NETWORK	-0,108	0,003	0,00464	1
DAVICIONI_TARGETS_OF_PAX_FOXO1_FUSION	-0,108	0,003	0,00018	1
PHOSPHATASE_INHIBITOR_ACTIVITY	-0,108	0,003	0,00275	1
NIKOLSKY_OVERCONNECTED_IN_BREAST_CAI	-0,108	0,003	0,00079	1
chr11q11	-0,108	0,003	0,00048	1
KRIEG_HYPOXIA_VIA_KDM3A	-0,108	0,003	0,05841	1
TRANSFERASE_ACTIVITY_TRANSFERRING_SUL	-0,108	0,003	0,00984	1
TONG_INTERACT_WITH_PTTG1	-0,108	0,003	0,00299	1
GTTTGTT,MIR-495	-0,108	0,003	0,00329	1
IWANAGA_CARCINOGENESIS_BY_KRAS_UP	-0,107	0,003	0,00822	1
REACTOME_TRANSLATION	-0,107	0,003	0,00469	1
GSE11864_CSF1_IFNG_VS_CSF1_IFNG_PAM3	-0,107	0,003	0,01498	1
POSITIVE_REGULATION_OF_PEPTIDYL_TYROSI	-0,107	0,003	0,0003	1
GSE2826_WT_VS_XID_BCELL_UP	-0,107	0,003	0,01143	1
HEIDENBLAD_AMPLICON_8Q24_UP	-0,107	0,003	0,0396	1
LEE_LIVER_CANCER_MYC_DN	-0,107	0,003	0,00206	1
MODULE_178	-0,107	0,003	0,00122	1
GGARNTKYCCA_UNKNOWN	-0,107	0,003	0,00056	1
ONKEN_UVEAL_MELANOMA_DN	-0,107	0,003	0,00014	1
CHESLER_BRAIN_HIGHEST_GENETIC_VARIANC	-0,107	0,003	0,00381	1
GSE17721_POLYIC_VS_GARDIQUIMOD_4H_BM	-0,107	0,003	0,00305	1
V$AML1_01	-0,107	0,003	0,00024	1
V$AML1_Q6	-0,107	0,003	0,00024	1
V$COREBINDINGFACTOR_Q6	-0,107	0,003	0,00031	1
chr11q21	-0,107	0,003	0,18196	1
GSE360_L_MAJOR_VS_B_MALAYI_LOW_DOS	-0,107	0,003	0,00047	1
BIOCARTA_CHREBP2_PATHWAY	-0,107	0,003	0,01385	1
GINESTIER_BREAST_CANCER_ZNF217_AMPLIF	-0,107	0,003	0,00105	1
GSE12366_PLASMA_CELL_VS_NAIVE_BCELL_	-0,107	0,003	0,00034	1
PID_NECTIN_PATHWAY	-0,107	0,003	0,04172	1
SENESE_HDAC1_TARGETS_UP	-0,107	0,003	0,05675	1

HEDVAT_ELF4_TARGETS_UP	-0,107	0,003	0,0033	1
ADENYL_RIBONUCLEOTIDE_BINDING	-0,107	0,003	0,00093	1
NIELSEN_LEIOMYOSARCOMA_DN	-0,107	0,003	0,00246	1
KEGG_SPLICEOSOME	-0,107	0,003	0,02568	1
REACTOME_UNWINDING_OF_DNA	-0,107	0,003	0,03856	1
REACTOME_G1_S_TRANSITION	-0,107	0,003	0,05594	1
REACTOME_CLASS_A1_RHODOPSIN_LIKE_REC	-0,107	0,003	0,0008	1
DELPUECH_FOXO3_TARGETS_UP	-0,107	0,003	0,00612	1
V$STAT3_02	-0,107	0,003	0,00015	1
KEGG_UBIQUITIN_MEDIATED_PROTEOLYSIS	-0,107	0,003	0,02187	1
PLASARI_TGFB1_SIGNALING_VIA_NFIC_1HR_I	-0,107	0,003	0,00641	1
RB_DN.V1_DN	-0,107	0,003	0,00046	1
KAAB_HEART_ATRIUM_VS_VENTRICLE_DN	-0,107	0,003	0,00049	1
GSE27786_LIN_NEG_VS_ERYTHROBLAST_UP	-0,107	0,003	0,00262	1
GSE36476_CTRL_VS_TSST_ACT_16H_MEMO	-0,107	0,003	0,09234	1
GSE1460_INTRATHYMIC_T_PROGENITOR_VS_	-0,107	0,004	0,0022	1
GSE20715_0H_VS_24H_OZONE_TLR4_KO_L	-0,107	0,004	0,00047	1
chr20p12	-0,107	0,004	0,03625	1
EUKARYOTIC_TRANSLATION_INITIATION_FACT	-0,107	0,004	0,15042	1
KAUFFMANN_MELANOMA_RELAPSE_UP	-0,107	0,004	0,02033	1
CAFFAREL_RESPONSE_TO_THC_24HR_5_DN	-0,107	0,004	0,08103	1
AMIT_EGF_RESPONSE_480_MCF10A	-0,107	0,004	0,04292	1
GCTTGAA,MIR-498	-0,107	0,004	0,00187	1
GSE9006_HEALTHY_VS_TYPE_1_DIABETES_I	-0,107	0,004	0,01823	1
GSE1460_CD4_THYMOCYTE_VS_THYMIC_STR	-0,107	0,004	0,00019	1
UDAYAKUMAR_MED1_TARGETS_UP	-0,107	0,004	0,18175	1
OSWALD_HEMATOPOIETIC_STEM_CELL_IN_CO	-0,107	0,004	0,00187	1
HUPER_BREAST_BASAL_VS_LUMINAL_DN	-0,107	0,004	0,00961	1
INTEGRATOR_COMPLEX	-0,107	0,004	0,00904	1
GSE17974_IL4_AND_ANTI_IL12_VS_UNTREA	-0,107	0,004	0,00045	1
MODULE_342	-0,107	0,004	0,00014	1
ATGAAGG,MIR-205	-0,107	0,004	0,00198	1
GSE17721_POLYIC_VS_PAM3CSK4_6H_BMDI	-0,107	0,004	0,05904	1
JAIN_NFKB_SIGNALING	-0,107	0,004	0,1229	1
BIOCARTA_IL22BP_PATHWAY	-0,107	0,004	0,00504	1
GSE17721_CTRL_VS_PAM3CSK4_2H_BMDM_	-0,107	0,004	0,00164	1
MODULE_280	-0,107	0,004	0,01425	1
GSE17721_PAM3CSK4_VS_CPG_16H_BMDM_	-0,107	0,004	0,00119	1
GSE17974_CTRL_VS_ACT_IL4_AND_ANTI_IL	-0,107	0,004	0,03503	1
PEREZ_TP63_TARGETS	-0,107	0,004	0,00039	1
REACTOME_G_PROTEIN_ACTIVATION	-0,106	0,004	0,00619	1
V$CEBP_Q2_01	-0,106	0,004	0,0005	1

GSE15930_NAIVE_VS_72H_IN_VITRO_STIM_	-0,106	0,004	0,05986	1
MOREAUX_MULTIPLE_MYELOMA_BY_TACI_DN	-0,106	0,004	0,08072	1
GSE15930_NAIVE_VS_48H_IN_VITRO_STIM_	-0,106	0,004	0,1038	1
ANTI_APOPTOSIS	-0,106	0,004	0,01516	1
ACTGTAG,MIR-139	-0,106	0,004	0,00091	1
BIOCARTA_BARRESTIN_PATHWAY	-0,106	0,004	0,00199	1
GSE7460_CTRL_VS_TGFB_TREATED_ACT_FC	-0,106	0,004	0,02794	1
MODULE_117	-0,106	0,004	0,00027	1
MODULE_414	-0,106	0,004	0,00776	1
REACTOME_MRNA_DECAY_BY_3_TO_5_EXOR	-0,106	0,004	0,04122	1
GSE9650_EFFECTOR_VS_EXHAUSTED_CD8_T	-0,106	0,004	0,00855	1
MODULE_238	-0,106	0,004	0,0012	1
POSITIVE_REGULATION_OF_PHOSPHORYLATIC	-0,106	0,004	0,00022	1
GSE11864_CSF1_VS_CSF1_IFNG_IN_MAC_DI	-0,106	0,004	0,02313	1
LIEN_BREAST_CARCINOMA_METAPLASTIC_VS	0,106	0,004	0,27574	1
GSE9006_1MONTH_VS_4MONTH_POST_TYPE	-0,106	0,004	0,00999	1
REACTOME_3_UTR_MEDIATED_TRANSLATION	-0,106	0,004	0,00263	1
GSE360_HIGH_DOSE_B_MALAYI_VS_M_TUBE	-0,106	0,004	0,00986	1
DACOSTA_ERCC3_ALLELE_XPCS_VS_TTD_DN	-0,106	0,004	0,0011	1
GAUSSMANN_MLL_AF4_FUSION_TARGETS_F_	-0,106	0,004	0,00014	1
MORF_PTPRR	-0,106	0,004	0,00386	1
GSE39820_TGFBETA1_VS_TGFBETA3_IN_IL6	-0,106	0,004	0,00316	1
MODULE_203	-0,106	0,004	0,10105	1
MODULE_254	-0,106	0,004	0,0494	1
RNA_CATABOLIC_PROCESS	-0,106	0,004	0,07697	1
GSE17721_0.5H_VS_8H_GARDIQUIMOD_BMD	-0,106	0,004	0,00976	1
chr1q22	-0,106	0,004	0,0032	1
GSE360_DC_VS_MAC_M_TUBERCULOSIS_DN	-0,106	0,004	0,01356	1
GSE15750_WT_VS_TRAF6KO_DAY10_EFF_C	-0,106	0,004	0,02056	1
GSE9988_LOW_LPS_VS_CTRL_TREATED_MO	-0,106	0,004	0,01241	1
LIU_TARGETS_OF_VMYB_VS_CMYB_DN	-0,106	0,004	0,0003	1
PHOSPHOLIPASE_C_ACTIVITY	-0,106	0,004	0,08406	1
TSUDA_ALVEOLAR_SOFT_PART_SARCOMA	-0,106	0,004	0,01363	1
HASLINGER_B_CLL_WITH_MUTATED_VH_GEN	-0,106	0,004	0,00115	1
GSE29618_PRE_VS_DAY7_FLU_VACCINE_PD	-0,106	0,004	0,02938	1
V$MAF_Q6	-0,106	0,004	0,00023	1
V$IRF7_01	-0,106	0,004	0,00138	1
MODULE_286	-0,106	0,004	0,00279	1
MATTHEWS_SKIN_CARCINOGENESIS_VIA_JUN	-0,106	0,004	0,00668	1
EHRLICH_ICF_SYNDROM_DN	-0,106	0,004	0,00151	1
PLASARI_TGFB1_TARGETS_10HR_DN	-0,106	0,004	0,00021	1
CELL_FRACTION	-0,106	0,004	0,00321	1

REACTOME_CELL_DEATH_SIGNALLING_VIA_N	-0,106	0,004	0,00023	1
BIOCARTA_PTC1_PATHWAY	-0,106	0,004	0,01817	1
KERLEY_RESPONSE_TO_CISPLATIN_DN	-0,106	0,004	0,00309	1
REACTOME_TRIGLYCERIDE_BIOSYNTHESIS	-0,106	0,004	0,00127	1
MORF_MDM2	-0,106	0,004	0,00072	1
TRANSITION_METAL_ION_TRANSPORT	0,106	0,004	0,08482	1
GSE27786_CD4_TCELL_VS_MONO_MAC_UP	-0,106	0,004	0,01657	1
MYLLYKANGAS_AMPLIFICATION_HOT_SPOT_1	-0,106	0,004	0,00233	1
MODULE_21	-0,106	0,004	0,10814	1
BANDRES_RESPONSE_TO_CARMUSTIN_MGMT_	-0,106	0,004	0,00074	1
REACTOME_HEMOSTASIS	-0,106	0,004	0,00107	1
BIOCARTA_ATM_PATHWAY	-0,106	0,004	0,0023	1
PROTEOGLYCAN_BIOSYNTHETIC_PROCESS	-0,106	0,004	0,00079	1
NIELSEN_GIST_VS_SYNOVIAL_SARCOMA_DN	-0,106	0,004	0,00153	1
V$HSF2_01	-0,106	0,004	0,00034	1
MYLLYKANGAS_AMPLIFICATION_HOT_SPOT_2	-0,106	0,004	0,00781	1
POSITIVE_REGULATION_OF_IMMUNE_RESPONS	-0,106	0,004	0,00441	1
GSE29618_PRE_VS_DAY7_POST_TIV_FLU_V	-0,106	0,004	0,0003	1
G_PROTEIN_COUPLED_RECEPTOR_PROTEIN_S	-0,106	0,004	0,00032	1
AMIT_EGF_RESPONSE_60_MCF10A	-0,106	0,004	0,00021	1
V$IRF1_Q6	-0,106	0,004	0,0013	1
KIM_RESPONSE_TO_TSA_AND_DECITABINE_U	-0,106	0,004	0,00297	1
REACTOME_ACTIVATION_OF_ATR_IN_RESPON	-0,106	0,004	0,02982	1
PROTEIN_STABILIZATION	-0,106	0,004	0,00031	1
MODULE_162	-0,105	0,004	0,08918	1
GSE17974_0H_VS_72H_IN_VITRO_ACT_CD4	-0,105	0,004	0,05555	1
ZHANG_ADIPOGENESIS_BY_BMP7	-0,105	0,004	0,00408	1
IL2_UP.V1_UP	-0,105	0,004	0,00282	1
GSE32423_IL7_VS_IL7_IL4_MEMORY_CD8_T	-0,105	0,004	0,00076	1
chr9p24	-0,105	0,004	0,00142	1
CHNG_MULTIPLE_MYELOMA_HYPERPLOID_UP	-0,105	0,004	0,0007	1
CHEMELLO_SOLEUS_VS_EDL_MYOFIBERS_UP	-0,105	0,004	0,00106	1
DAIRKEE_TERT_TARGETS_UP	-0,105	0,004	0,00848	1
BYSTRYKH_HEMATOPOIESIS_STEM_CELL_IL3F	-0,105	0,004	0,0033	1
MORF_ACTG1	-0,105	0,004	0,00489	1
GSE6566_STRONG_VS_WEAK_DC_STIMULAT	-0,105	0,004	0,00081	1
GSE15930_NAIVE_VS_24H_IN_VITRO_STIM_	-0,105	0,004	0,07677	1
GSE37416_CTRL_VS_48H_F_TULARENSIS_LV	-0,105	0,004	0,00069	1
SWEET_KRAS_TARGETS_UP	-0,105	0,004	0,00046	1
SERVITJA_ISLET_HNF1A_TARGETS_UP	-0,105	0,004	0,0005	1
ACTACCT,MIR-196A,MIR-196B	-0,105	0,004	0,00042	1
CEBALLOS_TARGETS_OF_TP53_AND_MYC_U	-0,105	0,004	0,00042	1

PHOSPHORIC_ESTER_HYDROLASE_ACTIVITY	-0,105	0,004	0,0007	1
MAHADEVAN_RESPONSE_TO_MP470_UP	-0,105	0,004	0,14214	1
GSE9988_ANTI_TREM1_AND_LPS_VS_VEHICI	-0,105	0,004	0,04834	1
SPINDLE_MICROTUBULE	-0,105	0,004	0,02632	1
EXTRACELLULAR_SPACE	-0,105	0,004	0,0027	1
KUMAR_TARGETS_OF_MLL_AF9_FUSION	-0,105	0,004	0,0022	1
WU_HBX_TARGETS_1_UP	-0,105	0,004	0,11573	1
MODULE_500	-0,105	0,004	0,02753	1
GERY_CEBP_TARGETS	-0,105	0,004	0,00455	1
JIANG_TIP30_TARGETS_DN	-0,105	0,004	0,02771	1
GSE30083_SP1_VS_SP3_THYMOCYTE_UP	-0,105	0,004	0,00232	1
GRAHAM_CML_DIVIDING_VS_NORMAL_QUIES(	-0,105	0,004	0,00263	1
chr7p15	-0,105	0,004	0,00588	1
IL15_UP.V1_UP	-0,105	0,004	0,00327	1
MARKS_HDAC_TARGETS_UP	-0,105	0,004	0,01948	1
MEISSNER_BRAIN_HCP_WITH_H3_UNMETHYL/	-0,105	0,004	0,03108	1
GSE360_CTRL_VS_B_MALAYI_LOW_DOSE_M	-0,105	0,004	0,00804	1
V$CEBP_01	-0,105	0,004	0,00022	1
BIOCARTA_CACAM_PATHWAY	-0,105	0,004	0,0008	1
MODULE_287	-0,105	0,004	0,00058	1
GSE7460_CD8_TCELL_VS_CD4_TCELL_ACT_	-0,105	0,004	0,00315	1
DARWICHE_SQUAMOUS_CELL_CARCINOMA_UI	-0,105	0,004	0,03079	1
GSE17721_PAM3CSK4_VS_GADIQUIMOD_16I	-0,105	0,004	0,00971	1
SWEET_KRAS_TARGETS_DN	-0,105	0,004	0,0273	1
BROWNE_HCMV_INFECTION_10HR_UP	-0,105	0,004	0,00462	1
NEUROPEPTIDE_HORMONE_ACTIVITY	-0,105	0,004	0,00053	1
POSITIVE_REGULATION_OF_PHOSPHATE_MET	-0,105	0,004	0,00016	1
FLECHNER_PBL_KIDNEY_TRANSPLANT_OK_V:	-0,105	0,004	0,07809	1
SEITZ_NEOPLASTIC_TRANSFORMATION_BY_8	-0,105	0,004	0,15022	1
TAKEDA_TARGETS_OF_NUP98_HOXA9_FUSIC	-0,105	0,004	0,00158	1
FIGUEROA_AML_METHYLATION_CLUSTER_1_I	-0,105	0,004	0,01097	1
chr3q24	-0,105	0,004	0,09059	1
V$GATA1_05	-0,105	0,004	0,00074	1
REGULATION_OF_IMMUNE_EFFECTOR_PROCES	-0,105	0,004	0,01025	1
MIKKELSEN_ES_LCP_WITH_H3K4ME3_AND_H	-0,105	0,004	0,00076	1
GSE34205_HEALTHY_VS_RSV_INF_INFANT_I	-0,104	0,004	0,0002	1
REACTOME_NEP_NS2_INTERACTS_WITH_THE	-0,104	0,004	0,05991	1
ITO_PTTG1_TARGETS_DN	-0,104	0,004	0,07093	1
GSE15930_NAIVE_VS_48H_IN_VITRO_STIM_	-0,104	0,004	0,15745	1
BONOME_OVARIAN_CANCER_POOR_SURVIVAI	-0,104	0,004	0,01551	1
KANG_GIST_WITH_PDGFRA_UP	-0,104	0,004	0,0008	1
GSE20151_CTRL_VS_FUSOBACT_NUCLEATUI	-0,104	0,004	0,00074	1

TAKEDA_TARGETS_OF_NUP98_HOXA9_FUSIC	-0,104	0,004	0,00155	1
GSE2197_IMMUNOSUPPRESSIVE_DNA_VS_UN	-0,104	0,004	0,00698	1
GSE10325_CD4_TCELL_VS_MYELOID_UP	-0,104	0,004	0,00042	1
REGULATION_OF_CELL_MIGRATION	-0,104	0,004	0,00214	1
TSAI_RESPONSE_TO_IONIZING_RADIATION	-0,104	0,004	0,00303	1
DORN_ADENOVIRUS_INFECTION_32HR_UP	-0,104	0,004	0,00285	1
MATHEW_FANCONI_ANEMIA_GENES	-0,104	0,004	0,03552	1
CHIANG_LIVER_CANCER_SUBCLASS_INTERFEF	-0,104	0,004	0,0013	1
GSE17721_12H_VS_24H_LPS_BMDM_UP	-0,104	0,004	0,0042	1
SCGGAAGY_V$ELK1_02	-0,104	0,004	0,0014	1
MYLLYKANGAS_AMPLIFICATION_HOT_SPOT_2	-0,104	0,004	0,0049	1
chr1p22	-0,104	0,004	0,02448	1
DNA_DAMAGE_RESPONSESIGNAL_TRANSDUCT	-0,104	0,004	0,0007	1
REACTOME_ACTIVATION_OF_THE_PRE_REPLI(	-0,104	0,004	0,04591	1
MALONEY_RESPONSE_TO_17AAG_DN	-0,104	0,004	0,04999	1
GSE17721_LPS_VS_POLYIC_24H_BMDM_UP	-0,104	0,004	0,11846	1
YANAGISAWA_LUNG_CANCER_RECURRENCE	-0,104	0,004	0,0773	1
GSE19825_NAIVE_VS_IL2RAHIGH_DAY3_EFF	-0,104	0,004	0,01051	1
V$NF1_Q6_01	-0,104	0,004	0,00013	1
GNF2_ST13	-0,104	0,004	0,00292	1
RIZKI_TUMOR_INVASIVENESS_2D_UP	-0,104	0,004	0,00153	1
NEGATIVE_REGULATION_OF_PHOSPHORYLATI	-0,104	0,004	0,01261	1
GSE16522_ANTI_CD3CD28_STIM_VS_UNSTIM	-0,104	0,004	0,04977	1
REGULATION_OF_SIGNAL_TRANSDUCTION	-0,104	0,004	0,00013	1
GSE39820_CTRL_VS_TGFBETA1_IL6_IL23A_	-0,104	0,004	0,00467	1
MORF_NOS2A	-0,104	0,004	0,00114	1
RIBONUCLEOPROTEIN_COMPLEX	-0,104	0,004	0,03321	1
TRANSLATION_REGULATOR_ACTIVITY	-0,104	0,004	0,12717	1
GSE17721_CPG_VS_GARDIQUIMOD_8H_BMDM	-0,104	0,004	0,00125	1
ELVIDGE_HIF1A_TARGETS_UP	-0,104	0,004	0,18179	1
MORF_MSH3	-0,104	0,004	0,00073	1
LABBE_WNT3A_TARGETS_DN	-0,104	0,004	0,00116	1
GSE17721_POLYIC_VS_CPG_1H_BMDM_DN	-0,104	0,004	0,00014	1
GSE22886_NAIVE_CD8_TCELL_VS_DC_UP	-0,104	0,005	0,00052	1
GSE9988_ANTI_TREM1_AND_LPS_VS_CTRL_	-0,104	0,005	0,0131	1
BASAL_LAMINA	-0,104	0,005	0,00015	1
HUNSBERGER_EXERCISE_REGULATED_GENES	-0,104	0,005	0,00515	1
MODULE_493	-0,104	0,005	0,0016	1
GCM_MAP1B	-0,104	0,005	0,00386	1
GSE17721_PAM3CSK4_VS_GADIQUIMOD_4H_	-0,104	0,005	0,0009	1
V$AR_Q6	-0,104	0,005	0,00021	1
REACTOME_ACTIVATION_OF_THE_MRNA_UP(	-0,104	0,005	0,00788	1

GAUSSMANN_MLL_AF4_FUSION_TARGETS_E_	-0,104	0,005	0,00103	1
GSE17721_CPG_VS_GARDIQUIMOD_6H_BMDM	-0,104	0,005	0,04824	1
REACTOME_HOST_INTERACTIONS_OF_HIV_FA	-0,104	0,005	0,12694	1
LEE_LIVER_CANCER_E2F1_UP	-0,104	0,005	0,01553	1
GSE10239_NAIVE_VS_MEMORY_CD8_TCELL_	-0,104	0,005	0,00021	1
chr2p22	-0,104	0,005	0,06942	1
GSE9006_HEALTHY_VS_TYPE_1_DIABETES_I	-0,104	0,005	0,08399	1
GSE9006_HEALTHY_VS_TYPE_1_DIABETES_I	-0,104	0,005	0,00023	1
PID_IL6_7PATHWAY	-0,104	0,005	0,00161	1
OZANNE_AP1_TARGETS_DN	-0,104	0,005	0,01343	1
GSE360_CTRL_VS_T_GONDII_DC_DN	-0,104	0,005	0,00215	1
ADENYL_NUCLEOTIDE_BINDING	-0,104	0,005	0,00078	1
V$RP58_01	-0,104	0,005	0,00022	1
chr17q23	-0,104	0,005	0,00176	1
MODULE_15	-0,104	0,005	0,01175	1
GSE29618_PRE_VS_DAY7_FLU_VACCINE_MC	-0,104	0,005	0,00029	1
KEGG_BASAL_CELL_CARCINOMA	-0,104	0,005	0,00101	1
LABBE_TGFB1_TARGETS_DN	-0,104	0,005	0,00071	1
DEOXYRIBONUCLEASE_ACTIVITY	-0,104	0,005	0,00302	1
REACTOME_STRIATED_MUSCLE_CONTRACTIOI	-0,104	0,005	0,00115	1
GSE3982_EOSINOPHIL_VS_EFF_MEMORY_CD4	-0,104	0,005	0,0137	1
REPLICATION_FORK	-0,103	0,005	0,04308	1
chr1p36	-0,103	0,005	0,00119	1
BIOCARTA_TALL1_PATHWAY	-0,103	0,005	0,00672	1
BIOCARTA_SET_PATHWAY	-0,103	0,005	0,01257	1
KEGG_HOMOLOGOUS_RECOMBINATION	-0,103	0,005	0,01791	1
MODULE_85	-0,103	0,005	0,00052	1
NEGATIVE_REGULATION_OF_DNA_REPLICATIC	-0,103	0,005	0,04111	1
GSE9037_WT_VS_IRAK4_KO_LPS_4H_STIM_	-0,103	0,005	0,00246	1
MORF_RUNX1	-0,103	0,005	0,00033	1
DAZARD_UV_RESPONSE_CLUSTER_G2	-0,103	0,005	0,01808	1
ODONNELL_METASTASIS_DN	-0,103	0,005	0,00468	1
BEGUM_TARGETS_OF_PAX3_FOXO1_FUSION_	-0,103	0,005	0,00099	1
BEIER_GLIOMA_STEM_CELL_UP	-0,103	0,005	0,00301	1
GSE360_DC_VS_MAC_L_MAJOR_UP	-0,103	0,005	0,00072	1
SHEPARD_CRUSH_AND_BURN_MUTANT_UP	-0,103	0,005	0,00086	1
BILBAN_B_CLL_LPL_DN	-0,103	0,005	0,00054	1
REACTOME_GABA_RECEPTOR_ACTIVATION	-0,103	0,005	0,00428	1
GLYCOPROTEIN_CATABOLIC_PROCESS	0,103	0,005	0,07653	1
TOOKER_GEMCITABINE_RESISTANCE_UP	-0,103	0,005	0,00899	1
GSE24081_CONTROLLER_VS_PROGRESSOR_H	-0,103	0,005	0,00036	1
GRAESSMANN_APOPTOSIS_BY_DOXORUBICIN_	-0,103	0,005	0,00141	1

BILBAN_B_CLL_LPL_UP	-0,103	0,005	0,00228	1
CHIARADONNA_NEOPLASTIC_TRANSFORMATI(	-0,103	0,005	0,00028	1
RUTELLA_RESPONSE_TO_HGF_VS_CSF2RB_A	-0,103	0,005	0,00249	1
PURINE_RIBONUCLEOTIDE_BINDING	-0,103	0,005	0,00044	1
chr10p13	-0,103	0,005	0,1366	1
AAGCCAT,MIR-135A,MIR-135B	-0,103	0,005	0,00028	1
GSE3982_DC_VS_MAC_LPS_STIM_UP	-0,103	0,005	0,00087	1
V$CDX2_Q5	-0,103	0,005	0,0002	1
GSE14000_TRANSLATED_RNA_VS_MRNA_DC	-0,103	0,005	0,08732	1
PID_ATR_PATHWAY	-0,103	0,005	0,02264	1
SMALL_NUCLEAR_RIBONUCLEOPROTEIN_COMI	-0,103	0,005	0,06313	1
MOREAUX_B_LYMPHOCYTE_MATURATION_BY_	-0,103	0,005	0,07449	1
MIKKELSEN_DEDIFFERENTIATED_STATE_UP	-0,103	0,005	0,00212	1
RESPONSE_TO_STRESS	-0,103	0,005	0,00573	1
GSE3982_MAST_CELL_VS_NEUTROPHIL_UP	-0,103	0,005	0,04181	1
chr21q11	-0,103	0,005	0,00914	1
MYLLYKANGAS_AMPLIFICATION_HOT_SPOT_1	0,103	0,005	0,00773	1
GSE15930_NAIVE_VS_48H_IN_VITRO_STIM_	-0,103	0,005	0,1671	1
AACYNNNNTTCCS_UNKNOWN	-0,103	0,005	0,00112	1
PRAMOONJAGO_SOX4_TARGETS_DN	-0,103	0,005	0,03303	1
GSE32423_IL7_VS_IL7_IL4_NAIVE_CD8_TCE	-0,103	0,005	0,00013	1
CALCIUM_MEDIATED_SIGNALING	-0,103	0,005	0,01093	1
UDAYAKUMAR_MED1_TARGETS_DN	-0,103	0,005	0,00062	1
GSE15659_CD45RA_NEG_CD4_TCELL_VS_N(	-0,103	0,005	0,01019	1
GSE1432_1H_VS_6H_IFNG_MICROGLIA_UP	-0,103	0,005	0,00025	1
POSITIVE_REGULATION_OF_CATALYTIC_ACTI'	-0,103	0,005	0,00085	1
LIGASE_ACTIVITY_FORMING_CARBON_OXYGEI	-0,103	0,005	0,38615	1
GNF2_TIMP2	-0,103	0,005	0,00145	1
MODULE_274	-0,103	0,005	0,00062	1
URS_ADIPOCYTE_DIFFERENTIATION_DN	-0,103	0,005	0,00254	1
MEMBRANE_PART	-0,103	0,005	0,00063	1
REACTOME_TRANSPORT_OF_MATURE_MRNA_	-0,103	0,005	0,06699	1
GSE360_L_DONOVANI_VS_B_MALAYI_LOW_	-0,103	0,005	0,00569	1
SUNG_METASTASIS_STROMA_UP	-0,103	0,005	0,00488	1
WONG_IFNA2_RESISTANCE_UP	-0,103	0,005	0,00017	1
TGAGATT,MIR-216	-0,103	0,005	0,00044	1
MODULE_64	-0,103	0,005	0,00158	1
KEGG_CELL_ADHESION_MOLECULES_CAMS	-0,103	0,005	0,00345	1
GSE17721_0.5H_VS_12H_LPS_BMDM_DN	-0,103	0,005	0,00268	1
RICKMAN_METASTASIS_DN	-0,103	0,005	0,03257	1
PHOSPHOLIPID_TRANSPORTER_ACTIVITY	-0,103	0,005	0,01693	1
GSE39820_CTRL_VS_IL1B_IL6_CD4_TCELL_	-0,103	0,005	0,00125	1

ACTIN_FILAMENT_BUNDLE_FORMATION	0,102	0,005	0,39448	1
ION_TRANSMEMBRANE_TRANSPORTER_ACTIV	-0,102	0,005	0,00016	1
V$CEBPDELTA_Q6	-0,102	0,005	0,00066	1
GSE15659_NONSUPPRESSIVE_TCELL_VS_AC	-0,102	0,005	0,00018	1
PASQUALUCCI_LYMPHOMA_BY_GC_STAGE_DI	-0,102	0,005	0,00181	1
GSE24634_NAIVE_CD4_TCELL_VS_DAY10_II	-0,102	0,005	0,0823	1
REACTOME_HS_GAG_BIOSYNTHESIS	-0,102	0,005	0,00879	1
SCHWAB_TARGETS_OF_BMYB_POLYMORPHIC_	-0,102	0,005	0,00088	1
GAUSSMANN_MLL_AF4_FUSION_TARGETS_F_	-0,102	0,005	0,06889	1
REACTOME_ANTIGEN_ACTIVATES_B_CELL_RI	-0,102	0,005	0,01972	1
GNF2_CBFB	-0,102	0,005	0,01602	1
MUNSHI_MULTIPLE_MYELOMA_UP	-0,102	0,005	0,00261	1
MICROTUBULE_ORGANIZING_CENTER_ORGANI	-0,102	0,005	0,00533	1
DNA_DEPENDENT_ATPASE_ACTIVITY	-0,102	0,005	0,02221	1
GSE360_DC_VS_MAC_T_GONDII_UP	-0,102	0,005	0,00054	1
MODULE_322	-0,102	0,005	0,00119	1
MODULE_89	-0,102	0,005	0,11711	1
GSE7460_TCONV_VS_TREG_LN_UP	-0,102	0,005	0,00067	1
MULLIGHAN_NPM1_SIGNATURE_3_UP	-0,102	0,005	0,00143	1
GSE15930_NAIVE_VS_24H_IN_VITRO_STIM_	-0,102	0,005	0,12692	1
REACTOME_P75_NTR_RECEPTOR_MEDIATED_	-0,102	0,005	0,00024	1
MIKKELSEN_MCV6_HCP_WITH_H3K27ME3	-0,102	0,005	0,00017	1
ENDONUCLEASE_ACTIVITY_GO_0016893	-0,102	0,005	0,20929	1
INTEGRAL_TO_PLASMA_MEMBRANE	-0,102	0,005	0,00061	1
LI_PROSTATE_CANCER_EPIGENETIC	-0,102	0,005	0,0009	1
CHEN_PDGF_TARGETS	-0,102	0,005	0,00342	1
GSE14308_NAIVE_CD4_TCELL_VS_NATURAL	-0,102	0,005	0,00126	1
RIBONUCLEOPROTEIN_BINDING	-0,102	0,005	0,06121	1
MODULE_301	-0,102	0,005	0,00177	1
GSE17721_POLYIC_VS_GARDIQUIMOD_0.5H_	-0,102	0,005	0,00277	1
PYEON_CANCER_HEAD_AND_NECK_VS_CERV	-0,102	0,005	0,05886	1
PRC1_BMI_UP.V1_DN	-0,102	0,005	0,00059	1
GSE36392_TYPE_2_MYELOID_VS_EOSINOPHI	-0,102	0,005	0,00054	1
TOOKER_GEMCITABINE_RESISTANCE_DN	-0,102	0,005	0,05874	1
REACTOME_SYNTHESIS_OF_DNA	-0,102	0,005	0,11506	1
GRATIAS_RETINOBLASTOMA_16Q24	-0,102	0,005	0,29074	1
PID_ALPHASYNUCLEIN_PATHWAY	-0,102	0,005	0,0115	1
PROTEIN_KINASE_REGULATOR_ACTIVITY	-0,102	0,005	0,00116	1
chr7q32	-0,102	0,005	0,02797	1
YUAN_ZNF143_PARTNERS	-0,102	0,005	0,05707	1
REACTOME_METAL_ION_SLC_TRANSPORTERS	0,102	0,005	0,00507	1
JAK_STAT_CASCADE	-0,102	0,005	0,00294	1

GARGALOVIC_RESPONSE_TO_OXIDIZED_PHOS	-0,102	0,005	0,01119	1
SNF5_DN.V1_UP	-0,102	0,005	0,00976	1
GSE22886_NAIVE_CD4_TCELL_VS_48H_ACT	-0,102	0,005	0,18879	1
ISHIDA_TARGETS_OF_SYT_SSX_FUSIONS	0,102	0,005	0,01936	1
PID_VEGF_VEGFR_PATHWAY	-0,102	0,005	0,00098	1
chr7q35	-0,102	0,006	0,0052	1
CSR_EARLY_UP.V1_UP	-0,102	0,006	0,19044	1
GSE1432_CTRL_VS_IFNG_1H_MICROGLIA_UP	-0,102	0,006	0,00051	1
INTRINSIC_TO_PLASMA_MEMBRANE	-0,102	0,006	0,00059	1
GSE3982_EOSINOPHIL_VS_MAC_DN	-0,102	0,006	0,10406	1
RELA_DN.V1_DN	-0,102	0,006	0,00053	1
MORF_ANP32B	-0,102	0,006	0,05744	1
BILANGES_SERUM_AND_RAPAMYCIN_SENSITI\	-0,102	0,006	0,00132	1
MORF_FSHR	-0,101	0,006	0,00084	1
MYELOID_LEUKOCYTE_DIFFERENTIATION	-0,101	0,006	0,04649	1
GSE6269_HEALTHY_VS_STREP_PNEUMO_INF.	-0,101	0,006	0,00065	1
V$MEF2_01	-0,101	0,006	0,00142	1
PILON_KLF1_TARGETS_DN	-0,101	0,006	0,03712	1
REACTOME_RECYCLING_PATHWAY_OF_L1	-0,101	0,006	0,18413	1
GSE26928_NAIVE_VS_EFF_MEMORY_CD4_TC	-0,101	0,006	0,00344	1
V$HIF1_Q3	-0,101	0,006	0,01056	1
GSE27786_CD4_TCELL_VS_MONO_MAC_DN	-0,101	0,006	0,00973	1
NITROGEN_COMPOUND_CATABOLIC_PROCESS	0,101	0,006	0,07327	1
GGTAACC,MIR-409-5P	-0,101	0,006	0,00932	1
BYSTRYKH_HEMATOPOIESIS_STEM_CELL_QTL	-0,101	0,006	0,01446	1
UEDA_PERIFERAL_CLOCK	-0,101	0,006	0,03471	1
chr10q21	-0,101	0,006	0,0035	1
TIAN_TNF_SIGNALING_VIA_NFKB	-0,101	0,006	0,01832	1
GSE17721_CTRL_VS_PAM3CSK4_4H_BMDM_	-0,101	0,006	0,00062	1
GRADE_COLON_VS_RECTAL_CANCER_UP	-0,101	0,006	0,00604	1
CHANG_IMMORTALIZED_BY_HPV31_UP	-0,101	0,006	0,00429	1
PID_ATF2_PATHWAY	-0,101	0,006	0,00149	1
REACTOME_DIGESTION_OF_DIETARY_CARBOH	-0,101	0,006	0,03276	1
RAMPON_ENRICHED_LEARNING_ENVIRONMENT	-0,101	0,006	0,0385	1
V$OSF2_Q6	-0,101	0,006	0,00099	1
V$IRF2_01	-0,101	0,006	0,00082	1
RELA_DN.V1_UP	-0,101	0,006	0,00152	1
V$AP1_Q6	-0,101	0,006	0,01951	1
GSE17721_0.5H_VS_8H_GARDIQUIMOD_BMD	-0,101	0,006	0,00462	1
GAVIN_FOXP3_TARGETS_CLUSTER_P4	-0,101	0,006	0,00397	1
REACTOME_SRP_DEPENDENT_COTRANSLATIO	-0,101	0,006	0,00456	1
GSE2706_R848_VS_LPS_2H_STIM_DC_DN	-0,101	0,006	0,00777	1

GCM_SMO	-0,101	0,006	0,00091	1
CAIRO_PML_TARGETS_BOUND_BY_MYC_DN	-0,101	0,006	0,00166	1
GROWTH_FACTOR_ACTIVITY	-0,101	0,006	0,00021	1
PID_PTP1BPATHWAY	-0,101	0,006	0,00233	1
HAHTOLA_SEZARY_SYNDROM_DN	-0,101	0,006	0,00114	1
GSE2706_UNSTIM_VS_8H_LPS_DC_DN	-0,101	0,006	0,01617	1
LOCKWOOD_AMPLIFIED_IN_LUNG_CANCER	-0,101	0,006	0,03744	1
GSE360_CTRL_VS_B_MALAYI_HIGH_DOSE_M	-0,101	0,006	0,00148	1
AACWWCAANK_UNKNOWN	-0,101	0,006	0,00014	1
MIKKELSEN_IPS_LCP_WITH_H3K27ME3	-0,101	0,006	0,00015	1
GSE8678_IL7R_LOW_VS_HIGH_EFF_CD8_TCI	-0,101	0,006	0,00268	1
MODULE_560	-0,101	0,006	0,03112	1
NIKOLSKY_BREAST_CANCER_20Q12_Q13_AI	-0,101	0,006	0,01109	1
VERHAAK_AML_WITH_NPM1_MUTATED_DN	-0,101	0,006	0,0002	1
GSE360_CTRL_VS_B_MALAYI_HIGH_DOSE_D	-0,101	0,006	0,00371	1
ALCOHOL_METABOLIC_PROCESS	-0,101	0,006	0,00274	1
GSE26669_CD4_VS_CD8_TCELL_IN_MLR_CC	-0,101	0,006	0,00131	1
MODULE_41	-0,101	0,006	0,0014	1
GSE1432_6H_VS_24H_IFNG_MICROGLIA_UP	-0,101	0,006	0,02676	1
GSE17721_PAM3CSK4_VS_GADIQUIMOD_12I	-0,101	0,006	0,00093	1
REACTOME_PURINE_SALVAGE	-0,101	0,006	0,05465	1
V$CACBINDINGPROTEIN_Q6	-0,101	0,006	0,00032	1
MODULE_366	-0,101	0,006	0,00016	1
GSE15930_NAIVE_VS_72H_IN_VITRO_STIM_	-0,101	0,006	0,08905	1
V$MSX1_01	-0,101	0,006	0,00053	1
NEGATIVE_REGULATION_OF_PHOSPHATE_ME	-0,101	0,006	0,01338	1
GSE22886_NAIVE_CD4_TCELL_VS_DC_UP	-0,101	0,006	0,00018	1
MORF_JUND	-0,101	0,006	0,00215	1
TRANSFERASE_ACTIVITY_TRANSFERRING_HEX	-0,101	0,006	0,04928	1
VERRECCHIA_DELAYED_RESPONSE_TO_TGFB	-0,1	0,006	0,01924	1
GSE32423_MEMORY_VS_NAIVE_CD8_TCELL_	-0,1	0,006	0,01469	1
SUBSTRATE_SPECIFIC_TRANSMEMBRANE_TRA	-0,1	0,006	0,00019	1
WOTTON_RUNX_TARGETS_DN	-0,1	0,006	0,00172	1
MORF_PTPN11	-0,1	0,006	0,25016	1
REACTOME_PLATELET_HOMEOSTASIS	-0,1	0,006	0,00218	1
GSE17721_POLYIC_VS_GARDIQUIMOD_6H_BI	-0,1	0,006	0,12174	1
GSE9988_LPS_VS_CTRL_TREATED_MONOCY	-0,1	0,006	0,02321	1
YEMELYANOV_GR_TARGETS_DN	-0,1	0,006	0,00607	1
PID_INSULIN_GLUCOSE_PATHWAY	-0,1	0,006	0,11941	1
MODULE_221	0,1	0,006	0,16759	1
LIU_NASOPHARYNGEAL_CARCINOMA	-0,1	0,006	0,00148	1
MORF_BCL2	-0,1	0,006	0,00015	1

LI_WILMS_TUMOR_ANAPLASTIC_DN	-0,1	0,006	0,00636	1
GSE6269_FLU_VS_STREP_AUREUS_INF_PBM(	-0,1	0,006	0,0223	1
V$EN1_01	-0,1	0,006	0,00047	1
KIM_BIPOLAR_DISORDER_OLIGODENDROCYTE_	-0,1	0,006	0,00604	1
BROWNE_HCMV_INFECTION_16HR_UP	-0,1	0,006	0,0314	1
PID_ARF6_TRAFFICKINGPATHWAY	-0,1	0,006	0,00042	1
MOROSETTI_FACIOSCAPULOHUMERAL_MUSCU	-0,1	0,006	0,03891	1
GSE17721_CTRL_VS_PAM3CSK4_2H_BMDM_	-0,1	0,006	0,00186	1
OLIGOSACCHARIDE_METABOLIC_PROCESS	-0,1	0,006	0,00074	1
GSE27786_BCELL_VS_CD8_TCELL_DN	-0,1	0,006	0,00015	1
INTRACELLULAR_RECEPTOR_MEDIATED_SIGN/	-0,1	0,006	0,00466	1
GSE17721_0.5H_VS_12H_GARDIQUIMOD_BM	-0,1	0,006	0,00409	1
MODULE_457	-0,1	0,006	0,00097	1
GSE3982_MAST_CELL_VS_BCELL_DN	-0,1	0,006	0,00214	1
GSE18791_UNSTIM_VS_NEWCATSLE_VIRUS_	-0,1	0,006	0,00031	1
KIM_ALL_DISORDERS_CALB1_CORR_UP	-0,1	0,006	0,00547	1
GSE3982_EFF_MEMORY_VS_CENT_MEMORY_	-0,1	0,006	0,00167	1
GAUSSMANN_MLL_AF4_FUSION_TARGETS_A_	-0,1	0,006	0,00527	1
MEMBRANE	-0,1	0,006	0,0008	1
REACTOME_FORMATION_OF_THE_TERNARY_(	-0,1	0,006	0,0076	1
GSE14769_UNSTIM_VS_20MIN_LPS_BMDM_[	-0,1	0,006	0,00142	1
HINATA_NFKB_MATRIX	-0,1	0,006	0,21579	1
RIBOSOME_BIOGENESIS_AND_ASSEMBLY	-0,1	0,006	0,04406	1
B_CELL_DIFFERENTIATION	-0,1	0,006	0,01549	1
GTCTACC,MIR-379	-0,1	0,006	0,0233	1
V$PAX2_01	-0,1	0,006	0,00682	1
GSE30083_SP1_VS_SP2_THYMOCYTE_DN	-0,1	0,006	0,00064	1
REACTOME_TRANS_GOLGI_NETWORK_VESICL	0,1	0,006	0,00208	1
PURINE_NUCLEOTIDE_BINDING	-0,1	0,006	0,00038	1
MORI_PLASMA_CELL_DN	-0,1	0,006	0,01623	1
GCGCTTT,MIR-518B,MIR-518C,MIR-518D	-0,1	0,006	0,18576	1
SU_KIDNEY	-0,1	0,006	0,05428	1
FULCHER_INFLAMMATORY_RESPONSE_LECTIN	-0,1	0,006	0,0399	1
YANG_MUC2_TARGETS_DUODENUM_3MO_DN	0,1	0,006	0,0592	1
PROTEIN_RNA_COMPLEX_ASSEMBLY	-0,1	0,006	0,04424	1
PLASMA_MEMBRANE_PART	-0,1	0,006	0,00057	1
V$PR_02	-0,1	0,006	0,00027	1
GOERING_BLOOD_HDL_CHOLESTEROL_QTL_T	-0,1	0,006	0,00195	1
GSE27786_NKTCELL_VS_MONO_MAC_UP	-0,1	0,007	0,01524	1
SU_THYMUS	-0,1	0,007	0,0112	1
ANDROGEN_RECEPTOR_SIGNALING_PATHWAY	-0,1	0,007	0,03785	1
PID_IL2_STAT5PATHWAY	-0,1	0,007	0,01156	1

IZADPANAH_STEM_CELL_ADIPOSE_VS_BONE_	-0,1	0,007	0,0487	1
GSE12366_PLASMA_CELL_VS_MEMORY_BCEI	-0,099	0,007	0,00283	1
FIGUEROA_AML_METHYLATION_CLUSTER_6_I	-0,099	0,007	0,00289	1
GNF2_TTN	-0,099	0,007	0,00031	1
MODULE_92	-0,099	0,007	0,00247	1
NEGATIVE_REGULATION_OF_IMMUNE_SYSTEM	-0,099	0,007	0,00865	1
GSE7460_WT_VS_FOXP3_HET_ACT_TCONV_	-0,099	0,007	0,01223	1
V$GATA1_04	-0,099	0,007	0,00073	1
GSE17721_CPG_VS_GARDIQUIMOD_6H_BMDN	-0,099	0,007	0,00374	1
BROWNE_HCMV_INFECTION_6HR_UP	-0,099	0,007	0,01526	1
DACOSTA_UV_RESPONSE_VIA_ERCC3_TTD_L	-0,099	0,007	0,0462	1
ACEVEDO_NORMAL_TISSUE_ADJACENT_TO_I	-0,099	0,007	0,01089	1
PYEON_CANCER_HEAD_AND_NECK_VS_CERV	-0,099	0,007	0,00992	1
GALI_TP53_TARGETS_APOPTOTIC_UP	-0,099	0,007	0,01244	1
KRIEG_KDM3A_TARGETS_NOT_HYPOXIA	-0,099	0,007	0,01383	1
CHEOK_RESPONSE_TO_HD_MTX_UP	-0,099	0,007	0,02909	1
LE_SKI_TARGETS_UP	-0,099	0,007	0,00026	1
GSE10856_CTRL_VS_TNFRSF6B_IN_MACROP	-0,099	0,007	0,01607	1
RAMALHO_STEMNESS_UP	-0,099	0,007	0,10986	1
GSE26928_EFF_MEMORY_VS_CXCR5_POS_CI	-0,099	0,007	0,00081	1
REACTOME_PLATELET_ADHESION_TO_EXPOS	-0,099	0,007	0,00141	1
MODULE_375	-0,099	0,007	0,00339	1
INOSITOL_OR_PHOSPHATIDYLINOSITOL_PHOS	-0,099	0,007	0,03152	1
PID_LYMPHANGIOGENESIS_PATHWAY	-0,099	0,007	0,00193	1
MORF_ERCC4	-0,099	0,007	0,00063	1
STEROID_HORMONE_RECEPTOR_SIGNALING_P	-0,099	0,007	0,00564	1
STEIN_ESRRA_TARGETS	-0,099	0,007	0,04107	1
YORDY_RECIPROCAL_REGULATION_BY_ETS1_	-0,099	0,007	0,00191	1
PID_INTEGRIN_A4B1_PATHWAY	-0,099	0,007	0,02974	1
GSE39820_TGFBETA3_IL6_VS_TGFBETA3_IL	-0,099	0,007	0,00209	1
MORF_TTN	-0,099	0,007	0,00083	1
KEGG_INSULIN_SIGNALING_PATHWAY	-0,099	0,007	0,00022	1
KONDO_EZH2_TARGETS	-0,099	0,007	0,00034	1
TUBE_MORPHOGENESIS	-0,099	0,007	0,00283	1
GSE11924_TH2_VS_TH17_CD4_TCELL_UP	-0,099	0,007	0,00383	1
PASTURAL_RIZ1_TARGETS_UP	-0,099	0,007	0,00367	1
GALIE_TUMOR_ANGIOGENESIS	-0,099	0,007	0,00065	1
BIOCARTA_CK1_PATHWAY	-0,099	0,007	0,01692	1
AAAGACA,MIR-511	-0,099	0,007	0,00066	1
V$ETS2_B	-0,099	0,007	0,00103	1
MODULE_186	-0,099	0,007	0,00996	1
MODULE_110	-0,099	0,007	0,36185	1

GSE15324_ELF4_KO_VS_WT_ACTIVATED_CI	-0,099	0,007	0,00029	1
RIZ_ERYTHROID_DIFFERENTIATION_HBZ	-0,099	0,007	0,01311	1
PROTEIN_TYROSINE_PHOSPHATASE_ACTIVITY	-0,099	0,007	0,00096	1
PERINUCLEAR_REGION_OF_CYTOPLASM	-0,099	0,007	0,04265	1
TAATGTG,MIR-323	-0,099	0,007	0,00428	1
INTRINSIC_TO_MEMBRANE	-0,099	0,007	0,00082	1
V$TAL1ALPHAE47_01	-0,099	0,007	0,00019	1
INTEGRAL_TO_MEMBRANE	-0,099	0,007	0,00086	1
ZHAN_MULTIPLE_MYELOMA_HP_UP	-0,099	0,007	0,0014	1
GSE22886_NAIVE_CD8_TCELL_VS_NEUTROP	-0,099	0,007	0,0082	1
ZINC_ION_BINDING	-0,099	0,007	0,00703	1
GSE20715_0H_VS_24H_OZONE_LUNG_DN	-0,099	0,007	0,00173	1
NEUROTRANSMITTER_RECEPTOR_ACTIVITY	-0,099	0,007	0,00944	1
GSE29618_BCELL_VS_MDC_UP	-0,099	0,007	0,00525	1
REGULATION_OF_IMMUNE_RESPONSE	-0,099	0,007	0,01088	1
EPITHELIAL_TO_MESENCHYMAL_TRANSITION	-0,099	0,007	0,00154	1
GSE15659_NAIVE_CD4_TCELL_VS_NONSUPF	-0,099	0,007	0,00033	1
AGGCACT,MIR-515-3P	-0,099	0,007	0,00629	1
GRAESSMANN_APOPTOSIS_BY_SERUM_DEPRIV	-0,099	0,007	0,00074	1
MCBRYAN_PUBERTAL_BREAST_3_4WK_DN	-0,099	0,007	0,00023	1
TAAWWATAG_V$RSRFC4_Q2	-0,099	0,007	0,00034	1
DRUG_BINDING	-0,099	0,007	0,00833	1
GSE10239_NAIVE_VS_KLRG1INT_EFF_CD8_T	-0,099	0,007	0,00013	1
GSE3982_BASOPHIL_VS_NKCELL_DN	-0,099	0,007	0,00059	1
RIZ_ERYTHROID_DIFFERENTIATION_CCNE1	-0,099	0,007	0,01045	1
PENG_RAPAMYCIN_RESPONSE_DN	-0,099	0,007	0,37619	1
KYNG_NORMAL_AGING_DN	-0,099	0,007	0,00029	1
PDGF_ERK_DN.V1_UP	-0,099	0,007	0,01218	1
LIU_COMMON_CANCER_GENES	-0,098	0,007	0,00226	1
REGULATION_OF_CELL_ADHESION	-0,098	0,007	0,00597	1
MODULE_236	-0,098	0,007	0,00197	1
REACTOME_PEPTIDE_CHAIN_ELONGATION	-0,098	0,007	0,00288	1
MODULE_226	-0,098	0,007	0,0514	1
LIU_IL13_MEMORY_MODEL_DN	0,098	0,007	0,03203	1
INTEGRAL_TO_GOLGI_MEMBRANE	0,098	0,007	0,07079	1
RASHI_NFKB1_TARGETS	-0,098	0,007	0,0078	1
GSE17721_LPS_VS_GARDIQUIMOD_6H_BMDM	-0,098	0,007	0,00103	1
NING_CHRONIC_OBSTRUCTIVE_PULMONARY_I	-0,098	0,007	0,00019	1
V$SRF_C	-0,098	0,007	0,00021	1
AUJLA_IL22_AND_IL17A_SIGNALING	-0,098	0,007	0,1944	1
GCM_AIP	-0,098	0,007	0,00531	1
chr12p	0,098	0,007	0,05318	1

GSE14000_TRANSLATED_RNA_VS_MRNA_4H	-0,098	0,007	0,02314	1
HOLLEMAN_VINCRISTINE_RESISTANCE_ALL_D	-0,098	0,007	0,00403	1
JOHNSTONE_PARVB_TARGETS_2_DN	-0,098	0,007	0,02471	1
BOQUEST_STEM_CELL_CULTURED_VS_FRESH	-0,098	0,007	0,00017	1
VANDESLUIS_COMMD1_TARGETS_GROUP_3_I	-0,098	0,007	0,00967	1
BROWNE_HCMV_INFECTION_48HR_DN	-0,098	0,007	0,00023	1
REACTOME_INFLUENZA_VIRAL_RNA_TRANSCI	-0,098	0,007	0,00458	1
POTTI_TOPOTECAN_SENSITIVITY	-0,098	0,007	0,00107	1
chrxq26	-0,098	0,007	0,05133	1
SENSORY_PERCEPTION	-0,098	0,007	0,00767	1
VANDESLUIS_COMMD1_TARGETS_GROUP_3_I	-0,098	0,007	0,02194	1
REGULATION_OF_PROTEIN_METABOLIC_PROC	-0,098	0,007	0,00327	1
KEGG_SYSTEMIC_LUPUS_ERYTHEMATOSUS	-0,098	0,007	0,0517	1
BROWNE_HCMV_INFECTION_30MIN_UP	-0,098	0,007	0,00028	1
NEGATIVE_REGULATION_OF_PROGRAMMED_C	-0,098	0,007	0,0113	1
PLASMA_MEMBRANE	-0,098	0,007	0,00073	1
LEE_METASTASIS_AND_ALTERNATIVE_SPLICI	-0,098	0,007	0,00033	1
V$ARNT_01	-0,098	0,007	0,00134	1
INNATE_IMMUNE_RESPONSE	-0,098	0,007	0,00416	1
TCTGATA,MIR-361	-0,098	0,007	0,00159	1
ONKEN_UVEAL_MELANOMA_UP	-0,098	0,007	0,01834	1
SMITH_LIVER_CANCER	-0,098	0,007	0,14101	1
YATTNATC_UNKNOWN	-0,098	0,007	0,0002	1
GSE3982_BCELL_VS_EFF_MEMORY_CD4_TCE	-0,098	0,007	0,00987	1
REACTOME_SEMA4D_IN_SEMAPHORIN_SIGNAI	-0,098	0,007	0,00048	1
MARTINEZ_RB1_AND_TP53_TARGETS_UP	-0,098	0,007	0,00121	1
SAKAI_TUMOR_INFILTRATING_MONOCYTES_D	-0,098	0,007	0,14105	1
CHENG_RESPONSE_TO_NICKEL_ACETATE	-0,098	0,007	0,01001	1
GRAHAM_CML_QUIESCENT_VS_NORMAL_DIVI	-0,098	0,008	0,02722	1
GSE17721_CTRL_VS_POLYIC_8H_BMDM_UP	-0,098	0,008	0,02485	1
FAELT_B_CLL_WITH_VH_REARRANGEMENTS_	-0,098	0,008	0,00049	1
ACETYLTRANSFERASE_ACTIVITY	0,098	0,008	0,05347	1
BIOCARTA_PDGF_PATHWAY	-0,098	0,008	0,00171	1
MORF_TERF2IP	-0,098	0,008	0,04149	1
HUMMERICH_SKIN_CANCER_PROGRESSION_DN	-0,098	0,008	0,00023	1
HILLION_HMGA1B_TARGETS	-0,098	0,008	0,01292	1
MEDIATOR_COMPLEX	-0,098	0,008	0,16934	1
REACTOME_DNA_STRAND_ELONGATION	-0,098	0,008	0,05331	1
MA_PITUITARY_FETAL_VS_ADULT_UP	-0,098	0,008	0,00497	1
HOLLMANN_APOPTOSIS_VIA_CD40_DN	-0,098	0,008	0,01205	1
GSE17721_CTRL_VS_PAM3CSK4_24H_BMDM	-0,098	0,008	0,05135	1
TAKEDA_TARGETS_OF_NUP98_HOXA9_FUSIC	-0,098	0,008	0,01894	1

MARSON_BOUND_BY_FOXP3_UNSTIMULATED	-0,098	0,008	0,00113	1
HEMOPOIESIS	-0,098	0,008	0,00564	1
REACTOME_VEGF_LIGAND_RECEPTOR_INTERA	-0,098	0,008	0,0014	1
CAGCTTT,MIR-320	-0,098	0,008	0,00133	1
SEKI_INFLAMMATORY_RESPONSE_LPS_UP	-0,098	0,008	0,01582	1
PETRETTO_CARDIAC_HYPERTROPHY	-0,098	0,008	0,00095	1
HAN_JNK_SINGALING_UP	-0,098	0,008	0,00126	1
KOYAMA_SEMA3B_TARGETS_UP	-0,098	0,008	0,00018	1
TGAATGT,MIR-181A,MIR-181B,MIR-181C,MIR-1	-0,098	0,008	0,00213	1
KOBAYASHI_RESPONSE_TO_ROMIDEPSIN	-0,097	0,008	0,01375	1
JAEGER_METASTASIS_UP	-0,097	0,008	0,17446	1
ZHU_CMV_24_HR_UP	-0,097	0,008	0,13956	1
ZHAN_MULTIPLE_MYELOMA_SUBGROUPS	-0,097	0,008	0,24785	1
GSE30083_SP1_VS_SP2_THYMOCYTE_UP	-0,097	0,008	0,00115	1
V$OCT_Q6	-0,097	0,008	0,00025	1
REACTOME_ORC1_REMOVAL_FROM_CHROMAT	-0,097	0,008	0,27861	1
ACTGAAA,MIR-30A-3P,MIR-30E-3P	-0,097	0,008	0,00111	1
REACTOME_RESPONSE_TO_ELEVATED_PLATE	-0,097	0,008	0,00145	1
GSE17721_PAM3CSK4_VS_CPG_12H_BMDM_	-0,097	0,008	0,00212	1
AMIT_SERUM_RESPONSE_60_MCF10A	-0,097	0,008	0,00149	1
MIKKELSEN_IPS_LCP_WITH_H3K4ME3_AND_H	-0,097	0,008	0,00068	1
CAGTGTT,MIR-141,MIR-200A	-0,097	0,008	0,00099	1
LIN_SILENCED_BY_TUMOR_MICROENVIRONME	-0,097	0,008	0,21043	1
AAGCACT,MIR-520F	-0,097	0,008	0,00138	1
RACCACAR_V$AML_Q6	-0,097	0,008	0,00039	1
WESTON_VEGFA_TARGETS	-0,097	0,008	0,00191	1
TRANSMEMBRANE_RECEPTOR_ACTIVITY	-0,097	0,008	0,00049	1
GAVIN_FOXP3_TARGETS_CLUSTER_T7	-0,097	0,008	0,03439	1
NIELSEN_MALIGNAT_FIBROUS_HISTIOCYTOMA	-0,097	0,008	0,01716	1
MORF_MAGEA9	-0,097	0,008	0,00021	1
GSE3982_MAC_VS_NEUTROPHIL_LPS_STIM_I	-0,097	0,008	0,00019	1
MODULE_362	-0,097	0,008	0,00211	1
FIRESTEIN_CTNNB1_PATHWAY	-0,097	0,008	0,10171	1
AGCTCCT,MIR-28	-0,097	0,008	0,00023	1
GSE17721_PAM3CSK4_VS_CPG_16H_BMDM_	-0,097	0,008	0,00402	1
GSE17721_CTRL_VS_GARDIQUIMOD_24H_BM	-0,097	0,008	0,09816	1
REGULATION_OF_CELLULAR_PROTEIN_METAB	-0,097	0,008	0,00441	1
MODULE_448	-0,097	0,008	0,00269	1
MORF_PTPRB	-0,097	0,008	0,00109	1
REACTOME_SYNTHESIS_OF_BILE_ACIDS_AND	0,097	0,008	0,20047	1
FONTAINE_FOLLICULAR_THYROID_ADENOMA_	-0,097	0,008	0,01131	1
GSE17721_LPS_VS_GARDIQUIMOD_8H_BMDM	-0,097	0,008	0,07598	1

GTTAAAG,MIR-302B	-0,097	0,008	0,0018	1
REACTOME_SIGNALLING_TO_RAS	-0,097	0,008	0,01052	1
GSE36476_CTRL_VS_TSST_ACT_16H_MEMO	-0,097	0,008	0,39224	1
chr13q21	-0,097	0,008	0,03165	1
KEGG_DNA_REPLICATION	-0,097	0,008	0,0464	1
MANALO_HYPOXIA_UP	-0,097	0,008	0,00162	1
MODULE_331	-0,097	0,008	0,01719	1
GNF2_TPT1	-0,097	0,008	0,00306	1
chr16q22	-0,097	0,008	0,09559	1
LOPES_METHYLATED_IN_COLON_CANCER_UF	-0,097	0,008	0,00189	1
WANG_RESPONSE_TO_FORSKOLIN_UP	-0,097	0,008	0,00248	1
TRANSFERASE_ACTIVITY_TRANSFERRING_PEN	-0,097	0,008	0,26373	1
V$PEA3_Q6	-0,097	0,008	0,00591	1
MARSON_FOXP3_CORE_DIRECT_TARGETS	-0,097	0,008	0,0072	1
GSE17721_PAM3CSK4_VS_GADIQUIMOD_8H_	-0,097	0,008	0,06868	1
V$COUP_01	-0,097	0,008	0,00028	1
MEISSNER_NPC_HCP_WITH_H3K4ME2_AND_H	-0,097	0,008	0,00077	1
GSE27786_LIN_NEG_VS_NEUTROPHIL_DN	-0,097	0,008	0,00051	1
KRAS.PROSTATE_UP.V1_UP	-0,097	0,008	0,00017	1
GSE17721_PAM3CSK4_VS_GADIQUIMOD_16H	-0,097	0,008	0,03864	1
JAK2_DN.V1_UP	-0,097	0,008	0,0002	1
CGGTGTG,MIR-220	0,097	0,008	0,19035	1
GSE20366_CD103_POS_VS_CD103_KLRG1_	-0,097	0,008	0,0002	1
GROSS_HYPOXIA_VIA_ELK3_ONLY_UP	-0,097	0,008	0,02585	1
MIKKELSEN_NPC_HCP_WITH_H3K27ME3	-0,097	0,008	0,00039	1
WORSCHECH_TUMOR_EVASION_AND_TOLERC	-0,097	0,008	0,02824	1
MIKKELSEN_PLURIPOTENT_STATE_DN	-0,096	0,008	0,0066	1
VISALA_AGING_LYMPHOCYTE_DN	-0,096	0,008	0,03209	1
GSE17721_LPS_VS_PAM3CSK4_6H_BMDM_U	-0,096	0,008	0,0047	1
KRAS.50_UP.V1_DN	-0,096	0,008	0,141	1
GSE17721_LPS_VS_CPG_8H_BMDM_UP	-0,096	0,008	0,00633	1
GSE17721_LPS_VS_POLYIC_16H_BMDM_DN	-0,096	0,008	0,01787	1
REGULATION_OF_MULTICELLULAR_ORGANISM	-0,096	0,008	0,00221	1
GSE3982_NEUTROPHIL_VS_BCELL_UP	-0,096	0,009	0,01343	1
GCM_TEC	-0,096	0,009	0,00223	1
MEISSNER_BRAIN_HCP_WITH_H3K27ME3	-0,096	0,009	0,01434	1
V$TAL1BETAE47_01	-0,096	0,009	0,0002	1
GSE37416_CTRL_VS_3H_F_TULARENSIS_LV!	-0,096	0,009	0,0498	1
MORF_G22P1	-0,096	0,009	0,14239	1
GENTILE_UV_RESPONSE_CLUSTER_D2	-0,096	0,009	0,00672	1
CERIBELLI_GENES_INACTIVE_AND_BOUND_BY	-0,096	0,009	0,00062	1
PID_FOXOPATHWAY	-0,096	0,009	0,02552	1

PIONTEK_PKD1_TARGETS_DN	-0,096	0,009	0,02203	1
REACTOME_CDC6_ASSOCIATION_WITH_THE_	-0,096	0,009	0,07072	1
GSE22886_DAY1_VS_DAY7_MONOCYTE_IN_	-0,096	0,009	0,02012	1
GCGSCMNTTT_UNKNOWN	-0,096	0,009	0,00721	1
PILON_KLF1_TARGETS_UP	-0,096	0,009	0,00016	1
E2F1_UP.V1_DN	-0,096	0,009	0,00139	1
ATATGCA,MIR-448	-0,096	0,009	0,00457	1
WESTON_VEGFA_TARGETS_3HR	-0,096	0,009	0,01071	1
LU_TUMOR_ENDOTHELIAL_MARKERS_UP	-0,096	0,009	0,00556	1
SMID_BREAST_CANCER_RELAPSE_IN_PLEURA	-0,096	0,009	0,32017	1
GSE6269_HEALTHY_VS_E_COLI_INF_PBMC_[	-0,096	0,009	0,01006	1
KONDO_PROSTATE_CANCER_WITH_H3K27ME	-0,096	0,009	0,00104	1
COATED_MEMBRANE	-0,096	0,009	0,0833	1
MEMBRANE_COAT	-0,096	0,009	0,0833	1
PID_CXCR4_PATHWAY	-0,096	0,009	0,00523	1
TCATCTC,MIR-143	-0,096	0,009	0,00032	1
PID_S1P_S1P1_PATHWAY	-0,096	0,009	0,0091	1
CASPASE_REGULATOR_ACTIVITY	-0,096	0,009	0,03291	1
KEGG_RIBOSOME	-0,096	0,009	0,00428	1
YANG_BREAST_CANCER_ESR1_BULK_UP	0,096	0,009	0,59709	1
GSE17721_LPS_VS_GARDIQUIMOD_1H_BMDN	-0,096	0,009	0,00428	1
TCGA_GLIOBLASTOMA_COPY_NUMBER_UP	-0,096	0,009	0,01688	1
GINESTIER_BREAST_CANCER_ZNF217_AMPLIF	0,096	0,009	0,08355	1
REACTOME_M_G1_TRANSITION	-0,096	0,009	0,2446	1
PID_INTEGRIN_CS_PATHWAY	-0,096	0,009	0,00433	1
KEGG_GLYCOSAMINOGLYCAN_BIOSYNTHESIS_	-0,096	0,009	0,01103	1
KORKOLA_SEMINOMA_UP	-0,096	0,009	0,01364	1
N_METHYLTRANSFERASE_ACTIVITY	-0,096	0,009	0,00848	1
GSE22886_CD8_VS_CD4_NAIVE_TCELL_DN	-0,096	0,009	0,00021	1
GSE15930_STIM_VS_STIM_AND_IFNAB_24H_	-0,096	0,009	0,00135	1
STEINER_ERYTHROCYTE_MEMBRANE_GENES	-0,096	0,009	0,01025	1
PID_RANBP2PATHWAY	-0,096	0,009	0,12088	1
GNF2_UBE2I	-0,096	0,009	0,02429	1
BIOCARTA_IL2RB_PATHWAY	-0,096	0,009	0,00541	1
SCIAN_CELL_CYCLE_TARGETS_OF_TP53_ANI	-0,096	0,009	0,00454	1
REGULATION_OF_G_PROTEIN_COUPLED_RECE	-0,096	0,009	0,00032	1
EPIDERMAL_GROWTH_FACTOR_RECEPTOR_SI(	-0,096	0,009	0,00918	1
MAEKAWA_ATF2_TARGETS	0,096	0,009	0,03765	1
REACTOME_TRAFFICKING_OF_GLUR2_CONTAI	-0,096	0,009	0,00068	1
GSE27786_CD8_TCELL_VS_NKTCELL_UP	-0,096	0,009	0,00034	1
NUCLEOTIDE_BINDING	-0,096	0,009	0,00045	1
V$GATA1_03	-0,096	0,009	0,00061	1

chr7p12	-0,096	0,009	0,2032	1
GSE17721_CTRL_VS_CPG_4H_BMDM_DN	-0,095	0,009	0,0033	1
MODULE_444	-0,095	0,009	0,00546	1
CYTOKINE_ACTIVITY	-0,095	0,009	0,0218	1
CORRE_MULTIPLE_MYELOMA_UP	-0,095	0,009	0,00244	1
GNF2_G22P1	-0,095	0,009	0,1525	1
REACTOME_IL_7_SIGNALING	-0,095	0,009	0,00332	1
GSE13306_RA_VS_UNTREATED_TCONV_DN	-0,095	0,009	0,00054	1
ACTIN_FILAMENT_BASED_MOVEMENT	-0,095	0,009	0,04542	1
MODULE_160	-0,095	0,009	0,67028	1
AAGCACA,MIR-218	-0,095	0,009	0,00035	1
GSE9650_NAIVE_VS_EFF_CD8_TCELL_DN	-0,095	0,009	0,03903	1
KEGG_ALANINE_ASPARTATE_AND_GLUTAMA	0,095	0,009	0,0329	1
GSE27786_BCELL_VS_NEUTROPHIL_UP	-0,095	0,009	0,00301	1
REACTOME_KERATAN_SULFATE_KERATIN_ME	-0,095	0,009	0,01489	1
GSE32423_MEMORY_VS_NAIVE_CD8_TCELL_	-0,095	0,009	0,00085	1
GSE9037_WT_VS_IRAK4_KO_LPS_4H_STIM_	-0,095	0,009	0,00548	1
GTGCCTT,MIR-506	-0,095	0,009	0,00285	1
GSE14350_IL2RB_KO_VS_WT_TREG_DN	-0,095	0,009	0,00095	1
IKEDA_MIR30_TARGETS_UP	-0,095	0,009	0,00191	1
GSE3982_EFF_MEMORY_CD4_TCELL_VS_TH1	-0,095	0,009	0,00018	1
MODULE_537	-0,095	0,009	0,14169	1
GSE17721_PAM3CSK4_VS_CPG_1H_BMDM_U	-0,095	0,009	0,00043	1
NUYTTEN_EZH2_TARGETS_UP	-0,095	0,009	0,02008	1
GSE17721_POLYIC_VS_CPG_4H_BMDM_DN	-0,095	0,009	0,02072	1
KEGG_LONG_TERM_DEPRESSION	-0,095	0,009	0,00119	1
NAKAMURA_ADIPOGENESIS_LATE_DN	-0,095	0,009	0,00474	1
NAKAMURA_ADIPOGENESIS_EARLY_DN	-0,095	0,009	0,0048	1
GSE17974_IL4_AND_ANTI_IL12_VS_UNTREA	-0,095	0,009	0,02124	1
MOLECULAR_ADAPTOR_ACTIVITY	-0,095	0,009	0,00014	1
CATION_TRANSMEMBRANE_TRANSPORTER_A(	-0,095	0,009	0,00072	1
ABE_VEGFA_TARGETS_30MIN	-0,095	0,009	0,00016	1
MODULE_47	-0,095	0,009	0,00161	1
CASTELLANO_HRAS_AND_NRAS_TARGETS_D	-0,095	0,009	0,00046	1
HASLINGER_B_CLL_WITH_6Q21_DELETION	-0,095	0,009	0,12204	1
INTEGRAL_TO_ENDOPLASMIC_RETICULUM_ME	0,095	0,009	0,03957	1
INTRINSIC_TO_ENDOPLASMIC_RETICULUM_ME	0,095	0,009	0,03957	1
ZEMBUTSU_SENSITIVITY_TO_METHOTREXATE	-0,095	0,009	0,00163	1
HYDROLASE_ACTIVITY_ACTING_ON_CARBON_	-0,095	0,01	0,00025	1
KLEIN_TARGETS_OF_BCR_ABL1_FUSION	-0,095	0,01	0,00823	1
GSE39820_CTRL_VS_IL1B_IL6_IL23A_CD4_	-0,095	0,01	0,00428	1
POSITIVE_REGULATION_OF_IMMUNE_SYSTEM_	-0,095	0,01	0,01182	1

BIOCARTA_CDMAC_PATHWAY	-0,095	0,01	0,0039	1
ZHOU_INFLAMMATORY_RESPONSE_FIMA_UP	-0,095	0,01	0,00178	1
GSE360_CTRL_VS_L_MAJOR_DC_DN	-0,095	0,01	0,00437	1
GTPASE_ACTIVATOR_ACTIVITY	-0,095	0,01	0,00032	1
ZHONG_SECRETOME_OF_LUNG_CANCER_AND	-0,095	0,01	0,13476	1
CLASPER_LYMPHATIC_VESSELS_DURING_MET	-0,095	0,01	0,00396	1
MODULE_102	-0,095	0,01	0,16044	1
REACTOME_CS_DS_DEGRADATION	-0,095	0,01	0,00471	1
KORKOLA_EMBRYONIC_CARCINOMA_VS_SEMI	-0,095	0,01	0,01168	1
ST_STAT3_PATHWAY	-0,095	0,01	0,00735	1
TERAMOTO_OPN_TARGETS_CLUSTER_3	-0,095	0,01	0,04361	1
BROWNE_HCMV_INFECTION_16HR_DN	-0,095	0,01	0,00016	1
MODULE_65	-0,095	0,01	0,00431	1
CRX_NRL_DN.V1_DN	-0,095	0,01	0,00027	1
MODULE_456	-0,095	0,01	0,00341	1
S_ADENOSYLMETHIONINE_DEPENDENT_METHY	-0,095	0,01	0,03371	1
GOLGI_VESICLE_TRANSPORT	0,095	0,01	0,06575	1
SHAFFER_IRF4_TARGETS_IN_PLASMA_CELL_\	-0,094	0,01	0,07255	1
DAIRKEE_CANCER_PRONE_RESPONSE_BPA	-0,094	0,01	0,02089	1
GCM_DENR	-0,094	0,01	0,02908	1
ELVIDGE_HIF1A_AND_HIF2A_TARGETS_UP	-0,094	0,01	0,40604	1
LEE_METASTASIS_AND_ALTERNATIVE_SPLICI	-0,094	0,01	0,00744	1
TCCAGAT,MIR-516-5P	-0,094	0,01	0,00104	1
ENDOPEPTIDASE_ACTIVITY	-0,094	0,01	0,006	1
GSE15930_NAIVE_VS_72H_IN_VITRO_STIM_	-0,094	0,01	0,00017	1
GSE32423_MEMORY_VS_NAIVE_CD8_TCELL_	-0,094	0,01	0,00329	1
REACTOME_BRANCHED_CHAIN_AMINO_ACID_	0,094	0,01	0,08763	1
GSE20715_0H_VS_6H_OZONE_LUNG_UP	-0,094	0,01	0,00548	1
chr5q33	-0,094	0,01	0,00645	1
VESICLE_COAT	-0,094	0,01	0,1319	1
GSE17974_IL4_AND_ANTI_IL12_VS_UNTREA	-0,094	0,01	0,00038	1
ELVIDGE_HYPOXIA_UP	-0,094	0,01	0,04863	1
GSE13306_TREG_VS_TCONV_SPLEEN_DN	-0,094	0,01	0,00353	1
ZHONG_SECRETOME_OF_LUNG_CANCER_AND	-0,094	0,01	0,23889	1
REACTOME_PRE_NOTCH_TRANSCRIPTION_AN	-0,094	0,01	0,00392	1
SAGIV_CD24_TARGETS_UP	-0,094	0,01	0,00832	1
AIGNER_ZEB1_TARGETS	-0,094	0,01	0,26856	1
SENESE_HDAC2_TARGETS_UP	-0,094	0,01	0,075	1
MORF_FOSL1	-0,094	0,01	0,00062	1
SPIRA_SMOKERS_LUNG_CANCER_DN	-0,094	0,01	0,05375	1
VERRECCHIA_RESPONSE_TO_TGFB1_C2	-0,094	0,01	0,00289	1
PROTEIN_AMINO_ACID_DEPHOSPHORYLATION	-0,094	0,01	0,00033	1

ACCATTT,MIR-522	-0,094	0,01	0,0061	1
chr9p12	-0,094	0,01	0,21343	1
CELLULAR_RESPONSE_TO_NUTRIENT_LEVELS	-0,094	0,01	0,02988	1
GSE13738_RESTING_VS_TCR_ACTIVATED_CI	-0,094	0,01	0,11404	1
MAP_KINASE_ACTIVITY	-0,094	0,01	0,05097	1
MORF_HAT1	-0,094	0,01	0,32205	1
DER_IFN_ALPHA_RESPONSE_DN	-0,094	0,01	0,02785	1
BOYLAN_MULTIPLE_MYELOMA_D_CLUSTER_D	-0,094	0,01	0,00569	1
LINDSTEDT_DENDRITIC_CELL_MATURATION_(	-0,094	0,01	0,00029	1
LIM_MAMMARY_STEM_CELL_DN	0,094	0,01	0,21426	1
MODULE_352	-0,094	0,01	0,15305	1
GSE1460_DP_THYMOCYTE_VS_THYMIC_STRO	-0,094	0,01	0,00055	1
GSE27786_CD8_TCELL_VS_ERYTHROBLAST_	-0,094	0,01	0,00724	1
V$OCT1_03	-0,094	0,01	0,00031	1
SA_PTEN_PATHWAY	-0,094	0,01	0,02378	1
GSE17974_CTRL_VS_ACT_IL4_AND_ANTI_IL	-0,094	0,01	0,00722	1
XU_HGF_TARGETS_INDUCED_BY_AKT1_48HR	-0,094	0,01	0,00751	1
CELLULAR_RESPIRATION	-0,094	0,01	0,05145	1
PID_INSULIN_PATHWAY	-0,094	0,01	0,00139	1
NEGATIVE_REGULATION_OF_APOPTOSIS	-0,094	0,01	0,01765	1
GCM_VAV1	-0,094	0,01	0,00374	1
ZHOU_PANCREATIC_BETA_CELL	0,094	0,01	0,12099	1
KIM_GERMINAL_CENTER_T_HELPER_UP	-0,094	0,01	0,0127	1
CHANG_IMMORTALIZED_BY_HPV31_DN	-0,094	0,01	0,14305	1
REACTOME_DESTABILIZATION_OF_MRNA_BY_	-0,094	0,01	0,08905	1
ACETYLGLUCOSAMINYLTRANSFERASE_ACTIVI	-0,094	0,01	0,07243	1
TAVAZOIE_METASTASIS	-0,094	0,01	0,00193	1
VANTVEER_BREAST_CANCER_ESR1_UP	0,094	0,01	0,40279	1
PROTEOGLYCAN_METABOLIC_PROCESS	-0,094	0,01	0,00315	1
V$USF_Q6_01	-0,094	0,01	0,00062	1
MODULE_35	-0,094	0,01	0,47697	1
REACTOME_MRNA_DECAY_BY_5_TO_3_EXOR	-0,094	0,01	0,08042	1
REACTOME_RNA_POL_III_TRANSCRIPTION_INI	-0,094	0,01	0,09994	1
HOWLIN_PUBERTAL_MAMMARY_GLAND	-0,094	0,01	0,00164	1
BROWNE_HCMV_INFECTION_6HR_DN	-0,094	0,01	0,00242	1
HENDRICKS_SMARCA4_TARGETS_UP	-0,094	0,01	0,10935	1
RYTTCCTG_V$ETS2_B	-0,094	0,011	0,00154	1
DANG_REGULATED_BY_MYC_DN	-0,094	0,011	0,00453	1
GSE27786_BCELL_VS_CD4_TCELL_DN	-0,094	0,011	0,00619	1
GSE8515_CTRL_VS_IL6_4H_STIM_MAC_UP	-0,094	0,011	0,00281	1
PID_IL2_1PATHWAY	-0,094	0,011	0,00224	1
AMINO_ACID_TRANSMEMBRANE_TRANSPORTE	-0,094	0,011	0,00935	1

GSE20366_EX_VIVO_VS_DEC205_CONVERSI(	-0,094	0,011	0,00358	1
chr16p12	0,094	0,011	0,25496	1
DURAND_STROMA_MAX_UP	-0,094	0,011	0,00068	1
GSE20366_TREG_VS_NAIVE_CD4_TCELL_UP	-0,094	0,011	0,01401	1
GSE15659_CD45RA_NEG_CD4_TCELL_VS_A(	-0,094	0,011	0,00086	1
ACEVEDO_LIVER_CANCER_WITH_H3K9ME3_[	-0,094	0,011	0,04642	1
GSE360_L_DONOVANI_VS_M_TUBERCULOSIS	-0,094	0,011	0,00539	1
GSE360_L_MAJOR_VS_B_MALAYI_HIGH_DOS	-0,093	0,011	0,00731	1
NATSUME_RESPONSE_TO_INTERFERON_BETA	-0,093	0,011	0,04068	1
chr1p33	0,093	0,011	0,24026	1
HEMOPOIETIC_OR_LYMPHOID_ORGAN_DEVEL(	-0,093	0,011	0,00744	1
RAMJAUN_APOPTOSIS_BY_TGFB1_VIA_MAPK	0,093	0,011	0,30257	1
MORF_PDCD1	-0,093	0,011	0,00311	1
SESTO_RESPONSE_TO_UV_C1	-0,093	0,011	0,31696	1
ALONSO_METASTASIS_UP	-0,093	0,011	0,0296	1
AKT_UP.V1_UP	-0,093	0,011	0,06541	1
chr20q13	-0,093	0,011	0,00667	1
V$ETS_Q4	-0,093	0,011	0,00193	1
PID_INTEGRIN_A9B1_PATHWAY	-0,093	0,011	0,04906	1
TTGGAGA,MIR-515-5P,MIR-519E	-0,093	0,011	0,00343	1
IGLESIAS_E2F_TARGETS_UP	-0,093	0,011	0,00066	1
REGULATION_OF_PROTEIN_AMINO_ACID_PHO	-0,093	0,011	0,00674	1
VANTVEER_BREAST_CANCER_METASTASIS_L	0,093	0,011	0,54461	1
GSE20366_TREG_VS_NAIVE_CD4_TCELL_DE	-0,093	0,011	0,00122	1
T_CELL_PROLIFERATION	-0,093	0,011	0,03036	1
NEUROPEPTIDE_SIGNALING_PATHWAY	-0,093	0,011	0,0086	1
YAGI_AML_FAB_MARKERS	-0,093	0,011	0,01419	1
GSE13411_IGM_MEMORY_BCELL_VS_PLASMA	-0,093	0,011	0,0008	1
ZHANG_RESPONSE_TO_IKK_INHIBITOR_AND_	-0,093	0,011	0,08019	1
RAS_GTPASE_ACTIVATOR_ACTIVITY	-0,093	0,011	0,00796	1
ZHAN_MULTIPLE_MYELOMA_HP_DN	-0,093	0,011	0,04327	1
CCCNNNNNNAAGWT_UNKNOWN	-0,093	0,011	0,00022	1
GSE17721_CTRL_VS_GARDIQUIMOD_12H_BM	-0,093	0,011	0,01343	1
V$NRF1_Q6	-0,093	0,011	0,00019	1
GCM_MAX	-0,093	0,011	0,03982	1
GSE3982_BCELL_VS_TH1_UP	-0,093	0,011	0,00038	1
BROWNE_HCMV_INFECTION_10HR_DN	-0,093	0,011	0,01032	1
MODULE_78	0,093	0,011	0,27829	1
GSE24142_EARLY_THYMIC_PROGENITOR_VS_	-0,093	0,011	0,00565	1
chr15q22	-0,093	0,011	0,00021	1
GCTCTTG,MIR-335	-0,093	0,011	0,00138	1
ACTIN_FILAMENT_BASED_PROCESS	-0,093	0,011	0,00016	1

LIN_TUMOR_ESCAPE_FROM_IMMUNE_ATTACK	-0,093	0,011	0,06014	1
VERRECCHIA_EARLY_RESPONSE_TO_TGFB1	-0,093	0,011	0,00102	1
ATCMNTCCGY_UNKNOWN	-0,093	0,011	0,00133	1
TCTAGAG,MIR-517	-0,093	0,011	0,01281	1
REACTOME_METABOLISM_OF_PORPHYRINS	0,093	0,011	0,02253	1
GSE10325_CD4_TCELL_VS_LUPUS_CD4_TCE	-0,093	0,011	0,06167	1
GSE9650_NAIVE_VS_MEMORY_CD8_TCELL_U	-0,093	0,011	0,00486	1
LINDGREN_BLADDER_CANCER_CLUSTER_2B	-0,093	0,011	0,00154	1
GTGACTT,MIR-224	-0,093	0,011	0,00228	1
MORF_PPP2CA	-0,093	0,011	0,29097	1
TAGHAVI_NEOPLASTIC_TRANSFORMATION	-0,093	0,011	0,00105	1
GSE360_LOW_DOSE_B_MALAYI_VS_M_TUBE	-0,093	0,011	0,01735	1
POSITIVE_REGULATION_OF_LYMPHOCYTE_AC	-0,093	0,011	0,01688	1
UDP_GLYCOSYLTRANSFERASE_ACTIVITY	-0,093	0,011	0,02468	1
MORF_BNIP1	-0,093	0,011	0,00145	1
DARWICHE_PAPILLOMA_RISK_LOW_DN	-0,093	0,011	0,00177	1
SIG_PIP3_SIGNALING_IN_B_LYMPHOCYTES	-0,093	0,012	0,01036	1
GSE7460_TREG_VS_TCONV_ACT_WITH_TGFI	-0,092	0,012	0,00032	1
MORF_SART1	-0,092	0,012	0,07078	1
GSE13485_DAY3_VS_DAY21_YF17D_VACCII	-0,092	0,012	0,02486	1
ZHU_CMV_24_HR_DN	-0,092	0,012	0,00034	1
GHANDHI_DIRECT_IRRADIATION_DN	-0,092	0,012	0,00121	1
TGCTTTG,MIR-330	-0,092	0,012	0,00257	1
WAKASUGI_HAVE_ZNF143_BINDING_SITES	-0,092	0,012	0,02256	1
SENESE_HDAC3_TARGETS_UP	-0,092	0,012	0,04513	1
GSE22886_IGM_MEMORY_BCELL_VS_BLOOD_	-0,092	0,012	0,00173	1
GCM_USP6	-0,092	0,012	0,0004	1
REACTOME_EXTENSION_OF_TELOMERES	-0,092	0,012	0,03665	1
REACTOME_PECAM1_INTERACTIONS	-0,092	0,012	0,03851	1
GSE10239_MEMORY_VS_DAY4.5_EFF_CD8_T	-0,092	0,012	0,22024	1
HOFMANN_CELL_LYMPHOMA_UP	-0,092	0,012	0,06012	1
SERVITJA_LIVER_HNF1A_TARGETS_DN	-0,092	0,012	0,00018	1
GCATTTG,MIR-105	-0,092	0,012	0,00837	1
GSE2706_UNSTIM_VS_2H_LPS_AND_R848_D	-0,092	0,012	0,00053	1
BIOCARTA_BCR_PATHWAY	-0,092	0,012	0,00459	1
GSE9988_LPS_VS_VEHICLE_TREATED_MONO	-0,092	0,012	0,05158	1
LINDSTEDT_DENDRITIC_CELL_MATURATION_E	-0,092	0,012	0,0322	1
XU_CREBBP_TARGETS_UP	-0,092	0,012	0,05008	1
TOMIDA_METASTASIS_DN	-0,092	0,012	0,11784	1
NEGATIVE_REGULATION_OF_CATALYTIC_ACT	-0,092	0,012	0,00193	1
NIKOLSKY_BREAST_CANCER_21Q22_AMPLICO	-0,092	0,012	0,05074	1
CHEMELLO_SOLEUS_VS_EDL_MYOFIBERS_DN	-0,092	0,012	0,00021	1

REACTOME_CYCLIN_E_ASSOCIATED_EVENTS_	-0,092	0,012	0,24467	1
ACEVEDO_LIVER_CANCER_WITH_H3K9ME3_U	-0,092	0,012	0,00046	1
GSE13411_NAIVE_BCELL_VS_PLASMA_CELL_	-0,092	0,012	0,00182	1
GSE17721_0.5H_VS_12H_CPG_BMDM_UP	-0,092	0,012	0,01176	1
TRNA_METABOLIC_PROCESS	-0,092	0,012	0,71242	1
KRIGE_AMINO_ACID_DEPRIVATION	-0,092	0,012	0,25991	1
DAVICIONI_MOLECULAR_ARMS_VS_ERMS_DN	-0,092	0,012	0,00042	1
GSE2197_CPG_DNA_VS_UNTREATED_IN_DC_	-0,092	0,012	0,00945	1
MIKKELSEN_DEDIFFERENTIATED_STATE_DN	-0,092	0,012	0,00072	1
CROONQUIST_STROMAL_STIMULATION_DN	-0,092	0,012	0,05433	1
GSE3337_CTRL_VS_4H_IFNG_IN_CD8POS_D(	-0,092	0,012	0,00263	1
REACTOME_POST_TRANSLATIONAL_PROTEIN_	-0,092	0,012	0,0404	1
ROZANOV_MMP14_TARGETS_UP	-0,092	0,012	0,002	1
SMALL_CONJUGATING_PROTEIN_BINDING	-0,092	0,012	0,03715	1
WANG_MLL_TARGETS	-0,092	0,012	0,00033	1
KAECH_DAY8_EFF_VS_DAY15_EFF_CD8_TCE	-0,092	0,012	0,0003	1
GSE17721_CTRL_VS_POLYIC_4H_BMDM_DN	-0,092	0,012	0,00197	1
GSE17721_0.5H_VS_24H_LPS_BMDM_UP	-0,092	0,012	0,00193	1
YANG_BREAST_CANCER_ESR1_UP	0,092	0,012	0,42708	1
GSE2706_R848_VS_R848_AND_LPS_2H_STI	-0,092	0,012	0,06187	1
REACTOME_APC_C_CDH1_MEDIATED_DEGRAI	-0,092	0,012	0,32379	1
REACTOME_SIGNALING_BY_FGFR1_MUTANTS	-0,092	0,012	0,01631	1
GSE18791_UNSTIM_VS_NEWCATSLE_VIRUS_	-0,092	0,012	0,01489	1
CHEMOKINE_ACTIVITY	-0,092	0,012	0,05785	1
MORF_HDAC1	-0,092	0,012	0,23132	1
GSE17721_CTRL_VS_GARDIQUIMOD_2H_BMD	-0,092	0,012	0,01325	1
REPRODUCTIVE_PROCESS	-0,092	0,012	0,01513	1
GSE12366_NAIVE_VS_MEMORY_BCELL_UP	-0,092	0,012	0,00073	1
GSE9037_CTRL_VS_LPS_1H_STIM_BMDM_DN	-0,092	0,012	0,00154	1
PID_TXA2PATHWAY	-0,092	0,012	0,00875	1
MACLACHLAN_BRCA1_TARGETS_UP	-0,092	0,012	0,02952	1
GSE17721_0.5H_VS_12H_CPG_BMDM_DN	-0,092	0,012	0,01648	1
ZHANG_GATA6_TARGETS_UP	-0,092	0,012	0,00349	1
GENTILE_UV_HIGH_DOSE_UP	-0,092	0,012	0,01605	1
MODULE_488	-0,092	0,012	0,10867	1
REACTOME_SYNTHESIS_OF_PE	-0,092	0,012	0,00651	1
REACTOME_GAB1_SIGNALOSOME	-0,092	0,012	0,00075	1
BERENJENO_ROCK_SIGNALING_NOT_VIA_RHC	-0,092	0,012	0,02733	1
GSE17721_CTRL_VS_LPS_12H_BMDM_UP	-0,092	0,012	0,00165	1
MATZUK_LUTEAL_GENES	-0,092	0,012	0,00023	1
REACTOME_DOWNSTREAM_SIGNALING_EVENT	-0,092	0,012	0,13435	1
TRANSITION_METAL_ION_BINDING	-0,092	0,012	0,00886	1

STRUCTURAL_CONSTITUENT_OF_RIBOSOME	-0,092	0,012	0,00651	1
FOSTER_TOLERANT_MACROPHAGE_DN	-0,092	0,012	0,0187	1
NOJIMA_SFRP2_TARGETS_DN	-0,091	0,012	0,06987	1
MODULE_145	-0,091	0,012	0,00333	1
ROME_INSULIN_TARGETS_IN_MUSCLE_UP	-0,091	0,012	0,08053	1
ACEVEDO_METHYLATED_IN_LIVER_CANCER_I	-0,091	0,013	0,00025	1
REACTOME_GLUCOSE_TRANSPORT	-0,091	0,013	0,10561	1
MATZUK_SPERMATID_DIFFERENTIATION	-0,091	0,013	0,01527	1
V$CDP_01	-0,091	0,013	0,00017	1
WU_ALZHEIMER_DISEASE_DN	-0,091	0,013	0,00026	1
ENZYME_INHIBITOR_ACTIVITY	-0,091	0,013	0,00119	1
ST_DIFFERENTIATION_PATHWAY_IN_PC12_CI	-0,091	0,013	0,00041	1
GSE17721_POLYIC_VS_PAM3CSK4_8H_BMDI	-0,091	0,013	0,08001	1
NEUROTRANSMITTER_BINDING	-0,091	0,013	0,00911	1
chr2q35	-0,091	0,013	0,0359	1
GSE15930_NAIVE_VS_72H_IN_VITRO_STIM_	-0,091	0,013	0,00015	1
GEISS_RESPONSE_TO_DSRNA_UP	-0,091	0,013	0,02357	1
chr8q21	-0,091	0,013	0,0262	1
MILI_PSEUDOPODIA_HAPTOTAXIS_DN	-0,091	0,013	0,02787	1
PID_WNT_NONCANONICAL_PATHWAY	-0,091	0,013	0,01788	1
RTTTNNNYTGGM_UNKNOWN	-0,091	0,013	0,00365	1
GGGGCCC,MIR-296	-0,091	0,013	0,00331	1
MTOR_UP.N4.V1_UP	-0,091	0,013	0,2724	1
UZONYI_RESPONSE_TO_LEUKOTRIENE_AND_T	-0,091	0,013	0,00066	1
REACTOME_SHC1_EVENTS_IN_EGFR_SIGNALI	-0,091	0,013	0,16565	1
HAN_SATB1_TARGETS_DN	-0,091	0,013	0,03236	1
CATION_BINDING	-0,091	0,013	0,00472	1
GSE15750_WT_VS_TRAF6KO_DAY6_EFF_CD	-0,091	0,013	0,00028	1
SULFOTRANSFERASE_ACTIVITY	-0,091	0,013	0,02414	1
ZEMBUTSU_SENSITIVITY_TO_VINCRISTINE	-0,091	0,013	0,0916	1
GSE11924_TH1_VS_TH17_CD4_TCELL_DN	-0,091	0,013	0,00122	1
INTERLEUKIN_2_PRODUCTION	-0,091	0,013	0,03427	1
GSE2706_2H_VS_8H_R848_STIM_DC_DN	-0,091	0,013	0,0689	1
CAFFAREL_RESPONSE_TO_THC_24HR_5_UP	-0,091	0,013	0,38121	1
PEPTIDASE_ACTIVITY	-0,091	0,013	0,00803	1
PID_PI3KCIPATHWAY	-0,091	0,013	0,01335	1
ZHANG_BREAST_CANCER_PROGENITORS_DN	-0,091	0,013	0,00175	1
HOMEOSTATIC_PROCESS	-0,091	0,013	0,00352	1
GSE3982_DC_VS_NEUTROPHIL_UP	-0,091	0,013	0,07167	1
GSE14308_TH1_VS_NATURAL_TREG_UP	-0,091	0,013	0,00063	1
BIOCARTA_IL2_PATHWAY	-0,091	0,013	0,0129	1
V$IK2_01	-0,091	0,013	0,00054	1

MODULE_115	-0,091	0,013	0,03754	1
ZHANG_TARGETS_OF_EWSR1_FLI1_FUSION	-0,091	0,013	0,00029	1
HASINA_NOL7_TARGETS_UP	-0,091	0,013	0,04298	1
PHOSPHOLIPASE_ACTIVITY	-0,091	0,013	0,0175	1
TGTGTGA,MIR-377	-0,091	0,013	0,00071	1
CHEMOKINE_RECEPTOR_BINDING	-0,091	0,013	0,0585	1
REACTOME_CLASS_I_MHC_MEDIATED_ANTIGE	-0,091	0,013	0,1834	1
TAKEDA_TARGETS_OF_NUP98_HOXA9_FUSIC	-0,091	0,013	0,00566	1
ZHU_CMV_ALL_DN	-0,091	0,013	0,00032	1
ZWANG_TRANSIENTLY_UP_BY_1ST_EGF_PUL	-0,091	0,013	0,00044	1
SA_G1_AND_S_PHASES	-0,091	0,013	0,02059	1
PEREZ_TP53_TARGETS	-0,091	0,013	0,00013	1
GSE17580_UNINFECTED_VS_S_MANSONI_INF	-0,091	0,013	0,00992	1
LY_AGING_OLD_UP	-0,091	0,013	0,02777	1
MORF_AP3D1	-0,091	0,013	0,52684	1
WANG_TUMOR_INVASIVENESS_DN	-0,091	0,013	0,04844	1
MODULE_27	-0,091	0,013	0,0028	1
BIOCARTA_TGFB_PATHWAY	-0,091	0,013	0,00816	1
KEGG_ARRHYTHMOGENIC_RIGHT_VENTRICULA	-0,091	0,013	0,00023	1
ROSS_AML_WITH_AML1_ETO_FUSION	-0,091	0,013	0,00157	1
REACTOME_CLASS_B_2_SECRETIN_FAMILY_R	-0,091	0,013	0,00329	1
DAVICIONI_PAX_FOXO1_SIGNATURE_IN_ARMS	-0,091	0,013	0,0007	1
BIOCARTA_IL7_PATHWAY	-0,091	0,013	0,00651	1
WATANABE_COLON_CANCER_MSI_VS_MSS_U	0,091	0,013	0,3152	1
LIEN_BREAST_CARCINOMA_METAPLASTIC	-0,091	0,013	0,00066	1
GSE17721_CTRL_VS_GARDIQUIMOD_0.5H_BM	-0,091	0,013	0,01197	1
BURTON_ADIPOGENESIS_8	-0,091	0,013	0,00715	1
GSE9988_ANTI_TREM1_VS_ANTI_TREM1_AN	-0,091	0,013	0,06908	1
KINETOCHORE	-0,091	0,013	0,08346	1
RESPONSE_TO_HYPOXIA	-0,09	0,013	0,0789	1
GSE27786_LIN_NEG_VS_CD8_TCELL_DN	-0,09	0,014	0,00188	1
chrxq22	-0,09	0,014	0,00197	1
GSE17721_LPS_VS_POLYIC_2H_BMDM_UP	-0,09	0,014	0,00886	1
GSE3982_CTRL_VS_IGE_STIM_MAST_CELL_U	-0,09	0,014	0,00277	1
GSE17721_0.5H_VS_8H_POLYIC_BMDM_UP	-0,09	0,014	0,02552	1
GSE22886_NEUTROPHIL_VS_MONOCYTE_DN	-0,09	0,014	0,14595	1
MAINTENANCE_OF_LOCALIZATION	-0,09	0,014	0,0852	1
SASSON_FSH_RESPONSE	-0,09	0,014	0,00051	1
NIELSEN_LEIOMYOSARCOMA_UP	-0,09	0,014	0,05908	1
REACTOME_RAF_MAP_KINASE_CASCADE	-0,09	0,014	0,08236	1
MORF_CASP2	-0,09	0,014	0,00043	1
GSE339_EX_VIVO_VS_IN_CULTURE_CD4POS_	-0,09	0,014	0,00662	1

TRANSMEMBRANE_TRANSPORTER_ACTIVITY	-0,09	0,014	0,00032	1
MORF_FANCG	-0,09	0,014	0,00188	1
REGULATION_OF_MUSCLE_CONTRACTION	-0,09	0,014	0,00557	1
ACTTTAT,MIR-142-5P	-0,09	0,014	0,00019	1
HUANG_FOXA2_TARGETS_DN	-0,09	0,014	0,05502	1
MORF_EIF3S6	-0,09	0,014	0,06036	1
FONTAINE_PAPILLARY_THYROID_CARCINOMA_	-0,09	0,014	0,05983	1
REACTOME_DNA_REPAIR	-0,09	0,014	0,02494	1
GOLDRATH_HOMEOSTATIC_PROLIFERATION	-0,09	0,014	0,06455	1
GSE17721_CTRL_VS_POLYIC_4H_BMDM_UP	-0,09	0,014	0,02585	1
GSE17721_POLYIC_VS_CPG_12H_BMDM_UP	-0,09	0,014	0,00443	1
CREIGHTON_ENDOCRINE_THERAPY_RESISTAN(	0,09	0,014	0,08539	1
POSITIVE_REGULATION_OF_T_CELL_ACTIVAT	-0,09	0,014	0,02167	1
GAVIN_IL2_RESPONSIVE_FOXP3_TARGETS_DI	-0,09	0,014	0,12637	1
GSE30962_ACUTE_VS_CHRONIC_LCMV_SEC(	-0,09	0,014	0,00022	1
GSE12366_PLASMA_CELL_VS_MEMORY_BCEI	-0,09	0,014	0,01299	1
CYTOPLASMIC_PART	-0,09	0,014	0,00128	1
METHYLTRANSFERASE_ACTIVITY	-0,09	0,014	0,04346	1
PROTEIN_SERINE_THREONINE_TYROSINE_KIN/	-0,09	0,014	0,29076	1
BONE_REMODELING	-0,09	0,014	0,01128	1
HEIDENBLAD_AMPLIFIED_IN_BONE_CANCER	-0,09	0,014	0,02549	1
GSE29618_BCELL_VS_PDC_DN	-0,09	0,014	0,01816	1
TSUTSUMI_FBXW8_TARGETS	-0,09	0,014	0,06228	1
MODULE_223	-0,09	0,014	0,0066	1
DOANE_BREAST_CANCER_ESR1_UP	0,09	0,014	0,34894	1
XU_HGF_TARGETS_INDUCED_BY_AKT1_6HR	-0,09	0,014	0,0235	1
chr16q21	-0,09	0,014	0,25619	1
REACTOME_APC_C_CDC20_MEDIATED_DEGR/	-0,09	0,014	0,29287	1
GSE17721_LPS_VS_GARDIQUIMOD_1H_BMDM	-0,09	0,014	0,00289	1
BIOCARTA_CREB_PATHWAY	-0,09	0,014	0,00466	1
MODULE_156	-0,09	0,014	0,00044	1
CAMP_UP.V1_UP	-0,09	0,014	0,03114	1
GSE2706_UNSTIM_VS_8H_LPS_AND_R848_[	-0,09	0,014	0,06244	1
YAGI_AML_RELAPSE_PROGNOSIS	-0,09	0,014	0,07915	1
GSE17974_0H_VS_2H_IN_VITRO_ACT_CD4_	-0,09	0,014	0,02583	1
GSE3337_CTRL_VS_16H_IFNG_IN_CD8POS_[	-0,09	0,014	0,0328	1
GSE18791_CTRL_VS_NEWCASTLE_VIRUS_DC	-0,09	0,014	0,0241	1
SCHAEFFER_PROSTATE_DEVELOPMENT_48HR	-0,09	0,014	0,00053	1
SUBSTRATE_SPECIFIC_TRANSPORTER_ACTIVI	-0,09	0,014	0,00055	1
MODULE_63	-0,09	0,014	0,00136	1
REACTOME_ASSEMBLY_OF_THE_PRE_REPLIC/	-0,09	0,014	0,41116	1
TTTGCAG,MIR-518A-2	-0,09	0,014	0,01069	1

GSE22886_NAIVE_TCELL_VS_MONOCYTE_UF	-0,09	0,014	0,00073	1
chr17q25	-0,09	0,014	0,01708	1
BIOCARTA_MET_PATHWAY	-0,09	0,014	0,0072	1
GSE30083_SP2_VS_SP3_THYMOCYTE_UP	-0,09	0,014	0,0011	1
HU_GENOTOXIC_DAMAGE_24HR	-0,09	0,014	0,28103	1
GSE22886_NAIVE_CD4_TCELL_VS_MONOCY	-0,09	0,014	0,00085	1
MODULE_491	-0,09	0,014	0,02894	1
RRNA_PROCESSING	-0,09	0,015	0,08206	1
QI_HYPOXIA	-0,089	0,015	0,0642	1
V$FOX_Q2	-0,089	0,015	0,00022	1
GSE19825_NAIVE_VS_DAY3_EFF_CD8_TCEL	-0,089	0,015	0,08874	1
GSE11057_CD4_CENT_MEM_VS_PBMC_UP	-0,089	0,015	0,0009	1
PID_AMB2_NEUTROPHILS_PATHWAY	-0,089	0,015	0,0058	1
LIANG_SILENCED_BY_METHYLATION_2	-0,089	0,015	0,23174	1
GSE24634_NAIVE_CD4_TCELL_VS_DAY5_IL	-0,089	0,015	0,37779	1
chr6q14	-0,089	0,015	0,01473	1
GSE17721_POLYIC_VS_PAM3CSK4_2H_BMDI	-0,089	0,015	0,00383	1
SHEPARD_BMYB_MORPHOLINO_UP	-0,089	0,015	0,0061	1
HIRSCH_CELLULAR_TRANSFORMATION_SIGNA	-0,089	0,015	0,07876	1
GSE13485_CTRL_VS_DAY21_YF17D_VACCII	-0,089	0,015	0,00016	1
GSE9037_CTRL_VS_LPS_4H_STIM_IRAK4_K(	-0,089	0,015	0,03963	1
KEGG_JAK_STAT_SIGNALING_PATHWAY	-0,089	0,015	0,00369	1
IMMUNE_SYSTEM_DEVELOPMENT	-0,089	0,015	0,00945	1
YAMASHITA_LIVER_CANCER_WITH_EPCAM_D	0,089	0,015	0,13784	1
CYCLIC_NUCLEOTIDE_PHOSPHODIESTERASE_/	-0,089	0,015	0,00144	1
ZHAN_MULTIPLE_MYELOMA_CD1_UP	-0,089	0,015	0,003	1
GRANDVAUX_IRF3_TARGETS_DN	-0,089	0,015	0,00444	1
KORKOLA_EMBRYONAL_CARCINOMA_UP	-0,089	0,015	0,10786	1
GSE29618_LAIV_VS_TIV_FLU_VACCINE_DAY	-0,089	0,015	0,00612	1
YTCCCRNNAGGY_UNKNOWN	-0,089	0,015	0,00193	1
MORF_RAF1	-0,089	0,015	0,12426	1
LINDVALL_IMMORTALIZED_BY_TERT_UP	-0,089	0,015	0,00123	1
MODULE_524	-0,089	0,015	0,00084	1
MISHRA_CARCINOMA_ASSOCIATED_FIBROBLA	-0,089	0,015	0,00258	1
OLIGOSACCHARYL_TRANSFERASE_COMPLEX	-0,089	0,015	0,50275	1
NIKOLSKY_BREAST_CANCER_8Q12_Q22_AMI	-0,089	0,015	0,05887	1
DARWICHE_PAPILLOMA_PROGRESSION_RISK	-0,089	0,015	0,00221	1
GSE9650_GP33_VS_GP276_LCMV_SPECIFIC_	-0,089	0,015	0,00052	1
HOQUE_METHYLATED_IN_CANCER	-0,089	0,015	0,00103	1
GSE30083_SP1_VS_SP3_THYMOCYTE_DN	-0,089	0,015	0,00048	1
MODULE_38	-0,089	0,015	0,0034	1
KYNG_DNA_DAMAGE_UP	-0,089	0,015	0,00141	1

REACTOME_ANTIVIRAL_MECHANISM_BY_IFN_	-0,089	0,015	0,11201	1
ZWANG_EGF_PERSISTENTLY_UP	-0,089	0,015	0,19042	1
ZHOU_PANCREATIC_ENDOCRINE_PROGENITOR	0,089	0,015	0,06505	1
ONO_FOXP3_TARGETS_UP	-0,089	0,015	0,0086	1
GLYCOSAMINOGLYCAN_BINDING	-0,089	0,015	0,00238	1
GSE24142_EARLY_THYMIC_PROGENITOR_VS_	-0,089	0,015	0,00037	1
DITTMER_PTHLH_TARGETS_DN	-0,089	0,015	0,18745	1
VERRECCHIA_RESPONSE_TO_TGFB1_C5	-0,089	0,015	0,04016	1
MODULE_568	-0,089	0,015	0,0422	1
PROTEIN_IMPORT	-0,089	0,015	0,1763	1
GSE9037_CTRL_VS_LPS_4H_STIM_BMDM_DN	-0,089	0,015	0,04971	1
MARTENS_TRETINOIN_RESPONSE_DN	-0,089	0,015	0,0127	1
ZWANG_EGF_INTERVAL_DN	-0,089	0,015	0,00016	1
KORKOLA_EMBRYONAL_CARCINOMA	-0,089	0,015	0,13385	1
WIERENGA_STAT5A_TARGETS_UP	-0,089	0,015	0,02024	1
JOHNSTONE_PARVB_TARGETS_1_UP	-0,089	0,015	0,00118	1
GSE31082_DP_VS_CD4_SP_THYMOCYTE_DN	-0,089	0,015	0,00238	1
CONTRACTILE_FIBER_PART	-0,089	0,015	0,00221	1
KEGG_VEGF_SIGNALING_PATHWAY	-0,089	0,015	0,00421	1
RODRIGUES_NTN1_TARGETS_DN	-0,089	0,015	0,00399	1
REACTOME_TRANSPORT_OF_RIBONUCLEOPRC	-0,089	0,015	0,14851	1
GROSS_HYPOXIA_VIA_HIF1A_DN	-0,089	0,015	0,00883	1
TISSUE_REMODELING	-0,089	0,015	0,01171	1
3_5_CYCLIC_NUCLEOTIDE_PHOSPHODIESTER/	-0,089	0,016	0,00126	1
MODULE_29	-0,089	0,016	0,01002	1
CELLULAR_MONOVALENT_INORGANIC_CATIOI	0,089	0,016	0,80612	1
GSE22886_NAIVE_BCELL_VS_BM_PLASMA_(	-0,089	0,016	0,0024	1
BIOCARTA_IL12_PATHWAY	-0,089	0,016	0,0251	1
HATADA_METHYLATED_IN_LUNG_CANCER_D	-0,089	0,016	0,27497	1
REACTOME_METABOLISM_OF_STEROID_HORM	-0,089	0,016	0,00479	1
PEREZ_TP53_AND_TP63_TARGETS	-0,089	0,016	0,00076	1
GSE39820_CTRL_VS_IL1B_IL6_CD4_TCELL_	-0,088	0,016	0,008	1
GSE16522_MEMORY_VS_NAIVE_ANTI_CD3CD	-0,088	0,016	0,00357	1
GSE11057_PBMC_VS_MEM_CD4_TCELL_DN	-0,088	0,016	0,00071	1
PEDRIOLI_MIR31_TARGETS_DN	-0,088	0,016	0,00021	1
AMUNDSON_POOR_SURVIVAL_AFTER_GAMMA	-0,088	0,016	0,0025	1
SCHAEFFER_PROSTATE_DEVELOPMENT_6HR_	-0,088	0,016	0,00102	1
REGULATION_OF_IMMUNE_SYSTEM_PROCESS	-0,088	0,016	0,01962	1
ZHU_CMV_ALL_UP	-0,088	0,016	0,18287	1
GSE22886_IGM_MEMORY_BCELL_VS_BM_PLA	-0,088	0,016	0,00489	1
PID_TCRRASPATHWAY	-0,088	0,016	0,02098	1
DORN_ADENOVIRUS_INFECTION_12HR_DN	-0,088	0,016	0,05282	1

V$TTF1_Q6	-0,088	0,016	0,00044	1
BIOCARTA_ERYTH_PATHWAY	-0,088	0,016	0,00031	1
GSE2706_UNSTIM_VS_8H_LPS_AND_R848_I	-0,088	0,016	0,0092	1
GSE9037_CTRL_VS_LPS_1H_STIM_BMDM_UI	-0,088	0,016	0,00189	1
MCGARVEY_SILENCED_BY_METHYLATION_IN_	-0,088	0,016	0,00061	1
HORMONE_SECRETION	-0,088	0,016	0,00705	1
GTPASE_ACTIVITY	-0,088	0,016	0,00047	1
ELVIDGE_HYPOXIA_BY_DMOG_UP	-0,088	0,016	0,09638	1
REACTOME_NEPHRIN_INTERACTIONS	-0,088	0,016	0,26451	1
YAO_TEMPORAL_RESPONSE_TO_PROGESTER(	-0,088	0,016	0,00229	1
LEIN_CEREBELLUM_MARKERS	-0,088	0,016	0,00637	1
V$ISRE_01	-0,088	0,016	0,01039	1
KIM_ALL_DISORDERS_OLIGODENDROCYTE_NU	-0,088	0,016	0,01303	1
MODULE_471	-0,088	0,016	0,28166	1
RECEPTOR_ACTIVITY	-0,088	0,016	0,00195	1
BROWNE_HCMV_INFECTION_20HR_DN	-0,088	0,016	0,00765	1
LE_EGR2_TARGETS_DN	-0,088	0,016	0,00033	1
ATPASE_ACTIVITY	-0,088	0,016	0,01112	1
MULLIGHAN_NPM1_MUTATED_SIGNATURE_1_	-0,088	0,016	0,00292	1
REACTOME_TRANSFERRIN_ENDOCYTOSIS_ANI	-0,088	0,016	0,36301	1
GSE15659_CD45RA_NEG_CD4_TCELL_VS_RI	-0,088	0,016	0,01508	1
MYLLYKANGAS_AMPLIFICATION_HOT_SPOT_2	-0,088	0,016	0,04508	1
EXOPEPTIDASE_ACTIVITY	-0,088	0,016	0,00192	1
PID_RHODOPSIN_PATHWAY	-0,088	0,016	0,04351	1
PID_HDAC_CLASSII_PATHWAY	0,088	0,016	0,92684	1
GSE11057_NAIVE_VS_EFF_MEMORY_CD4_TC	-0,088	0,016	0,03496	1
GSE17721_CTRL_VS_LPS_4H_BMDM_DN	-0,088	0,016	0,00425	1
LIGAND_GATED_CHANNEL_ACTIVITY	-0,088	0,016	0,01209	1
GOLDRATH_NAIVE_VS_EFF_CD8_TCELL_UP	-0,088	0,016	0,00054	1
GSE37416_CTRL_VS_24H_F_TULARENSIS_L\	-0,088	0,016	0,00722	1
REACTOME_IL_3_5_AND_GM_CSF_SIGNALIN(	-0,088	0,016	0,04448	1
GSE360_L_DONOVANI_VS_B_MALAYI_HIGH_	-0,088	0,016	0,0439	1
REACTOME_P38MAPK_EVENTS	-0,088	0,016	0,07832	1
MORF_PRKAG1	-0,088	0,016	0,48262	1
STARK_HYPPOCAMPUS_22Q11_DELETION_UI	-0,088	0,016	0,00911	1
GSE7764_NKCELL_VS_SPLENOCYTE_UP	-0,088	0,016	0,00152	1
GSE17721_POLYIC_VS_CPG_24H_BMDM_UP	-0,088	0,016	0,00117	1
AMINO_ACID_DERIVATIVE_METABOLIC_PROCI	-0,088	0,016	0,00195	1
V$STAT5A_04	-0,088	0,016	0,00028	1
KENNY_CTNNB1_TARGETS_UP	-0,088	0,016	0,18058	1
BIOCARTA_PPARA_PATHWAY	-0,088	0,017	0,00251	1
CYSTEINE_TYPE_ENDOPEPTIDASE_ACTIVITY	-0,088	0,017	0,14048	1

MODULE_361	-0,088	0,017	0,0045	1
GSE18791_CTRL_VS_NEWCASTLE_VIRUS_DC	-0,088	0,017	0,01283	1
KEGG_PRIMARY_IMMUNODEFICIENCY	-0,088	0,017	0,01887	1
GTGCAAT,MIR-25,MIR-32,MIR-92,MIR-363,MIR-	-0,088	0,017	0,00338	1
IVANOVA_HEMATOPOIESIS_STEM_CELL_SHOI	-0,088	0,017	0,00161	1
GSE7460_WT_VS_FOXP3_HET_ACT_WITH_T	-0,088	0,017	0,00019	1
REACTOME_RNA_POL_II_PRE_TRANSCRIPTION	-0,088	0,017	0,12442	1
GSE10325_LUPUS_CD4_TCELL_VS_LUPUS_B	-0,088	0,017	0,00249	1
PROTEIN_TARGETING	-0,088	0,017	0,15329	1
GSE17974_IL4_AND_ANTI_IL12_VS_UNTREA	-0,088	0,017	0,00562	1
RORIE_TARGETS_OF_EWSR1_FLI1_FUSION_UI	-0,088	0,017	0,00189	1
TOMLINS_PROSTATE_CANCER_UP	-0,088	0,017	0,0009	1
TURJANSKI_MAPK8_AND_MAPK9_TARGETS	-0,088	0,017	0,00477	1
GSE14769_UNSTIM_VS_40MIN_LPS_BMDM_D	-0,088	0,017	0,02233	1
GSE17721_12H_VS_24H_CPG_BMDM_UP	-0,088	0,017	0,00262	1
CTTTGCA,MIR-527	-0,088	0,017	0,00754	1
TGCACTT,MIR-519C,MIR-519B,MIR-519A	-0,088	0,017	0,00256	1
GSE17721_LPS_VS_PAM3CSK4_1H_BMDM_D	-0,088	0,017	0,01544	1
GSE17974_1H_VS_72H_UNTREATED_IN_VIT	-0,088	0,017	0,01389	1
KOHOUTEK_CCNT1_TARGETS	-0,088	0,017	0,01108	1
KEGG_MISMATCH_REPAIR	-0,088	0,017	0,07425	1
GCM_RAP2A	-0,088	0,017	0,08003	1
SENSORY_PERCEPTION_OF_CHEMICAL_STIMUI	-0,088	0,017	0,02181	1
DNA_HELICASE_ACTIVITY	-0,088	0,017	0,04531	1
GSE18791_CTRL_VS_NEWCASTLE_VIRUS_DC	-0,087	0,017	0,00014	1
KEGG_CYTOKINE_CYTOKINE_RECEPTOR_INTEI	-0,087	0,017	0,01351	1
APOPTOTIC_PROGRAM	-0,087	0,017	0,05675	1
KAPOSI_LIVER_CANCER_MET_DN	0,087	0,017	0,08582	1
AKL_HTLV1_INFECTION_DN	-0,087	0,017	0,02465	1
BUYTAERT_PHOTODYNAMIC_THERAPY_STRES	-0,087	0,017	0,01484	1
DOANE_BREAST_CANCER_CLASSES_UP	0,087	0,017	0,5539	1
MODULE_521	-0,087	0,017	0,02534	1
V$IK1_01	-0,087	0,017	0,00022	1
PID_IL23PATHWAY	-0,087	0,017	0,02541	1
MA_PITUITARY_FETAL_VS_ADULT_DN	0,087	0,017	0,06346	1
GARY_CD5_TARGETS_UP	-0,087	0,017	0,00741	1
GSE22886_NAIVE_CD4_TCELL_VS_NKCELL_	-0,087	0,017	0,00028	1
CHARAFE_BREAST_CANCER_LUMINAL_VS_ME	0,087	0,017	0,74601	1
MODULE_522	-0,087	0,017	0,07253	1
V$TEF_Q6	-0,087	0,017	0,00065	1
V$HMEF2_Q6	-0,087	0,017	0,00126	1
BIOCARTA_EDG1_PATHWAY	-0,087	0,017	0,0213	1

chr9q32	-0,087	0,017	0,00071	1
MODULE_511	-0,087	0,017	0,13459	1
MODULE_419	-0,087	0,017	0,00255	1
chr2q11	-0,087	0,017	0,00019	1
GSE9988_ANTI_TREM1_VS_VEHICLE_TREATE	-0,087	0,017	0,15104	1
PID_TCPTP_PATHWAY	-0,087	0,017	0,04915	1
JAZAG_TGFB1_SIGNALING_DN	-0,087	0,017	0,00337	1
DACOSTA_UV_RESPONSE_VIA_ERCC3_COMM	-0,087	0,017	0,05062	1
ACTIVATION_OF_JNK_ACTIVITY	-0,087	0,017	0,02627	1
PICCALUGA_ANGIOIMMUNOBLASTIC_LYMPHON	-0,087	0,017	0,00065	1
GSE7460_TREG_VS_TCONV_ACT_UP	-0,087	0,017	0,00924	1
SHIPP_DLBCL_VS_FOLLICULAR_LYMPHOMA_U	-0,087	0,017	0,49158	1
ENK_UV_RESPONSE_KERATINOCYTE_UP	-0,087	0,017	0,04369	1
MODULE_139	0,087	0,017	0,64048	1
ATGCTGC,MIR-103,MIR-107	-0,087	0,017	0,00124	1
YANG_BCL3_TARGETS_DN	-0,087	0,017	0,0015	1
MORF_ARL3	-0,087	0,017	0,00116	1
KEGG_PROPANOATE_METABOLISM	0,087	0,017	0,0435	1
REGULATION_OF_PROTEIN_IMPORT_INTO_NU(	-0,087	0,017	0,08268	1
MORF_UNG	-0,087	0,017	0,43834	1
GAUSSMANN_MLL_AF4_FUSION_TARGETS_A_	-0,087	0,017	0,00157	1
KRAS.BREAST_UP.V1_DN	-0,087	0,018	0,00038	1
BRUINS_UVC_RESPONSE_VIA_TP53_GROUP_E	-0,087	0,018	0,00189	1
MOREIRA_RESPONSE_TO_TSA_DN	-0,087	0,018	0,01004	1
REACTOME_SCFSKP2_MEDIATED_DEGRADATI(	-0,087	0,018	0,44193	1
CGTSACG_V$PAX3_B	-0,087	0,018	0,0024	1
GSE360_DC_VS_MAC_L_DONOVANI_UP	-0,087	0,018	0,01534	1
PID_VEGFR1_2_PATHWAY	-0,087	0,018	0,04628	1
KAYO_CALORIE_RESTRICTION_MUSCLE_UP	-0,087	0,018	0,00158	1
KAECH_DAY15_EFF_VS_MEMORY_CD8_TCELL	-0,087	0,018	0,01646	1
GSE22886_CD8_TCELL_VS_BCELL_NAIVE_D	-0,087	0,018	0,0096	1
chryq11	0,087	0,018	0,0083	1
ENDORIBONUCLEASE_ACTIVITY	-0,087	0,018	0,52571	1
MODULE_1	-0,087	0,018	0,0002	1
LIANG_HEMATOPOIESIS_STEM_CELL_NUMBER	-0,087	0,018	0,00742	1
GSE339_EX_VIVO_VS_IN_CULTURE_CD8POS_	-0,087	0,018	0,01496	1
HERNANDEZ_MITOTIC_ARREST_BY_DOCETAXE	-0,087	0,018	0,00407	1
JI_RESPONSE_TO_FSH_UP	-0,087	0,018	0,0259	1
KEGG_GLYCOSAMINOGLYCAN_BIOSYNTHESIS_	-0,087	0,018	0,26967	1
PHOSPHORIC_MONOESTER_HYDROLASE_ACTI	-0,087	0,018	0,00066	1
PID_IL4_2PATHWAY	-0,087	0,018	0,00187	1
GSE32423_IL7_VS_IL7_IL4_MEMORY_CD8_T	-0,087	0,018	0,00593	1

GSE22886_NAIVE_BCELL_VS_BLOOD_PLASM	-0,087	0,018	0,00038	1
GSE17974_CTRL_VS_ACT_IL4_AND_ANTI_IL	-0,087	0,018	0,00081	1
ACACTGG,MIR-199A,MIR-199B	-0,087	0,018	0,00049	1
ZHANG_TLX_TARGETS_DN	-0,087	0,018	0,0011	1
PID_AR_NONGENOMIC_PATHWAY	-0,087	0,018	0,00462	1
chr3q13	-0,087	0,018	0,00457	1
HONMA_DOCETAXEL_RESISTANCE	-0,087	0,018	0,43523	1
MEK_UP.V1_UP	-0,087	0,018	0,06828	1
REACTOME_NOTCH_HLH_TRANSCRIPTION_PA	-0,087	0,018	0,01478	1
PETRETTO_HEART_MASS_QTL_CIS_DN	-0,087	0,018	0,01396	1
chr4p14	-0,087	0,018	0,03832	1
REACTOME_INWARDLY_RECTIFYING_K_CHANN	-0,087	0,018	0,00209	1
GSE30962_PRIMARY_VS_SECONDARY_ACUTE	-0,087	0,018	0,00326	1
SCHLOSSER_MYC_TARGETS_REPRESSED_BY_S	-0,087	0,018	0,50885	1
MODULE_257	-0,087	0,018	0,00085	1
RADMACHER_AML_PROGNOSIS	-0,087	0,018	0,0045	1
KEGG_CHEMOKINE_SIGNALING_PATHWAY	-0,086	0,018	0,02213	1
BYSTRYKH_HEMATOPOIESIS_STEM_CELL_RUN	-0,086	0,018	0,00233	1
GSE31082_DP_VS_CD8_SP_THYMOCYTE_DN	-0,086	0,018	0,00015	1
FINAK_BREAST_CANCER_SDPP_SIGNATURE	-0,086	0,018	0,00105	1
GSE20366_TREG_VS_TCONV_DN	-0,086	0,018	0,00385	1
REACTOME_SIGNALING_BY_INSULIN_RECEPTC	-0,086	0,018	0,00179	1
GSE20366_TREG_VS_NAIVE_CD4_TCELL_HC	-0,086	0,018	0,00253	1
GSE18148_CBFB_KO_VS_WT_TREG_UP	-0,086	0,018	0,00032	1
GSE17721_POLYIC_VS_PAM3CSK4_6H_BMDI	-0,086	0,018	0,01859	1
GSE29617_CTRL_VS_DAY3_TIV_FLU_VACCII	-0,086	0,018	0,00293	1
BUCKANOVICH_T_LYMPHOCYTE_HOMING_ON_	-0,086	0,018	0,00027	1
MILI_PSEUDOPODIA_CHEMOTAXIS_DN	-0,086	0,018	0,01592	1
POSITIVE_REGULATION_OF_MULTICELLULAR_	-0,086	0,018	0,01707	1
MODULE_71	-0,086	0,018	0,00219	1
ST_P38_MAPK_PATHWAY	-0,086	0,018	0,00377	1
PEPTIDE_METABOLIC_PROCESS	-0,086	0,018	0,00222	1
ZWANG_EGF_PERSISTENTLY_DN	-0,086	0,018	0,00134	1
3_5_EXONUCLEASE_ACTIVITY	-0,086	0,018	0,09803	1
MODULE_256	-0,086	0,018	0,00022	1
MODULE_562	-0,086	0,018	0,15259	1
PID_ARF6DOWNSTREAMPATHWAY	-0,086	0,019	0,14838	1
RASHI_RESPONSE_TO_IONIZING_RADIATION_6	-0,086	0,019	0,05264	1
GSE17721_LPS_VS_PAM3CSK4_6H_BMDM_D	-0,086	0,019	0,19002	1
MATZUK_SPERMATOGONIA	-0,086	0,019	0,00059	1
TONKS_TARGETS_OF_RUNX1_RUNX1T1_FUSI	-0,086	0,019	0,00094	1
chr6q	-0,086	0,019	0,12398	1

ATAGGAA,MIR-202	-0,086	0,019	0,00242	1
GSE22886_TH1_VS_TH2_12H_ACT_DN	-0,086	0,019	0,00173	1
CORTICAL_CYTOSKELETON	0,086	0,019	0,0781	1
YAGI_AML_WITH_T_8_21_TRANSLOCATION	-0,086	0,019	0,00516	1
OKAMOTO_LIVER_CANCER_MULTICENTRIC_O(	-0,086	0,019	0,00312	1
KRAS.AMP.LUNG_UP.V1_DN	-0,086	0,019	0,00031	1
VECCHI_GASTRIC_CANCER_ADVANCED_VS_E	-0,086	0,019	0,01247	1
JAZAG_TGFB1_SIGNALING_VIA_SMAD4_UP	-0,086	0,019	0,00993	1
chr16q23	0,086	0,019	0,0075	1
HORTON_SREBF_TARGETS	-0,086	0,019	0,28962	1
RODWELL_AGING_KIDNEY_DN	-0,086	0,019	0,00082	1
REACTOME_REGULATION_OF_MRNA_STABILIT	-0,086	0,019	0,41462	1
GSE9650_EFFECTOR_VS_EXHAUSTED_CD8_T	-0,086	0,019	0,00078	1
GSE3982_CTRL_VS_LPS_4H_MAC_UP	-0,086	0,019	0,01599	1
RAHMAN_TP53_TARGETS_PHOSPHORYLATED	-0,086	0,019	0,73572	1
NAGASHIMA_EGF_SIGNALING_UP	-0,086	0,019	0,00016	1
MORF_TFDP2	-0,086	0,019	0,00064	1
GSE29618_LAIV_VS_TIV_FLU_VACCINE_DAY	-0,086	0,019	0,0048	1
ZHAN_MULTIPLE_MYELOMA_UP	-0,086	0,019	0,04146	1
GSE22886_NAIVE_TCELL_VS_NKCELL_DN	-0,086	0,019	0,0489	1
REGULATION_OF_TRANSFORMING_GROWTH_F	-0,086	0,019	0,00014	1
GOLGI_MEMBRANE	0,086	0,019	0,09053	1
GSE14308_TH17_VS_NATURAL_TREG_DN	-0,086	0,019	0,00219	1
GSE17721_0.5H_VS_8H_POLYIC_BMDM_DN	-0,086	0,019	0,00721	1
GSE3982_BCELL_VS_NKCELL_UP	-0,086	0,019	0,01919	1
MAHAJAN_RESPONSE_TO_IL1A_UP	-0,086	0,019	0,00684	1
HSIAO_HOUSEKEEPING_GENES	-0,086	0,019	0,02853	1
GSE360_HIGH_VS_LOW_DOSE_B_MALAYI_M/	-0,086	0,019	0,0058	1
GSE17974_2H_VS_72H_UNTREATED_IN_VIT	-0,086	0,019	0,03553	1
REACTOME_POL_SWITCHING	-0,086	0,019	0,07223	1
GSE22886_NAIVE_CD4_TCELL_VS_NKCELL_	-0,086	0,019	0,05429	1
LEIN_MEDULLA_MARKERS	-0,086	0,019	0,00282	1
KRIGE_RESPONSE_TO_TOSEDOSTAT_6HR_UP	-0,086	0,019	0,0243	1
PID_CXCR3PATHWAY	-0,086	0,019	0,05617	1
GSE33513_TCF7_KO_VS_HET_EARLY_THYMI	-0,086	0,019	0,0016	1
RECEPTOR_COMPLEX	-0,086	0,019	0,00504	1
GSE360_DC_VS_MAC_B_MALAYI_LOW_DOSE	-0,086	0,019	0,00961	1
GNF2_EGFR	-0,086	0,019	0,01987	1
V$NKX22_01	-0,086	0,019	0,00015	1
ZHOU_INFLAMMATORY_RESPONSE_LIVE_DN	-0,086	0,019	0,00091	1
WANG_SMARCE1_TARGETS_UP	-0,086	0,019	0,00452	1
IL21_UP.V1_DN	-0,086	0,019	0,00042	1

ROY_WOUND_BLOOD_VESSEL_UP	-0,086	0,019	0,01547	1
KUNINGER_IGF1_VS_PDGFB_TARGETS_DN	-0,086	0,019	0,01117	1
REACTOME_CREB_PHOSPHORYLATION_THROU	-0,086	0,019	0,01589	1
REACTOME_UNFOLDED_PROTEIN_RESPONSE	-0,086	0,019	0,37318	1
KLEIN_PRIMARY_EFFUSION_LYMPHOMA_UP	-0,086	0,019	0,05525	1
OUELLET_OVARIAN_CANCER_INVASIVE_VS_L	-0,086	0,019	0,99334	1
GSE20366_TREG_VS_NAIVE_CD4_TCELL_DN	-0,086	0,019	0,00514	1
GSE20715_0H_VS_48H_OZONE_TLR4_KO_L	-0,086	0,019	0,00018	1
GSE13484_12H_VS_3H_YF17D_VACCINE_ST	-0,086	0,02	0,01339	1
PID_IL8CXCR1_PATHWAY	-0,086	0,02	0,02743	1
STAMBOLSKY_RESPONSE_TO_VITAMIN_D3_D	-0,086	0,02	0,0365	1
V$LFA1_Q6	-0,086	0,02	0,00054	1
ALONSO_METASTASIS_DN	-0,086	0,02	0,04063	1
CAR_WBSCR22	-0,085	0,02	0,00963	1
SHIN_B_CELL_LYMPHOMA_CLUSTER_2	-0,085	0,02	0,00813	1
TAVOR_CEBPA_TARGETS_UP	-0,085	0,02	0,05169	1
GSE24142_EARLY_THYMIC_PROGENITOR_VS_	-0,085	0,02	0,01457	1
BOYLAN_MULTIPLE_MYELOMA_C_CLUSTER_D	-0,085	0,02	0,00832	1
PID_ERBB1_RECEPTOR_PROXIMAL_PATHWAY	-0,085	0,02	0,10957	1
MORF_RPA1	-0,085	0,02	0,57495	1
GSE360_L_DONOVANI_VS_M_TUBERCULOSIS	-0,085	0,02	0,02251	1
GSE17721_CTRL_VS_POLYIC_8H_BMDM_DN	-0,085	0,02	0,00176	1
GSE13306_RA_VS_UNTREATED_TCONV_UP	-0,085	0,02	0,00023	1
MODULE_211	-0,085	0,02	0,13399	1
GSE3982_MEMORY_CD4_TCELL_VS_BCELL_D	-0,085	0,02	0,03156	1
GAJATE_RESPONSE_TO_TRABECTEDIN_UP	-0,085	0,02	0,03274	1
GCM_PPM1D	-0,085	0,02	0,0151	1
GSE3982_CTRL_VS_PMA_STIM_EOSINOPHIL_	-0,085	0,02	0,00756	1
MARKEY_RB1_CHRONIC_LOF_DN	-0,085	0,02	0,06583	1
GSE12845_NAIVE_VS_DARKZONE_GC_TONSI	-0,085	0,02	0,00255	1
FURUKAWA_DUSP6_TARGETS_PCI35_UP	-0,085	0,02	0,01397	1
TCTGATC,MIR-383	0,085	0,02	0,07926	1
ORGANELLE_LOCALIZATION	-0,085	0,02	0,05994	1
GNF2_PPP6C	-0,085	0,02	0,21104	1
chr3q21	-0,085	0,02	0,03898	1
KAECH_NAIVE_VS_DAY15_EFF_CD8_TCELL_	-0,085	0,02	0,03265	1
GSE17721_POLYIC_VS_GARDIQUIMOD_2H_BM	-0,085	0,02	0,00023	1
FAELT_B_CLL_WITH_VH3_21_UP	-0,085	0,02	0,08388	1
BIOCARTA_HSP27_PATHWAY	-0,085	0,02	0,00886	1
GSE10239_NAIVE_VS_MEMORY_CD8_TCELL_	-0,085	0,02	0,08796	1
TCCCCAC,MIR-491	-0,085	0,02	0,00074	1
KEGG_SULFUR_METABOLISM	0,085	0,02	0,05282	1

BIOCARTA_PAR1_PATHWAY	-0,085	0,02	0,00392	1
MODULE_388	-0,085	0,02	0,20526	1
DAVICIONI_RHABDOMYOSARCOMA_PAX_FOXO	-0,085	0,02	0,06307	1
GSE7460_TREG_VS_TCONV_ACT_DN	-0,085	0,02	0,00044	1
KIM_GASTRIC_CANCER_CHEMOSENSITIVITY	-0,085	0,02	0,00241	1
V$RREB1_01	-0,085	0,02	0,00254	1
REACTOME_RNA_POL_III_CHAIN_ELONGATION	-0,085	0,02	0,09439	1
GSE360_CTRL_VS_M_TUBERCULOSIS_DC_UP	-0,085	0,02	0,00245	1
MCDOWELL_ACUTE_LUNG_INJURY_DN	-0,085	0,02	0,00013	1
chr13q32	-0,085	0,02	0,04972	1
ATP_DEPENDENT_HELICASE_ACTIVITY	-0,085	0,02	0,17625	1
CONCANNON_APOPTOSIS_BY_EPOXOMICIN_U	-0,085	0,02	0,01875	1
V$CEBP_Q2	-0,085	0,02	0,00323	1
GSE20715_WT_VS_TLR4_KO_24H_OZONE_L	-0,085	0,02	0,00061	1
GSE13484_12H_UNSTIM_VS_YF17D_VACCIN	-0,085	0,02	0,04271	1
REACTOME_G_ALPHA1213_SIGNALLING_EVE	-0,085	0,02	0,0024	1
GSE26669_CD4_VS_CD8_TCELL_IN_MLR_CC	-0,085	0,02	0,00814	1
WIERENGA_STAT5A_TARGETS_GROUP1	-0,085	0,021	0,02176	1
MMS_MOUSE_LYMPH_HIGH_4HRS_UP	-0,085	0,021	0,19384	1
NEGATIVE_REGULATION_OF_RESPONSE_TO_S	-0,085	0,021	0,03327	1
CCGNMNNTNACG_UNKNOWN	-0,085	0,021	0,01357	1
GSE27786_LSK_VS_BCELL_DN	-0,085	0,021	0,01823	1
REACTOME_METABOLISM_OF_NUCLEOTIDES	-0,085	0,021	0,23537	1
DER_IFN_BETA_RESPONSE_DN	-0,085	0,021	0,01796	1
DE_YY1_TARGETS_DN	-0,085	0,021	0,06689	1
CHIARADONNA_NEOPLASTIC_TRANSFORMATI	-0,085	0,021	0,00248	1
GSE22886_NAIVE_VS_IGM_MEMORY_BCELL_	-0,085	0,021	0,05622	1
PID_INTEGRIN2_PATHWAY	-0,085	0,021	0,01044	1
GTACTGT,MIR-101	-0,085	0,021	0,00169	1
GSE7460_CTRL_VS_TGFB_TREATED_ACT_CD	-0,085	0,021	0,00521	1
POOLA_INVASIVE_BREAST_CANCER_UP	-0,085	0,021	0,0853	1
POTTI_PACLITAXEL_SENSITIVITY	-0,085	0,021	0,01542	1
REACTOME_PLATELET_ACTIVATION_SIGNALI	-0,085	0,021	0,00193	1
GNF2_NS	-0,085	0,021	0,40276	1
REGULATION_OF_T_CELL_PROLIFERATION	-0,085	0,021	0,04791	1
GSE22886_NAIVE_CD8_TCELL_VS_MONOCY	-0,085	0,021	0,00485	1
TAKEDA_TARGETS_OF_NUP98_HOXA9_FUSIO	-0,085	0,021	0,00094	1
GNF2_SERPINI2	-0,085	0,021	0,04642	1
RAFFEL_VEGFA_TARGETS_DN	-0,085	0,021	0,32139	1
GSE339_EX_VIVO_VS_IN_CULTURE_CD4CD8	-0,085	0,021	0,03568	1
NUCLEOLAR_PART	-0,085	0,021	0,24546	1
WINTER_HYPOXIA_METAGENE	-0,085	0,021	0,05528	1

GTP_BINDING	-0,085	0,021	0,01512	1
GSE20715_WT_VS_TLR4_KO_LUNG_UP	-0,085	0,021	0,00509	1
GSE17721_LPS_VS_POLYIC_6H_BMDM_DN	-0,085	0,021	0,05104	1
HAEGERSTRAND_RESPONSE_TO_IMATINIB	-0,085	0,021	0,00031	1
GSE17721_LPS_VS_GARDIQUIMOD_12H_BMD	-0,084	0,021	0,00742	1
PID_FANCONI_PATHWAY	-0,084	0,021	0,06316	1
MCDOWELL_ACUTE_LUNG_INJURY_UP	-0,084	0,021	0,02253	1
SMID_BREAST_CANCER_LUMINAL_B_UP	0,084	0,021	0,3858	1
BIOCARTA_GABA_PATHWAY	-0,084	0,021	0,16856	1
BIOCARTA_GPCR_PATHWAY	-0,084	0,021	0,0036	1
GSE29614_DAY3_VS_DAY7_TIV_FLU_VACCI	-0,084	0,021	0,00036	1
chr6p24	-0,084	0,021	0,07	1
REACTOME_CTLA4_INHIBITORY_SIGNALING	-0,084	0,021	0,14121	1
RAMASWAMY_METASTASIS_DN	-0,084	0,021	0,00066	1
BASSO_CD40_SIGNALING_DN	-0,084	0,021	0,00783	1
ZHOU_INFLAMMATORY_RESPONSE_LPS_UP	-0,084	0,021	0,00973	1
MILICIC_FAMILIAL_ADENOMATOUS_POLYPOSIS	-0,084	0,021	0,36693	1
GSE17974_1H_VS_72H_UNTREATED_IN_VIT	-0,084	0,021	0,13801	1
RB_P130_DN.V1_DN	-0,084	0,021	0,15166	1
MODULE_133	-0,084	0,021	0,88608	1
GSE10325_LUPUS_CD4_TCELL_VS_LUPUS_B	-0,084	0,021	0,03323	1
GSE11924_TH1_VS_TH2_CD4_TCELL_DN	-0,084	0,021	0,00597	1
CASORELLI_ACUTE_PROMYELOCYTIC_LEUKEM	-0,084	0,021	0,00131	1
GSE17721_12H_VS_24H_GARDIQUIMOD_BMI	-0,084	0,021	0,04507	1
REACTOME_IL_2_SIGNALING	-0,084	0,021	0,03893	1
GSE3982_MAST_CELL_VS_BASOPHIL_DN	-0,084	0,021	0,0013	1
KINSEY_TARGETS_OF_EWSR1_FLII_FUSION_D	-0,084	0,021	0,0002	1
SANSOM_APC_MYC_TARGETS	-0,084	0,021	0,0035	1
STEGMEIER_PRE-MITOTIC_CELL_CYCLE_REGUL	-0,084	0,021	0,01617	1
GSE18791_CTRL_VS_NEWCASTLE_VIRUS_DC	-0,084	0,022	0,00029	1
SANA_TNF_SIGNALING_DN	-0,084	0,022	0,00052	1
GSE14308_TH1_VS_NAIVE_CD4_TCELL_UP	-0,084	0,022	0,01608	1
KRAS.PROSTATE_UP.V1_DN	-0,084	0,022	0,02334	1
TURASHVILI_BREAST_LOBULAR_CARCINOMA_	-0,084	0,022	0,00541	1
chr2q36	-0,084	0,022	0,02846	1
LASTOWSKA_NEUROBLASTOMA_COPY_NUMB	-0,084	0,022	0,05439	1
GSE17721_CTRL_VS_LPS_24H_BMDM_UP	-0,084	0,022	0,00089	1
GCACTTT,MIR-17-5P,MIR-20A,MIR-106A,MIR-1	-0,084	0,022	0,00599	1
GSE360_CTRL_VS_B_MALAYI_LOW_DOSE_M	-0,084	0,022	0,01177	1
KEGG_ADIPOCYTOKINE_SIGNALING_PATHWAY	-0,084	0,022	0,00045	1
REGULATION_OF_BIOLOGICAL_QUALITY	-0,084	0,022	0,00071	1
GSE9988_ANTI_TREM1_VS_CTRL_TREATED_	-0,084	0,022	0,04866	1

CHARAFE_BREAST_CANCER_LUMINAL_VS_BA	0,084	0,022	0,2674	1
ST_T_CELL_SIGNAL_TRANSDUCTION	-0,084	0,022	0,01823	1
GSE10325_CD4_TCELL_VS_BCELL_DN	-0,084	0,022	0,02356	1
CERAMIDE_METABOLIC_PROCESS	0,084	0,022	0,1415	1
GSE13229_IMM_VS_MATURE_NKCELL_DN	-0,084	0,022	0,00248	1
HOSHIDA_LIVER_CANCER_SUBCLASS_S1	-0,084	0,022	0,03963	1
HOFFMAN_CLOCK_TARGETS_DN	-0,084	0,022	0,02084	1
GSE26669_CD4_VS_CD8_TCELL_IN_MLR_DN	-0,084	0,022	0,00198	1
PID_IL3_PATHWAY	-0,084	0,022	0,01984	1
GSE15750_DAY6_VS_DAY10_EFF_CD8_TCEI	-0,084	0,022	0,00015	1
LEE_LIVER_CANCER_E2F1_DN	-0,084	0,022	0,13987	1
BIOCARTA_RAS_PATHWAY	-0,084	0,022	0,06072	1
GSE17721_CTRL_VS_CPG_4H_BMDM_UP	-0,084	0,022	0,00167	1
RRNA_METABOLIC_PROCESS	-0,084	0,022	0,15103	1
ZHANG_TLX_TARGETS_60HR_UP	-0,084	0,022	0,00035	1
GNF2_GLTSCR2	-0,084	0,022	0,00738	1
REACTOME_YAP1_AND_WWTR1_TAZ_STIMUL	-0,084	0,022	0,03846	1
CHESLER_BRAIN_QTL_CIS	-0,084	0,022	0,00034	1
BIOCARTA_PLCE_PATHWAY	-0,084	0,022	0,00314	1
GSE17974_2H_VS_72H_UNTREATED_IN_VIT	-0,084	0,022	0,10171	1
SMID_BREAST_CANCER_RELAPSE_IN_LIVER_L	-0,084	0,022	0,26158	1
REACTOME_XENOBIOTICS	0,084	0,022	0,12669	1
PHOSPHOINOSITIDE_METABOLIC_PROCESS	0,084	0,022	0,06443	1
DNA_REPLICATION_INITIATION	-0,084	0,022	0,14665	1
GSE18791_UNSTIM_VS_NEWCATSLE_VIRUS_	-0,084	0,022	0,00038	1
HALMOS_CEBPA_TARGETS_DN	-0,084	0,022	0,00033	1
GSE1448_CTRL_VS_ANTI_VBETA5_DP_THYM	-0,084	0,022	0,004	1
CORTICAL_ACTIN_CYTOSKELETON	0,084	0,022	0,07667	1
TNCATNTCCYR_UNKNOWN	-0,084	0,022	0,00133	1
PRC1_BMI_UP.V1_UP	-0,084	0,022	0,00174	1
GSE22886_CD8_TCELL_VS_BCELL_NAIVE_UI	-0,084	0,022	0,00782	1
ZHOU_INFLAMMATORY_RESPONSE_LIVE_UP	-0,084	0,022	0,0059	1
IKEDA_MIR1_TARGETS_UP	-0,084	0,022	0,06694	1
LIU_CMYB_TARGETS_UP	-0,084	0,022	0,00447	1
RECEPTOR_SIGNALING_PROTEIN_SERINE_THRI	-0,084	0,022	0,0295	1
GSE22886_NAIVE_VS_IGG_IGA_MEMORY_BCI	-0,084	0,022	0,05227	1
chr2p13	-0,084	0,023	0,0622	1
chr2q23	-0,084	0,023	0,00515	1
PAPASPYRIDONOS_UNSTABLE_ATEROSCLERO	-0,084	0,023	0,00061	1
GACAATC,MIR-219	-0,084	0,023	0,00053	1
REACTOME_SLC_MEDIATED_TRANSMEMBRANI	-0,084	0,023	0,04655	1
VANHARANTA_UTERINE_FIBROID_DN	-0,084	0,023	0,00041	1

CELLULAR_RESPONSE_TO_EXTRACELLULAR_!	-0,084	0,023	0,04314	1
GSE13738_RESTING_VS_BYSTANDER_ACTIV/	-0,084	0,023	0,07295	1
GSE16522_MEMORY_VS_NAIVE_ANTI_CD3CD	-0,084	0,023	0,03512	1
FIGUEROA_AML_METHYLATION_CLUSTER_5_I	-0,083	0,023	0,00222	1
GSE20715_WT_VS_TLR4_KO_6H_OZONE_LU	-0,083	0,023	0,00022	1
HAMAI_APOPTOSIS_VIA_TRAIL_DN	-0,083	0,023	0,03514	1
MONOCARBOXYLIC_ACID_METABOLIC_PROCES	-0,083	0,023	0,00024	1
GSE22886_CD4_TCELL_VS_BCELL_NAIVE_UI	-0,083	0,023	0,0047	1
NEWMAN_ERCC6_TARGETS_UP	-0,083	0,023	0,09427	1
DELACROIX_RARG_BOUND_MEF	-0,083	0,023	0,002	1
MODULE_418	-0,083	0,023	0,00866	1
GSE3982_CTRL_VS_LPS_48H_DC_DN	-0,083	0,023	0,05759	1
GSE6269_FLU_VS_STREP_PNEUMO_INF_PBM(	-0,083	0,023	0,00045	1
CHARAFE_BREAST_CANCER_BASAL_VS_MESI	-0,083	0,023	0,00466	1
ZHAN_MULTIPLE_MYELOMA_CD2_UP	-0,083	0,023	0,01253	1
SENSORY_PERCEPTION_OF_TASTE	-0,083	0,023	0,02632	1
GSE11057_CD4_EFF_MEM_VS_PBMC_UP	-0,083	0,023	0,00036	1
HOFFMANN_PRE_BI_TO_LARGE_PRE_BII_LYMI	-0,083	0,023	0,00897	1
GSE20366_EX_VIVO_VS_HOMEOSTATIC_CON	-0,083	0,023	0,00363	1
AAGTCCA,MIR-422B,MIR-422A	-0,083	0,023	0,00489	1
PAL_PRMT5_TARGETS_DN	-0,083	0,023	0,00773	1
AACATTC,MIR-409-3P	-0,083	0,023	0,0024	1
GSE12845_IGD_POS_BLOOD_VS_DARKZONE_	-0,083	0,023	0,00049	1
TRACEY_RESISTANCE_TO_IFNA2_DN	-0,083	0,023	0,02137	1
GROSS_HYPOXIA_VIA_ELK3_AND_HIF1A_UP	-0,083	0,023	0,02541	1
KEGG_HEDGEHOG_SIGNALING_PATHWAY	-0,083	0,023	0,0017	1
POSITIVE_REGULATION_OF_T_CELL_PROLIFEF	-0,083	0,023	0,04944	1
FUNG_IL2_SIGNALING_2	-0,083	0,023	0,04711	1
GSE17721_PAM3CSK4_VS_GADIQUIMOD_1H_	-0,083	0,023	0,0035	1
VALK_AML_CLUSTER_9	-0,083	0,023	0,01349	1
GRADE_METASTASIS_DN	-0,083	0,023	0,40866	1
MODULE_408	0,083	0,023	0,06971	1
BARIS_THYROID_CANCER_DN	-0,083	0,023	0,00697	1
GSE17721_CTRL_VS_LPS_2H_BMDM_UP	-0,083	0,023	0,01469	1
REACTOME_IL_RECEPTOR_SHC_SIGNALING	-0,083	0,024	0,04109	1
AMIT_SERUM_RESPONSE_40_MCF10A	-0,083	0,024	0,00243	1
GSE3982_CTRL_VS_LPS_4H_MAC_DN	-0,083	0,024	0,05673	1
AGCACTT,MIR-93,MIR-302A,MIR-302B,MIR-302	-0,083	0,024	0,00764	1
CHUANG_OXIDATIVE_STRESS_RESPONSE_UP	-0,083	0,024	0,03302	1
ANATOMICAL_STRUCTURE_FORMATION	-0,083	0,024	0,01021	1
SCHLOSSER_MYC_AND_SERUM_RESPONSE_SY	-0,083	0,024	0,09251	1
KOKKINAKIS_METHIONINE_DEPRIVATION_96H	-0,083	0,024	0,00362	1

BIOCARTA_ERK5_PATHWAY	-0,083	0,024	0,00425	1
GSE17721_LPS_VS_GARDIQUIMOD_12H_BMD	-0,083	0,024	0,18937	1
ZHENG_FOXP3_TARGETS_IN_T_LYMPHOCYTE_	-0,083	0,024	0,00246	1
GSE17721_CPG_VS_GARDIQUIMOD_4H_BMDI	-0,083	0,024	0,02049	1
HOLLEMAN_DAUNORUBICIN_B_ALL_UP	-0,083	0,024	0,05962	1
REGULATION_OF_LYMPHOCYTE_ACTIVATION	-0,083	0,024	0,03128	1
GSE17721_LPS_VS_PAM3CSK4_12H_BMDM_	-0,083	0,024	0,18557	1
GSE13485_DAY1_VS_DAY7_YF17D_VACCINI	-0,083	0,024	0,00031	1
GSE7852_TREG_VS_TCONV_THYMUS_UP	-0,083	0,024	0,00575	1
DELASERNA_TARGETS_OF_MYOD_AND_SMAF	-0,083	0,024	0,00323	1
SMID_BREAST_CANCER_RELAPSE_IN_BRAIN_I	0,083	0,024	0,1816	1
PRC2_EDD_UP.V1_DN	-0,083	0,024	0,01006	1
TCANNTGAY_V$SREBP1_01	-0,083	0,024	0,00031	1
GSE339_EX_VIVO_VS_IN_CULTURE_CD4POS_	-0,083	0,024	0,10064	1
CHOI_ATL_CHRONIC_VS_ACUTE_DN	-0,083	0,024	0,35762	1
KAYO_CALORIE_RESTRICTION_MUSCLE_DN	-0,083	0,024	0,02852	1
OXFORD_RALB_TARGETS_DN	-0,083	0,024	0,94976	1
JOHNSTONE_PARVB_TARGETS_2_UP	-0,083	0,024	0,00375	1
METALLOENDOPEPTIDASE_ACTIVITY	-0,082	0,024	0,06581	1
V$HNF4_DR1_Q3	-0,082	0,024	0,00068	1
GSE17721_CTRL_VS_CPG_8H_BMDM_UP	-0,082	0,024	0,00019	1
LUCAS_HNF4A_TARGETS_DN	-0,082	0,025	0,02039	1
REACTOME_PEPTIDE_HORMONE_BIOSYNTHESI	-0,082	0,025	0,04454	1
GSE22886_NAIVE_VS_IGG_IGA_MEMORY_BCI	-0,082	0,025	0,00038	1
GAL_LEUKEMIC_STEM_CELL_DN	-0,082	0,025	0,05551	1
GSE29618_BCELL_VS_PDC_DAY7_FLU_VAC(	-0,082	0,025	0,01198	1
chr9q31	-0,082	0,025	0,06675	1
INWARD_RECTIFIER_POTASSIUM_CHANNEL_A	-0,082	0,025	0,02122	1
MATZUK_MALE_REPRODUCTION_SERTOLI	-0,082	0,025	0,0002	1
REACTOME_REGULATION_OF_SIGNALING_BY_	-0,082	0,025	0,12174	1
GSE17721_CPG_VS_GARDIQUIMOD_1H_BMDI	-0,082	0,025	0,00048	1
GENE_SILENCING	-0,082	0,025	0,14372	1
MOTAMED_RESPONSE_TO_ANDROGEN_DN	-0,082	0,025	0,08408	1
STRESS_ACTIVATED_PROTEIN_KINASE_SIGNA	-0,082	0,025	0,00428	1
HOSHIDA_LIVER_CANCER_SUBCLASS_S2	-0,082	0,025	0,02078	1
PID_S1P_S1P3_PATHWAY	-0,082	0,025	0,01326	1
GSE17721_POLYIC_VS_CPG_2H_BMDM_DN	-0,082	0,025	0,01219	1
ST_G_ALPHA_I_PATHWAY	-0,082	0,025	0,07233	1
ZHONG_SECRETOME_OF_LUNG_CANCER_AND	-0,082	0,025	0,2218	1
CHANDRAN_METASTASIS_UP	-0,082	0,025	0,04168	1
CHEMNITZ_RESPONSE_TO_PROSTAGLANDIN_I	-0,082	0,025	0,00013	1
TRICARBOXYLIC_ACID_CYCLE_INTERMEDIATE_	0,082	0,025	0,01327	1

AXON_GUIDANCE	-0,082	0,025	0,0024	1
MODULE_169	-0,082	0,025	0,01218	1
MODULE_61	-0,082	0,025	0,64797	1
GSE7460_TCONV_VS_TREG_LN_DN	-0,082	0,025	0,0078	1
BERENJENO_ROCK_SIGNALING_NOT_VIA_RHC	-0,082	0,025	0,00351	1
RHO_GTPASE_ACTIVATOR_ACTIVITY	-0,082	0,025	0,00196	1
PID_EPOPATHWAY	-0,082	0,025	0,0064	1
KASLER_HDAC7_TARGETS_1_UP	-0,082	0,025	0,00313	1
REACTOME_ERKS_ARE_INACTIVATED	0,082	0,025	0,04414	1
GSE17721_PAM3CSK4_VS_GADIQUIMOD_8H_	-0,082	0,025	0,08152	1
MORF_GSPT1	-0,082	0,026	0,40289	1
ZHOU_INFLAMMATORY_RESPONSE_FIMA_DN	-0,082	0,026	0,00016	1
KEGG_ONE_CARBON_POOL_BY_FOLATE	-0,082	0,026	0,0757	1
G_PROTEIN_COUPLED_RECEPTOR_BINDING	-0,082	0,026	0,08014	1
GALE_APL_WITH_FLT3_MUTATED_UP	-0,082	0,026	0,06056	1
DE_YY1_TARGETS_UP	-0,082	0,026	0,00353	1
TORCHIA_TARGETS_OF_EWSR1_FLI1_FUSION	-0,082	0,026	0,08939	1
JNK_CASCADE	-0,082	0,026	0,00478	1
MAHADEVAN_IMATINIB_RESISTANCE_DN	-0,082	0,026	0,00098	1
REACTOME_TRANSMISSION_ACROSS_CHEMIC/	-0,082	0,026	0,00113	1
REACTOME_RECEPTOR_LIGAND_BINDING_INIT	-0,082	0,026	0,00677	1
REACTOME_GOLGI_ASSOCIATED_VESICLE_BIC	0,082	0,026	0,00866	1
MODULE_56	-0,082	0,026	0,45636	1
V$OCT1_04	-0,082	0,026	0,00076	1
GSE17580_UNINFECTED_VS_S_MANSONI_INF	-0,082	0,026	0,10158	1
GSE17721_POLYIC_VS_GARDIQUIMOD_6H_BN	-0,082	0,026	0,02111	1
HUMMERICH_SKIN_CANCER_PROGRESSION_UP	-0,082	0,026	0,04734	1
REACTOME_GABA_SYNTHESIS_RELEASE_REUI	0,082	0,026	0,11335	1
ATTCTTT,MIR-186	-0,082	0,026	0,01484	1
ACTGCAG,MIR-17-3P	-0,082	0,026	0,00592	1
PID_MYC_REPRESSPATHWAY	-0,082	0,026	0,01293	1
DACOSTA_UV_RESPONSE_VIA_ERCC3_TTD_I	-0,082	0,026	0,02407	1
ENDOSOME_TRANSPORT	0,082	0,026	0,25667	1
SCHUETZ_BREAST_CANCER_DUCTAL_INVASI\	-0,081	0,026	0,01248	1
PID_CD40_PATHWAY	-0,081	0,026	0,01313	1
ALK_DN.V1_DN	-0,081	0,026	0,00072	1
WATANABE_RECTAL_CANCER_RADIOTHERAP'	-0,081	0,026	0,19144	1
BEGUM_TARGETS_OF_PAX3_FOXO1_FUSION_	-0,081	0,026	0,00028	1
chr11q	-0,081	0,026	0,00133	1
PIGF_UP.V1_UP	-0,081	0,026	0,00625	1
BIOCARTA_SPPA_PATHWAY	-0,081	0,026	0,09069	1
GSE360_LOW_DOSE_B_MALAYI_VS_M_TUBE	-0,081	0,026	0,00888	1

GSE1432_1H_VS_24H_IFNG_MICROGLIA_UP	-0,081	0,026	0,00546	1
GLYCOLIPID_METABOLIC_PROCESS	-0,081	0,026	0,0842	1
MODULE_528	-0,081	0,026	0,41682	1
ACTIN_CYTOSKELETON_ORGANIZATION_AND_	-0,081	0,027	0,00031	1
THEILGAARD_NEUTROPHIL_AT_SKIN_WOUND_	-0,081	0,027	0,01548	1
BROWNE_HCMV_INFECTION_8HR_UP	-0,081	0,027	0,00965	1
GSE17721_CTRL_VS_LPS_6H_BMDM_UP	-0,081	0,027	0,00115	1
NUCLEAR_MATRIX	-0,081	0,027	0,02425	1
FONTAINE_FOLLICULAR_THYROID_ADENOMA_	-0,081	0,027	0,00825	1
HAMAI_APOPTOSIS_VIA_TRAIL_UP	-0,081	0,027	0,04703	1
chr1q23	-0,081	0,027	0,00835	1
chr16q11	-0,081	0,027	0,44403	1
GSE360_CTRL_VS_L_MAJOR_DC_UP	-0,081	0,027	0,00029	1
GSE9988_LOW_LPS_VS_ANTI_TREM1_AND_	-0,081	0,027	0,06971	1
MODULE_188	-0,081	0,027	0,01006	1
BOSCO_ALLERGEN_INDUCED_TH2_ASSOCIATI	-0,081	0,027	0,00541	1
REACTOME_ADAPTIVE_IMMUNE_SYSTEM	-0,081	0,027	0,08543	1
GNF2_IL2RB	-0,081	0,027	0,02261	1
LEUKOCYTE_DIFFERENTIATION	-0,081	0,027	0,04319	1
ACETYLCHOLINE_BINDING	-0,081	0,027	0,10471	1
REACTOME_REPAIR_SYNTHESIS_FOR_GAP_FIL	-0,081	0,027	0,0802	1
COATED_VESICLE	-0,081	0,027	0,05377	1
GSE39820_CTRL_VS_TGFBETA3_IL6_IL23A_	-0,081	0,027	0,00698	1
IKEDA_MIR133_TARGETS_UP	-0,081	0,027	0,06787	1
G_PROTEIN_COUPLED_RECEPTOR_ACTIVITY	-0,081	0,027	0,00111	1
REACTOME_SIGNALING_BY_HIPPO	-0,081	0,027	0,13858	1
OXFORD_RALA_TARGETS_UP	-0,081	0,027	0,64827	1
PROTEIN_COMPLEX_ASSEMBLY	-0,081	0,027	0,00111	1
HOLLEMAN_PREDNISOLONE_RESISTANCE_ALL	-0,081	0,027	0,02611	1
GSE29618_PRE_VS_DAY7_POST_LAIV_FLU_	-0,081	0,027	0,02383	1
REACTOME_LAGGING_STRAND_SYNTHESIS	-0,081	0,027	0,10963	1
REACTOME_CD28_CO_STIMULATION	-0,081	0,027	0,04304	1
GSE17721_4_VS_24H_CPG_BMDM_DN	-0,081	0,027	0,00205	1
GNF2_SPINK1	-0,081	0,027	0,00865	1
TONKS_TARGETS_OF_RUNX1_RUNX1T1_FUSI	-0,081	0,027	0,00507	1
MODULE_237	-0,081	0,027	0,00748	1
MORF_LMO1	-0,081	0,027	0,00031	1
MORF_RAN	-0,081	0,027	0,27121	1
V$AR_02	-0,081	0,027	0,00038	1
MODULE_218	-0,081	0,028	0,35047	1
SRC_UP.V1_UP	-0,081	0,028	0,0009	1
PID_HIVNEFPATHWAY	-0,081	0,028	0,02696	1

SIMBULAN_UV_RESPONSE_NORMAL_UP	-0,081	0,028	0,16868	1
SEROTONIN_RECEPTOR_ACTIVITY	-0,081	0,028	0,00165	1
KEGG_T_CELL_RECEPTOR_SIGNALING_PATHW	-0,081	0,028	0,02282	1
PID_WNT_SIGNALING_PATHWAY	-0,081	0,028	0,11469	1
AGUIRRE_PANCREATIC_CANCER_COPY_NUMBI	-0,081	0,028	0,00516	1
ZEMBUTSU_SENSITIVITY_TO_VINBLASTINE	-0,081	0,028	0,07316	1
BHATI_G2M_ARREST_BY_2METHOXYESTRADI(	-0,081	0,028	0,00111	1
LEE_LIVER_CANCER_MYC_E2F1_UP	-0,081	0,028	0,19426	1
GSE14308_NAIVE_CD4_TCELL_VS_NATURAL	-0,081	0,028	0,00467	1
LY_AGING_MIDDLE_UP	-0,081	0,028	0,04943	1
GSE14000_4H_VS_16H_LPS_DC_UP	-0,081	0,028	0,04267	1
MODULE_330	-0,081	0,028	0,00031	1
SCHLOSSER_SERUM_RESPONSE_AUGMENTED_	-0,081	0,028	0,03644	1
CADWELL_ATG16L1_TARGETS_UP	-0,081	0,028	0,00102	1
CASORELLI_APL_SECONDARY_VS_DE_NOVO_	-0,081	0,028	0,00147	1
GSE27786_CD4_TCELL_VS_NKCELL_DN	-0,081	0,028	0,06744	1
PID_ER_NONGENOMIC_PATHWAY	-0,081	0,028	0,00379	1
DNA_DIRECTED_RNA_POLYMERASEII_CORE_C	-0,08	0,028	0,02437	1
REACTOME_REGULATION_OF_RHEB_GTPASE_	-0,08	0,028	0,07406	1
GAZIN_EPIGENETIC_SILENCING_BY_KRAS	-0,08	0,028	0,22956	1
GOUYER_TATI_TARGETS_UP	-0,08	0,028	0,01588	1
REACTOME_INITIAL_TRIGGERING_OF_COMPLEI	0,08	0,028	0,27393	1
MODULE_87	-0,08	0,028	0,06705	1
CENTROSOME_ORGANIZATION_AND_BIOGENE:	-0,08	0,028	0,01703	1
GSE9006_HEALTHY_VS_TYPE_2_DIABETES_I	-0,08	0,028	0,0627	1
KUUSELO_PANCREATIC_CANCER_19Q13_AMI	-0,08	0,028	0,02478	1
BIOCARTA_P53_PATHWAY	-0,08	0,028	0,00194	1
TGCCTTA,MIR-124A	-0,08	0,028	0,00579	1
NEGATIVE_REGULATION_OF_MYELOID_CELL_	-0,08	0,028	0,00189	1
GSE17721_POLYIC_VS_CPG_0.5H_BMDM_DN	-0,08	0,028	0,00204	1
chr12p13	-0,08	0,028	0,01578	1
HOWLIN_CITED1_TARGETS_1_UP	-0,08	0,028	0,00184	1
MORF_RAD21	-0,08	0,028	0,56426	1
PID_THROMBIN_PAR4_PATHWAY	-0,08	0,028	0,00197	1
MARSON_FOXP3_TARGETS_STIMULATED_UP	-0,08	0,028	0,02042	1
MORF_CNTN1	-0,08	0,029	0,00138	1
PID_LKB1_PATHWAY	-0,08	0,029	0,00034	1
MILI_PSEUDOPODIA_HAPTOTAXIS_UP	-0,08	0,029	0,13913	1
MISSIAGLIA_REGULATED_BY_METHYLATION_I	-0,08	0,029	0,09608	1
PID_TGFBRPATHWAY	-0,08	0,029	0,00288	1
MIKKELSEN_PLURIPOTENT_STATE_UP	-0,08	0,029	0,10591	1
MODULE_83	-0,08	0,029	0,0614	1

SCHLOSSER_SERUM_RESPONSE_DN	-0,08	0,029	0,10544	1
GSE29618_PRE_VS_DAY7_FLU_VACCINE_BC	-0,08	0,029	0,00018	1
LANG_MYB_FAMILY_TARGETS	-0,08	0,029	0,2789	1
GSE339_CD4POS_VS_CD4CD8DN_DC_IN_CUI	-0,08	0,029	0,0211	1
FERRANDO_TAL1_NEIGHBORS	-0,08	0,029	0,05691	1
V$HOXA3_01	-0,08	0,029	0,01421	1
CTTGTAT,MIR-381	-0,08	0,029	0,00564	1
MODULE_278	-0,08	0,029	0,75744	1
GSE14308_TH2_VS_TH1_DN	-0,08	0,029	0,00506	1
NUCLEOTIDE_METABOLIC_PROCESS	-0,08	0,029	0,09265	1
GSE13484_3H_UNSTIM_VS_YF17D_VACCINE	-0,08	0,029	0,00245	1
HU_ANGIOGENESIS_UP	-0,08	0,029	0,00108	1
ADDYA_ERYTHROID_DIFFERENTIATION_BY_HE	-0,08	0,029	0,02808	1
BARRIER_CANCER_RELAPSE_NORMAL_SAMPL	-0,08	0,029	0,00085	1
GSE17974_CTRL_VS_ACT_IL4_AND_ANTI_IL	-0,08	0,029	0,00046	1
chr21q22	-0,08	0,029	0,02349	1
GCM_NCAM1	-0,08	0,029	0,01044	1
BIOCARTA_CASPASE_PATHWAY	-0,08	0,029	0,12213	1
GSE27786_LIN_NEG_VS_BCELL_DN	-0,08	0,029	0,00041	1
GSE11864_UNTREATED_VS_CSF1_IFNG_PAM	-0,08	0,029	0,10488	1
GSE29618_BCELL_VS_PDC_UP	-0,08	0,029	0,01536	1
STRIATED_MUSCLE_CONTRACTION_GO_0006	-0,08	0,029	0,00319	1
VEGF_A_UP.V1_UP	-0,08	0,029	0,00073	1
HASLINGER_B_CLL_WITH_17P13_DELETION	-0,08	0,029	0,11539	1
SECRETORY_PATHWAY	0,08	0,029	0,56484	1
CHLORIDE_CHANNEL_ACTIVITY	-0,08	0,029	0,00501	1
BIOCARTA_PITX2_PATHWAY	-0,08	0,029	0,00955	1
WNT_UP.V1_DN	-0,08	0,029	0,00142	1
SCHAEFFER_PROSTATE_DEVELOPMENT_AND_	-0,08	0,029	0,00057	1
REGULATION_OF_TRANSLATION	-0,08	0,029	0,04424	1
PECE_MAMMARY_STEM_CELL_UP	-0,08	0,029	0,00394	1
chr4q35	-0,08	0,029	0,03095	1
chr7p21	-0,08	0,029	0,00234	1
ENDOPLASMIC_RETICULUM_LUMEN	-0,08	0,029	0,3833	1
PENG_LEUCINE_DEPRIVATION_UP	-0,08	0,03	0,02883	1
GSE22886_DAY0_VS_DAY1_MONOCYTE_IN_	-0,08	0,03	0,28302	1
PID_P38_MKK3_6PATHWAY	-0,08	0,03	0,00046	1
CHYLA_CBFA2T3_TARGETS_DN	-0,08	0,03	0,00089	1
GSE13229_IMM_VS_INTMATURE_NKCELL_DN	-0,08	0,03	0,00109	1
GNF2_KISS1	-0,08	0,03	0,00654	1
YGTCCTTGR_UNKNOWN	-0,08	0,03	0,00238	1
CACGTTT,MIR-302A	-0,08	0,03	0,01497	1

GSE20366_TREG_VS_TCONV_UP	-0,08	0,03	0,00734	1
chr7q21	-0,08	0,03	0,00031	1
POSITIVE_REGULATION_OF_CELLULAR_PROTE	-0,08	0,03	0,00719	1
THUM_MIR21_TARGETS_HEART_DISEASE_UP	-0,08	0,03	0,00076	1
PID_HES_HEYPATHWAY	-0,08	0,03	0,00108	1
ST_B_CELL_ANTIGEN_RECEPTOR	-0,08	0,03	0,11237	1
REACTOME_LIPOPROTEIN_METABOLISM	-0,08	0,03	0,01282	1
REACTOME_RAS_ACTIVATION_UOPN_CA2_IN	-0,08	0,03	0,01607	1
POSITIVE_REGULATION_OF_PROTEIN_METABC	-0,079	0,03	0,00672	1
TERAMOTO_OPN_TARGETS_CLUSTER_6	-0,079	0,03	0,00141	1
REACTOME_RNA_POL_II_TRANSCRIPTION_PRE	-0,079	0,03	0,16271	1
MIKKELSEN_IPS_ICP_WITH_H3K27ME3	-0,079	0,03	0,00044	1
GSE7460_TREG_VS_TCONV_ACT_WITH_TGFI	-0,079	0,03	0,00548	1
chr18q23	-0,079	0,03	0,00153	1
chr4q11	-0,079	0,03	0,02965	1
MODULE_6	-0,079	0,03	0,00321	1
GRUETZMANN_PANCREATIC_CANCER_DN	-0,079	0,03	0,00021	1
MORF_STK17A	-0,079	0,03	0,00146	1
GSE3982_EOSINOPHIL_VS_TH2_UP	-0,079	0,03	0,00659	1
GSE2826_WT_VS_XID_BCELL_DN	-0,079	0,03	0,00744	1
GSE17721_POLYIC_VS_CPG_8H_BMDM_UP	-0,079	0,03	0,01217	1
REACTOME_THE_ACTIVATION_OF_ARYLSULF/	-0,079	0,03	0,04066	1
KYNG_ENVIRONMENTAL_STRESS_RESPONSE_	-0,079	0,031	0,03649	1
REACTOME_ENDOGENOUS_STEROLS	-0,079	0,031	0,01812	1
AMIT_EGF_RESPONSE_240_MCF10A	-0,079	0,031	0,03529	1
FIGUEROA_AML_METHYLATION_CLUSTER_2_I	-0,079	0,031	0,05119	1
MODULE_146	-0,079	0,031	0,00304	1
MOOTHA_GLYCOLYSIS	0,079	0,031	0,02153	1
YAO_TEMPORAL_RESPONSE_TO_PROGESTERC	-0,079	0,031	0,03392	1
GSE17721_POLYIC_VS_GARDIQUIMOD_2H_BI	-0,079	0,031	0,08365	1
GSE2706_UNSTIM_VS_8H_R848_DC_DN	-0,079	0,031	0,10987	1
MARIADASON_RESPONSE_TO_BUTYRATE_CUF	-0,079	0,031	0,01095	1
MANTOVANI_VIRAL_GPCR_SIGNALING_UP	-0,079	0,031	0,02471	1
GSE17721_0.5H_VS_8H_PAM3CSK4_BMDM_	-0,079	0,031	0,07018	1
GSE17721_12H_VS_24H_CPG_BMDM_DN	-0,079	0,031	0,0705	1
MEMBRANE_FRACTION	-0,079	0,031	0,02394	1
SCHOEN_NFKB_SIGNALING	-0,079	0,031	0,0287	1
GSE360_L_MAJOR_VS_T_GONDII_MAC_UP	-0,079	0,031	0,0036	1
GSE9006_TYPE_1_DIABETES_AT_DX_VS_4M	-0,079	0,031	0,02206	1
INDUCTION_OF_APOPTOSIS_BY_INTRACELLUL	-0,079	0,031	0,00874	1
MA_MYELOID_DIFFERENTIATION_DN	-0,079	0,031	0,05873	1
MIYAGAWA_TARGETS_OF_EWSR1_ETS_FUSIC	-0,079	0,031	0,00393	1

INGRAM_SHH_TARGETS_UP	-0,079	0,031	0,00041	1
GSE17721_ALL_VS_24H_PAM3CSK4_BMDM_	-0,079	0,031	0,05803	1
KATSANOU_ELAVL1_TARGETS_UP	-0,079	0,031	0,00234	1
GSE15930_NAIVE_VS_24H_IN_VITRO_STIM_	-0,079	0,031	0,00086	1
MIKKELSEN_NPC_LCP_WITH_H3K4ME3	-0,079	0,031	0,00051	1
FIRESTEIN_CTNNB1_PATHWAY_AND_PROLIFE	-0,079	0,031	0,09637	1
SH3_SH2_ADAPTOR_ACTIVITY	-0,079	0,031	0,00081	1
GSE17721_CPG_VS_GARDIQUIMOD_24H_BMI	-0,079	0,031	0,00803	1
ENVELOPE	-0,079	0,031	0,19135	1
ORGANELLE_ENVELOPE	-0,079	0,031	0,19135	1
KEGG_NON_HOMOLOGOUS_END_JOINING	-0,079	0,031	0,09154	1
DURAND_STROMA_MAX_DN	-0,079	0,031	0,00091	1
ST_G_ALPHA_S_PATHWAY	0,079	0,031	0,44517	1
GSE13306_RA_VS_UNTREATED_MEM_CD4_T	-0,079	0,031	0,00489	1
GSE26928_NAIVE_VS_CXCR5_POS_CD4_TCE	-0,079	0,031	0,01194	1
GSE25087_TREG_VS_TCONV_ADULT_UP	-0,079	0,031	0,00721	1
V$HOX13_01	-0,079	0,031	0,00134	1
GSE10325_CD4_TCELL_VS_BCELL_UP	-0,079	0,031	0,00653	1
GSE3982_BCELL_VS_BASOPHIL_DN	-0,079	0,031	0,00268	1
PID_NFAT_TFPATHWAY	-0,079	0,031	0,02735	1
GHO_ATF5_TARGETS_UP	-0,079	0,031	0,05475	1
WORSCHECH_TUMOR_REJECTION_UP	-0,079	0,032	0,05241	1
GSE17721_LPS_VS_GARDIQUIMOD_8H_BMDM	-0,079	0,032	0,01439	1
GSE13484_12H_VS_3H_YF17D_VACCINE_ST	-0,079	0,032	0,01581	1
LIGASE_ACTIVITY	-0,079	0,032	0,07008	1
MODULE_81	-0,079	0,032	0,27436	1
MILI_PSEUDOPODIA	-0,079	0,032	0,09478	1
BASSO_HAIRY_CELL_LEUKEMIA_DN	-0,079	0,032	0,00061	1
GSE3982_BASOPHIL_VS_CENT_MEMORY_CD4	-0,079	0,032	0,02686	1
WHITESIDE_CISPLATIN_RESISTANCE_DN	-0,079	0,032	0,00148	1
VANOEVELEN_MYOGENESIS_SIN3A_TARGETS	-0,079	0,032	0,02044	1
PLASARI_TGFB1_TARGETS_1HR_UP	-0,079	0,032	0,00039	1
RAY_TARGETS_OF_P210_BCR_ABL_FUSION_	-0,079	0,032	0,0661	1
GSE17974_0.5H_VS_72H_IL4_AND_ANTI_IL	-0,079	0,032	0,02018	1
PROTEIN_BINDING_BRIDGING	-0,079	0,032	0,01248	1
GSE13485_CTRL_VS_DAY3_YF17D_VACCINE	-0,079	0,032	0,01036	1
GSE17974_IL4_AND_ANTI_IL12_VS_UNTREA	-0,079	0,032	0,00019	1
V$DR1_Q3	-0,079	0,032	0,00071	1
INTRACELLULAR_TRANSPORT	-0,079	0,032	0,13619	1
V$EVI1_05	-0,078	0,032	0,00073	1
GSE22886_IGG_IGA_MEMORY_BCELL_VS_BM	-0,078	0,032	0,00853	1
WAMUNYOKOLI_OVARIAN_CANCER_LMP_DN	-0,078	0,032	0,00249	1

KLEIN_PRIMARY_EFFUSION_LYMPHOMA_DN	-0,078	0,032	0,03925	1
REGULATION_OF_T_CELL_ACTIVATION	-0,078	0,032	0,04457	1
CATTTCA,MIR-203	-0,078	0,032	0,00491	1
BOYAULT_LIVER_CANCER_SUBCLASS_G1_DN	-0,078	0,032	0,05579	1
GCM_FANCL	-0,078	0,032	0,00486	1
DASU_IL6_SIGNALING_SCAR_DN	-0,078	0,032	0,00806	1
chr7q34	-0,078	0,032	0,18853	1
GSE22886_NAIVE_CD8_TCELL_VS_MEMORY_	-0,078	0,032	0,00136	1
KAECH_NAIVE_VS_MEMORY_CD8_TCELL_DN	-0,078	0,032	0,02475	1
NEWMAN_ERCC6_TARGETS_DN	-0,078	0,032	0,02558	1
REACTOME_NEGATIVE_REGULATION_OF_FGFI	-0,078	0,032	0,0007	1
V$OCT1_02	-0,078	0,032	0,00017	1
GSE360_L_DONOVANI_VS_T_GONDII_DC_UP	-0,078	0,033	0,00189	1
chr6p11	0,078	0,033	0,0706	1
GSE25087_TREG_VS_TCONV_FETUS_UP	-0,078	0,033	0,02746	1
REACTOME_SLBP_DEPENDENT_PROCESSING_(	-0,078	0,033	0,18512	1
RAS_PROTEIN_SIGNAL_TRANSDUCTION	-0,078	0,033	0,00445	1
GSE11057_EFF_MEM_VS_CENT_MEM_CD4_T	-0,078	0,033	0,0186	1
DAUER_STAT3_TARGETS_UP	-0,078	0,033	0,0407	1
HELLER_SILENCED_BY_METHYLATION_UP	-0,078	0,033	0,08324	1
BEHAVIOR	-0,078	0,033	0,06055	1
LIPID_BINDING	-0,078	0,033	0,00294	1
REACTOME_PLATELET_SENSITIZATION_BY_LI	-0,078	0,033	0,25901	1
V$PU1_Q6	-0,078	0,033	0,00261	1
PID_ECADHERIN_NASCENTAJ_PATHWAY	-0,078	0,033	0,27316	1
YAMASHITA_LIVER_CANCER_STEM_CELL_DN	-0,078	0,033	0,00288	1
REACTOME_NOD1_2_SIGNALING_PATHWAY	-0,078	0,033	0,19176	1
GSE15324_NAIVE_VS_ACTIVATED_CD8_TCE	-0,078	0,033	0,00059	1
chr19q13	-0,078	0,033	0,00205	1
GSE360_DC_VS_MAC_B_MALAYI_HIGH_DOSI	-0,078	0,033	0,00899	1
GSE14308_TH1_VS_INDUCED_TREG_UP	-0,078	0,033	0,03396	1
SMITH_TERT_TARGETS_DN	-0,078	0,033	0,01594	1
CELL_ACTIVATION	-0,078	0,033	0,03443	1
BIOCARTA_PYK2_PATHWAY	-0,078	0,033	0,05856	1
GCM_ZNF198	-0,078	0,033	0,04146	1
THEILGAARD_NEUTROPHIL_AT_SKIN_WOUND_	-0,078	0,033	0,24243	1
GSE24142_ADULT_VS_FETAL_DN3_THYMOC	-0,078	0,033	0,0134	1
DENDRITE	-0,078	0,033	0,15271	1
MCCLUNG_DELTA_FOSB_TARGETS_8WK	-0,078	0,033	0,00201	1
BIOCARTA_SARS_PATHWAY	-0,078	0,033	0,3896	1
SMID_BREAST_CANCER_RELAPSE_IN_BONE_L	0,078	0,033	0,15012	1
GSE3982_CENT_MEMORY_CD4_TCELL_VS_TI	-0,078	0,033	0,00059	1

BIOCARTA_RACCYCD_PATHWAY	-0,078	0,033	0,10028	1
V$TEL2_Q6	-0,078	0,034	0,01027	1
GSE17721_0.5H_VS_4H_LPS_BMDM_DN	-0,078	0,034	0,051	1
MORF_CSNK2B	-0,078	0,034	0,44976	1
BIOCARTA_EGF_PATHWAY	-0,078	0,034	0,01349	1
GSE17721_CTRL_VS_POLYIC_24H_BMDM_UF	-0,078	0,034	0,00117	1
REGULATION_OF_MAP_KINASE_ACTIVITY	-0,078	0,034	0,03148	1
GSE7460_FOXP3_MUT_VS_HET_ACT_TCONV	-0,078	0,034	0,00173	1
CELLULAR_CARBOHYDRATE_CATABOLIC_PRO	0,078	0,034	0,11199	1
chr1q24	-0,078	0,034	0,01547	1
GSE7852_TREG_VS_TCONV_DN	-0,078	0,034	0,00597	1
TCGA_GLIOBLASTOMA_MUTATED	-0,078	0,034	0,09922	1
QI_PLASMACYTOMA_UP	-0,078	0,034	0,02992	1
METALLOPEPTIDASE_ACTIVITY	-0,078	0,034	0,04082	1
TTCCGTT,MIR-191	-0,078	0,034	0,01291	1
HAHTOLA_CTCL_PATHOGENESIS	-0,078	0,034	0,04305	1
BLUM_RESPONSE_TO_SALIRASIB_UP	-0,078	0,034	0,63849	1
WU_SILENCED_BY_METHYLATION_IN_BLADDE	-0,078	0,034	0,00014	1
MODULE_525	-0,078	0,034	0,03866	1
APICAL_PART_OF_CELL	-0,078	0,034	0,17775	1
LEUKOCYTE_ACTIVATION	-0,078	0,034	0,03471	1
RECEPTOR_SIGNALING_PROTEIN_ACTIVITY	-0,078	0,034	0,01491	1
PENG_RAPAMYCIN_RESPONSE_UP	-0,078	0,034	0,00251	1
MODULE_149	-0,077	0,034	0,09017	1
PICCALUGA_ANGIOIMMUNOBLASTIC_LYMPHON	-0,077	0,034	0,00865	1
TURASHVILI_BREAST_DUCTAL_CARCINOMA_\	-0,077	0,034	0,0192	1
MODULE_111	-0,077	0,034	0,01008	1
BIOCARTA_KERATINOCYTE_PATHWAY	-0,077	0,035	0,00544	1
GSE20715_WT_VS_TLR4_KO_24H_OZONE_L	-0,077	0,035	0,00287	1
GSE31082_DN_VS_CD4_SP_THYMOCYTE_UP	-0,077	0,035	0,33755	1
KEGG_ALDOSTERONE_REGULATED_SODIUM_R	-0,077	0,035	0,00047	1
TONKS_TARGETS_OF_RUNX1_RUNX1T1_FUSI	-0,077	0,035	0,02215	1
TONKS_TARGETS_OF_RUNX1_RUNX1T1_FUSI	-0,077	0,035	0,00037	1
PROTEIN_KINASE_CASCADE	-0,077	0,035	0,00813	1
WEST_ADRENOCORTICAL_TUMOR_MARKERS_	-0,077	0,035	0,00071	1
FUNG_IL2_TARGETS_WITH_STAT5_BINDING_	-0,077	0,035	0,04638	1
chr9p	-0,077	0,035	0,1015	1
GSE17721_CTRL_VS_GARDIQUIMOD_6H_BMD	-0,077	0,035	0,01459	1
DETECTION_OF_CHEMICAL_STIMULUS	-0,077	0,035	0,00078	1
LIU_CDX2_TARGETS_DN	-0,077	0,035	0,07451	1
GSE14308_TH1_VS_NATURAL_TREG_DN	-0,077	0,035	0,00078	1
SHIN_B_CELL_LYMPHOMA_CLUSTER_5	-0,077	0,035	0,08705	1

SKELETAL_DEVELOPMENT	-0,077	0,035	0,006	1
MODULE_88	-0,077	0,035	0,00144	1
GSE17974_0H_VS_6H_IN_VITRO_ACT_CD4_	-0,077	0,035	0,00035	1
GSE17974_0H_VS_1H_IN_VITRO_ACT_CD4_	-0,077	0,035	0,07431	1
ATAACCT,MIR-154	-0,077	0,035	0,00274	1
MORF_PHB	-0,077	0,035	0,10006	1
ROPERO_HDAC2_TARGETS	-0,077	0,035	0,00598	1
FLECHNER_BIOPSY_KIDNEY_TRANSPLANT_OK	-0,077	0,035	0,02362	1
REACTOME_SEMA4D_INDUCED_CELL_MIGRAT	-0,077	0,035	0,00052	1
REACTOME_CELL_SURFACE_INTERACTIONS_A	-0,077	0,035	0,05441	1
GSE7852_TREG_VS_TCONV_LN_UP	-0,077	0,035	0,01883	1
MODULE_213	-0,077	0,035	0,00304	1
GSE10239_KLRG1INT_VS_KLRG1HIGH_EFF_C	-0,077	0,035	0,06617	1
KIM_GLIS2_TARGETS_UP	-0,077	0,035	0,00699	1
NIELSEN_GIST	-0,077	0,035	0,00355	1
GSE360_T_GONDII_VS_B_MALAYI_HIGH_DOS	-0,077	0,035	0,00205	1
ONDER_CDH1_TARGETS_2_UP	-0,077	0,035	0,00043	1
REGULATION_OF_NUCLEOCYTOPLASMIC_TRAI	-0,077	0,035	0,1557	1
chr3q22	-0,077	0,035	0,22619	1
BAKER_HEMATOPOESIS_STAT1_TARGETS	-0,077	0,036	0,00959	1
GSE20366_EX_VIVO_VS_DEC205_CONVERSI	-0,077	0,036	0,01385	1
PHONG_TNF_TARGETS_DN	-0,077	0,036	0,01254	1
RNA_EXPORT_FROM_NUCLEUS	-0,077	0,036	0,21675	1
GSE24081_CONTROLLER_VS_PROGRESSOR_H	-0,077	0,036	0,03157	1
REACTOME_FACILITATIVE_NA_INDEPENDENT_	-0,077	0,036	0,14938	1
GSE3982_DC_VS_BASOPHIL_UP	-0,077	0,036	0,02303	1
REACTOME_PRE_NOTCH_EXPRESSION_AND_P	-0,077	0,036	0,00768	1
JIANG_HYPOXIA_CANCER	-0,077	0,036	0,04187	1
GSE17721_0.5H_VS_12H_LPS_BMDM_UP	-0,077	0,036	0,03418	1
REACTOME_CD28_DEPENDENT_PI3K_AKT_SI	-0,077	0,036	0,0339	1
ZHONG_RESPONSE_TO_AZACITIDINE_AND_TS	-0,077	0,036	0,02596	1
V$XBP1_01	-0,077	0,036	0,05296	1
BOSCO_TH1_CYTOTOXIC_MODULE	-0,077	0,036	0,08469	1
GSE1460_DP_VS_CD4_THYMOCYTE_DN	-0,077	0,036	0,00518	1
ZHU_CMV_8_HR_DN	-0,077	0,036	0,00083	1
GSE17721_CTRL_VS_PAM3CSK4_1H_BMDM_	-0,077	0,036	0,01802	1
ANION_CHANNEL_ACTIVITY	-0,077	0,036	0,00708	1
REACTOME_ROLE_OF_DCC_IN_REGULATING_A	0,077	0,036	0,06354	1
ROVERSI_GLIOMA_COPY_NUMBER_UP	-0,077	0,036	0,00013	1
MODULE_275	-0,077	0,037	0,02643	1
TERAO_AOX4_TARGETS_SKIN_UP	-0,077	0,037	0,00154	1
GSE7460_FOXP3_MUT_VS_WT_ACT_WITH_T	-0,077	0,037	0,00017	1

GCM_CASP2	-0,077	0,037	0,02386	1
V$EVI1_02	-0,077	0,037	0,00284	1
ENDOSOME	-0,077	0,037	0,0468	1
NUCLEOTIDE_BIOSYNTHETIC_PROCESS	-0,077	0,037	0,19764	1
VALK_AML_WITH_FLT3_ITD	-0,077	0,037	0,01181	1
chr15q23	-0,077	0,037	0,00043	1
GSE17721_PAM3CSK4_VS_CPG_4H_BMDM_[	-0,077	0,037	0,04118	1
AGGTGCA,MIR-500	-0,076	0,037	0,00058	1
NEGATIVE_REGULATION_OF_ANGIOGENESIS	-0,076	0,037	0,03483	1
GSE22886_TCELL_VS_BCELL_NAIVE_UP	-0,076	0,037	0,01055	1
BERENJENO_TRANSFORMED_BY_RHOA_DN	-0,076	0,037	0,00076	1
GSE1432_CTRL_VS_IFNG_6H_MICROGLIA_UP	-0,076	0,037	0,00242	1
NING_CHRONIC_OBSTRUCTIVE_PULMONARY_[	-0,076	0,037	0,0038	1
BURTON_ADIPOGENESIS_9	-0,076	0,037	0,00582	1
V$HNF4_01_B	-0,076	0,037	0,0004	1
TAAYNRNNTCC_UNKNOWN	-0,076	0,037	0,00208	1
WIERENGA_STAT5A_TARGETS_DN	-0,076	0,037	0,00242	1
REACTOME_ADVANCED_GLYCOSYLATION_ENI	-0,076	0,037	0,12945	1
LIM_MAMMARY_LUMINAL_MATURE_UP	0,076	0,037	0,22768	1
GSE14308_TH2_VS_INDUCED_TREG_UP	-0,076	0,037	0,00017	1
MODULE_55	-0,076	0,037	0,00183	1
GSE17974_IL4_AND_ANTI_IL12_VS_UNTREA	-0,076	0,037	0,00079	1
POSITIVE_REGULATION_OF_JNK_ACTIVITY	-0,076	0,037	0,07556	1
GSE24142_DN2_VS_DN3_THYMOCYTE_UP	-0,076	0,037	0,02626	1
GAZDA_DIAMOND_BLACKFAN_ANEMIA_PROG	-0,076	0,037	0,05143	1
NOUSHMEHR_GBM_SOMATIC_MUTATED	-0,076	0,037	0,23442	1
KEEN_RESPONSE_TO_ROSIGLITAZONE_DN	-0,076	0,038	0,02281	1
MORF_RAP1A	-0,076	0,038	0,02788	1
REACTOME_FRS2_MEDIATED_CASCADE	-0,076	0,038	0,00174	1
V$HFH3_01	-0,076	0,038	0,0002	1
ACEVEDO_LIVER_TUMOR_VS_NORMAL_ADJA	-0,076	0,038	0,25751	1
GSE17721_CTRL_VS_GARDIQUIMOD_8H_BMD	-0,076	0,038	0,04995	1
CELLULAR_RESPONSE_TO_STIMULUS	-0,076	0,038	0,12873	1
GSE9988_ANTI_TREM1_VS_LPS_MONOCYTE_	-0,076	0,038	0,15151	1
COWLING_MYCN_TARGETS	-0,076	0,038	0,00614	1
GSE9650_NAIVE_VS_MEMORY_CD8_TCELL_[	-0,076	0,038	0,03227	1
IIZUKA_LIVER_CANCER_PROGRESSION_G1_G2	-0,076	0,038	0,91335	1
RAY_TARGETS_OF_P210_BCR_ABL_FUSION_	-0,076	0,038	0,00757	1
REGULATION_OF_LIPID_METABOLIC_PROCESS	-0,076	0,038	0,01153	1
BROCKE_APOPTOSIS_REVERSED_BY_IL6	-0,076	0,038	0,03845	1
FINETTI_BREAST_CANCERS_KINOME_BLUE	0,076	0,038	0,14287	1
GSE24142_EARLY_THYMIC_PROGENITOR_VS_	-0,076	0,038	0,00355	1

MARSON_BOUND_BY_FOXP3_STIMULATED	-0,076	0,038	0,03594	1
IZADPANAH_STEM_CELL_ADIPOSE_VS_BONE_	-0,076	0,038	0,00052	1
GSE360_L_DONOVANI_VS_B_MALAYI_HIGH_	-0,076	0,038	0,03911	1
RHODOPSIN_LIKE_RECEPTOR_ACTIVITY	-0,076	0,038	0,00085	1
CHEOK_RESPONSE_TO_MERCAPTOPURINE_AN	-0,076	0,038	0,14459	1
LU_TUMOR_VASCULATURE_UP	-0,076	0,038	0,03095	1
GSE17721_CTRL_VS_CPG_1H_BMDM_DN	-0,076	0,039	0,00073	1
POLYSACCHARIDE_BINDING	-0,076	0,039	0,00828	1
REACTOME_CLASS_C_3_METABOTROPIC_GLU	-0,076	0,039	0,11307	1
WEIGEL_OXIDATIVE_STRESS_BY_HNE_AND_T	-0,076	0,039	0,00032	1
POSITIVE_REGULATION_OF_PROTEIN_MODIFIC	-0,076	0,039	0,00133	1
CATGTAA,MIR-496	-0,076	0,039	0,00469	1
GSE1460_NAIVE_CD4_TCELL_ADULT_BLOOD	-0,076	0,039	0,00053	1
LIN_APC_TARGETS	-0,076	0,039	0,10585	1
BIOCARTA_SPRY_PATHWAY	-0,076	0,039	0,04572	1
PROTEOLYSIS	-0,076	0,039	0,03393	1
REACTOME_CHEMOKINE_RECEPTORS_BIND_CH	-0,076	0,039	0,09094	1
MALIK_REPRESSED_BY_ESTROGEN	-0,076	0,039	0,13357	1
GSE32423_CTRL_VS_IL7_MEMORY_CD8_TCE	-0,076	0,039	0,0002	1
BIOCARTA_IGF1_PATHWAY	-0,076	0,039	0,00013	1
BOYLAN_MULTIPLE_MYELOMA_C_D_DN	-0,076	0,039	0,00873	1
GSE22886_IGG_IGA_MEMORY_BCELL_VS_BLO	-0,076	0,039	0,00058	1
CELLULAR_HOMEOSTASIS	-0,076	0,039	0,01443	1
GSE12845_IGD_POS_BLOOD_VS_NAIVE_TON	-0,076	0,039	0,00112	1
PID_RHOA_REG_PATHWAY	-0,076	0,039	0,00029	1
PROTEIN_HOMODIMERIZATION_ACTIVITY	-0,076	0,039	0,001	1
BIOCARTA_VEGF_PATHWAY	-0,075	0,039	0,19417	1
KEGG_PYRIMIDINE_METABOLISM	-0,075	0,039	0,11075	1
BIOCARTA_ETS_PATHWAY	-0,075	0,039	0,03431	1
GSE27786_NKTCELL_VS_ERYTHROBLAST_UP	-0,075	0,04	0,00042	1
BIOCARTA_NFAT_PATHWAY	-0,075	0,04	0,01868	1
ALCALA_APOPTOSIS	-0,075	0,04	0,0815	1
REGULATION_OF_HEART_CONTRACTION	-0,075	0,04	0,00368	1
MORF_NME2	-0,075	0,04	0,07124	1
GSE12845_IGD_NEG_BLOOD_VS_NAIVE_TON	-0,075	0,04	0,22497	1
GSE17721_12H_VS_24H_GARDIQUIMOD_BMI	-0,075	0,04	0,0582	1
OUELLET_CULTURED_OVARIAN_CANCER_INV/	-0,075	0,04	0,10779	1
AMIT_EGF_RESPONSE_60_HELA	-0,075	0,04	0,00448	1
BIOCARTA_CHEMICAL_PATHWAY	-0,075	0,04	0,02307	1
DACOSTA_UV_RESPONSE_VIA_ERCC3_DN	-0,075	0,04	0,07876	1
GSE29617_CTRL_VS_TIV_FLU_VACCINE_PBN	-0,075	0,04	0,01381	1
TSUNODA_CISPLATIN_RESISTANCE_UP	-0,075	0,04	0,01554	1

MUELLER_COMMON_TARGETS_OF_AML_FUSI(	-0,075	0,04	0,17585	1
PID_RAC1_REG_PATHWAY	-0,075	0,04	0,00578	1
GSE1460_INTRATHYMIC_T_PROGENITOR_VS_	-0,075	0,041	0,00188	1
BIOCARTA_NO2IL12_PATHWAY	-0,075	0,041	0,05663	1
LEIN_ASTROCYTE_MARKERS	-0,075	0,041	0,00127	1
LYMPHOCYTE_ACTIVATION	-0,075	0,041	0,04909	1
MODULE_263	-0,075	0,041	0,16305	1
LEE_LIVER_CANCER_MYC_TGFA_DN	-0,075	0,041	0,02921	1
WANG_HCP_PROSTATE_CANCER	-0,075	0,041	0,01234	1
GSE9988_LPS_VS_LPS_AND_ANTI_TREM1_M	-0,075	0,041	0,05855	1
GAVIN_IL2_RESPONSIVE_FOXP3_TARGETS_UI	-0,075	0,041	0,13845	1
BIOCARTA_MEF2D_PATHWAY	-0,075	0,041	0,09332	1
CALCIUM_ION_TRANSPORT	-0,075	0,041	0,03574	1
YAGI_AML_WITH_11Q23_REARRANGED	-0,075	0,041	0,00216	1
MORF_DAP3	-0,075	0,041	0,56116	1
KONDO_HYPOXIA	-0,075	0,041	0,02127	1
HYDROLASE_ACTIVITY_ACTING_ON_CARBON_	-0,075	0,041	0,10818	1
WAGSCHAL_EHMT2_TARGETS_UP	-0,075	0,041	0,0708	1
MACROMOLECULE_LOCALIZATION	-0,075	0,041	0,04207	1
chr3p13	-0,075	0,041	0,0314	1
REACTOME_P53_INDEPENDENT_G1_S_DNA_I	-0,075	0,041	0,80909	1
MUNSHI_MULTIPLE_MYELOMA_DN	-0,075	0,041	0,40523	1
REACTOME_CA_DEPENDENT_EVENTS	-0,075	0,041	0,00021	1
GRAESSMANN_RESPONSE_TO_MC_AND_DOX(	-0,075	0,041	0,0076	1
COLLIS_PRKDC_REGULATORS	0,075	0,041	0,05073	1
GSE17721_CTRL_VS_PAM3CSK4_24H_BMDM	-0,075	0,041	0,00298	1
chr4q26	-0,075	0,041	0,00694	1
GYORFFY_MITOXANTRONE_RESISTANCE	-0,075	0,041	0,00105	1
REN_ALVEOLAR_RHABDOMYOSARCOMA_DN	-0,075	0,041	0,06418	1
LEIN_CHOROID_PLEXUS_MARKERS	-0,075	0,041	0,0007	1
NIKOLSKY_BREAST_CANCER_14Q22_AMPLIC(	-0,075	0,041	0,00528	1
BIOCARTA_MCALPAIN_PATHWAY	-0,075	0,041	0,16233	1
PODAR_RESPONSE_TO_ADAPHOSTIN_UP	-0,075	0,042	0,07532	1
BIOCARTA_TCR_PATHWAY	-0,075	0,042	0,01156	1
CYTOSOLIC_PART	0,075	0,042	0,50038	1
GSE20366_TREG_VS_NAIVE_CD4_TCELL_DE	-0,075	0,042	0,01544	1
MAINA_HYPOXIA_VHL_TARGETS_UP	-0,075	0,042	0,03074	1
GROSS_HIF1A_TARGETS_DN	-0,075	0,042	0,39385	1
GSE17974_0H_VS_72H_IN_VITRO_ACT_CD4	-0,075	0,042	0,0037	1
ZHENG_RESPONSE_TO_ARSENITE_UP	-0,075	0,042	0,27046	1
chr15q14	-0,075	0,042	0,01632	1
HARRIS_HYPOXIA	-0,075	0,042	0,09384	1

MORF_CTBP1	-0,075	0,042	0,44502	1
GSE13484_3H_UNSTIM_VS_YF17D_VACCINE	-0,075	0,042	0,12984	1
REGULATION_OF_ANGIOGENESIS	-0,075	0,042	0,02985	1
GSE1448_CTRL_VS_ANTI_VALPHA2_DP_THY	-0,075	0,042	0,00652	1
RODWELL_AGING_KIDNEY_UP	-0,075	0,042	0,02257	1
chr8q13	-0,075	0,042	0,2118	1
MODULE_43	0,074	0,042	0,32097	1
GSE360_HIGH_DOSE_B_MALAYI_VS_M_TUBE	-0,074	0,042	0,01116	1
GSE360_L_MAJOR_VS_B_MALAYI_HIGH_DOS	-0,074	0,042	0,03229	1
ACEVEDO_LIVER_TUMOR_VS_NORMAL_ADJA	-0,074	0,042	0,00027	1
ABE_INNER_EAR	-0,074	0,042	0,02226	1
GSE17721_POLYIC_VS_PAM3CSK4_12H_BMI	-0,074	0,042	0,00357	1
BROWNE_HCMV_INFECTION_1HR_UP	-0,074	0,042	0,00049	1
REACTOME_P53_DEPENDENT_G1_DNA_DAM/	-0,074	0,043	0,70507	1
V$HNF4ALPHA_Q6	-0,074	0,043	0,00033	1
WAGNER_APO2_SENSITIVITY	-0,074	0,043	0,30894	1
GSE360_DC_VS_MAC_M_TUBERCULOSIS_UP	-0,074	0,043	0,0267	1
GTGTCAA,MIR-514	-0,074	0,043	0,00832	1
GSE9006_HEALTHY_VS_TYPE_2_DIABETES_I	-0,074	0,043	0,04627	1
TIEN_INTESTINE_PROBIOTICS_24HR_DN	-0,074	0,043	0,24859	1
ONO_AML1_TARGETS_UP	-0,074	0,043	0,04719	1
SCHLESINGER_METHYLATED_DE_NOVO_IN_C/	-0,074	0,043	0,0007	1
chr2p15	-0,074	0,043	0,50849	1
COLIN_PILOCYTIC_ASTROCYTOMA_VS_GLIOBI	-0,074	0,043	0,2665	1
EXTRINSIC_TO_MEMBRANE	-0,074	0,043	0,01514	1
KANG_GIST_WITH_PDGFRA_DN	-0,074	0,043	0,1722	1
GSE22886_DC_VS_MONOCYTE_UP	-0,074	0,043	0,11492	1
JAK2_DN.V1_DN	-0,074	0,043	0,00161	1
CHEOK_RESPONSE_TO_HD_MTX_DN	-0,074	0,043	0,00813	1
INGA_TP53_TARGETS	-0,074	0,043	0,00032	1
SHAFFER_IRF4_TARGETS_IN_ACTIVATED_B_I	-0,074	0,043	0,92811	1
MIKKELSEN_MEF_ICP_WITH_H3K27ME3	-0,074	0,043	0,00631	1
ESTABLISHMENT_OF_LOCALIZATION	-0,074	0,044	0,00121	1
FERRARI_RESPONSE_TO_FENRETINIDE_UP	-0,074	0,044	0,05216	1
NEGATIVE_REGULATION_OF_TRANSFERASE_/	-0,074	0,044	0,00278	1
GSE26928_EFF_MEMORY_VS_CXCR5_POS_CI	-0,074	0,044	0,00225	1
KEGG_HYPERTROPHIC_CARDIOMYOPATHY_HCI	-0,074	0,044	0,00036	1
GSE9988_ANTI_TREM1_VS_LOW_LPS_MONO	-0,074	0,044	0,15574	1
SCHAEFFER_PROSTATE_DEVELOPMENT_AND_	-0,074	0,044	0,00018	1
GSE7852_LN_VS_FAT_TREG_DN	-0,074	0,044	0,00682	1
KEGG_B_CELL_RECEPTOR_SIGNALING_PATHW	-0,074	0,044	0,04352	1
MEMBRANE_LIPID_BIOSYNTHETIC_PROCESS	-0,074	0,044	0,00832	1

GSE3982_EOSINOPHIL_VS_CENT_MEMORY_CI	-0,074	0,044	0,04431	1
GCM_CSNK1D	0,074	0,044	0,08458	1
REGULATION_OF_CELLULAR_PH	0,074	0,044	0,94732	1
GSE17721_CTRL_VS_PAM3CSK4_0.5H_BMDI	-0,074	0,044	0,07938	1
SARCOMERE	-0,074	0,044	0,01583	1
GSE360_T_GONDII_VS_M_TUBERCULOSIS_MA	-0,074	0,044	0,01345	1
CARBOHYDRATE_CATABOLIC_PROCESS	0,074	0,044	0,13618	1
CARBON_CARBON_LYASE_ACTIVITY	0,074	0,044	0,04725	1
MODULE_455	-0,074	0,044	0,00365	1
MARKS_ACETYLATED_NON_HISTONE_PROTEII	-0,074	0,044	0,14565	1
KEGG_NATURAL_KILLER_CELL_MEDIATED_CY	-0,074	0,044	0,1435	1
DACOSTA_UV_RESPONSE_VIA_ERCC3_XPCS_	-0,074	0,044	0,02696	1
GSE17721_LPS_VS_PAM3CSK4_12H_BMDM_	-0,074	0,044	0,02146	1
GCACCTT,MIR-18A,MIR-18B	-0,074	0,045	0,00152	1
REACTOME_SHC_MEDIATED_CASCADE	-0,074	0,045	0,00166	1
REACTOME_METABOLISM_OF_AMINO_ACIDS_	-0,074	0,045	0,12982	1
YAO_HOXA10_TARGETS_VIA_PROGESTERONE	-0,074	0,045	0,00912	1
RGAGGAARY_V$PU1_Q6	-0,074	0,045	0,0038	1
KEGG_AMINOACYL_TRNA_BIOSYNTHESIS	-0,074	0,045	0,75311	1
GSE3982_CENT_MEMORY_CD4_TCELL_VS_N	-0,074	0,045	0,01458	1
ZHENG_BOUND_BY_FOXP3	-0,074	0,045	0,01809	1
chr1q25	-0,074	0,045	0,02985	1
REACTOME_PYRIMIDINE_METABOLISM	-0,074	0,045	0,10499	1
GSE2706_UNSTIM_VS_2H_R848_DC_DN	-0,074	0,045	0,0638	1
REACTOME_REMOVAL_OF_THE_FLAP_INTERM	-0,074	0,045	0,13965	1
REACTOME_INSULIN_RECEPTOR_SIGNALLING_	-0,074	0,045	0,00037	1
CYTOSKELETON_DEPENDENT_INTRACELLULAI	-0,073	0,045	0,23991	1
GSE3982_MAST_CELL_VS_NKCELL_DN	-0,073	0,045	0,00088	1
DAZARD_UV_RESPONSE_CLUSTER_G5	-0,073	0,045	0,53066	1
TONKS_TARGETS_OF_RUNX1_RUNX1T1_FUSI	-0,073	0,045	0,00901	1
V$USF_Q6	-0,073	0,045	0,00099	1
GSE29617_CTRL_VS_DAY7_TIV_FLU_VACCII	-0,073	0,045	0,01219	1
GSE11057_NAIVE_VS_MEMORY_CD4_TCELL_	-0,073	0,045	0,05785	1
GSE36476_CTRL_VS_TSST_ACT_40H_MEMO	-0,073	0,045	0,00214	1
GSE18791_CTRL_VS_NEWCASTLE_VIRUS_DC	-0,073	0,045	0,08766	1
BASSO_HAIRY_CELL_LEUKEMIA_UP	-0,073	0,045	0,03912	1
PID_S1P_S1P2_PATHWAY	-0,073	0,045	0,00212	1
WANG_RESPONSE_TO_PACLITAXEL_VIA_MAF	-0,073	0,045	0,00252	1
GSE11057_NAIVE_VS_CENT_MEMORY_CD4_	-0,073	0,045	0,05723	1
SIG_BCR_SIGNALING_PATHWAY	-0,073	0,045	0,0306	1
GSE360_DC_VS_MAC_UP	-0,073	0,045	0,02586	1
GENTILE_UV_RESPONSE_CLUSTER_D6	-0,073	0,045	0,02342	1

INTRAMOLECULAR_OXIDOREDUCTASE_ACTIVIT	-0,073	0,045	0,40643	1
HORIUCHI_WTAP_TARGETS_UP	-0,073	0,045	0,01663	1
PETRETTO_HEART_MASS_QTL_CIS_UP	-0,073	0,045	0,00235	1
YAO_TEMPORAL_RESPONSE_TO_PROGESTERC	-0,073	0,045	0,03589	1
MCBRYAN_PUBERTAL_BREAST_6_7WK_UP	-0,073	0,045	0,0188	1
GLUTAMATE_RECEPTOR_ACTIVITY	-0,073	0,045	0,00032	1
CHAUHAN_RESPONSE_TO_METHOXYESTRADIC	-0,073	0,045	0,06018	1
GSE39820_IL1B_IL6_VS_IL1B_IL6_IL23A_TF	-0,073	0,046	0,01476	1
CALCIUM_ION_BINDING	-0,073	0,046	0,01617	1
GNF2_MMP11	-0,073	0,046	0,00802	1
REACTOME_INTRINSIC_PATHWAY_FOR_APOPT	-0,073	0,046	0,02179	1
GSE3982_DC_VS_MAC_LPS_STIM_DN	-0,073	0,046	0,06852	1
ROSS_LEUKEMIA_WITH_MLL_FUSIONS	-0,073	0,046	0,01302	1
GSE7460_CTRL_VS_FOXP3_OVEREXPR_TCON	-0,073	0,046	0,00337	1
WANG_RESPONSE_TO_BEXAROTENE_DN	-0,073	0,046	0,15249	1
BOYLAN_MULTIPLE_MYELOMA_D_DN	-0,073	0,046	0,05261	1
ACTAYRNNNCCCR_UNKNOWN	-0,073	0,046	0,00461	1
RATTENBACHER_BOUND_BY_CELF1	-0,073	0,046	0,00529	1
GSE22886_NAIVE_CD8_TCELL_VS_NKCELL_	-0,073	0,046	0,06383	1
GARGALOVIC_RESPONSE_TO_OXIDIZED_PHOS	-0,073	0,046	0,00564	1
BIOCARTA_VDR_PATHWAY	0,073	0,046	0,07188	1
KEGG_PHOSPHATIDYLINOSITOL_SIGNALING_S	-0,073	0,046	0,00551	1
GILDEA_METASTASIS	-0,073	0,046	0,01846	1
LAIHO_COLORECTAL_CANCER_SERRATED_UP	-0,073	0,046	0,1617	1
BIOCARTA_EIF4_PATHWAY	-0,073	0,046	0,02696	1
FLOTHO_PEDIATRIC_ALL_THERAPY_RESPONS	-0,073	0,046	0,00234	1
BOYAULT_LIVER_CANCER_SUBCLASS_G56_D	-0,073	0,046	0,04347	1
KEGG_FRUCTOSE_AND_MANNOSE_METABOLIS	0,073	0,046	0,00659	1
CELLULAR_CARBOHYDRATE_METABOLIC_PRO	-0,073	0,046	0,06222	1
KAECH_NAIVE_VS_MEMORY_CD8_TCELL_UP	-0,073	0,046	0,00527	1
MAINTENANCE_OF_CELLULAR_LOCALIZATION	-0,073	0,047	0,20256	1
GSE17721_0.5H_VS_24H_GARDIQUIMOD_BM	-0,073	0,047	0,03044	1
IGLESIAS_E2F_TARGETS_DN	-0,073	0,047	0,00792	1
MORF_GMPS	-0,073	0,047	0,88202	1
V$ELF1_Q6	-0,073	0,047	0,01688	1
GSE7460_CTRL_VS_TGFB_TREATED_ACT_FC	-0,073	0,047	0,00227	1
PIEPOLI_LGI1_TARGETS_UP	-0,073	0,047	0,00984	1
MODULE_182	-0,073	0,047	0,09111	1
GOLDRATH_IMMUNE_MEMORY	-0,073	0,047	0,01516	1
GSE3982_MAC_VS_NEUTROPHIL_DN	-0,073	0,047	0,01673	1
ST_GA12_PATHWAY	-0,073	0,047	0,01843	1
REACTOME_TRANSMEMBRANE_TRANSPORT_C	-0,073	0,047	0,01999	1

RESPONSE_TO_TEMPERATURE_STIMULUS	0,073	0,047	0,00635	1
SCHAEFFER_PROSTATE_DEVELOPMENT_AND_	-0,073	0,047	0,08898	1
GSE13411_NAIVE_VS_SWITCHED_MEMORY_E	-0,073	0,047	0,02495	1
GAVIN_FOXP3_TARGETS_CLUSTER_P3	-0,073	0,047	0,01646	1
RAMPON_ENRICHED_LEARNING_ENVIRONMENT	-0,073	0,047	0,29068	1
GSE17974_0H_VS_24H_IN_VITRO_ACT_CD4	-0,073	0,047	0,00017	1
NEUTRAL_AMINO_ACID_TRANSMEMBRANE_TF	0,073	0,047	0,02776	1
DIAZ_CHRONIC_MEYLOGENOUS_LEUKEMIA_DN	-0,073	0,047	0,01588	1
chr10q24	-0,073	0,047	0,0264	1
BIOCARTA_AKAP13_PATHWAY	-0,073	0,047	0,17918	1
KEGG_DRUG_METABOLISM_OTHER_ENZYMES	0,073	0,047	0,04451	1
AMIT_EGF_RESPONSE_40_HELA	-0,073	0,047	0,00212	1
YOSHIOKA_LIVER_CANCER_EARLY_RECURREN	-0,073	0,047	0,1417	1
chr1q	-0,073	0,047	0,14136	1
REACTOME_SIGNALING_BY_FGFR_MUTANTS	-0,073	0,047	0,00525	1
TTGGGAG,MIR-150	-0,073	0,047	0,00027	1
JOHNSTONE_PARVB_TARGETS_1_DN	-0,073	0,047	0,05335	1
GSE27786_ERYTHROBLAST_VS_NEUTROPHIL_	-0,073	0,048	0,06452	1
LEUKOCYTE_CHEMOTAXIS	-0,073	0,048	0,10562	1
MODULE_113	-0,073	0,048	0,00147	1
FARMER_BREAST_CANCER_CLUSTER_5	-0,073	0,048	0,00797	1
GALINDO_IMMUNE_RESPONSE_TO_ENTEROTO	-0,073	0,048	0,09024	1
V$PAX5_02	-0,073	0,048	0,01282	1
V$MEF2_04	-0,073	0,048	0,01059	1
CENTROSOME_CYCLE	-0,073	0,048	0,12083	1
GRAHAM_CML_QUIESCENT_VS_CML_DIVIDING	-0,072	0,048	0,09223	1
chr8p23	-0,072	0,048	0,00572	1
REACTOME_SIGNALING_BY_ACTIVATED_POIN	-0,072	0,048	0,05601	1
BASOLATERAL_PLASMA_MEMBRANE	-0,072	0,048	0,031	1
BIOCARTA_MTOR_PATHWAY	-0,072	0,048	0,02481	1
ZHANG_ANTIVIRAL_RESPONSE_TO_RIBAVIRIN	-0,072	0,048	0,06516	1
BUSA_SAM68_TARGETS_DN	0,072	0,048	0,52598	1
GNF2_ZAP70	-0,072	0,048	0,02048	1
EXCRETION	-0,072	0,048	0,04604	1
REACTOME_MITOCHONDRIAL_TRNA_AMINOAC	-0,072	0,048	0,32938	1
PID_BETACATENIN_DEG_PATHWAY	0,072	0,048	0,28494	1
BERTUCCI_MEDULLARY_VS_DUCTAL_BREAST_	-0,072	0,048	0,17384	1
PID_TCRCALCIUMPATHWAY	-0,072	0,049	0,06544	1
T_CELL_ACTIVATION	-0,072	0,049	0,06291	1
REACTOME_ADP_SIGNALLING_THROUGH_P2R	-0,072	0,049	0,06578	1
WEBER_METHYLATED_ICP_IN_SPERM_DN	-0,072	0,049	0,84255	1
GSE3982_BCELL_VS_NKCELL_DN	-0,072	0,049	0,00597	1

GSE37416_0H_VS_3H_F_TULARENSIS_LVS_	-0,072	0,049	0,4219	1
GSE7764_NKCELL_VS_SPLENOCYTE_DN	-0,072	0,049	0,03479	1
SERVITJA_ISLET_HNF1A_TARGETS_DN	-0,072	0,049	0,00022	1
PLASARI_NFIC_TARGETS_BASAL_DN	-0,072	0,049	0,00411	1
YAO_TEMPORAL_RESPONSE_TO_PROGESTERO	-0,072	0,049	0,00222	1
GSE14000_UNSTIM_VS_16H_LPS_DC_TRANS	-0,072	0,049	0,18002	1
MALONEY_RESPONSE_TO_17AAG_UP	-0,072	0,049	0,00218	1
WHITFIELD_CELL_CYCLE_M_G1	-0,072	0,049	0,11295	1
GCM_BNIP1	-0,072	0,049	0,00497	1
ACTGCCT,MIR-34B	-0,072	0,05	0,00356	1
GSE7460_FOXP3_MUT_VS_WT_ACT_WITH_T	-0,072	0,05	0,07519	1
BIOCARTA_BARRESTIN_SRC_PATHWAY	-0,072	0,05	0,02327	1
DING_LUNG_CANCER_MUTATED_FREQUENTLY	-0,072	0,05	0,00088	1
VIRAL_GENOME_REPLICATION	-0,072	0,05	0,48999	1
TIMOFEEVA_GROWTH_STRESS_VIA_STAT1_D	-0,072	0,05	0,10151	1
ZHAN_V2_LATE_DIFFERENTIATION_GENES	-0,072	0,05	0,24713	1
UBIQUITIN_BINDING	-0,072	0,05	0,09497	1
SEMENZA_HIF1_TARGETS	-0,072	0,05	0,38544	1
RESPONSE_TO_WOUNDING	-0,072	0,05	0,04231	1
TRANSPORT	-0,072	0,05	0,00091	1
ER_TO_GOLGI_VESICLE_MEDIATED_TRANSPO	0,072	0,05	0,02579	1
GSE27786_CD4_TCELL_VS_ERYTHTROBLAST	-0,072	0,05	0,01212	1
MICROVILLUS	-0,072	0,05	0,00942	1
PID_PS1PATHWAY	-0,072	0,05	0,00488	1
YANG_MUC2_TARGETS_DUODENUM_6MO_UP	-0,072	0,05	0,16005	1
GSE17721_LPS_VS_POLYIC_0.5H_BMDM_DN	-0,072	0,05	0,00063	1
PHOSPHOLIPID_BINDING	-0,072	0,05	0,00091	1
TSENG_ADIPOGENIC_POTENTIAL_DN	-0,072	0,05	0,00409	1
BIOCARTA_FMLP_PATHWAY	-0,072	0,05	0,08612	1
CHEMICAL_HOMEOSTASIS	-0,072	0,05	0,01756	1
BIOCARTA_ARENRF2_PATHWAY	-0,072	0,051	0,02411	1
ONO_FOXP3_TARGETS_DN	-0,072	0,051	0,05081	1
RAMJAUN_APOPTOSIS_BY_TGFB1_VIA_SMAD	-0,072	0,051	0,01441	1
GSE10239_MEMORY_VS_KLRG1HIGH_EFF_CD	-0,072	0,051	0,41118	1
CAGCACT,MIR-512-3P	-0,072	0,051	0,00692	1
MCBRYAN_PUBERTAL_BREAST_5_6WK_DN	-0,072	0,051	0,0273	1
MODULE_358	-0,072	0,051	0,01883	1
MORPHOGENESIS_OF_AN_EPITHELIUM	-0,072	0,051	0,2396	1
MARZEC_IL2_SIGNALING_UP	-0,072	0,051	0,11831	1
HAHTOLA_SEZARY_SYNDROM_UP	-0,072	0,051	0,17225	1
ZHAN_MULTIPLE_MYELOMA_CD1_AND_CD2_	-0,072	0,051	0,02566	1
MODULE_107	-0,072	0,051	0,06438	1

BUSA_SAM68_TARGETS_UP	-0,072	0,051	0,00088	1
FARMER_BREAST_CANCER_CLUSTER_1	-0,071	0,051	0,07543	1
BRUNEAU_SEPTATION_ATRIAL	-0,071	0,051	0,11644	1
THIOLESTER_HYDROLASE_ACTIVITY	-0,071	0,051	0,58739	1
TARTE_PLASMA_CELL_VS_PLASMABLAST_UI	-0,071	0,051	0,01271	1
PID_AP1_PATHWAY	-0,071	0,051	0,00784	1
RHO_PROTEIN_SIGNAL_TRANSDUCTION	-0,071	0,051	0,00653	1
V$NKX25_01	-0,071	0,051	0,00892	1
GSE7460_TCONV_VS_TREG_THYMUS_DN	-0,071	0,051	0,04328	1
GSE17974_CTRL_VS_ACT_IL4_AND_ANTI_IL	-0,071	0,052	0,00237	1
GSE37416_CTRL_VS_48H_F_TULARENSIS_L'	0,071	0,052	0,00652	1
ATGTACA,MIR-493	-0,071	0,052	0,00539	1
GSE14000_UNSTIM_VS_4H_LPS_DC_TRANSL	-0,071	0,052	0,0048	1
VILIMAS_NOTCH1_TARGETS_UP	-0,071	0,052	0,03086	1
GSE15930_NAIVE_VS_24H_IN_VITRO_STIM_	-0,071	0,052	0,00137	1
ATF2_UP.V1_DN	-0,071	0,052	0,00719	1
BOYLAN_MULTIPLE_MYELOMA_C_DN	-0,071	0,052	0,02402	1
GTGCAAA,MIR-507	-0,071	0,052	0,00304	1
NIELSEN_SCHWANNOMA_UP	-0,071	0,052	0,00713	1
FOSTER_KDM1A_TARGETS_DN	-0,071	0,052	0,0153	1
GSE2706_UNSTIM_VS_2H_LPS_AND_R848_I	-0,071	0,052	0,08989	1
SECRETIN_LIKE_RECEPTOR_ACTIVITY	-0,071	0,052	0,0004	1
GSE17721_CTRL_VS_POLYIC_1H_BMDM_DN	-0,071	0,052	0,00578	1
GSE27786_BCELL_VS_NKTCELL_UP	-0,071	0,052	0,05088	1
GAGACTG,MIR-452	-0,071	0,052	0,05822	1
MARCHINI_TRABECTEDIN_RESISTANCE_UP	0,071	0,052	0,2121	1
MODULE_417	0,071	0,052	0,49406	1
LIPID_RAFT	-0,071	0,052	0,06529	1
MARTINEZ_RESPONSE_TO_TRABECTEDIN_UP	-0,071	0,052	0,14353	1
CORRE_MULTIPLE_MYELOMA_DN	-0,071	0,052	0,00287	1
GNF2_MATK	-0,071	0,053	0,02677	1
WILSON_PROTEASES_AT_TUMOR_BONE_INTE	-0,071	0,053	0,23246	1
GNF2_DDX5	-0,071	0,053	0,00431	1
chr11p12	-0,071	0,053	0,02118	1
JOHANSSON_BRAIN_CANCER_EARLY_VS_LAT	-0,071	0,053	0,00356	1
HU_GENOTOXIN_ACTION_DIRECT_VS_INDIREC	-0,071	0,053	0,11159	1
GCM_BAG5	0,071	0,053	0,10463	1
GSE24634_TEFF_VS_TCONV_DAY5_IN_CULT	-0,071	0,053	0,00262	1
GSE13738_RESTING_VS_TCR_ACTIVATED_CI	-0,071	0,053	0,0017	1
POSITIVE_REGULATION_OF_SIGNAL_TRANSDU	-0,071	0,053	0,00774	1
DIRMEIER_LMP1_RESPONSE_LATE_UP	-0,071	0,053	0,17685	1
RAGHAVACHARI_PLATELET_SPECIFIC_GENES	-0,071	0,053	0,09845	1

GSE14000_TRANSLATED_RNA_VS_MRNA_DC	-0,071	0,053	0,01639	1
GSE7460_CTRL_VS_FOXP3_OVEREXPR_TCON	-0,071	0,053	0,00503	1
ZHAN_LATE_DIFFERENTIATION_GENES_UP	-0,071	0,053	0,00546	1
V$IRF1_01	-0,071	0,053	0,01098	1
CHEN_HOXA5_TARGETS_6HR_DN	-0,071	0,053	0,32258	1
TRANSLATIONAL_INITIATION	-0,071	0,053	0,30708	1
KIM_MYCN_AMPLIFICATION_TARGETS_DN	-0,071	0,053	0,05942	1
URS_ADIPOCYTE_DIFFERENTIATION_UP	-0,071	0,053	0,00243	1
chr8q22	-0,071	0,053	0,20873	1
GRADE_COLON_CANCER_DN	-0,071	0,053	0,00943	1
GSE360_L_MAJOR_VS_M_TUBERCULOSIS_DC	-0,071	0,053	0,01894	1
GSE15215_CD2_POS_VS_NEG_PDC_DN	-0,071	0,053	0,13337	1
NAKAMURA_TUMOR_ZONE_PERIPHERAL_VS_(	-0,071	0,054	0,02379	1
chr9p11	-0,071	0,054	0,17368	1
FINETTI_BREAST_CANCER_KINOME_GREEN	-0,071	0,054	0,06587	1
SERINE_HYDROLASE_ACTIVITY	-0,071	0,054	0,00194	1
GSE7852_LN_VS_FAT_TCONV_DN	-0,071	0,054	0,00629	1
ACEVEDO_LIVER_CANCER_UP	-0,071	0,054	0,10149	1
GSE3337_CTRL_VS_16H_IFNG_IN_CD8POS_I	-0,071	0,054	0,06284	1
KIM_PTEN_TARGETS_UP	-0,071	0,054	0,05511	1
EGFR_UP.V1_UP	-0,071	0,054	0,18638	1
BIOCARTA_MITOCHONDRIA_PATHWAY	-0,071	0,054	0,02591	1
chr18p	0,071	0,054	0,26099	1
GSE8678_IL7R_LOW_VS_HIGH_EFF_CD8_TCI	-0,071	0,054	0,00711	1
INTERFERON_GAMMA_PRODUCTION	-0,07	0,054	0,10394	1
ENDOMEMBRANE_SYSTEM	-0,07	0,054	0,27479	1
KYNG_RESPONSE_TO_H2O2	-0,07	0,054	0,03563	1
MODULE_119	-0,07	0,055	0,03311	1
GSE339_CD4POS_VS_CD8POS_DC_UP	-0,07	0,055	0,03267	1
MORF_ATRX	-0,07	0,055	0,00088	1
KEGG_ENDOCYTOSIS	-0,07	0,055	0,00125	1
REACTOME_ENOS_ACTIVATION_AND_REGULA	0,07	0,055	0,19591	1
GSE10325_BCELL_VS_LUPUS_BCELL_DN	-0,07	0,055	0,2725	1
OXFORD_RALA_OR_RALB_TARGETS_DN	-0,07	0,055	0,00845	1
LIN_MELANOMA_COPY_NUMBER_UP	-0,07	0,055	0,00288	1
GSE17721_CTRL_VS_GARDIQUIMOD_8H_BMD	-0,07	0,055	0,14291	1
chrxq27	-0,07	0,055	0,1722	1
YAO_TEMPORAL_RESPONSE_TO_PROGESTER(	-0,07	0,055	0,15922	1
BIOCARTA_CALCINEURIN_PATHWAY	-0,07	0,055	0,01559	1
REACTOME_NA_CL_DEPENDENT_NEUROTRAN	-0,07	0,055	0,74489	1
TAGCTTT,MIR-9	-0,07	0,055	0,00523	1
MODULE_426	-0,07	0,055	0,0249	1

CACTTTG,MIR-520G,MIR-520H	-0,07	0,055	0,01999	1
GSE17974_CTRL_VS_ACT_IL4_AND_ANTI_IL	-0,07	0,055	0,00313	1
LYMPHOCYTE_DIFFERENTIATION	-0,07	0,055	0,05502	1
HELICASE_ACTIVITY	-0,07	0,055	0,2972	1
HOFFMANN_IMMATURE_TO_MATURE_B_LYMP	-0,07	0,055	0,01099	1
HOLLEMAN_VINCRISTINE_RESISTANCE_B_ALL	-0,07	0,055	0,0555	1
BOYLAN_MULTIPLE_MYELOMA_PCA1_DN	0,07	0,055	0,01531	1
WONG_PROTEASOME_GENE_MODULE	-0,07	0,055	0,96705	1
OXIDOREDUCTASE_ACTIVITY_ACTING_ON_TH	0,07	0,056	0,32324	1
GSE6269_FLU_VS_STREP_AUREUS_INF_PBM(	-0,07	0,056	0,00122	1
PID_ALK1PATHWAY	-0,07	0,056	0,00591	1
DAZARD_UV_RESPONSE_CLUSTER_G28	-0,07	0,056	0,17254	1
WOOD_EBV_EBNA1_TARGETS_DN	-0,07	0,056	0,07324	1
KIM_HYPOXIA	-0,07	0,056	0,2476	1
GSE3982_NEUTROPHIL_VS_TH2_UP	-0,07	0,056	0,01623	1
LABBE_TARGETS_OF_TGFB1_AND_WNT3A_[	-0,07	0,056	0,05063	1
IMMUNOLOGICAL_SYNAPSE	-0,07	0,056	0,04369	1
GNF2_PTPN4	-0,07	0,056	0,02851	1
LEE_LIVER_CANCER_CIPROFIBRATE_UP	-0,07	0,056	0,01806	1
chrxq21	-0,07	0,056	0,00252	1
TRANSMEMBRANE_RECEPTOR_PROTEIN_PHOS	-0,07	0,056	0,00446	1
GSE3982_EOSINOPHIL_VS_MAST_CELL_UP	-0,07	0,056	0,01625	1
EXTRACELLULAR_STRUCTURE_ORGANIZATION	-0,07	0,056	0,12632	1
YIH_RESPONSE_TO_ARSENITE_C1	-0,07	0,057	0,12632	1
LUI_THYROID_CANCER_PAX8_PPARG_UP	-0,07	0,057	0,03784	1
OXYGEN_BINDING	0,07	0,057	0,46537	1
VASCULATURE_DEVELOPMENT	-0,07	0,057	0,04125	1
ZHENG_IL22_SIGNALING_UP	-0,07	0,057	0,04212	1
RYCACNNRNNRNCAG_UNKNOWN	-0,07	0,057	0,00652	1
GSE3982_EOSINOPHIL_VS_TH1_UP	-0,07	0,057	0,01857	1
ATM_DN.V1_DN	-0,07	0,057	0,02118	1
YAGUE_PRETUMOR_DRUG_RESISTANCE_DN	-0,07	0,057	0,03622	1
BROWNE_HCMV_INFECTION_2HR_UP	-0,07	0,057	0,00038	1
GUANYL_NUCLEOTIDE_BINDING	-0,07	0,057	0,0113	1
GENTILE_UV_LOW_DOSE_DN	-0,07	0,057	0,05073	1
GSE3982_MEMORY_CD4_TCELL_VS_TH2_UP	-0,07	0,057	0,00239	1
chr8q11	-0,07	0,057	0,06963	1
CYTOPLASMIC_MEMBRANE_BOUND_VESICLE	-0,07	0,057	0,00547	1
PETRETTO_LEFT_VENTRICLE_MASS_QTL_CIS_	-0,07	0,057	0,13015	1
RNA_POLYMERASE_ACTIVITY	-0,07	0,057	0,1784	1
SHIPP_DLBCL_VS_FOLLICULAR_LYMPHOMA_[	-0,07	0,057	0,00299	1
DEFENSE_RESPONSE_TO_BACTERIUM	-0,07	0,057	0,0368	1

GSE12845_NAIVE_VS_PRE_GC_TONSIL_BCEL	-0,07	0,058	0,0002	1
GINESTIER_BREAST_CANCER_20Q13_AMPLIFI	0,07	0,058	0,90664	1
FARMER_BREAST_CANCER_APOCRINE_VS_BA	-0,07	0,058	0,00928	1
WONG_ENDMETRIUM_CANCER_UP	-0,07	0,058	0,18573	1
GSE3337_4H_VS_16H_IFNG_IN_CD8POS_DC	-0,07	0,058	0,26686	1
SERINE_TYPE_ENDOPEPTIDASE_ACTIVITY	-0,07	0,058	0,00148	1
BOYLAN_MULTIPLE_MYELOMA_PCA3_UP	-0,07	0,058	0,00357	1
chr6p25	-0,07	0,058	0,19167	1
SYNAPSE_ORGANIZATION_AND_BIOGENESIS	-0,069	0,058	0,2507	1
NIELSEN_SYNOVIAL_SARCOMA_UP	-0,069	0,058	0,2631	1
POSITIVE_REGULATION_OF_TRANSFERASE_A(	-0,069	0,058	0,01835	1
SANA_TNF_SIGNALING_UP	-0,069	0,058	0,18514	1
MARZEC_IL2_SIGNALING_DN	-0,069	0,058	0,02554	1
KEGG_ETHER_LIPID_METABOLISM	-0,069	0,058	0,0095	1
MILI_PSEUDOPODIA_CHEMOTAXIS_UP	-0,069	0,058	0,10527	1
chr6q26	-0,069	0,058	0,0603	1
GSE37416_CTRL_VS_24H_F_TULARENSIS_L\	-0,069	0,058	0,1802	1
IL15_UP.V1_DN	-0,069	0,059	0,00028	1
MCBRYAN_PUBERTAL_BREAST_5_6WK_UP	-0,069	0,059	0,01111	1
PDGF_UP.V1_DN	-0,069	0,059	0,00471	1
GSE29618_BCELL_VS_PDC_DAY7_FLU_VAC(	-0,069	0,059	0,02346	1
GSE3982_EFF_MEMORY_CD4_TCELL_VS_NK(	-0,069	0,059	0,02497	1
LIPID_HOMEOSTASIS	-0,069	0,059	0,02665	1
REACTOME_ION_CHANNEL_TRANSPORT	-0,069	0,059	0,05839	1
WUNDER_INFLAMMATORY_RESPONSE_AND_C	-0,069	0,059	0,00488	1
GSE2706_R848_VS_LPS_8H_STIM_DC_DN	-0,069	0,059	0,08679	1
V$EVI1_01	0,069	0,059	0,55915	1
CATION_HOMEOSTASIS	-0,069	0,059	0,02375	1
REACTOME_GPVI_MEDIATED_ACTIVATION_CA	-0,069	0,059	0,01913	1
GSE27786_LIN_NEG_VS_MONO_MAC_DN	-0,069	0,059	0,11152	1
REACTOME_CDT1_ASSOCIATION_WITH_THE_	-0,069	0,059	0,85332	1
V$HNF4_01	-0,069	0,059	0,00052	1
GSE339_CD4POS_VS_CD4CD8DN_DC_UP	-0,069	0,059	0,04729	1
WIERENGA_STAT5A_TARGETS_GROUP2	-0,069	0,059	0,09944	1
GSE6269_STREP_AUREUS_VS_STREP_PNEUM	-0,069	0,059	0,00301	1
MODULE_46	-0,069	0,06	0,03436	1
SMID_BREAST_CANCER_BASAL_DN	0,069	0,06	0,02983	1
BURTON_ADIPOGENESIS_11	-0,069	0,06	0,03411	1
GSE7460_CD8_TCELL_VS_TREG_ACT_UP	-0,069	0,06	0,00202	1
REACTOME_PROCESSIVE_SYNTHESIS_ON_THE	-0,069	0,06	0,18562	1
KEGG_MAPK_SIGNALING_PATHWAY	-0,069	0,06	0,00091	1
IGARASHI_ATF4_TARGETS_UP	-0,069	0,06	0,01518	1

REACTOME_REVERSIBLE_HYDRATION_OF_CAI	-0,069	0,06	0,05933	1
MODULE_44	-0,069	0,06	0,02709	1
GCM_MSN	-0,069	0,06	0,15888	1
GSE9988_LOW_LPS_VS_ANTI_TREM1_AND_	-0,069	0,06	0,08697	1
PID_ANGIOPOIETINRECEPTOR_PATHWAY	-0,069	0,06	0,02078	1
PARK_HSC_AND_MULTIPOTENT_PROGENITOR:	-0,069	0,06	0,01396	1
GSE7460_CD8_TCELL_VS_CD4_TCELL_ACT_	-0,069	0,06	0,03084	1
CHEOK_RESPONSE_TO_MERCAPTOPURINE_AN	-0,069	0,06	0,59203	1
ACTIN_BINDING	-0,069	0,06	0,02908	1
KEGG_BASE_EXCISION_REPAIR	-0,069	0,06	0,02806	1
MIKKELSEN_IPS_WITH_HCP_H3K27ME3	-0,069	0,06	0,00757	1
GENTLES_LEUKEMIC_STEM_CELL_UP	-0,069	0,061	0,00712	1
BIOCARTA_NOS1_PATHWAY	-0,069	0,061	0,05456	1
MITOCHONDRIAL_RESPIRATORY_CHAIN_COMP	0,069	0,061	0,06141	1
NADH_DEHYDROGENASE_COMPLEX	0,069	0,061	0,06141	1
RESPIRATORY_CHAIN_COMPLEX_I	0,069	0,061	0,06141	1
GSE7852_LN_VS_THYMUS_TREG_UP	-0,069	0,061	0,01379	1
INTERCALATED_DISC	0,069	0,061	0,03178	1
KYNG_DNA_DAMAGE_BY_UV	-0,069	0,061	0,09274	1
GSE9988_ANTI_TREM1_AND_LPS_VS_CTRL_	-0,069	0,061	0,02453	1
GSE17721_CTRL_VS_GARDIQUIMOD_12H_BM	-0,069	0,061	0,00737	1
WATANABE_COLON_CANCER_MSI_VS_MSS_I	-0,069	0,061	0,01319	1
chr2q32	-0,069	0,061	0,00867	1
PID_ARF6_PATHWAY	-0,069	0,061	0,0044	1
chr2p12	-0,069	0,061	0,13435	1
RANKIN_ANGIOGENIC_TARGETS_OF_VHL_HIF	0,069	0,062	0,50574	1
GSE20715_0H_VS_24H_OZONE_LUNG_UP	-0,069	0,062	0,00769	1
GGAANCGGAANY_UNKNOWN	-0,068	0,062	0,05841	1
chr13q14	-0,068	0,062	0,03536	1
GSE20366_CD103_POS_VS_CD103_KLRG1_	-0,068	0,062	0,00229	1
TAKAO_RESPONSE_TO_UVB_RADIATION_UP	-0,068	0,062	0,13691	1
BCAT_GDS748_UP	-0,068	0,062	0,00243	1
REACTOME_RNA_POL_III_TRANSCRIPTION_INI	-0,068	0,062	0,13085	1
ION_HOMEOSTASIS	-0,068	0,062	0,02921	1
CHANGOLKAR_H2AFY_TARGETS_UP	-0,068	0,062	0,06653	1
MARIADASON_RESPONSE_TO_BUTYRATE_SUL	-0,068	0,062	0,0011	1
WANG_METASTASIS_OF_BREAST_CANCER_E	0,068	0,062	0,6361	1
KYNG_WERNER_SYNDROM_DN	-0,068	0,062	0,01095	1
GSE13485_DAY1_VS_DAY3_YF17D_VACCINI	-0,068	0,062	0,00242	1
INFLAMMATORY_RESPONSE	-0,068	0,062	0,1104	1
MURAKAMI_UV_RESPONSE_1HR_DN	-0,068	0,062	0,03322	1
LEE_LIVER_CANCER_CIPROFIBRATE_DN	-0,068	0,063	0,01331	1

MITOCHONDRIAL_MEMBRANE_ORGANIZATION_	-0,068	0,063	0,04102	1
GARGALOVIC_RESPONSE_TO_OXIDIZED_PHOS	-0,068	0,063	0,01253	1
PLASARI_NFIC_TARGETS_BASAL_UP	-0,068	0,063	0,00026	1
ATF2_S_UP.V1_DN	-0,068	0,063	0,00417	1
chr12q14	-0,068	0,063	0,00463	1
REACTOME_PKB_MEDIATED_EVENTS	-0,068	0,063	0,01212	1
SEMBA_FHIT_TARGETS_UP	-0,068	0,063	0,22492	1
GSE14000_UNSTIM_VS_16H_LPS_DC_DN	-0,068	0,063	0,21403	1
GSE13485_CTRL_VS_DAY1_YF17D_VACCINE	-0,068	0,063	0,00028	1
LIPASE_ACTIVITY	-0,068	0,063	0,05162	1
BOSCO_EPITHELIAL_DIFFERENTIATION_MODUI	-0,068	0,063	0,83877	1
MODULE_573	-0,068	0,063	0,17227	1
JI_CARCINOGENESIS_BY_KRAS_AND_STK11_	-0,068	0,063	0,02086	1
GSE17580_TREG_VS_TEFF_DN	-0,068	0,063	0,04019	1
PID_IL8CXCR2_PATHWAY	-0,068	0,063	0,08179	1
GSE17721_POLYIC_VS_GARDIQUIMOD_8H_BI	-0,068	0,063	0,00458	1
BIOCARTA_HDAC_PATHWAY	-0,068	0,063	0,00404	1
MARTENS_BOUND_BY_PML_RARA_FUSION	-0,068	0,064	0,00504	1
TUOMISTO_TUMOR_SUPPRESSION_BY_COL13,	-0,068	0,064	0,0433	1
BILANGES_RAPAMYCIN_SENSITIVE_VIA_TSC1_	-0,068	0,064	0,18529	1
SINGLE_STRANDED_RNA_BINDING	-0,068	0,064	0,04133	1
GSE360_CTRL_VS_L_MAJOR_MAC_UP	-0,068	0,064	0,01465	1
TAKEDA_TARGETS_OF_NUP98_HOXA9_FUSIC	-0,068	0,064	0,13085	1
AMINE_BINDING	-0,068	0,064	0,0703	1
MORF_PAPSS1	-0,068	0,064	0,83289	1
REACTOME_CTNNB1_PHOSPHORYLATION_CA!	0,068	0,064	0,08863	1
GSE17974_IL4_AND_ANTI_IL12_VS_UNTREA	-0,068	0,064	0,05299	1
CARBOHYDRATE_METABOLIC_PROCESS	-0,068	0,064	0,03808	1
GSE22886_NAIVE_VS_MEMORY_TCELL_DN	-0,068	0,064	0,04674	1
YAO_TEMPORAL_RESPONSE_TO_PROGESTER(	-0,068	0,064	0,07424	1
POSITIVE_REGULATION_OF_TRANSLATION	-0,068	0,064	0,0688	1
CARBOXY_LYASE_ACTIVITY	0,068	0,064	0,09572	1
PID_BCR_5PATHWAY	-0,068	0,064	0,05957	1
chr4q13	-0,068	0,064	0,08356	1
GSE18791_CTRL_VS_NEWCASTLE_VIRUS_DC	-0,068	0,064	0,16746	1
GSE17974_CTRL_VS_ACT_IL4_AND_ANTI_IL	-0,068	0,065	0,00019	1
PROTEIN_PHOSPHATASE_TYPE_2A_REGULAT(	-0,068	0,065	0,38468	1
YAMANAKA_GLIOBLASTOMA_SURVIVAL_UP	-0,068	0,065	0,32726	1
SMALL_GTPASE_MEDIATED_SIGNAL_TRANSDI	-0,068	0,065	0,00159	1
REACTOME_THROMBIN_SIGNALLING_THROUGI	-0,068	0,065	0,0044	1
AKL_HTLV1_INFECTION_UP	-0,068	0,065	0,67572	1
SERINE_TYPE_PEPTIDASE_ACTIVITY	-0,068	0,065	0,00178	1

LIU_IL13_MEMORY_MODEL_UP	-0,068	0,065	0,10716	1
LEONARD_HYPOXIA	-0,068	0,065	0,04726	1
EIF4E_DN	-0,068	0,065	0,12242	1
INTRACELLULAR_PROTEIN_TRANSPORT	-0,068	0,065	0,28736	1
V$SREBP1_01	-0,068	0,065	0,00775	1
WAMUNYOKOLI_OVARIAN_CANCER_GRADES_	-0,068	0,065	0,00531	1
EMBRYONIC_MORPHOGENESIS	-0,068	0,065	0,02016	1
GSE17721_LPS_VS_CPG_0.5H_BMDM_DN	-0,068	0,065	0,00772	1
MAYBURD_RESPONSE_TO_L663536_UP	-0,068	0,065	0,02996	1
LI_WILMS_TUMOR_VS_FETAL_KIDNEY_2_DN	-0,068	0,065	0,0053	1
GSE360_CTRL_VS_L_DONOVANI_DC_UP	-0,068	0,065	0,01896	1
OHGUCHI_LIVER_HNF4A_TARGETS_DN	-0,068	0,065	0,00806	1
V$PAX3_01	-0,068	0,065	0,01598	1
GSE24026_PD1_LIGATION_VS_CTRL_IN_ACT	-0,068	0,065	0,0533	1
YIH_RESPONSE_TO_ARSENITE_C5	-0,068	0,065	0,1683	1
TESAR_JAK_TARGETS_MOUSE_ES_D3_DN	-0,068	0,065	0,02691	1
SANSOM_WNT_PATHWAY_REQUIRE_MYC	-0,068	0,066	0,00815	1
chr11p15	-0,067	0,066	0,00035	1
GSE20715_0H_VS_48H_OZONE_LUNG_UP	-0,067	0,066	0,00561	1
ST_TYPE_I_INTERFERON_PATHWAY	-0,067	0,066	0,22799	1
GSE27786_CD4_VS_CD8_TCELL_UP	-0,067	0,066	0,01564	1
REACTOME_STEROID_HORMONES	-0,067	0,066	0,01219	1
CTCNANGTGNY_UNKNOWN	-0,067	0,066	0,00771	1
GSE18791_UNSTIM_VS_NEWCATSLE_VIRUS_	-0,067	0,066	0,00013	1
GSE34205_HEALTHY_VS_FLU_INF_INFANT_F	-0,067	0,066	0,34742	1
LYSOSOME_ORGANIZATION_AND_BIOGENESIS	0,067	0,066	0,23857	1
MATTIOLI_MULTIPLE_MYELOMA_WITH_14Q32	-0,067	0,066	0,00145	1
DER_IFN_GAMMA_RESPONSE_UP	-0,067	0,066	0,20437	1
PID_SMAD2_3NUCLEARPATHWAY	-0,067	0,066	0,00046	1
GSE360_LOW_DOSE_B_MALAYI_VS_M_TUBE	-0,067	0,066	0,0042	1
MORF_EI24	-0,067	0,067	0,82642	1
GSE17721_CTRL_VS_CPG_0.5H_BMDM_UP	-0,067	0,067	0,08815	1
POSITIVE_REGULATION_OF_CYTOKINE_BIOSYI	-0,067	0,067	0,05741	1
GTPASE_REGULATOR_ACTIVITY	-0,067	0,067	0,00402	1
BIOCARTA_ALK_PATHWAY	-0,067	0,067	0,00571	1
CELLULAR_CATION_HOMEOSTASIS	-0,067	0,067	0,0268	1
LOPEZ_EPITHELIOID_MESOTHELIOMA	-0,067	0,067	0,00745	1
MODULE_97	-0,067	0,067	0,1202	1
VANDESLUIS_NORMAL_EMBRYOS_UP	-0,067	0,067	0,07797	1
MODULE_367	-0,067	0,067	0,01752	1
RAMPON_ENRICHED_LEARNING_ENVIRONMENT	-0,067	0,067	0,00304	1
V$AP1_Q6_01	-0,067	0,067	0,03607	1

V$FAC1_01	-0,067	0,067	0,00044	1
GREGORY_SYNTHETIC_LETHAL_WITH_IMATINI	-0,067	0,067	0,01107	1
PID_CD8TCRDOWNSTREAMPATHWAY	-0,067	0,067	0,08689	1
GSE17721_LPS_VS_POLYIC_12H_BMDM_DN	-0,067	0,067	0,0875	1
KEGG_FC_EPSILON_RI_SIGNALING_PATHWAY	-0,067	0,068	0,02026	1
MODULE_157	-0,067	0,068	0,03425	1
MODULE_516	-0,067	0,068	0,03199	1
HOSHIDA_LIVER_CANCER_LATE_RECURRENCE	-0,067	0,068	0,00392	1
GSE2706_UNSTIM_VS_2H_LPS_DC_DN	-0,067	0,068	0,11556	1
HOFMANN_MYELODYSPLASTIC_SYNDROM_RIS	-0,067	0,068	0,00833	1
SESTO_RESPONSE_TO_UV_C3	-0,067	0,068	0,35936	1
chr8q12	-0,067	0,068	0,08986	1
YTTCCNNNGGAMR_UNKNOWN	-0,067	0,068	0,00263	1
SMID_BREAST_CANCER_NORMAL_LIKE_DN	0,067	0,068	0,23221	1
MEMBRANE_BOUND_VESICLE	-0,067	0,068	0,00624	1
PROTEIN_DOMAIN_SPECIFIC_BINDING	-0,067	0,068	0,00015	1
GSE22886_NAIVE_CD4_TCELL_VS_NEUTROP	-0,067	0,068	0,14531	1
REACTOME_THE_ROLE_OF_NEF_IN_HIV1_REF	-0,067	0,068	0,10559	1
HERNANDEZ_ABERRANT_MITOSIS_BY_DOCETA	-0,067	0,068	0,02334	1
MODULE_261	-0,067	0,068	0,03392	1
JIANG_HYPOXIA_NORMAL	-0,067	0,068	0,02739	1
GSE17721_CTRL_VS_PAM3CSK4_6H_BMDM_	-0,067	0,068	0,00775	1
REACTOME_GAP_JUNCTION_TRAFFICKING	-0,067	0,069	0,78171	1
IIZUKA_LIVER_CANCER_EARLY_RECURRENCE	-0,067	0,069	0,04264	1
GSE17721_LPS_VS_PAM3CSK4_8H_BMDM_U	-0,067	0,069	0,01048	1
KEGG_HEMATOPOIETIC_CELL_LINEAGE	-0,067	0,069	0,0328	1
TURASHVILI_BREAST_LOBULAR_CARCINOMA_	-0,067	0,069	0,01263	1
PID_FRA_PATHWAY	-0,067	0,069	0,22897	1
REACTOME_N_GLYCAN_ANTENNAE_ELONGAT	-0,067	0,069	0,12503	1
GSE10239_MEMORY_VS_DAY4.5_EFF_CD8_T	-0,067	0,069	0,00102	1
WANG_LSD1_TARGETS_UP	-0,067	0,069	0,00988	1
GSE3337_CTRL_VS_4H_IFNG_IN_CD8POS_DC	-0,067	0,069	0,09275	1
MOREIRA_RESPONSE_TO_TSA_UP	-0,067	0,069	0,0685	1
GSE27786_LSK_VS_CD4_TCELL_DN	-0,067	0,069	0,04258	1
DACOSTA_LOW_DOSE_UV_RESPONSE_VIA_E	-0,067	0,069	0,09141	1
CAR_MLANA	-0,067	0,069	0,06771	1
LYSOSOMAL_MEMBRANE	0,067	0,069	0,06519	1
LEIN_NEURON_MARKERS	-0,067	0,069	0,00459	1
INTERLEUKIN_BINDING	-0,067	0,069	0,12441	1
ICHIBA_GRAFT_VERSUS_HOST_DISEASE_D7_I	-0,067	0,069	0,00059	1
GSE3982_MAST_CELL_VS_NEUTROPHIL_DN	-0,067	0,069	0,00974	1
MONOVALENT_INORGANIC_CATION_HOMEOST	0,066	0,07	0,90142	1

BIOCARTA_GLEEVEC_PATHWAY	-0,066	0,07	0,00843	1
chr8q23	-0,066	0,07	0,04902	1
GSE360_CTRL_VS_L_DONOVANI_MAC_DN	-0,066	0,07	0,08401	1
V$PAX5_01	-0,066	0,07	0,00036	1
chr12q21	-0,066	0,07	0,00954	1
REACTOME_G_PROTEIN_BETA_GAMMA_SIGNA	-0,066	0,07	0,03317	1
WAKABAYASHI_ADIPOGENESIS_PPARG_RXRA_	-0,066	0,07	0,00073	1
IVANOVA_HEMATOPOIESIS_LATE_PROGENITO	-0,066	0,07	0,14829	1
HYDROLASE_ACTIVITY_HYDROLYZING_N_GLY	-0,066	0,071	0,00727	1
GTTATAT,MIR-410	-0,066	0,071	0,03811	1
GSE17721_0.5H_VS_24H_CPG_BMDM_UP	-0,066	0,071	0,07032	1
EHRLICH_ICF_SYNDROM_UP	-0,066	0,071	0,03805	1
ST_GA13_PATHWAY	-0,066	0,071	0,09711	1
GSE17974_0.5H_VS_72H_UNTREATED_IN_V	-0,066	0,071	0,00603	1
GSE13738_RESTING_VS_BYSTANDER_ACTIVA	-0,066	0,071	0,00072	1
chr16p13	0,066	0,071	0,69748	1
REACTOME_SIGNALING_BY_FGFR1_FUSION_M	-0,066	0,071	0,08802	1
RESPONSE_TO_NUTRIENT	0,066	0,071	0,37295	1
DAZARD_RESPONSE_TO_UV_SCC_UP	-0,066	0,071	0,53842	1
MODULE_109	-0,066	0,071	0,00444	1
MAPKKK_CASCADE_GO_0000165	-0,066	0,071	0,00247	1
VITAMIN_TRANSPORT	-0,066	0,071	0,02862	1
CHIARADONNA_NEOPLASTIC_TRANSFORMATI	-0,066	0,071	0,01615	1
MODY_HIPPOCAMPUS_POSTNATAL	-0,066	0,071	0,00187	1
CELL_RECOGNITION	-0,066	0,071	0,02188	1
PROTEIN_TRANSPORT	-0,066	0,071	0,15465	1
MORF_SOD1	-0,066	0,072	0,83384	1
chr1p13	-0,066	0,072	0,01213	1
PID_ERB_GENOMIC_PATHWAY	-0,066	0,072	0,14392	1
GSE17721_ALL_VS_24H_PAM3CSK4_BMDM_	-0,066	0,072	0,07795	1
SMIRNOV_RESPONSE_TO_IR_2HR_UP	-0,066	0,072	0,06277	1
chr17q22	-0,066	0,072	0,00172	1
REACTOME_TRANSPORT_OF_ORGANIC_ANION	-0,066	0,072	0,07763	1
CALVET_IRINOTECAN_SENSITIVE_VS_REVERT	-0,066	0,072	0,04259	1
GSE37416_CTRL_VS_6H_F_TULARENSIS_LVS	-0,066	0,072	0,41219	1
PALOMERO_GSI_SENSITIVITY_UP	0,066	0,072	0,3915	1
CLAUS_PGR_POSITIVE_MENINGIOMA_UP	-0,066	0,072	0,00647	1
GNATENKO_PLATELET_SIGNATURE	-0,066	0,072	0,22368	1
EIF4E_UP	-0,066	0,072	0,20023	1
KEGG_GALACTOSE_METABOLISM	-0,066	0,072	0,76301	1
GSE3982_MAST_CELL_VS_EFF_MEMORY_CD4	-0,066	0,072	0,00299	1
HUTTMANN_B_CLL_POOR_SURVIVAL_UP	-0,066	0,072	0,00258	1

BERENJENO_TRANSFORMED_BY_RHOA_FOREV	-0,066	0,073	0,00564	1
AGING	-0,066	0,073	0,0066	1
WU_HBX_TARGETS_3_UP	-0,066	0,073	0,03414	1
ABBUD_LIF_SIGNALING_2_UP	-0,066	0,073	0,03809	1
REACTOME_SEROTONIN_RECEPTORS	-0,066	0,073	0,02942	1
GSE3982_DC_VS_MAC_DN	-0,066	0,073	0,15127	1
GSE3982_EOSINOPHIL_VS_BCELL_UP	-0,066	0,073	0,00945	1
WOUND_HEALING	-0,066	0,073	0,00047	1
SCHAEFFER_PROSTATE_DEVELOPMENT_AND_	-0,066	0,073	0,4465	1
BIOCARTA_P53HYPOXIA_PATHWAY	-0,066	0,073	0,01161	1
GSE22886_NAIVE_CD4_TCELL_VS_12H_ACT	-0,066	0,073	0,80347	1
GSE13411_SWITCHED_MEMORY_BCELL_VS_I	-0,066	0,073	0,04658	1
GARGALOVIC_RESPONSE_TO_OXIDIZED_PHOS	-0,066	0,073	0,80911	1
WALLACE_PROSTATE_CANCER_RACE_UP	-0,066	0,073	0,04685	1
TRANSFERASE_ACTIVITY_TRANSFERRING_ONI	-0,066	0,073	0,15829	1
VALK_AML_CLUSTER_15	-0,066	0,073	0,04083	1
EXTERNAL_SIDE_OF_PLASMA_MEMBRANE	-0,066	0,073	0,03802	1
BURTON_ADIPOGENESIS_PEAK_AT_0HR	-0,066	0,073	0,00144	1
chr9p22	-0,066	0,073	0,04064	1
MODULE_116	0,066	0,073	0,03995	1
WEBER_METHYLATED_LCP_IN_FIBROBLAST_I	-0,066	0,074	0,90022	1
OXIDOREDUCTASE_ACTIVITY_ACTING_ON_NA	0,066	0,074	0,028	1
RESPONSE_TO_EXTERNAL_STIMULUS	-0,066	0,074	0,03966	1
ONDER_CDH1_SIGNALING_VIA_CTNNB1	-0,065	0,074	0,00866	1
REGULATION_OF_HORMONE_SECRETION	-0,065	0,074	0,14743	1
chrxq12	-0,065	0,074	0,01943	1
LUI_THYROID_CANCER_CLUSTER_3	-0,065	0,074	0,08139	1
REACTOME_PI_3K_CASCADE	-0,065	0,074	0,0002	1
MORF_RAGE	-0,065	0,075	0,00062	1
ENZYME_BINDING	-0,065	0,075	0,00964	1
BIOPOLYMER_CATABOLIC_PROCESS	-0,065	0,075	0,38598	1
chr8p12	-0,065	0,075	0,09818	1
SHIN_B_CELL_LYMPHOMA_CLUSTER_8	-0,065	0,075	0,39757	1
GSE3982_DC_VS_NEUTROPHIL_LPS_STIM_DN	-0,065	0,075	0,05287	1
REACTOME_HDL_MEDIATED_LIPID_TRANSPOR	-0,065	0,075	0,01866	1
MODULE_245	-0,065	0,075	0,86482	1
NADERI_BREAST_CANCER_PROGNOSIS_DN	-0,065	0,075	0,00139	1
CYTOKINE_METABOLIC_PROCESS	-0,065	0,076	0,05775	1
GSE3982_EOSINOPHIL_VS_NKCELL_UP	-0,065	0,076	0,07432	1
MULLIGHAN_NPM1_MUTATED_SIGNATURE_2_	-0,065	0,076	0,00523	1
AKT_UP_MTOR_DN.V1_UP	-0,065	0,076	0,12715	1
FARMER_BREAST_CANCER_CLUSTER_6	-0,065	0,076	0,05481	1

B_CELL_ACTIVATION	-0,065	0,076	0,06852	1
RUFFLE	-0,065	0,076	0,04888	1
KEGG_LYSOSOME	0,065	0,076	0,07262	1
GNF2_IGFBP1	-0,065	0,076	0,00716	1
MANTOVANI_NFKB_TARGETS_UP	-0,065	0,076	0,33326	1
ELVIDGE_HIF2A_TARGETS_UP	0,065	0,076	0,43752	1
TSENG_IRS1_TARGETS_DN	-0,065	0,076	0,00593	1
GSE14000_UNSTIM_VS_4H_LPS_DC_UP	-0,065	0,077	0,00207	1
LIU_SOX4_TARGETS_DN	-0,065	0,077	0,09024	1
KREPPEL_CD99_TARGETS_UP	-0,065	0,077	0,52948	1
CASTELLANO_NRAS_TARGETS_UP	-0,065	0,077	0,13757	1
HASLINGER_B_CLL_WITH_11Q23_DELETION	-0,065	0,077	0,14621	1
V$USF_01	-0,065	0,077	0,03039	1
BURTON_ADIPOGENESIS_12	-0,065	0,077	0,15586	1
chr12q13	-0,065	0,077	0,00293	1
MODULE_240	-0,065	0,077	0,00156	1
GSE17721_LPS_VS_PAM3CSK4_2H_BMDM_U	-0,065	0,077	0,03883	1
BOYLAN_MULTIPLE_MYELOMA_PCA1_UP	-0,065	0,077	0,02805	1
REACTOME_PI3K_CASCADE	-0,065	0,077	0,00021	1
PID_UPA_UPAR_PATHWAY	-0,065	0,077	0,10415	1
MATZUK_POST-IMPLANTATION_AND_POST-PA	-0,065	0,077	0,07967	1
REACTOME_G_BETA_GAMMA_SIGNALLING_TH	-0,065	0,077	0,06542	1
RUTELLA_RESPONSE_TO_CSF2RB_AND_IL4_U	-0,065	0,077	0,20478	1
GSE26928_CENTR_MEMORY_VS_CXCR5_POS_	-0,065	0,078	0,0077	1
GSE26669_CTRL_VS_COSTIM_BLOCK_MLR_C	-0,065	0,078	0,00248	1
REGULATED_SECRETORY_PATHWAY	0,065	0,078	0,95579	1
BIOCARTA_AT1R_PATHWAY	-0,065	0,078	0,02862	1
LAMELLIPODIUM	-0,065	0,078	0,00189	1
REACTOME_DIABETES_PATHWAYS	-0,065	0,078	0,13687	1
LEADING_EDGE	-0,065	0,078	0,01908	1
chr5q	0,065	0,078	0,49743	1
RIZ_ERYTHROID_DIFFERENTIATION_12HR	-0,065	0,078	0,00034	1
SULFUR_COMPOUND_BIOSYNTHETIC_PROCESS	-0,064	0,079	0,11928	1
LEUKOCYTE_MIGRATION	-0,064	0,079	0,11409	1
MODULE_334	-0,064	0,079	0,00062	1
UBIQUITIN_LIGASE_COMPLEX	-0,064	0,079	0,48085	1
GSE37416_CTRL_VS_12H_F_TULARENSIS_LV	-0,064	0,079	0,49169	1
REACTOME_FORMATION_OF_INCISION_COMPL	0,064	0,079	0,56115	1
AMINO_SUGAR_METABOLIC_PROCESS	-0,064	0,079	0,47669	1
MODULE_190	-0,064	0,079	0,00113	1
GGTGAAG,MIR-412	-0,064	0,079	0,04105	1
MIKKELSEN_PARTIALLY_REPROGRAMMED_TO_	-0,064	0,079	0,47402	1

RUTELLA_RESPONSE_TO_HGF_DN	-0,064	0,079	0,02471	1
CGCAAAA,MIR-450	-0,064	0,08	0,00738	1
GSE30962_PRIMARY_VS_SECONDARY_CHRON	-0,064	0,08	0,01591	1
MODULE_547	-0,064	0,08	0,00057	1
SESTO_RESPONSE_TO_UV_C5	-0,064	0,08	0,21412	1
GSE28237_EARLY_VS_LATE_GC_BCELL_UP	-0,064	0,08	0,00307	1
GSE1460_CD4_THYMOCYTE_VS_NAIVE_CD4_	-0,064	0,08	0,00423	1
PROTEIN_AMINO_ACID_N_LINKED_GLYCOSYL	-0,064	0,08	0,14509	1
ATGTTAA,MIR-302C	-0,064	0,08	0,01463	1
CYTOKINE_BINDING	-0,064	0,08	0,09115	1
MODULE_75	-0,064	0,08	0,04409	1
MORF_ERH	-0,064	0,08	0,81118	1
GSE22886_NAIVE_CD8_TCELL_VS_MEMORY_	-0,064	0,081	0,03807	1
REGULATION_OF_VIRAL_REPRODUCTION	-0,064	0,081	0,28215	1
GSE28237_EARLY_VS_LATE_GC_BCELL_DN	-0,064	0,081	0,00399	1
ONGUSAHA_BRCA1_TARGETS_DN	-0,064	0,081	0,79124	1
MORF_TPR	-0,064	0,081	0,16057	1
V$HNF1_Q6	-0,064	0,081	0,00285	1
BCAT_BILD_ET_AL_DN	-0,064	0,081	0,04803	1
ZHENG_FOXP3_TARGETS_IN_THYMUS_UP	-0,064	0,081	0,0559	1
GNF2_PCAF	0,064	0,081	0,22177	1
REACTOME_INTEGRATION_OF_ENERGY_METAI	-0,064	0,081	0,00065	1
GSE3982_MAC_VS_BASOPHIL_UP	-0,064	0,081	0,07871	1
NEGATIVE_REGULATION_OF_MULTICELLULAR_	-0,064	0,081	0,02094	1
PROTEIN_LOCALIZATION	-0,064	0,081	0,05958	1
KAUFFMANN_MELANOMA_RELAPSE_DN	0,064	0,081	0,63264	1
REACTOME_DARPP_32_EVENTS	-0,064	0,081	0,2774	1
PID_CD8TCRPATHWAY	-0,064	0,081	0,08802	1
GSE17974_IL4_AND_ANTI_IL12_VS_UNTREA	-0,064	0,081	0,01665	1
NIKOLSKY_BREAST_CANCER_7P15_AMPLICON	-0,064	0,082	0,06358	1
ZHANG_ANTIVIRAL_RESPONSE_TO_RIBAVIRIN	-0,064	0,082	0,02954	1
V$HNF4_Q6	-0,064	0,082	0,00028	1
GSE37416_0H_VS_12H_F_TULARENSIS_LVS	-0,064	0,082	0,18448	1
CHESLER_BRAIN_D6MIT150_QTL_CIS	0,064	0,082	0,78303	1
JOSEPH_RESPONSE_TO_SODIUM_BUTYRATE_I	-0,064	0,082	0,00377	1
MARTENS_TRETINOIN_RESPONSE_UP	-0,064	0,082	0,00967	1
MODULE_292	-0,064	0,082	0,07264	1
TATCTGG,MIR-488	-0,064	0,082	0,03325	1
FARMER_BREAST_CANCER_CLUSTER_4	-0,064	0,082	0,4958	1
TTGCACT,MIR-130A,MIR-301,MIR-130B	-0,064	0,082	0,00966	1
GNF2_JAK1	-0,064	0,082	0,05025	1
LOCOMOTORY_BEHAVIOR	-0,064	0,083	0,1727	1

GSE2706_R848_VS_R848_AND_LPS_8H_STI	-0,064	0,083	0,20474	1
REACTOME_AMINO_ACID_SYNTHESIS_AND_IN	-0,064	0,083	0,90032	1
BIOCARTA_TFF_PATHWAY	-0,064	0,083	0,29654	1
ATGCACG,MIR-517B	-0,064	0,083	0,0533	1
GSE17721_POLYIC_VS_PAM3CSK4_12H_BMI	-0,064	0,083	0,17216	1
PARK_APL_PATHOGENESIS_UP	-0,064	0,083	0,22023	1
GSE27786_LSK_VS_NKCELL_DN	-0,064	0,083	0,01974	1
TAANNYSGCG_UNKNOWN	-0,064	0,083	0,26378	1
REACTOME_CHOLESTEROL_BIOSYNTHESIS	-0,064	0,083	0,56535	1
GUTIERREZ_WALDENSTROEMS_MACROGLOBUI	-0,064	0,083	0,05578	1
GNF2_MLF1	-0,064	0,083	0,04044	1
GSE20366_CD103_POS_VS_NEG_TREG_KLR(	-0,064	0,083	0,04046	1
REACTOME_AUTODEGRADATION_OF_THE_E3_	-0,064	0,083	0,97083	1
REGULATION_OF_NEUROTRANSMITTER_LEVEL	0,063	0,083	0,9089	1
VIRAL_REPRODUCTIVE_PROCESS	-0,063	0,083	0,74545	1
TTTGCAC,MIR-19A,MIR-19B	-0,063	0,083	0,00494	1
VIRAL_INFECTIOUS_CYCLE	-0,063	0,083	0,85647	1
MULLIGHAN_NPM1_MUTATED_SIGNATURE_2_	-0,063	0,083	0,01132	1
chr17q	-0,063	0,084	0,07738	1
RESPONSE_TO_STEROID_HORMONE_STIMULUS	0,063	0,084	0,86157	1
GSE29618_BCELL_VS_MDC_DN	-0,063	0,084	0,10821	1
TONKS_TARGETS_OF_RUNX1_RUNX1T1_FUSI	-0,063	0,084	0,01091	1
GSE36476_CTRL_VS_TSST_ACT_16H_MEMO	-0,063	0,084	0,0079	1
ATPASE_ACTIVITY_COUPLED	-0,063	0,084	0,0246	1
MORF_PPP1CA	-0,063	0,084	0,57988	1
CRX_DN.V1_UP	-0,063	0,084	0,00015	1
STARK_BRAIN_22Q11_DELETION	-0,063	0,084	0,01476	1
HAHTOLA_MYCOSIS_FUNGOIDES_CD4_UP	-0,063	0,084	0,08609	1
ROESSLER_LIVER_CANCER_METASTASIS_UP	-0,063	0,084	0,21909	1
STARK_PREFRONTAL_CORTEX_22Q11_DELET	-0,063	0,084	0,22925	1
MODULE_191	-0,063	0,084	0,12212	1
N_ACETYLGLUCOSAMINE_METABOLIC_PROCES	-0,063	0,085	0,30503	1
V$GABP_B	-0,063	0,085	0,05699	1
chr20p11	0,063	0,085	0,86336	1
chr19q	-0,063	0,085	0,13628	1
WANG_RESPONSE_TO_ANDROGEN_UP	-0,063	0,085	0,0576	1
VIRAL_REPRODUCTION	-0,063	0,085	0,61234	1
GSE17721_LPS_VS_POLYIC_16H_BMDM_UP	-0,063	0,085	0,08297	1
KINASE_ACTIVATOR_ACTIVITY	-0,063	0,085	0,12693	1
RIBOSOME	-0,063	0,085	0,14033	1
GSE17721_LPS_VS_CPG_2H_BMDM_DN	-0,063	0,085	0,02484	1
GSE17721_CTRL_VS_GARDIQUIMOD_1H_BMI	-0,063	0,085	0,00967	1

REACTOME_NFKB_ACTIVATION_THROUGH_FA	0,063	0,085	0,15572	1
GSE17721_CTRL_VS_POLYIC_12H_BMDM_UI	-0,063	0,085	0,034	1
RODRIGUES_DCC_TARGETS_UP	0,063	0,085	0,05515	1
GSE17721_PAM3CSK4_VS_GADIQUIMOD_12I	-0,063	0,085	0,11017	1
DANG_MYC_TARGETS_DN	-0,063	0,085	0,04672	1
SILIGAN_TARGETS_OF_EWS_FLI1_FUSION_DN	-0,063	0,086	0,0319	1
GSE17721_PAM3CSK4_VS_GADIQUIMOD_4H_	-0,063	0,086	0,00241	1
HINATA_NFKB_IMMU_INF	-0,063	0,086	0,44843	1
ACACTAC,MIR-142-3P	-0,063	0,086	0,08585	1
GSE36392_TYPE_2_MYELOID_VS_NEUTROPH	-0,063	0,086	0,0072	1
GSE17721_CTRL_VS_GARDIQUIMOD_24H_BN	-0,063	0,086	0,00085	1
FATTY_ACID_BIOSYNTHETIC_PROCESS	-0,063	0,086	0,14369	1
GSE34205_RSV_VS_FLU_INF_INFANT_PBMC_	-0,063	0,086	0,16275	1
ONO_AML1_TARGETS_DN	-0,063	0,086	0,09797	1
chr4q	-0,063	0,086	0,02179	1
DACOSTA_UV_RESPONSE_VIA_ERCC3_UP	-0,063	0,087	0,13354	1
COULOUARN_TEMPORAL_TGFB1_SIGNATURE_	-0,063	0,087	0,00239	1
LEE_LIVER_CANCER_DENA_DN	-0,063	0,087	0,01756	1
RHEIN_ALL_GLUCOCORTICOID_THERAPY_UP	-0,063	0,087	0,10344	1
JAATINEN_HEMATOPOIETIC_STEM_CELL_DN	-0,063	0,087	0,06079	1
PROTEASOME_COMPLEX	-0,063	0,087	0,81395	1
GLINSKY_CANCER_DEATH_UP	-0,063	0,087	0,72222	1
GSE17974_CTRL_VS_ACT_IL4_AND_ANTI_IL	-0,063	0,087	0,00317	1
PATTERN_BINDING	-0,063	0,087	0,01754	1
WEBER_METHYLATED_LCP_IN_SPERM_DN	-0,063	0,087	0,7803	1
TURJANSKI_MAPK14_TARGETS	-0,063	0,087	0,00893	1
GUTIERREZ_CHRONIC_LYMPHOCYTIC_LEUKEMI	-0,063	0,087	0,00398	1
MELLMAN_TUT1_TARGETS_UP	-0,063	0,087	0,72215	1
GSE39820_TGFBETA1_IL6_VS_TGFBETA1_IL	-0,063	0,087	0,06043	1
TAVOR_CEBPA_TARGETS_DN	-0,063	0,087	0,02138	1
NAGASHIMA_NRG1_SIGNALING_DN	-0,063	0,087	0,04518	1
PID_KITPATHWAY	-0,063	0,087	0,02218	1
GSE15930_STIM_VS_STIM_AND_IL-12_48H_	-0,063	0,087	0,02153	1
CHEN_METABOLIC_SYNDROM_NETWORK	-0,063	0,088	0,03597	1
SMALL_GTPASE_BINDING	-0,063	0,088	0,188	1
DORSAM_HOXA9_TARGETS_UP	-0,063	0,088	0,00785	1
MORF_KDR	-0,063	0,088	0,00154	1
JI_RESPONSE_TO_FSH_DN	-0,063	0,088	0,37203	1
VANASSE_BCL2_TARGETS_DN	-0,063	0,088	0,01961	1
GSE17721_CTRL_VS_POLYIC_0.5H_BMDM_U	-0,063	0,088	0,0117	1
NEUROPEPTIDE_RECEPTOR_ACTIVITY	-0,062	0,088	0,02254	1
GNF2_RAB7L1	-0,062	0,089	0,08592	1

MODULE_155	-0,062	0,089	0,78961	1
CELLULAR_DEFENSE_RESPONSE	-0,062	0,089	0,07785	1
chr14q21	-0,062	0,089	0,00528	1
NEGATIVE_REGULATION_OF_SIGNAL_TRANSD	-0,062	0,089	0,00049	1
MORF_MTA1	-0,062	0,089	0,82049	1
KYNG_DNA_DAMAGE_BY_GAMMA_AND_UV_I	-0,062	0,089	0,01289	1
PLASARI_TGFB1_SIGNALING_VIA_NFIC_10HR_	-0,062	0,089	0,02037	1
GSE17974_0H_VS_12H_IN_VITRO_ACT_CD4	-0,062	0,089	0,00152	1
GSE3982_EOSINOPHIL_VS_DC_DN	-0,062	0,089	0,20144	1
GSE17721_LPS_VS_PAM3CSK4_24H_BMDM_	-0,062	0,089	0,01243	1
REACTOME_ACETYLCHOLINE_BINDING_AND_D	-0,062	0,089	0,27102	1
CAFFAREL_RESPONSE_TO_THC_UP	-0,062	0,089	0,29204	1
VESICLE	-0,062	0,089	0,01023	1
HADDAD_T_LYMPHOCYTE_AND_NK_PROGENI	-0,062	0,089	0,04069	1
REACTOME_SCF_BETA_TRCP_MEDIATED_DEG	-0,062	0,089	0,99176	1
RAS_GTPASE_BINDING	-0,062	0,09	0,33724	1
RNA_HELICASE_ACTIVITY	-0,062	0,09	0,76803	1
APPIERTO_RESPONSE_TO_FENRETINIDE_UP	-0,062	0,09	0,22136	1
GSE30083_SP1_VS_SP4_THYMOCYTE_DN	-0,062	0,09	0,03011	1
FUNG_IL2_SIGNALING_1	-0,062	0,09	0,27182	1
IVANOVA_HEMATOPOIESIS_INTERMEDIATE_PR	-0,062	0,09	0,21662	1
CYTOKINE_BIOSYNTHETIC_PROCESS	-0,062	0,09	0,06037	1
CROMER_METASTASIS_UP	-0,062	0,09	0,19064	1
MURATA_VIRULENCE_OF_H_PILORI	-0,062	0,09	0,51171	1
ANGIOGENESIS	-0,062	0,091	0,08118	1
chr1q12	-0,062	0,091	0,16169	1
MORF_USP5	-0,062	0,091	0,88816	1
GALI_TP53_TARGETS_APOPTOTIC_DN	-0,062	0,091	0,7544	1
MORF_FDXR	-0,062	0,091	0,00304	1
GSE24142_DN2_VS_DN3_THYMOCYTE_ADUL	-0,062	0,091	0,04078	1
CYTOPLASMIC_VESICLE	-0,062	0,091	0,01062	1
ST_ERK1_ERK2_MAPK_PATHWAY	-0,062	0,091	0,12199	1
MATZUK_EMBRYONIC_GERM_CELL	-0,062	0,091	0,0085	1
ROLEF_GLIS3_TARGETS	-0,062	0,091	0,00056	1
REACTOME_AKT_PHOSPHORYLATES_TARGETS	-0,062	0,091	0,00082	1
GSE22886_NAIVE_CD8_TCELL_VS_NEUTROP	-0,062	0,091	0,20762	1
SHETH_LIVER_CANCER_VS_TXNIP_LOSS_PAM	-0,062	0,091	0,00152	1
MODULE_480	-0,062	0,091	0,00035	1
GSE18791_CTRL_VS_NEWCASTLE_VIRUS_DC	-0,062	0,092	0,26292	1
BIOCARTA_TOB1_PATHWAY	-0,062	0,092	0,02758	1
PID_TCR_PATHWAY	-0,062	0,092	0,08425	1
REACTOME_GAP_JUNCTION_ASSEMBLY	-0,062	0,092	0,92802	1

REACTOME_TRAF6_MEDIATED_INDUCTION_OI	-0,062	0,092	0,11311	1
GSE39820_CTRL_VS_TGFBETA1_IL6_IL23A_	-0,062	0,092	0,07431	1
GSE3982_MAC_VS_BCELL_UP	-0,062	0,092	0,3583	1
REACTOME_SIGNAL_AMPLIFICATION	-0,062	0,092	0,09146	1
REACTOME_IMMUNE_SYSTEM	-0,062	0,093	0,1522	1
XU_HGF_SIGNALING_NOT_VIA_AKT1_48HR_I	-0,062	0,093	0,41518	1
GNF2_BNIP2	-0,062	0,093	0,26003	1
REACTOME_CDK_MEDIATED_PHOSPHORYLATI	-0,062	0,093	0,98725	1
V$HNF1_01	-0,062	0,093	0,00027	1
WANG_BARRETTS_ESOPHAGUS_UP	-0,062	0,093	0,06212	1
SHIN_B_CELL_LYMPHOMA_CLUSTER_6	-0,061	0,094	0,64682	1
ABBUD_LIF_SIGNALING_1_UP	-0,061	0,094	0,06177	1
PRODUCTION_OF_MOLECULAR_MEDIATOR_OF	-0,061	0,094	0,0459	1
REACTOME_COPI_MEDIATED_TRANSPORT	0,061	0,094	0,01679	1
REACTOME_GRB2_SOS_PROVIDES_LINKAGE_	-0,061	0,094	0,10258	1
BIOCARTA_IGF1MTOR_PATHWAY	-0,061	0,094	0,00155	1
DEBOSSCHER_NFKB_TARGETS_REPRESSED_B	-0,061	0,094	0,29536	1
CHIANG_LIVER_CANCER_SUBCLASS_CTNNB1_	-0,061	0,094	0,00021	1
ENDODEOXYRIBONUCLEASE_ACTIVITY	-0,061	0,094	0,36344	1
GSE16755_CTRL_VS_IFNA_TREATED_MAC_D	-0,061	0,094	0,24503	1
GSE17721_LPS_VS_POLYIC_4H_BMDM_DN	-0,061	0,094	0,06572	1
GSE2826_WT_VS_BTK_KO_BCELL_DN	-0,061	0,094	0,04795	1
GSE14308_TH2_VS_TH17_DN	-0,061	0,095	0,02357	1
MODULE_73	-0,061	0,095	0,17017	1
PML_BODY	-0,061	0,095	0,00312	1
GSE13738_TCR_VS_BYSTANDER_ACTIVATED	-0,061	0,095	0,03892	1
SECRETION_BY_CELL	0,061	0,095	0,81765	1
BIOCARTA_FCER1_PATHWAY	-0,061	0,095	0,03071	1
chr1q21	-0,061	0,095	0,11694	1
LANDIS_BREAST_CANCER_PROGRESSION_UP	-0,061	0,095	0,8997	1
BIOCARTA_WNT_PATHWAY	-0,061	0,095	0,00616	1
BIOCARTA_UCALPAIN_PATHWAY	-0,061	0,095	0,45981	1
GOLGI_APPARATUS_PART	0,061	0,095	0,16768	1
NUCLEAR_REPLICATION_FORK	-0,061	0,095	0,57812	1
GSE24142_EARLY_THYMIC_PROGENITOR_VS_	-0,061	0,095	0,02834	1
MODULE_325	-0,061	0,095	0,00101	1
XU_AKT1_TARGETS_48HR	-0,061	0,096	0,0396	1
AMIT_EGF_RESPONSE_120_MCF10A	-0,061	0,096	0,46082	1
GSE17721_POLYIC_VS_PAM3CSK4_2H_BMDI	-0,061	0,096	0,02512	1
KOBAYASHI_EGFR_SIGNALING_6HR_DN	-0,061	0,096	0,10048	1
REACTOME_TRNA_AMINOACYLATION	-0,061	0,096	0,81898	1
GSE17721_PAM3CSK4_VS_CPG_2H_BMDM_I	-0,061	0,096	0,06031	1

chr15q21	-0,061	0,096	0,04106	1
IRITANI_MAD1_TARGETS_UP	-0,061	0,096	0,09604	1
REACTOME_PROSTACYCLIN_SIGNALLING_THR	-0,061	0,097	0,09085	1
FATTY_ACID_OXIDATION	-0,061	0,097	0,0198	1
MULLIGAN_NTF3_SIGNALING_VIA_INSR_AND_	-0,061	0,097	0,00235	1
LIPOPROTEIN_METABOLIC_PROCESS	0,061	0,097	0,52503	1
VERHAAK_GLIOBLASTOMA_NEURAL	-0,061	0,097	0,12914	1
DEFENSE_RESPONSE	-0,061	0,097	0,08726	1
BIOCARTA_ATRBRCA_PATHWAY	-0,061	0,097	0,34424	1
YANG_MUC2_TARGETS_DUODENUM_6MO_DN	0,061	0,097	0,45554	1
CCACACA,MIR-147	-0,061	0,097	0,01168	1
TCTCTCC,MIR-185	-0,061	0,098	0,01316	1
JONES_TCOF1_TARGETS	0,061	0,098	0,27426	1
GSE24142_ADULT_VS_FETAL_DN2_THYMOC	-0,061	0,098	0,02628	1
GSE22886_NAIVE_CD4_TCELL_VS_MEMORY_	-0,061	0,098	0,23777	1
CHANDRAN_METASTASIS_TOP50_DN	-0,061	0,098	0,00067	1
GSE17721_LPS_VS_CPG_12H_BMDM_UP	-0,061	0,098	0,0726	1
chr3p21	0,061	0,098	0,67129	1
MORF_MAP2K2	-0,061	0,098	0,72923	1
V$TFIII_Q6	-0,061	0,098	0,00129	1
REACTOME_CD28_DEPENDENT_VAV1_PATHW	-0,061	0,098	0,14764	1
SPHINGOID_METABOLIC_PROCESS	0,061	0,098	0,3428	1
GSE6269_HEALTHY_VS_E_COLI_INF_PBMC_U	-0,061	0,098	0,00358	1
MIKKELSEN_ES_LCP_WITH_H3K4ME3	-0,061	0,098	0,00153	1
KHETCHOUMIAN_TRIM24_TARGETS_UP	-0,061	0,098	0,05174	1
MCCABE_HOXC6_TARGETS_UP	-0,061	0,098	0,11741	1
PID_ECADHERIN_STABILIZATION_PATHWAY	-0,061	0,098	0,29861	1
REACTOME_REGULATION_OF_INSULIN_SECRET	-0,061	0,098	0,0041	1
GSE11864_UNTREATED_VS_CSF1_IFNG_IN_M	-0,061	0,098	0,48004	1
GSE17721_0.5H_VS_24H_CPG_BMDM_DN	-0,061	0,098	0,15687	1
IMMUNE_SYSTEM_PROCESS	-0,061	0,099	0,09683	1
GSE9037_WT_VS_IRAK4_KO_BMDM_UP	-0,061	0,099	0,03981	1
REACTOME_UNBLOCKING_OF_NMDA_RECEPTO	-0,06	0,099	0,00426	1
MOOTHA_FFA_OXYDATION	0,06	0,099	0,44777	1
NEGATIVE_REGULATION_OF_CELL_MIGRATION	-0,06	0,099	0,03246	1
REACTOME_IMMUNOREGULATORY_INTERACTIO	-0,06	0,099	0,20624	1
NON_MEMBRANE_SPANNING_PROTEIN_TYROS	-0,06	0,099	0,01356	1
MOOTHA_ROS	0,06	0,099	0,08831	1
REGULATION_OF_BLOOD_PRESSURE	-0,06	0,099	0,04091	1
ATGTTTC,MIR-494	-0,06	0,099	0,00679	1
GSE30083_SP2_VS_SP4_THYMOCYTE_DN	-0,06	0,1	0,03191	1
KENNY_CTNNB1_TARGETS_DN	-0,06	0,1	0,00073	1

GSE17974_CTRL_VS_ACT_IL4_AND_ANTI_IL	-0,06	0,1	0,0115	1
BOCHKIS_FOXA2_TARGETS	-0,06	0,1	0,00098	1
REGULATION_OF_CYTOKINE_BIOSYNTHETIC_F	-0,06	0,1	0,06538	1
JOHANSSON_BRAIN_CANCER_EARLY_VS_LAT	-0,06	0,1	0,04134	1
REACTOME_PRESYNAPTIC_NICOTINIC_ACETYL	-0,06	0,1	0,22494	1
REACTOME_CHYLOMICRON_MEDIATED_LIPID_	-0,06	0,1	0,07022	1
CYSTEINE_TYPE_PEPTIDASE_ACTIVITY	-0,06	0,1	0,5819	1
MODULE_108	-0,06	0,1	0,29858	1
GSE37416_12H_VS_24H_F_TULARENSIS_LV	-0,06	0,1	0,12863	1
TCCCRNNRTGC_UNKNOWN	-0,06	0,101	0,04001	1
IKEDA_MIR133_TARGETS_DN	-0,06	0,101	0,20022	1
REACTOME_FGFR2C_LIGAND_BINDING_AND_/	-0,06	0,101	0,02734	1
MODULE_288	-0,06	0,101	0,0229	1
STK33_SKM_UP	-0,06	0,101	0,05125	1
TBK1.DF_DN	-0,06	0,101	0,15635	1
LOPEZ_MESOTHELIOMA_SURVIVAL_WORST_V	-0,06	0,101	0,24952	1
KMCATNNWGGA_UNKNOWN	-0,06	0,101	0,02962	1
KOINUMA_COLON_CANCER_MSI_DN	-0,06	0,102	0,23876	1
GSE360_DC_VS_MAC_T_GONDII_DN	-0,06	0,102	0,10788	1
REACTOME_IKK_COMPLEX_RECRUITMENT_MEI	0,06	0,102	0,28383	1
SHIN_B_CELL_LYMPHOMA_CLUSTER_3	-0,06	0,102	0,02327	1
REACTOME_IRAK1_RECRUITS_IKK_COMPLEX	-0,06	0,102	0,26705	1
COURTOIS_SENESCENCE_TRIGGERS	-0,06	0,102	0,25567	1
GSE9006_TYPE_1_VS_TYPE_2_DIABETES_PE	-0,06	0,102	0,08846	1
chr3p12	-0,06	0,102	0,0184	1
GSE339_CD8POS_VS_CD4CD8DN_DC_IN_CUI	-0,06	0,102	0,13226	1
GSE6269_FLU_VS_STREP_PNEUMO_INF_PBM(	-0,06	0,102	0,24932	1
MODULE_474	-0,06	0,102	0,0027	1
LIPID_KINASE_ACTIVITY	-0,06	0,102	0,26435	1
GSE26928_NAIVE_VS_CENT_MEMORY_CD4_	-0,06	0,102	0,00156	1
MOREAUX_B_LYMPHOCYTE_MATURATION_BY_	-0,06	0,102	0,03176	1
GSE360_L_DONOVANI_VS_L_MAJOR_DC_DN	-0,06	0,102	0,0034	1
REACTOME_PROLACTIN_RECEPTOR_SIGNALIN(	0,06	0,102	0,92038	1
GSE24634_IL4_VS_CTRL_TREATED_NAIVE_(	-0,06	0,102	0,06213	1
LINDGREN_BLADDER_CANCER_CLUSTER_1_UI	-0,06	0,102	0,0033	1
chr19p12	-0,06	0,102	0,02672	1
V$OCT1_01	-0,06	0,102	0,00435	1
GSE27786_BCELL_VS_CD4_TCELL_UP	-0,06	0,103	0,01946	1
GSE5960_TH1_VS_ANERGIC_TH1_DN	-0,06	0,103	0,11646	1
GSE22886_NAIVE_CD4_TCELL_VS_DC_DN	0,06	0,103	0,02883	1
VALK_AML_WITH_11Q23_REARRANGED	-0,06	0,103	0,03132	1
REGULATION_OF_CATABOLIC_PROCESS	-0,06	0,103	0,00404	1

GSE3982_MAST_CELL_VS_BCELL_UP	-0,06	0,103	0,11154	1
MIDORIKAWA_AMPLIFIED_IN_LIVER_CANCER	-0,06	0,103	0,14093	1
MODULE_262	-0,06	0,103	0,13301	1
chr20q12	-0,06	0,103	0,09513	1
MIKKELSEN_MCV6_ICP_WITH_H3K27ME3	-0,06	0,103	0,03295	1
GNF2_MSN	-0,06	0,103	0,37345	1
GSE3982_MEMORY_CD4_TCELL_VS_BCELL_U	-0,06	0,103	0,02072	1
CROONQUIST_NRAS_VS_STROMAL_STIMULAT	-0,06	0,103	0,02215	1
BAE_BRCA1_TARGETS_UP	-0,06	0,103	0,1507	1
INTERLEUKIN_RECEPTOR_ACTIVITY	-0,06	0,103	0,19838	1
REACTOME_NOREPINEPHRINE_NEUROTRANSMI	0,06	0,103	0,88881	1
FIGUEROA_AML_METHYLATION_CLUSTER_4_I	-0,06	0,104	0,09658	1
AMINE_TRANSMEMBRANE_TRANSPORTER_AC	-0,06	0,104	0,06308	1
MORF_LTK	-0,06	0,104	0,00059	1
HUANG_DASATINIB_RESISTANCE_DN	0,06	0,104	0,16614	1
GSE14000_UNSTIM_VS_4H_LPS_DC_TRANSL	-0,06	0,104	0,40425	1
REACTOME_MTORC1_MEDIATED_SIGNALLING	-0,06	0,104	0,20146	1
GSE7852_TREG_VS_TCONV_THYMUS_DN	-0,06	0,104	0,00037	1
chr5q23	-0,06	0,104	0,00056	1
chr10p14	-0,06	0,104	0,699	1
BIOCARTA_STEM_PATHWAY	-0,06	0,104	0,2582	1
GSE11864_UNTREATED_VS_CSF1_PAM3CYS_	-0,06	0,105	0,01741	1
MODULE_165	-0,06	0,105	0,12305	1
TRIACYLGLYCEROL_METABOLIC_PROCESS	-0,059	0,105	0,09995	1
JIANG_AGING_CEREBRAL_CORTEX_DN	-0,059	0,105	0,01618	1
V$IRF_Q6	-0,059	0,105	0,03742	1
GTTNYYNNGGTNA_UNKNOWN	-0,059	0,105	0,00097	1
PROTEIN_FOLDING	-0,059	0,105	0,72011	1
GSE14769_UNSTIM_VS_240MIN_LPS_BMDM_	-0,059	0,105	0,08946	1
BYSTRYKH_HEMATOPOIESIS_STEM_CELL_ANE	-0,059	0,105	0,28108	1
DACOSTA_UV_RESPONSE_VIA_ERCC3_COMM	-0,059	0,105	0,0836	1
GRAESSMANN_RESPONSE_TO_MC_AND_SERU	-0,059	0,106	0,02597	1
DIRMEIER_LMP1_RESPONSE_EARLY	-0,059	0,106	0,03089	1
BIOCARTA_NKT_PATHWAY	-0,059	0,106	0,13837	1
GSE13484_UNSTIM_VS_YF17D_VACCINE_STI	-0,059	0,106	0,27954	1
GSE3982_BASOPHIL_VS_TH2_UP	-0,059	0,106	0,02245	1
MODULE_19	-0,059	0,106	0,00085	1
CARBOXYPEPTIDASE_ACTIVITY	-0,059	0,106	0,00787	1
GSE13306_TREG_VS_TCONV_SPLEEN_UP	-0,059	0,106	0,00444	1
chr7q36	-0,059	0,106	0,04357	1
GSE27786_CD4_TCELL_VS_NEUTROPHIL_DN	-0,059	0,106	0,00028	1
GSE360_L_DONOVANI_VS_L_MAJOR_DC_UP	-0,059	0,106	0,00395	1

CYTOKINE_PRODUCTION	-0,059	0,106	0,0472	1
GSE12366_GC_BCELL_VS_PLASMA_CELL_DN	-0,059	0,106	0,06594	1
SYMPORTER_ACTIVITY	-0,059	0,107	0,61888	1
SCHMIDT_POR_TARGETS_IN_LIMB_BUD_DN	-0,059	0,107	0,00049	1
BIOCARTA_VIP_PATHWAY	-0,059	0,107	0,00054	1
ANASTASSIOU_CANCER_MESENCHYMAL_TRAI	-0,059	0,107	0,0399	1
GSE17721_CPG_VS_GARDIQUIMOD_16H_BMD	-0,059	0,107	0,06432	1
ELVIDGE_HYPOXIA_DN	-0,059	0,107	0,25915	1
GSE37416_12H_VS_48H_F_TULARENSIS_LV	-0,059	0,107	0,00893	1
NUNODA_RESPONSE_TO_DASATINIB_IMATINII	-0,059	0,107	0,23656	1
SCIAN_INVERSED_TARGETS_OF_TP53_AND_	-0,059	0,107	0,25294	1
REACTOME_MICRORNA_MIRNA_BIOGENESIS	-0,059	0,108	0,61722	1
KEEN_RESPONSE_TO_ROSIGLITAZONE_UP	0,059	0,108	0,91896	1
GSE13306_TREG_VS_TCONV_LAMINA_PROPI	-0,059	0,108	0,00428	1
WILCOX_PRESPONSE_TO_ROGESTERONE_DN	-0,059	0,108	0,01237	1
MYLLYKANGAS_AMPLIFICATION_HOT_SPOT_	-0,059	0,108	0,59967	1
REACTOME_G_BETA_GAMMA_SIGNALLING_TH	-0,059	0,108	0,04069	1
HOFFMANN_SMALL_PRE_BII_TO_IMMATURE_I	-0,059	0,108	0,04818	1
REACTOME_GLUCONEOGENESIS	0,059	0,108	0,06689	1
KEGG_TASTE_TRANSDUCTION	-0,059	0,108	0,0275	1
ISOPRENOID_METABOLIC_PROCESS	-0,059	0,108	0,2593	1
GSE30962_ACUTE_VS_CHRONIC_LCMV_PRIM	-0,059	0,108	0,00834	1
HEIDENBLAD_AMPLICON_8Q24_DN	-0,059	0,108	0,07998	1
GSE1460_INTRATHYMIC_T_PROGENITOR_VS_	-0,059	0,109	0,00781	1
CONRAD_GERMLINE_STEM_CELL	-0,059	0,109	0,30697	1
BIOCARTA_GH_PATHWAY	-0,059	0,109	0,01781	1
REACTOME_ALPHA_LINOLENIC_ACID_ALA_MI	-0,059	0,109	0,06341	1
MODULE_241	-0,059	0,109	0,23384	1
FEVR_CTNNB1_TARGETS_UP	-0,059	0,109	0,01167	1
BIOCARTA_IL6_PATHWAY	-0,059	0,109	0,04743	1
GSE12366_GC_VS_NAIVE_BCELL_DN	-0,059	0,109	0,01641	1
REACTOME_SIGNALING_BY_ILS	-0,059	0,109	0,20082	1
PROTEIN_CATABOLIC_PROCESS	-0,059	0,109	0,34484	1
BIOCARTA_TH1TH2_PATHWAY	-0,059	0,109	0,22533	1
MODULE_151	-0,059	0,109	0,17706	1
GSE22886_UNSTIM_VS_IL2_STIM_NKCELL_U	-0,059	0,109	0,00042	1
GSE10463_CD40L_AND_VA347_VS_CD40L_	-0,059	0,11	0,02137	1
WONG_ENDOMETRIAL_CANCER_LATE	-0,059	0,11	0,04339	1
REACTOME_ACTIVATION_OF_BH3_ONLY_PRC	-0,059	0,11	0,01191	1
VANDESLUIS_COMMD1_TARGETS_GROUP_4_I	-0,059	0,11	0,00105	1
KEGG_VIRAL_MYOCARDITIS	-0,059	0,11	0,07672	1
GSE1460_INTRATHYMIC_T_PROGENITOR_VS_	-0,059	0,11	0,02202	1

FEMALE_PREGNANCY	-0,059	0,11	0,03216	1
GSE17721_POLYIC_VS_PAM3CSK4_16H_BMI	-0,059	0,11	0,0351	1
BODY_FLUID_SECRETION	0,059	0,11	0,2234	1
CTCTATG,MIR-368	-0,059	0,11	0,00171	1
FARMER_BREAST_CANCER_CLUSTER_8	-0,059	0,11	0,09355	1
REGULATION_OF_CELL_MORPHOGENESIS	-0,059	0,11	0,05512	1
RUIZ_TNC_TARGETS_UP	-0,059	0,111	0,00384	1
MORF_REV3L	-0,059	0,111	0,16607	1
FUNG_IL2_TARGETS_WITH_STAT5_BINDING_	-0,058	0,111	0,17901	1
CHENG_TAF7L_TARGETS	0,058	0,111	0,23824	1
MONNIER_POSTRADIATION_TUMOR_ESCAPE_I	-0,058	0,111	0,0122	1
MYLLYKANGAS_AMPLIFICATION_HOT_SPOT_7	-0,058	0,112	0,27917	1
GLUCOSAMINE_METABOLIC_PROCESS	-0,058	0,112	0,33497	1
WINTER_HYPOXIA_DN	-0,058	0,113	0,01026	1
PROTEIN_PROCESSING	-0,058	0,113	0,04396	1
REACTOME_REGULATED_PROTEOLYSIS_OF_P7	-0,058	0,113	0,00304	1
REACTOME_ANDROGEN_BIOSYNTHESIS	-0,058	0,113	0,17942	1
MORF_NF1	-0,058	0,113	0,00855	1
REACTOME_PLATELET_AGGREGATION_PLUG_	-0,058	0,113	0,01024	1
chr2q13	-0,058	0,113	0,3135	1
chr4q23	-0,058	0,114	0,00072	1
MORF_SKP1A	-0,058	0,114	0,84224	1
GSE24634_NAIVE_CD4_TCELL_VS_DAY3_IL4	-0,058	0,114	0,00036	1
CYTOKINE_AND_CHEMOKINE_MEDIATED_SIGN	-0,058	0,114	0,14555	1
NUCLEOBASENUCLEOSIDE_AND_NUCLEOTIDE_	-0,058	0,114	0,15966	1
LIU_VAV3_PROSTATE_CARCINOGENESIS_DN	-0,058	0,114	0,38751	1
GSE17721_PAM3CSK4_VS_GADIQUIMOD_6H_	-0,058	0,114	0,01344	1
BIOCARTA_HIVNEF_PATHWAY	-0,058	0,114	0,06239	1
chr12q11	-0,058	0,114	0,04116	1
RAFFEL_VEGFA_TARGETS_UP	-0,058	0,114	0,80817	1
WHITEHURST_PACLITAXEL_SENSITIVITY	-0,058	0,114	0,00176	1
SCHAEFFER_PROSTATE_DEVELOPMENT_AND_	-0,058	0,115	0,00071	1
REACTOME_NETRIN1_SIGNALING	-0,058	0,115	0,01837	1
PEPPER_CHRONIC_LYMPHOCYTIC_LEUKEMIA_U	-0,058	0,115	0,25576	1
REACTOME_SYNTHESIS_OF_PA	-0,058	0,115	0,01401	1
GSE11864_CSF1_PAM3CYS_VS_CSF1_IFNG_	-0,058	0,116	0,08522	1
HO_LIVER_CANCER_VASCULAR_INVASION	-0,058	0,116	0,05254	1
GSE26495_NAIVE_VS_PD1HIGH_CD8_TCELL_	-0,058	0,116	0,1056	1
RAY_TUMORIGENESIS_BY_ERBB2_CDC25A_DN	-0,058	0,116	0,00054	1
REACTOME_MRNA_SPLICING_MINOR_PATHWA	-0,058	0,116	0,75305	1
MIKKELSEN_ES_ICP_WITH_H3K27ME3	-0,058	0,116	0,02626	1
MODULE_171	-0,058	0,116	0,09301	1

GSE14769_UNSTIM_VS_80MIN_LPS_BMDM_D	-0,058	0,116	0,12044	1
NIKOLSKY_BREAST_CANCER_16P13_AMPLICO	0,058	0,116	0,69545	1
SHIRAISHI_PLZF_TARGETS_DN	-0,058	0,117	0,10958	1
REACTOME_P130CAS_LINKAGE_TO_MAPK_SI	-0,057	0,117	0,14206	1
LINDGREN_BLADDER_CANCER_CLUSTER_2A_	-0,057	0,117	0,03866	1
HYDROGEN_ION_TRANSMEMBRANE_TRANSPO	0,057	0,117	0,05155	1
REACTOME_REGULATION_OF_GENE_EXPRESSI	-0,057	0,118	0,08345	1
GTGCCAA,MIR-96	-0,057	0,118	0,0084	1
GSE14769_UNSTIM_VS_360MIN_LPS_BMDM_	-0,057	0,118	0,09091	1
chr2q33	-0,057	0,119	0,05917	1
N_TERMINAL_PROTEIN_AMINO_ACID_MODIFIC	-0,057	0,119	0,56718	1
GSE9988_LPS_VS_LPS_AND_ANTI_TREM1_M	-0,057	0,119	0,15894	1
MIKHAYLOVA_OXIDATIVE_STRESS_RESPONSE	-0,057	0,119	0,69815	1
ZHAN_V1_LATE_DIFFERENTIATION_GENES_UI	0,057	0,119	0,25397	1
RESPONSE_TO_HEAT	0,057	0,119	0,00628	1
AMIT_EGF_RESPONSE_20_MCF10A	-0,057	0,119	0,62483	1
GSE18791_CTRL_VS_NEWCASTLE_VIRUS_DC	-0,057	0,119	0,17461	1
FOURNIER_ACINAR_DEVELOPMENT_EARLY_UF	-0,057	0,119	0,00315	1
GCM_DDX11	-0,057	0,119	0,014	1
IVANOV_MUTATED_IN_COLON_CANCER	-0,057	0,12	0,39318	1
NIKOLSKY_BREAST_CANCER_20P13_AMPLICO	0,057	0,12	0,57697	1
chr6q27	0,057	0,12	0,18874	1
TGFB_UP.V1_DN	-0,057	0,12	0,00722	1
GSE37416_0H_VS_6H_F_TULARENSIS_LVS_	-0,057	0,12	0,53539	1
SCHAEFFER_PROSTATE_DEVELOPMENT_AND_	-0,057	0,12	0,3032	1
GSE12845_PRE_GC_VS_DARKZONE_GC_TON	-0,057	0,12	0,04904	1
NEUROPEPTIDE_BINDING	-0,057	0,12	0,03129	1
REACTOME_LIPID_DIGESTION_MOBILIZATION_	-0,057	0,12	0,01088	1
FALVELLA_SMOKERS_WITH_LUNG_CANCER	-0,057	0,121	0,13678	1
KEGG_NICOTINATE_AND_NICOTINAMIDE_MET/	-0,057	0,121	0,02228	1
ACTIN_POLYMERIZATION_AND_OR_DEPOLYME	-0,057	0,121	0,00198	1
KYNG_WERNER_SYNDROM_AND_NORMAL_AG	-0,057	0,121	0,01481	1
KEGG_PRION_DISEASES	-0,057	0,121	0,04784	1
MODULE_363	-0,057	0,121	0,66541	1
MORF_MYST2	-0,057	0,121	0,02556	1
GSE7460_TCONV_VS_TREG_THYMUS_UP	-0,057	0,121	0,00819	1
REACTOME_MHC_CLASS_II_ANTIGEN_PRESEN	-0,057	0,121	0,45162	1
REACTOME_AUTODEGRADATION_OF_CDH1_B	-0,057	0,121	0,97013	1
CGTCTTA,MIR-208	-0,057	0,121	0,16937	1
GSE14769_UNSTIM_VS_120MIN_LPS_BMDM_	-0,057	0,122	0,07471	1
BIOCARTA_RNA_PATHWAY	-0,057	0,122	0,36914	1
VALK_AML_CLUSTER_1	-0,057	0,122	0,00801	1

BONOME_OVARIAN_CANCER_SURVIVAL_OPTI	-0,057	0,123	0,00169	1
GSE14000_TRANSLATED_RNA_VS_MRNA_16	-0,057	0,123	0,05266	1
GSE3982_BCELL_VS_CENT_MEMORY_CD4_T	-0,057	0,123	0,11499	1
LEE_EARLY_T_LYMPHOCYTE_DN	-0,057	0,123	0,04294	1
EXOCYTOSIS	0,057	0,123	0,86768	1
LU_IL4_SIGNALING	-0,056	0,123	0,09102	1
HOFMANN_MYELODYSPLASTIC_SYNDROM_HIG	-0,056	0,123	0,09302	1
ACEVEDO_NORMAL_TISSUE_ADJACENT_TO_I	-0,056	0,124	0,02684	1
REACTOME_THROMBOXANE_SIGNALLING_THR	-0,056	0,124	0,05295	1
chr2q14	-0,056	0,124	0,30321	1
LAMB_CCND1_TARGETS	-0,056	0,124	0,15006	1
TORCHIA_TARGETS_OF_EWSR1_FLI1_FUSION	-0,056	0,124	0,00072	1
MODULE_495	-0,056	0,124	0,00135	1
BASSO_CD40_SIGNALING_UP	-0,056	0,124	0,15	1
REGULATION_OF_TRANSLATIONAL_INITIATION	-0,056	0,124	0,41166	1
KIM_GERMINAL_CENTER_T_HELPER_DN	-0,056	0,124	0,01035	1
MAHADEVAN_RESPONSE_TO_MP470_DN	-0,056	0,124	0,01863	1
SOTIRIOU_BREAST_CANCER_GRADE_1_VS_3_	0,056	0,124	0,23168	1
SIMBULAN_PARP1_TARGETS_UP	-0,056	0,124	0,00883	1
GSE17721_0.5H_VS_12H_PAM3CSK4_BMDM	-0,056	0,125	0,00954	1
SHARMA_PILOCYTIC_ASTROCYTOMA_LOCATI	-0,056	0,125	0,16035	1
BIOCARTA_GCR_PATHWAY	-0,056	0,125	0,18093	1
GSE3982_MAST_CELL_VS_CENT_MEMORY_C	-0,056	0,125	0,23517	1
GNF2_ITGAL	-0,056	0,125	0,06088	1
MODULE_150	-0,056	0,125	0,03348	1
GSE360_HIGH_DOSE_B_MALAYI_VS_M_TUBE	-0,056	0,125	0,51861	1
LUI_TARGETS_OF_PAX8_PPARG_FUSION	-0,056	0,125	0,19157	1
KIM_ALL_DISORDERS_DURATION_CORR_UP	-0,056	0,125	0,20554	1
HORMONE_METABOLIC_PROCESS	-0,056	0,125	0,01749	1
GNF2_CD7	-0,056	0,126	0,06372	1
GSE13484_UNSTIM_VS_3H_YF17D_VACCINE	-0,056	0,126	0,35616	1
HOSHIDA_LIVER_CANCER_SURVIVAL_DN	0,056	0,126	0,66749	1
BIOCARTA_FAS_PATHWAY	-0,056	0,126	0,19774	1
DI___TRI_VALENT_INORGANIC_CATION_TRAN	-0,056	0,126	0,03941	1
MYLLYKANGAS_AMPLIFICATION_HOT_SPOT_2	-0,056	0,126	0,02095	1
GAVIN_FOXP3_TARGETS_CLUSTER_T4	-0,056	0,126	0,16587	1
CELLULAR_RESPONSE_TO_STRESS	-0,056	0,126	0,23264	1
MORF_IKBKG	-0,056	0,126	0,01341	1
GSE22886_NAIVE_TCELL_VS_NEUTROPHIL_D	-0,056	0,127	0,28815	1
CARDOSO_RESPONSE_TO_GAMMA_RADIATIOI	-0,056	0,127	0,01239	1
REACTOME_ABORTIVE_ELONGATION_OF_HIV	-0,056	0,127	0,55804	1
GSE29614_CTRL_VS_DAY3_TIV_FLU_VACCII	-0,056	0,127	0,00421	1

VANHARANTA_UTERINE_FIBROID_WITH_7Q_[	-0,056	0,127	0,11056	1
NEGATIVE_REGULATION_OF_HYDROLASE_AC	-0,056	0,127	0,58236	1
CUI_TCF21_TARGETS_2_DN	-0,056	0,127	0,0188	1
OVULATION_CYCLE	-0,056	0,127	0,30374	1
GSE14769_UNSTIM_VS_60MIN_LPS_BMDM_[	-0,056	0,127	0,14147	1
REACTOME_PYRUVATE_METABOLISM	-0,056	0,127	0,80503	1
MODULE_208	-0,056	0,127	0,09135	1
BCAT.100_UP.V1_UP	-0,056	0,127	0,01637	1
CHOW_RASSF1_TARGETS_DN	-0,056	0,127	0,25877	1
MIKKELSEN_IPS_HCP_WITH_H3_UNMETHYLAT	-0,056	0,127	0,00137	1
chr11q25	-0,056	0,128	0,00503	1
REACTOME_COMMON_PATHWAY	-0,056	0,128	0,0452	1
REACTOME_HS_GAG_DEGRADATION	-0,056	0,128	0,02353	1
REACTOME_NEF_MEDIATES_DOWN_MODULAT	-0,056	0,128	0,22114	1
V$STAT6_02	-0,056	0,128	0,00209	1
MTOR_UP.N4.V1_DN	-0,056	0,128	0,00028	1
GSE17721_CTRL_VS_GARDIQUIMOD_4H_BMD	-0,056	0,129	0,00025	1
REACTOME_N_GLYCAN_ANTENNAE_ELONGAT	-0,056	0,129	0,14075	1
IMMUNE_RESPONSE	-0,056	0,129	0,13138	1
PID_NETRIN_PATHWAY	-0,056	0,129	0,25836	1
CELLULAR_LOCALIZATION	-0,056	0,129	0,08578	1
WANG_LMO4_TARGETS_DN	-0,056	0,129	0,06395	1
MODULE_436	-0,056	0,129	0,09028	1
JAERVINEN_AMPLIFIED_IN_LARYNGEAL_CANC	0,056	0,129	0,30735	1
SHARMA_ASTROCYTOMA_WITH_NF1_SYNDRC	-0,056	0,129	0,00199	1
GSE17974_IL4_AND_ANTI_IL12_VS_UNTREA	-0,056	0,129	0,00249	1
GENTILE_UV_RESPONSE_CLUSTER_D9	-0,056	0,13	0,03679	1
MODULE_299	-0,056	0,13	0,7185	1
SENGUPTA_EBNA1_ANTICORRELATED	-0,056	0,13	0,01248	1
CELLULAR_PROTEIN_COMPLEX_ASSEMBLY	-0,055	0,13	0,03354	1
LEE_LIVER_CANCER_ACOX1_DN	-0,055	0,13	0,06144	1
chr2q24	-0,055	0,13	0,05048	1
KAAB_FAILED_HEART_ATRIUM_DN	-0,055	0,131	0,4234	1
REACTOME_ACTIVATION_OF_CHAPERONES_B	0,055	0,131	0,05073	1
GSE11864_UNTREATED_VS_CSF1_IFNG_PAM	-0,055	0,131	0,01086	1
chr14q22	-0,055	0,131	0,03754	1
GSE17721_CTRL_VS_PAM3CSK4_4H_BMDM_	-0,055	0,131	0,01634	1
WCAANNNYCAG_UNKNOWN	-0,055	0,131	0,00034	1
chr11p13	-0,055	0,131	0,52507	1
ZHANG_TLX_TARGETS_36HR_UP	-0,055	0,131	0,00523	1
ELLWOOD_MYC_TARGETS_DN	0,055	0,131	0,02701	1
GSE14308_TH1_VS_TH17_DN	-0,055	0,131	0,00207	1

AMIT_DELAYED_EARLY_GENES	-0,055	0,131	0,01016	1
GSE13411_PLASMA_CELL_VS_MEMORY_BCEI	-0,055	0,131	0,03579	1
REACTOME_DESTABILIZATION_OF_MRNA_BY_	-0,055	0,132	0,87259	1
VALK_AML_WITH_CEBPA	-0,055	0,132	0,1098	1
DER_IFN_BETA_RESPONSE_UP	-0,055	0,132	0,33705	1
CAFFAREL_RESPONSE_TO_THC_8HR_3_DN	-0,055	0,132	0,68522	1
V$AHRARNT_02	-0,055	0,132	0,01881	1
PROTEIN_SERINE_THREONINE_PHOSPHATASE_	-0,055	0,132	0,03311	1
GSE13306_TREG_VS_TCONV_UP	-0,055	0,132	0,00297	1
T_CELL_DIFFERENTIATION	-0,055	0,133	0,11953	1
chr3q27	-0,055	0,133	0,38227	1
MARIADASON_RESPONSE_TO_CURCUMIN_SUL	-0,055	0,133	0,45313	1
GSE17721_0.5H_VS_4H_PAM3CSK4_BMDM_	-0,055	0,133	0,06384	1
GAGCTGG,MIR-337	-0,055	0,133	0,00192	1
SCHLINGEMANN_SKIN_CARCINOGENESIS_TPA_	-0,055	0,133	0,73425	1
chr5p12	-0,055	0,134	0,18641	1
POSITIVE_REGULATION_OF_CYTOSKELETON_(	-0,055	0,134	0,00216	1
DIRMEIER_LMP1_RESPONSE_LATE_DN	-0,055	0,134	0,17516	1
chr1q31	-0,055	0,134	0,16503	1
KEGG_INOSITOL_PHOSPHATE_METABOLISM	-0,055	0,134	0,02143	1
BIOCARTA_HER2_PATHWAY	0,055	0,134	0,98876	1
chr7q33	-0,055	0,135	0,85658	1
GCM_MYST2	-0,055	0,135	0,1676	1
WANG_CLIM2_TARGETS_DN	-0,055	0,135	0,54985	1
REACTOME_TAK1_ACTIVATES_NFKB_BY_PHC	-0,055	0,135	0,33846	1
GSE3982_CENT_MEMORY_CD4_TCELL_VS_TI	-0,055	0,135	0,00374	1
LINDSTEDT_DENDRITIC_CELL_MATURATION_/	-0,055	0,135	0,56657	1
MODULE_114	-0,055	0,136	0,2096	1
GSE17974_CTRL_VS_ACT_IL4_AND_ANTI_IL	-0,055	0,136	0,00891	1
GSE14000_4H_VS_16H_LPS_DC_TRANSLATI	-0,055	0,136	0,05157	1
ZHAN_MULTIPLE_MYELOMA_MF_DN	-0,055	0,137	0,11942	1
GSE15930_NAIVE_VS_24H_IN_VITRO_STIM_	-0,055	0,137	0,00741	1
GSE360_L_DONOVANI_VS_M_TUBERCULOSIS	-0,054	0,137	0,07939	1
BIOCARTA_RAB_PATHWAY	-0,054	0,138	0,4767	1
GSE24142_DN2_VS_DN3_THYMOCYTE_DN	-0,054	0,138	0,00522	1
WENDT_COHESIN_TARGETS_UP	-0,054	0,138	0,36177	1
BCAT.100_UP.V1_DN	-0,054	0,138	0,00429	1
GSE17974_0H_VS_1H_IN_VITRO_ACT_CD4_	-0,054	0,138	0,01163	1
THYROID_HORMONE_RECEPTOR_BINDING	-0,054	0,138	0,43816	1
OXFORD_RALB_TARGETS_UP	-0,054	0,139	0,06328	1
BIOCARTA_INFLAM_PATHWAY	-0,054	0,139	0,26913	1
GSE3982_DC_VS_BCELL_UP	-0,054	0,139	0,18921	1

CYTOCHROME_C_OXIDASE_ACTIVITY	0,054	0,139	0,32525	1
LI_CYTIDINE_ANALOG_PATHWAY	-0,054	0,139	0,73879	1
MODULE_345	-0,054	0,139	0,12666	1
GSE17721_LPS_VS_POLYIC_2H_BMDM_DN	-0,054	0,139	0,01693	1
KEGG_APOPTOSIS	-0,054	0,139	0,19556	1
POSITIVE_REGULATION_OF_PROTEIN_AMINO_	-0,054	0,139	0,0072	1
GSE30083_SP3_VS_SP4_THYMOCYTE_DN	-0,054	0,139	0,0939	1
PID_NFKAPPABATYPICALPATHWAY	-0,054	0,14	0,09909	1
GSE360_L_DONOVANI_VS_B_MALAYI_LOW_	-0,054	0,14	0,069	1
ICOSANOID_METABOLIC_PROCESS	-0,054	0,141	0,01963	1
YOSHIMURA_MAPK8_TARGETS_DN	-0,054	0,141	0,08355	1
MODULE_427	-0,054	0,141	0,00017	1
CLATHRIN_COATED_VESICLE	-0,054	0,142	0,18645	1
LI_INDUCED_T_TO_NATURAL_KILLER_UP	-0,054	0,142	0,08988	1
GSE360_DC_VS_MAC_B_MALAYI_HIGH_DOSE	-0,054	0,142	0,20082	1
CGCTGCT,MIR-503	-0,054	0,142	0,04142	1
VARELA_ZMPSTE24_TARGETS_DN	-0,054	0,142	0,01475	1
KEGG_GLYCEROPHOSPHOLIPID_METABOLISM	-0,054	0,142	0,00789	1
REACTOME_CYTOSOLIC_TRNA_AMINOACYLAT	-0,054	0,142	0,31614	1
MCLACHLAN_DENTAL_CARIES_DN	-0,054	0,142	0,27846	1
MODULE_440	-0,054	0,142	0,4089	1
TAKEDA_TARGETS_OF_NUP98_HOXA9_FUSIC	-0,054	0,142	0,55478	1
GSE14350_TREG_VS_TEFF_IN_IL2RB_KO_UP	-0,054	0,143	0,02878	1
GAUSSMANN_MLL_AF4_FUSION_TARGETS_B_	0,054	0,143	0,36558	1
BIOCARTA_EXTRINSIC_PATHWAY	-0,054	0,143	0,0046	1
GSE360_T_GONDII_VS_B_MALAYI_LOW_DOS	-0,054	0,143	0,03172	1
WIEMANN_TELOMERE_SHORTENING_AND_CHI	-0,054	0,143	0,33698	1
GSE17974_IL4_AND_ANTI_IL12_VS_UNTREA	-0,054	0,143	0,0064	1
GSE17721_POLYIC_VS_PAM3CSK4_24H_BMI	-0,054	0,143	0,18655	1
MODULE_134	-0,054	0,144	0,11762	1
MODULE_177	-0,054	0,144	0,25186	1
GSE360_CTRL_VS_B_MALAYI_HIGH_DOSE_M	-0,054	0,144	0,10701	1
chr6q16	-0,054	0,144	0,78098	1
REGULATION_OF_SMALL_GTPASE_MEDIATED_	-0,054	0,144	0,07485	1
GNF2_HPX	0,054	0,144	0,70023	1
chrxp11	0,054	0,145	0,14804	1
PID_CONE_PATHWAY	-0,053	0,145	0,06126	1
ST_TUMOR_NECROSIS_FACTOR_PATHWAY	-0,053	0,145	0,24654	1
ABDULRAHMAN_KIDNEY_CANCER_VHL_UP	-0,053	0,145	0,70567	1
REGULATION_OF_CELL_SHAPE	-0,053	0,145	0,07607	1
KRASNOSELSKAYA_ILF3_TARGETS_UP	-0,053	0,145	0,15691	1
PID_IL5_PATHWAY	-0,053	0,146	0,12787	1

GSE9988_LPS_VS_CTRL_TREATED_MONOCY	-0,053	0,146	0,09678	1
KEGG_FOLATE_BIOSYNTHESIS	-0,053	0,146	0,23042	1
MODULE_326	-0,053	0,146	0,02624	1
HAHTOLA_MYCOSIS_FUNGOIDES_UP	-0,053	0,146	0,59644	1
REGULATION_OF_INTERFERON_GAMMA_BIOSY	-0,053	0,146	0,19196	1
GSE360_CTRL_VS_M_TUBERCULOSIS_MAC_L	-0,053	0,146	0,02362	1
NUCLEAR_EXPORT	-0,053	0,146	0,37581	1
GSE20366_CD103_KLRG1_DP_VS_DN_TREG	-0,053	0,146	0,06089	1
BIOCARTA_IGF1R_PATHWAY	-0,053	0,146	0,00277	1
GTAGGCA,MIR-189	-0,053	0,146	0,17935	1
VESICULAR_FRACTION	0,053	0,147	0,42084	1
GSE3982_EOSINOPHIL_VS_EFF_MEMORY_CD4	-0,053	0,147	0,15337	1
FARMER_BREAST_CANCER_CLUSTER_7	0,053	0,147	0,64516	1
KEGG_SNARE_INTERACTIONS_IN_VESICULAR_	0,053	0,147	0,3447	1
REACTOME_REGULATORY_RNA_PATHWAYS	-0,053	0,147	0,4803	1
KERLEY_RESPONSE_TO_CISPLATIN_UP	-0,053	0,147	0,00241	1
PID_IL27PATHWAY	-0,053	0,147	0,13897	1
LEE_DIFFERENTIATING_T_LYMPHOCYTE	-0,053	0,147	0,03854	1
GSE17721_LPS_VS_POLYIC_24H_BMDM_DN	-0,053	0,147	0,02338	1
LEE_CALORIE_RESTRICTION_MUSCLE_DN	-0,053	0,147	0,39124	1
BIOCARTA_TCRA_PATHWAY	-0,053	0,147	0,09698	1
BIOCARTA_DEATH_PATHWAY	-0,053	0,148	0,05916	1
FATTY_ACID_METABOLIC_PROCESS	-0,053	0,148	0,00085	1
GSE29614_CTRL_VS_TIV_FLU_VACCINE_PBM	-0,053	0,148	0,00134	1
P53_DN.V2_DN	-0,053	0,148	0,00596	1
VALK_AML_CLUSTER_16	-0,053	0,148	0,00038	1
BIOCARTA_CSK_PATHWAY	-0,053	0,148	0,07939	1
MIKKELSEN_NPC_ICP_WITH_H3K27ME3	0,053	0,148	0,55467	1
ATCTTGC,MIR-31	-0,053	0,148	0,04369	1
V$ICSBP_Q6	-0,053	0,149	0,0584	1
ELVIDGE_HIF1A_TARGETS_DN	-0,053	0,149	0,80168	1
KEGG_TERPENOID_BACKBONE_BIOSYNTHESIS	0,053	0,149	0,10894	1
PID_IL12_2PATHWAY	-0,053	0,149	0,17924	1
PID_TNFPATHWAY	-0,053	0,149	0,12485	1
PROTEIN_TETRAMERIZATION	-0,053	0,149	0,00306	1
GSE14000_UNSTIM_VS_4H_LPS_DC_DN	-0,053	0,149	0,40529	1
CHIANG_LIVER_CANCER_SUBCLASS_UNANNO	-0,053	0,149	0,60185	1
GSE6269_E_COLI_VS_STREP_PNEUMO_INF_P	-0,053	0,15	0,41727	1
AMIT_EGF_RESPONSE_40_MCF10A	-0,053	0,15	0,00621	1
MODULE_86	-0,053	0,15	0,28007	1
REACTOME_FATTY_ACID_TRIACYLGLYCEROL_	-0,053	0,15	0,09487	1
CHYLA_CBFA2T3_TARGETS_UP	-0,053	0,151	0,00863	1

GSE14000_UNSTIM_VS_16H_LPS_DC_UP	-0,053	0,151	0,0656	1
MODULE_180	0,053	0,151	0,48718	1
GNF2_RAD23A	-0,053	0,151	0,18009	1
PID_IFNGPATHWAY	-0,053	0,151	0,314	1
RODWELL_AGING_KIDNEY_NO_BLOOD_DN	-0,053	0,151	0,00385	1
YAGI_AML_WITH_T_9_11_TRANSLOCATION	-0,053	0,151	0,00602	1
LEE_CALORIE_RESTRICTION_MUSCLE_UP	-0,053	0,151	0,10749	1
MODULE_233	-0,053	0,151	0,89738	1
MODULE_76	-0,053	0,152	0,38934	1
KRAS.KIDNEY_UP.V1_DN	-0,053	0,152	0,03166	1
SANSOM_APC_TARGETS_DN	-0,053	0,152	0,02563	1
GSE14000_TRANSLATED_RNA_VS_MRNA_4H	-0,053	0,152	0,10057	1
BIOCARTA_IL17_PATHWAY	-0,053	0,152	0,09109	1
REACTOME_EFFECTS_OF_PIP2_HYDROLYSIS	-0,052	0,153	0,02598	1
YEGNASUBRAMANIAN_PROSTATE_CANCER	-0,052	0,153	0,10731	1
INTERLEUKIN_1_SECRETION	0,052	0,153	0,90078	1
WU_HBX_TARGETS_2_DN	-0,052	0,153	0,47905	1
chr1p11	-0,052	0,153	0,40425	1
NEUROTRANSMITTER_SECRETION	0,052	0,153	0,738	1
ANCHORED_TO_MEMBRANE	-0,052	0,153	0,0435	1
ANCHORED_TO_PLASMA_MEMBRANE	-0,052	0,153	0,0435	1
GSE1432_CTRL_VS_IFNG_1H_MICROGLIA_DN	-0,052	0,153	0,11222	1
GSE17721_POLYIC_VS_PAM3CSK4_4H_BMDM	-0,052	0,153	0,03739	1
chr22q13	-0,052	0,154	0,22662	1
GSE3982_DC_VS_MAC_UP	-0,052	0,154	0,02574	1
KIM_ALL_DISORDERS_DURATION_CORR_DN	-0,052	0,154	0,25937	1
CALVET_IRINOTECAN_SENSITIVE_VS_REVERT	-0,052	0,154	0,00019	1
POSITIVE_REGULATION_OF_MAP_KINASE_ACT	-0,052	0,154	0,17503	1
INTERFERON_GAMMA_BIOSYNTHETIC_PROCES	-0,052	0,154	0,20429	1
BIOCARTA_IL3_PATHWAY	-0,052	0,154	0,01787	1
BIOCARTA_TCYTOTOXIC_PATHWAY	-0,052	0,154	0,10951	1
TRNA_PROCESSING	-0,052	0,155	0,7296	1
GCM_DFFA	-0,052	0,155	0,17741	1
MODULE_84	-0,052	0,155	0,12513	1
GTGTTGA,MIR-505	-0,052	0,155	0,06902	1
chr10q23	-0,052	0,155	0,00614	1
GARGALOVIC_RESPONSE_TO_OXIDIZED_PHOS	-0,052	0,155	0,01286	1
GSE17721_CTRL_VS_GARDIQUIMOD_4H_BMD	-0,052	0,155	0,09926	1
MODULE_42	0,052	0,155	0,14739	1
GSE17721_4_VS_24H_CPG_BMDM_UP	-0,052	0,155	0,07595	1
BRACHAT_RESPONSE_TO_CISPLATIN	0,052	0,156	0,59486	1
LYSOSOMAL_TRANSPORT	0,052	0,156	0,56963	1

CHAN_INTERFERON_PRODUCING_DENDRITIC_(	-0,052	0,156	0,13377	1
FUJIWARA_PARK2_IN_LIVER_CANCER_UP	-0,052	0,156	0,16916	1
GSE27786_BCELL_VS_NKTCELL_DN	-0,052	0,157	0,10351	1
REACTOME_HIGHLY_CALCIUM_PERMEABLE_P(	-0,052	0,157	0,42117	1
REGULATION_OF_PH	0,052	0,157	0,67929	1
REGULATION_OF_JNK_ACTIVITY	-0,052	0,157	0,17222	1
PID_IL12_STAT4PATHWAY	-0,052	0,157	0,13896	1
chr2p11	-0,052	0,157	0,29987	1
HOLLEMAN_ASPARAGINASE_RESISTANCE_ALI	-0,052	0,157	0,2937	1
GSE3982_MEMORY_CD4_TCELL_VS_TH1_UP	-0,052	0,157	0,00099	1
GSE6269_E_COLI_VS_STREP_AUREUS_INF_PI	-0,052	0,157	0,38007	1
GTPASE_BINDING	-0,052	0,157	0,24234	1
GSE3982_DC_VS_NKCELL_UP	-0,052	0,158	0,26827	1
GSE12366_NAIVE_VS_MEMORY_BCELL_DN	-0,052	0,158	0,04593	1
DASU_IL6_SIGNALING_DN	-0,052	0,158	0,02716	1
ATTACAT,MIR-380-3P	-0,052	0,158	0,14513	1
GSE36392_EOSINOPHIL_VS_NEUTROPHIL_IL2	-0,052	0,158	0,03935	1
GCM_HBP1	-0,052	0,158	0,39421	1
GSE22886_DC_VS_MONOCYTE_DN	-0,052	0,158	0,10063	1
RESPONSE_TO_BIOTIC_STIMULUS	-0,052	0,159	0,25065	1
REGULATION_OF_TRANSPORT	-0,052	0,159	0,01169	1
TAKEDA_TARGETS_OF_NUP98_HOXA9_FUSIC	-0,052	0,159	0,30663	1
ENDOPLASMIC_RETICULUM	-0,052	0,159	0,28348	1
DALESSIO_TSA_RESPONSE	-0,052	0,159	0,09224	1
ORGANELLE_MEMBRANE	-0,052	0,159	0,63854	1
chr11q12	-0,052	0,159	0,16945	1
PARK_OSTEOBLAST_DIFFERENTIATION_BY_PH	-0,052	0,159	0,40853	1
LOPEZ_MESOTHELIOMA_SURVIVAL_OVERALL_	-0,052	0,159	0,24052	1
GCM_MAP4K4	-0,052	0,159	0,14516	1
GSE26928_EFF_MEM_VS_CENTR_MEM_CD4_	-0,052	0,16	0,00125	1
MIZUKAMI_HYPOXIA_UP	-0,052	0,16	0,17567	1
CAR_TNFRSF25	-0,052	0,16	0,16259	1
GSE3982_MAST_CELL_VS_MAC_DN	-0,052	0,16	0,43967	1
PETRETTO_BLOOD_PRESSURE_DN	0,052	0,16	0,78501	1
REACTOME_MYD88_MAL_CASCADE_INITIATEI	-0,052	0,16	0,18221	1
TSAI_RESPONSE_TO_RADIATION_THERAPY	-0,051	0,16	0,01239	1
GARGALOVIC_RESPONSE_TO_OXIDIZED_PHOS	-0,051	0,161	0,5017	1
WENG_POR_DOSAGE	0,051	0,161	0,32978	1
INSULIN_RECEPTOR_SIGNALING_PATHWAY	-0,051	0,161	0,00025	1
GSE36476_CTRL_VS_TSST_ACT_16H_MEMO	-0,051	0,161	0,01126	1
TERAMOTO_OPN_TARGETS_CLUSTER_4	0,051	0,161	0,17035	1
ZEMBUTSU_SENSITIVITY_TO_CISPLATIN	-0,051	0,161	0,04417	1

ESC_V6.5_UP_EARLY.V1_UP	-0,051	0,161	0,00096	1
GSE17721_POLYIC_VS_GARDIQUIMOD_16H_I	-0,051	0,161	0,02693	1
GSE17721_CPG_VS_GARDIQUIMOD_2H_BMDN	-0,051	0,162	0,11782	1
REACTOME_SHC_MEDIATED_SIGNALLING	-0,051	0,162	0,33555	1
REACTOME_FGFR_LIGAND_BINDING_AND_AC	-0,051	0,162	0,00797	1
GSE17721_LPS_VS_GARDIQUIMOD_16H_BMD	-0,051	0,162	0,03757	1
chr8p22	-0,051	0,162	0,04534	1
GSE360_LOW_DOSE_B_MALAYI_VS_M_TUBE	-0,051	0,162	0,74949	1
PID_FCER1PATHWAY	-0,051	0,162	0,02089	1
GNF2_CASP4	-0,051	0,162	0,15536	1
LEI_HOXC8_TARGETS_UP	-0,051	0,162	0,90775	1
GOLDRATH_NAIVE_VS_MEMORY_CD8_TCELL_	-0,051	0,163	0,10792	1
MODULE_489	-0,051	0,163	0,42712	1
MARIADASON_REGULATED_BY_HISTONE_ACE	-0,051	0,163	0,00368	1
GEORGANTAS_HSC_MARKERS	-0,051	0,163	0,01005	1
MODULE_343	0,051	0,164	0,57432	1
GNF2_LCAT	0,051	0,164	0,6126	1
GSE27786_NKCELL_VS_ERYTHROBLAST_UP	-0,051	0,164	0,05801	1
chr6q12	-0,051	0,164	0,37961	1
MODULE_540	-0,051	0,164	0,11194	1
GSE27786_CD8_TCELL_VS_NKCELL_DN	-0,051	0,165	0,08863	1
GOERING_BLOOD_HDL_CHOLESTEROL_QTL_C	-0,051	0,165	0,08824	1
chr3p26	-0,051	0,166	0,00078	1
APOPTOTIC_MITOCHONDRIAL_CHANGES	-0,051	0,166	0,27976	1
SYNAPTOGENESIS	-0,051	0,166	0,42737	1
GSE7852_LN_VS_FAT_TCONV_UP	-0,051	0,166	0,03182	1
BIOCARTA_CTLA4_PATHWAY	-0,051	0,166	0,1757	1
GSE7852_TREG_VS_TCONV_FAT_UP	-0,051	0,167	0,03961	1
ATGTAGC,MIR-221,MIR-222	-0,051	0,167	0,00535	1
BIOCARTA_FIBRINOLYSIS_PATHWAY	-0,051	0,167	0,20027	1
SHEN_SMARCA2_TARGETS_UP	0,051	0,167	0,26374	1
GSE27786_LIN_NEG_VS_CD4_TCELL_DN	-0,051	0,167	0,03097	1
LOPEZ_MESOTHELIOMA_SURVIVAL_OVERALL_	-0,051	0,167	0,00624	1
BECKER_TAMOXIFEN_RESISTANCE_UP	-0,051	0,167	0,00153	1
PID_NCADHERINPATHWAY	-0,051	0,168	0,0819	1
OXIDOREDUCTASE_ACTIVITY_ACTING_ON_CH	-0,051	0,168	0,09405	1
TASTE_RECEPTOR_ACTIVITY	-0,051	0,168	0,09471	1
GSE26495_NAIVE_VS_PD1LOW_CD8_TCELL_	-0,051	0,168	0,13294	1
REACTOME_TRIF_MEDIATED_TLR3_SIGNALING	-0,05	0,169	0,12869	1
PROTEIN_AMINO_ACID_ADP_RIBOSYLATION	-0,05	0,169	0,94445	1
GSE39820_TGFBETA1_VS_TGFBETA3_IN_IL6	-0,05	0,17	0,02568	1
SPIRA_SMOKERS_LUNG_CANCER_UP	-0,05	0,17	0,12649	1

BIOCARTA_RELA_PATHWAY	0,05	0,17	0,3503	1
GSE17974_0H_VS_0.5H_IN_VITRO_ACT_CD4	-0,05	0,17	0,01124	1
BOYAULT_LIVER_CANCER_SUBCLASS_G1_UP	-0,05	0,17	0,05853	1
COFACTOR_BIOSYNTHETIC_PROCESS	0,05	0,17	0,01513	1
SECONDARY_ACTIVE_TRANSMEMBRANE_TRAI	-0,05	0,171	0,77135	1
BIOCARTA_GLYCOLYSIS_PATHWAY	-0,05	0,171	0,61168	1
GSE10239_KLRG1INT_VS_KLRG1HIGH_EFF_C	-0,05	0,171	0,09883	1
GSE8384_CTRL_VS_B_ABORTUS_4H_MAC_C	-0,05	0,171	0,24089	1
BIOCARTA_CARM1_PATHWAY	0,05	0,171	0,55053	1
GOUYER_TATI_TARGETS_DN	-0,05	0,171	0,03454	1
REACTOME_ELONGATION_ARREST_AND_RECO	-0,05	0,171	0,71756	1
GNF2_HPN	0,05	0,171	0,65187	1
STEROID_DEHYDROGENASE_ACTIVITY_ACTING	0,05	0,172	0,28704	1
CAVARD_LIVER_CANCER_MALIGNANT_VS_BE	-0,05	0,172	0,31203	1
BIOCARTA_AKAPCENTROSOME_PATHWAY	-0,05	0,172	0,30362	1
PEPTIDE_RECEPTOR_ACTIVITY	-0,05	0,172	0,01282	1
LEE_AGING_MUSCLE_DN	-0,05	0,173	0,01809	1
TSUNODA_CISPLATIN_RESISTANCE_DN	-0,05	0,173	0,12038	1
MODULE_399	-0,05	0,173	0,25575	1
GSE24142_EARLY_THYMIC_PROGENITOR_VS_	-0,05	0,173	0,05099	1
MODULE_307	0,05	0,173	0,15405	1
SA_B_CELL_RECEPTOR_COMPLEXES	-0,05	0,173	0,02765	1
PID_GMCSF_PATHWAY	-0,05	0,173	0,23389	1
MITOCHONDRIAL_TRANSPORT	-0,05	0,173	0,48374	1
chr11q24	-0,05	0,173	0,20057	1
PID_HNF3APATHWAY	0,05	0,174	0,10943	1
GSE14308_TH17_VS_INDUCED_TREG_UP	-0,05	0,174	0,03517	1
LEIN_PONS_MARKERS	-0,05	0,174	0,04794	1
KIM_ALL_DISORDERS_CALB1_CORR_DN	-0,05	0,174	0,06256	1
REACTOME_PYRIMIDINE_CATABOLISM	-0,05	0,174	0,14751	1
GSE17974_IL4_AND_ANTI_IL12_VS_UNTREA	-0,05	0,174	0,13384	1
REACTOME_REGULATION_OF_COMPLEMENT_C	0,05	0,174	0,41775	1
MEISSNER_NPC_HCP_WITH_H3K27ME3	-0,05	0,175	0,18544	1
REACTOME_ACETYLCHOLINE_NEUROTRANSMIT	0,05	0,175	0,82577	1
GSE17721_0.5H_VS_4H_CPG_BMDM_DN	-0,05	0,175	0,07926	1
NITROGEN_COMPOUND_METABOLIC_PROCESS	-0,05	0,175	0,06308	1
SUH_COEXPRESSED_WITH_ID1_AND_ID2_UP	0,05	0,176	0,34858	1
LEE_LIVER_CANCER_MYC_E2F1_DN	-0,05	0,176	0,30612	1
MORF_MYL3	-0,05	0,176	0,00701	1
PID_INTEGRIN5_PATHWAY	-0,05	0,176	0,42956	1
chr9q22	-0,05	0,176	0,01968	1
GSE7460_WT_VS_FOXP3_HET_ACT_TCONV_	-0,05	0,176	0,00013	1

ELVIDGE_HIF1A_AND_HIF2A_TARGETS_DN	-0,05	0,176	0,54515	1
SANCHEZ_MDM2_TARGETS	0,05	0,177	0,49588	1
CHEN_LVAD_SUPPORT_OF_FAILING_HEART_[	-0,05	0,177	0,09367	1
GROSS_HYPOXIA_VIA_ELK3_AND_HIF1A_DN	-0,05	0,177	0,20389	1
WESTON_VEGFA_TARGETS_6HR	-0,049	0,177	0,01014	1
VISALA_AGING_LYMPHOCYTE_UP	-0,049	0,177	0,202	1
GSE6269_FLU_VS_E_COLI_INF_PBMC_UP	-0,049	0,177	0,28577	1
GSE27786_NEUTROPHIL_VS_MONO_MAC_UP	-0,049	0,178	0,00796	1
GENTILE_UV_LOW_DOSE_UP	-0,049	0,178	0,07924	1
MODULE_62	0,049	0,178	0,14543	1
PID_CIRCADIANPATHWAY	-0,049	0,178	0,01817	1
THUM_SYSTOLIC_HEART_FAILURE_UP	-0,049	0,178	0,102	1
GSE360_L_MAJOR_VS_B_MALAYI_LOW_DOS	-0,049	0,178	0,11883	1
BIOCARTA_TCAPOPTOSIS_PATHWAY	-0,049	0,178	0,14019	1
FUCOSYLTRANSFERASE_ACTIVITY	-0,049	0,178	0,69919	1
GSE11924_TFH_VS_TH2_CD4_TCELL_UP	-0,049	0,178	0,00027	1
MODULE_132	0,049	0,178	0,16425	1
NAKAMURA_ADIPOGENESIS_LATE_UP	-0,049	0,178	0,00155	1
PROTEIN_C_TERMINUS_BINDING	-0,049	0,178	0,00258	1
ZHANG_RESPONSE_TO_IKK_INHIBITOR_AND_	-0,049	0,178	0,00384	1
ZHAN_MULTIPLE_MYELOMA_PR_DN	-0,049	0,178	0,0463	1
GSE29617_CTRL_VS_TIV_FLU_VACCINE_PBM	-0,049	0,178	0,9186	1
MITOCHONDRION_ORGANIZATION_AND_BIOGE	-0,049	0,179	0,37596	1
VACUOLAR_TRANSPORT	0,049	0,179	0,64773	1
GSE31082_DN_VS_CD4_SP_THYMOCYTE_DN	-0,049	0,179	0,00868	1
TOMLINS_METASTASIS_DN	0,049	0,179	0,73944	1
PID_HIF1_TFPATHWAY	-0,049	0,18	0,17266	1
MONOOXYGENASE_ACTIVITY	-0,049	0,18	0,08347	1
SARTIPY_BLUNTED_BY_INSULIN_RESISTANCE_	-0,049	0,18	0,00026	1
MODULE_486	-0,049	0,18	0,0319	1
HUMORAL_IMMUNE_RESPONSE	-0,049	0,18	0,13049	1
MODULE_101	0,049	0,18	0,18352	1
DACOSTA_LOW_DOSE_UV_RESPONSE_VIA_E	-0,049	0,181	0,12871	1
STARK_PREFRONTAL_CORTEX_22Q11_DELET	-0,049	0,181	0,02371	1
REACTOME_HORMONE_LIGAND_BINDING_RECE	-0,049	0,181	0,03788	1
DEURIG_T_CELL_PROLYMPHOCYTIC_LEUKEMIA	-0,049	0,181	0,10694	1
VALK_AML_CLUSTER_3	-0,049	0,181	0,0504	1
IMMUNE_EFFECTOR_PROCESS	-0,049	0,181	0,10943	1
EHLERS_ANEUPLOIDY_UP	-0,049	0,181	0,0036	1
CYCLASE_ACTIVITY	-0,049	0,181	0,05594	1
PHOSPHORUS_OXYGEN_LYASE_ACTIVITY	-0,049	0,181	0,05594	1
MYLLYKANGAS_AMPLIFICATION_HOT_SPOT_1	-0,049	0,182	0,00616	1

RYAAAKNNNNNNTTGW_UNKNOWN	-0,049	0,182	0,01586	1
FERRANDO_LYL1_NEIGHBORS	-0,049	0,182	0,00482	1
NIELSEN_SYNOVIAL_SARCOMA_DN	0,049	0,182	0,55109	1
REACTOME_REGULATION_OF_INSULIN_LIKE_G	-0,049	0,182	0,00095	1
SH2_DOMAIN_BINDING	-0,049	0,182	0,06088	1
GSE18791_UNSTIM_VS_NEWCATSLE_VIRUS_	-0,049	0,182	0,3454	1
GSE13485_DAY3_VS_DAY7_YF17D_VACCINI	-0,049	0,183	0,42573	1
REACTOME_OTHER_SEMAPHORIN_INTERACTIC	-0,049	0,183	0,05001	1
THUM_SYSTOLIC_HEART_FAILURE_DN	-0,049	0,183	0,00737	1
WOO_LIVER_CANCER_RECURRENCE_DN	0,049	0,183	0,37924	1
LINDSTEDT_DENDRITIC_CELL_MATURATION_[	-0,049	0,183	0,20233	1
GNF2_SPRR1B	-0,049	0,183	0,63158	1
REACTOME_IRAK2_MEDIATED_ACTIVATION_C	-0,049	0,183	0,29756	1
WARTERS_IR_RESPONSE_5GY	-0,049	0,183	0,00222	1
GSE17974_IL4_AND_ANTI_IL12_VS_UNTREA	-0,049	0,183	0,28737	1
chr1q42	-0,049	0,183	0,23895	1
ESTABLISHMENT_OF_CELLULAR_LOCALIZATIC	-0,049	0,183	0,11217	1
POTTI_ETOPOSIDE_SENSITIVITY	-0,049	0,183	0,31463	1
TCCAGAG,MIR-518C	-0,049	0,184	0,03798	1
GSE13485_DAY7_VS_DAY21_YF17D_VACCII	-0,049	0,184	0,37087	1
SIGNAL_SEQUENCE_BINDING	-0,049	0,185	0,74308	1
chr8p11	-0,049	0,185	0,18261	1
GGGACCA,MIR-133A,MIR-133B	-0,049	0,185	0,04908	1
FATTY_ACID_BETA_OXIDATION	0,049	0,185	0,73418	1
PID_NFKAPPABCANONICALPATHWAY	-0,049	0,185	0,31496	1
MIKKELSEN_MEF_LCP_WITH_H3K4ME3	-0,049	0,185	0,0248	1
KEGG_NITROGEN_METABOLISM	-0,049	0,185	0,01707	1
GNF2_ATM	-0,049	0,185	0,02907	1
SENGUPTA_NASOPHARYNGEAL_CARCINOMA_	-0,049	0,186	0,00176	1
ZHAN_MULTIPLE_MYELOMA_LB_DN	-0,049	0,186	0,25154	1
GSE22886_NAIVE_BCELL_VS_BM_PLASMA_(	-0,049	0,186	0,27417	1
TACAATC,MIR-508	-0,049	0,186	0,01586	1
GSE360_L_DONOVANI_VS_B_MALAYI_HIGH_	-0,048	0,186	0,05134	1
GSE17721_CTRL_VS_POLYIC_0.5H_BMDM_D	-0,048	0,186	0,0046	1
MODULE_514	-0,048	0,187	0,0021	1
WANG_TARGETS_OF_MLL_CBP_FUSION_UP	-0,048	0,187	0,22284	1
VALK_AML_CLUSTER_4	-0,048	0,187	0,01344	1
PROTEIN_IMPORT_INTO_NUCLEUS_TRANSLOC	-0,048	0,187	0,06887	1
BIOCARTA_INSULIN_PATHWAY	-0,048	0,188	0,01396	1
GSE22886_IGA_VS_IGM_MEMORY_BCELL_DN	-0,048	0,188	0,01866	1
GSE17974_1.5H_VS_72H_IL4_AND_ANTI_IL	-0,048	0,188	0,03869	1
GSE18791_CTRL_VS_NEWCASTLE_VIRUS_DC	-0,048	0,188	0,42181	1

OXIDOREDUCTASE_ACTIVITY_GO_0016616	-0,048	0,188	0,10326	1
CLIMENT_BREAST_CANCER_COPY_NUMBER_D	-0,048	0,188	0,02964	1
KIM_RESPONSE_TO_TSA_AND_DECITABINE_D	-0,048	0,188	0,46368	1
ORGANIC_ANION_TRANSMEMBRANE_TRANSP(	-0,048	0,189	0,27027	1
REACTOME_PHASE1_FUNCTIONALIZATION_OF	-0,048	0,189	0,00289	1
MODULE_45	-0,048	0,189	0,156	1
SCHAEFFER_PROSTATE_DEVELOPMENT_AND_	-0,048	0,189	0,00397	1
BIOCARTA_THELPER_PATHWAY	-0,048	0,189	0,14612	1
MATZUK_IMPLANTATION_AND_UTERINE	-0,048	0,19	0,13764	1
UEDA_CENTRAL_CLOCK	-0,048	0,19	0,07607	1
WORSCHECH_TUMOR_EVASION_AND_TOLERC	-0,048	0,19	0,50389	1
AAGWWRNYGGC_UNKNOWN	-0,048	0,19	0,06319	1
DACOSTA_UV_RESPONSE_VIA_ERCC3_XPCS_	-0,048	0,19	0,40953	1
TURASHVILI_BREAST_NORMAL_DUCTAL_VS_	-0,048	0,19	0,16233	1
FOSTER_TOLERANT_MACROPHAGE_UP	-0,048	0,19	0,35561	1
PENG_GLUCOSE_DEPRIVATION_UP	-0,048	0,191	0,17208	1
TURASHVILI_BREAST_DUCTAL_CARCINOMA_\	-0,048	0,191	0,04323	1
GSE17721_LPS_VS_GARDIQUIMOD_24H_BMD	-0,048	0,191	0,00746	1
GSE17721_PAM3CSK4_VS_CPG_8H_BMDM_[	-0,048	0,191	0,0864	1
MEISSNER_NPC_ICP_WITH_H3_UNMETHYLATE	-0,048	0,191	0,00388	1
chr14q	-0,048	0,191	0,00449	1
AGARWAL_AKT_PATHWAY_TARGETS	-0,048	0,191	0,79792	1
INSOLUBLE_FRACTION	-0,048	0,191	0,09225	1
MYLLYKANGAS_AMPLIFICATION_HOT_SPOT_1	0,048	0,191	0,73687	1
KEGG_INTESTINAL_IMMUNE_NETWORK_FOR_I	-0,048	0,192	0,18073	1
EXTRINSIC_TO_PLASMA_MEMBRANE	0,048	0,192	0,45861	1
GTATGAT,MIR-154,MIR-487	-0,048	0,192	0,01026	1
HAHTOLA_CTCL_CUTANEOUS	-0,048	0,192	0,11171	1
DUNNE_TARGETS_OF_AML1_MTG8_FUSION_(	-0,048	0,192	0,11861	1
HOFFMANN_IMMATURE_TO_MATURE_B_LYMP	-0,048	0,192	0,07015	1
HUMMERICH_MALIGNANT_SKIN_TUMOR_UP	-0,048	0,192	0,64327	1
BAFNA_MUC4_TARGETS_UP	-0,048	0,193	0,72737	1
MODULE_412	-0,048	0,193	0,11071	1
HOEBEKE_LYMPHOID_STEM_CELL_UP	-0,048	0,193	0,02344	1
MYLLYKANGAS_AMPLIFICATION_HOT_SPOT_1	0,048	0,193	0,77926	1
GSE24142_DN2_VS_DN3_THYMOCYTE_FETA	-0,048	0,193	0,00985	1
HOLLEMAN_DAUNORUBICIN_ALL_DN	-0,048	0,194	0,02989	1
KYNG_ENVIRONMENTAL_STRESS_RESPONSE_	-0,048	0,194	0,00022	1
ST_IL_13_PATHWAY	-0,048	0,194	0,15292	1
ST_INTERLEUKIN_13_PATHWAY	-0,048	0,194	0,15292	1
GSE2706_2H_VS_8H_LPS_STIM_DC_DN	-0,048	0,194	0,27169	1
GARGALOVIC_RESPONSE_TO_OXIDIZED_PHOS	-0,048	0,195	0,62982	1

MEDINA_SMARCA4_TARGETS	-0,048	0,195	0,04143	1
GSE13484_UNSTIM_VS_YF17D_VACCINE_STI	-0,048	0,195	0,14806	1
GSE39820_CTRL_VS_IL1B_IL6_IL23A_CD4_	-0,048	0,195	0,02499	1
MODULE_22	0,047	0,195	0,23519	1
BANDRES_RESPONSE_TO_CARMUSTIN_WITHC	0,047	0,195	0,24947	1
BIOCARTA_NO1_PATHWAY	-0,047	0,196	0,20666	1
FU_INTERACT_WITH_ALKBH8	-0,047	0,196	0,41943	1
ACTIVATION_OF_IMMUNE_RESPONSE	-0,047	0,196	0,16771	1
GCM_TPR	-0,047	0,196	0,74016	1
chr3q29	-0,047	0,197	0,60996	1
GSE29617_DAY3_VS_DAY7_TIV_FLU_VACCI	-0,047	0,197	0,15296	1
BANDRES_RESPONSE_TO_CARMUSTIN_MGMT_	-0,047	0,197	0,89546	1
GSE17721_0.5H_VS_24H_POLYIC_BMDM_DN	-0,047	0,197	0,18923	1
INTERLEUKIN_8_BIOSYNTHETIC_PROCESS	0,047	0,197	0,36393	1
KIM_MYC_AMPLIFICATION_TARGETS_DN	-0,047	0,197	0,02835	1
GCM_GSTA4	-0,047	0,197	0,00819	1
RESPONSE_TO_DRUG	0,047	0,197	0,20654	1
chr15q15	-0,047	0,198	0,14962	1
REACTOME_RIP_MEDIATED_NFKB_ACTIVATIO	-0,047	0,198	0,13793	1
HEMATOPOIETIN_INTERFERON_CLASSD200_D	-0,047	0,198	0,14011	1
KEGG_AMINO_SUGAR_AND_NUCLEOTIDE_SUG	0,047	0,198	0,02297	1
WONG_MITOCHONDRIA_GENE_MODULE	0,047	0,198	0,16954	1
MODULE_24	-0,047	0,199	0,0258	1
MULTI_ORGANISM_PROCESS	-0,047	0,199	0,22703	1
REACTOME_TRAF6_MEDIATED_NFKB_ACTIVA	-0,047	0,199	0,21709	1
GSE25087_FETAL_VS_ADULT_TREG_DN	-0,047	0,199	0,00328	1
CREIGHTON_AKT1_SIGNALING_VIA_MTOR_UP	0,047	0,199	0,3226	1
OXIDOREDUCTASE_ACTIVITY_GO_0016705	-0,047	0,2	0,434	1
chr6p21	-0,047	0,2	0,24849	1
BOQUEST_STEM_CELL_CULTURED_VS_FRESH	-0,047	0,2	0,0004	1
chr21p11	0,047	0,2	0,10297	1
GCM_PTK2	-0,047	0,2	0,21792	1
NLS_BEARING_SUBSTRATE_IMPORT_INTO_NU	-0,047	0,2	0,65096	1
SMALL_GTPASE_REGULATOR_ACTIVITY	-0,047	0,2	0,06723	1
FLECHNER_BIOPSY_KIDNEY_TRANSPLANT_OK	-0,047	0,2	0,51743	1
FRASOR_RESPONSE_TO_ESTRADIOL_DN	0,047	0,2	0,93806	1
GALLUZZI_PREVENT_MITOCHONDIAL_PERMEA	0,047	0,2	0,77346	1
BIOCARTA_BAD_PATHWAY	-0,047	0,2	0,00069	1
ALONSO_METASTASIS_EMT_DN	-0,047	0,2	0,40875	1
ROZANOV_MMP14_TARGETS_DN	-0,047	0,2	0,36592	1
MODULE_148	-0,047	0,201	0,0425	1
REACTOME_CROSS_PRESENTATION_OF_SOLU	-0,047	0,201	0,67451	1

MORF_SP3	-0,047	0,201	0,83254	1
GSE14308_TH2_VS_TH1_UP	-0,047	0,201	0,33961	1
SESTO_RESPONSE_TO_UV_C2	-0,047	0,201	0,68494	1
REACTOME_CYTOKINE_SIGNALING_IN_IMMUNE	-0,047	0,202	0,31651	1
REACTOME_SULFUR_AMINO_ACID_METABOLIS	-0,047	0,202	0,07376	1
MCCOLLUM_GELDANAMYCIN_RESISTANCE_DN	-0,047	0,202	0,22682	1
REACTOME_HYALURONAN_METABOLISM	-0,047	0,202	0,1324	1
GSE17721_0.5H_VS_8H_LPS_BMDM_DN	-0,047	0,202	0,04706	1
DEAMINASE_ACTIVITY	-0,047	0,203	0,09609	1
BOQUEST_STEM_CELL_DN	-0,047	0,203	0,01098	1
PROTEIN_MODIFICATION_BY_SMALL_PROTEIN	-0,047	0,203	0,34396	1
REACTOME_VIF_MEDIATED_DEGRADATION_OI	-0,047	0,204	0,73937	1
VANDESLUIS_NORMAL_EMBRYOS_DN	-0,047	0,204	0,01991	1
REACTOME_REGULATION_OF_WATER_BALAN	-0,047	0,204	0,00427	1
GNF2_CCNA1	-0,047	0,204	0,17531	1
GSE22886_CD8_VS_CD4_NAIVE_TCELL_UP	-0,047	0,204	0,15962	1
ROSS_AML_WITH_CBFB_MYH11_FUSION	-0,047	0,204	0,20139	1
ISOMERASE_ACTIVITY	-0,047	0,204	0,91098	1
CDC42_PROTEIN_SIGNAL_TRANSDUCTION	-0,047	0,205	0,05018	1
REACTOME_INTRINSIC_PATHWAY	-0,046	0,205	0,02465	1
AMINOPEPTIDASE_ACTIVITY	-0,046	0,205	0,04208	1
GCM_DEAF1	0,046	0,205	0,46753	1
ZAIDI_OSTEOBLAST_TRANSCRIPTION_FACTOF	-0,046	0,205	0,03334	1
BORCZUK_MALIGNANT_MESOTHELIOMA_DN	-0,046	0,206	0,00377	1
BIOCARTA_TPO_PATHWAY	-0,046	0,206	0,03798	1
GTAAACC,MIR-299-5P	-0,046	0,206	0,04461	1
YAO_TEMPORAL_RESPONSE_TO_PROGESTERC	-0,046	0,206	0,00713	1
GCM_NUMA1	-0,046	0,207	0,03154	1
REACTOME_FGFR1_LIGAND_BINDING_AND_AC	-0,046	0,207	0,14316	1
chr12q12	-0,046	0,207	0,21269	1
GSE17721_CTRL_VS_LPS_0.5H_BMDM_DN	-0,046	0,208	0,00639	1
MORF_BECN1	-0,046	0,208	0,1479	1
GSE13493_DP_VS_CD4INTCD8POS_THYMOCY	-0,046	0,208	0,00886	1
GSE9006_HEALTHY_VS_TYPE_1_DIABETES_I	-0,046	0,208	0,46934	1
GSE24634_IL4_VS_CTRL_TREATED_NAIVE_C	-0,046	0,208	0,17825	1
GSE339_EX_VIVO_VS_IN_CULTURE_CD8POS_	-0,046	0,208	0,031	1
GSE3982_MAST_CELL_VS_EFF_MEMORY_CD4	-0,046	0,209	0,60016	1
POSITIVE_REGULATION_OF_ANGIOGENESIS	-0,046	0,209	0,12431	1
DUTERTRE_ESTRADIOL_RESPONSE_24HR_DN	-0,046	0,209	0,10065	1
ACGCACA,MIR-210	0,046	0,209	0,62558	1
REACTOME_ER_PHAGOSOME_PATHWAY	-0,046	0,209	0,71976	1
SMIRNOV_CIRCULATING_ENDOTHELIOCYTES_I	-0,046	0,209	0,33224	1

KEGG_AMYOTROPHIC_LATERAL_SCLEROSIS_A	-0,046	0,209	0,02854	1
KANG_CISPLATIN_RESISTANCE_DN	0,046	0,209	0,41026	1
NEURON_APOPTOSIS	-0,046	0,209	0,02186	1
SMIRNOV_RESPONSE_TO_IR_6HR_UP	-0,046	0,21	0,0092	1
KINASE_BINDING	-0,046	0,21	0,11992	1
REACTOME_SYNTHESIS_OF_PIPS_AT_THE_EA	0,046	0,21	0,28751	1
MODULE_174	-0,046	0,21	0,33591	1
LANDIS_ERBB2_BREAST_TUMORS_324_DN	-0,046	0,21	0,01073	1
YANG_MUC2_TARGETS_COLON_3MO_DN	0,046	0,21	0,39417	1
ALTEMEIER_RESPONSE_TO_LPS_WITH_MECHA	-0,046	0,211	0,34541	1
GSE17974_0H_VS_48H_IN_VITRO_ACT_CD4	-0,046	0,211	0,00771	1
YAO_TEMPORAL_RESPONSE_TO_PROGESTERC	-0,046	0,211	0,00117	1
GSE7852_TREG_VS_TCONV_UP	-0,046	0,211	0,14332	1
KEGG_STEROID_BIOSYNTHESIS	-0,046	0,211	0,8848	1
REACTOME_SYNTHESIS_SECRETION_AND_INA	-0,046	0,212	0,39711	1
V$RFX1_01	-0,046	0,212	0,00037	1
KEGG_PROTEASOME	-0,046	0,212	0,63415	1
OZEN_MIR125B1_TARGETS	-0,046	0,212	0,67421	1
KEGG_GLYCEROLIPID_METABOLISM	-0,046	0,213	0,16999	1
HOOI_ST7_TARGETS_DN	-0,046	0,213	0,06009	1
BOYAULT_LIVER_CANCER_SUBCLASS_G23_D	-0,046	0,213	0,00021	1
ALCALAY_AML_BY_NPM1_LOCALIZATION_UP	-0,046	0,214	0,03044	1
REACTOME_ACTIVATED_POINT_MUTANTS_OF	-0,046	0,214	0,05312	1
ESTABLISHMENT_OF_PROTEIN_LOCALIZATION	-0,046	0,214	0,16236	1
GSE15930_STIM_VS_STIM_AND_IFNAB_72H_	-0,046	0,214	0,56735	1
GSE12366_GC_VS_MEMORY_BCELL_DN	-0,046	0,214	0,02064	1
MODULE_272	-0,046	0,214	0,33079	1
GSE3982_NKCELL_VS_TH1_UP	-0,046	0,214	0,00908	1
GLUTATHIONE_TRANSFERASE_ACTIVITY	0,046	0,215	0,60849	1
KORKOLA_CHORIOCARCINOMA_UP	-0,046	0,215	0,72153	1
GSE18791_UNSTIM_VS_NEWCATSLE_VIRUS_	-0,046	0,215	0,12403	1
GSE39820_TGFBETA1_VS_TGFBETA3_IN_IL6	-0,046	0,215	0,21187	1
GSE14308_TH2_VS_NAIVE_CD4_TCELL_DN	-0,045	0,215	0,01291	1
REACTOME_CREB_PHOSPHORYLATION_THROU	-0,045	0,215	0,07013	1
OHM_METHYLATED_IN_ADULT_CANCERS	-0,045	0,215	0,01596	1
REACTOME_PROSTANOID_LIGAND_RECEPTORS	-0,045	0,215	0,01304	1
KAAB_FAILED_HEART_VENTRICLE_DN	-0,045	0,215	0,39777	1
WEBER_METHYLATED_ICP_IN_FIBROBLAST	-0,045	0,216	0,49133	1
BIOCARTA_ACH_PATHWAY	-0,045	0,216	0,56059	1
CARBOXYLIC_ACID_TRANSMEMBRANE_TRANS	-0,045	0,216	0,16327	1
GSE3982_MAC_VS_NKCELL_UP	-0,045	0,216	0,41816	1
GSE9037_CTRL_VS_LPS_1H_STIM_IRAK4_KC	-0,045	0,216	0,20626	1

REACTOME_COSTIMULATION_BY_THE_CD28_	-0,045	0,216	0,22926	1
SHI_SPARC_TARGETS_UP	-0,045	0,216	0,01667	1
GSE14308_INDUCED_VS_NATURAL_TREG_DN	-0,045	0,216	0,05839	1
KEGG_LIMONENE_AND_PINENE_DEGRADATION	0,045	0,217	0,17614	1
FAELT_B_CLL_WITH_VH_REARRANGEMENTS_	-0,045	0,217	0,07473	1
chr7p	-0,045	0,217	0,30726	1
CYTOPLASM_ORGANIZATION_AND_BIOGENESI	-0,045	0,218	0,07517	1
MODULE_305	-0,045	0,218	0,3426	1
GCTGAGT,MIR-512-5P	-0,045	0,219	0,02874	1
SA_CASPASE_CASCADE	-0,045	0,219	0,3556	1
TURASHVILI_BREAST_CARCINOMA_DUCTAL_\	-0,045	0,219	0,12762	1
GSE22886_CTRL_VS_LPS_24H_DC_DN	-0,045	0,219	0,40356	1
MEISSNER_NPC_ICP_WITH_H3K4ME3	-0,045	0,219	0,03508	1
GSE15930_STIM_VS_STIM_AND_IFNAB_48H_	-0,045	0,22	0,2753	1
LEE_NAIVE_T_LYMPHOCYTE	-0,045	0,22	0,03358	1
chr10q22	-0,045	0,22	0,06128	1
CAMPS_COLON_CANCER_COPY_NUMBER_DN	-0,045	0,22	0,07697	1
MICROTUBULE_POLYMERIZATION_OR_DEPOLY	-0,045	0,22	0,00406	1
CHEOK_RESPONSE_TO_MERCAPTOPURINE_UP	-0,045	0,22	0,19232	1
LI_DCP2_BOUND_MRNA	-0,045	0,22	0,49082	1
REACTOME_DOUBLE_STRAND_BREAK_REPAIR	-0,045	0,221	0,70214	1
GSE3982_MAST_CELL_VS_DC_DN	-0,045	0,221	0,14131	1
BIOCARTA_MTA3_PATHWAY	-0,045	0,221	0,0068	1
REACTOME_NFKB_AND_MAP_KINASES_ACTIV	-0,045	0,221	0,25635	1
MENSE_HYPOXIA_UP	-0,045	0,222	0,42961	1
MODULE_277	-0,045	0,222	0,6851	1
CAIRO_HEPATOBLASTOMA_CLASSES_DN	-0,045	0,222	0,00165	1
UBIQUITIN_CYCLE	-0,045	0,222	0,44737	1
CCAATNNSNNNGCG_UNKNOWN	-0,045	0,222	0,85363	1
REACTOME_ACTIVATION_OF_NF_KAPPAB_IN_	-0,045	0,223	0,79034	1
REACTOME_INTEGRIN_ALPHAIIB_BETA3_SIGN.	-0,045	0,223	0,10467	1
NIELSEN_GIST_VS_SYNOVIAL_SARCOMA_UP	-0,045	0,223	0,31286	1
V$HNF1_C	-0,045	0,223	0,00645	1
NEUTRAL_AMINO_ACID_TRANSPORT	0,045	0,224	0,30401	1
MODULE_291	-0,045	0,224	0,53947	1
REACTOME_SEMA3A_PLEXIN_REPULSION_SIGI	-0,045	0,224	0,04159	1
WEBER_METHYLATED_HCP_IN_FIBROBLAST_I	-0,045	0,224	0,11537	1
SETLUR_PROSTATE_CANCER_TMPRSS2_ERG_	-0,045	0,224	0,00815	1
FINETTI_BREAST_CANCERS_KINOME_GRAY	0,045	0,224	0,47538	1
REACTOME_TCR_SIGNALING	-0,045	0,225	0,15626	1
AMIT_EGF_RESPONSE_20_HELA	-0,045	0,225	0,00227	1
MODULE_249	0,044	0,226	0,69114	1

VANLOO_SP3_TARGETS_UP	0,044	0,226	0,13082	1
CHASSOT_SKIN_WOUND	-0,044	0,226	0,00228	1
MODULE_234	-0,044	0,226	0,06167	1
PROTEIN_UBIQUITINATION	-0,044	0,226	0,33254	1
TING_SILENCED_BY_DICER	-0,044	0,227	0,00779	1
GSE29618_LAIV_VS_TIV_FLU_VACCINE_DAY	-0,044	0,227	0,24766	1
MCBRYAN_PUBERTAL_BREAST_4_5WK_DN	-0,044	0,227	0,02079	1
MORF_BAG5	-0,044	0,227	0,89993	1
NAKAMURA_CANCER_MICROENVIRONMENT_UI	-0,044	0,228	0,00567	1
BIOCARTA_CCR5_PATHWAY	-0,044	0,228	0,17091	1
BIOCARTA_CELL2CELL_PATHWAY	-0,044	0,228	0,70731	1
SU_PLACENTA	-0,044	0,228	0,41538	1
RESPONSE_TO_CHEMICAL_STIMULUS	-0,044	0,228	0,10682	1
chr2q12	-0,044	0,229	0,71576	1
NIKOLSKY_BREAST_CANCER_12Q13_Q21_AN	-0,044	0,229	0,21145	1
GSE7460_CTRL_VS_TGFB_TREATED_ACT_FC	-0,044	0,229	0,00539	1
GSE3982_EFF_MEMORY_CD4_TCELL_VS_TH2	-0,044	0,229	0,02657	1
PEPTIDE_BINDING	-0,044	0,229	0,04744	1
SCIBETTA_KDM5B_TARGETS_UP	0,044	0,229	0,2921	1
GNF2_GSTM1	0,044	0,229	0,40882	1
WILLERT_WNT_SIGNALING	-0,044	0,229	0,00523	1
BIOCARTA_BARR_MAPK_PATHWAY	-0,044	0,229	0,07308	1
GSE36476_CTRL_VS_TSST_ACT_72H_MEMO	-0,044	0,23	0,00518	1
LENAOUR_DENDRITIC_CELL_MATURATION_UP	-0,044	0,23	0,2387	1
KESHELAVA_MULTIPLE_DRUG_RESISTANCE	-0,044	0,23	0,04615	1
SHIPP_DLBCL_CURED_VS_FATAL_DN	-0,044	0,23	0,48819	1
PROTEIN_KINASE_BINDING	-0,044	0,23	0,30012	1
KEGG_PORPHYRIN_AND_CHLOROPHYLL_META	0,044	0,231	0,05471	1
BIOCARTA_CYTOKINE_PATHWAY	-0,044	0,231	0,57578	1
GSE3982_BASOPHIL_VS_TH1_UP	-0,044	0,231	0,02534	1
XU_RESPONSE_TO_TRETINOIN_UP	-0,044	0,232	0,37728	1
KEGG_RENIN_ANGIOTENSIN_SYSTEM	-0,044	0,232	0,01294	1
SHEDDEN_LUNG_CANCER_GOOD_SURVIVAL_/	-0,044	0,232	0,00393	1
GNF2_RAP1B	-0,044	0,232	0,44088	1
REACTOME_SIGNALING_BY_FGFR3_MUTANTS	-0,044	0,233	0,0317	1
MORF_AP2M1	-0,044	0,233	0,93999	1
MODULE_77	0,044	0,233	0,2643	1
GSE17721_LPS_VS_CPG_8H_BMDM_DN	-0,044	0,234	0,76515	1
GSE17721_CTRL_VS_CPG_1H_BMDM_UP	-0,044	0,234	0,003	1
APICAL_PLASMA_MEMBRANE	-0,044	0,234	0,74714	1
GSE37416_0H_VS_48H_F_TULARENSIS_LVS	-0,044	0,235	0,53522	1
REACTOME_REGULATION_OF_APOPTOSIS	-0,044	0,235	0,62369	1

LIPID_TRANSPORT	-0,044	0,235	0,037	1
MORF_XPC	-0,044	0,235	0,74552	1
KEGG_GLYCOSPHINGOLIPID_BIOSYNTHESIS_GL	-0,043	0,236	0,27348	1
KONDO_COLON_CANCER_HCP_WITH_H3K27M	-0,043	0,236	0,777	1
REACTOME_PRE_NOTCH_PROCESSING_IN_GOI	-0,043	0,236	0,05926	1
SYNAPTIC_VESICLE	-0,043	0,237	0,12153	1
GSE17721_0.5H_VS_24H_GARDIQUIMOD_BM	-0,043	0,237	0,27713	1
chr10p15	-0,043	0,237	0,5985	1
KORKOLA_CHORIOCARCINOMA	-0,043	0,237	0,01947	1
GSE39820_TGFBETA1_VS_TGFBETA3_IN_IL6	-0,043	0,237	0,08673	1
RICKMAN_HEAD_AND_NECK_CANCER_E	-0,043	0,237	0,67026	1
GNF2_SNRK	-0,043	0,238	0,16982	1
BRACHAT_RESPONSE_TO_METHOTREXATE_DI	-0,043	0,238	0,53668	1
REACTOME_BASIGIN_INTERACTIONS	-0,043	0,238	0,65526	1
GSE17721_POLYIC_VS_PAM3CSK4_8H_BMDI	-0,043	0,238	0,06619	1
ACAACTT,MIR-382	-0,043	0,238	0,04044	1
REACTOME_GLUCAGON_SIGNALING_IN_METAI	-0,043	0,239	0,00361	1
PID_TRAIL_PATHWAY	0,043	0,239	0,29447	1
chr3p25	-0,043	0,239	0,21178	1
POMEROY_MEDULLOBLASTOMA_DESMOPLASI	-0,043	0,239	0,063	1
GSE37416_0H_VS_48H_F_TULARENSIS_LVS	-0,043	0,24	0,11435	1
GSE7764_IL15_NK_CELL_24H_VS_SPLENOC	-0,043	0,24	0,04794	1
GERHOLD_RESPONSE_TO_TZD_DN	-0,043	0,24	0,01548	1
LEE_AGING_CEREBELLUM_UP	-0,043	0,24	0,13481	1
KEGG_VALINE_LEUCINE_AND_ISOLEUCINE_BI	0,043	0,24	0,08606	1
GSE10094_LCMV_VS_LISTERIA_IND_EFF_CD	0,043	0,241	0,99912	1
LAU_APOPTOSIS_CDKN2A_DN	-0,043	0,241	0,11042	1
GSE27786_CD8_TCELL_VS_NKTCELL_DN	-0,043	0,241	0,12033	1
ZEILSTRA_CD44_TARGETS_UP	-0,043	0,241	0,12193	1
GUENTHER_GROWTH_SPHERICAL_VS_ADHERE	-0,043	0,241	0,1527	1
KEGG_GLYOXYLATE_AND_DICARBOXYLATE_M	-0,043	0,241	0,77044	1
GSE7400_CTRL_VS_CSF3_IN_VIVO_TREATED	-0,043	0,241	0,3224	1
WANG_NFKB_TARGETS	-0,043	0,241	0,03176	1
VISALA_RESPONSE_TO_HEAT_SHOCK_AND_	-0,043	0,241	0,35841	1
KEGG_ANTIGEN_PROCESSING_AND_PRESENT	-0,043	0,242	0,40199	1
MEISSNER_BRAIN_ICP_WITH_H3K4ME3	-0,043	0,242	0,00113	1
IVANOVSKA_MIR106B_TARGETS	-0,043	0,242	0,10215	1
GSE17721_0.5H_VS_24H_PAM3CSK4_BMDM	-0,043	0,242	0,46197	1
KEGG_TAURINE_AND_HYPOTAURINE_METABO	0,043	0,242	0,95371	1
DNA_DAMAGE_RESPONSESIGNAL_TRANSDUC	-0,043	0,242	0,80083	1
SCHLOSSER_SERUM_RESPONSE_UP	-0,043	0,243	0,01457	1
WU_HBX_TARGETS_1_DN	-0,043	0,243	0,12428	1

SHARMA_PILOCYTIC_ASTROCYTOMA_LOCATI(	-0,043	0,243	0,30082	1
KRAS.AMP.LUNG_UP.V1_UP	-0,043	0,243	0,05766	1
chr2q21	-0,043	0,244	0,0004	1
MACROMOLECULE_CATABOLIC_PROCESS	-0,043	0,244	0,69469	1
KYNG_DNA_DAMAGE_BY_4NQO	-0,043	0,244	0,07551	1
KASLER_HDAC7_TARGETS_2_UP	-0,043	0,244	0,02043	1
NELSON_RESPONSE_TO_ANDROGEN_DN	-0,043	0,244	0,03831	1
LUI_THYROID_CANCER_CLUSTER_5	0,043	0,244	0,28142	1
MORF_FLT1	-0,043	0,244	0,01595	1
MARKS_HDAC_TARGETS_DN	-0,043	0,245	0,63797	1
WOTTON_RUNX_TARGETS_UP	-0,043	0,245	0,20863	1
VECCHI_GASTRIC_CANCER_ADVANCED_VS_E	0,043	0,245	0,47904	1
SUBTIL_PROGESTIN_TARGETS	-0,043	0,245	0,03305	1
HUMMERICH_MALIGNANT_SKIN_TUMOR_DN	-0,043	0,246	0,03886	1
PARENT_MTOR_SIGNALING_UP	-0,043	0,246	0,00149	1
GROSS_HIF1A_TARGETS_UP	-0,043	0,246	0,21822	1
GAUSSMANN_MLL_AF4_FUSION_TARGETS_B_	-0,043	0,246	0,02729	1
REGULATION_OF_RAS_PROTEIN_SIGNAL_TRA	-0,043	0,246	0,14216	1
MEINHOLD_OVARIAN_CANCER_LOW_GRADE_	-0,042	0,247	0,11225	1
DOANE_RESPONSE_TO_ANDROGEN_UP	-0,042	0,247	0,00496	1
MICROSOME	0,042	0,247	0,49073	1
GSE22886_IGM_MEMORY_BCELL_VS_BLOOD_	-0,042	0,247	0,84627	1
COFACTOR_BINDING	-0,042	0,247	0,00217	1
MODULE_310	0,042	0,247	0,30852	1
GSE24142_EARLY_THYMIC_PROGENITOR_VS_	-0,042	0,247	0,15692	1
GNF2_CDKN1C	-0,042	0,248	0,03139	1
GSE18791_CTRL_VS_NEWCASTLE_VIRUS_DC	-0,042	0,248	0,55562	1
GSE37416_0H_VS_24H_F_TULARENSIS_LVS	-0,042	0,248	0,21346	1
GSE9988_LOW_LPS_VS_CTRL_TREATED_MO	-0,042	0,248	0,0909	1
ZHU_CMV_8_HR_UP	-0,042	0,248	0,60553	1
ACTIVATION_OF_PROTEIN_KINASE_ACTIVITY	-0,042	0,249	0,07704	1
MORF_PML	-0,042	0,249	0,00749	1
PHOSPHOLIPASE_A2_ACTIVITY	-0,042	0,25	0,02854	1
LAIHO_COLORECTAL_CANCER_SERRATED_DN	-0,042	0,25	0,01536	1
GSE17974_0H_VS_2H_IN_VITRO_ACT_CD4_	-0,042	0,25	0,00691	1
ROYLANCE_BREAST_CANCER_16Q_COPY_NU	-0,042	0,25	0,97182	1
MORF_ETV3	-0,042	0,25	0,01799	1
chr19p	0,042	0,251	0,33084	1
REACTOME_REGULATION_OF_IFNA_SIGNALIN(	-0,042	0,251	0,55693	1
WIKMAN_ASBESTOS_LUNG_CANCER_UP	-0,042	0,251	0,04689	1
REACTOME_METABOLISM_OF_LIPIDS_AND_LII	-0,042	0,251	0,03424	1
REACTOME_RESOLUTION_OF_AP_SITES_VIA_	-0,042	0,251	0,09016	1

MODULE_346	0,042	0,251	0,21313	1
STAMBOLSKY_TARGETS_OF_MUTATED_TP53	-0,042	0,252	0,09633	1
HYDROLASE_ACTIVITY_ACTING_ON_ACID_AN	0,042	0,252	0,48371	1
NAKAJIMA_MAST_CELL	-0,042	0,253	0,00472	1
LUND_SILENCED_BY_METHYLATION	-0,042	0,253	0,56454	1
ADAPTIVE_IMMUNE_RESPONSE	-0,042	0,253	0,29774	1
REGULATION_OF_RHO_PROTEIN_SIGNAL_TRA	-0,042	0,254	0,09562	1
HOFFMANN_LARGE_TO_SMALL_PRE_BII_LYMI	-0,042	0,254	0,03348	1
REACTOME_ACTIVATED_TLR4_SIGNALLING	-0,042	0,254	0,25577	1
GSE360_CTRL_VS_L_DONOVANI_MAC_UP	-0,042	0,254	0,15039	1
REACTOME_ADP_SIGNALLING_THROUGH_P2R	-0,042	0,255	0,16107	1
STREICHER_LSM1_TARGETS_UP	-0,042	0,255	0,06132	1
RICKMAN_TUMOR_DIFFERENTIATED_WELL_VS	-0,042	0,256	0,01492	1
MORF_JAK3	-0,042	0,256	0,00254	1
KEGG_ARGININE_AND_PROLINE_METABOLISM	-0,042	0,256	0,09689	1
GSE13484_12H_UNSTIM_VS_YF17D_VACCIN	-0,042	0,256	0,12077	1
ORGANIC_ACID_TRANSMEMBRANE_TRANSPOF	-0,042	0,256	0,19833	1
REACTOME_SIGNALING_BY_NOTCH2	0,042	0,257	0,931	1
GSE27786_BCELL_VS_ERYTHROBLAST_UP	-0,042	0,257	0,22416	1
SIRNA_EIF4GI_UP	-0,042	0,257	0,37798	1
NEGATIVE_REGULATION_OF_CELLULAR_BIOS	0,042	0,257	0,91191	1
REACTOME_BIOSYNTHESIS_OF_THE_N_GLYC	0,042	0,257	0,21632	1
GSE3982_MAC_VS_TH2_UP	0,042	0,258	0,65946	1
MODULE_152	0,041	0,258	0,16092	1
REACTOME_SIGNALING_BY_NOTCH3	0,041	0,259	0,94079	1
CHEOK_RESPONSE_TO_MERCAPTOPURINE_AN	-0,041	0,259	0,09576	1
GCM_LTK	-0,041	0,259	0,08139	1
REACTOME_SIGNALING_BY_NOTCH4	0,041	0,259	0,75008	1
GSE17721_POLYIC_VS_GARDIQUIMOD_4H_BI	-0,041	0,26	0,04881	1
RAMALHO_STEMNESS_DN	-0,041	0,26	0,06753	1
ADAPTIVE_IMMUNE_RESPONSE_GO_0002460	-0,041	0,26	0,30271	1
REACTOME_GLUCAGON_TYPE_LIGAND_RECEP	-0,041	0,26	0,01866	1
CARBOHYDRATE_BINDING	-0,041	0,261	0,06548	1
PROTEIN_HOMOOLIGOMERIZATION	0,041	0,261	0,93647	1
chr2q31	0,041	0,261	0,66395	1
chr5q34	-0,041	0,262	0,07574	1
BUCKANOVICH_T_LYMPHOCYTE_HOMING_ON	-0,041	0,262	0,04359	1
ORGANIC_ACID_METABOLIC_PROCESS	-0,041	0,263	0,01348	1
GSE24634_IL4_VS_CTRL_TREATED_NAIVE_C	-0,041	0,263	0,4983	1
BIOCARTA_DC_PATHWAY	-0,041	0,263	0,3821	1
KEGG_LINOLEIC_ACID_METABOLISM	0,041	0,263	0,65349	1
GSE37416_0H_VS_12H_F_TULARENSIS_LVS	-0,041	0,263	0,07218	1

GSE22886_DAY0_VS_DAY7_MONOCYTE_IN_	-0,041	0,263	0,04645	1
CTGTTAC,MIR-194	-0,041	0,263	0,25407	1
SAGIV_CD24_TARGETS_DN	-0,041	0,263	0,21059	1
GSE22886_UNSTIM_VS_STIM_MEMORY_TCEL	-0,041	0,264	0,02594	1
NEMETH_INFLAMMATORY_RESPONSE_LPS_UP	-0,041	0,264	0,54726	1
STEIN_ESRRA_TARGETS_UP	-0,041	0,264	0,62105	1
OUELLET_OVARIAN_CANCER_INVASIVE_VS_L	0,041	0,264	0,33351	1
REACTOME_REGULATION_OF_ORNITHINE_DEC	-0,041	0,264	0,56426	1
LU_TUMOR_VASCULATURE_DN	-0,041	0,265	0,00029	1
RESPONSE_TO_VIRUS	-0,041	0,265	0,35503	1
GESERICK_TERT_TARGETS_DN	-0,041	0,265	0,00093	1
GUILLAUMOND_KLF10_TARGETS_DN	-0,041	0,265	0,39141	1
GSE17721_0.5H_VS_12H_POLYIC_BMDM_DN	-0,041	0,266	0,21787	1
BARRIER_CANCER_RELAPSE_TUMOR_SAMPLE_	-0,041	0,266	0,39114	1
ROSS_AML_WITH_MLL_FUSIONS	0,041	0,266	0,81115	1
YOKOE_CANCER_TESTIS_ANTIGENS	-0,041	0,266	0,0908	1
TONKS_TARGETS_OF_RUNX1_RUNX1T1_FUSI	-0,041	0,266	0,23318	1
GSE1432_1H_VS_6H_IFNG_MICROGLIA_DN	-0,041	0,267	0,60748	1
chr7p11	-0,041	0,267	0,55875	1
AGUIRRE_PANCREATIC_CANCER_COPY_NUMBI	-0,041	0,268	0,07372	1
SPECIFIC_TRANSCRIPTIONAL_REPRESSOR_ACT	-0,041	0,268	0,0072	1
BIOCARTA_COMP_PATHWAY	0,041	0,268	0,69074	1
VERNELL_RETINOBLASTOMA_PATHWAY_DN	-0,041	0,268	0,06108	1
NICK_RESPONSE_TO_PROC_TREATMENT_UP	-0,041	0,268	0,28528	1
XU_RESPONSE_TO_TRETINOIN_AND_NSC682!	-0,041	0,269	0,83524	1
HECKER_IFNB1_TARGETS	-0,041	0,269	0,47679	1
chr16p11	0,041	0,269	0,99546	1
PLASARI_TGFB1_TARGETS_1HR_DN	-0,041	0,269	0,00773	1
GSE3982_BASOPHIL_VS_EFF_MEMORY_CD4_	-0,041	0,269	0,07309	1
GSE360_CTRL_VS_T_GONDII_DC_UP	-0,041	0,269	0,04996	1
GSE24634_IL4_VS_CTRL_TREATED_NAIVE_C	-0,041	0,269	0,41247	1
CELLULAR_LIPID_METABOLIC_PROCESS	-0,041	0,269	0,02234	1
MODULE_79	-0,041	0,27	0,19429	1
PRIMARY_ACTIVE_TRANSMEMBRANE_TRANSP	0,041	0,27	0,45705	1
REACTOME_RNA_POL_I_TRANSCRIPTION_TER	-0,04	0,27	0,18523	1
PHOTOTRANSDUCTION	-0,04	0,27	0,001	1
GSE3982_NEUTROPHIL_VS_EFF_MEMORY_CD	-0,04	0,27	0,41366	1
GLUCOSE_CATABOLIC_PROCESS	0,04	0,27	0,43256	1
REACTOME_GLUCOSE_METABOLISM	0,04	0,27	0,06622	1
REACTOME_FORMATION_OF_FIBRIN_CLOT_CL	-0,04	0,271	0,01592	1
REACTOME_HOMOLOGOUS_RECOMBINATION_I	-0,04	0,271	0,68236	1
GSE360_L_MAJOR_VS_M_TUBERCULOSIS_DC	-0,04	0,271	0,07949	1

MODULE_335	0,04	0,271	0,07388	1
chr2q22	-0,04	0,272	0,41304	1
EXTRACELLULAR_LIGAND_GATED_ION_CHANI	-0,04	0,272	0,14374	1
LANDIS_ERBB2_BREAST_TUMORS_65_UP	-0,04	0,273	0,59124	1
HEME_METABOLIC_PROCESS	0,04	0,273	0,50579	1
KOBAYASHI_EGFR_SIGNALING_6HR_UP	-0,04	0,273	0,09192	1
GSE20151_CTRL_VS_FUSOBACT_NUCLEATUI	-0,04	0,273	0,03525	1
GSE3982_NEUTROPHIL_VS_CENT_MEMORY_C	-0,04	0,273	0,42821	1
TCGA_GLIOBLASTOMA_COPY_NUMBER_DN	-0,04	0,273	0,18072	1
chr3p24	-0,04	0,274	0,03363	1
GSE7460_FOXP3_MUT_VS_WT_ACT_TCONV_	-0,04	0,274	0,19048	1
GSE3982_MAC_VS_TH1_UP	0,04	0,274	0,46313	1
CHESLER_BRAIN_D6MIT150_QTL_TRANS	-0,04	0,274	0,55616	1
MODULE_50	-0,04	0,275	0,65938	1
WAKABAYASHI_ADIPOGENESIS_PPARG_RXRA_	0,04	0,275	0,7332	1
VANDESLUIS_COMMD1_TARGETS_GROUP_2_I	-0,04	0,275	0,41324	1
KEGG_SPHINGOLIPID_METABOLISM	0,04	0,275	0,60432	1
BRACHAT_RESPONSE_TO_METHOTREXATE_UI	0,04	0,275	0,37059	1
GSE3982_NEUTROPHIL_VS_BASOPHIL_UP	-0,04	0,275	0,02277	1
chr7p13	-0,04	0,275	0,02836	1
ATPASE_ACTIVITY_COUPLED_TO_MOVEMENT	0,04	0,275	0,46383	1
REACTOME_GAMMA_CARBOXYLATION_TRANS	0,04	0,276	0,45734	1
CAR_IGFBP1	-0,04	0,276	0,01081	1
PELLICCIOTTA_HDAC_IN_ANTIGEN_PRESENTA	0,04	0,276	0,02089	1
BOYAULT_LIVER_CANCER_SUBCLASS_G12_U	0,04	0,276	0,29224	1
SPERM_MOTILITY	-0,04	0,277	0,19189	1
GNF2_PRDX2	-0,04	0,277	0,4846	1
JIANG_AGING_HYPOTHALAMUS_DN	-0,04	0,277	0,09872	1
REACTOME_ABCA_TRANSPORTERS_IN_LIPID_	-0,04	0,278	0,00053	1
TARTE_PLASMA_CELL_VS_B_LYMPHOCYTE_I	-0,04	0,278	0,15063	1
MIKKELSEN_IPS_LCP_WITH_H3K4ME3	-0,04	0,278	0,01827	1
REACTOME_ADENYLATE_CYCLASE_INHIBITOR	-0,04	0,279	0,00099	1
SEKI_INFLAMMATORY_RESPONSE_LPS_DN	-0,04	0,279	0,00173	1
TAATAAT,MIR-126	-0,04	0,279	0,06705	1
REACTOME_VITAMIN_B5_PANTOTHENATE_MI	-0,04	0,279	0,11966	1
BIOCARTA_AKAP95_PATHWAY	-0,04	0,279	0,63249	1
GSE3982_BCELL_VS_TH2_UP	-0,04	0,279	0,08016	1
MORF_PSMC1	-0,04	0,279	0,9892	1
GSE7852_THYMUS_VS_FAT_TREG_DN	-0,04	0,279	0,0766	1
CHANG_POU5F1_TARGETS_UP	-0,04	0,28	0,2003	1
REACTOME_NUCLEOTIDE_BINDING_DOMAIN_L	-0,04	0,28	0,44869	1
DEMAGALHAES_AGING_DN	-0,04	0,28	0,09865	1

GSE13484_UNSTIM_VS_3H_YF17D_VACCINE	-0,04	0,28	0,00193	1
REACTOME_PHASE_II_CONJUGATION	0,04	0,281	0,23996	1
CERVERA_SDHB_TARGETS_1_DN	-0,04	0,281	0,01958	1
MODULE_172	-0,04	0,281	0,24095	1
GSE14769_40MIN_VS_360MIN_LPS_BMDM_U	-0,04	0,282	0,1247	1
LIU_SMARCA4_TARGETS	-0,04	0,282	0,02223	1
PID_HDAC_CLASSIII_PATHWAY	-0,039	0,282	0,1428	1
LU_EZH2_TARGETS_UP	-0,039	0,282	0,25012	1
GSE2706_UNSTIM_VS_8H_LPS_DC_UP	-0,039	0,282	0,00171	1
NEGATIVE_REGULATION_OF_BIOSYNTHETIC_F	0,039	0,282	0,82869	1
REACTOME_BASE_EXCISION_REPAIR	-0,039	0,283	0,08979	1
SCHRAETS_MLL_TARGETS_DN	-0,039	0,283	0,14554	1
REACTOME_INTERACTION_BETWEEN_L1_AND	-0,039	0,283	0,03404	1
GROSS_ELK3_TARGETS_DN	-0,039	0,284	0,74022	1
REACTOME_INHIBITION_OF_REPLICATION_INIT	-0,039	0,284	0,98463	1
ACTIN_FILAMENT_ORGANIZATION	0,039	0,284	0,36199	1
GSE17721_CPG_VS_GARDIQUIMOD_12H_BMD	-0,039	0,284	0,24311	1
BILANGES_RAPAMYCIN_SENSITIVE_GENES	-0,039	0,284	0,12812	1
MYLLYKANGAS_AMPLIFICATION_HOT_SPOT_6	0,039	0,284	0,74166	1
GABRIELY_MIR21_TARGETS	-0,039	0,285	0,35926	1
chr12q15	-0,039	0,285	0,10375	1
ROETH_TERT_TARGETS_UP	-0,039	0,285	0,50562	1
REACTOME_SIGNALING_BY_WNT	-0,039	0,285	0,54906	1
MCLACHLAN_DENTAL_CARIES_UP	-0,039	0,285	0,5137	1
WEST_ADRENOCORTICAL_CARCINOMA_VS_AI	-0,039	0,285	0,9151	1
MOOTHA_PGC	-0,039	0,286	0,77943	1
BAUS_TFF2_TARGETS_UP	-0,039	0,286	0,04314	1
ATP_DEPENDENT_RNA_HELICASE_ACTIVITY	-0,039	0,286	0,97767	1
VALK_AML_CLUSTER_11	-0,039	0,286	0,09546	1
KOINUMA_COLON_CANCER_MSI_UP	-0,039	0,287	0,42686	1
PID_TELOMERASEPATHWAY	-0,039	0,287	0,19336	1
OXIDOREDUCTASE_ACTIVITY_ACTING_ON_TH	-0,039	0,287	0,02504	1
V$FXR_IR1_Q6	-0,039	0,287	0,0008	1
CYTOSOL	-0,039	0,287	0,00768	1
IIZUKA_LIVER_CANCER_PROGRESSION_L1_G1	-0,039	0,288	0,2116	1
REACTOME_BINDING_AND_ENTRY_OF_HIV_VI	-0,039	0,288	0,39254	1
HSIAO_LIVER_SPECIFIC_GENES	0,039	0,288	0,83722	1
MCCOLLUM_GELDANAMYCIN_RESISTANCE_UP	-0,039	0,288	0,01095	1
PID_VEGFR1_PATHWAY	-0,039	0,288	0,35942	1
REACTOME_MITOCHONDRIAL_FATTY_ACID_BI	0,039	0,289	0,63965	1
KANG_AR_TARGETS_DN	-0,039	0,289	0,5524	1
GSE1432_CTRL_VS_IFNG_6H_MICROGLIA_DN	-0,039	0,29	0,54438	1

BIOCARTA_LEPTIN_PATHWAY	-0,039	0,29	0,06472	1
V$PPARG_01	-0,039	0,29	0,43213	1
RESPONSE_TO_OTHER_ORGANISM	-0,039	0,29	0,37713	1
MARSON_FOXP3_TARGETS_UP	-0,039	0,29	0,18059	1
LUCAS_HNF4A_TARGETS_UP	0,039	0,291	0,72811	1
PROTEIN_N_TERMINUS_BINDING	0,039	0,291	0,21734	1
DONATO_CELL_CYCLE_TRETINOIN	0,039	0,291	0,47254	1
CARBONATE_DEHYDRATASE_ACTIVITY	-0,039	0,291	0,0889	1
MONOVALENT_INORGANIC_CATION_TRANSME	0,039	0,292	0,86192	1
METABOTROPIC_GLUTAMATEGABA_B_LIKE_R	-0,039	0,292	0,35531	1
chr8q	-0,039	0,292	0,92412	1
MORF_DDX11	-0,039	0,292	0,01611	1
BOYAULT_LIVER_CANCER_SUBCLASS_G5_DN	-0,039	0,292	0,40412	1
GSE360_L_DONOVANI_VS_M_TUBERCULOSIS	-0,039	0,293	0,647	1
GSE13484_UNSTIM_VS_12H_YF17D_VACCIN	-0,039	0,293	0,40471	1
GAUSSMANN_MLL_AF4_FUSION_TARGETS_D_	0,039	0,293	0,89483	1
HOEGERKORP_CD44_TARGETS_TEMPORAL_DI	-0,039	0,293	0,65465	1
HOLLEMAN_ASPARAGINASE_RESISTANCE_B_/	-0,038	0,294	0,19265	1
JI_METASTASIS_REPRESSED_BY_STK11	-0,038	0,294	0,05566	1
MITOCHONDRIAL_RESPIRATORY_CHAIN	0,038	0,294	0,21286	1
SHIRAISHI_PLZF_TARGETS_UP	-0,038	0,294	0,3465	1
REACTOME_ACTIVATED_TAK1_MEDIATES_P3	-0,038	0,295	0,88295	1
PID_RETINOIC_ACID_PATHWAY	-0,038	0,295	0,09531	1
REACTOME_FORMATION_OF_TRANSCRIPTION_	0,038	0,295	0,43771	1
SCHAEFFER_PROSTATE_DEVELOPMENT_AND_	-0,038	0,296	0,20951	1
HEMOSTASIS	-0,038	0,296	0,00211	1
GSE17721_0.5H_VS_4H_GARDIQUIMOD_BMD	-0,038	0,296	0,15299	1
MODULE_333	0,038	0,297	0,30856	1
GNF2_SPI1	0,038	0,297	0,24573	1
KEGG_GLYCOSAMINOGLYCAN_DEGRADATION	-0,038	0,298	0,16086	1
REACTOME_SPHINGOLIPID_METABOLISM	0,038	0,298	0,5525	1
MODULE_128	-0,038	0,298	0,22459	1
MODULE_492	0,038	0,298	0,99647	1
RODRIGUES_NTN1_AND_DCC_TARGETS	-0,038	0,298	0,00054	1
KARLSSON_TGFB1_TARGETS_DN	-0,038	0,298	0,02202	1
GSE22886_IGG_IGA_MEMORY_BCELL_VS_BL(	-0,038	0,298	0,62232	1
GSE17721_PAM3CSK4_VS_CPG_12H_BMDM_	-0,038	0,298	0,18827	1
KINNEY_DNMT1_METHYLATION_TARGETS	-0,038	0,299	0,01389	1
JOHNSTONE_PARVB_TARGETS_3_UP	-0,038	0,299	0,09815	1
SEITZ_NEOPLASTIC_TRANSFORMATION_BY_8	-0,038	0,299	0,31028	1
REACTOME_TOLL_RECEPTOR_CASCADES	-0,038	0,299	0,39166	1
ICHIBA_GRAFT_VERSUS_HOST_DISEASE_35D_	0,038	0,299	0,28437	1

REACTOME_ABACAVIR_TRANSPORT_AND_ME	-0,038	0,299	0,02065	1
OUYANG_PROSTATE_CANCER_PROGRESSION_	-0,038	0,3	0,14782	1
GSE339_CD4POS_VS_CD8POS_DC_IN_CULTU	-0,038	0,3	0,35172	1
LIPOPROTEIN_BINDING	0,038	0,301	0,77997	1
GUANYL_NUCLEOTIDE_EXCHANGE_FACTOR_A	-0,038	0,301	0,02878	1
GARGALOVIC_RESPONSE_TO_OXIDIZED_PHOS	-0,038	0,301	0,42524	1
BIOCARTA_EPO_PATHWAY	-0,038	0,301	0,05007	1
GSE17580_UNINFECTED_VS_S_MANSONI_INF	-0,038	0,301	0,05476	1
GENTILE_UV_RESPONSE_CLUSTER_D5	-0,038	0,301	0,08672	1
TERAMOTO_OPN_TARGETS_CLUSTER_5	0,038	0,302	0,51535	1
REACTOME_RIG_I_MDA5_MEDIATED_INDUCTI(	-0,038	0,302	0,27837	1
MODULE_5	-0,038	0,302	0,12273	1
ER_GOLGI_INTERMEDIATE_COMPARTMENT	-0,038	0,302	0,68124	1
MODULE_91	-0,038	0,302	0,45643	1
chr5q11	-0,038	0,303	0,0053	1
GSE22886_IGG_IGA_MEMORY_BCELL_VS_BM	-0,038	0,303	0,14137	1
GSE360_CTRL_VS_M_TUBERCULOSIS_DC_DN	-0,038	0,303	0,93783	1
HAHTOLA_MYCOSIS_FUNGOIDES_CD4_DN	-0,038	0,303	0,124	1
LANDIS_BREAST_CANCER_PROGRESSION_DN	-0,038	0,305	0,02261	1
REACTOME_DOWNSTREAM_TCR_SIGNALING	-0,038	0,305	0,25713	1
CHO_NR4A1_TARGETS	-0,038	0,305	0,35861	1
PETRETTO_BLOOD_PRESSURE_UP	-0,038	0,305	0,38504	1
GALE_APL_WITH_FLT3_MUTATED_DN	0,038	0,305	0,24137	1
HORMONE_BINDING	0,038	0,305	0,56408	1
GSE1432_CTRL_VS_IFNG_24H_MICROGLIA_U	-0,038	0,305	0,03571	1
MODULE_355	-0,038	0,306	0,78542	1
chr7p22	-0,038	0,306	0,08793	1
REACTOME_SPHINGOLIPID_DE_NOVO_BIOSYN	0,038	0,307	0,58199	1
AGCYRWTTC_UNKNOWN	-0,037	0,307	0,00441	1
HOUSTIS_ROS	0,037	0,307	0,30032	1
chr17p12	-0,037	0,308	0,1804	1
HOWLIN_CITED1_TARGETS_1_DN	-0,037	0,308	0,05211	1
NOUSHMEHR_GBM_GERMLINE_MUTATED	-0,037	0,308	0,50197	1
TURJANSKI_MAPK11_TARGETS	-0,037	0,308	0,10534	1
PURINE_RIBONUCLEOTIDE_METABOLIC_PROCE	0,037	0,308	0,44163	1
APPEL_IMATINIB_RESPONSE	0,037	0,308	0,21897	1
XU_AKT1_TARGETS_6HR	-0,037	0,309	0,24732	1
SMID_BREAST_CANCER_ERBB2_UP	0,037	0,309	0,24477	1
GSE20366_EX_VIVO_VS_HOMEOSTATIC_CON	-0,037	0,309	0,0805	1
KANG_DOXORUBICIN_RESISTANCE_DN	-0,037	0,31	0,67029	1
GSE1460_DP_THYMOCYTE_VS_NAIVE_CD4_	-0,037	0,31	0,03105	1
GLAND_DEVELOPMENT	-0,037	0,31	0,04308	1

CARBOXYLIC_ACID_METABOLIC_PROCESS	-0,037	0,31	0,01642	1
REACTOME_RECRUITMENT_OF_NUMA_TO_MIT	-0,037	0,311	0,0953	1
REACTOME_PROTEOLYTIC_CLEAVAGE_OF_SN	0,037	0,311	0,74298	1
GLIOGENESIS	-0,037	0,311	0,07345	1
RESPONSE_TO_BACTERIUM	-0,037	0,311	0,29758	1
REACTOME_SIGNAL_REGULATORY_PROTEIN_S	-0,037	0,312	0,51675	1
GNF2_PAK2	-0,037	0,312	0,39703	1
GARGALOVIC_RESPONSE_TO_OXIDIZED_PHOS	-0,037	0,312	0,18676	1
chr13q13	-0,037	0,312	0,0507	1
chr5q12	-0,037	0,312	0,05232	1
SPHINGOLIPID_BIOSYNTHETIC_PROCESS	-0,037	0,313	0,06847	1
ENERGY_DERIVATION_BY_OXIDATION_OF_OR(	-0,037	0,313	0,14556	1
NUMATA_CSF3_SIGNALING_VIA_STAT3	-0,037	0,313	0,08744	1
REACTOME_GLYCOPROTEIN_HORMONES	-0,037	0,313	0,20381	1
HENDRICKS_SMARCA4_TARGETS_DN	-0,037	0,314	0,0218	1
DEMAGALHAES_AGING_UP	-0,037	0,314	0,32522	1
GSE16755_CTRL_VS_IFNA_TREATED_MAC_U	-0,037	0,314	0,02981	1
HOEGERKORP_CD44_TARGETS_DIRECT_DN	0,037	0,315	0,92731	1
CHEOK_RESPONSE_TO_MERCAPTOPURINE_AN	0,037	0,315	0,28153	1
DNA_CATABOLIC_PROCESS	-0,037	0,315	0,60699	1
ACETYLGALACTOSAMINYLTRANSFERASE_ACT	-0,037	0,315	0,30036	1
BIOCARTA_CTL_PATHWAY	-0,037	0,316	0,3757	1
GSE29618_PRE_VS_DAY7_FLU_VACCINE_MD	-0,037	0,316	0,04918	1
LEE_LIVER_CANCER_SURVIVAL_UP	0,037	0,316	0,40663	1
REACTOME_AQUAPORIN_MEDIATED_TRANSPO	-0,037	0,318	0,06701	1
ROZANOV_MMP14_CORRELATED	-0,037	0,318	0,52122	1
AMINE_METABOLIC_PROCESS	-0,037	0,318	0,0835	1
KEGG_PROXIMAL_TUBULE_BICARBONATE_REC	0,037	0,319	0,63658	1
STTTCRNTTT_V$IRF_Q6	-0,037	0,319	0,11413	1
NUCLEOBASENUCLEOSIDENUCLEOTIDE_AND_N	-0,037	0,319	0,56546	1
chr17q12	-0,036	0,32	0,12478	1
GSE3982_EOSINOPHIL_VS_NEUTROPHIL_DN	-0,036	0,32	0,18054	1
SARTIPY_NORMAL_AT_INSULIN_RESISTANCE_	-0,036	0,32	0,00154	1
HOLLEMAN_DAUNORUBICIN_B_ALL_DN	-0,036	0,32	0,02648	1
BIOCARTA_LYM_PATHWAY	-0,036	0,321	0,66996	1
GSE18791_UNSTIM_VS_NEWCATSLE_VIRUS_	-0,036	0,321	0,44944	1
REACTOME_OLFACTORY_SIGNALING_PATHWA	-0,036	0,321	0,45062	1
chr4p11	0,036	0,322	0,54124	1
GSE11864_CSF1_IFNG_VS_CSF1_PAM3CYS_	-0,036	0,322	0,15914	1
DETECTION_OF_STIMULUS_INVOLVED_IN_SEN	-0,036	0,322	0,00371	1
BIOCARTA_LAIR_PATHWAY	-0,036	0,322	0,17989	1
GSE17721_LPS_VS_PAM3CSK4_16H_BMDM_	-0,036	0,323	0,19889	1

RHYTHMIC_PROCESS	-0,036	0,324	0,10084	1
ZHAN_MULTIPLE_MYELOMA_CD1_AND_CD2_	-0,036	0,324	0,01923	1
LEE_SP4_THYMOCYTE	-0,036	0,324	0,0204	1
CHEN_ETV5_TARGETS_SERTOLI	-0,036	0,325	0,64405	1
chr5q14	0,036	0,325	0,62803	1
MODULE_519	-0,036	0,325	0,34408	1
COFACTOR_TRANSPORT	0,036	0,326	0,77586	1
WENG_POR_TARGETS_LIVER_DN	-0,036	0,326	0,03363	1
CELL_STRUCTURE_DISASSEMBLY_DURING_AP	-0,036	0,326	0,39558	1
GSE17721_PAM3CSK4_VS_GADIQUIMOD_24H	-0,036	0,327	0,26632	1
GSE22886_CTRL_VS_LPS_24H_DC_UP	-0,036	0,327	0,1927	1
MODULE_265	-0,036	0,327	0,21569	1
DOUGLAS_BMI1_TARGETS_DN	-0,036	0,328	0,10352	1
MICROTUBULE_BASED_MOVEMENT	-0,036	0,328	0,77333	1
ACTIVE_TRANSMEMBRANE_TRANSPORTER_AC	-0,036	0,328	0,13297	1
MODULE_445	-0,036	0,329	0,45391	1
chr3p14	0,036	0,33	0,55314	1
MODULE_313	-0,036	0,33	0,00703	1
GNF2_CASP8	-0,036	0,33	0,26947	1
KYNG_ENVIRONMENTAL_STRESS_RESPONSE_	-0,036	0,331	0,03674	1
OXIDOREDUCTASE_ACTIVITY_ACTING_ON_PEI	-0,036	0,331	0,12008	1
GSE339_CD4POS_VS_CD8POS_DC_DN	-0,036	0,331	0,66457	1
MODULE_295	0,036	0,331	0,30164	1
MARIADASON_RESPONSE_TO_BUTYRATE_CUF	-0,036	0,331	0,14023	1
MORI_MATURE_B_LYMPHOCYTE_UP	-0,036	0,332	0,19939	1
CHUNG_BLISTER_CYTOTOXICITY_UP	-0,036	0,332	0,62094	1
JIANG_AGING_HYPOTHALAMUS_UP	0,036	0,332	0,30873	1
V$AR_Q2	-0,036	0,333	0,05639	1
PID_AR_PATHWAY	-0,036	0,333	0,02632	1
chr16q12	-0,036	0,333	0,58887	1
GGCGGCA,MIR-371	-0,035	0,334	0,87964	1
REACTOME_ACTIVATION_OF_CHAPERONE_GE	0,035	0,334	0,18121	1
SODIUM_ION_TRANSPORT	-0,035	0,334	0,00027	1
GCM_ERBB2IP	0,035	0,334	0,46747	1
REGULATION_OF_HOMEOSTATIC_PROCESS	0,035	0,334	0,63601	1
GSE17721_12H_VS_24H_PAM3CSK4_BMDM_	-0,035	0,335	0,05979	1
PID_EPHA2_FWDPATHWAY	-0,035	0,335	0,23316	1
REACTOME_REGULATION_OF_AMPK_ACTIVITY	-0,035	0,335	0,14425	1
DUNNE_TARGETS_OF_AML1_MTG8_FUSION_I	-0,035	0,335	0,09621	1
REACTOME_REGULATION_OF_HYPOXIA_INDUC	-0,035	0,335	0,79434	1
EPITHELIAL_CELL_DIFFERENTIATION	-0,035	0,335	0,87894	1
CAFFAREL_RESPONSE_TO_THC_8HR_3_UP	0,035	0,337	0,74162	1

PID_ANTHRAXPATHWAY	-0,035	0,337	0,135	1
HESS_TARGETS_OF_HOXA9_AND_MEIS1_DN	-0,035	0,337	0,53662	1
GSE18791_CTRL_VS_NEWCASTLE_VIRUS_DC	-0,035	0,337	0,57153	1
GSE22886_NAIVE_BCELL_VS_MONOCYTE_DN	0,035	0,338	0,13592	1
HOXA9_DN.V1_UP	-0,035	0,338	0,22551	1
SMALL_CONJUGATING_PROTEIN_SPECIFIC_PR	-0,035	0,338	0,66445	1
BIOCARTA_CARM_ER_PATHWAY	0,035	0,339	0,42019	1
GSE360_T_GONDII_VS_M_TUBERCULOSIS_DC	-0,035	0,339	0,35239	1
GSE37416_0H_VS_24H_F_TULARENSIS_LVS	-0,035	0,339	0,22459	1
VANDESLUIS_COMMD1_TARGETS_GROUP_4_	-0,035	0,339	0,31718	1
MARKEY_RB1_ACUTE_LOF_UP	-0,035	0,339	0,45029	1
GSE36476_YOUNG_VS_OLD_DONOR_MEMOR	-0,035	0,339	0,00965	1
NEGATIVE_REGULATION_OF_SECRETION	-0,035	0,339	0,18265	1
SAKAI_CHRONIC_HEPATITIS_VS_LIVER_CANC	-0,035	0,34	0,02967	1
PID_CDC42_REG_PATHWAY	-0,035	0,34	0,04935	1
GSE31082_DN_VS_CD8_SP_THYMOCYTE_DN	-0,035	0,34	0,04612	1
BALLIF_DEVELOPMENTAL_DISABILITY_P16_P	-0,035	0,34	0,19175	1
YGACNNYACAR_UNKNOWN	-0,035	0,341	0,01984	1
GSE17721_0.5H_VS_8H_CPG_BMDM_DN	-0,035	0,341	0,40243	1
HERNANDEZ_ABERRANT_MITOSIS_BY_DOCET	-0,035	0,342	0,82047	1
GSE3982_MAST_CELL_VS_TH1_UP	-0,035	0,342	0,08925	1
REACTOME_ACTIVATION_OF_CHAPERONE_GE	-0,035	0,342	0,88703	1
DEN_INTERACT_WITH_LCA5	-0,035	0,342	0,34766	1
WANG_RESPONSE_TO_FORSKOLIN_DN	-0,035	0,343	0,15424	1
KEGG_PYRUVATE_METABOLISM	0,035	0,343	0,26527	1
DOUBLE_STRANDED_RNA_BINDING	0,035	0,344	0,56145	1
GSE22886_NAIVE_CD4_TCELL_VS_12H_ACT	-0,035	0,344	0,00022	1
GSE9988_ANTI_TREM1_VS_LOW_LPS_MONO	-0,035	0,344	0,53239	1
DELACROIX_RAR_TARGETS_UP	-0,035	0,344	0,09305	1
MODULE_170	-0,035	0,344	0,24742	1
chr2q37	-0,035	0,344	0,14153	1
MODULE_28	-0,035	0,344	0,30357	1
ROETH_TERT_TARGETS_DN	-0,035	0,345	0,27211	1
EHLERS_ANEUPLOIDY_DN	-0,035	0,345	0,88237	1
DAVIES_MULTIPLE_MYELOMA_VS_MGUS_DN	-0,035	0,345	0,40669	1
CELL_SOMA	-0,035	0,346	0,0162	1
WALLACE_PROSTATE_CANCER_RACE_DN	-0,035	0,346	0,03943	1
GSE39820_TGFBETA1_IL6_VS_TGFBETA1_IL	-0,035	0,346	0,25852	1
chr13q34	-0,035	0,346	0,03789	1
PARK_APL_PATHOGENESIS_DN	-0,035	0,346	0,72467	1
chr10p11	-0,035	0,347	0,01402	1
YRCCAKNNGNCGC_UNKNOWN	-0,035	0,347	0,00024	1

REACTOME_ANTIGEN_PROCESSING_CROSS_PF	-0,035	0,347	0,68276	1
ATPASE_ACTIVITY_COUPLED_TO_TRANSMEM	0,035	0,347	0,74246	1
MIKKELSEN_MCV6_LCP_WITH_H3K4ME3	-0,034	0,348	0,28219	1
BRAIN_DEVELOPMENT	-0,034	0,349	0,0863	1
GCM_GSPT1	-0,034	0,349	0,08124	1
KEGG_REGULATION_OF_AUTOPHAGY	-0,034	0,349	0,35098	1
REACTOME_ASSOCIATION_OF_TRIC_CCT_WIT	-0,034	0,349	0,56145	1
SHEDDEN_LUNG_CANCER_GOOD_SURVIVAL_/	0,034	0,349	0,28093	1
ONGUSAHA_BRCA1_TARGETS_UP	-0,034	0,35	0,4816	1
FRASOR_TAMOXIFEN_RESPONSE_DN	0,034	0,35	0,03527	1
DER_IFN_ALPHA_RESPONSE_UP	-0,034	0,351	0,49848	1
STARK_HYPPOCAMPUS_22Q11_DELETION_DN	-0,034	0,351	0,27866	1
REACTOME_GAP_JUNCTION_DEGRADATION	-0,034	0,351	0,4627	1
GSE339_CD8POS_VS_CD4CD8DN_DC_UP	-0,034	0,352	0,5118	1
EXCITATORY_EXTRACELLULAR_LIGAND_GATE	-0,034	0,352	0,19973	1
GSE360_T_GONDII_VS_B_MALAYI_LOW_DOS	-0,034	0,352	0,07677	1
GSE3337_4H_VS_16H_IFNG_IN_CD8POS_DC	-0,034	0,352	0,18311	1
GSE17721_PAM3CSK4_VS_GADIQUIMOD_0.5	-0,034	0,352	0,0068	1
RNA_DEPENDENT_ATPASE_ACTIVITY	-0,034	0,352	0,9749	1
GSE20715_WT_VS_TLR4_KO_LUNG_DN	-0,034	0,352	0,00141	1
WINNEPENNINCKX_MELANOMA_METASTASIS_	-0,034	0,353	0,01951	1
REACTOME_TRANSCRIPTIONAL_REGULATION_	-0,034	0,353	0,04718	1
PID_RET_PATHWAY	-0,034	0,354	0,00312	1
GCM_ATM	-0,034	0,354	0,12379	1
NUCLEAR_ORGANIZATION_AND_BIOGENESIS	-0,034	0,354	0,37694	1
GSE9988_LOW_LPS_VS_VEHICLE_TREATED_	-0,034	0,355	0,24489	1
GSE1432_CTRL_VS_IFNG_24H_MICROGLIA_D	-0,034	0,355	0,62919	1
WEBER_METHYLATED_HCP_IN_FIBROBLAST_U	0,034	0,355	0,88573	1
MODULE_60	-0,034	0,356	0,02033	1
REACTOME_GENERIC_TRANSCRIPTION_PATHW	-0,034	0,356	0,0284	1
RODRIGUES_NTN1_TARGETS_UP	-0,034	0,356	0,00287	1
REACTOME_NEGATIVE_REGULATORS_OF_RIG_	-0,034	0,357	0,39779	1
INOSITOL_OR_PHOSPHATIDYLINOSITOL_KINAS	0,034	0,357	0,56276	1
PID_TCRJNKPATHWAY	-0,034	0,357	0,12967	1
GSE39820_CTRL_VS_TGFBETA1_IL6_CD4_T	-0,034	0,357	0,15455	1
ACTIVATION_OF_MAPK_ACTIVITY	-0,034	0,357	0,19427	1
TARTE_PLASMA_CELL_VS_B_LYMPHOCYTE_U	-0,034	0,357	0,95967	1
GSE37416_CTRL_VS_6H_F_TULARENSIS_LVS	-0,034	0,358	0,28651	1
MORI_PLASMA_CELL_UP	-0,034	0,358	0,90281	1
PATTERSON_DOCETAXEL_RESISTANCE	-0,034	0,359	0,11803	1
REACTOME_INTERFERON_SIGNALING	-0,034	0,359	0,57963	1
chr6q24	0,034	0,36	0,03556	1

NELSON_RESPONSE_TO_ANDROGEN_UP	-0,034	0,36	0,04522	1
AXON	-0,034	0,36	0,00098	1
RADAEVA_RESPONSE_TO_IFNA1_UP	-0,034	0,361	0,4376	1
BIOCARTA_AKT_PATHWAY	0,034	0,361	0,79051	1
REACTOME_PURINE_RIBONUCLEOSIDE_MONOF	-0,033	0,362	0,81537	1
MORF_CASP10	-0,033	0,362	0,02557	1
GSE17721_4_VS_24H_GARDIQUIMOD_BMDM_	-0,033	0,362	0,04473	1
ST_GAQ_PATHWAY	0,033	0,362	0,86422	1
GNF2_MAP2K3	-0,033	0,362	0,43058	1
LUI_THYROID_CANCER_CLUSTER_2	0,033	0,363	0,29614	1
PROTEIN_DEACETYLASE_ACTIVITY	0,033	0,363	0,92182	1
chr3p	0,033	0,363	0,39456	1
FUJII_YBX1_TARGETS_UP	-0,033	0,364	0,15311	1
GSE17721_0.5H_VS_4H_POLYIC_BMDM_DN	-0,033	0,364	0,22707	1
REGULATION_OF_CYTOSKELETON_ORGANIZAT	-0,033	0,365	0,00301	1
SYED_ESTRADIOL_RESPONSE	-0,033	0,365	0,40248	1
HUMMERICH_BENIGN_SKIN_TUMOR_UP	-0,033	0,365	0,21415	1
MONOSACCHARIDE_BINDING	-0,033	0,366	0,51132	1
MORI_PRE_BI_LYMPHOCYTE_DN	-0,033	0,366	0,12055	1
GSE14350_TREG_VS_TEFF_UP	-0,033	0,366	0,05713	1
PROTEASE_INHIBITOR_ACTIVITY	-0,033	0,367	0,07812	1
RGTTAMWNATT_V$HNF1_01	-0,033	0,367	0,01947	1
GNF2_TST	0,033	0,367	0,31638	1
BIOCARTA_HIF_PATHWAY	-0,033	0,367	0,85853	1
STK33_UP	-0,033	0,368	0,21139	1
GSE37416_CTRL_VS_12H_F_TULARENSIS_L\	-0,033	0,368	0,21506	1
BIOCARTA_FREE_PATHWAY	-0,033	0,369	0,60984	1
MODULE_400	-0,033	0,369	0,06809	1
HYDROLASE_ACTIVITY_HYDROLYZING_O_GLY	0,033	0,369	0,79717	1
POTTI_DOCETAXEL_SENSITIVITY	-0,033	0,369	0,64331	1
REGULATION_OF_MEMBRANE_POTENTIAL	-0,033	0,37	0,1313	1
chr17p11	0,033	0,371	0,90423	1
MODULE_454	-0,033	0,371	0,00613	1
chr22q11	-0,033	0,371	0,34214	1
CLIMENT_BREAST_CANCER_COPY_NUMBER_U	-0,033	0,372	0,00537	1
MODULE_478	-0,033	0,372	0,4821	1
BYSTROEM_CORRELATED_WITH_IL5_DN	-0,033	0,373	0,34918	1
REACTOME_GENERATION_OF_SECOND_MESSE	-0,033	0,374	0,20504	1
chr20p13	-0,033	0,374	0,38589	1
chr2q	-0,033	0,374	0,87136	1
YAMANAKA_GLIOBLASTOMA_SURVIVAL_DN	0,033	0,374	0,23398	1
REGULATION_OF_SECRETION	-0,033	0,375	0,07678	1

GSE27786_NKCELL_VS_NKTCELL_UP	-0,033	0,375	0,49022	1
GNF2_PTPRC	-0,033	0,375	0,28157	1
VALK_AML_CLUSTER_5	0,033	0,375	0,35878	1
GCM_MYCL1	-0,033	0,375	0,05321	1
CAIRO_LIVER_DEVELOPMENT_DN	-0,033	0,376	0,17991	1
MODULE_300	-0,032	0,376	0,62409	1
KUROKAWA_LIVER_CANCER_CHEMOTHERAPY_	-0,032	0,376	0,00742	1
NICOTINIC_ACETYLCHOLINE_ACTIVATED_CAT	-0,032	0,376	0,42298	1
NICOTINIC_ACETYLCHOLINE_GATED_RECEPTO	-0,032	0,376	0,42298	1
GSE36392_TYPE_2_MYELOID_VS_EOSINOPHI	-0,032	0,377	0,36346	1
SAKAI_CHRONIC_HEPATITIS_VS_LIVER_CANC	-0,032	0,377	0,7383	1
GSE17721_POLYIC_VS_PAM3CSK4_4H_BMDM	-0,032	0,377	0,23892	1
HOFMANN_MYELODYSPLASTIC_SYNDROM_LO	0,032	0,377	0,05483	1
AGCGCTT,MIR-518F,MIR-518E,MIR-518A	-0,032	0,377	0,03627	1
SYNAPSE_PART	-0,032	0,377	0,03461	1
POSITIVE_REGULATION_OF_DNA_BINDING	-0,032	0,378	0,15986	1
BRUNO_HEMATOPOIESIS	-0,032	0,379	0,50204	1
GSE24142_EARLY_THYMIC_PROGENITOR_VS_	-0,032	0,379	0,22124	1
SA_FAS_SIGNALING	-0,032	0,379	0,16774	1
GSE22886_DAY0_VS_DAY1_MONOCYTE_IN_	-0,032	0,38	0,09369	1
LIU_SOX4_TARGETS_UP	-0,032	0,381	0,43457	1
REACTOME_INHIBITION_OF_INSULIN_SECRETIC	-0,032	0,381	0,01698	1
MOOTHA_GLUCONEOGENESIS	0,032	0,381	0,25408	1
GSE17721_CTRL_VS_POLYIC_1H_BMDM_UP	-0,032	0,381	0,19632	1
GSE14769_UNSTIM_VS_80MIN_LPS_BMDM_U	-0,032	0,381	0,06451	1
MITOCHONDRIAL_OUTER_MEMBRANE	0,032	0,382	0,44913	1
GCM_MLL	-0,032	0,383	0,1051	1
PROTEIN_SERINE_THREONINE_PHOSPHATASE_	0,032	0,383	0,3105	1
HETEROCYCLE_METABOLIC_PROCESS	0,032	0,385	0,07434	1
chr4q32	-0,032	0,386	0,06509	1
PUJANA_BREAST_CANCER_WITH_BRCA1_MU	-0,032	0,386	0,13792	1
GSE9988_ANTI_TREM1_VS_CTRL_TREATED_	-0,032	0,387	0,21076	1
POSITIVE_REGULATION_OF_BINDING	-0,032	0,387	0,19816	1
GSE13411_IGM_VS_SWITCHED_MEMORY_BCE	-0,032	0,387	0,42156	1
KUROZUMI_RESPONSE_TO_ONCOCYTIC_VIRUS	-0,032	0,387	0,58282	1
BLOOD_COAGULATION	-0,032	0,388	0,00518	1
chr5q13	0,032	0,388	0,18098	1
NIKOLSKY_BREAST_CANCER_8Q23_Q24_AMI	-0,032	0,389	0,56185	1
BURTON_ADIPOGENESIS_6	-0,032	0,39	0,23964	1
ITO_PTTG1_TARGETS_UP	-0,032	0,39	0,22143	1
BANDRES_RESPONSE_TO_CARMUSTIN_WITHC	-0,032	0,39	0,30253	1
CELL_CORTEX_PART	0,032	0,391	0,42429	1

KUROKAWA_LIVER_CANCER_CHEMOTHERAPY_	-0,031	0,391	0,91764	1
GCM_FCGR2B	-0,031	0,391	0,00237	1
GSE14769_40MIN_VS_360MIN_LPS_BMDM_[	-0,031	0,391	0,16959	1
ACCAATC,MIR-509	0,031	0,391	0,75887	1
GNF2_CD53	-0,031	0,391	0,45312	1
BOSCO_INTERFERON_INDUCED_ANTIVIRAL_M(	-0,031	0,392	0,56549	1
ELECTRON_TRANSPORT_GO_0006118	-0,031	0,392	0,16874	1
SAENZ_DETOX_PATHWAY_AND_CARCINOGEN	0,031	0,392	0,40032	1
NAKAJIMA_EOSINOPHIL	0,031	0,392	0,97329	1
ICHIBA_GRAFT_VERSUS_HOST_DISEASE_35D_	-0,031	0,393	0,59328	1
MODULE_575	-0,031	0,393	0,12159	1
MODULE_96	-0,031	0,393	0,17252	1
WENG_POR_TARGETS_GLOBAL_UP	0,031	0,394	0,20029	1
MORF_ARAF1	-0,031	0,394	0,10091	1
LIPID_METABOLIC_PROCESS	-0,031	0,395	0,04686	1
ZHENG_FOXP3_TARGETS_UP	-0,031	0,395	0,49372	1
GSE17721_0.5H_VS_12H_PAM3CSK4_BMDM	-0,031	0,395	0,67602	1
REACTOME_GLYCOSPHINGOLIPID_METABOLISM	0,031	0,395	0,63424	1
GSE19825_CD24LOW_VS_IL2RA_HIGH_DAY3	-0,031	0,396	0,05156	1
REACTOME_SHC_RELATED_EVENTS	-0,031	0,396	0,53644	1
METALLOEXOPEPTIDASE_ACTIVITY	-0,031	0,396	0,03394	1
TRANSFERASE_ACTIVITY_TRANSFERRING_GRO	0,031	0,397	0,77822	1
chr11q14	-0,031	0,397	0,60037	1
VISALA_RESPONSE_TO_HEAT_SHOCK_AND_A	-0,031	0,397	0,12086	1
MODULE_227	0,031	0,398	0,18729	1
NIELSEN_GIST_AND_SYNOVIAL_SARCOMA_DN	-0,031	0,399	0,47691	1
GAURNIER_PSMD4_TARGETS	-0,031	0,399	0,71364	1
POSITIVE_REGULATION_OF_TRANSPORT	0,031	0,399	0,86409	1
chr14q12	0,031	0,399	0,03519	1
BRACHAT_RESPONSE_TO_CAMPTOTHECIN_UP	0,031	0,4	0,52682	1
PROTEIN_EXPORT_FROM_NUCLEUS	-0,031	0,4	0,68935	1
DORSAM_HOXA9_TARGETS_DN	0,031	0,4	0,27394	1
chr3q23	-0,031	0,401	0,63014	1
FREDERICK_PRKCI_TARGETS	-0,031	0,401	0,97906	1
REACTOME_ENDOSOMAL_VACUOLAR_PATHW	0,031	0,402	0,16325	1
chr9q33	-0,031	0,402	0,02904	1
KEGG_RETINOL_METABOLISM	0,031	0,402	0,87548	1
FARDIN_HYPOXIA_11	0,031	0,402	0,23408	1
LEF1_UP.V1_DN	-0,031	0,402	0,00778	1
REACTOME_ACYL_CHAIN_REMODELLING_OF_	0,031	0,402	0,64508	1
TAKEDA_TARGETS_OF_NUP98_HOXA9_FUSIC	-0,031	0,402	0,13302	1
GSE17974_0H_VS_4H_IN_VITRO_ACT_CD4_	-0,031	0,403	0,01254	1

COAGULATION	-0,031	0,403	0,00473	1
AEROBIC_RESPIRATION	-0,031	0,404	0,59517	1
PALOMERO_GSI_SENSITIVITY_DN	-0,031	0,404	0,77104	1
PENG_GLUTAMINE_DEPRIVATION_UP	-0,031	0,405	0,06158	1
GSE17721_CTRL_VS_PAM3CSK4_8H_BMDM_	-0,031	0,405	0,12969	1
GNF2_INPP5D	-0,031	0,405	0,42811	1
MASSARWEH_TAMOXIFEN_RESISTANCE_UP	0,031	0,405	0,86431	1
chrxp21	-0,031	0,406	0,82965	1
MARTINELLI_IMMATURE_NEUTROPHIL_UP	-0,03	0,406	0,47614	1
NIKOLSKY_BREAST_CANCER_10Q22_AMPLIC(	-0,03	0,407	0,03402	1
BARRIER_COLON_CANCER_RECURRENCE_UP	-0,03	0,407	0,74357	1
COENZYME_BIOSYNTHETIC_PROCESS	0,03	0,408	0,02498	1
REACTOME_ENERGY_DEPENDENT_REGULATIO	-0,03	0,409	0,15841	1
MATZUK_SPERMATOZOA	-0,03	0,409	0,00133	1
MEMBRANE_ORGANIZATION_AND_BIOGENESIS	-0,03	0,41	0,01848	1
LANDIS_ERBB2_BREAST_PRENEOPLASTIC_DN	-0,03	0,41	0,08014	1
LIU_VAV3_PROSTATE_CARCINOGENESIS_UP	-0,03	0,41	0,17181	1
GSE3982_DC_VS_TH2_UP	-0,03	0,41	0,33757	1
REACTOME_OXYGEN_DEPENDENT_PROLINE_H	-0,03	0,41	0,59009	1
GAZDA_DIAMOND_BLACKFAN_ANEMIA_ERYTI	-0,03	0,411	0,34307	1
CELLULAR_CATABOLIC_PROCESS	0,03	0,411	0,56577	1
STEROID_DEHYDROGENASE_ACTIVITY	0,03	0,411	0,67838	1
MOOTHA_VOXPHOS	0,03	0,411	0,37165	1
GATGKMRGCG_UNKNOWN	-0,03	0,411	0,0138	1
TERAMOTO_OPN_TARGETS_CLUSTER_8	0,03	0,412	0,79481	1
ZHAN_MULTIPLE_MYELOMA_DN	-0,03	0,412	0,64361	1
REGULATION_OF_DNA_BINDING	-0,03	0,412	0,14707	1
BIOCARTA_TNFR1_PATHWAY	-0,03	0,412	0,47196	1
MODULE_462	-0,03	0,413	0,04684	1
NIKOLSKY_BREAST_CANCER_6P24_P22_AMP	-0,03	0,414	0,7478	1
HEME_BIOSYNTHETIC_PROCESS	0,03	0,414	0,45036	1
HUANG_GATA2_TARGETS_UP	-0,03	0,415	0,2758	1
ISHIKAWA_STING_SIGNALING	-0,03	0,415	0,35641	1
MODULE_396	0,03	0,415	0,72801	1
NEURON_PROJECTION	-0,03	0,416	0,0067	1
KHETCHOUMIAN_TRIM24_TARGETS_DN	0,03	0,416	0,14167	1
GSE10325_LUPUS_BCELL_VS_LUPUS_MYELO	0,03	0,416	0,46662	1
chr3p22	0,03	0,416	0,93891	1
CHAPERONE_BINDING	-0,03	0,416	0,89675	1
VIETOR_IFRD1_TARGETS	-0,03	0,417	0,2687	1
BERENJENO_TRANSFORMED_BY_RHOA_REVEF	-0,03	0,418	0,66975	1
TIAN_BHLHA15_TARGETS	-0,03	0,418	0,09268	1

REACTOME_BOTULINUM_NEUROTOXICITY	0,03	0,418	0,49072	1
BIOPOLYMER_BIOSYNTHETIC_PROCESS	-0,03	0,419	0,07024	1
OSAWA_TNF_TARGETS	-0,03	0,419	0,60681	1
REACTOME_BILE_ACID_AND_BILE_SALT_MET	0,03	0,42	0,8473	1
BIOCARTA_IL1R_PATHWAY	-0,03	0,42	0,41516	1
ORGANELLE_INNER_MEMBRANE	-0,03	0,421	0,71389	1
GAZDA_DIAMOND_BLACKFAN_ANEMIA_MYEL	0,03	0,421	0,9229	1
GSE32423_IL7_VS_IL7_IL4_NAIVE_CD8_TCE	-0,029	0,421	0,06017	1
REACTOME_PTM_GAMMA_CARBOXYLATION_H	-0,029	0,422	0,0713	1
GSE29618_PDC_VS_MDC_DAY7_FLU_VACCII	-0,029	0,422	0,38241	1
REACTOME_TRANSCRIPTION_COUPLED_NER_T	-0,029	0,422	0,55909	1
chr5q15	0,029	0,422	0,68217	1
MOOTHA_MITOCHONDRIA	0,029	0,422	0,58597	1
REACTOME_COMPLEMENT_CASCADE	0,029	0,423	0,75581	1
SYNAPSE	-0,029	0,424	0,03236	1
REACTOME_IRON_UPTAKE_AND_TRANSPORT	-0,029	0,424	0,89438	1
MODULE_404	0,029	0,425	0,68513	1
DODD_NASOPHARYNGEAL_CARCINOMA_UP	-0,029	0,425	0,00173	1
MANN_RESPONSE_TO_AMIFOSTINE_UP	0,029	0,425	0,44995	1
LINDGREN_BLADDER_CANCER_CLUSTER_2A_	-0,029	0,425	0,21538	1
HOEGERKORP_CD44_TARGETS_DIRECT_UP	-0,029	0,425	0,11767	1
MODULE_429	-0,029	0,427	0,33033	1
MULLIGHAN_MLL_SIGNATURE_1_UP	-0,029	0,427	0,31579	1
TORCHIA_TARGETS_OF_EWSR1_FLI1_FUSION	0,029	0,428	0,92209	1
TAGAACC,MIR-182	-0,029	0,428	0,06162	1
REACTOME_AMINE_DERIVED_HORMONES	-0,029	0,429	0,10162	1
MONTERO_THYROID_CANCER_POOR_SURVIVA	-0,029	0,429	0,11451	1
REACTOME_ACYL_CHAIN_REMODELLING_OF_	0,029	0,43	0,74968	1
LIU_LIVER_CANCER	0,029	0,43	0,25406	1
GSE24142_EARLY_THYMIC_PROGENITOR_VS_	-0,029	0,43	0,178	1
MODULE_209	-0,029	0,43	0,29458	1
CHEN_LIVER_METABOLISM_QTL_CIS	-0,029	0,431	0,29383	1
CARBOHYDRATE_KINASE_ACTIVITY	0,029	0,431	0,0896	1
BIOCARTA_LONGEVITY_PATHWAY	-0,029	0,431	0,07546	1
BIOCARTA_PROTEASOME_PATHWAY	-0,029	0,431	0,16555	1
KRIGE_RESPONSE_TO_TOSEDOSTAT_24HR_U	-0,029	0,431	0,2634	1
YU_MYC_TARGETS_DN	-0,029	0,432	0,17196	1
BETA_TUBULIN_BINDING	-0,029	0,432	0,65942	1
REGULATION_OF_BINDING	-0,029	0,432	0,09813	1
MARIADASON_RESPONSE_TO_CURCUMIN_SUL	0,029	0,432	0,92394	1
KEGG_ALZHEIMERS_DISEASE	-0,029	0,432	0,46321	1
ZIRN_TRETINOIN_RESPONSE_UP	-0,029	0,433	0,0249	1

SCHAEFFER_PROSTATE_DEVELOPMENT_AND_	-0,029	0,433	0,00129	1
GCM_PTPRD	-0,029	0,433	0,17984	1
REACTOME_PASSIVE_TRANSPORT_BY_AQUAI	-0,029	0,433	0,93912	1
chr14q11	-0,029	0,433	0,04539	1
COVALENT_CHROMATIN_MODIFICATION	-0,029	0,434	0,09174	1
CELL_MATRIX_JUNCTION	-0,029	0,435	0,14735	1
KANG_FLUOROURACIL_RESISTANCE_UP	-0,029	0,435	0,22451	1
REACTOME_REGULATION_OF_IFNG_SIGNALIN(	-0,029	0,436	0,85998	1
WACKER_HYPOXIA_TARGETS_OF_VHL	-0,029	0,436	0,21191	1
VALK_AML_CLUSTER_13	-0,029	0,436	0,0034	1
BUDHU_LIVER_CANCER_METASTASIS_DN	-0,029	0,437	0,96849	1
APOPTOTIC_NUCLEAR_CHANGES	-0,029	0,437	0,58596	1
NIKOLSKY_BREAST_CANCER_11Q12_Q14_AI	-0,029	0,437	0,26075	1
NUCLEAR_UBIQUITIN_LIGASE_COMPLEX	-0,028	0,437	0,58435	1
CUI_TCF21_TARGETS_DN	-0,028	0,438	0,10758	1
BIOCARTA_AHSP_PATHWAY	0,028	0,438	0,69861	1
LIU_PROSTATE_CANCER_UP	0,028	0,438	0,68286	1
MODULE_484	-0,028	0,438	0,12907	1
GENERATION_OF_A_SIGNAL_INVOLVED_IN_C	-0,028	0,439	0,02607	1
CALCIUM_ION_TRANSMEMBRANE_TRANSPORT	0,028	0,439	0,63168	1
SCHAEFFER_SOX9_TARGETS_IN_PROSTATE_I	-0,028	0,439	0,1225	1
WAKABAYASHI_ADIPOGENESIS_PPARG_RXRA_	0,028	0,439	0,57891	1
CALCIUM_CHANNEL_ACTIVITY	-0,028	0,44	0,00899	1
MODULE_349	-0,028	0,44	0,44577	1
CRGAARNNNNCGA_UNKNOWN	-0,028	0,44	0,00885	1
GNF2_CYP2B6	0,028	0,44	0,50648	1
REGULATION_OF_ACTIN_POLYMERIZATION_AI	-0,028	0,44	0,14758	1
NADLER_OBESITY_UP	-0,028	0,441	0,63806	1
GSE17721_LPS_VS_CPG_16H_BMDM_UP	-0,028	0,441	0,08984	1
GCM_DPF2	-0,028	0,441	0,03495	1
MORF_PPP6C	-0,028	0,441	0,95802	1
GSE29618_BCELL_VS_MDC_DAY7_FLU_VAC	-0,028	0,442	0,4432	1
LIAN_LIPA_TARGETS_6M	-0,028	0,442	0,72422	1
KEGG_O_GLYCAN_BIOSYNTHESIS	-0,028	0,443	0,41273	1
GSE13485_CTRL_VS_DAY7_YF17D_VACCINE	-0,028	0,443	0,75787	1
chr6q15	-0,028	0,443	0,81443	1
TIAN_TNF_SIGNALING_NOT_VIA_NFKB	-0,028	0,444	0,01598	1
MONOSACCHARIDE_TRANSMEMBRANE_TRANS	-0,028	0,444	0,90111	1
ELECTRON_CARRIER_ACTIVITY	-0,028	0,445	0,41696	1
MORF_PRDX3	-0,028	0,445	0,3001	1
GCM_BMPR2	-0,028	0,446	0,42616	1
PID_AR_TF_PATHWAY	0,028	0,446	0,51715	1

LOPEZ_MESOTHELIOMA_SURVIVAL_UP	-0,028	0,446	0,01702	1
GSE36392_EOSINOPHIL_VS_MAC_IL25_TREA	-0,028	0,446	0,06554	1
BIOCARTA_EIF_PATHWAY	-0,028	0,447	0,73714	1
KEGG_PENTOSE_AND_GLUCURONATE_INTERC	0,028	0,447	0,17612	1
NEGATIVE_REGULATION_OF_TRANSLATION	0,028	0,448	0,97845	1
BREDEMEYER_RAG_SIGNALING_VIA_ATM_NOT	-0,028	0,448	0,09353	1
GOUYER_TUMOR_INVASIVENESS	0,028	0,448	0,10983	1
FLECHNER_BIOPSY_KIDNEY_TRANSPLANT_RE.	-0,028	0,449	0,56739	1
LATE_ENDOSOME	-0,028	0,449	0,36987	1
TRANSFERASE_ACTIVITY_TRANSFERRING_ALK	0,028	0,45	0,78741	1
INTERLEUKIN_8_PRODUCTION	0,028	0,45	0,88995	1
GLYCOPROTEIN_BIOSYNTHETIC_PROCESS	-0,028	0,45	0,32404	1
WALLACE_PROSTATE_CANCER_UP	-0,028	0,45	0,20798	1
SILIGAN_TARGETS_OF_EWS_FLI1_FUSION_UP	-0,028	0,45	0,83205	1
SERINE_TYPE_ENDOPEPTIDASE_INHIBITOR_AC	-0,028	0,451	0,3499	1
BIOCARTA_STRESS_PATHWAY	-0,028	0,451	0,37859	1
REACTOME_GLYCEROPHOSPHOLIPID_BIOSYNTI	-0,028	0,452	0,00904	1
RODRIGUES_THYROID_CARCINOMA_UP	-0,028	0,452	0,05145	1
chr17q21	-0,028	0,452	0,0159	1
NIKOLSKY_BREAST_CANCER_7P22_AMPLICON	-0,028	0,453	0,28739	1
KORKOLA_SEMINOMA_DN	0,028	0,453	0,44372	1
SITE_OF_POLARIZED_GROWTH	-0,028	0,453	0,00236	1
BIOCARTA_PML_PATHWAY	-0,028	0,453	0,24557	1
RANKIN_ANGIOGENIC_TARGETS_OF_VHL_HIF2	0,028	0,453	0,73671	1
GSE9988_ANTI_TREM1_VS_ANTI_TREM1_AN	-0,027	0,454	0,58928	1
KEGG_HUNTINGTONS_DISEASE	-0,027	0,454	0,36489	1
REACTOME_LATENT_INFECTION_OF_HOMO_S	-0,027	0,454	0,74199	1
RPS14_DN.V1_UP	-0,027	0,455	0,38604	1
MORF_PDPK1	-0,027	0,455	0,02352	1
MYOSIN_COMPLEX	0,027	0,455	0,25445	1
STANHILL_HRAS_TRANSFROMATION_UP	-0,027	0,455	0,16677	1
PATTERN_RECOGNITION_RECEPTOR_ACTIVITY	0,027	0,456	0,24549	1
GSE2197_CPG_DNA_VS_UNTREATED_IN_DC_	-0,027	0,456	0,00497	1
V$EVI1_03	-0,027	0,457	0,01413	1
HYDROLASE_ACTIVITY_ACTING_ON_CARBON_	0,027	0,457	0,07483	1
GNF2_CDC27	-0,027	0,457	0,97725	1
ELVIDGE_HYPOXIA_BY_DMOG_DN	-0,027	0,458	0,09037	1
DAIRKEE_TERT_TARGETS_DN	-0,027	0,458	0,10633	1
NEGATIVE_REGULATION_OF_BINDING	-0,027	0,458	0,61058	1
AMINO_ACID_AND_DERIVATIVE_METABOLIC_	-0,027	0,459	0,05827	1
GRANDVAUX_IFN_RESPONSE_NOT_VIA_IRF3	-0,027	0,459	0,42627	1
MARTINELLI_IMMATURE_NEUTROPHIL_DN	0,027	0,46	0,53244	1

GSE14769_UNSTIM_VS_120MIN_LPS_BMDM_	-0,027	0,46	0,06293	1
MYLLYKANGAS_AMPLIFICATION_HOT_SPOT_2	0,027	0,46	0,30583	1
GSE17721_0.5H_VS_4H_LPS_BMDM_UP	-0,027	0,46	0,51558	1
GSE14769_UNSTIM_VS_60MIN_LPS_BMDM_U	-0,027	0,46	0,1097	1
GSE9988_ANTI_TREM1_AND_LPS_VS_VEHICL	-0,027	0,46	0,3408	1
VESICLE_MEDIATED_TRANSPORT	0,027	0,461	0,25783	1
YAMASHITA_SILENCED_BY_METHYLATION	-0,027	0,462	0,01356	1
GSE17721_0.5H_VS_4H_PAM3CSK4_BMDM_	-0,027	0,462	0,06064	1
HISTONE_DEACETYLASE_BINDING	0,027	0,462	0,80708	1
chr14q13	0,027	0,462	0,95414	1
KREPPEL_CD99_TARGETS_DN	-0,027	0,463	0,45463	1
REACTOME_SYNTHESIS_OF_BILE_ACIDS_AND	0,027	0,463	0,66331	1
GSE37416_12H_VS_48H_F_TULARENSIS_LV	-0,027	0,463	0,43466	1
SPHINGOLIPID_METABOLIC_PROCESS	0,027	0,464	0,90722	1
GSE3982_MAC_VS_EFF_MEMORY_CD4_TCEL	0,027	0,464	0,27723	1
MORF_CSNK1D	-0,027	0,465	0,3996	1
GSE9988_ANTI_TREM1_VS_LPS_MONOCYTE_	-0,027	0,465	0,35348	1
GSE10856_CTRL_VS_TNFRSF6B_IN_MACROP	-0,027	0,465	0,12155	1
CHIBA_RESPONSE_TO_TSA	0,027	0,466	0,9426	1
chr14q23	0,027	0,467	0,55749	1
GSE13485_CTRL_VS_DAY3_YF17D_VACCINE	-0,027	0,467	0,7308	1
CAR_HPX	-0,027	0,467	0,09431	1
GSE15930_STIM_VS_STIM_AND_TRICHOSTAT	-0,027	0,467	0,48519	1
DNA_FRAGMENTATION_DURING_APOPTOSIS	-0,027	0,467	0,72222	1
GNF2_HCK	0,027	0,467	0,42401	1
GNF2_CARD15	0,027	0,468	0,4794	1
REACTOME_KERATAN_SULFATE_DEGRADATIC	-0,027	0,468	0,03359	1
PID_ARF_3PATHWAY	-0,027	0,468	0,59168	1
GSE11924_TFH_VS_TH1_CD4_TCELL_UP	-0,027	0,469	0,01662	1
NEGATIVE_REGULATION_OF_PROTEIN_METAB	-0,027	0,469	0,24992	1
LYASE_ACTIVITY	0,027	0,47	0,92047	1
WEBER_METHYLATED_LCP_IN_FIBROBLAST_U	0,027	0,47	0,86298	1
AAGGGAT,MIR-188	-0,027	0,47	0,11735	1
GSE29618_PDC_VS_MDC_DN	-0,026	0,47	0,42996	1
chrxq13	-0,026	0,47	0,05237	1
NIKOLSKY_BREAST_CANCER_1Q21_AMPLICOI	0,026	0,47	0,63796	1
BIOCARTA_DREAM_PATHWAY	-0,026	0,471	0,60791	1
GSE24634_TEFF_VS_TCONV_DAY3_IN_CULT	0,026	0,471	0,4751	1
MODULE_410	-0,026	0,472	0,18493	1
ST_GRANULE_CELL_SURVIVAL_PATHWAY	0,026	0,472	0,65881	1
GSE27786_BCELL_VS_CD8_TCELL_UP	-0,026	0,472	0,2215	1
NAKAYAMA_SOFT_TISSUE_TUMORS_PCA2_DI	-0,026	0,473	0,00863	1

NEGATIVE_REGULATION_OF_CELLULAR_PROT	-0,026	0,473	0,30431	1
GSE22886_NAIVE_CD4_TCELL_VS_48H_ACT	-0,026	0,473	0,00106	1
ST_ADRENERGIC	0,026	0,473	0,88759	1
PEDERSEN_METASTASIS_BY_ERBB2_ISOFORM	-0,026	0,474	0,00048	1
REACTOME_NUCLEOTIDE_LIKE_PURINERGIC_R	-0,026	0,475	0,12763	1
KEGG_RIBOFLAVIN_METABOLISM	0,026	0,475	0,99699	1
PHOSPHOLIPID_BIOSYNTHETIC_PROCESS	-0,026	0,475	0,15119	1
REACTOME_TRANSPORT_TO_THE_GOLGI_ANI	-0,026	0,475	0,48264	1
ONDER_CDH1_TARGETS_3_UP	0,026	0,475	0,06592	1
GCM_CSNK1A1	-0,026	0,476	0,49547	1
GNF2_STAT6	-0,026	0,476	0,60238	1
MITOCHONDRIAL_INNER_MEMBRANE	-0,026	0,476	0,74504	1
GCM_IL6ST	0,026	0,477	0,69368	1
PROTON_TRANSPORTING_TWO_SECTOR_ATP,	-0,026	0,477	0,8674	1
AGGGCAG,MIR-18A	-0,026	0,477	0,0379	1
GSE22886_NAIVE_BCELL_VS_DC_DN	0,026	0,477	0,36724	1
RUTELLA_RESPONSE_TO_HGF_UP	-0,026	0,477	0,84875	1
REACTOME_SYNTHESIS_OF_PC	-0,026	0,478	0,00985	1
NICK_RESPONSE_TO_PROC_TREATMENT_DN	0,026	0,478	0,98821	1
VESICLE_LOCALIZATION	0,026	0,478	0,72567	1
REGULATION_OF_NEURON_APOPTOSIS	-0,026	0,478	0,17234	1
REACTOME_INSULIN_RECEPTOR_RECYCLING	-0,026	0,479	0,61674	1
REN_MIF_TARGETS_DN	-0,026	0,479	0,23808	1
MIZUKAMI_HYPOXIA_DN	-0,026	0,479	0,67158	1
REACTOME_ELEVATION_OF_CYTOSOLIC_CA2	-0,026	0,48	0,35193	1
ACTIN_FILAMENT	-0,026	0,48	0,35702	1
REGULATION_OF_RHO_GTPASE_ACTIVITY	-0,026	0,48	0,27045	1
SHIN_B_CELL_LYMPHOMA_CLUSTER_1	-0,026	0,48	0,77998	1
GSE1432_1H_VS_24H_IFNG_MICROGLIA_DN	-0,026	0,48	0,58513	1
RASHI_RESPONSE_TO_IONIZING_RADIATION_3	-0,026	0,48	0,00451	1
PID_LPA4_PATHWAY	-0,026	0,481	0,00188	1
REACTOME_GLOBAL_GENOMIC_NER_GG_NER	-0,026	0,481	0,23486	1
PIEPOLI_LGI1_TARGETS_DN	-0,026	0,482	0,20853	1
WENG_POR_TARGETS_LIVER_UP	0,026	0,482	0,85736	1
MAHAJAN_RESPONSE_TO_IL1A_DN	-0,026	0,483	0,34901	1
POST_GOLGI_VESICLE_MEDIATED_TRANSPOR	0,026	0,485	0,59483	1
CASTELLANO_HRAS_TARGETS_UP	0,026	0,485	0,31301	1
DUTTA_APOPTOSIS_VIA_NFKB	-0,026	0,485	0,59182	1
ZEMBUTSU_SENSITIVITY_TO_FLUOROURACIL	-0,026	0,485	0,65781	1
REACTOME_GLUCURONIDATION	0,026	0,487	0,1502	1
MUELLER_COMMON_TARGETS_OF_AML_FUSI(	-0,025	0,487	0,56892	1
MODULE_40	0,025	0,488	0,47319	1

DARWICHE_PAPILLOMA_RISK_HIGH_VS_LOW_	-0,025	0,488	0,6084	1
chr3q26	-0,025	0,488	0,46867	1
KUROZUMI_RESPONSE_TO_ONCOCYTIC_VIRUS	-0,025	0,489	0,57753	1
chrxp22	-0,025	0,489	0,98018	1
MORF_PSMC2	-0,025	0,489	0,30755	1
GSE17721_LPS_VS_CPG_24H_BMDM_UP	-0,025	0,489	0,30224	1
CHOI_ATL_ACUTE_STAGE	-0,025	0,489	0,54119	1
MEMBRANE_LIPID_METABOLIC_PROCESS	-0,025	0,489	0,15888	1
REACTOME_VIRAL_MESSENGER_RNA_SYNTHE	-0,025	0,49	0,83117	1
MODULE_23	-0,025	0,49	0,04038	1
CHNG_MULTIPLE_MYELOMA_HYPERPLOID_DN	0,025	0,49	0,05965	1
GUTIERREZ_WALDENSTROEMS_MACROGLOBUL	-0,025	0,491	0,08346	1
KEGG_ABC_TRANSPORTERS	-0,025	0,491	0,00945	1
REGULATION_OF_BODY_FLUID_LEVELS	-0,025	0,492	0,00859	1
V$HTF_01	-0,025	0,492	0,34532	1
MIKKELSEN_MCV6_LCP_WITH_H3K27ME3	-0,025	0,492	0,32884	1
WATANABE_ULCERATIVE_COLITIS_WITH_CAN	-0,025	0,492	0,53455	1
MODULE_360	-0,025	0,493	0,29054	1
ROSS_ACUTE_MYELOID_LEUKEMIA_CBF	-0,025	0,493	0,02681	1
GNF2_ICAM3	-0,025	0,493	0,33351	1
MODULE_430	-0,025	0,493	0,21507	1
REACTOME_CIRCADIAN_REPRESSION_OF_EXPI	-0,025	0,493	0,62236	1
GSE2706_2H_VS_8H_LPS_STIM_DC_UP	-0,025	0,493	0,00716	1
CAIRO_HEPATOBLASTOMA_DN	-0,025	0,495	0,01798	1
SU_SALIVARY_GLAND	-0,025	0,495	0,76078	1
RESPONSE_TO_HORMONE_STIMULUS	0,025	0,496	0,28036	1
MEMBRANE_FUSION	0,025	0,496	0,40011	1
LENAOUR_DENDRITIC_CELL_MATURATION_DN	-0,025	0,496	0,87813	1
CELL_PROJECTION_PART	-0,025	0,497	0,02641	1
chryp11	-0,025	0,497	0,8459	1
GSE17721_12H_VS_24H_LPS_BMDM_DN	-0,025	0,497	0,48654	1
MORF_ERCC2	-0,025	0,497	0,04303	1
CHANG_CORE_SERUM_RESPONSE_DN	-0,025	0,497	0,02038	1
HERNANDEZ_ABERRANT_MITOSIS_BY_DOCET/	-0,025	0,498	0,52742	1
REACTOME_ORGANIC_CATION_ANION_ZWITTI	0,025	0,498	0,76369	1
RAMJAUN_APOPTOSIS_BY_TGFB1_VIA_SMAD	-0,025	0,499	0,44926	1
NIKOLSKY_BREAST_CANCER_17P11_AMPLICO	-0,025	0,499	0,08145	1
RUAN_RESPONSE_TO_TROGLITAZONE_DN	-0,025	0,5	0,12091	1
REACTOME_RESPIRATORY_ELECTRON_TRANSI	0,025	0,501	0,34094	1
BIOCARTA_AGPCR_PATHWAY	-0,025	0,502	0,70788	1
REACTOME_RESPIRATORY_ELECTRON_TRANSI	0,025	0,502	0,3483	1
L_AMINO_ACID_TRANSMEMBRANE_TRANSPOI	-0,025	0,502	0,83341	1

HISTONE_ACETYLTRANSFERASE_ACTIVITY	0,025	0,502	0,33622	1
MODULE_130	0,025	0,503	0,97433	1
ENDONUCLEASE_ACTIVITY	-0,025	0,503	0,39264	1
MODULE_273	-0,025	0,503	0,79915	1
MORF_RAB5A	0,025	0,504	0,05437	1
COATES_MACROPHAGE_M1_VS_M2_UP	-0,025	0,504	0,15473	1
PETRETTO_LEFT_VENTRICLE_MASS_QTL_CIS_	-0,025	0,504	0,38711	1
GNF2_TNFSF10	0,024	0,505	0,5135	1
TRANSFERASE_ACTIVITY_TRANSFERRING_ACY	-0,024	0,505	0,1653	1
CELLULAR_PROTEIN_CATABOLIC_PROCESS	-0,024	0,505	0,73788	1
LOW_DENSITY_LIPOPROTEIN_BINDING	0,024	0,505	0,94326	1
GSE17721_LPS_VS_POLYIC_8H_BMDM_DN	-0,024	0,506	0,25717	1
chr14q31	0,024	0,506	0,47448	1
HASEGAWA_TUMORIGENESIS_BY_RET_C634R	-0,024	0,507	0,66559	1
GGTGTGT,MIR-329	-0,024	0,507	0,08382	1
chr4q24	-0,024	0,507	0,34256	1
GSE16522_MEMORY_VS_NAIVE_CD8_TCELL_	-0,024	0,507	0,15794	1
GUO_HEX_TARGETS_DN	-0,024	0,507	0,05713	1
BIOCARTA_P35ALZHEIMERS_PATHWAY	0,024	0,508	0,81799	1
CREIGHTON_AKT1_SIGNALING_VIA_MTOR_DN	0,024	0,508	0,48488	1
REACTOME_PHOSPHORYLATION_OF_CD3_ANI	-0,024	0,508	0,38189	1
GSE9006_TYPE_1_VS_TYPE_2_DIABETES_PE	-0,024	0,508	0,69301	1
GSE1432_6H_VS_24H_IFNG_MICROGLIA_DN	-0,024	0,509	0,51043	1
ACTIN_FILAMENT_BINDING	-0,024	0,509	0,235	1
YAO_TEMPORAL_RESPONSE_TO_PROGESTERC	-0,024	0,509	0,68064	1
PROTEIN_PHOSPHATASE_BINDING	0,024	0,509	0,27666	1
GSE22886_DAY1_VS_DAY7_MONOCYTE_IN_	-0,024	0,51	0,93541	1
RNA_ELONGATION	-0,024	0,51	0,87206	1
STEARMAN_TUMOR_FIELD_EFFECT_UP	0,024	0,51	0,564	1
chr4q28	-0,024	0,51	0,16584	1
PID_HIF1APATHWAY	-0,024	0,51	0,87729	1
GOLUB_ALL_VS_AML_DN	0,024	0,51	0,72481	1
chr5q22	0,024	0,511	0,35498	1
REACTOME_FORMATION_OF_ATP_BY_CHEMIC	-0,024	0,511	0,90821	1
KIM_LRRC3B_TARGETS	-0,024	0,512	0,83693	1
chr22q	-0,024	0,512	0,95255	1
MORF_HEAB	-0,024	0,513	0,00599	1
WANG_TNF_TARGETS	-0,024	0,513	0,32404	1
MOOTHA_TCA	-0,024	0,514	0,40392	1
NIELSEN_LIPOSARCOMA_UP	-0,024	0,514	0,09602	1
REACTOME_CYTOCHROME_P450_ARRANGED_	-0,024	0,515	0,03223	1
PID_P38GAMMADELTAPATHWAY	0,024	0,515	0,24508	1

GSE24634_IL4_VS_CTRL_TREATED_NAIVE_(	-0,024	0,515	0,55473	1
REACTOME_TRANSLOCATION_OF_ZAP_70_T(	-0,024	0,515	0,33621	1
GUTIERREZ_MULTIPLE_MYELOMA_DN	0,024	0,516	0,28586	1
chr10q25	0,024	0,517	0,1198	1
GSE29615_CTRL_VS_DAY3_LAIV_IFLU_VAC(	-0,024	0,517	0,09773	1
MODULE_323	-0,024	0,517	0,86945	1
GSE9988_LPS_VS_VEHICLE_TREATED_MONO	-0,024	0,517	0,30319	1
GSE12845_IGD_NEG_BLOOD_VS_DARKZONE_	-0,024	0,518	0,3897	1
MORF_RAC1	-0,024	0,518	0,24324	1
YAN_ESCAPE_FROM_ANOIKIS	-0,024	0,518	0,8385	1
REACTOME_NEUROTRANSMITTER_RELEASE_C	-0,024	0,519	0,03569	1
TSAI_DNAJB4_TARGETS_UP	0,024	0,52	0,88111	1
GSE11864_CSF1_IFNG_VS_CSF1_IFNG_PAM3	-0,024	0,52	0,24315	1
GARGALOVIC_RESPONSE_TO_OXIDIZED_PHOS	0,024	0,52	0,8382	1
BIDUS_METASTASIS_DN	0,024	0,52	0,37361	1
NAKAMURA_BRONCHIAL_AND_BRONCHIOLAR_	0,024	0,52	0,38492	1
GSE11864_CSF1_VS_CSF1_IFNG_PAM3CYS_	0,024	0,521	0,34217	1
KAMIKUBO_MYELOID_MN1_NETWORK	0,024	0,521	0,82656	1
KEGG_NOD_LIKE_RECEPTOR_SIGNALING_PATI	-0,024	0,522	0,9488	1
GLYCEROPHOSPHOLIPID_BIOSYNTHETIC_PROC	0,023	0,522	0,85252	1
GCM_NF2	-0,023	0,522	0,13513	1
MYLLYKANGAS_AMPLIFICATION_HOT_SPOT_1	0,023	0,522	0,82666	1
SMALL_PROTEIN_CONJUGATING_ENZYME_AC	-0,023	0,523	0,33994	1
REACTOME_ENDOSOMAL_SORTING_COMPLEX_	0,023	0,523	0,80557	1
MANNOSYLTRANSFERASE_ACTIVITY	0,023	0,524	0,50999	1
ODONNELL_TARGETS_OF_MYC_AND_TFRC_U	-0,023	0,524	0,12213	1
RIBONUCLEOTIDE_METABOLIC_PROCESS	0,023	0,525	0,18468	1
WUNDER_INFLAMMATORY_RESPONSE_AND_C	-0,023	0,525	0,66479	1
GOZGIT_ESR1_TARGETS_DN	-0,023	0,525	0,00021	1
MATZUK_STEROIDOGENESIS	-0,023	0,526	0,14721	1
DAUER_STAT3_TARGETS_DN	-0,023	0,526	0,58718	1
MORI_LARGE_PRE_BII_LYMPHOCYTE_DN	-0,023	0,526	0,39531	1
GUTIERREZ_WALDENSTROEMS_MACROGLOBUI	-0,023	0,526	0,03939	1
MAINA_VHL_TARGETS_DN	-0,023	0,526	0,5737	1
MODULE_311	-0,023	0,528	0,65116	1
BAELDE_DIABETIC_NEPHROPATHY_UP	-0,023	0,529	0,09207	1
VOLTAGE_GATED_CALCIUM_CHANNEL_COMP	0,023	0,529	0,07146	1
WEBER_METHYLATED_HCP_IN_SPERM_DN	-0,023	0,529	0,30816	1
REACTOME_PD1_SIGNALING	-0,023	0,529	0,43706	1
GARGALOVIC_RESPONSE_TO_OXIDIZED_PHOS	-0,023	0,53	0,01022	1
MODULE_103	0,023	0,53	0,61191	1
BYSTRYKH_SCP2_QTL	0,023	0,531	0,1017	1

OXIDOREDUCTASE_ACTIVITY	-0,023	0,531	0,41575	1
COENZYME_BINDING	-0,023	0,532	0,14119	1
KEGG_N_GLYCAN_BIOSYNTHESIS	-0,023	0,532	0,95669	1
KEGG_GRAFT_VERSUS_HOST_DISEASE	-0,023	0,532	0,76858	1
MOOTHA_PYR	0,023	0,532	0,26422	1
GSE17721_CTRL_VS_PAM3CSK4_12H_BMDM	-0,023	0,533	0,14039	1
ZHAN_VARIABLE_EARLY_DIFFERENTIATION_G	-0,023	0,533	0,31787	1
chr4q25	-0,023	0,534	0,09392	1
WIELAND_UP_BY_HBV_INFECTION	-0,023	0,534	0,76731	1
HOWLIN_CITED1_TARGETS_2_DN	-0,023	0,534	0,09178	1
AIYAR_COBRA1_TARGETS_DN	0,023	0,535	0,71053	1
GSE10325_LUPUS_CD4_TCELL_VS_LUPUS_M	0,023	0,535	0,41722	1
GNF2_MYD88	-0,023	0,536	0,65477	1
NUCLEOTIDE_SUGAR_METABOLIC_PROCESS	-0,023	0,536	0,71761	1
PELLICCIOTTA_HDAC_IN_ANTIGEN_PRESENTA	-0,023	0,536	0,37922	1
GCM_UBE2N	-0,023	0,536	0,28584	1
GSE36476_YOUNG_VS_OLD_DONOR_MEMOR	-0,023	0,537	0,03843	1
REACTOME_CASPASE_MEDIATED_CLEAVAGE_	-0,023	0,537	0,00219	1
DETECTION_OF_EXTERNAL_STIMULUS	-0,023	0,538	0,00594	1
REACTOME_TRAF6_MEDIATED_IRF7_ACTIVAT	-0,023	0,538	0,93801	1
REGULATION_OF_CELLULAR_COMPONENT_SI	-0,023	0,539	0,11255	1
REGULATION_OF_ACTIN_FILAMENT_LENGTH	-0,023	0,54	0,11601	1
GREENBAUM_E2A_TARGETS_DN	-0,022	0,541	0,25046	1
REACTOME_ADENYLATE_CYCLASE_ACTIVATI	-0,022	0,541	0,00179	1
ABBUD_LIF_SIGNALING_1_DN	-0,022	0,541	0,2232	1
MORF_MBD4	-0,022	0,542	0,30954	1
REACTOME_ETHANOL_OXIDATION	-0,022	0,543	0,02943	1
PROTEIN_MATURATION	0,022	0,543	0,93866	1
NIKOLSKY_BREAST_CANCER_19Q13.4_AMPLI	0,022	0,544	0,71111	1
REACTOME_ERK_MAPK_TARGETS	0,022	0,544	0,50269	1
MORF_RAD23B	-0,022	0,544	0,285	1
chr19p13	-0,022	0,544	0,0914	1
MODULE_293	0,022	0,545	0,43509	1
REACTOME_EICOSANOID_LIGAND_BINDING_RE	-0,022	0,545	0,05646	1
KASLER_HDAC7_TARGETS_2_DN	0,022	0,546	0,016	1
GSE22886_NAIVE_TCELL_VS_MONOCYTE_DN	0,022	0,547	0,44394	1
MODULE_350	-0,022	0,547	0,30898	1
KANG_CISPLATIN_RESISTANCE_UP	-0,022	0,548	0,39168	1
GLYCOPROTEIN_METABOLIC_PROCESS	-0,022	0,548	0,41001	1
CHIANG_LIVER_CANCER_SUBCLASS_UNANNO	-0,022	0,548	0,11543	1
GLYCOSPHINGOLIPID_METABOLIC_PROCESS	-0,022	0,548	0,21861	1
PROTEIN_TARGETING_TO_MEMBRANE	-0,022	0,549	0,24795	1

MODULE_141	-0,022	0,549	0,11682	1
GSE22886_NAIVE_TCELL_VS_DC_DN	0,022	0,55	0,48143	1
PURINE_NUCLEOTIDE_METABOLIC_PROCESS	0,022	0,55	0,43138	1
WEST_ADRENOCORTICAL_CARCINOMA_VS_AI	-0,022	0,55	0,15483	1
chr5p15	-0,022	0,552	0,05223	1
GSE22886_NAIVE_CD8_TCELL_VS_DC_DN	0,022	0,554	0,38585	1
MCGOWAN_RSP6_TARGETS_DN	0,022	0,555	0,10373	1
PID_AVB3_OPN_PATHWAY	-0,022	0,556	0,46216	1
REGULATION_OF_PROTEIN_POLYMERIZATION	0,022	0,556	0,2392	1
ACTIVATION_OF_NF_KAPPAB_TRANSCRIPTIOI	-0,022	0,557	0,29879	1
PROTEIN_TARGETING_TO_MITOCHONDRION	-0,022	0,557	0,568	1
CHIANG_LIVER_CANCER_SUBCLASS_INTERFEI	-0,022	0,557	0,49229	1
VANTVEER_BREAST_CANCER_BRCA1_DN	0,022	0,557	0,02682	1
WEBER_METHYLATED_HCP_IN_SPERM_UP	-0,022	0,557	0,20638	1
PYRIMIDINE_NUCLEOTIDE_METABOLIC_PROCES	0,022	0,557	0,68094	1
WEBER_METHYLATED_ICP_IN_SPERM_UP	0,021	0,558	0,13931	1
MODULE_470	-0,021	0,558	0,02237	1
CELL_CORTEX	0,021	0,559	0,82208	1
GSE2706_UNSTIM_VS_2H_R848_DC_UP	-0,021	0,559	0,00175	1
MODULE_432	0,021	0,559	0,26689	1
RAS_GUANYL_NUCLEOTIDE_EXCHANGE_FACT	-0,021	0,56	0,0256	1
DEACETYLASE_ACTIVITY	0,021	0,56	0,82854	1
GARGALOVIC_RESPONSE_TO_OXIDIZED_PHOS	-0,021	0,561	0,02192	1
REACTOME_HYALURONAN_UPTAKE_AND_DEC	-0,021	0,561	0,32786	1
KEGG_TOLL_LIKE_RECEPTOR_SIGNALING_PAT	-0,021	0,561	0,97648	1
GSE15767_MED_VS_SCS_MAC_LN_UP	-0,021	0,561	0,22217	1
KEGG_HISTIDINE_METABOLISM	0,021	0,561	0,96057	1
CARBOHYDRATE_TRANSMEMBRANE_TRANSPC	0,021	0,562	0,07027	1
chr1q44	-0,021	0,562	0,10897	1
chr14q24	-0,021	0,562	0,06428	1
RUTELLA_RESPONSE_TO_CSF2RB_AND_IL4_I	-0,021	0,563	0,51456	1
BOYAULT_LIVER_CANCER_SUBCLASS_G12_D	-0,021	0,563	0,05969	1
MODULE_59	0,021	0,563	0,31595	1
RESPONSE_TO_XENOBIOTIC_STIMULUS	0,021	0,563	0,65813	1
BONOME_OVARIAN_CANCER_POOR_SURVIVAI	-0,021	0,563	0,04807	1
GSE13485_DAY1_VS_DAY7_YF17D_VACCINI	-0,021	0,563	0,96658	1
REACTOME_ASPARAGINE_N_LINKED_GLYCOS'	-0,021	0,563	0,97648	1
GSE3982_MAC_VS_CENT_MEMORY_CD4_TCE	-0,021	0,564	0,83736	1
RESPONSE_TO_CARBOHYDRATE_STIMULUS	-0,021	0,564	0,48863	1
WANG_THOC1_TARGETS_UP	0,021	0,565	0,07856	1
STK33_NOMO_UP	-0,021	0,565	0,43407	1
COENZYME_METABOLIC_PROCESS	-0,021	0,565	0,52959	1

REACTOME_DOPAMINE_NEUROTRANSMITTER_	-0,021	0,565	0,02965	1
PID_IL1PATHWAY	-0,021	0,567	0,85997	1
ACEVEDO_LIVER_CANCER_DN	-0,021	0,567	0,02057	1
CHIANG_LIVER_CANCER_SUBCLASS_POLYSON	-0,021	0,567	0,06339	1
ZHANG_INTERFERON_RESPONSE	-0,021	0,568	0,86019	1
GSE10325_MYELOID_VS_LUPUS_MYELOID_DI	-0,021	0,569	0,85629	1
LI_ADIPOGENESIS_BY_ACTIVATED_PPARG	0,021	0,57	0,30549	1
ZIRN_TRETINOIN_RESPONSE_DN	-0,021	0,57	0,20435	1
ICHIBA_GRAFT_VERSUS_HOST_DISEASE_D7_I	-0,021	0,573	0,67182	1
REGULATION_OF_ENDOTHELIAL_CELL_PROLIF	0,021	0,573	0,65875	1
MORF_RAB6A	-0,021	0,573	0,3943	1
NIKOLSKY_BREAST_CANCER_8P12_P11_AMP	-0,021	0,574	0,40413	1
INOSITOL_OR_PHOSPHATIDYLINOSITOL_PHOS	-0,021	0,574	0,34063	1
GNF2_VAV1	-0,021	0,574	0,60492	1
MODULE_20	-0,021	0,575	0,38555	1
AMINE_TRANSPORT	0,021	0,575	0,52784	1
CATABOLIC_PROCESS	0,021	0,576	0,79579	1
POSITIVE_REGULATION_OF_TRANSCRIPTION_I	-0,021	0,576	0,27092	1
VALK_AML_WITH_EVI1	-0,021	0,576	0,00323	1
CHANGOLKAR_H2AFY_TARGETS_DN	-0,02	0,577	0,20661	1
HISTONE_DEACETYLASE_COMPLEX	0,02	0,578	0,58365	1
WAKABAYASHI_ADIPOGENESIS_PPARG_BOUNI	0,02	0,578	0,3793	1
KOBAYASHI_EGFR_SIGNALING_24HR_UP	0,02	0,579	0,63597	1
WANG_CLASSIC_ADIPOGENIC_TARGETS_OF_I	0,02	0,579	0,57923	1
MAGRANGEAS_MULTIPLE_MYELOMA_IGG_VS_	-0,02	0,579	0,01007	1
GSE22886_IGM_MEMORY_BCELL_VS_BM_PLA	0,02	0,58	0,78873	1
GARGALOVIC_RESPONSE_TO_OXIDIZED_PHOS	0,02	0,58	0,58138	1
POSITIVE_REGULATION_OF_I_KAPPAB_KINASI	-0,02	0,581	0,43345	1
GSE11057_PBMC_VS_MEM_CD4_TCELL_UP	-0,02	0,581	0,62868	1
GSE27786_LIN_NEG_VS_NKTCELL_DN	-0,02	0,581	0,02266	1
VILIMAS_NOTCH1_TARGETS_DN	-0,02	0,581	0,13808	1
NUCLEOTIDE_EXCISION_REPAIR	0,02	0,582	0,67527	1
ST_MYOCYTE_AD_PATHWAY	-0,02	0,582	0,08457	1
ESC_J1_UP_EARLY.V1_UP	-0,02	0,582	0,00169	1
XENOBIOTIC_METABOLIC_PROCESS	0,02	0,582	0,64646	1
MODULE_235	-0,02	0,583	0,98517	1
LUI_THYROID_CANCER_PAX8_PPARG_DN	-0,02	0,583	0,66558	1
TRANSCRIPTION_FROM_RNA_POLYMERASE_III_	-0,02	0,584	0,33772	1
REACTOME_SYNTHESIS_OF_PIPS_AT_THE_PL	-0,02	0,584	0,14211	1
DI___TRI_VALENT_INORGANIC_CATION_TRAN	0,02	0,584	0,64648	1
GSE17721_0.5H_VS_4H_CPG_BMDM_UP	0,02	0,585	0,79431	1
AROMATIC_COMPOUND_METABOLIC_PROCESS	0,02	0,585	0,66951	1

WANG_THOC1_TARGETS_DN	-0,02	0,585	0,55824	1
GSE22886_TH1_VS_TH2_48H_ACT_DN	-0,02	0,586	0,34199	1
KRISHNAN_FURIN_TARGETS_UP	-0,02	0,586	0,45789	1
BRUNEAU_SEPTATION_VENTRICULAR	-0,02	0,586	0,85887	1
KUMAMOTO_RESPONSE_TO_NUTLIN_3A_UP	0,02	0,587	0,05592	1
chr12q24	-0,02	0,587	0,29978	1
LYSOSOME	0,02	0,587	0,76306	1
LYTIC_VACUOLE	0,02	0,587	0,76306	1
chr6q13	-0,02	0,587	0,78792	1
REACTOME_SYNTHESIS_OF_PIPS_AT_THE_LA	-0,02	0,587	0,97118	1
MOTAMED_RESPONSE_TO_ANDROGEN_UP	0,02	0,587	0,32699	1
chr11q13	-0,02	0,588	0,39962	1
WELCH_GATA1_TARGETS	-0,02	0,588	0,28538	1
SMID_BREAST_CANCER_RELAPSE_IN_PLEURA	-0,02	0,589	0,00276	1
BIOCARTA_GSK3_PATHWAY	-0,02	0,591	0,70096	1
VALK_AML_CLUSTER_8	-0,02	0,591	0,32461	1
GNF2_CD14	0,02	0,592	0,60787	1
REACTOME_SYNTHESIS_SECRETION_AND_DE/	0,02	0,593	0,77685	1
REACTOME_PPARA_ACTIVATES_GENE_EXPRES	-0,02	0,594	0,86892	1
BIOCARTA_SODD_PATHWAY	-0,02	0,594	0,6874	1
KEGG_ARACHIDONIC_ACID_METABOLISM	0,02	0,594	0,40079	1
GSE22886_NAIVE_CD8_TCELL_VS_MONOCY	0,02	0,594	0,50914	1
GSE24634_TREG_VS_TCONV_POST_DAY3_IL	0,02	0,595	0,84092	1
REACTOME_PI_METABOLISM	-0,02	0,595	0,24418	1
FERRARI_RESPONSE_TO_FENRETINIDE_DN	0,019	0,595	0,00594	1
MODULE_401	0,019	0,595	0,7054	1
NIKOLSKY_BREAST_CANCER_12Q24_AMPLIC	0,019	0,596	0,70975	1
GSE20715_WT_VS_TLR4_KO_48H_OZONE_L	-0,019	0,596	0,21184	1
GSE3982_MAST_CELL_VS_TH2_UP	-0,019	0,597	0,16809	1
GSE9037_CTRL_VS_LPS_4H_STIM_IRAK4_K	-0,019	0,597	0,13245	1
chr8q24	-0,019	0,597	0,84258	1
DOUBLE_STRAND_BREAK_REPAIR	-0,019	0,597	0,60125	1
REACTOME_PYRUVATE_METABOLISM_AND_C	-0,019	0,598	0,24932	1
REACTOME_TCA_CYCLE_AND_RESPIRATORY_	0,019	0,598	0,27451	1
GSE29617_CTRL_VS_DAY3_TIV_FLU_VACCI	-0,019	0,598	0,5397	1
PKCA_DN.V1_UP	-0,019	0,599	0,04236	1
CAR_MYST2	0,019	0,599	0,5299	1
REACTOME_LYSOSOME_VESICLE_BIOGENESIS	-0,019	0,602	0,52171	1
CARBOXYLESTERASE_ACTIVITY	-0,019	0,602	0,10651	1
KEGG_DRUG_METABOLISM_CYTOCHROME_P4	0,019	0,603	0,92672	1
OUTER_MEMBRANE	-0,019	0,603	0,79374	1
THILLAINADESAN_ZNF217_TARGETS_DN	0,019	0,603	0,2472	1

REACTOME_NUCLEOTIDE_EXCISION_REPAIR	-0,019	0,603	0,60806	1
VERNOCHET_ADIPOGENESIS	0,019	0,604	0,59417	1
MODULE_465	-0,019	0,604	0,60448	1
RHO_GUANYL_NUCLEOTIDE_EXCHANGE_FACT	-0,019	0,606	0,02687	1
RESPONSE_TO_OXIDATIVE_STRESS	-0,019	0,606	0,83575	1
KAMIKUBO_MYELOID_CEBPA_NETWORK	0,019	0,606	0,27515	1
IIZUKA_LIVER_CANCER_PROGRESSION_G2_G3	-0,019	0,606	0,32664	1
GSE9988_ANTI_TREM1_VS_VEHICLE_TREATE	-0,019	0,607	0,31448	1
CARBOHYDRATE_TRANSPORT	-0,019	0,607	0,8616	1
GSE31082_DN_VS_DP_THYMOCYTE_UP	-0,019	0,607	0,83393	1
SECONDARY_METABOLIC_PROCESS	-0,019	0,607	0,67836	1
KEGG_OXIDATIVE_PHOSPHORYLATION	0,019	0,607	0,31226	1
REACTOME_SYNTHESIS_OF_PIPS_AT_THE_GC	-0,019	0,608	0,83186	1
SMID_BREAST_CANCER_RELAPSE_IN_LUNG_D	0,019	0,608	0,00569	1
GROWTH_CONE	-0,019	0,609	0,00352	1
VETTER_TARGETS_OF_PRKCA_AND_ETS1_DN	0,019	0,609	0,6505	1
KUROKAWA_LIVER_CANCER_EARLY_RECURRE	0,019	0,609	0,11747	1
BIOCARTA_CD40_PATHWAY	-0,019	0,609	0,09026	1
REACTOME_INNATE_IMMUNE_SYSTEM	-0,019	0,609	0,45122	1
GCGCCTT,MIR-525,MIR-524	0,019	0,61	0,856	1
BIOCARTA_D4GDI_PATHWAY	-0,019	0,61	0,78653	1
REACTOME_GLYCOLYSIS	0,019	0,61	0,12789	1
chr10q	-0,019	0,611	0,56638	1
ENDOPLASMIC_RETICULUM_PART	-0,019	0,611	0,74823	1
LIGASE_ACTIVITY_FORMING_CARBON_NITROG	-0,019	0,611	0,54847	1
LIN_MELANOMA_COPY_NUMBER_DN	-0,019	0,611	0,79027	1
KEGG_ASCORBATE_AND_ALDARATE_METABC	0,019	0,611	0,34106	1
REACTOME_REGULATION_OF_INSULIN_SECRET	-0,019	0,612	0,10036	1
BURTON_ADIPOGENESIS_5	-0,019	0,613	0,98355	1
SENSORY_ORGAN_DEVELOPMENT	-0,019	0,614	0,69081	1
POLYSACCHARIDE_METABOLIC_PROCESS	0,019	0,614	0,86856	1
REACTOME_HORMONE_SENSITIVE_LIPASE_HS	-0,019	0,614	0,05227	1
REACTOME_PLATELET_CALCIUM_HOMEOSTAS	-0,019	0,614	0,50298	1
NIKOLSKY_BREAST_CANCER_20Q11_AMPLICO	0,018	0,615	0,69553	1
MITOCHONDRIAL_MEMBRANE	-0,018	0,615	0,84531	1
XU_HGF_SIGNALING_NOT_VIA_AKT1_6HR	-0,018	0,615	0,87787	1
GSE13485_DAY1_VS_DAY3_YF17D_VACCINI	-0,018	0,615	0,94479	1
REGULATION_OF_GENE_SPECIFIC_TRANSCRIPT	-0,018	0,615	0,21604	1
REACTOME_MAP_KINASE_ACTIVATION_IN_TL	-0,018	0,615	0,68017	1
chr1p12	-0,018	0,616	0,96302	1
GCM_RBM8A	-0,018	0,617	0,36716	1
BIOCARTA_TOLL_PATHWAY	-0,018	0,617	0,64997	1

GNF2_CD1D	0,018	0,618	0,64293	1
TACGGGT,MIR-99A,MIR-100,MIR-99B	-0,018	0,618	0,13457	1
CELLULAR_MACROMOLECULE_CATABOLIC_PR	-0,018	0,619	0,61612	1
LUDWICZEK_TREATING_IRON_OVERLOAD	-0,018	0,62	0,55271	1
GSE31082_CD4_VS_CD8_SP_THYMOCYTE_U	-0,018	0,62	0,15046	1
BIOCARTA_NFKB_PATHWAY	0,018	0,621	0,62471	1
MATTIOLI_MULTIPLE_MYELOMA_SUBGROUPS	0,018	0,621	0,07422	1
REACTOME_FORMATION_OF_RNA_POL_II_ELC	-0,018	0,621	0,84942	1
OXIDOREDUCTASE_ACTIVITY_ACTING_ON_TH	-0,018	0,621	0,15929	1
REACTOME_N_GLYCAN_TRIMMING_IN_THE_EI	0,018	0,622	0,11992	1
KEGG_PHENYLALANINE_METABOLISM	-0,018	0,623	0,19176	1
VERHAAK_AML_WITH_NPM1_MUTATED_UP	-0,018	0,624	0,87667	1
LIPID_TRANSPORTER_ACTIVITY	-0,018	0,624	0,08047	1
ANTIGEN_BINDING	-0,018	0,624	0,77858	1
MATZUK_FERTILIZATION	-0,018	0,625	0,80041	1
RODRIGUES_THYROID_CARCINOMA_ANAPLAST	-0,018	0,625	0,00435	1
NUCLEOBASENUCLEOSIDENUCLEOTIDE_AND_I	0,018	0,625	0,23442	1
GSE11864_UNTREATED_VS_CSF1_IFNG_IN_N	-0,018	0,626	0,00473	1
GSE11864_CSF1_VS_CSF1_IFNG_IN_MAC_UI	-0,018	0,626	0,02657	1
CIRCADIAN_RHYTHM	0,018	0,626	0,15784	1
BIOCARTA_CLASSIC_PATHWAY	0,018	0,626	0,46453	1
KEGG_STEROID_HORMONE_BIOSYNTHESIS	-0,018	0,627	0,8639	1
BRUNEAU_HEART_GREAT_VESSELS_AND_VAI	-0,018	0,627	0,3616	1
KEGG_ALPHA_LINOLENIC_ACID_METABOLISM	-0,018	0,629	0,26813	1
MYLLYKANGAS_AMPLIFICATION_HOT_SPOT_3	-0,018	0,629	0,16337	1
REACTOME_EXTRINSIC_PATHWAY_FOR_APOP	0,018	0,63	0,47845	1
HISTONE_MODIFICATION	-0,018	0,63	0,1088	1
LI_WILMS_TUMOR_VS_FETAL_KIDNEY_1_UP	-0,018	0,633	0,09048	1
BIOCARTA_41BB_PATHWAY	-0,018	0,633	0,69226	1
TONKS_TARGETS_OF_RUNX1_RUNX1T1_FUSI	-0,018	0,633	0,07022	1
PID_WNT_CANONICAL_PATHWAY	-0,018	0,633	0,3987	1
REACTOME_FORMATION_OF_THE_HIV1_EARL	-0,017	0,634	0,71753	1
MITOCHONDRIAL_ENVELOPE	-0,017	0,636	0,81109	1
GCNP_SHH_UP_EARLY.V1_DN	-0,017	0,636	0,05854	1
GNF2_PECAM1	0,017	0,637	0,79045	1
REACTOME_ACTIVATION_OF_IRF3_IRF7_MEDI	-0,017	0,637	0,43963	1
BIOCARTA_BLYMPHOCYTE_PATHWAY	-0,017	0,637	0,86186	1
REACTOME_IL_6_SIGNALING	-0,017	0,637	0,41066	1
BIOCARTA_CERAMIDE_PATHWAY	0,017	0,638	0,56543	1
SUMI_HNF4A_TARGETS	0,017	0,638	0,8847	1
WANG_RECURRENT_LIVER_CANCER_DN	-0,017	0,638	0,38443	1
BIOCARTA_MONOCYTE_PATHWAY	-0,017	0,639	0,40403	1

SPIELMAN_LYMPHOBLAST_EUROPEAN_VS_AS	-0,017	0,639	0,52937	1
TAKADA_GASTRIC_CANCER_COPY_NUMBER_I	-0,017	0,641	0,74735	1
PID_TOLL_ENDOGENOUS_PATHWAY	-0,017	0,641	0,45171	1
MORI_IMMATURE_B_LYMPHOCYTE_UP	-0,017	0,642	0,77798	1
MANTOVANI_NFKB_TARGETS_DN	0,017	0,644	0,15332	1
REACTOME_SYNTHESIS_SECRETION_AND_INA	0,017	0,644	0,98349	1
EMBRYO_IMPLANTATION	-0,017	0,645	0,73724	1
MYLLYKANGAS_AMPLIFICATION_HOT_SPOT_2	-0,017	0,645	0,54321	1
TRANSPORT_VESICLE	0,017	0,645	0,41069	1
GNF2_CD48	-0,017	0,646	0,65319	1
REGULATION_OF_JNK_CASCADE	-0,017	0,646	0,04203	1
chr3p11	0,017	0,647	0,53266	1
RIBOSOMAL_SUBUNIT	-0,017	0,647	0,75174	1
HYDRO_LYASE_ACTIVITY	-0,017	0,648	0,56036	1
CARBON_OXYGEN_LYASE_ACTIVITY	-0,017	0,648	0,58019	1
YAP1_DN	0,017	0,648	0,08128	1
NUCLEAR_ENVELOPE_ENDOPLASMIC_RETICUL	-0,017	0,648	0,76974	1
ZIRN_TRETINOIN_RESPONSE_WT1_DN	-0,017	0,649	0,0561	1
REACTOME_DSCAM_INTERACTIONS	0,017	0,65	0,85549	1
ZHAN_MULTIPLE_MYELOMA_CD1_DN	-0,017	0,65	0,10169	1
chr17q11	0,017	0,651	0,83936	1
PARK_TRETINOIN_RESPONSE_AND_RARA_PLZ	0,017	0,651	0,45562	1
ONDER_CDH1_TARGETS_1_UP	-0,017	0,652	0,02157	1
BHAT_ESR1_TARGETS_NOT_VIA_AKT1_DN	0,017	0,652	0,30519	1
WIKMAN_ASBESTOS_LUNG_CANCER_DN	-0,017	0,653	0,41738	1
chr4p13	-0,016	0,653	0,12886	1
REACTOME_INTERFERON_ALPHA_BETA_SIGNA	-0,016	0,653	0,74839	1
REACTOME_TETRAHYDROBIOPTERIN_BH4_SYN	0,016	0,653	0,25724	1
MORF_PPP2R5E	-0,016	0,654	0,38282	1
FULCHER_INFLAMMATORY_RESPONSE_LECTIN	-0,016	0,654	0,5597	1
GSE27786_BCELL_VS_NKCELL_DN	-0,016	0,654	0,03926	1
KEGG_NUCLEOTIDE_EXCISION_REPAIR	-0,016	0,655	0,6992	1
REGULATION_OF_MAPKKK_CASCADE	0,016	0,655	0,20076	1
KEGG_LEISHMANIA_INFECTION	-0,016	0,656	0,78751	1
PROTEIN_AMINO_ACID_AUTOPHOSPHORYLATI	0,016	0,656	0,76906	1
NIKOLSKY_BREAST_CANCER_17Q11_Q21_AM	-0,016	0,657	0,43747	1
GNF2_CD97	-0,016	0,657	0,62925	1
WILSON_PROTEASES_AT_TUMOR_BONE_INTE	-0,016	0,657	0,96308	1
REACTOME_SYNTHESIS_OF_BILE_ACIDS_AND	0,016	0,658	0,77576	1
REACTOME_TRAFFICKING_AND_PROCESSING_	0,016	0,658	0,47287	1
FUJIWARA_PARK2_HEPATOCYTE_PROLIFERAT	-0,016	0,658	0,08298	1
RICKMAN_TUMOR_DIFFERENTIATED_MODERAT	-0,016	0,658	0,2272	1

BHAT_ESR1_TARGETS_VIA_AKT1_DN	0,016	0,659	0,54514	1
BIOCARTA_ASBCELL_PATHWAY	-0,016	0,659	0,64405	1
SABATES_COLORECTAL_ADENOMA_SIZE_UP	-0,016	0,66	0,0194	1
BIOCARTA_ETC_PATHWAY	0,016	0,66	0,10293	1
KASLER_HDAC7_TARGETS_1_DN	0,016	0,66	0,60565	1
GSE24102_GRANULOCYSTIC_MDSC_VS_NEUT	-0,016	0,66	0,00555	1
KRISHNAN_FURIN_TARGETS_DN	0,016	0,661	0,16931	1
GNF2_CD33	0,016	0,661	0,77225	1
SANA_RESPONSE_TO_IFNG_UP	-0,016	0,662	0,81941	1
BUDHU_LIVER_CANCER_METASTASIS_UP	-0,016	0,663	0,92179	1
FRASOR_TAMOXIFEN_RESPONSE_UP	0,016	0,664	0,44661	1
CTACTAG,MIR-325	-0,016	0,664	0,98826	1
GCM_RAN	-0,016	0,664	0,4377	1
UNFOLDED_PROTEIN_BINDING	-0,016	0,664	0,93501	1
HOLLEMAN_PREDNISOLONE_RESISTANCE_B_A	-0,016	0,665	0,15761	1
GSE22886_NAIVE_CD4_TCELL_VS_MONOCYT	0,016	0,666	0,49369	1
PHAGOCYTOSIS	-0,016	0,666	0,09453	1
GSE13485_CTRL_VS_DAY1_YF17D_VACCINE	0,016	0,666	0,37869	1
BIOCARTA_NTHI_PATHWAY	-0,016	0,667	0,85746	1
INTERACTION_WITH_HOST	0,016	0,667	0,43096	1
NEGATIVE_REGULATION_OF_DNA_BINDING	-0,016	0,667	0,69649	1
GSE6269_HEALTHY_VS_FLU_INF_PBMC_DN	-0,016	0,667	0,97589	1
KEGG_TRYPTOPHAN_METABOLISM	-0,016	0,668	0,91512	1
INTERCELLULAR_JUNCTION_ASSEMBLY	0,016	0,669	0,48451	1
RACTNNRTTTNC_UNKNOWN	-0,016	0,669	0,19987	1
REACTOME_AMINE_COMPOUND_SLC_TRANSP	-0,016	0,669	0,9258	1
FLECHNER_BIOPSY_KIDNEY_TRANSPLANT_RE	-0,016	0,67	0,2237	1
KEGG_GLYCOLYSIS_GLUCONEOGENESIS	-0,016	0,67	0,75555	1
GSE13485_PRE_VS_POST_YF17D_VACCINAT	-0,016	0,67	0,90057	1
DETECTION_OF_BIOTIC_STIMULUS	-0,016	0,67	0,19062	1
ANTIPORTER_ACTIVITY	0,016	0,67	0,15174	1
GNF2_SELL	-0,016	0,671	0,55597	1
chrxq	0,016	0,671	0,64682	1
GNF2_PTPN6	-0,016	0,672	0,59413	1
REACTOME_GROWTH_HORMONE_RECEPTOR_S	-0,015	0,673	0,02423	1
BIOCARTA_EIF2_PATHWAY	0,015	0,673	0,05889	1
MODULE_25	0,015	0,674	0,49081	1
CAGNYGKNAAA_UNKNOWN	-0,015	0,674	0,00023	1
BIOCARTA_PTEN_PATHWAY	0,015	0,674	0,94014	1
MODULE_212	-0,015	0,675	0,57711	1
REACTOME_REGULATION_OF_BETA_CELL_DE	-0,015	0,676	0,23461	1
REACTOME_GLUTAMATE_NEUROTRANSMITTEI	-0,015	0,676	0,01925	1

REACTOME_ACTIVATION_OF_THE_AP1_FAMIL	0,015	0,677	0,64352	1
MORF_RAB11A	-0,015	0,677	0,48414	1
RECEPTOR_MEDIATED_ENDOCYTOSIS	-0,015	0,677	0,09105	1
KEGG_CITRATE_CYCLE_TCA_CYCLE	0,015	0,677	0,11417	1
WU_HBX_TARGETS_3_DN	-0,015	0,678	0,29928	1
TRANS_GOLGI_NETWORK_TRANSPORT_VESIC	0,015	0,679	0,9171	1
BAUS_TFF2_TARGETS_DN	-0,015	0,679	0,8824	1
MORF_PPP2R4	-0,015	0,679	0,83202	1
REACTOME_BILE_SALT_AND_ORGANIC_ANIOI	-0,015	0,68	0,69545	1
MIKKELSEN_ES_LCP_WITH_H3K27ME3	-0,015	0,683	0,84365	1
BIOCARTA_EPONFKB_PATHWAY	0,015	0,684	0,82747	1
MODULE_415	-0,015	0,684	0,66936	1
ER_NUCLEAR_SIGNALING_PATHWAY	-0,015	0,685	0,55001	1
VALK_AML_CLUSTER_7	0,015	0,686	0,42514	1
REGULATION_OF_ENDOCYTOSIS	0,015	0,687	0,06891	1
REACTOME_NUCLEAR_RECEPTOR_TRANSCRIPT	0,015	0,687	0,00761	1
REACTOME_DCC_MEDIATED_ATTRACTIVE_SIG	-0,015	0,687	0,51498	1
MODULE_82	-0,015	0,689	0,627	1
chr6p23	-0,015	0,689	0,3545	1
SECRETORY_GRANULE	-0,015	0,689	0,24872	1
KEGG_ALLOGRAFT_REJECTION	-0,015	0,691	0,82708	1
GNF2_BNIP3L	-0,015	0,691	0,54366	1
ZHAN_MULTIPLE_MYELOMA_LB_UP	-0,015	0,692	0,00491	1
GAVIN_PDE3B_TARGETS	-0,015	0,692	0,60213	1
MAP_KINASE_KINASE_KINASE_ACTIVITY	-0,015	0,692	0,94473	1
chr10q26	0,015	0,693	0,26751	1
VERRECCHIA_RESPONSE_TO_TGFB1_C3	-0,014	0,693	0,24028	1
SUZUKI_CTCFL_TARGETS_UP	-0,014	0,693	0,00185	1
TRANSITION_METAL_ION_TRANSMEMBRANE_	0,014	0,694	0,87563	1
PIGMENT_BIOSYNTHETIC_PROCESS	-0,014	0,694	0,47864	1
CROMER_TUMORIGENESIS_DN	-0,014	0,694	0,90668	1
REACTOME_NEF_MEDIATED_DOWNREGULATIC	-0,014	0,694	0,57586	1
KEGG_GLYCOSPHINGOLIPID_BIOSYNTHESIS_G/	-0,014	0,695	0,86491	1
MORF_ATOX1	-0,014	0,695	0,34032	1
GSE17721_0.5H_VS_8H_CPG_BMDM_UP	-0,014	0,696	0,64725	1
CSR_EARLY_UP.V1_DN	-0,014	0,696	0,0015	1
GSE29618_BCELL_VS_MONOCYTE_DAY7_FLU	0,014	0,698	0,71393	1
GSE360_L_DONOVANI_VS_B_MALAYI_HIGH_	-0,014	0,698	0,41531	1
IVANOVA_HEMATOPOIESIS_MATURE_CELL	-0,014	0,698	0,71545	1
REACTOME_INTERFERON_GAMMA_SIGNALING	-0,014	0,698	0,88108	1
BIOCARTA_TNFR2_PATHWAY	-0,014	0,698	0,24014	1
GCM_BECN1	0,014	0,698	0,43368	1

GSE37416_0H_VS_3H_F_TULARENSIS_LVS_	-0,014	0,698	0,24648	1
GSE16522_ANTI_CD3CD28_STIM_VS_UNSTIM	-0,014	0,699	0,26414	1
SA_PROGRAMMED_CELL_DEATH	-0,014	0,699	0,02499	1
GSE11057_CD4_EFF_MEM_VS_PBMC_DN	-0,014	0,7	0,88523	1
COLDREN_GEFITINIB_RESISTANCE_DN	-0,014	0,701	0,32548	1
LUI_THYROID_CANCER_CLUSTER_4	0,014	0,701	0,78854	1
MOOTHA_GLYCOGEN_METABOLISM	0,014	0,701	0,51152	1
GSE360_L_DONOVANI_VS_T_GONDII_MAC_U	-0,014	0,702	0,25528	1
KEGG_PANTOTHENATE_AND_COA_BIOSYNTH	0,014	0,702	0,82333	1
HOFFMAN_CLOCK_TARGETS_UP	-0,014	0,702	0,0197	1
RESPONSE_TO_TOXIN	0,014	0,703	0,19847	1
REACTOME_ION_TRANSPORT_BY_P_TYPE_AT	-0,014	0,703	0,38729	1
GNF2_S100A4	0,014	0,703	0,82536	1
MAGRANGEAS_MULTIPLE_MYELOMA_IGG_VS_	0,014	0,704	0,26035	1
SIG_CD40PATHWAYMAP	0,014	0,704	0,33842	1
MORF_GPX4	-0,014	0,704	0,68473	1
BIOCARTA_NEUROTRANSMITTERS_PATHWAY	0,014	0,704	0,37703	1
GSE17721_CTRL_VS_POLYIC_6H_BMDM_UP	-0,014	0,705	0,49514	1
ST_WNT_CA2_CYCLIC_GMP_PATHWAY	-0,014	0,707	0,09479	1
MODULE_93	0,014	0,708	0,69594	1
ESTABLISHMENT_AND_OR_MAINTENANCE_OF	-0,014	0,709	0,48515	1
CHIANG_LIVER_CANCER_SUBCLASS_PROLIFER	0,014	0,709	0,05488	1
GNF2_ITGB2	-0,014	0,711	0,61095	1
BYSTROEM_CORRELATED_WITH_IL5_UP	-0,014	0,711	0,88424	1
GARGALOVIC_RESPONSE_TO_OXIDIZED_PHOS	0,014	0,712	0,79175	1
KEGG_TYROSINE_METABOLISM	-0,014	0,712	0,06934	1
ZHANG_RESPONSE_TO_CANTHARIDIN_UP	-0,013	0,714	0,83191	1
NIKOLSKY_BREAST_CANCER_19P13_AMPLICO	0,013	0,715	0,24424	1
TCCGTCC,MIR-184	0,013	0,716	0,52098	1
ABRAHAM_ALPC_VS_MULTIPLE_MYELOMA_UI	-0,013	0,717	0,0865	1
IIZUKA_LIVER_CANCER_PROGRESSION_L0_L1_	0,013	0,717	0,5022	1
FERREIRA_EWINGS_SARCOMA_UNSTABLE_VS_	0,013	0,717	0,0816	1
PID_ALK2PATHWAY	0,013	0,718	0,82832	1
CHUNG_BLISTER_CYTOTOXICITY_DN	-0,013	0,718	0,33572	1
MATZUK_OVULATION	-0,013	0,718	0,09479	1
MITOCHONDRIAL_PART	-0,013	0,718	0,85364	1
ST_INTERFERON_GAMMA_PATHWAY	-0,013	0,719	0,56657	1
CHANDRAN_METASTASIS_TOP50_UP	0,013	0,719	0,78603	1
POSITIVE_REGULATION_OF_SECRETION	0,013	0,719	0,61298	1
PARK_TRETINOIN_RESPONSE	-0,013	0,72	0,9172	1
GENERATION_OF_PRECURSOR_METABOLITES_	-0,013	0,72	0,13287	1
RODRIGUES_THYROID_CARCINOMA_POORLY_D	-0,013	0,72	0,03847	1

TESAR_ALK_TARGETS_HUMAN_ES_5D_UP	0,013	0,72	0,02804	1
JIANG_CORE_DUPLICON_GENES	0,013	0,722	0,42224	1
WILENSKY_RESPONSE_TO_DARAPLADIB	-0,013	0,723	0,96828	1
MORI_EMU_MYC_LYMPHOMA_BY_ONSET_TIMI	-0,013	0,723	0,4099	1
KEGG_TYPE_II_DIABETES_MELLITUS	-0,013	0,725	0,0043	1
GSE11057_NAIVE_CD4_VS_PBMC_CD4_TCEL	-0,013	0,725	0,93211	1
MODULE_431	-0,013	0,725	0,22628	1
MYLLYKANGAS_AMPLIFICATION_HOT_SPOT_1	0,013	0,726	0,85416	1
AMUNDSON_DNA_DAMAGE_RESPONSE_TP53	-0,013	0,726	0,12375	1
ATPASE_ACTIVITY_COUPLED_TO_TRANSMEM	0,013	0,726	0,30499	1
NAKAMURA_ALVEOLAR_EPITHELIUM	0,013	0,726	0,16617	1
REACTOME_P2Y_RECEPTORS	0,013	0,726	0,67972	1
REACTOME_BIOLOGICAL_OXIDATIONS	-0,013	0,726	0,17999	1
chr12q22	-0,013	0,727	0,19582	1
GERHOLD_RESPONSE_TO_TZD_UP	0,013	0,728	0,1924	1
NADLER_OBESITY_DN	0,013	0,729	0,72278	1
ENERGY_RESERVE_METABOLIC_PROCESS	-0,013	0,729	0,36992	1
JUBAN_TARGETS_OF_SPI1_AND_FLI1_UP	0,013	0,729	0,40636	1
PID_FASPATHWAY	-0,013	0,729	0,66702	1
REACTOME_ACYL_CHAIN_REMODELLING_OF_	-0,013	0,729	0,09309	1
OUYANG_PROSTATE_CANCER_PROGRESSION_	-0,013	0,73	0,2503	1
KEGG_AUTOIMMUNE_THYROID_DISEASE	-0,013	0,73	0,72192	1
REACTOME_REGULATION_OF_PYRUVATE_DEH	0,013	0,731	0,32369	1
GSE14308_NAIVE_CD4_TCELL_VS_INDUCED_	-0,013	0,732	0,01135	1
SMID_BREAST_CANCER_RELAPSE_IN_LIVER_D	0,013	0,732	0,00071	1
KEGG_CYTOSOLIC_DNA_SENSING_PATHWAY	-0,012	0,733	0,80743	1
REACTOME_TRANSPORT_OF_GLUCOSE_AND_	0,012	0,734	0,49622	1
KEGG_COMPLEMENT_AND_COAGULATION_CA	-0,012	0,734	0,12276	1
GOLGI_ASSOCIATED_VESICLE	-0,012	0,735	0,77413	1
TERAMOTO_OPN_TARGETS_CLUSTER_1	0,012	0,735	0,58862	1
YAO_TEMPORAL_RESPONSE_TO_PROGESTERO	-0,012	0,735	0,66274	1
VACUOLE	-0,012	0,736	0,27338	1
MARSON_FOXP3_TARGETS_STIMULATED_DN	-0,012	0,737	0,1035	1
chr6q25	-0,012	0,737	0,46311	1
GCM_CHUK	-0,012	0,737	0,85495	1
BIOCARTA_LECTIN_PATHWAY	0,012	0,738	0,11186	1
MAGRANGEAS_MULTIPLE_MYELOMA_IGLL_VS	-0,012	0,738	0,02998	1
BURTON_ADIPOGENESIS_10	-0,012	0,738	0,39353	1
BARRIER_COLON_CANCER_RECURRENCE_DN	0,012	0,738	0,58891	1
BASAKI_YBX1_TARGETS_DN	-0,012	0,739	0,18304	1
TURASHVILI_BREAST_CARCINOMA_DUCTAL_V	0,012	0,739	0,69658	1
GSE3982_DC_VS_EFF_MEMORY_CD4_TCELL_	0,012	0,739	0,83576	1

SHANK_TAL1_TARGETS_DN	-0,012	0,739	0,095	1
MORF_PTPN9	-0,012	0,74	0,18521	1
HISTONE_METHYLTRANSFERASE_ACTIVITY	-0,012	0,74	0,50238	1
I_KAPPAB_KINASE_NF_KAPPAB_CASCADE	-0,012	0,74	0,50141	1
SH3_DOMAIN_BINDING	-0,012	0,741	0,00476	1
INAMURA_LUNG_CANCER_SCC_SUBTYPES_DN	-0,012	0,743	0,8532	1
LIAN_LIPA_TARGETS_3M	-0,012	0,744	0,89912	1
GSE13306_LAMINA_PROPRIA_VS_SPLEEN_TF	-0,012	0,745	0,16257	1
GSE22886_DAY0_VS_DAY7_MONOCYTE_IN_	-0,012	0,746	0,44257	1
STEROID_METABOLIC_PROCESS	-0,012	0,746	0,44552	1
BOYAULT_LIVER_CANCER_SUBCLASS_G123_	-0,012	0,746	0,23045	1
chr8p21	-0,012	0,746	0,21026	1
STEGER_ADIPOGENESIS_UP	0,012	0,746	0,41192	1
KIM_BIPOLAR_DISORDER_OLIGODENDROCYTE_	-0,012	0,747	0,58927	1
CREIGHTON_ENDOCRINE_THERAPY_RESISTAN(	0,012	0,747	0,77219	1
GOLGI_APPARATUS	-0,012	0,748	0,48494	1
GNF2_MCL1	0,012	0,749	0,99646	1
PID_SMAD2_3PATHWAY	-0,012	0,75	0,2545	1
chr21q21	-0,012	0,75	0,38861	1
AMINO_ACID_TRANSPORT	0,012	0,751	0,47318	1
NAKAMURA_LUNG_CANCER_DIFFERENTIATION	0,012	0,752	0,80651	1
MODULE_135	-0,012	0,752	0,14433	1
VANLOO_SP3_TARGETS_DN	0,012	0,753	0,90656	1
GSE3982_NEUTROPHIL_VS_NKCELL_UP	-0,011	0,754	0,97526	1
chr7q11	-0,011	0,756	0,30444	1
MIZUSHIMA_AUTOPHAGOSOME_FORMATION	0,011	0,756	0,95842	1
JU_AGING_TERC_TARGETS_DN	-0,011	0,757	0,40055	1
REACTOME_ACYL_CHAIN_REMODELLING_OF_	0,011	0,757	0,44069	1
MIKHAYLOVA_OXIDATIVE_STRESS_RESPONSE	-0,011	0,757	0,81974	1
MIKI_COEXPRESSED_WITH_CYP19A1	0,011	0,758	0,70866	1
ST_JNK_MAPK_PATHWAY	-0,011	0,758	0,19902	1
BROWNE_INTERFERON_RESPONSIVE_GENES	-0,011	0,758	0,99694	1
MODULE_368	-0,011	0,758	0,70418	1
GCM_SIRT2	-0,011	0,758	0,20888	1
GSE29618_MONOCYTE_VS_PDC_UP	-0,011	0,759	0,80786	1
KEGG_CIRCADIAN_RHYTHM_MAMMAL	0,011	0,759	0,13884	1
chr14q32	0,011	0,759	0,2454	1
REACTOME_MRNA_CAPPING	-0,011	0,76	0,92093	1
VERRECCHIA_RESPONSE_TO_TGFB1_C6	-0,011	0,761	0,40765	1
KYNG_ENVIRONMENTAL_STRESS_RESPONSE_	0,011	0,761	0,67753	1
BIOCARTA_CFTR_PATHWAY	-0,011	0,762	0,17321	1
chr6q21	-0,011	0,763	0,39198	1

CELLULAR_POLYSACCHARIDE_METABOLIC_PR	0,011	0,763	0,7866	1
BIOCARTA_TID_PATHWAY	0,011	0,763	0,64125	1
MODULE_106	-0,011	0,764	0,09912	1
LOPEZ_MESOTELIOMA_SURVIVAL_TIME_DN	0,011	0,766	0,88929	1
REACTOME_THE_NLRP3_INFLAMMASOME	0,011	0,766	0,75561	1
REACTOME_JNK_C_JUN_KINASES_PHOSPHOR	0,011	0,766	0,46674	1
PIONTEK_PKD1_TARGETS_UP	-0,011	0,767	0,15969	1
chr22q12	-0,011	0,767	0,45595	1
GSE31082_DN_VS_DP_THYMOCYTE_DN	-0,011	0,768	0,28441	1
KEGG_RIG_I_LIKE_RECEPTOR_SIGNALING_PAT	-0,011	0,769	0,9973	1
SWEET_KRAS_ONCOGENIC_SIGNATURE	-0,011	0,77	0,86776	1
KIM_GLIS2_TARGETS_DN	0,011	0,77	0,72497	1
NAKAYAMA_SOFT_TISSUE_TUMORS_PCA1_UI	-0,011	0,77	0,75412	1
DING_LUNG_CANCER_EXPRESSION_BY_COPY_	-0,011	0,772	0,89659	1
MYLLYKANGAS_AMPLIFICATION_HOT_SPOT_S	-0,011	0,772	0,36157	1
REACTOME_CREATION_OF_C4_AND_C2_ACTI	0,011	0,773	0,94017	1
LIU_TOPBP1_TARGETS	-0,011	0,773	0,07644	1
ALDO_KETO_REDUCTASE_ACTIVITY	0,011	0,773	0,88083	1
WEBER_METHYLATED_LCP_IN_SPERM_UP	0,011	0,773	0,64744	1
KEGG_PROTEIN_EXPORT	-0,011	0,774	0,50668	1
WILLIAMS_ESR1_TARGETS_DN	-0,01	0,776	0,16667	1
TONKS_TARGETS_OF_RUNX1_RUNX1T1_FUSI	-0,01	0,777	0,15976	1
GSE11864_UNTREATED_VS_CSF1_IN_MAC_D	0,01	0,777	0,51004	1
ZHOU_PANCREATIC_EXOCRINE_PROGENITOR	-0,01	0,778	0,57387	1
GAZDA_DIAMOND_BLACKFAN_ANEMIA_MYELO	0,01	0,778	0,2363	1
chr17p13	-0,01	0,779	0,38777	1
GILMORE_CORE_NFKB_PATHWAY	0,01	0,78	0,93232	1
REACTOME_SYNTHESIS_OF_SUBSTRATES_IN_	-0,01	0,78	0,9852	1
WIEMANN_TELOMERE_SHORTENING_AND_CHI	0,01	0,781	0,42342	1
BIOCARTA_PS1_PATHWAY	-0,01	0,782	0,65139	1
BIOCARTA_FEEDER_PATHWAY	-0,01	0,782	0,19929	1
REACTOME_NUCLEAR_EVENTS_KINASE_AND_	0,01	0,783	0,86653	1
LOPEZ_MBD_TARGETS_IMPRINTED_AND_X_LI	0,01	0,784	0,44633	1
BARRIER_CANCER_RELAPSE_NORMAL_SAMPL	-0,01	0,784	0,13619	1
SCHURINGA_STAT5A_TARGETS_DN	0,01	0,784	0,65867	1
YORDY_RECIPROCAL_REGULATION_BY_ETS1_	-0,01	0,784	0,54568	1
GNF2_CASP1	0,01	0,785	0,87335	1
GRANDVAUX_IRF3_TARGETS_UP	-0,01	0,786	0,92326	1
CREIGHTON_ENDOCRINE_THERAPY_RESISTANC	-0,01	0,786	0,01234	1
NEGATIVE_REGULATION_OF_CELL_ADHESION	-0,01	0,786	0,77433	1
MONOCARBOXYLIC_ACID_TRANSMEMBRANE_T	0,01	0,787	0,8126	1
chr9q34	-0,01	0,788	0,10436	1

GSE29618_MONOCYTE_VS_MDC_DAY7_FLU_	0,01	0,788	0,58189	1
NIKOLSKY_BREAST_CANCER_5P15_AMPLICON	-0,01	0,788	0,56428	1
RESPONSE_TO_NUTRIENT_LEVELS	0,01	0,79	0,05565	1
chr5q31	0,01	0,79	0,31053	1
BALDWIN_PRKCI_TARGETS_UP	-0,01	0,79	0,45768	1
HYDROLASE_ACTIVITY_ACTING_ON_GLYCOSY	0,01	0,792	0,5189	1
PRAMOONJAGO_SOX4_TARGETS_UP	0,01	0,793	0,51328	1
CAFFAREL_RESPONSE_TO_THC_24HR_3_UP	0,01	0,793	0,81807	1
PHOSPHOINOSITIDE_BINDING	-0,01	0,793	0,29888	1
KORKOLA_CHORIOCARCINOMA_DN	0,01	0,794	0,2926	1
OSMAN_BLADDER_CANCER_UP	-0,009	0,796	0,99472	1
GERHOLD_ADIPOGENESIS_UP	-0,009	0,796	0,158	1
REGULATION_OF_PROTEIN_SECRETION	0,009	0,797	0,29716	1
GSE39820_CTRL_VS_TGFBETA3_IL6_CD4_T	0,009	0,797	0,28991	1
SMIRNOV_CIRCULATING_ENDOTHELIOCYTES_I	0,009	0,798	0,75592	1
GCM_DLG1	-0,009	0,799	0,60966	1
LIGAND_DEPENDENT_NUCLEAR_RECEPTOR_A	0,009	0,799	0,00842	1
BREDEMEYER_RAG_SIGNALING_VIA_ATM_NOT	0,009	0,8	0,12056	1
GNF2_HLA-C	-0,009	0,8	0,71752	1
INGRAM_SHH_TARGETS	-0,009	0,8	0,08438	1
CYTAGCAAY_UNKNOWN	-0,009	0,8	0,00035	1
SU_PANCREAS	0,009	0,8	0,16764	1
WATANABE_ULCERATIVE_COLITIS_WITH_CAN	-0,009	0,801	0,23736	1
PID_HDAC_CLASSI_PATHWAY	-0,009	0,802	0,163	1
STEROID_BINDING	0,009	0,802	0,36434	1
REGULATION_OF_RAS_GTPASE_ACTIVITY	-0,009	0,803	0,46919	1
WHITESIDE_CISPLATIN_RESISTANCE_UP	0,009	0,804	0,25792	1
REACTOME_INFLAMMASOMES	0,009	0,805	0,97978	1
ABDULRAHMAN_KIDNEY_CANCER_VHL_DN	-0,009	0,806	0,87896	1
ORGANELLE_OUTER_MEMBRANE	0,009	0,806	0,81557	1
SU_LIVER	0,009	0,808	0,13898	1
RUTELLA_RESPONSE_TO_HGF_VS_CSF2RB_A	-0,009	0,808	0,92273	1
MODULE_58	0,009	0,808	0,8	1
MODULE_173	0,009	0,808	0,29677	1
CATION_TRANSPORTING_ATPASE_ACTIVITY	0,009	0,81	0,49001	1
CORNIFIED_ENVELOPE	-0,009	0,81	0,14213	1
WANG_ADIPOGENIC_GENES_REPRESSED_BY_S	-0,009	0,811	0,72178	1
ZHAN_EARLY_DIFFERENTIATION_GENES_UP	0,009	0,811	0,94283	1
chr6q22	-0,009	0,812	0,96186	1
RESPIRATORY_GASEOUS_EXCHANGE	-0,009	0,812	0,52927	1
REACTOME_ANTIGEN_PRESENTATION_FOLDIN	-0,009	0,812	0,46736	1
BERNARD_PPAPDC1B_TARGETS_UP	-0,009	0,814	0,95106	1

RUAN_RESPONSE_TO_TROGLITAZONE_UP	-0,009	0,815	0,17332	1
GSE25087_FETAL_VS_ADULT_TCONV_DN	-0,009	0,815	0,02384	1
GNF2_TNFRSF1B	0,009	0,816	0,86747	1
MITOCHONDRIAL_MEMBRANE_PART	-0,009	0,816	0,99786	1
BROWN_MYELOID_CELL_DEVELOPMENT_UP	-0,009	0,816	0,98705	1
NAISHIRO_CTNNB1_TARGETS_WITH_LEF1_MO	0,009	0,816	0,67739	1
GSE360_DC_VS_MAC_L_DONOVANI_DN	0,009	0,816	0,86908	1
PID_P38ALPHABETADOWNSTREAMPATHWAY	-0,008	0,817	0,31965	1
MORF_UBE2A	-0,008	0,818	0,33769	1
SINGH_NFE2L2_TARGETS	0,008	0,818	0,07753	1
SPERMATID_DIFFERENTIATION	-0,008	0,819	0,12132	1
RUAN_RESPONSE_TO_TNF_UP	0,008	0,82	0,69092	1
MORF_JAG1	-0,008	0,821	0,04607	1
REACTOME_CALNEXIN_CALRETICULIN_CYCLE	0,008	0,822	0,19954	1
UNFOLDED_PROTEIN_RESPONSE	-0,008	0,823	0,78157	1
PROTEIN_POLYUBIQUITINATION	0,008	0,823	0,6565	1
GSE3982_CTRL_VS_LPS_1H_NEUTROPHIL_UI	0,008	0,823	0,70804	1
BARIS_THYROID_CANCER_UP	-0,008	0,824	0,80113	1
KEGG_PARKINSONS_DISEASE	0,008	0,824	0,68806	1
GCM_CRKL	-0,008	0,827	0,8779	1
RIGGINS_TAMOXIFEN_RESISTANCE_DN	0,008	0,827	0,03728	1
MODULE_557	0,008	0,828	0,3865	1
MODULE_143	0,008	0,828	0,63754	1
REACTOME_MEMBRANE_TRAFFICKING	0,008	0,829	0,11952	1
GSE6269_HEALTHY_VS_STREP_AUREUS_INF_	-0,008	0,829	0,66141	1
KIM_ALL_DISORDERS_OLIGODENDROCYTE_NU	-0,008	0,83	0,74434	1
ENDOTHELIAL_CELL_PROLIFERATION	0,008	0,83	0,56456	1
NEGATIVE_REGULATION_OF_CYTOKINE_BIOSY	0,008	0,83	0,72598	1
PID_CERAMIDE_PATHWAY	-0,008	0,83	0,11404	1
RUAN_RESPONSE_TO_TNF_TROGLITAZONE_U	0,008	0,831	0,70328	1
HOSHIDA_LIVER_CANCER_SUBCLASS_S3	0,008	0,832	0,31297	1
BIOCARTA_KREB_PATHWAY	-0,008	0,834	0,12826	1
PHOSPHATASE_BINDING	0,008	0,834	0,71755	1
MOSERLE_IFNA_RESPONSE	-0,008	0,835	0,86899	1
PHOSPHOTRANSFERASE_ACTIVITY_PHOSPHAT	0,008	0,836	0,06432	1
MIKKELSEN_ES_HCP_WITH_H3K27ME3	-0,008	0,836	0,27194	1
IGARASHI_ATF4_TARGETS_DN	-0,007	0,839	0,53548	1
GSE22886_NEUTROPHIL_VS_DC_DN	-0,007	0,839	0,15545	1
MODULE_505	0,007	0,839	0,98287	1
REGULATION_OF_TRANSCRIPTION_FACTOR_A	-0,007	0,84	0,33574	1
KEGG_MATURITY_ONSET_DIABETES_OF_THE_	0,007	0,84	0,91687	1
BIOCARTA_GRANULOCYTES_PATHWAY	-0,007	0,84	0,8234	1

MEISSNER_ES_ICP_WITH_H3K4ME3	-0,007	0,841	0,06357	1
GSE11057_CD4_CENT_MEM_VS_PBMC_DN	0,007	0,841	0,87914	1
CROONQUIST_NRAS_SIGNALING_UP	0,007	0,842	0,91618	1
PIGMENT_METABOLIC_PROCESS	-0,007	0,842	0,48995	1
CAMPS_COLON_CANCER_COPY_NUMBER_UP	-0,007	0,843	0,28427	1
GSE2706_LPS_VS_R848_AND_LPS_8H_STIM	-0,007	0,844	0,68329	1
KEGG_ASTHMA	-0,007	0,844	0,55314	1
ANION_CATION_SYMPORTER_ACTIVITY	-0,007	0,844	0,30189	1
BOWIE_RESPONSE_TO_EXTRACELLULAR_MAT	0,007	0,845	0,74953	1
BOHN_PRIMARY_IMMUNODEFICIENCY_SYNDRO	0,007	0,845	0,90365	1
JISON_SICKLE_CELL_DISEASE_UP	-0,007	0,845	0,99423	1
IKEDA_MIR1_TARGETS_DN	-0,007	0,846	0,49853	1
REACTOME_ABC_FAMILY_PROTEINS_MEDIATE	-0,007	0,846	0,00763	1
RUAN_RESPONSE_TO_TNF_TROGLITAZONE_D	-0,007	0,847	0,09961	1
KEGG_FATTY_ACID_METABOLISM	-0,007	0,848	0,10401	1
ANTIOXIDANT_ACTIVITY	0,007	0,848	0,78097	1
GSE13485_DAY1_VS_DAY21_YF17D_VACCII	-0,007	0,849	0,67457	1
REACTOME_DEFENSINS	-0,007	0,849	0,93378	1
OXIDOREDUCTASE_ACTIVITY_ACTING_ON_TH	0,007	0,849	0,32548	1
FOCAL_ADHESION	0,007	0,849	0,76298	1
INTERCELLULAR_JUNCTION_ASSEMBLY_AND_	-0,007	0,85	0,19955	1
KAAB_FAILED_HEART_ATRIUM_UP	-0,007	0,85	0,29858	1
MYLLYKANGAS_AMPLIFICATION_HOT_SPOT_5	-0,007	0,85	0,0937	1
EINAV_INTERFERON_SIGNATURE_IN_CANCER	-0,007	0,851	0,85126	1
INORGANIC_CATION_TRANSMEMBRANE_TRAN	0,007	0,852	0,95177	1
GSE24634_TEFF_VS_TCONV_DAY10_IN_CUL	0,007	0,852	0,68055	1
CELL_SUBSTRATE_ADHERENS_JUNCTION	-0,007	0,853	0,45997	1
chr20q11	0,007	0,853	0,73645	1
GCM_SUFU	0,007	0,853	0,6411	1
OUYANG_PROSTATE_CANCER_MARKERS	0,007	0,853	0,00318	1
BIOCARTA_HCMV_PATHWAY	-0,007	0,855	0,82392	1
SCHUHMACHER_MYC_TARGETS_DN	-0,007	0,856	0,48269	1
BILE_ACID_METABOLIC_PROCESS	-0,007	0,856	0,89919	1
RESPONSE_TO_EXTRACELLULAR_STIMULUS	0,007	0,857	0,04324	1
ORGANIC_ACID_TRANSPORT	0,007	0,858	0,7987	1
chr5p14	-0,007	0,858	0,22616	1
REACTOME_MITOCHONDRIAL_PROTEIN_IMPOR	-0,007	0,859	0,60564	1
REACTOME_BMAL1_CLOCK_NPAS2_ACTIVAT	-0,007	0,859	0,26851	1
CAFFAREL_RESPONSE_TO_THC_8HR_5_UP	0,006	0,86	0,87005	1
GRABARCZYK_BCL11B_TARGETS_UP	-0,006	0,86	0,67165	1
STEROL_BINDING	0,006	0,86	0,22387	1
PROTEIN_AUTOPROCESSING	-0,006	0,861	0,43692	1

MODULE_294	-0,006	0,861	0,13079	1
COATES_MACROPHAGE_M1_VS_M2_DN	-0,006	0,862	0,87908	1
HOFMANN_MYELODYSPLASTIC_SYNDROM_RIS	0,006	0,863	0,41739	1
MODULE_464	-0,006	0,863	0,21082	1
VOLTAGE_GATED_CALCIUM_CHANNEL_ACTIV	0,006	0,863	0,01181	1
REACTOME_ACTIVATED_AMPK_STIMULATES_	-0,006	0,863	0,15808	1
ERB2_UP.V1_UP	-0,006	0,863	0,86503	1
TBK1.DN.48HRS_DN	0,006	0,863	0,26572	1
chr4p16	-0,006	0,864	0,00848	1
HOWLIN_CITED1_TARGETS_2_UP	-0,006	0,865	0,26373	1
SCHRAMM_INHBA_TARGETS_UP	-0,006	0,865	0,34111	1
GNF2_MYL2	0,006	0,865	0,38322	1
COFACTOR_CATABOLIC_PROCESS	0,006	0,865	0,90499	1
REACTOME_INCRETIN_SYNTHESIS_SECRETION	0,006	0,867	0,74517	1
KEGG_CARDIAC_MUSCLE_CONTRACTION	0,006	0,868	0,10265	1
REACTOME_GLYCOGEN_BREAKDOWN_GLYCOC	0,006	0,868	0,59978	1
KORKOLA_TERATOMA_UP	0,006	0,868	0,86953	1
VACUOLAR_MEMBRANE	-0,006	0,868	0,90522	1
GSE37416_CTRL_VS_3H_F_TULARENSIS_LVS	-0,006	0,868	0,24734	1
SUGAR_BINDING	-0,006	0,868	0,49233	1
BIOCARTA_EGFR_SMRTE_PATHWAY	0,006	0,869	0,10026	1
MODULE_327	0,006	0,869	0,2931	1
NUCLEOBASENUCLEOSIDENUCLEOTIDE_KINASI	0,006	0,869	0,11917	1
UBIQUITIN_PROTEIN_LIGASE_ACTIVITY	-0,006	0,87	0,46048	1
REACTOME_GLUTATHIONE_CONJUGATION	-0,006	0,87	0,73977	1
REACTOME_CYTOSOLIC_SULFONATION_OF_SI	-0,006	0,871	0,85005	1
KEGG_STARCH_AND_SUCROSE_METABOLISM	0,006	0,871	0,48552	1
RODRIGUES_DCC_TARGETS_DN	-0,006	0,872	0,1165	1
DIERICK_SEROTONIN_FUNCTION_GENES	0,006	0,873	0,27199	1
chr2p14	0,006	0,873	0,58837	1
GCM_CALM1	-0,006	0,874	0,31725	1
DELLA_RESPONSE_TO_TSA_AND_BUTYRATE	0,006	0,874	0,87565	1
SA_MMP_CYTOKINE_CONNECTION	-0,006	0,875	0,91644	1
REGULATION_OF_I_KAPPAB_KINASE_NF_KAP	-0,006	0,876	0,51643	1
AGTGCGT,MIR-521	-0,006	0,876	0,40574	1
SUZUKI_RESPONSE_TO_TSA	0,006	0,876	0,76099	1
CROONQUIST_IL6_DEPRIVATION_UP	0,006	0,876	0,19253	1
REACTOME_PKA_MEDIATED_PHOSPHORYLATI	0,006	0,877	0,05708	1
BENNETT_SYSTEMIC_LUPUS_ERYTHEMATOSUS	-0,006	0,877	0,65141	1
RAMJAUN_APOPTOSIS_BY_TGFB1_VIA_MAPK	0,006	0,879	0,86923	1
PARK_HSC_VS_MULTIPOTENT_PROGENITORS_	0,006	0,88	0,88373	1
STEARMAN_LUNG_CANCER_EARLY_VS_LATE_	0,006	0,88	0,98797	1

KEGG_GLUTATHIONE_METABOLISM	-0,006	0,88	0,75543	1
V$SEF1_C	-0,006	0,881	0,73397	1
PID_HIF2PATHWAY	-0,006	0,881	0,60533	1
RUAN_RESPONSE_TO_TNF_DN	0,005	0,881	0,2343	1
chr2q34	-0,005	0,882	0,15305	1
chr3q	-0,005	0,882	0,96216	1
VITAMIN_BINDING	0,005	0,882	0,46912	1
MODULE_306	0,005	0,882	0,07646	1
MITOCHONDRIAL_SMALL_RIBOSOMAL_SUBUN	-0,005	0,882	0,66333	1
ORGANELLAR_SMALL_RIBOSOMAL_SUBUNIT	-0,005	0,882	0,66333	1
SMALL_RIBOSOMAL_SUBUNIT	-0,005	0,882	0,66333	1
GSE27786_LIN_NEG_VS_NKCELL_DN	-0,005	0,882	0,0563	1
SMAD_BINDING	0,005	0,882	0,0905	1
GSE17721_PAM3CSK4_VS_GADIQUIMOD_2H_	-0,005	0,884	0,27477	1
GSE2706_2H_VS_8H_R848_AND_LPS_STIM_	0,005	0,884	0,58099	1
REACTOME_RECYCLING_OF_BILE_ACIDS_AND	0,005	0,885	0,69568	1
GSE360_CTRL_VS_T_GONDII_MAC_UP	-0,005	0,888	0,54989	1
PID_HNF3BPATHWAY	-0,005	0,888	0,00488	1
REACTOME_METABOLISM_OF_VITAMINS_AND	0,005	0,889	0,10277	1
COPPER_ION_BINDING	0,005	0,889	0,44981	1
chr16p	0,005	0,89	0,73653	1
BOGNI_TREATMENT_RELATED_MYELOID_LEUK	0,005	0,89	0,13388	1
MORF_RAB1A	0,005	0,891	0,11179	1
SMALL_CONJUGATING_PROTEIN_LIGASE_ACT	-0,005	0,893	0,4763	1
KEGG_PRIMARY_BILE_ACID_BIOSYNTHESIS	-0,005	0,893	0,37435	1
MCCABE_HOXC6_TARGETS_CANCER_DN	-0,005	0,893	0,22931	1
WANG_BARRETTS_ESOPHAGUS_AND_ESOPHA	0,005	0,893	0,24563	1
GSE17721_CTRL_VS_LPS_4H_BMDM_UP	-0,005	0,893	0,18656	1
SEIKE_LUNG_CANCER_POOR_SURVIVAL	-0,005	0,893	0,38097	1
HEDENFALK_BREAST_CANCER_BRACX_DN	-0,005	0,893	0,50593	1
RAY_ALZHEIMERS_DISEASE	-0,005	0,894	0,29327	1
SUPEROXIDE_METABOLIC_PROCESS	0,005	0,894	0,974	1
GLUCAN_METABOLIC_PROCESS	-0,005	0,896	0,49518	1
U12_DEPENDENT_SPLICEOSOME	0,005	0,897	0,98791	1
CHESLER_BRAIN_QTL_TRANS	-0,005	0,898	0,51219	1
GSE22886_NAIVE_BCELL_VS_NEUTROPHIL_D	-0,005	0,901	0,6307	1
GNF2_TYK2	-0,005	0,901	0,76671	1
BIOCARTA_GATA3_PATHWAY	0,005	0,902	0,2512	1
LIU_BREAST_CANCER	0,004	0,903	0,80812	1
BIOCARTA_NUCLEARRS_PATHWAY	-0,004	0,904	0,14542	1
PROTEIN_METHYLTRANSFERASE_ACTIVITY	0,004	0,905	0,19295	1
GSE17721_0.5H_VS_8H_PAM3CSK4_BMDM_	-0,004	0,906	0,08821	1

GCM_BCL2L1	-0,004	0,907	0,89038	1
GNF2_ANK1	0,004	0,907	0,94724	1
GNF2_SPTB	0,004	0,907	0,94724	1
SPERMATID_DEVELOPMENT	0,004	0,909	0,22179	1
REACTOME_PHOSPHOLIPID_METABOLISM	-0,004	0,909	0,11013	1
GSE11864_CSF1_VS_CSF1_PAM3CYS_IN_MA	0,004	0,909	0,22337	1
CELLULAR_LIPID_CATABOLIC_PROCESS	0,004	0,909	0,55646	1
KEGG_SELENOAMINO_ACID_METABOLISM	0,004	0,91	0,48457	1
STEIN_ESTROGEN_RESPONSE_NOT_VIA_ESRR	0,004	0,91	0,00045	1
PROTEIN_SECRETION	0,004	0,91	0,32492	1
SUGAR_TRANSMEMBRANE_TRANSPORTER_AC	-0,004	0,911	0,36024	1
NOUZOVA_TRETINOIN_AND_H4_ACETYLATIO	-0,004	0,911	0,97618	1
GROSS_HYPOXIA_VIA_HIF1A_ONLY	0,004	0,912	0,321	1
REACTOME_IL1_SIGNALING	-0,004	0,912	0,55929	1
GNF2_MYL3	-0,004	0,912	0,26157	1
BOYAULT_LIVER_CANCER_SUBCLASS_G6_DN	0,004	0,912	0,50719	1
REACTOME_TRYPTOPHAN_CATABOLISM	-0,004	0,912	0,28243	1
PARK_TRETINOIN_RESPONSE_AND_PML_RAR/	0,004	0,912	0,87027	1
KEGG_METABOLISM_OF_XENOBIOTICS_BY_CY	0,004	0,913	0,95739	1
MITOCHONDRION	-0,004	0,913	0,55333	1
FARMER_BREAST_CANCER_APOCRINE_VS_LU	-0,004	0,913	0,12954	1
ENDOPLASMIC_RETICULUM_MEMBRANE	0,004	0,913	0,47853	1
GCM_RAB10	-0,004	0,913	0,86991	1
RUNNE_GENDER_EFFECT_UP	0,004	0,914	0,43219	1
GSE3982_DC_VS_TH1_UP	0,004	0,914	0,79022	1
MORF_DAP	-0,004	0,914	0,25192	1
AMINO_ACID_METABOLIC_PROCESS	0,004	0,915	0,3036	1
MATTIOLI_MGUS_VS_MULTIPLE_MYELOMA	-0,004	0,916	0,44168	1
BOYAULT_LIVER_CANCER_SUBCLASS_G3_DN	0,004	0,916	0,06426	1
GNF2_FOS	0,004	0,918	0,69185	1
MODULE_424	0,004	0,918	0,60783	1
NUCLEOTIDE_KINASE_ACTIVITY	-0,004	0,919	0,05213	1
AIYAR_COBRA1_TARGETS_UP	0,004	0,919	0,18216	1
REACTOME_RORA_ACTIVATES_CIRCADIAN_E)	0,004	0,92	0,93337	1
PEDERSEN_METASTASIS_BY_ERBB2_ISOFORM	0,004	0,92	0,49203	1
COFACTOR_METABOLIC_PROCESS	0,004	0,921	0,34667	1
UROSEVIC_RESPONSE_TO_IMIQUIMOD	0,004	0,921	0,71602	1
GSE24634_TEFF_VS_TCONV_DAY7_IN_CULT	0,004	0,921	0,95744	1
CGGAARNGGCNG_UNKNOWN	0,004	0,922	0,14884	1
OXFORD_RALA_AND_RALB_TARGETS_DN	-0,004	0,922	0,60706	1
GSE6269_HEALTHY_VS_STREP_PNEUMO_INF	-0,004	0,923	0,74687	1
BIOCARTA_ARAP_PATHWAY	0,004	0,923	0,46419	1

WANG_IMMORTALIZED_BY_HOXA9_AND_MEIS	-0,004	0,924	0,6635	1
GNF2_TAL1	0,003	0,925	0,96083	1
FRASOR_RESPONSE_TO_SERM_OR_FULVESTR	-0,003	0,926	0,40578	1
SPIELMAN_LYMPHOBLAST_EUROPEAN_VS_AS	-0,003	0,926	0,81566	1
MITOCHONDRIAL_RIBOSOME	-0,003	0,926	0,75703	1
ORGANELLAR_RIBOSOME	-0,003	0,926	0,75703	1
chrxq28	-0,003	0,926	0,13947	1
KEGG_GLYCINE_SERINE_AND_THREONINE_ME	0,003	0,926	0,62338	1
MULLIGHAN_MLL_SIGNATURE_2_UP	-0,003	0,927	0,49612	1
ZHENG_FOXP3_TARGETS_IN_THYMUS_DN	0,003	0,927	0,00366	1
REACTOME_MAPK_TARGETS_NUCLEAR_EVEN	0,003	0,928	0,65672	1
MODULE_402	-0,003	0,928	0,5389	1
GSE10325_BCELL_VS_MYELOID_DN	0,003	0,928	0,9618	1
GSE360_T_GONDII_VS_B_MALAYI_HIGH_DOS	0,003	0,93	0,34116	1
chrxq24	0,003	0,93	0,3937	1
IIZUKA_LIVER_CANCER_PROGRESSION_G2_G3	-0,003	0,931	0,6198	1
GSE24634_TREG_VS_TCONV_POST_DAY10_	0,003	0,931	0,72805	1
REACTOME_ACYL_CHAIN_REMODELLING_OF_	0,003	0,932	0,2987	1
MODULE_194	-0,003	0,932	0,26483	1
SEIDEN_MET_SIGNALING	0,003	0,932	0,79323	1
GSE3982_DC_VS_CENT_MEMORY_CD4_TCEL	0,003	0,932	0,9504	1
REACTOME_CITRIC_ACID_CYCLE_TCA_CYCLE	0,003	0,933	0,12628	1
MODULE_247	0,003	0,933	0,82745	1
TRANS_GOLGI_NETWORK	-0,003	0,935	0,79155	1
PROTEIN_AMINO_ACID_O_LINKED_GLYCOSYL	-0,003	0,935	0,21864	1
GNF2_LYN	-0,003	0,935	0,79441	1
IIZUKA_LIVER_CANCER_PROGRESSION_L1_G1	-0,003	0,936	0,44108	1
ACOSTA_PROLIFERATION_INDEPENDENT_MYC	-0,003	0,936	0,5027	1
MATZUK_PREOVULATORY_FOLLICLE	0,003	0,938	0,00639	1
POSITIVE_REGULATION_OF_PROTEIN_SECRETI	0,003	0,938	0,49828	1
LTE2_UP.V1_UP	0,003	0,938	0,93367	1
BIOCARTA_IL5_PATHWAY	-0,003	0,938	0,98019	1
GSE24634_TREG_VS_TCONV_POST_DAY5_IL	0,003	0,938	0,87323	1
POSITIVE_REGULATION_OF_CYTOKINE_SECRE	0,003	0,939	0,50144	1
KEGG_BETA_ALANINE_METABOLISM	-0,003	0,939	0,32838	1
GSE17721_CTRL_VS_PAM3CSK4_1H_BMDM_	0,003	0,94	0,78197	1
FAELT_B_CLL_WITH_VH3_21_DN	-0,003	0,94	0,74658	1
HUMMEL_BURKITTS_LYMPHOMA_DN	0,003	0,941	0,98643	1
CARBOXYLIC_ACID_TRANSPORT	0,003	0,941	0,88455	1
LINDGREN_BLADDER_CANCER_WITH_LOH_IN_	-0,003	0,942	0,19246	1
MODULE_416	0,003	0,942	0,36737	1
RESPONSE_TO_ORGANIC_SUBSTANCE	0,003	0,942	0,61088	1

REACTOME_CIRCADIAN_CLOCK	-0,003	0,942	0,26128	1
HUMMERICH_BENIGN_SKIN_TUMOR_DN	-0,003	0,942	0,35967	1
WENG_POR_TARGETS_GLOBAL_DN	0,003	0,942	0,32696	1
REACTOME_TRAF6_MEDIATED_IRF7_ACTIVAT	-0,003	0,942	0,97987	1
BIOCARTA_IL10_PATHWAY	-0,003	0,943	0,96964	1
EARLY_ENDOSOME	-0,003	0,943	0,48307	1
DAZARD_UV_RESPONSE_CLUSTER_G1	-0,003	0,943	0,84962	1
DEFENSE_RESPONSE_TO_VIRUS	0,003	0,943	0,66672	1
MOOTHA_HUMAN_MITODB_6_2002	0,003	0,943	0,93889	1
chr5p13	-0,003	0,943	0,16276	1
REACTOME_RNA_POL_I_TRANSCRIPTION_INIT	-0,003	0,945	0,85105	1
KEGG_TYPE_I_DIABETES_MELLITUS	-0,002	0,946	0,89608	1
GSE24634_IL4_VS_CTRL_TREATED_NAIVE_(	-0,002	0,946	0,42952	1
PROTEIN_OLIGOMERIZATION	0,002	0,947	0,15967	1
GNF2_IGF1	-0,002	0,947	0,2525	1
REGULATION_OF_GTPASE_ACTIVITY	0,002	0,948	0,6032	1
GSE37416_12H_VS_24H_F_TULARENSIS_LV	0,002	0,949	0,58612	1
MORF_PRKAR1A	0,002	0,949	0,16155	1
NIKOLSKY_BREAST_CANCER_22Q13_AMPLIC(	-0,002	0,949	0,9071	1
LIPID_CATABOLIC_PROCESS	0,002	0,95	0,31776	1
GNF2_CEBPA	0,002	0,95	0,22776	1
TERAO_AOX4_TARGETS_HG_UP	-0,002	0,95	0,49284	1
RNA_POLYMERASE_II_TRANSCRIPTION_FACTC	-0,002	0,95	0,62019	1
GSE2706_UNSTIM_VS_8H_R848_DC_UP	0,002	0,95	0,067	1
GARCIA_TARGETS_OF_FLI1_AND_DAX1_UP	0,002	0,951	0,00972	1
OKAMOTO_LIVER_CANCER_MULTICENTRIC_O(	0,002	0,952	0,7564	1
TESAR_JAK_TARGETS_MOUSE_ES_D3_UP	0,002	0,952	0,5069	1
REACTOME_BETA_DEFENSINS	-0,002	0,952	0,94201	1
REACTOME_TRAF3_DEPENDENT_IRF_ACTIVAT	0,002	0,954	0,84109	1
GSE360_HIGH_DOSE_B_MALAYI_VS_M_TUBE	-0,002	0,955	0,57945	1
MIKKELSEN_MEF_LCP_WITH_H3K27ME3	0,002	0,955	0,08776	1
KORKOLA_EMBRYONAL_CARCINOMA_DN	0,002	0,957	0,27742	1
SECRETION	-0,002	0,957	0,15773	1
GSE29618_MONOCYTE_VS_PDC_DAY7_FLU_	-0,002	0,957	0,99881	1
FARDIN_HYPOXIA_9	0,002	0,958	0,04539	1
POSITIVE_REGULATION_OF_MAPKKK_CASCAD	0,002	0,958	0,14616	1
BRUSH_BORDER	-0,002	0,959	0,44219	1
MODULE_243	-0,002	0,959	0,00408	1
GSE29618_MONOCYTE_VS_MDC_UP	0,002	0,959	0,78112	1
CYTOKINE_SECRETION	0,002	0,96	0,28581	1
REACTOME_OPSINS	-0,002	0,96	0,22028	1
BEIER_GLIOMA_STEM_CELL_DN	-0,002	0,96	0,3023	1

REACTOME_BASE_FREE_SUGAR_PHOSPHATE_	-0,002	0,961	0,32	1
ACID_AMINO_ACID_LIGASE_ACTIVITY	0,002	0,962	0,78025	1
REACTOME_SYNTHESIS_AND_INTERCONVERSI	-0,002	0,963	0,52315	1
ACUTE_INFLAMMATORY_RESPONSE	0,002	0,964	0,39092	1
GSE10325_CD4_TCELL_VS_MYELOID_DN	-0,002	0,964	0,95041	1
GSE9037_CTRL_VS_LPS_4H_STIM_BMDM_UF	-0,002	0,964	0,09401	1
GSE2706_2H_VS_8H_R848_STIM_DC_UP	0,002	0,964	0,19334	1
GSE17721_CTRL_VS_LPS_8H_BMDM_UP	0,002	0,965	0,26171	1
ZEMBUTSU_SENSITIVITY_TO_DOXORUBICIN	0,002	0,965	0,70171	1
NEGATIVE_REGULATION_OF_TRANSCRIPTION_	0,002	0,966	0,83091	1
NEGATIVE_REGULATION_OF_CYTOSKELETON_	-0,002	0,966	0,50768	1
ESTABLISHMENT_OF_VESICLE_LOCALIZATION	-0,002	0,966	0,25069	1
MODULE_539	-0,002	0,966	0,68937	1
IIZUKA_LIVER_CANCER_PROGRESSION_G1_G2	0,002	0,967	0,72172	1
KEGG_VASOPRESSIN_REGULATED_WATER_RE	0,001	0,967	0,70912	1
GSE17721_CTRL_VS_CPG_6H_BMDM_UP	-0,001	0,968	0,08003	1
YAO_TEMPORAL_RESPONSE_TO_PROGESTERO	0,001	0,968	0,47544	1
GSE29618_BCELL_VS_MONOCYTE_DN	0,001	0,969	0,91768	1
MODULE_239	-0,001	0,969	0,70196	1
MORF_PRKACA	-0,001	0,97	0,06493	1
GSE11924_TFH_VS_TH17_CD4_TCELL_UP	0,001	0,97	0,01409	1
PAPASPYRIDONOS_UNSTABLE_ATEROSCLERO	-0,001	0,971	0,54503	1
MODULE_373	0,001	0,971	0,18747	1
MONOCARBOXYLIC_ACID_TRANSPORT	0,001	0,971	0,56003	1
MODULE_407	0,001	0,971	0,16812	1
BOWIE_RESPONSE_TO_TAMOXIFEN	0,001	0,971	0,89827	1
chr5q35	0,001	0,972	0,38766	1
MODULE_285	-0,001	0,972	0,22536	1
OXYGEN_AND_REACTIVE_OXYGEN_SPECIES_M	0,001	0,972	0,45463	1
WAMUNYOKOLI_OVARIAN_CANCER_LMP_UP	-0,001	0,973	0,33224	1
chr6q23	0,001	0,973	0,71156	1
REACTOME_TRAF6_MEDIATED_INDUCTION_OI	0,001	0,974	0,91689	1
AMIT_SERUM_RESPONSE_20_MCF10A	0,001	0,975	0,09628	1
ADHERENS_JUNCTION	-0,001	0,975	0,366	1
FOCAL_ADHESION_FORMATION	-0,001	0,975	0,52304	1
REACTOME_MEMBRANE_BINDING_AND_TARGE	-0,001	0,976	0,71315	1
chr5q21	-0,001	0,976	0,12887	1
GNF2_FGR	-0,001	0,978	0,99739	1
BERGER_MBD2_TARGETS	1E-03	0,978	0,32985	1
REGULATION_OF_CYTOKINE_SECRETION	9E-04	0,98	0,24065	1
VACUOLAR_PART	-9E-04	0,98	0,8401	1
OLSSON_E2F3_TARGETS_UP	9E-04	0,98	0,82411	1

chr1q41	9E-04	0,981	0,65644	1
REACTOME_RAP1_SIGNALLING	-9E-04	0,981	0,42638	1
WANG_RESPONSE_TO_GSK3_INHIBITOR_SB21	-9E-04	0,981	0,30014	1
STEROID_HORMONE_RECEPTOR_ACTIVITY	8E-04	0,982	0,03581	1
REACTOME_SIGNAL_ATTENUATION	8E-04	0,983	0,04632	1
GCM_ING1	7E-04	0,984	0,44507	1
KANNAN_TP53_TARGETS_UP	7E-04	0,985	0,10998	1
chr9p23	6E-04	0,986	0,83868	1
DISTECHE_ESCAPED_FROM_X_INACTIVATION	-6E-04	0,987	0,69513	1
BANDRES_RESPONSE_TO_CARMUSTIN_MGMT_	6E-04	0,987	0,62126	1
KEGG_PENTOSE_PHOSPHATE_PATHWAY	-6E-04	0,988	0,14006	1
MODULE_4	-6E-04	0,988	0,43384	1
MORF_MYC	5E-04	0,988	0,05513	1
MITOCHONDRIAL_LUMEN	5E-04	0,988	0,93366	1
MITOCHONDRIAL_MATRIX	5E-04	0,988	0,93366	1
MODULE_530	5E-04	0,989	0,29981	1
SOLUTE_SODIUM_SYMPORTER_ACTIVITY	5E-04	0,989	0,16463	1
RIGGINS_TAMOXIFEN_RESISTANCE_UP	5E-04	0,989	0,01157	1
BERNARD_PPAPDC1B_TARGETS_DN	-5E-04	0,99	0,02001	1
HEMATOPOIETIN_INTERFERON_CLASSD200_D	5E-04	0,99	0,76259	1
GLYCEROPHOSPHOLIPID_METABOLIC_PROCES	-4E-04	0,99	0,28013	1
NIKOLSKY_BREAST_CANCER_19Q13.1_AMPLI	4E-04	0,99	0,75334	1
BAKKER_FOXO3_TARGETS_UP	-4E-04	0,991	0,17324	1
DUTERTRE_ESTRADIOL_RESPONSE_6HR_DN	4E-04	0,991	0,23769	1
chr3p23	-4E-04	0,991	0,88843	1
GNF2_SPTA1	-4E-04	0,991	0,49652	1
AGTCAGC,MIR-345	-4E-04	0,991	0,25604	1
REACTOME_ROLE_OF_SECOND_MESSENGERS_	-3E-04	0,993	0,78972	1
ZAMORA_NOS2_TARGETS_DN	3E-04	0,993	0,78288	1
LEE_LIVER_CANCER	3E-04	0,994	0,06912	1
REACTOME_GABA_A_RECEPTOR_ACTIVATION	-3E-04	0,994	0,32453	1
MARTINEZ_RESPONSE_TO_TRABECTEDIN	2E-04	0,995	0,58769	1
GSE12845_IGD_NEG_BLOOD_VS_PRE_GC_TC	-2E-04	0,995	0,17917	1
PID_RXR_VDR_PATHWAY	-2E-04	0,995	0,05831	1
JOSEPH_RESPONSE_TO_SODIUM_BUTYRATE_I	-2E-04	0,995	0,90038	1
PHOSPHOLIPID_METABOLIC_PROCESS	-2E-04	0,995	0,23697	1
GSE14000_UNSTIM_VS_16H_LPS_DC_TRANS	-2E-04	0,996	0,14492	1
GCM_TINF2	2E-04	0,996	0,24744	1
GSE37416_0H_VS_6H_F_TULARENSIS_LVS_	-1E-04	0,997	0,61586	1
GSE24634_TREG_VS_TCONV_POST_DAY7_IL	-1E-04	0,997	0,93725	1
GSE17721_4H_VS_24H_POLYIC_BMDM_DN	-1E-04	0,997	0,99473	1
REACTOME_INSULIN_SYNTHESIS_AND_PROCE	1E-04	0,998	0,93971	1

WAMUNYOKOLI_OVARIAN_CANCER_GRADES_	9E-05	0,998	0,81776	1
LIAN_NEUTROPHIL_GRANULE_CONSTITUENTS	-8E-05	0,998	0,8693	1
LIU_TARGETS_OF_VMYB_VS_CMYB_UP	-5E-05	0,999	0,15549	1
KEGG_PPAR_SIGNALING_PATHWAY	3E-05	0,999	0,3729	1
REACTOME_RESOLUTION_OF_AP_SITES_VIA_	2E-05	1	0,29862	1
GKCGCNNNNNNNTGAYG_UNKNOWN	-1E-05	1	0,63394	1
